# Directional Ionic Bonds

**DOI:** 10.1021/jacs.3c01030

**Published:** 2023-04-07

**Authors:** Illia Hutskalov, Anthony Linden, Ilija Čorić

**Affiliations:** Department of Chemistry, University of Zurich, Winterthurerstrasse 190, CH-8057 Zurich, Switzerland

## Abstract

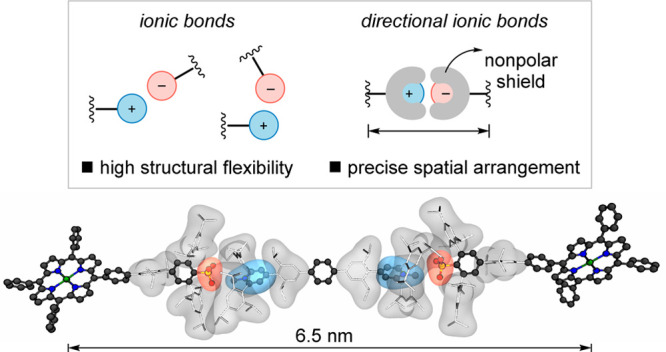

Covalent and ionic bonds represent two fundamental forms of bonding between atoms. In contrast to bonds with significant covalent character, ionic bonds are of limited use for the spatial structuring of matter because of the lack of directionality of the electric field around simple ions. We describe a predictable directional orientation of ionic bonds that contain concave nonpolar shields around the charged sites. Such directional ionic bonds offer an alternative to hydrogen bonds and other directional noncovalent interactions for the structuring of organic molecules and materials.

The spatial orientation of bonding orbitals imparts directionality to bonds with significant covalent character,^1−3^ thereby enabling the three-dimensional structuring of complex molecules.^4−16^ Next to the directionality of covalent bonds, the presence of directional noncovalent interactions is often essential for the structure of biological and synthetic organic matter. Most notably, the directionality of hydrogen bonds is relevant for the base pairing in DNA and for other molecular recognition phenomena in biology and chemistry^17^ and it is a ubiquitous design element for synthetic supramolecular chemistry.^18−25^ In comparison with hydrogen bonds, the utility of other directional noncovalent interactions, of which halogen bonds are the main representative, is more limited.^26−30^ Here, we focus on imparting directionality to ionic bonds in an attempt to provide a distinct directional noncovalent interaction for the three-dimensional structuring of matter. Ionic bonds are present in ionic solids, such as sodium chloride, and are relevant for the structure of organic materials,^31−34^ synthetic supramolecular assemblies,^35,36^ and biological molecules, including DNA^37^ and proteins.^38^ In polar solvents, because of the solvation of ions, ionic bonds behave as labile noncovalent interactions and are energetically comparable to hydrogen bonds.^39−41^ Furthermore, the binding of two separated ions in vacuum or the presence of multiple ionic interactions, even in water, can be energetically comparable to the strength of covalent bonds.^41,42^ However, the lack of the dependence of the electrostatic attraction on the relative orientation of the cation and the anion results in the structural flexibility of ionic bonds (Figure 1a; see Figure 1b for the comparison with covalent bonding). As a result, ionic bonds are of limited use for directional connectivity,^36,43−46^ unless oriented by multiple interactions,^47,48^ hydrogen bonding,^19,33,36,49−59^ planar π-systems,^60−62^ or metal coordination.^63^

**Figure 1:**
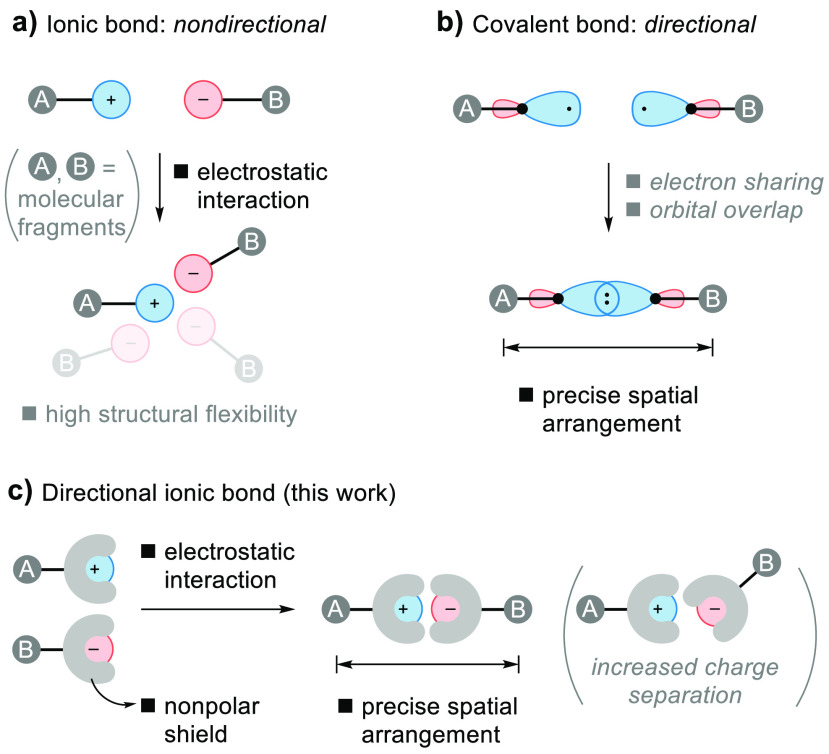
Design of directional ionic bonds.

Our strategy for the design of directional ionic bonds involves the placement of ions in a sterically demanding, shielding, nonpolar hydrocarbon environment that leaves the charged group exposed in one direction only (Figure 1c). A directional approach of two oppositely charged ions with such structure from the sterically accessible sides minimizes the charge separation and maximizes the Coulomb attraction. Other relative orientations result in larger separations because of the steric repulsion by the shielding backbone and are, therefore, less favorable (Figure 1c). Directional ionic bonds might display directionality in solution and in the solid state, thus offering a new strategy for building molecules, supramolecular assemblies, and materials.

For the demonstration of the concept of directional ionic bonds, we selected *N*-methylpyridinium cations and arylsulfonate anions because of their potential for structural modifications at the carbon sites. The structural requirements for the construction of an effective nonpolar shield around the ionic moieties were studied by the introduction of sterically demanding 2,6-bisisopropylphenyl and 2,4,6-trisisopropylphenyl substituents at the positions 4-, 2,6-, or 2,4,6- of the central (hetero)arene groups of the two ions (Figure 2a). The examination of the electrostatic potential maps of the ions that contain the shielding group placed in the 4-position only (**C2**^**+**^ and **A2**^**–**^) reveals little impact on the directionality of the approach to the charged site. However, a significant shielding of the ionic moieties is achieved when the nonpolar groups are placed in positions 2- and 6- (**C3**^**+**^ and **A3**^**–**^). Because of the localization of the negative charge, the −SO_3_^–^ group appears similarly shielded by the nonpolar environments in **A3**^**–**^ and **A4**^–^. However, for the shielding of the *N*-methylpyridinium cation, in which the positive charge is delocalized across the aromatic ring, the presence of nonpolar groups in all three positions (2,4,6-) is necessary to ensure that the charged site is exposed only from the side with the *N*-CH_3_ group (**C3**^**+**^ vs **C4**^**+**^).

**Figure 2:**
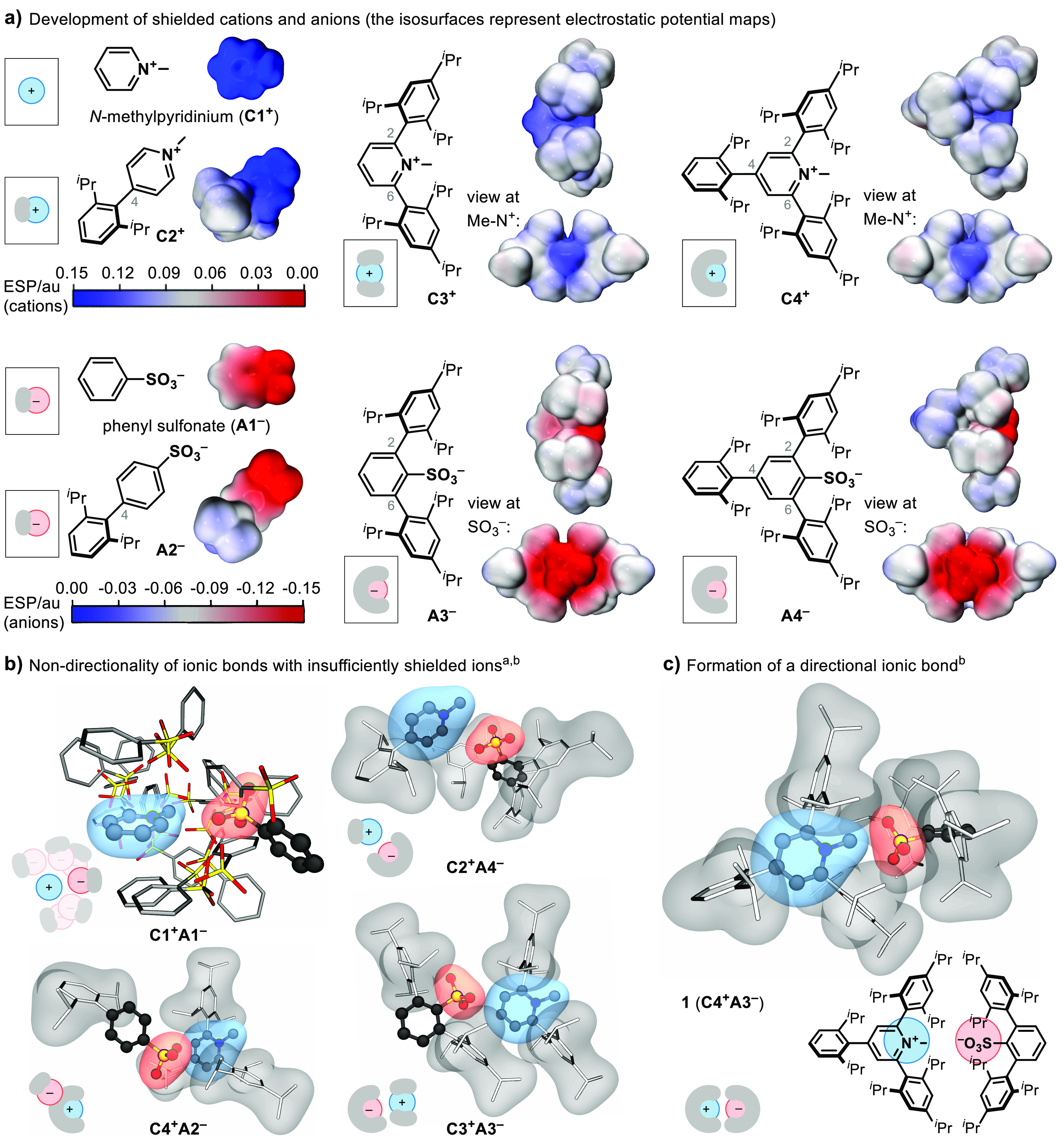
Toward directional ionic bonds. ^a^Under **C1**^**+**^**A1**^**–**^, the truncated geometries of a subset of previously reported substituted *N*-methylpyridinium arylsulfonates are shown. ^b^The structures are experimental solid-state molecular structures. Hydrogen atoms are omitted for clarity, and the transparent surfaces emphasize the charged (blue, red) and the shielding (gray) groups.

The examination of a set of previously reported crystal structures of substituted *N*-methylpyridinium arylsulfonate salts confirms that a wide range of relative orientations of the two ions is possible (**C1**^**+**^**A1**^**–**^, Figure 2b). This structural flexibility is expected for a bond with high ionic character (Figure 1a) and is consistent with the nondirectional distribution of the electrostatic potential of ions **C1**^**+**^ and **A1**^**–**^ (Figure 2a). Furthermore, the molecular structures of ion pairs **C2**^**+**^**A4**^**–**^ and **C4**^**+**^**A2**^**–**^, which each contain one insufficiently shielded ion, display a strongly bent arrangement of the anion and the cation, while in the case of **C3**^**+**^**A3**^**–**^, the anion approaches the cation from the second exposed site rather than from the side with the *N*-CH_3_ group (Figure 2b). However, as indicated by the analysis of the electrostatic potential maps, the restricted access to the charged areas between positions 2- and 6- in cation **C4**^**+**^ and anion **A3**^**–**^ results in the predicted geometry of the ionic bond (Figure 2c). The geometric consequence of the closest possible approach of the two ionic moieties, which maximizes the Coulomb interaction, is a close-to-linear arrangement of the backbones of the two ions in ion pair **1**. Because of the predictable and spatially well-defined molecular assembly, which contrasts the usual lack of directionality of ionic bonds that are not supported by hydrogen bonds^49−51^ (Figures 1a and 2b), we propose that the ionic bond in **1** (Figure 2c) can be considered as a realization of a directional ionic bond (Figure 1c). The ionic bond in **1** is distinct from other types of directional bonding because it does not rely on the covalent contribution for the directionality of bonding, although oriented electrostatic potential also plays a role in halogen and hydrogen bonds.^64−68^

The generality of directional ionic bonds is supported by the observation of the expected bonding geometry for ions with varied shielding substituents at the positions 2- and 6- (**2**–**6**, Figure 3a). To compare the directionality of different ionic bonds, we define angles α and β as shown in Figure 3a, with higher directionality of the ionic bond corresponding to the values of the angles that are closer to 180°. A comparison of the α and β angles for all ionic bonds discussed here is given in the two-dimensional plot in Figure 3b. While the ion pairs shown in Figure 2b, which do not contain sufficient shielding around the ions, give more varied and lower values for both of the angles (red circles), a narrower distribution closer to 180° is observed for directional ionic bonds (blue circles, the additional data points represent the examples discussed below). The deviation of the angles in the directional ionic bonds from 180° is a combined result of the lack of perfect shielding of the charged sites, crystal packing effects, and the presence of H_2_O molecules in some of the structures. Additionally, dispersion interactions are likely responsible for the tendency toward orientations that maximize the contact surface between the shielding substituents on the anion and those on the cation (**2** and **3**). With larger shielding groups, the central (hetero)arene planes of the ions are twisted toward a dihedral angle of 90° to give interlocked structures **4**–**6** in which the ionic bonds appear further shielded, but the α and β angles remain similar to those in **1**–**3**.

**Figure 3:**
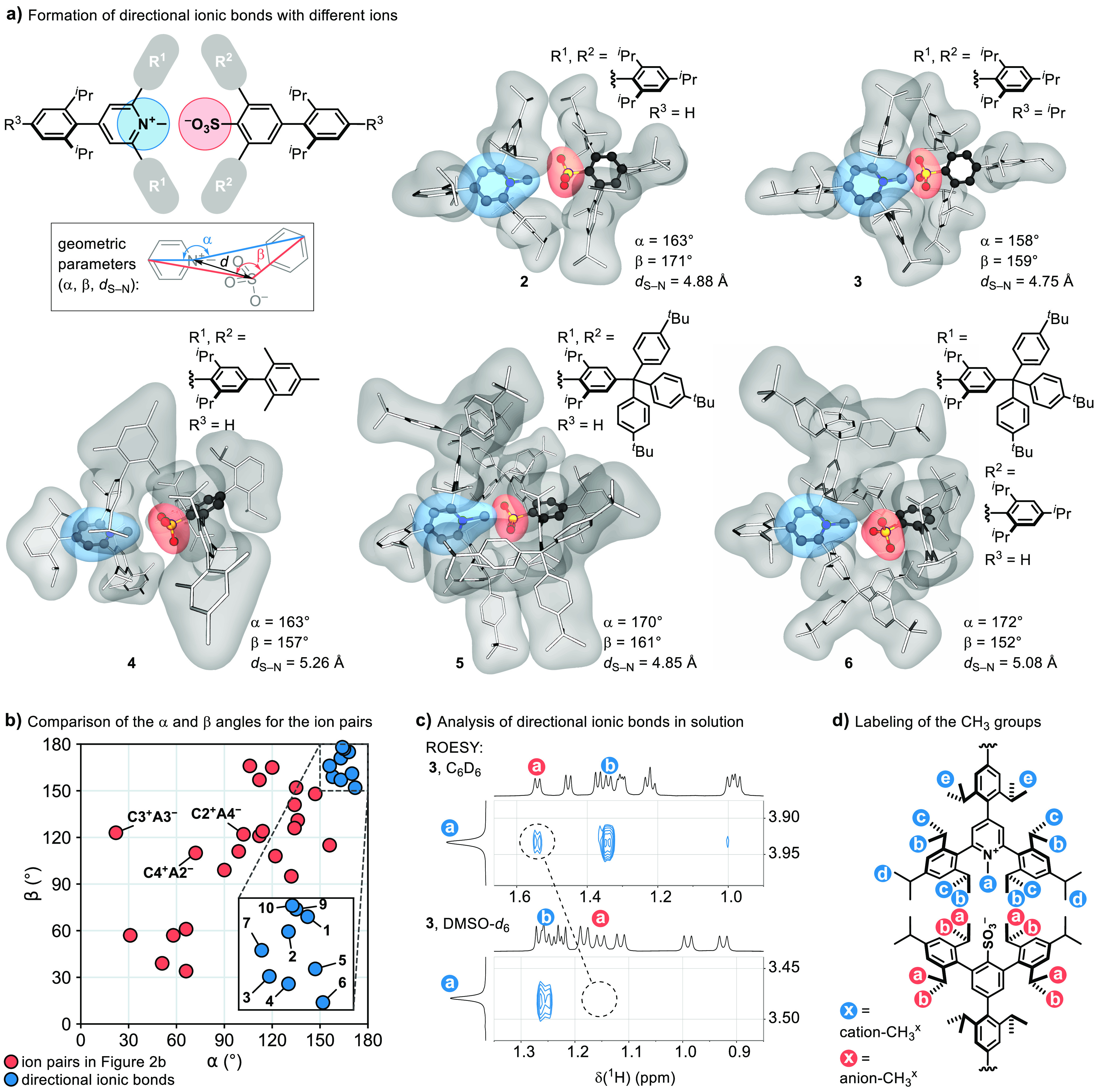
Generality and analysis of directional ionic bonds.

The study of the ionic bond in **3** in nonpolar solvents benzene-*d*_6_ and toluene-*d*_8_ by ^1^H–^1^H rotating frame Overhauser enhancement spectroscopy (ROESY) indicates the presence of the directional interaction in solution.^69^ The through-space ROE correlations in the ROESY spectrum indicate spatial proximity of the *N*-methyl group (labeled as cation-CH_3_^a^) of the cation and the methyl groups closest to the −SO_3_^–^ group (labeled as anion-CH_3_^a^) on the anion (Figure 3c, top; see Figure 3d for the labeling of the groups). Further strong anion–cation correlations are observed between anion-CH_3_^a^ and cation-CH_3_^b^ and cation-CH_3_^d^ protons, while weaker (cation-CH_3_^c^) or no correlations (cation-CH_3_^e^) are observed with more distant methyl groups (Supporting Information and Figure S1c, top). The calculated binding free energy for **3** at the CPCM(benzene)/M06-2X/6-311++G(3df,2p)//M06-2X/6-31+G(d,p) level of theory is 15 kcal/mol (see the Supporting Information). For comparison, the estimation of the binding electrostatic potential energy by approximating the ions in **3** with two point charges centered on the sulfur atom and the pyridine ring gives a value of 25 kcal/mol in benzene. The addition of an external ion pair ^*n*^Bu_4_N^+^BF_4_^–^ to the solution of **3** in C_6_D_6_ results in the loss of the cation–anion ROE correlations, thereby demonstrating the cleavage of the directional ionic bond by the exogenous ion pair (Figure S1). Furthermore, the cation–anion correlations are not observed in the polar solvent DMSO-*d*_6_, which indicates ion separation or potentially a less directional ion pairing (Figure 3c, bottom).

The SAPT analysis^70^ of the truncated structures indicates that the presence of the shielding groups contributes around 13% to the total interaction energy in **3** (Figure S2a, **3** vs **3**_trunc_**A**) with the dispersion and induction contributions being largely offset by the steric repulsion (**3**_trunc_**B** and **3**_trunc_**C**). The electrostatic contribution dominates the interaction in the directional ionic bond and it is comparable to the energy between two point charges centered on the N and S atoms (Figure S2c; for a comparison with a simpler ion pair and hydrogen- and halogen-bonded systems, see Figure S2d).^71^ However, larger shielding groups provide higher stabilization of the interaction (25% estimated in the case of **6**, Figure S2b).

Following the observation of the directionality in the solid state and in nonpolar solvents, we next show the potential of directional ionic bonds for the spatial organization of matter over distances of several nanometers. The modular synthetic approach to the ions in **1**–**6** (Figures S3 and S4) enables the connection of additional molecular units to the directional ionic bond at the *para*-positions of the shielding substituents that are present at the back sides of the cation and the anion (Figures S5 and S6). The approximate geometric collinearity of the covalent bonds at these sites enables the use of directional ionic bonds as structurally well-defined linear bonds (Figure 4a). For example, linear and bent supramolecular systems, such as **7** and **8** (Figure 4a), can be accessed by the use of two directional ionic bonds and 1,4- and 1,3-disubstituted benzene groups as spacers. The ROESY analysis of **7** and **8** reveals a similar interaction pattern to that of **3**, although the signals between anion-CH_3_^a^ and cation-CH_3_^c,e^ appear stronger, which suggests a more flexible orientation of the cation (Figure 4b; see Figure 3d for the labeling of the groups). Water molecules can bind to the oxygen atoms of arylsulfonate anions under air, and the presence of a water molecule is observed in the solid-state structure of **7**. However, the approximate linear geometry remains preserved (Figure 4c), which shows that the directionality of directional ionic bonds can be maintained in the presence of strong hydrogen bond donors. Furthermore, directional ionic bonds can be applied to the molecular architecture at the nanoscale level, as shown with the molecular structure of **9**, in which the binding of two palladium-porphyrin groups through two directional ionic bonds leads to a separation of the metal centers by 6.5 nm (Figure 4a,d). The precise spatial organization of functional molecular units, such as porphyrins, over large distances is relevant in synthetic and biological systems, such as molecular light-harvesting devices.^72−74^

The use of more complex multi-ionic units enables access to structurally well-defined materials with directional ionic bonds, as exemplified by the formation of a two-dimensional hexagonal structure in **10** (Figure 4e). The hexagons in **10** are composed of six directional ionic bonds, with the long and the short diagonals of the hexagon measuring 5.2 and 4.6 nm, respectively. In the crystal structure, the 2D layers form an ABCA′B′C′ stacking pattern (where ′ indicates a glide plane relationship) in which cavities are present between the ions of non-neighboring layers (Figure 4e, Figure S7). Transmission electron microscopy (TEM) shows the formation of thin plates upon drop casting (Figure 4e, Figure S8), while the thermal analysis of the constituent ions demonstrates partial decomposition upon the heating of **10** at 250 °C for 1 h (see the Supporting Information). Porous materials, such as metal–organic frameworks,^75−77^ covalent organic frameworks,^78^ and (charge-assisted)^54−56^ hydrogen-bonded organic frameworks,^21,22,59^ have a range of applications in diverse areas of chemistry and can have geometries related to that in **10**.^52,79,80^ However, directional ionic bonds offer a complementary strategy for the construction of organic materials by utilizing otherwise nondirectional Coulomb interactions between two ions as a geometrically well-defined linear bonding element.

Directional ionic bonds, which are demonstrated with the structures of **1**–**10**, open new avenues for the structuring of organic matter by imparting directionality to ionic bonding, which is a fundamental^81−83^ and omnipresent mode of chemical bonding. Several properties of directional ionic bonds, including the binding strength and the modulation with solvent polarity and competing ions, are comparable with the structural and dynamical properties of hydrogen bonds. Therefore, the use of directional ionic bonds might be envisioned in diverse areas of supramolecular and materials chemistry. Additionally, as demonstrated with the structures of **7**–**10**, directional ionic bonds connect molecular fragments in a linear geometry at a distance of >2 nm, which makes such interactions especially suited for molecular architecture at the nanoscale.

**Figure 4:**
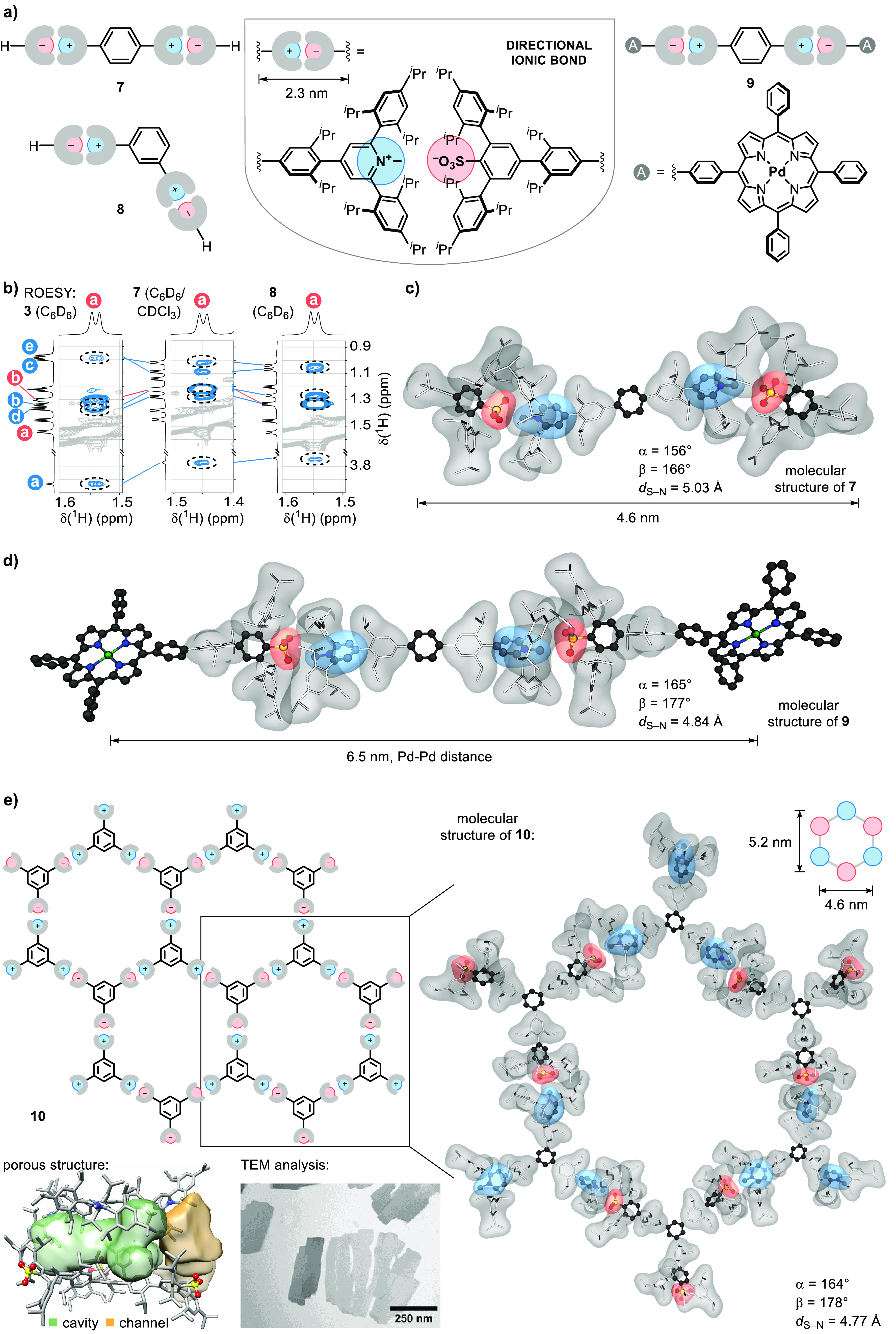
Molecular architecture at the nanoscale. (a) Construction of supramolecular assemblies. (b–d), Characterization of **7**–**9**. (e) Directional ionic materials.

## Supporting Information Available

Experimental details, characterization spectra, single crystal data, and computational details (PDF)

## References

[ref1] Pauling L. The nature of the chemical bond. Application of results obtained from the quantum mechanics and from a theory of paramagnetic susceptibility to the structure of molecules. J. Am. Chem. Soc. 1931, 53, 1367 10.1021/ja01355a027.

[ref2] Zhao L.; Schwarz W. H. E.; Frenking G. The Lewis electron-pair bonding model: the physical background, one century later. Nat. Rev. Chem. 2019, 3, 35 10.1038/s41570-018-0052-4.

[ref3] The Chemical Bond; Frenking G., Shaik S., Eds.; Wiley-VCH: Weinheim, Germany, 2014.

[ref4] Trauner D. The Chemist and the Architect. Angew. Chem., Int. Ed. 2018, 57, 4177 10.1002/anie.201708325. 29281154

[ref5] Nicolaou K. C.; Heretsch P.; Nakamura T.; Rudo A.; Murata M.; Konoki K. Synthesis and biological evaluation of QRSTUVWXYZA′ domains of maitotoxin. J. Am. Chem. Soc. 2014, 136, 16444 10.1021/ja509829e. 25374117 PMC4244842

[ref6] Grigalunas M.; Brakmann S.; Waldmann H. Chemical Evolution of Natural Product Structure. J. Am. Chem. Soc. 2022, 144, 3314 10.1021/jacs.1c11270. 35188375 PMC8895405

[ref7] Reymond J.-L.; Awale M. Exploring chemical space for drug discovery using the chemical universe database. ACS Chem. Neurosci. 2012, 3, 649 10.1021/cn3000422. 23019491 PMC3447393

[ref8] Vinothkumar K. R.; Henderson R. Structures of membrane proteins. Q. Rev. Biophys. 2010, 43, 65 10.1017/S0033583510000041. 20667175 PMC3604715

[ref9] Yao H.; Song Y.; Chen Y.; Wu N.; Xu J.; Sun C.; Zhang J.; Weng T.; Zhang Z.; Wu Z.; Cheng L.; Shi D.; Lu X.; Lei J.; Crispin M.; Shi Y.; Li L.; Li S. Molecular Architecture of the SARS-CoV-2 Virus. Cell 2020, 183, 730 10.1016/j.cell.2020.09.018. 32979942 PMC7474903

[ref10] Wicky B. I. M.; Milles L. F.; Courbet A.; Ragotte R. J.; Dauparas J.; Kinfu E.; Tipps S.; Kibler R. D.; Baek M.; DiMaio F.; Li X.; Carter L.; Kang A.; Nguyen H.; Bera A. K.; Baker D. Hallucinating symmetric protein assemblies. Science 2022, 378, 56 10.1126/science.add1964. 36108048 PMC9724707

[ref11] Bols P. S.; Anderson H. L. Template-Directed Synthesis of Molecular Nanorings and Cages. Acc. Chem. Res. 2018, 51, 2083 10.1021/acs.accounts.8b00313. 30156831

[ref12] Fujita D.; Ueda Y.; Sato S.; Mizuno N.; Kumasaka T.; Fujita M. Self-assembly of tetravalent Goldberg polyhedra from 144 small components. Nature 2016, 540, 563 10.1038/nature20771. 30905932

[ref13] Smulders M. M. J.; Riddell I. A.; Browne C.; Nitschke J. R. Building on architectural principles for three-dimensional metallosupramolecular construction. Chem. Soc. Rev. 2013, 42, 1728 10.1039/C2CS35254K. 23032789

[ref14] Cook T. R.; Stang P. J. Recent Developments in the Preparation and Chemistry of Metallacycles and Metallacages via Coordination. Chem. Rev. 2015, 115, 7001 10.1021/cr5005666. 25813093

[ref15] Ashbridge Z.; Fielden S. D. P.; Leigh D. A.; Pirvu L.; Schaufelberger F.; Zhang L. Knotting matters: orderly molecular entanglements. Chem. Soc. Rev. 2022, 51, 7779 10.1039/D2CS00323F. 35979715 PMC9486172

[ref16] Zhang G.; Mastalerz M. Organic cage compounds – from shape-persistency to function. Chem. Soc. Rev. 2014, 43, 1934 10.1039/C3CS60358J. 24336604

[ref17] Desiraju G. R.; Steiner T. The Weak Hydrogen Bond: In Structural Chemistry and Biology; Oxford University Press: New York, 1999.

[ref18] Ajami D.; Rebek J. Jr. More chemistry in small spaces. Acc. Chem. Res. 2013, 46, 990 10.1021/ar300038r. 22574934

[ref19] Liu Y.; Hu C.; Comotti A.; Ward M. D. Supramolecular Archimedean Cages Assembled with 72 Hydrogen Bonds. Science 2011, 333, 436 10.1126/science.1204369. 21778396

[ref20] Troselj P.; Bolgar P.; Ballester P.; Hunter C. A. High-Fidelity Sequence-Selective Duplex Formation by Recognition-Encoded Melamine Oligomers. J. Am. Chem. Soc. 2021, 143, 8669 10.1021/jacs.1c02275. 34081864 PMC8213060

[ref21] Lin R.-B.; He Y.; Li P.; Wang H.; Zhou W.; Chen B. Multifunctional porous hydrogen-bonded organic framework materials. Chem. Soc. Rev. 2019, 48, 1362 10.1039/C8CS00155C. 30676603 PMC11061856

[ref22] Li P.; Ryder M. R.; Stoddart J. F. Hydrogen-Bonded Organic Frameworks: A Rising Class of Porous Molecular Materials. Acc. Mater. Res. 2020, 1, 77 10.1021/accountsmr.0c00019.

[ref23] Liang L.; Zhao W.; Yang X.-J.; Wu B. Anion-Coordination-Driven Assembly. Acc. Chem. Res. 2022, 55, 3218 10.1021/acs.accounts.2c00435. 36331808

[ref24] Evans N. H.; Beer P. D. Advances in anion supramolecular chemistry: From recognition to chemical applications. Angew. Chem., Int. Ed. 2014, 53, 11716 10.1002/anie.201309937. 25204549

[ref25] Aida T.; Meijer E. W. Supramolecular Polymers – we’ve Come Full Circle. Isr. J. Chem. 2020, 60, 33 10.1002/ijch.201900165.

[ref26] Gilday L. C.; Robinson S. W.; Barendt T. A.; Langton M. J.; Mullaney B. R.; Beer P. D. Halogen Bonding in Supramolecular Chemistry. Chem. Rev. 2015, 115, 7118 10.1021/cr500674c. 26165273

[ref27] Auffinger P.; Hays F. A.; Westhof E.; Ho P. S. Halogen bonds in biological molecules. Proc. Natl. Acad. Sci. U. S. A. 2004, 101, 16789 10.1073/pnas.0407607101. 15557000 PMC529416

[ref28] Metrangolo P.; Neukirch H.; Pilati T.; Resnati G. Halogen bonding based recognition processes: A world parallel to hydrogen bonding. Acc. Chem. Res. 2005, 38, 386 10.1021/ar0400995. 15895976

[ref29] Wilcken R.; Zimmermann M. O.; Lange A.; Joerger A. C.; Boeckler F. M. Principles and applications of halogen bonding in medicinal chemistry and chemical biology. J. Med. Chem. 2013, 56, 1363 10.1021/jm3012068. 23145854

[ref30] Vogel L.; Wonner P.; Huber S. M. Chalcogen Bonding: An Overview. Angew. Chem., Int. Ed. 2019, 58, 1880 10.1002/anie.201809432. 30225899

[ref31] Stewart R. J.; Wang C. S.; Shao H. Complex coacervates as a foundation for synthetic underwater adhesives. Adv. Colloid Interface Sci. 2011, 167, 85 10.1016/j.cis.2010.10.009. 21081223 PMC3130813

[ref32] Decher G. Fuzzy nanoassemblies: Toward layered polymeric multicomposites. Science 1997, 277, 1232 10.1126/science.277.5330.1232.

[ref33] Faul C. F. J.; Antonietti M. Ionic self-assembly: Facile synthesis of supramolecular materials. Adv. Mater. 2003, 15, 673 10.1002/adma.200300379.

[ref34] Guldi D. M.; Prato M. Electrostatic interactions by design. Versatile methodology towards multifunctional assemblies/nanostructured electrodes. Chem. Commun. 2004, 2517 10.1039/b410541a. 15543261

[ref35] Lehn J.-M. Supramolecular Chemistry—Scope and Perspectives Molecules, Supermolecules, and Molecular Devices (Nobel Lecture). Angew. Chem., Int. Ed. Engl. 1988, 27, 89 10.1002/anie.198800891.

[ref36] Lin X.; Grinstaff M. W. Ionic supramolecular assemblies. Isr. J. Chem. 2013, 53, 498 10.1002/ijch.201300034.

[ref37] Cherstvy A. G. Electrostatic interactions in biological DNA-related systems. Phys. Chem. Chem. Phys. 2011, 13, 9942 10.1039/c0cp02796k. 21431196

[ref38] Perutz M. F. Electrostatic Effects in Proteins. Science 1978, 201, 1187 10.1126/science.694508. 694508

[ref39] Marcus Y.; Hefter G. Ion pairing. Chem. Rev. 2006, 106, 4585 10.1021/cr040087x. 17091929

[ref40] Biedermann F.; Schneider H.-J. Experimental Binding Energies in Supramolecular Complexes. Chem. Rev. 2016, 116, 5216 10.1021/acs.chemrev.5b00583. 27136957

[ref41] Li Y.; Wang Y.; Huang G.; Gao J. Cooperativity Principles in Self-Assembled Nanomedicine. Chem. Rev. 2018, 118, 5359 10.1021/acs.chemrev.8b00195. 29693377 PMC6524957

[ref42] Spruijt E.; van den Berg S. A.; Cohen Stuart M. A.; van der Gucht J. Direct measurement of the strength of single ionic bonds between hydrated charges. ACS Nano 2012, 6, 5297 10.1021/nn301097y. 22559075

[ref43] Goossens K.; Lava K.; Bielawski C. W.; Binnemans K. Ionic Liquid Crystals: Versatile Materials. Chem. Rev. 2016, 116, 4643 10.1021/cr400334b. 27088310

[ref44] Wathier M.; Grinstaff M. W. Synthesis and properties of supramolecular ionic networks. J. Am. Chem. Soc. 2008, 130, 9648 10.1021/ja803248q. 18593156

[ref45] Cuthbert T. J.; Jadischke J. J.; de Bruyn J. R.; Ragogna P. J.; Gillies E. R. Self-Healing Polyphosphonium Ionic Networks. Macromolecules 2017, 50, 5253 10.1021/acs.macromol.7b00955.

[ref46] Xu L.; Jiang L.; Drechsler M.; Sun Y.; Liu Z.; Huang J.; Tang B. Z.; Li Z.; Cohen Stuart M. A.; Yan Y. Self-assembly of ultralong polyion nanoladders facilitated by ionic recognition and molecular stiffness. J. Am. Chem. Soc. 2014, 136, 1942 10.1021/ja410443n. 24417504

[ref47] Fiammengo R.; Timmerman P.; de Jong F.; Reinhoudt D. N. Highly stable cage-like complexes by self-assembly of tetracationic Zn(II) porphyrinates and tetrasulfonatocalix[4]arenes in polar solvents. Chem. Commun. 2000, 2313 10.1039/b006960o.

[ref48] Oshovsky G. V.; Reinhoudt D. N.; Verboom W. Self-assembled hemicapsules with inherent functionalities: Modeling of a supramolecular electrostatic self-assembly. J. Org. Chem. 2006, 71, 7441 10.1021/jo061344w. 16958540

[ref49] Rehm T. H.; Schmuck C. Ion-pair induced self-assembly in aqueous solvents. Chem. Soc. Rev. 2010, 39, 3597 10.1039/b926223g. 20552123

[ref50] Tanaka Y.; Katagiri H.; Furusho Y.; Yashima E. A Modular Strategy to Artificial Double Helices. Angew. Chem., Int. Ed. 2005, 44, 3867 10.1002/anie.200501028. 15900525

[ref51] Maeda T.; Furusho Y.; Sakurai S.-I.; Kumaki J.; Okoshi K.; Yashima E. Double-stranded helical polymers consisting of complementary homopolymers. J. Am. Chem. Soc. 2008, 130, 7938 10.1021/ja711447s. 18510315

[ref52] Liu Y.; Xiao W.; Yi J. J.; Hu C.; Park S.-J.; Ward M. D. Regulating the architectures of hydrogen-bonded frameworks through topological enforcement. J. Am. Chem. Soc. 2015, 137, 3386 10.1021/jacs.5b00534. 25730635

[ref53] Ganie A. A.; Ahangar A. A.; Dar A. A. Sulfonate···Pyridinium Supramolecular Synthon: A Robust Interaction Utilized to Design Molecular Assemblies. Cryst. Growth Des. 2019, 19, 4650 10.1021/acs.cgd.9b00555.

[ref54] White N. G. Recent advances in self-assembled amidinium and guanidinium frameworks. Dalton Trans. 2019, 48, 7062 10.1039/C8DT05030A. 30667427

[ref55] Yu S.; Xing G.-L.; Chen L.-H.; Ben T.; Su B.-L. Crystalline Porous Organic Salts: From Micropore to Hierarchical Pores. Adv. Mater. 2020, 32, 2003270 10.1002/adma.202003270. 32930443

[ref56] Mottillo C.; Friščić T. Supramolecular imidazolium frameworks: direct analogues of metal azolate frameworks with charge-inverted node-and-linker structure. Chem. Commun. 2015, 51, 8924 10.1039/C5CC01645B. 25779369

[ref57] Sessler J. L.; Gross D. E.; Cho W.-S.; Lynch V. M.; Schmidtchen F. P.; Bates G. W.; Light M. E.; Gale P. A. Calix[4]pyrrole as a chloride anion receptor: Solvent and countercation effects. J. Am. Chem. Soc. 2006, 128, 12281 10.1021/ja064012h. 16967979 PMC2572717

[ref58] Kim S. K.; Sessler J. L. Calix[4]pyrrole-based ion pair receptors. Acc. Chem. Res. 2014, 47, 2525 10.1021/ar500157a. 24977935

[ref59] Skala L. P.; Stern C. L.; Bancroft L.; Moisanu C. M.; Pelkowski C.; Aguilar-Enriquez X.; Swartz J. L.; Wasielewski M. R.; Dichtel W. R. A modular platform for the precise assembly of molecular frameworks composed of ion pairs. Chem. 2023, 10.1016/j.chempr.2023.01.011.

[ref60] Haketa Y.; Sasaki S.; Ohta N.; Masunaga H.; Ogawa H.; Mizuno N.; Araoka F.; Takezoe H.; Maeda H. Oriented Salts: Dimension-Controlled Charge-by-Charge Assemblies from Planar Receptor–Anion Complexes. Angew. Chem., Int. Ed. 2010, 49, 10079 10.1002/anie.201006356. 21117126

[ref61] Dong B.; Sakurai T.; Honsho Y.; Seki S.; Maeda H. Cation modules as building blocks forming supramolecular assemblies with planar receptor-anion complexes. J. Am. Chem. Soc. 2013, 135, 1284 10.1021/ja312214a. 23301540

[ref62] Haketa Y.; Maeda H. Dimension-controlled ion-pairing assemblies based on π-electronic charged species. Chem. Commun. 2017, 53, 2894 10.1039/C6CC10255G. 28225096

[ref63] Shimizu G. K. H.; Vaidhyanathan R.; Taylor J. M. Phosphonate and sulfonate metal organic frameworks. Chem. Soc. Rev. 2009, 38, 1430 10.1039/b802423p. 19384446

[ref64] van der Lubbe S. C. C.; Fonseca Guerra C. The Nature of Hydrogen Bonds: A Delineation of the Role of Different Energy Components on Hydrogen Bond Strengths and Lengths. Chem. Asian J. 2019, 14, 2760 10.1002/asia.201900717. 31241855 PMC6771679

[ref65] Wang C.; Guan L.; Danovich D.; Shaik S.; Mo Y. The origins of the directionality of noncovalent intermolecular interactions. J. Comput. Chem. 2016, 37, 34 10.1002/jcc.23946. 26010349

[ref66] Politzer P.; Murray J. S.; Clark T. Halogen bonding: an electrostatically-driven highly directional noncovalent interaction. Phys. Chem. Chem. Phys. 2010, 12, 7748 10.1039/c004189k. 20571692

[ref67] Kellett C. W.; Kennepohl P.; Berlinguette C. P. π covalency in the halogen bond. Nat. Commun. 2020, 11, 3310 10.1038/s41467-020-17122-7. 32620765 PMC7335087

[ref68] Pascoe D. J.; Ling K. B.; Cockroft S. L. The Origin of Chalcogen-Bonding Interactions. J. Am. Chem. Soc. 2017, 139, 15160 10.1021/jacs.7b08511. 28985065

[ref69] Moreno A.; Pregosin P. S.; Veiros L. F.; Albinati A.; Rizzato S. Ion Pairing and Salt Structure in Organic Salts through Diffusion, Overhauser, DFT and X-ray Methods. Chem. Eur. J. 2009, 15, 6848 10.1002/chem.200900021. 19504520

[ref70] Patkowski K. Recent developments in symmetry-adapted perturbation theory. WIREs Comput. Mol. Sci. 2020, 10, e1452 10.1002/wcms.1452.

[ref71] Su P.; Li H. Energy decomposition analysis of covalent bonds and intermolecular interactions. J. Chem. Phys. 2009, 131, 014102 10.1063/1.3159673. 19586091

[ref72] Arsenault E. A.; Yoneda Y.; Iwai M.; Niyogi K. K.; Fleming G. R. Vibronic mixing enables ultrafast energy flow in light-harvesting complex II. Nat. Commun. 2020, 11, 1460 10.1038/s41467-020-14970-1. 32193383 PMC7081214

[ref73] Uetomo A.; Kozaki M.; Suzuki S.; Yamanaka K.; Ito O.; Okada K. Efficient light-harvesting antenna with a multi-porphyrin cascade. J. Am. Chem. Soc. 2011, 133, 13276 10.1021/ja2050343. 21790118

[ref74] Drain C. M.; Varotto A.; Radivojevic I. Self-organized porphyrinic materials. Chem. Rev. 2009, 109, 1630 10.1021/cr8002483. 19253946 PMC2681784

[ref75] Furukawa H.; Cordova K. E.; O’Keeffe M.; Yaghi O. M. The chemistry and applications of metal-organic frameworks. Science 2013, 341, 1230444 10.1126/science.1230444. 23990564

[ref76] Sumida K.; Rogow D. L.; Mason J. A.; McDonald T. M.; Bloch E. D.; Herm Z. R.; Bae T.-H.; Long J. R. Carbon dioxide capture in metal-organic frameworks. Chem. Rev. 2012, 112, 724 10.1021/cr2003272. 22204561

[ref77] Xie L. S.; Skorupskii G.; Dincǎ M. Electrically Conductive Metal-Organic Frameworks. Chem. Rev. 2020, 120, 8536 10.1021/acs.chemrev.9b00766. 32275412 PMC7453401

[ref78] Diercks C. S.; Yaghi O. M. The atom, the molecule, and the covalent organic framework. Science 2017, 355, eaal1585 10.1126/science.aal1585. 28254887

[ref79] Calik M.; Auras F.; Salonen L. M.; Bader K.; Grill I.; Handloser M.; Medina D. D.; Dogru M.; Löbermann F.; Trauner D.; Hartschuh A.; Bein T. Extraction of photogenerated electrons and holes from a covalent organic framework integrated heterojunction. J. Am. Chem. Soc. 2014, 136, 17802 10.1021/ja509551m. 25412210 PMC4706362

[ref80] Huang N.; Ding X.; Kim J.; Ihee H.; Jiang D. A Photoresponsive Smart Covalent Organic Framework. Angew. Chem., Int. Ed. 2015, 54, 8704 10.1002/anie.201503902. PMC4531826 26095503

[ref81] Kossel W. Über Molekülbildung als Frage des Atombaus. Ann. Phys. 1916, 354, 229 10.1002/andp.19163540302.

[ref82] Lewis G. N. The atom and the molecule. J. Am. Chem. Soc. 1916, 38, 762 10.1021/ja02261a002.

[ref83] Constable E. C.; Housecroft C. E. Chemical Bonding: The Journey from Miniature Hooks to Density Functional Theory. Molecules 2020, 25, 2623 10.3390/molecules25112623. 32516906 PMC7321411

