# Supplementary material for: Directional Ionic Bonds
Source: J Am Chem Soc. 2023 Apr 7;145(15):8291–8. doi: 10.1021/jacs.3c01030 (PMC10119990; doi:10.1021/jacs.3c01030)
Supplement: Supplementary file 1 — ja3c01030_si_001.pdf [file ja3c01030_si_001.pdf]

Supporting Information

Directional Ionic Bonds

Illia Hutskalov, Anthony Linden, and Ilija Čorić*

Department of Chemistry, University of Zurich, Winterthurerstrasse 190, CH-8057 Zurich, Switzerland.

Table of Contents

1 Figures S1-S8 ... 3
2 Materials and Methods ... 10
2.1 General information ... 10
2.2 Synthesis of cations **C2**$^+$ and **C3**$^+$ ... 13
2.3 Synthesis of anions **A2**$^-$ and **A3**$^-$ ... 16
2.4 Synthesis of cations present in **1-6** ... 21
2.5 Synthesis of anions present in **1-6** ... 32
2.6 Synthesis of cations present in **7-10** ... 45
2.7 Synthesis of anions present in **9-10** ... 51
2.8 Synthesis and characterization of the ion pairs ... 57
2.9 Analysis of the directionality of the ionic bonds in solution ... 67
2.9.1 Analysis of **C3**$^+$**A3**$^-$ by ROESY ... 67
2.9.2 Analysis of **3** by ROESY ... 70
2.9.3 Analysis of **7** by ROESY ... 78
2.9.4 Analysis of **8** by ROESY ... 82
2.9.5 Analysis of **3** in the presence of $^{n}Bu_4N^+BF_4^-$ ... 85
2.10 ^1H-^1H DOSY NMR experiments ... 88
2.11 Thermal stability analysis ... 96
2.11.1 Thermogravimetric analysis (TGA) and melting points ... 96
2.11.2 NMR studies of thermal stability of **10** ... 97
2.12 Computational studies ... 98
3 Supplementary Data ... 104
3.1 NMR spectra ... 104

3.2 Cartesian coordinates of DFT and PM6 optimized structures 199
3.2.1 Structures optimized at the M06-2X/6-31+G(d,p) level of theory 199
3.2.2 Structures optimized at the M06-2X/6-31G(d) level of theory 215
3.2.3 Structure optimized with the PM6 semi-empirical method 219
3.3 Crystallographic data 222
4 Supplementary References 277

1 Figures S1-S8

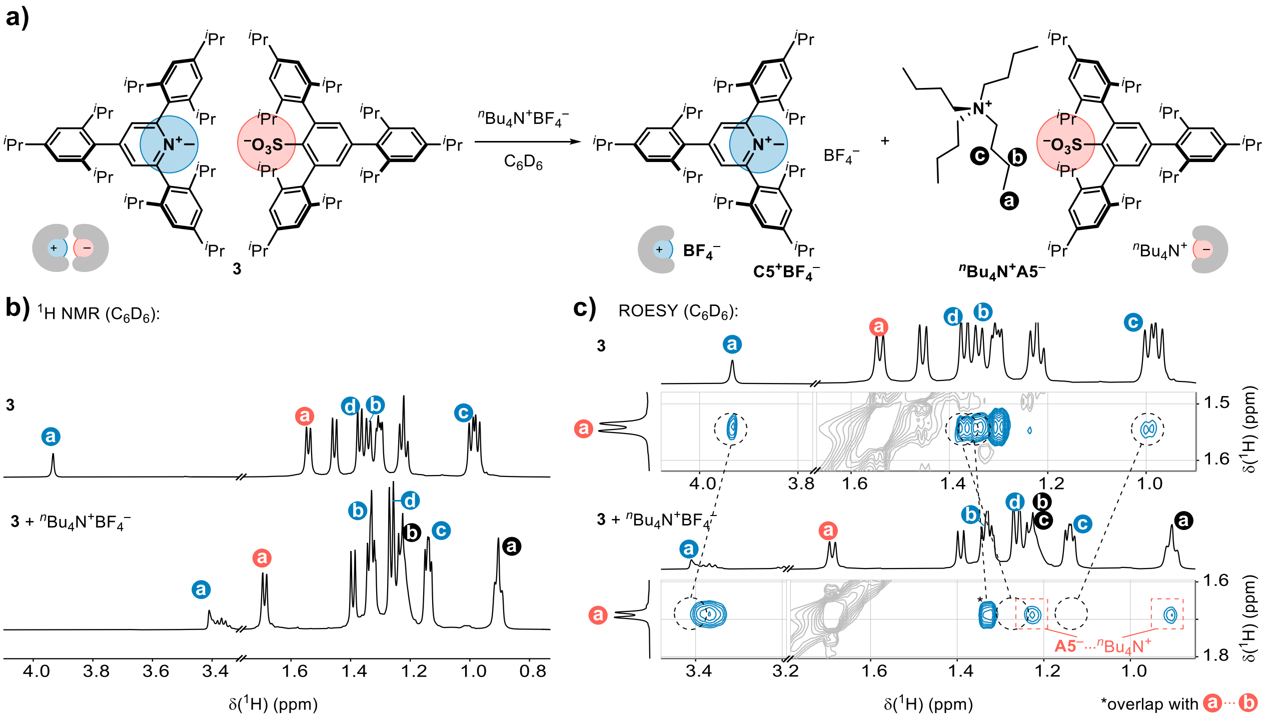

Figure S1. (a) The cleavage of the directional ionic bond. (b)-(c) Comparisons of the selected regions of the ^{1}H NMR and ROESY spectra of **3** before and after the addition of $^nBu_4N^+BF_4^-$.

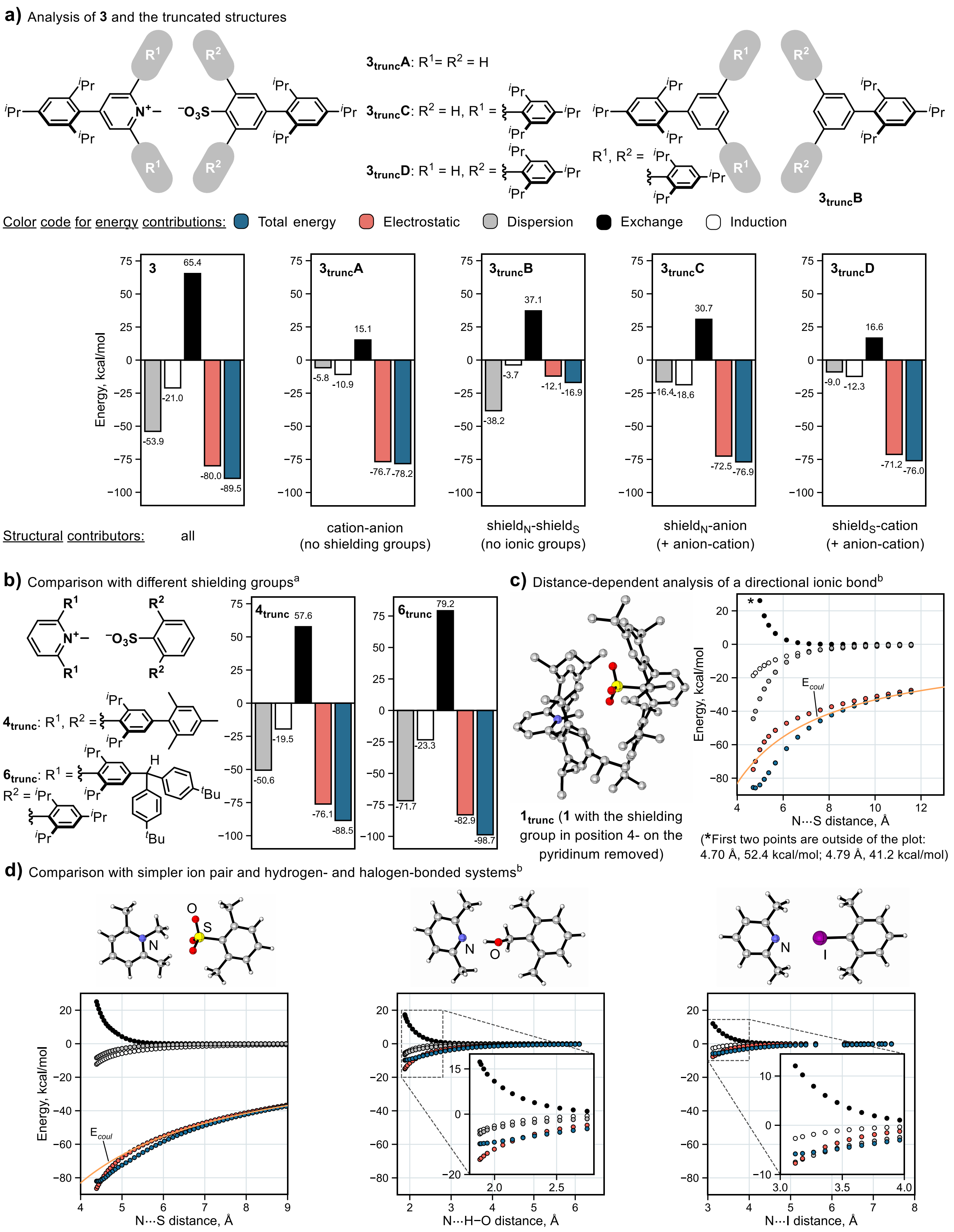

Figure S2. Symmetry-Adapted Perturbation Theory (SAPT) analysis of energy components of the interaction energy for directional ionic bonds. For the details of the calculations, see Section 2.12. [a] For comparison with the corresponding structures without the shielding groups, see Section 2.12. [b] E_{coul} = electrostatic energy between two point charges at the same distance (given for comparison).

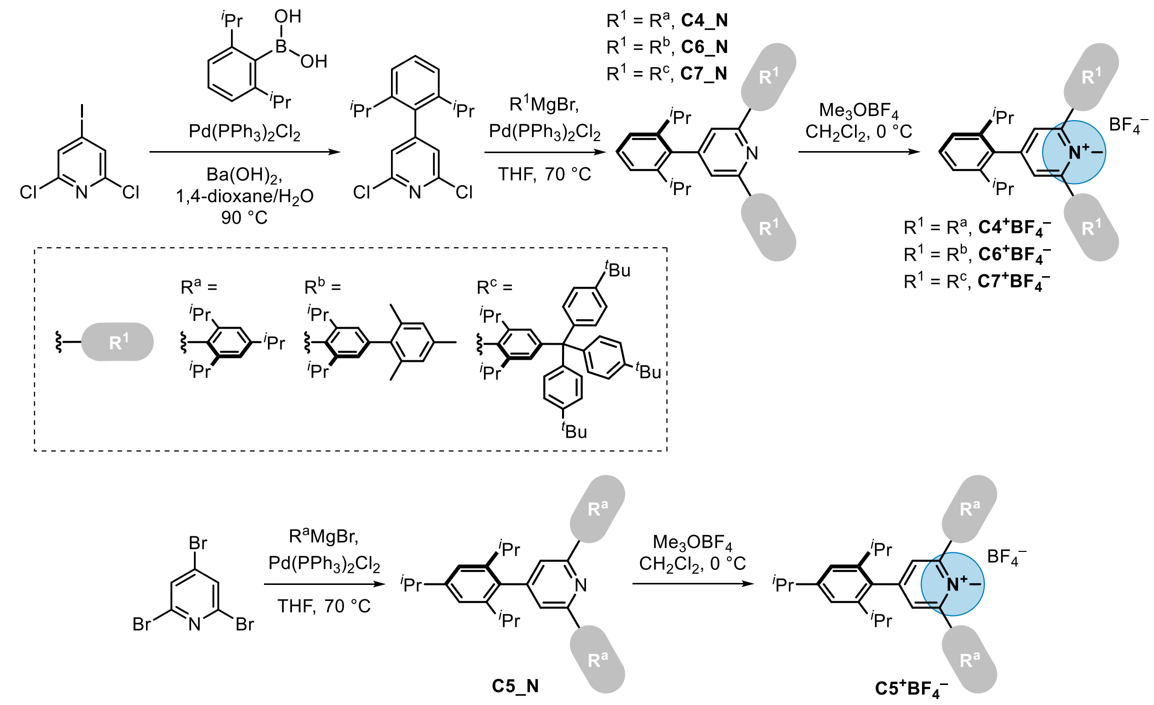

Figure S3. Synthesis of cations present in **1-6**.

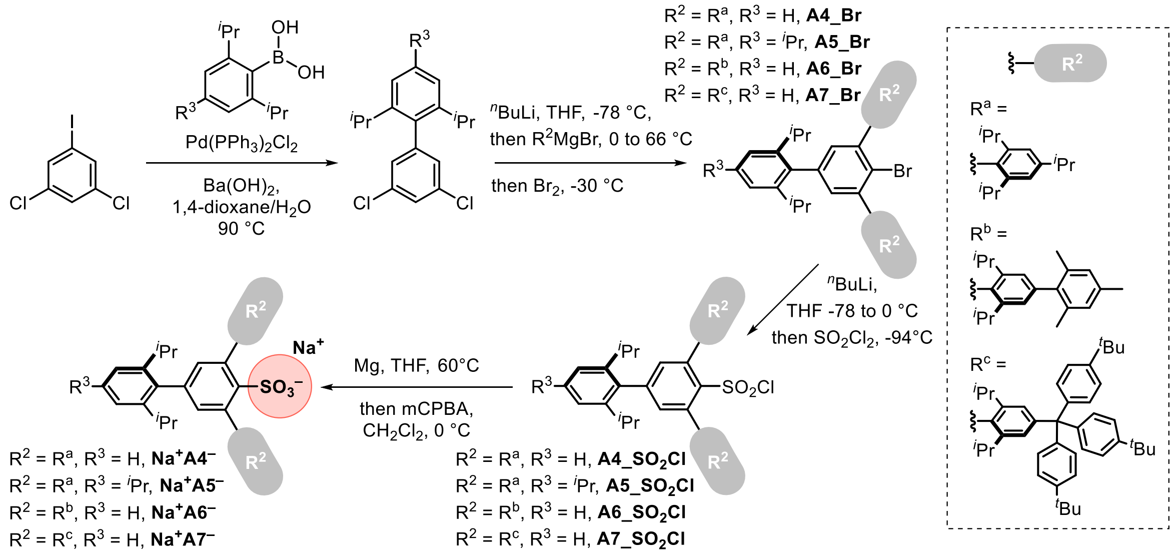

Figure S4. Synthesis of anions present in **1-6**.

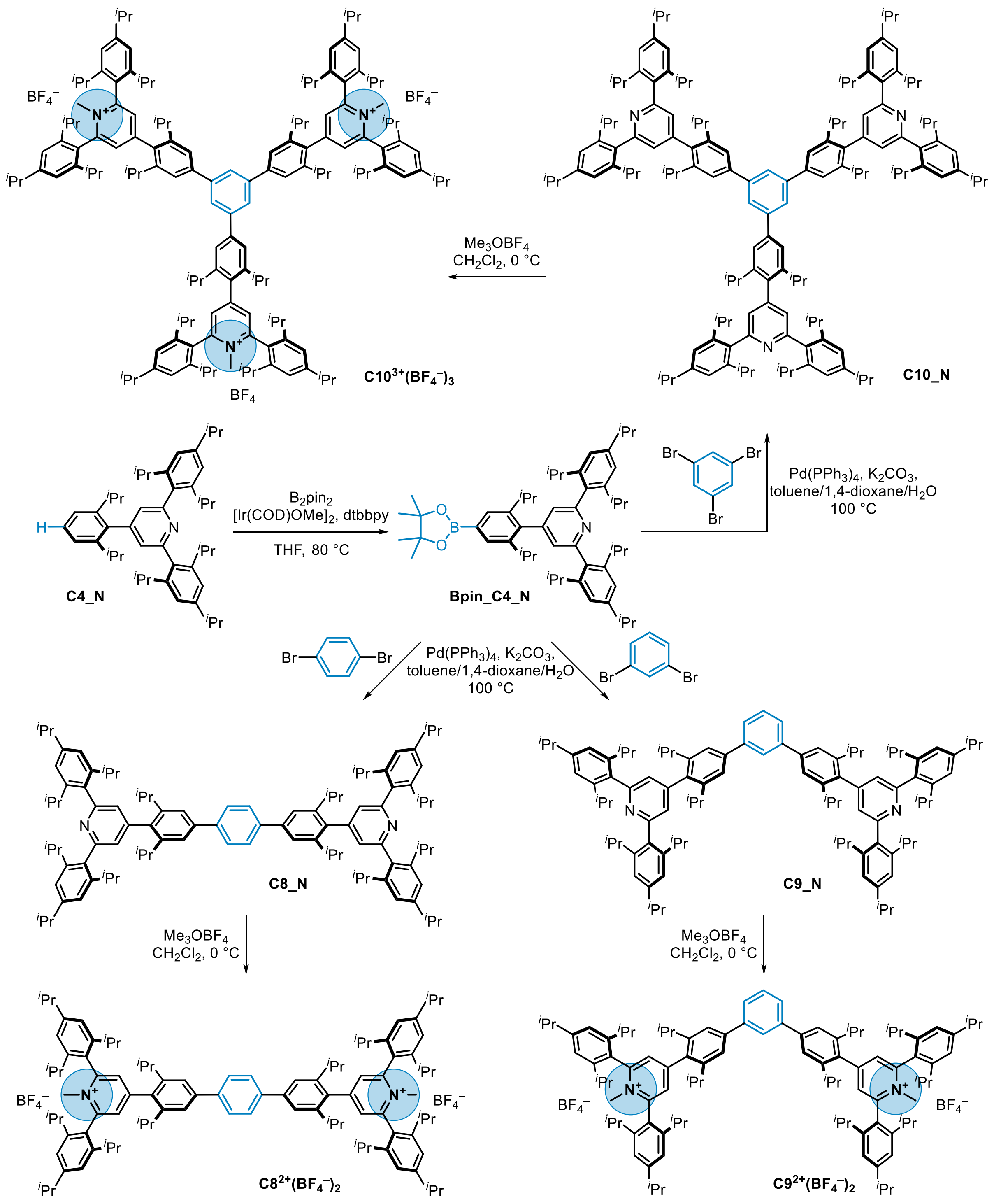

Figure S5. Synthesis of cations present in **7-10**.

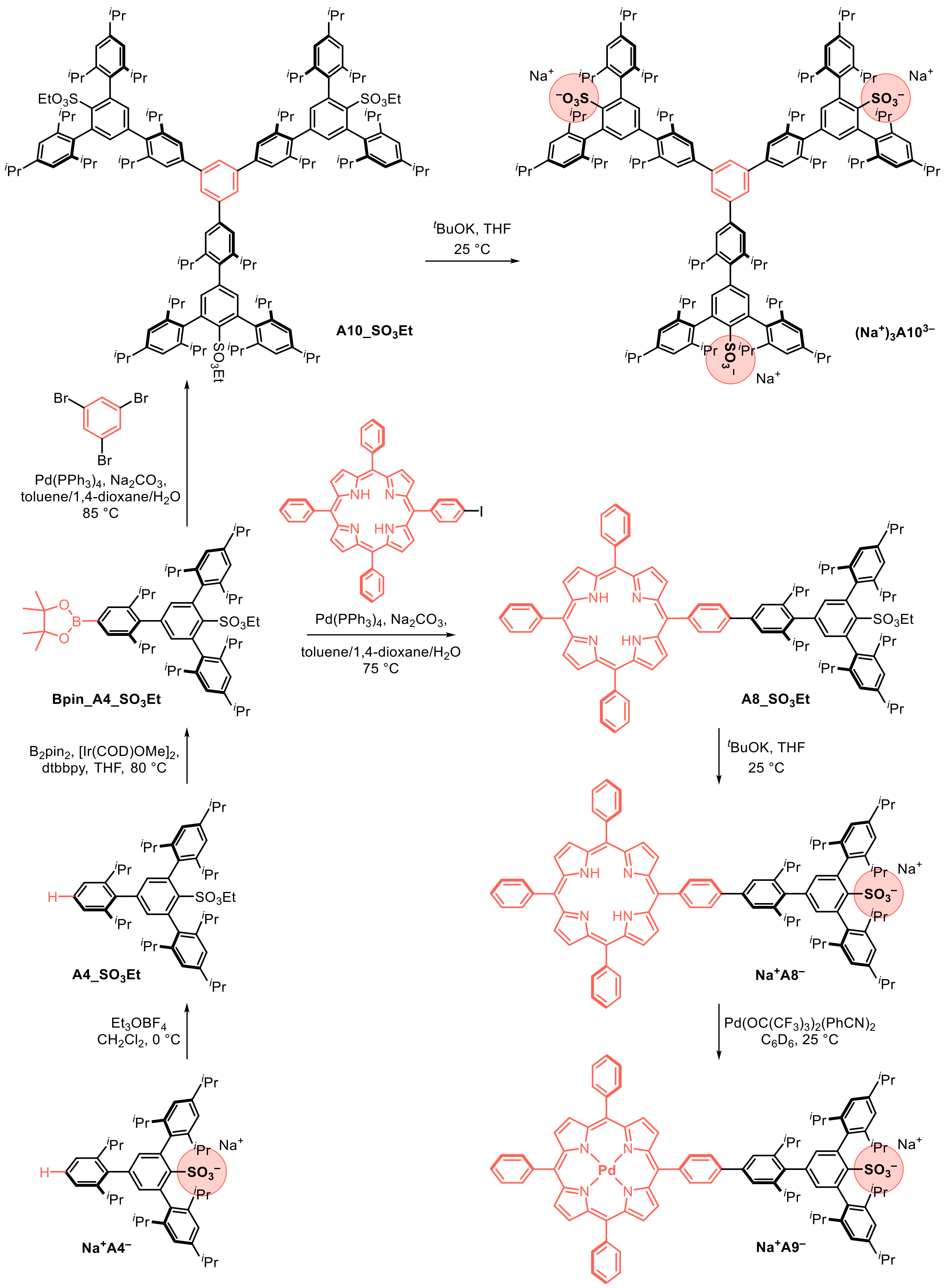

Figure S6. Synthesis of anions present in **9-10**.

a) ABCA'B'C' stacking pattern

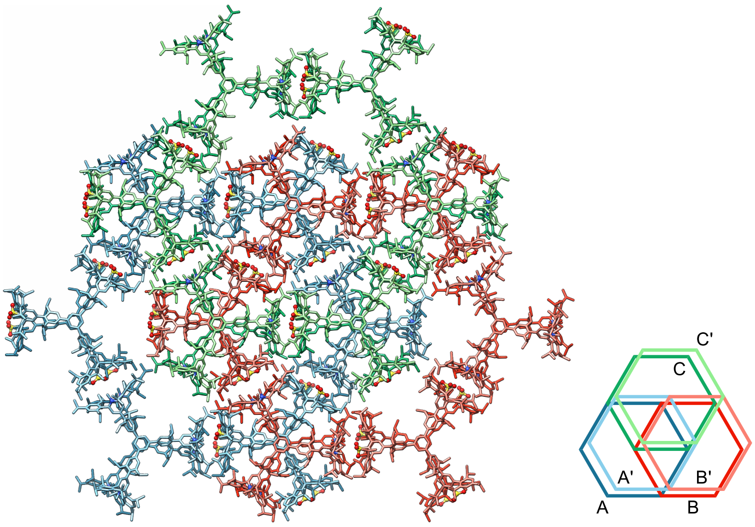

b) ABC layers only

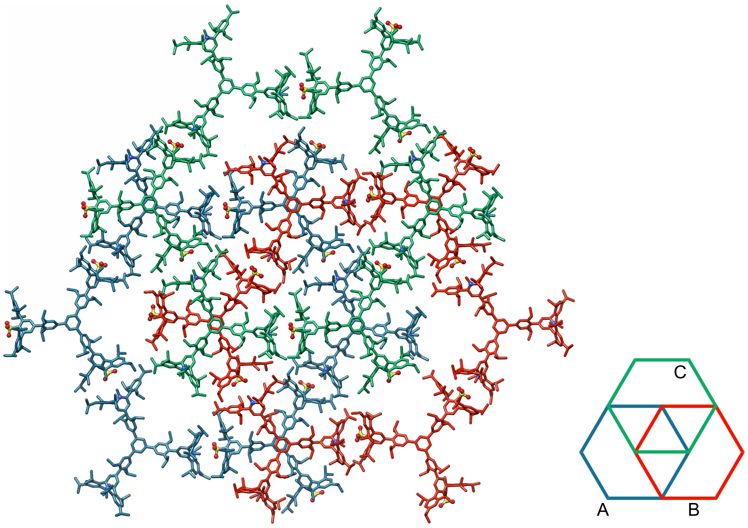

c) Pore and stacking analysis (shown starting from two neighbouring vertices of the hexagon in the A layer)

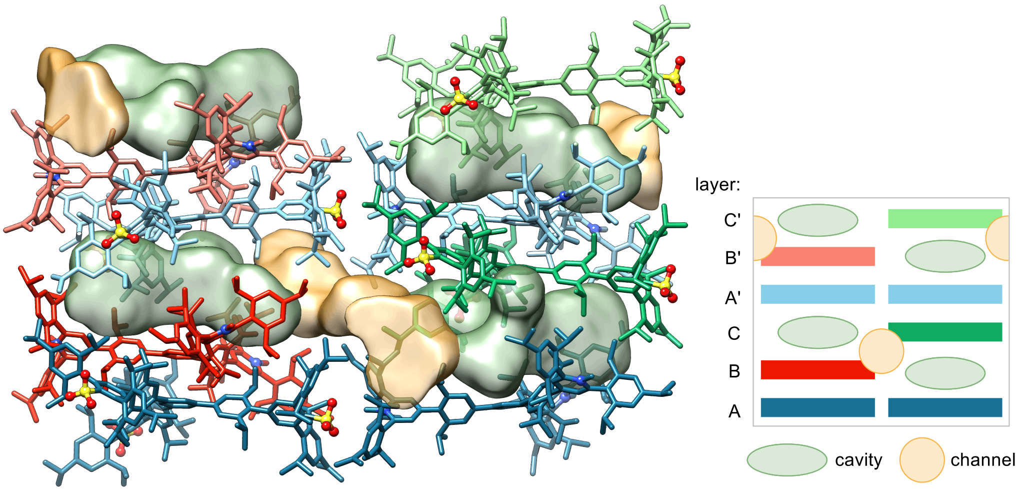

Figure S7. Structural analysis of **10**. The A', B', and C' layers are related through a crystallographic c-glide plane to the A, B, and C layers, respectively. The graphics were generated with UCSF Chimera[1] and converted to vector artwork with Adobe Illustrator. The calculation of pores was performed with 3V (outer probe radius, 10.0 Å; inner probe radius, 2.0 Å; the generated surfaces were smoothed for visualization).[2]

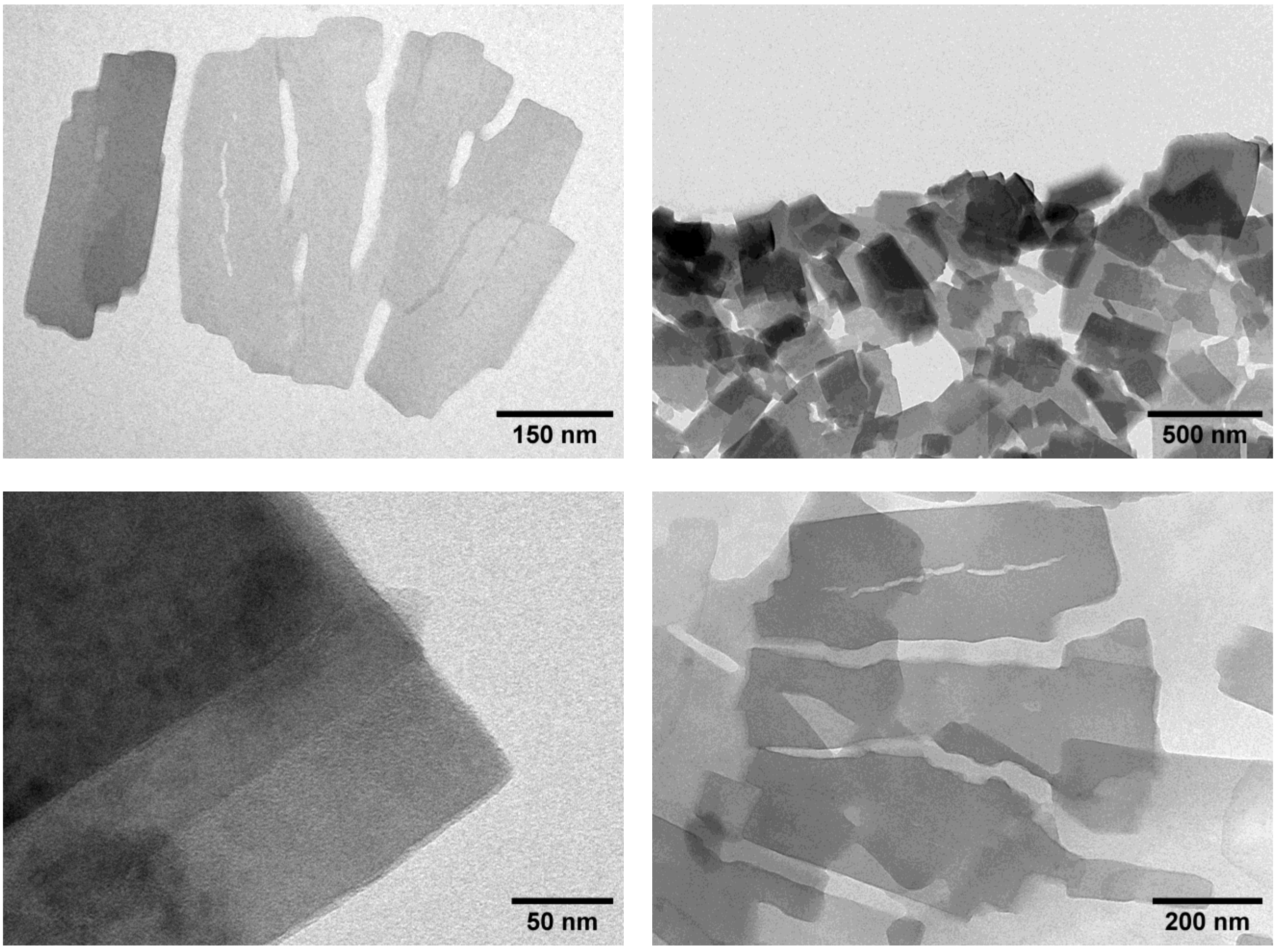

Figure S8. TEM images of **10** on a carbon surface.

2 Materials and Methods

2.1 General information

All reagents were purchased from commercial suppliers (Acros, Fluorochem, Alfa Aesar, Sigma-Aldrich, TCI) and used without further purification unless otherwise noted. Molecular sieves, Celite, and neutral alumina were dried under high vacuum overnight at 200-250 °C and kept in the glovebox. The reactions were carried out under an N_2 atmosphere, either on a Schlenk line or inside a glovebox (H_2O and O_2 levels below 1 ppm). Glassware was dried at 180 °C for at least 2 h or heated for a few minutes using a heat gun set at 450 °C. The solvents used in the glovebox and for reactions under an inert atmosphere were degassed and purified by distillation over the indicated drying agents, or were purchased from commercial suppliers and used without further drying, as indicated below: THF, Et_2O (K/benzophenone, stored over 4 Å molecular sieves), CH_2Cl_2 (CaH_2, stored over 4 Å molecular sieves), hexane, toluene, benzene (neutral dry alumina, stored over 4 Å molecular sieves). Alternatively, dry THF and CH_2Cl_2 were purchased from Acros, additionally degassed, and placed on freshly dried molecular sieves. Trimethyloxonium tetrafluoroborate, triethyloxonium tetrafluoroborate, and $[Ir(COD)OMe]_2$ were weighed into a vial inside a glovebox and added to reaction mixtures without exposure to air. Aqueous solutions of $NaBF_4$ were prepared fresh before use.

Reactions were monitored by thin-layer chromatography (TLC) analysis on Merck silica gel 60 F254 TLC plates, using visualization by UV irradiation. Aqueous workups were performed under air. Column chromatographic separations were performed using Merck 60 silica gel (40-63µm) and, unless otherwise stated, the samples were loaded as solutions in the eluent.

Nuclear magnetic resonance (NMR) spectra were recorded on Bruker Avance III 500 MHz, Bruker Avance NEO 500 MHz, and Bruker Avance III 400 MHz NMR spectrometers. For ROESY characterizations of ion pairs, dry C_6D_6, toluene-*d*$_8$, and $CDCl_3$ were used and the samples were prepared in a glove box. Otherwise, the deuterated solvents were purchased from Apollo Scientific and used from a freshly opened bottle. ^1H and ^{13}C chemical shifts (δ) are reported in ppm relative to tetramethylsilane, using solvent residual signals as the references: $CDCl_3$ (7.26 ppm for ^1H NMR and 77.0 ppm for ^{13}C NMR), C_6D_6 (7.16 ppm for ^1H NMR and 128.39 ppm for ^{13}C NMR). ^{19}F chemical shifts are given in ppm relative to $CFCl_3$ (external standard, absolute referencing). NMR data were processed using Mestrenova 14.1.2 or TopSpin 4.1.1. Data are reported as follows: chemical shift, multiplicity (s = singlet, d = doublet, t = triplet, q = quartet, quint = quintet, sext = sextet, hept = heptet, m = multiplet, br = broad signal), coupling constants (*J*) and integration.

2D-ROESY NMR spectra were acquired on a Bruker Avance III 500 MHz spectrometer. A pulse sequence with two purge 180° pulses of opposite phase (*roesyphpp.2*) was used to remove the undesirable TOCSY contributions to the integral intensities (mixing times p15 = 400 ms or 600 ms, spin-lock power γB_1 = 5000 Hz). The absence of spin diffusion was determined from the ROE build-up curve. In order to obtain approximate

distances for qualitative comparison, between twelve and fifteen ROE correlations were integrated and the distances were calculated, including the offset-compensated corrections, as described by Cadet *et al*.[3]

High resolution electron ionization mass spectrometry (HR-EI-MS): double-focusing (BE geometry) magnetic sector mass spectrometer DFS (ThermoFisher Scientific, Bremen, Germany); solid probe inlet; EI at 70 eV; source temperature 200 °C; acceleration voltage 5 kV; electric scan mode; mass range e.g. 300–350 m/z at 10'000 resolution (10% valley definition) and scan rate of 100–200 s per decade; mass accuracy ≤ 2 ppm after calibration with perfluorokerosene (PFK, Fluorochem, Derbyshire, UK). Liquid chromatography high resolution electrospray ionization mass spectrometry (LC-HR-ESI-MS): Acquity UPLC (Waters, Milford, USA) connected to an Acquity eλ diode array detector and a Synapt G2 HR-ESI-QTOF-MS (Waters, Milford, USA); injection of 1 μL sample (c = ca. 10-100 μg mL^{-1}); Acquity BEH C18 HPLC column (1.7 μm particle size, 2 × 50 mm, Waters) kept at 30 °C; elution at a flow rate of 400 μL min^{-1} with A: H_2O + 1% HCO_2H and B: CH_3CN + 0.1% HCO_2H, linear gradient from 5– 98% B within 5 min, then isocratic for 1 min;* UV spectra recorded from 200–600 nm at 1.2 nm resolution and 20 points s^{-1}; ESI: positive ionization mode, capillary voltage 3.0 kV, sampling cone 40V, extraction cone 4V, N_2 cone gas 4 L h^{-1}, N_2 desolvation gas 800 L min^{-1}, source temperature 120°C; mass analyzer in resolution mode: mass range 100– 2'000 m/z with a scan rate of 1 Hz; mass calibration < 2 ppm within 50–2'500 m/z with a 5mM aq. soln. of HCO_2Na, lockmasses: m/z 195.0882 (caffein, 0.7 ng mL^{-1}) and 556.2771 (Leucine-enkephalin, 2 ng mL^{-1}).

Single crystals suitable for X-ray diffraction analysis were grown under the conditions described for each case. A representative procedure for the handling of crystals: most of the mother liquor was removed from the crystals, a few of which were then picked up with a needle with the help of a drop of oil (Polybutenes, average Mn ~920 by VPO, isobutylene >90 %, viscosity 200 - 235 cSt(100 °C), Sigma-Aldrich). The selected crystals were placed on a glass slide, covered in oil, and immediately transferred to a diffractometer for analysis.

For the generation of the image for **C1$^+$A1$^-$** (Figure 2b), a CCDC database search was performed with ConQuest,[4] using N-methylpyridinium benzene sulfonate as a substructure search query. The hits were analysed in the Mercury software, where 20 structures were chosen arbitrarily (the refcodes are as follows: POXJAQ, NOQWUO, NOQWOI, IDODIQ02, GEJCEE, FAKBIC, EHAHIH, EHAHIH01, DUSRIT, DUMWEO, DOKSOM, DOKRUR, CIXCIY, CIRMAT, CIMCOS, CESBEI, CEFPEL, CEDVOX, CEDNEG, BUZSAR). All substituents at the pyridinium (usually annulated rings, halogens and small alkyl groups) and benzenesulfonate (usually alkyl groups at position 4) rings were removed and the cations in each structure were overlapped using Chemcraft.[5]

TEM imaging was performed with an FEI Tecnai G2 Spirit (Thermo Fisher Scientific, Eindhoven, The Netherlands) transmission electron microscope at 120 kV. The instrument was equipped with a side-mounted Gatan Orius 1000 CCD camera, and a digital micrograph acquisition software (Ametek GmbH, Unterschleissheim, Germany) was used. The samples were prepared by adding a few µl of a solution in CH_2Cl_2/$PhCF_3$ 1:1

($7.8 \cdot 10^{-5}$ M) onto a carbon coated Ni-grid (300 mesh, 5 nm of carbon). The sample was air dried and after 1 minute of incubation, any remaining solvent was completely removed by touching the side of the grid with filter paper. The sample was then coated unidirectionally with 2 or 2.5 nm of tungsten at an angle of 3° by electron beam evaporation in an MED 020 coater (Leica Microsystems, Vienna, Austria).

Thermal gravimetric analyses were carried out on a Netzsch STA 449 F3 Jupiter® Thermal Analysis System by heating typically 3-6 mg of the samples from 27 to 370 °C (compounds **3** and **5**) or from 27 to 700 °C (compound **10**) under argon atmosphere at a heating rate of 10 °C/min.

The melting points were measured on a Büchi M-560 Melting Point System at heating rate of 10 °C/min.

Abbreviations used: B_2pin_2, bis(pinacolato)diboron; DMF, *N,N*-dimethylformamide; dppf, 1,1'-bis(di-phenylphosphino)ferrocene; EtOAc, ethyl acetate; mCPBA, 3-chloroperbenzoic acid; Mes, mesityl (2,4,6-tri-methylphenyl); THF, tetrahydrofuran; COD, 1,5-cyclooctadiene; dtbbpy, 4,4′-di-*tert*-butyl-2,2′-dipyridyl; CV, column volume; DOSY, diffusion-ordered NMR spectroscopy; ROESY, rotating frame Overhauser enhancement NMR spectroscopy; ROE, rotating frame Overhauser effect.

2.2 Synthesis of cations C2$^+$ and C3$^+$

Synthesis of C2$^+$

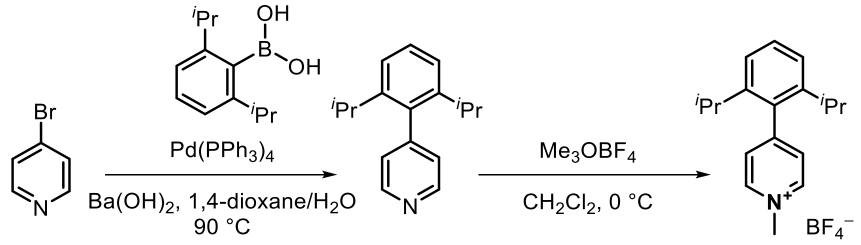

C2_N **C2$^+$BF$_4^-$**

4-(2,6-diisopropylphenyl)pyridine (C2_N)

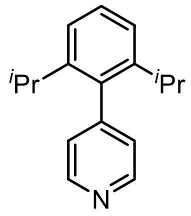

A 25 mL Schlenk flask was loaded with (2,6-diisopropylphenyl)boronic acid (200 mg, 0.97 mmol), 4-bromopyridine hydrochloride (189 mg, 0.97 mmol, 1 equiv.), Pd(PPh_3)$_4$ (56 mg, 0.049 mmol, 0.05 equiv.), and Ba(OH)$_2$·8H$_2$O (673 mg, 2.13 mmol, 2.2 equiv.) and was placed under an inert atmosphere with three vacuum/N_2 cycles. Degassed 1,4-dioxane (3 mL) and degassed H_2O (2 mL) were added and the resulting suspension was stirred at 90 °C for 18 h. The reaction mixture was allowed to cool to room temperature and NH_4Cl (sat. aq., 20 mL) and EtOAc (20 mL) were added. The layers were separated and the aqueous layer was extracted with EtOAc (3 x 20 mL). The combined organic extracts were washed with brine (30 mL), dried over Na_2SO_4, filtered, and the solvent was removed under reduced pressure. The residue was purified by column chromatography (40 g of silica gel, Et_2O/CH_2Cl_2 1:9) to give the title compound as an off-white solid (150 mg, 65% yield).

^{1}H NMR (400 MHz, $CDCl_3$) δ 8.69 – 8.64 (m, 2H), 7.38 (t, J = 8.1 Hz, 1H), 7.23 (d, *J* = 7.7 Hz, 2H), 7.17 – 7.14(m, 2H), 2.49 (hept, *J* = 6.9 Hz, 2H), 1.08 (d, *J* = 6.9 Hz, 12H).

^{13}C NMR (101 MHz, $CDCl_3$) δ 149.7, 149.5, 146.1, 136.7, 128.8, 125.2, 122.9, 30.5, 24.2.

HRMS (ESI+) (m/z): [M+H$^+$] calculated for $C_{17}H_{22}N$, 240.17468; found, 240.17451.

4-(2,6-diisopropylphenyl)-1-methylpyridin-1-ium tetrafluoroborate (C2$^+$BF$_4^-$)

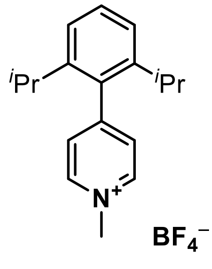

A 3 mL Schlenk flask was loaded with **C2_N** (30 mg, 0.125 mmol) and placed under an inert atmosphere with three vacuum/N_2 cycles. Dry CH_2Cl_2 (0.5 mL) was added and the mixture was cooled to 0 °C. Trimethyloxonium tetrafluoroborate (55.5 mg, 0.375 mmol, 3 equiv.) was added in one portion and the resulting suspension was stirred at 0 °C for 3 h. Then, *N,N*-diisopropylethylamine (0.065 mL, 0.375 mmol, 3 equiv.) was added, followed by the addition of trimethyloxonium tetrafluoroborate (55.5 mg, 0.375 mmol, 3 equiv.), and the mixture was warmed

to room temperature, and stirred for another 30 min. After that, it was separated between CH_2Cl_2 (5 mL) and H_2O (5 mL), the aqueous layer was extracted with CH_2Cl_2 (2 x 5 mL), the combined organic extracts were dried over Na_2SO_4, filtered, and the solvent was removed under reduced pressure. The residue was re-dissolved in CH_2Cl_2 (5 mL) and washed with $NaBF_4$ (10% aq. soln., 3 x 5 mL). The organic layer was dried over Na_2SO_4, filtered, and the solvent was evaporated under reduced pressure. The obtained white solid was washed with cold Et_2O (10 mL) and dried under high vacuum for 18 h to afford the title compound as an off-white solid (35 mg, 82% yield).

^{1}H NMR (400 MHz, $CDCl_3$) δ 8.89 (d, *J* = 6.2 Hz, 2H), 7.78 (d, *J* = 6.1 Hz, 2H), 7.45 (t, *J* = 7.8 Hz, 1H), 7.26 (d, *J* = 7.8 Hz, 3H), 4.57 (s, 3H), 2.33 (hept, *J* = 6.8 Hz, 2H), 1.09 (d, *J* = 6.8 Hz, 12H).

^{13}C NMR (101 MHz, $CDCl_3$) δ 160.2, 145.7, 145.0, 133.2, 130.6, 129.5, 123.6, 48.6, 30.9, 24.1.

^{19}F NMR (376 MHz, $CDCl_3$) δ -151.91, -151.96.

HRMS (ESI+) (m/z): [M^+] calculated for $C_{18}H_{24}N$, 254.19033; found, 254.19036.

Synthesis of C3$^+$

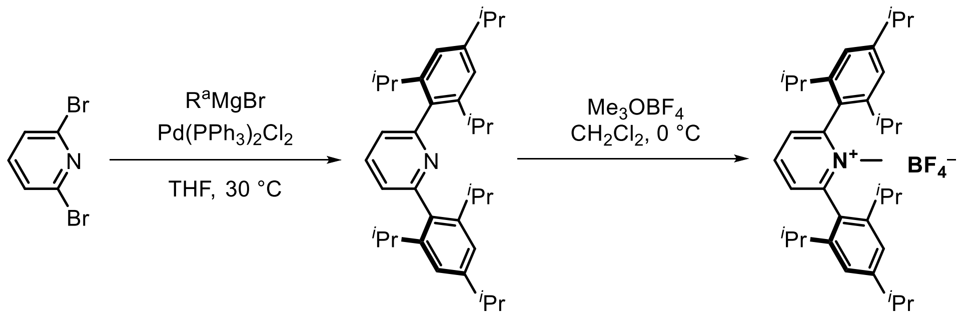

C3_N C3$^+$BF$_4^-$

2,6-bis(2,4,6-triisopropylphenyl)pyridine (C3_N)

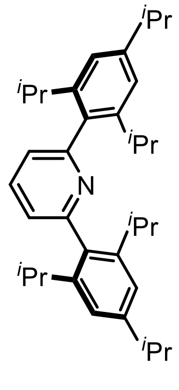

Part A. Magnesium (371 mg, 15.45 mmol, 2.57 equiv.) was placed in a Schlenk flask containing a stirring bar. The flask was closed with a septum, evacuated, flame-dried under vacuum for 1 min and left to cool down. Then, the flask was refilled with nitrogen and dry THF (2 mL) was added followed by slow addition of 1,2-dibromoethane (0.05 mL). To activate the magnesium, the mixture was repeatedly briefly heated to reflux with a heat gun. A solution of 1-bromo-2,4,6-triisopropylbenzene (3.74 g, 13.2 mmol, 2.2 equiv.) in dry THF (9 mL) was added dropwise, while maintaining the reflux of the reaction mixture. After that, the flask was sealed and the resulting mixture was stirred at 60 °C for an additional 6 h.

Part B. A separate 25 mL Schlenk flask was loaded with 2,6-dibromopyridine (1.42 mg, 6 mmol) and $Pd(PPh_3)_2Cl_2$ (84 mg, 0.12 mmol, 0.02 equiv.), placed under an inert atmosphere with three vacuum/N_2 cycles, and THF (13 mL) was added. The mixture was cooled to – 10 °C, and the Grignard reagent solution from *Part A* was added with a syringe. The resulting suspension was stirred at 60 °C for 15 h. Then, the mixture was cooled to 0 °C and filtered through a filter frit, the precipitate was washed with cold THF (15 mL), H_2O (5 x 20 mL), and dried under high vacuum, to give the title compound as a light greyish solid (2.48 g, 86% yield).

^{1}H NMR (400 MHz, $CDCl_3$) δ 7.75 (t, *J* = 7.7 Hz, 1H), 7.24 (d, *J* = 7.9 Hz, 2H), 7.02 (s, 4H), 2.89 (hept, *J* = 6.8 Hz, 2H), 2.58 (hept, *J* = 6.9 Hz, 4H), 1.25 (d, *J* = 6.9 Hz, 12H), 1.11 (d, *J* = 6.9 Hz, 12H), 1.10 (d, *J* = 6.9 Hz, 12H).

^{13}C NMR (101 MHz, $CDCl_3$) δ 160.1, 148.7, 146.2, 136.9, 135.4, 123.0, 120.6, 34.6, 30.6, 24.7, 24.3, 23.8.

HRMS (ESI+) (m/z): [$M+H^+$] calculated for $C_{35}H_{50}N$, 484.39378; found, 484.39670.

1-methyl-2,6-bis(2,4,6-triisopropylphenyl)pyridin-1-ium tetrafluoroborate ($C3^+BF_4^-$)

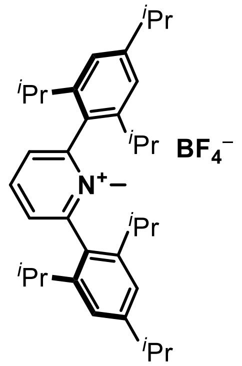

A 10 mL Schlenk flask was loaded with **C3_N** (242 mg, 0.5 mmol) and placed under an inert atmosphere with three vacuum/N_2 cycles. Dry CH_2Cl_2 (3 mL) was added and the mixture was cooled to 0 °C. Trimethyloxonium tetrafluoroborate (148 mg, 1 mmol, 2 equiv.) was added in one portion and the resulting suspension was stirred at 0 °C for 1 h. Then, H_2O (3 mL) was added, the aqueous layer was extracted with CH_2Cl_2 (10 mL, 2 x 5 mL), the combined organic extracts were dried over $MgSO_4$, filtered, and the solvent was removed under reduced pressure. The residue was purified by column chromatography (5 g of silica gel, CH_2Cl_2, then 5% *i*PrOH in CH_2Cl_2), the collected fractions were washed with $NaBF_4$ (10% aq. soln., 10 mL), filtered through a filter paper and the solvent was evaporated under reduced pressure to afford the title compound as a colourless solid (242 mg, 83% yield).

^{1}H NMR (400 MHz, $CDCl_3$) δ 9.12 (t, *J* = 7.9 Hz, 1H), 8.09 (d, *J* = 7.8 Hz, 2H), 7.20 (s, 4H), 3.53 (s, 3H), 2.98 (hept, *J* = 7.0 Hz, 2H), 2.11 (hept, *J* = 6.3 Hz, 4H), 1.31 (d, *J* = 6.9 Hz, 12H), 1.21 (d, *J* = 6.7 Hz, 12H), 1.18 (d, *J* = 6.9 Hz, 12H).

^{13}C NMR (101 MHz, $CDCl_3$) δ 157.1, 153.6, 147.8, 146.0, 130.8, 126.2, 122.8, 43.1, 34.7, 32.2, 24.9, 24.0, 23.5.

^{19}F NMR (376 MHz, $CDCl_3$) δ -153.6, -153.6.

HRMS (ESI+) (m/z): [M^+] calculated for $C_{36}H_{52}N$, 498.40943; found, 498.41309.

2.3 Synthesis of anions A2⁻ and A3⁻

Synthesis of A2⁻

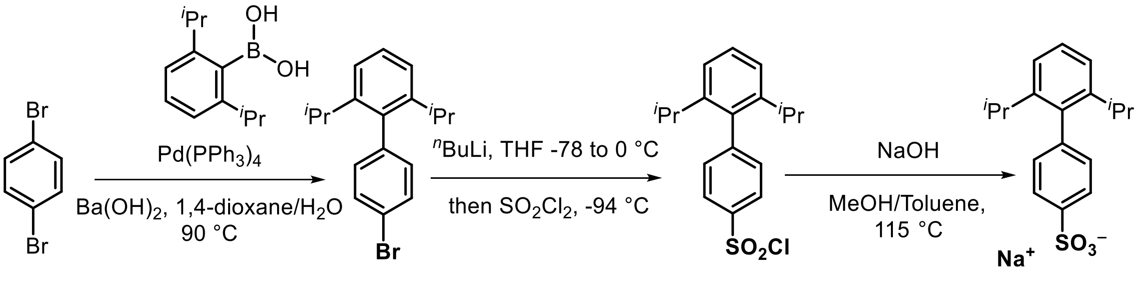

A2_Br A2_SO₂Cl Na⁺A2⁻

4'-bromo-2,6-diisopropyl-1,1'-biphenyl (A2_Br)

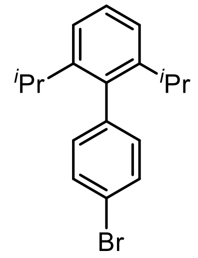

A 25 mL Schlenk flask was loaded with 1,4-dibromobenzene (229 mg, 0.97 mmol), (2,6-diisopropylphenyl)boronic acid (200 mg, 0.97 mmol, 1.0 equiv.), $Pd(PPh_3)_4$ (56 mg, 0.049 mmol, 0.05 equiv.), and $Ba(OH)_2 \cdot 8H_2O$ (367 mg, 1.16 mmol, 1.2 equiv.) and was placed under an inert atmosphere with three vacuum/N_2 cycles. Then, degassed 1,4-dioxane (3 mL) and degassed H_2O (2 mL) were added and the resulting suspension was stirred at 90 °C for 18 h. Then, the reaction mixture was allowed to cool to room temperature, poured into a sat. aq. solution of NH_4Cl (20 mL) and extracted with EtOAc (3 x 20 mL). The combined organic extracts were washed with brine (30 mL), dried over Na_2SO_4, filtered, and the solvent was removed under reduced pressure. The residue was purified by column chromatography (40 g of silica gel, hexanes, dry loading on 4 g of silica gel) to give the title compound as a colourless solid (180 mg, 59% yield).

^{1}H NMR (400 MHz, $CDCl_3$) δ 7.58 – 7.52 (m, 2H), 7.36 (t, *J* = 7.8 Hz, 1H), 7.21 (d, *J* = 7.8 Hz, 2H), 7.09 – 7.04(m, 2H), 2.58 (hept, *J* = 6.9 Hz, 2H), 1.09 (d, *J* = 6.9 Hz, 12H).

^{13}C NMR (101 MHz, $CDCl_3$) δ 146.9, 139.7, 138.3, 131.41, 131.35, 128.3, 122.7, 120.8, 30.4, 24.2.

HRMS (EI) (m/z): [M^+] calculated for $C_{18}H_{21}Br$, 316.08232; found, 316.08211.

2',6'-diisopropyl-[1,1'-biphenyl]-4-sulfonyl chloride (A2_SO₂Cl)

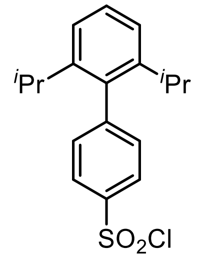

A2_Br (50 mg, 0.158 mmol) was placed in a flame-dried 3 mL Schlenk flask under a nitrogen atmosphere. Dry THF (0.5 mL) was added and the solution was cooled to -78 °C. *n*-BuLi (2.5 M in hexanes, 0.076 ml, 0.19 mmol, 1.2 equiv.) was added slowly with vigorous stirring. The reaction mixture was stirred for 1 h at -78 °C, then it was cooled to – 94 °C using an acetone/liquid N_2 bath and SO_2Cl_2 (32 mg, 0.019 mL, 0.237 mmol, 1.5 equiv.) was quickly added

in one portion. The reaction mixture was kept stirring at -94 °C for 10 min, then it was taken from the bath and allowed to warm to 0 °C. H_2O (7 mL) was added and the mixture was extracted with CH_2Cl_2 (3 x 10 mL). The combined organic fractions were washed with brine (20 mL), dried over Na_2SO_4, filtered, and concentrated under reduced pressure. The residue was purified by column chromatography (50 g of silica gel, hexanes/CH_2Cl_2 2:1), to provide the title compound (18 mg, 34%) as a yellowish solid.

^{1}H NMR (400 MHz, $CDCl_3$) δ 8.12 – 8.08 (m, 2H), 7.47 – 7.43 (m, 2H), 7.40 (t, *J* = 7.8 Hz, 1H), 7.25 (d, *J* = 7.8 Hz, 2H), 2.44 (hept, *J* = 7.0 Hz, 2H), 1.10 (d, *J* = 6.9 Hz, 12H).

^{13}C NMR (101 MHz, $CDCl_3$) δ 149.2, 146.3, 143.0, 136.9, 131.0, 129.1, 126.9, 123.1, 77.5, 77.2, 76.8, 30.6, 24.2.

HRMS (EI) (m/z): [M^+] calculated for $C_{18}H_{21}O_2ClS$, 336.09453; found, 336.09517.

Sodium 2',6'-diisopropyl-[1,1'-biphenyl]-4-sulfonate (Na^+A2^-)

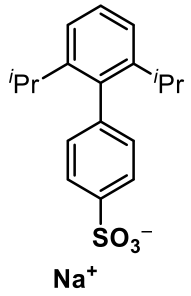

A round bottom flask equipped with a condenser was loaded with **A2_SO$_2$Cl** (30 mg, 0.089 mmol) and toluene (0.5 mL). Then, a solution of NaOH in methanol (2 M in MeOH, 2.5 mL, 50 equiv.) was added, and the reaction mixture was heated at reflux for 5 h. Then, the mixture was cooled to room temperature, diluted with H_2O (15 mL), and extracted with *i*PrOH/EtOAc 1:5 (3 x 15 mL). The combined extracts were washed with brine (20 mL), dried over Na_2SO_4, filtered, and concentrated under reduced pressure. The residue was dried *in vacuo* overnight to afford the title compound as a colourless solid (23 mg, 76% yield).

^{1}H NMR (500 MHz, CD_3OD) δ 7.91 (d, *J* = 7.7 Hz, 2H), 7.32 (t, *J* = 7.8 Hz, 1H), 7.22 (d, *J* = 7.6 Hz, 2H), 7.19 (d, *J* = 7.9 Hz, 2H), 2.56 (hept, *J* = 7.0 Hz, 2H), 1.06 (d, *J* = 6.9 Hz, 12H).

^{13}C NMR (126 MHz, CD_3OD) δ 147.7, 144.9, 144.4, 139.9, 130.6, 129.2, 126.9, 123.6, 31.4, 24.4.

HRMS (ESI-) (m/z): [M-Na^+] calculated for $C_{18}H_{21}O_3S$, 317.12059; found, 317.12936.

Synthesis of A3⁻

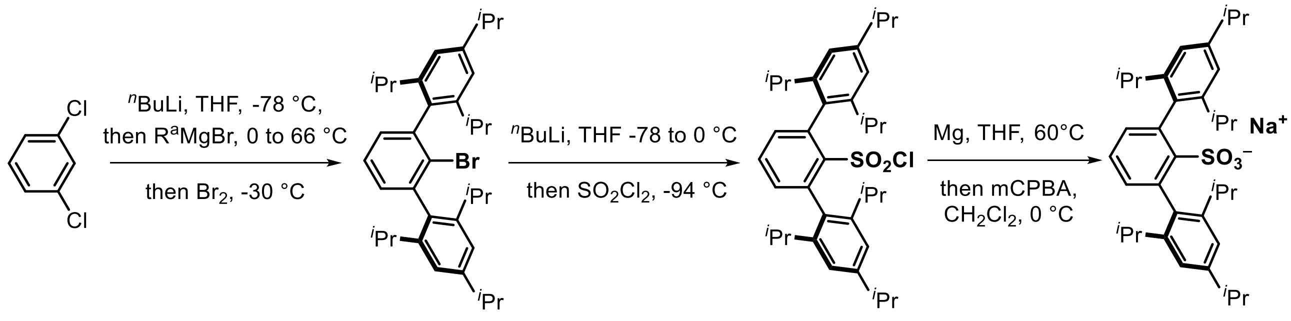

A3_Br **A3_SO$_2$Cl** **Na$^+$A3$^-$**

2'-bromo-2,2'',4,4'',6,6''-hexaisopropyl-1,1':3',1''-terphenyl (A3_Br)

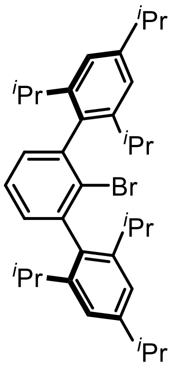

Part A. Magnesium (372 mg, 15.5 mmol, 2.9 equiv.) was placed in a Schlenk flask containing a stirring bar. The flask was closed with a septum, evacuated, dried with a heat gun under vacuum for 1 min and left to cool down to room temperature. Then, the flask was refilled with nitrogen and dry THF (1 mL) was added followed by slow addition of 1,2-dibromoethane (0.05 mL). To activate the magnesium, the mixture was repeatedly briefly heated to reflux with a heat gun. A solution of 1-bromo-2,4,6-triisopropylbenzene (3.75 g, 13.25 mmol, 2.5 equiv.) in dry THF (9 mL) was added dropwise while maintaining the reflux of the reaction mixture by occasionally heating the flask with a heat-gun. After that, the flask was sealed and the resulting mixture was stirred at 60 °C for an additional 3 h.

Part B. A separate 25 mL Schlenk flask was loaded with 1,3-dichlorobenzene (779 mg, 5.3 mmol) and was placed under an inert atmosphere with three vacuum/N_2 cycles. Dry THF (15 mL) was added, and the solution was cooled to -78 °C. *n*-BuLi (1.6 M in hexanes, 3.3 mL, 5.3 mmol, 1 equiv.) was added dropwise and the mixture was stirred at -78 °C for 1.5 h. Then, the Grignard reagent solution from *Part A* was quickly added with a syringe with vigorous stirring and the resulting solution was heated to 66 °C and stirred at that temperature for 3 h. After that, it was cooled to -30 °C and bromine (0.4 mL, 1.27 g, 7.95 mmol, 1.5 equiv.) was added, the mixture was stirred for 5 min before being warmed to 0 °C. Na_2SO_3 (sat. aq. soln., 5 mL) was added with vigorous stirring, the mixture was separated between H_2O (15 mL) and EtOAc (20 mL), the aqueous phase was extracted with EtOAc (2 x 20 mL), the combined organic layers were dried over $MgSO_4$, filtered, and the solvent was removed under reduced pressure. The crude material was recrystallized from boiling EtOAc (10 mL) to afford the title compound as a colourless solid (1.14 g, 38%).

^{1}H NMR (400 MHz, $CDCl_3$) δ 7.36 (t, *J* = 7.4 Hz, 1H), 7.17 (d, *J* = 7.5 Hz, 2H), 7.06 (s, 4H), 2.95 (hept, *J* = 6.8 Hz, 2H), 2.55 (hept, *J* = 6.9 Hz, 4H), 1.31 (d, *J* = 6.9 Hz, 12H), 1.16 (d, *J* = 6.9 Hz, 12H), 1.10 (d, *J* = 6.8 Hz, 12H).

^{13}C NMR (101 MHz, $CDCl_3$) δ 148.4, 146.0, 142.5, 136.7, 129.9, 128.6, 126.6, 120.8, 34.3, 31.0, 24.8, 24.2, 23.7.

HRMS (APCI+) (m/z): [$M+H^+$] calculated for $C_{36}H_{50}Br$, 561.3090; found, 561.3090.

2,2'',4,4'',6,6''-hexaisopropyl-[1,1':3',1''-terphenyl]-2'-sulfonyl chloride (A3_SO₂Cl)

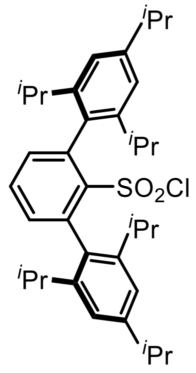

Compound **A3_Br** (562 mg, 1.0 mmol) was placed in a flame-dried Schlenk flask under a nitrogen atmosphere. Dry THF (10 mL) was added and the mixture was cooled to -78 °C. *n*-BuLi (1.6 M in hexanes, 1.56 ml, 2.5 mmol, 2.5 equiv.) was slowly added with vigorous stirring. The reaction mixture was stirred for 20 min at -78 °C and then placed in an ice-bath, and stirred at 0 °C for 15 min. The resulting suspension was cooled to -94 °C and SO_2Cl_2 (405 mg, 0.243 mL, 3 mmol, 3 equiv.) was quickly added in one portion. The reaction mixture was kept stirring at -94 °C for 20 min, then it was taken from the bath and allowed to warm to 0 °C. H_2O (5 mL) was added and the mixture was extracted with EtOAc (3 x 10 mL), the combined organic fractions were dried over $MgSO_4$, filtered, and concentrated under reduced pressure. The residue was purified by column chromatography (10 g of silica gel, hexanes 2 CV, then hexanes/CH_2Cl_2 95:5 2 CV, then 90:10 2 CV, then 85:15 2 CV) to give the title compound as a colourless solid (286 mg, 49% yield).

^{1}H NMR (400 MHz, $CDCl_3$) δ 7.64 (t, *J* = 7.6 Hz, 1H), 7.34 (d, *J* = 7.6 Hz, 2H), 7.05 (s, 4H), 2.94 (hept, *J* = 6.9 Hz, 2H), 2.48 (hept, *J* = 6.6 Hz, 4H), 1.30 (d, *J* = 6.9 Hz, 12H), 1.25 (d, *J* = 6.8 Hz, 12H), 1.05 (d, *J* = 6.8 Hz, 12H).

^{13}C NMR (101 MHz, $CDCl_3$) δ 148.9, 145.9, 142.21, 142.18, 133.9, 133.3, 132.6, 120.8, 34.3, 31.7, 25.6, 24.2, 23.0.

HRMS (ESI+) (m/z): [$M+Na^+$] calculated for $C_{36}H_{49}SO_2Cl$, 603.30340; found, 603.30292.

Sodium 2,2'',4,4'',6,6''-hexaisopropyl-[1,1':3',1''-terphenyl]-2'-sulfonate (Na^+A3^-)

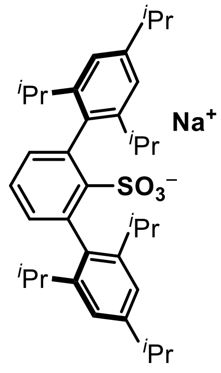

Magnesium (49 mg, 2 mmol, 4.5 equiv.) was placed in a 10 mL Schlenk flask containing a stirring bar. The flask was closed with a septum, evacuated, flame-dried under vacuum for 1 min and left to cool down to room temperature. Then, the flask was refilled with nitrogen and dry THF (0.3 mL) was added followed by slow addition of 1,2-dibromoethane (0.02 mL). To activate the magnesium, the mixture was repeatedly briefly heated to reflux with a heat gun. Then, it was heated to 60 °C and a solution of **A3_SO₂Cl** (276 mg, 0.447 mmol) in dry THF (3 mL) was added dropwise. The resulting mixture was stirred at 60 °C for an additional 1 h, then H_2O (2 mL) was added and the mixture was extracted with EtOAc (4 x 5 mL). The combined organic extracts were dried over $MgSO_4$, filtered, and concentrated under reduced pressure. The residue was dissolved in dry CH_2Cl_2 (5 mL) and the solution was cooled to 0 °C. Then, 3-chloroperoxybenzoic acid (77%, 125 mg, 0.558 mmol, 1.2 equiv.) was added and the reaction mixture was stirred at 0 °C for 0.5 h,

then it was diluted with CH_2Cl_2 (100 mL), washed with NaOH (1M aq. soln., 6 x 10 mL), dried over $MgSO_4$, filtered, and concentrated under reduced pressure. The residue was purified by column chromatography (10 g of silica gel, 5% *i*PrOH in CH_2Cl_2, 5 CV; then 10% *i*PrOH in CH_2Cl_2, 1 CV), the collected fractions were washed with NaOH (5% aq. soln., 2 x 10 mL), filtered through a filter paper, and concentrated under reduced pressure to give the title compound as a colourless solid (257 mg, 92% yield).

^{1}H NMR (400 MHz, $CDCl_3$) δ 7.30 (t, *J* = 7.5 Hz, 1H), 7.04 (d, *J* = 7.5 Hz, 2H), 6.92 (s, 4H), 2.85 (hept, *J* = 6.9 Hz, 2H), 2.54 (hept, *J* = 6.6 Hz, 4H), 1.24 (d, *J* = 6.9 Hz, 12H), 1.10 (d, *J* = 6.8 Hz, 12H), 0.96 (d, *J* = 6.9 Hz, 12H).

^{13}C NMR (101 MHz, $CDCl_3$) δ 147.4, 146.9, 141.0, 139.4, 137.9, 131.3, 128.1, 119.7, 34.1, 31.1, 25.3, 24.3, 23.1.

HRMS (ESI-) (m/z): [$(M-Na^+)^-$] calculated for $C_{36}H_{49}SO_3$, 561.33969; found, 561.34052.

2.4 Synthesis of cations present in 1-6

2,6-dichloro-4-(2,6-diisopropylphenyl)pyridine

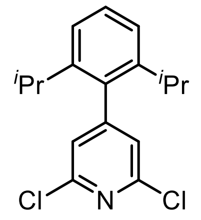

A 100 mL Schlenk flask was loaded with 2,6-dichloro-4-iodopyridine (1 g, 3.65 mmol), (2,6-diisopropylphenyl)boronic acid (0.827 g, 4.02 mmol, 1.1 equiv.), $Pd(PPh_3)_2Cl_2$ (0.128 g, 0.183 mmol, 0.05 equiv.), and $Ba(OH)_2{\cdot}8H_2O$ (2.35 g, 7.3 mmol, 2 equiv.) and was placed under an inert atmosphere with three vacuum/N_2 cycles. Degassed 1,4-dioxane (12 mL) and degassed H_2O (5 mL) were added and the resulting suspension was stirred at 90 °C for 18 h. The reaction mixture was allowed to cool to room temperature and NH_4Cl (sat. aq. soln. 50 mL) and EtOAc (50 mL) were added. The layers were separated and the aqueous layer was extracted with EtOAc (3 x 50 mL). The combined organic extracts were washed with brine (100 mL), dried over Na_2SO_4, filtered, and the solvent was removed under reduced pressure. The residue was purified by column chromatography (150 g of silica gel, hexanes/CH_2Cl_2 1:1) to give the title compound as a colourless solid (843 mg, 75% yield).

^{1}H NMR (500 MHz, $CDCl_3$) δ 7.39 (t, *J* = 7.8 Hz, 1H), 7.23 (d, *J* = 7.8 Hz, 2H), 7.13 (s, 2H), 2.46 (hept, *J* = 6.8 Hz, 2H), 1.11 (d, *J* = 6.8 Hz, 12H).

^{13}C NMR (126 MHz, $CDCl_3$) δ 155.3, 150.6, 146.0, 134.3, 129.5, 124.0, 123.2, 30.6, 24.2.

HRMS (ESI+) (m/z): [M+H$^+$] calculated for $C_{17}H_{20}NCl_2$, 308.09673; found, 308.09692.

4-(2,6-diisopropylphenyl)-2,6-bis(2,4,6-triisopropylphenyl)pyridine (C4_N)

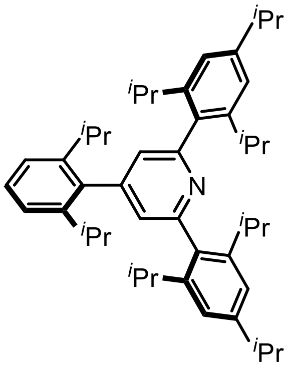

Part A. Magnesium (0.441 g, 18.4 mmol, 3.15 equiv.) was placed in a 100 mL Schlenk flask containing a stirring bar. The flask was closed with a septum, evacuated, flame-dried under vacuum for 1 min and left to cool down. Then, the flask was refilled with nitrogen and dry THF (2 mL) was added followed by slow addition of 1,2-dibromoethane (0.05 mL). To activate the magnesium, the mixture was repeatedly briefly heated to reflux with a heat gun. A solution of 1-bromo-2,4,6-triisopropylbenzene (3.97 g, 14 mmol, 2.4 equiv) in dry THF (15 mL) was added dropwise while maintaining the reflux of the reaction mixture. After that, the flask was sealed and the resulting mixture was stirred at 70 °C for an additional 24 h.

Part B. A 100 mL Schlenk flask was loaded with 2,6-dichloro-4-(2,6-diisopropylphenyl)pyridine (1.8 g, 5.84 mmol) and $Pd(PPh_3)_2Cl_2$ (0.246 g, 0.35 mmol, 0.06 equiv.) and was placed under an inert atmosphere with three vacuum/N_2 cycles. Dry THF (18 mL) was added, followed by quick addition of the Grignard reagent solution from *Part A* with vigorous stirring. The resulting suspension was stirred at 70 °C for 18 h. Then, the mixture was allowed to cool to room temperature, and NH_4Cl (sat. aq. soln. 100 mL) and EtOAc (100 mL) were added.

The layers were separated and the aqueous layer was extracted with EtOAc (3 x 100 mL). The combined organic extracts were washed with brine (100 mL), dried over Na_2SO_4, filtered, and the solvent was removed under reduced pressure. The residue was purified by column chromatography (200 g of silica gel, hexanes/CH_2Cl_2 1:1) to give the title compound as a colourless solid (3.4 g, 90% yield).

^{1}H NMR (500 MHz, $CDCl_3$) δ 7.37 (t, *J* = 7.7 Hz, 1H), 7.22 (d, *J* = 7.8 Hz, 2H), 7.15 (s, 2H), 7.03 (s, 4H), 2.91 (hept, *J* = 6.9 Hz, 2H), 2.83 – 2.68 (m, 6H), 1.26 (d, *J* = 6.9 Hz, 12H), 1.19 (d, *J* = 6.9 Hz, 12H), 1.13 (d, *J* = 6.9 Hz, 12H, overlapped), 1.12 (d, *J* = 6.9 Hz, 12H, overlapped).

^{13}C NMR (126 MHz, $CDCl_3$) δ 159.9, 148.9, 146.11, 146.09, 136.9, 136.7, 128.6, 124.5, 122.8, 120.6, 34.6, 30.9, 30.8, 24.7, 24.3, 24.2, 23.6.

HRMS (ESI+) (m/z): [M+H$^+$] calculated for $C_{47}H_{66}N$, 644.51898; found, 644.51916.

4-(2,6-diisopropylphenyl)-1-methyl-2,6-bis(2,4,6-triisopropylphenyl)pyridin-1-ium tetrafluoroborate ($C4^+BF_4^-$)

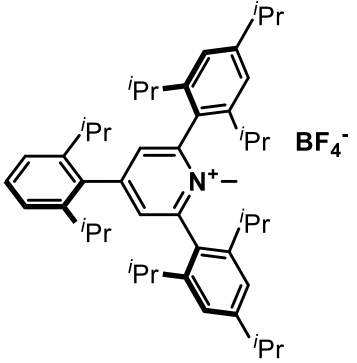

A 10 mL Schlenk flask was loaded with **C4_N** (200 mg, 0.311 mmol) and placed under an inert atmosphere with three vacuum/N_2 cycles. Dry CH_2Cl_2 (4 mL) was added and the mixture was cooled to 0 °C. Trimethyloxonium tetrafluoroborate (230 mg, 1.56 mmol, 5 equiv.) was added in one portion and the resulting suspension was stirred at 0 °C for 3 h. Then, *N,N*-diisopropylethylamine (201 mg, 0.271 mL, 1.56 mmol, 5 equiv.) was added, followed by the addition of trimethyloxonium tetrafluoroborate (230 mg, 1.56 mmol, 5 equiv), and the mixture was allowed to warm to room temperature, and stirred for another 30 min. After that, it was separated between CH_2Cl_2 (20 mL) and H_2O (20 mL), the aqueous layer was extracted with CH_2Cl_2 (2 x 20 mL), the combined organic extracts were dried over Na_2SO_4, filtered, and the solvent was removed under reduced pressure. The residue was purified by column chromatography (25 g of silica gel, CH_2Cl_2, 0.5 CV; then 10% *i*PrOH in CH_2Cl_2, 2 CV), the collected fractions were washed with $NaBF_4$ (10% aq. soln., 50 mL), filtered through a filter paper, and the solvent was evaporated under reduced pressure. The title compound was obtained as an off-white solid (198 mg, 85% yield).

^{1}H NMR (500 MHz, $CDCl_3$) δ 7.78 (s, 2H), 7.47 (t, *J* = 7.8 Hz, 1H), 7.28 (d, *J* = 7.8 Hz, 2H), 7.22 (s, 4H), 3.76 (s, 3H), 2.99 (hept, *J* = 6.9 Hz, 2H), 2.46 (hept, *J* = 6.8 Hz, 2H), 2.19 (hept, *J* = 6.8 Hz, 4H), 1.32 (d, *J* = 6.8 Hz, 12H), 1.30 (d, *J* = 6.9 Hz, 12H), 1.20 (d, *J* = 6.8 Hz, 12H), 1.17 (d, *J* = 6.8 Hz, 12H).

^{13}C NMR (126 MHz, $CDCl_3$) δ 159.6, 157.6, 154.2, 145.7, 145.0, 132.0, 131.04, 131.03, 125.5, 123.7, 123.0, 43.8, 34.6, 32.8, 31.7, 24.9, 24.1, 23.9, 23.4.

^{19}F NMR (471 MHz, $CDCl_3$) δ -154.47, -154.52.

HRMS (ESI+) (m/z): [M^+] calculated for $C_{48}H_{68}N$, 658.53463; found, 658.53302.

2,4,6-tris(2,4,6-triisopropylphenyl)pyridine (C5_N)

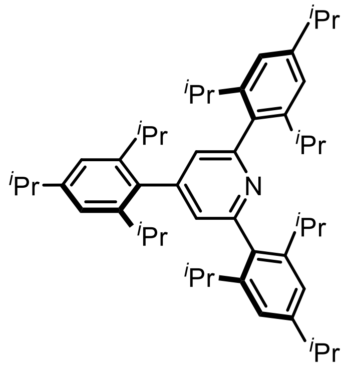

Part A. Magnesium (0.63 g, 26.2 mmol, 5.0 equiv.) was placed in a 100 mL Schlenk flask containing a stirring bar. The flask was closed with a septum, evacuated, flame-dried under vacuum for 1 min and left to cool down. Then, the flask was refilled with nitrogen and dry THF (2 mL) was added followed by slow addition of 1,2-dibromoethane (0.05 mL). To activate the magnesium, the mixture was repeatedly briefly heated to reflux with a heat gun. A solution of 1-bromo-2,4,6-triisopropylbenzene (5.39 g, 19.0 mmol, 3.6 equiv.) in dry THF (28 mL) was added dropwise while maintaining the reflux of the reaction mixture. After that, the flask was sealed and the resulting mixture was stirred at 70 °C for an additional 24 h.

Part B. A separate 100 mL Schlenk flask was loaded with 2,4,6-tribromopyridine (1.7 g, 5.28 mmol) and $Pd(PPh_3)_2Cl_2$ (0.371 g, 0.529 mmol, 0.1 equiv.) and was placed under an inert atmosphere with three vacuum/N_2 cycles. Dry THF (20 mL) was added, followed by quick addition of the Grignard reagent solution from *Part A* with vigorous stirring. The resulting suspension was stirred at 70 °C for 18 h. Then, the mixture was allowed to cool to room temperature, and NH_4Cl (sat. aq. soln. 100 mL) and EtOAc (100 mL) were added. The layers were separated and the aqueous layer was extracted with EtOAc (3 x 100 mL). The combined organic extracts were washed with brine (200 mL), dried over Na_2SO_4, filtered, and the solvent was removed under reduced pressure. The residue was purified by column chromatography (200 g of silica gel, hexanes/CH_2Cl_2 1:1) to give the title compound as a white solid (2.2 g, 61% yield).

^1H NMR (400 MHz, $CDCl_3$) δ 7.14 (s, 2H), 7.07 (s, 2H), 7.02 (s, 4H), 2.98 – 2.85 (hept, J = 6.9 Hz, 2H + hept, J = 6.9 Hz, 1H), 2.82 – 2.70 (hept, J = 6.9 Hz, 4H + hept, J = 6.9 Hz, 2H), 1.30 (d, J = 6.9 Hz, 6H), 1.25 (d, J = 6.9 Hz, 12H), 1.18 (d, J = 6.9 Hz, 12H), 1.13 (d, J = 6.9 Hz, 12H), 1.11 (d, J = 6.9 Hz, 12H).

^{13}C NMR (101 MHz, $CDCl_3$) δ 159.9, 148.9, 148.8, 148.7, 146.1, 145.9, 136.8, 134.6, 124.7, 120.8, 120.5, 34.6, 34.4, 30.84, 30.76, 24.7, 24.3, 24.21, 24.17, 23.6.

HRMS (ESI+) (m/z): [$M+H^+$] calculated for $C_{50}H_{72}N$, 686.56593; found, 686.56624.

1-methyl-2,4,6-tris(2,4,6-triisopropylphenyl)pyridin-1-ium tetrafluoroborate ($C5^+BF_4^-$)

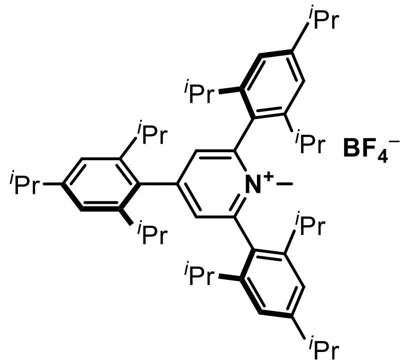

A 50 mL Schlenk flask was loaded with **C5_N** (1 g, 1.46 mmol) and placed under an inert atmosphere with three vacuum/N_2 cycles. Dry CH_2Cl_2 (15 mL) was added and the mixture was cooled to 0 °C. Trimethyloxonium tetrafluoroborate (1.08 g, 7.3 mmol, 5 equiv.) was added in one portion and the resulting suspension was stirred at 0 °C for 3 h. Then, *N,N*-diisopropylethylamine (0.943 g, 1.27 mL, 7.3 mmol, 5 equiv.) was added, followed by the addition of trimethyloxonium tetrafluoroborate (1.08 g, 7.3 mmol, 5 equiv), and the mixture was warmed to room temperature and stirred for another 30 min. After that, it was separated between CH_2Cl_2 (50 mL) and H_2O (50 mL), the aqueous layer was extracted with CH_2Cl_2 (2 x 50 mL), the combined organic extracts were dried over Na_2SO_4, filtered, and the solvent was removed under reduced pressure. The residue was purified by column chromatography (150 g of silica gel, CH_2Cl_2, 1 CV; then 10% *i*PrOH in CH_2Cl_2, 2 CV), the collected fractions were washed with $NaBF_4$ (10% aq. soln., 50 mL), filtered through a filter paper and the solvent was evaporated under reduced pressure. The obtained colourless solid was recrystallized from hot toluene/hexanes 1:10 (15 mL), the precipitate was washed with cold Et_2O (2 x 10 mL) and dried under high vacuum for 18 h to afford the title compound as an off-white solid (0.96 g, 83% yield).

^{1}H NMR (500 MHz, $CDCl_3$) δ 7.76 (s, 2H), 7.22 (s, 4H), 7.10 (s, 2H), 3.75 (s, 3H), 2.99 (hept, *J* = 6.9 Hz, 2H), 2.92 (hept, *J* = 6.9 Hz, 1H), 2.47 (hept, *J* = 6.9 Hz, 2H), 2.19 (hept, *J* = 6.9 Hz, 4H), 1.32 (d, *J* = 6.9 Hz, 12H), 1.30 (d, *J* = 6.9 Hz, 12H), 1.25 (d, *J* = 6.9 Hz, 6H), 1.19 (d, *J* = 6.9 Hz, 12H), 1.17 (d, *J* = 6.9 Hz, 12H).

^{13}C NMR (126 MHz, $CDCl_3$) δ 160.1, 157.4, 154.1, 151.9, 145.7, 145.0, 131.3, 129.7, 125.5, 123.0, 121.6, 43.7, 34.64, 34.57, 32.8, 31.7, 24.9, 24.1, 24.0, 23.9, 23.4.

^{19}F NMR (471 MHz, $CDCl_3$) δ -154.74, -154.79.

HRMS (ESI+) (m/z): [M^+] calculated for $C_{51}H_{74}N$, 700.58158; found, 700.58008.

Synthesis of R^b-Br

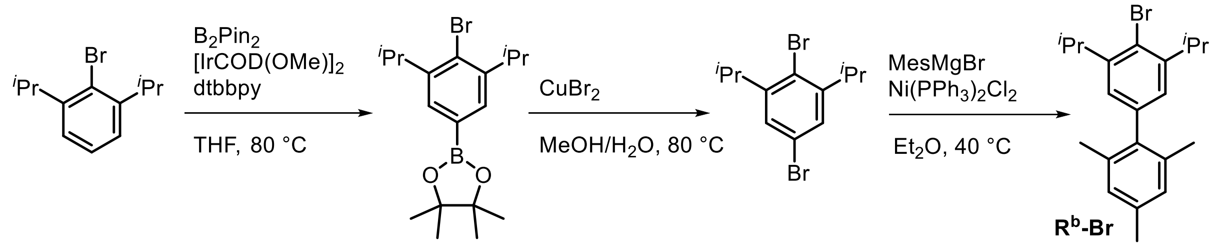

2-(4-bromo-3,5-diisopropylphenyl)-4,4,5,5-tetramethyl-1,3,2-dioxaborolane

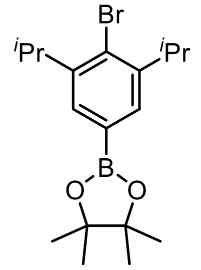

A flame dried 50 mL Schlenk flask was loaded with (1,5-cyclooctadiene)(methoxy)iridium(I) dimer (13.2 mg, 0.02 mmol, 0.0002 equiv.) in an N_2-filled glovebox. Then, the flask was taken out and bis(pinacolato)diboron (3.05 g, 12 mmol, 1.2 equiv.) and 4,4'-di-tert-butyl-2,2'-bi-pyridyl (10.8 mg, 0.04 mmol, 0.0004 equiv.) were added, followed by the addition of 2-bromo-1,3-diisopropylbenzene (2.41 g, 2.06 mL, 10 mmol) and dry THF (5 ml) under an N_2 atmosphere. The flask was sealed and the resulting mixture was stirred at 80 °C for 20 h. Then, the mixture was allowed to cool to room temperature and concentrated under reduced pressure. The residue was purified by column chromatography (30 g of silica gel, hexanes, 1.5 CV; then hexanes/EtOAc 95:5, 1.5 CV; then hex-anes/EtOAc 90:10, 1.5 CV) to give the title compound as a colourless solid (3.34 g, 91% yield).

^{1}H NMR (400 MHz, $CDCl_3$) δ 7.54 (s, 2H), 3.51 (hept, J = 6.8 Hz, 2H), 1.35 (s, 12H), 1.27 (d, J = 6.9 Hz, 12H).

^{13}C NMR (101 MHz, $CDCl_3$) δ 147.2, 130.7, 130.6, 84.0, 33.8, 25.0, 23.2.

HRMS (ESI+) (m/z): [M+NH_4^+] calculated for $C_{18}H_{32}BBrNO_2$, 384.17040; found, 384.17125.

2,5-dibromo-1,3-diisopropylbenzene

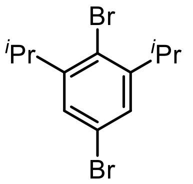

The reaction was performed under air. 2-(4-bromo-3,5-diisopropylphenyl)-4,4,5,5-tetra-methyl-1,3,2-dioxaborolane (6.72 g, 18.3 mmol) and $CuBr_2$ (12.3 g, 54.9 mmol, 3 equiv.) were placed in a 500 mL Schlenk flask. MeOH (130 mL) and H_2O (130 mL) were added, the flask was sealed, and the mixture was stirred at 80 °C for 7 days. Then, it was cooled to room temperature, diluted with H_2O (150 mL), and extracted with CH_2Cl_2 (3 x 200 mL). The combined organic extracts were dried over $MgSO_4$, filtered, and the solvent was removed under reduced pressure. The residue was purified by column chromatography (500 g of silica gel, hexanes) to give the title compound as a colourless oil (5.15 g, 88% yield). The ^{1}H NMR analysis was in accordance with the previously reported data.[6]

4'-bromo-3',5'-diisopropyl-2,4,6-trimethyl-1,1'-biphenyl (R^b-Br)

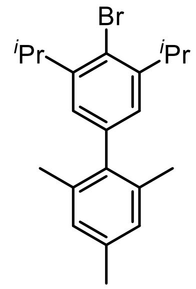

Part A. Magnesium (277 mg, 11.4 mmol, 2 equiv.) was placed in a Schlenk flask containing a stirring bar. The flask was closed with a septum, evacuated, flame-dried under vacuum for 1 min and left to cool to room temperature. Then, the flask was refilled with nitrogen and dry Et_2O (2 mL) was added, followed by slow addition of 1,2-dibromoethane (0.1 mL). To activate the magnesium, the mixture was repeatedly briefly heated to reflux with a heat gun. A solution of 2-bromo-1,3,5-trimethylbenzene (1.67 g, 8.39 mmol, 1.5 equiv.) in dry Et_2O (8 mL) was added dropwise while maintaining the reflux of the reaction mixture. After that, the flask was sealed and the resulting mixture was stirred at 40 °C for an additional 23 h.

Part B. A separate 100 mL Schlenk flask was placed under an N_2 atmosphere and loaded with a solution of the Grignard reagent from *Part A*. 2,5-Dibromo-1,3-diisopropylbenzene (1.79 g, 5.59 mmol) was added, followed by the addition of $Ni(PPh_3)_2Cl_2$ (183 mg, 0.28 mmol, 0.05 equiv.) and the resulting suspension was stirred at 40 °C for 6 h. Then, the mixture was allowed to cool to room temperature, NH_4Cl (sat. aq. soln. 10 mL) and H_2O (10 mL) were added, and the mixture was extracted with EtOAc (20 mL, then 2 x 5 mL). The combined organic extracts were dried over $MgSO_4$, filtered, and the solvent was removed under reduced pressure. The residue was purified by column chromatography (100 g of silica gel, hexanes) to give the title compound as a colourless solid (1.26 g, 63% yield).

^{1}H NMR (400 MHz, $CDCl_3$) δ 6.97 (s, 2H), 6.92 (s, 2H), 3.55 (hept, *J* = 6.7 Hz, 2H), 2.35 (s, 3H), 2.02 (s, 6H), 1.25 (d, *J* = 6.8 Hz, 12H).

^{13}C NMR (101 MHz, $CDCl_3$) δ 147.8, 140.0, 138.9, 136.9, 136.1, 128.3, 125.4, 124.6, 33.7, 23.3, 21.2, 20.9.

HRMS (EI+) (m/z): [M^+] calculated for $C_{21}H_{27}Br$, 358.12907; found, 358.12974.

2,6-bis(3,5-diisopropyl-2',4',6'-trimethyl-[1,1'-biphenyl]-4-yl)-4-(2,6-diisopropylphenyl)pyridine (C6_N)

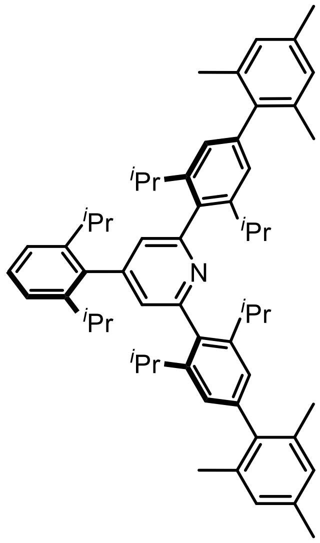

Part A. Magnesium (108 mg, 4.5 mmol, 3 equiv.) was placed in a Schlenk flask containing a stirring bar. The flask was closed with a septum, evacuated, flame-dried under vacuum for 1 min and left to cool down. Then, the flask was refilled with nitrogen and dry THF (0.3 mL) was added followed by slow addition of 1,2-dibromoethane (0.02 mL). To activate the magnesium, the mixture was repeatedly briefly heated to reflux with a heat gun. A solution of R^b-Br (1.29 g, 3.6 mmol, 2.4 equiv.) in dry THF (3 mL) was added dropwise while maintaining the reflux of the reaction mixture. After that, the flask was sealed and the resulting mixture was stirred at 60 °C for an additional 4 h.

Part B. A separate 25 mL Schlenk flask was loaded with 2,6-dichloro-4-(2,6-diisopropylphenyl)pyridine (462 mg, 1.5 mmol, prepared as described above) and $Pd(PPh_3)_2Cl_2$ (53 mg, 0.075 mmol, 0.05 equiv.) and was placed under an inert atmosphere with three vacuum/N_2 cycles. The Grignard regent solution from *Part A* was added, followed by THF (1 mL), and the resulting suspension was stirred at 80 °C for 24 h. Then, the mixture was allowed to cool to room temperature, and H_2O (10 mL) and CH_2Cl_2 (20 mL) were added. The layers were separated and the aqueous layer was extracted with CH_2Cl_2 (2 x 10 mL). The combined organic extracts were dried over $MgSO_4$, filtered, and the solvent was removed under reduced pressure. The residue was purified by column chromatography (50 g of silica gel, hexanes/CH_2Cl_2 7:3) to give the title compound as a colourless solid (1.12 g, 94% yield).

^{1}H NMR (400 MHz, $CDCl_3$) δ 7.37 (t, *J* = 7.7 Hz, 1H), 7.27 (s, 2H), 7.24 (d, *J* = 7.8 Hz, 2H), 7.01 (s, 4H), 6.98 (s, 4H), 2.91 – 2.78 (m, 6H), 2.35 (s, 6H), 2.06 (s, 12H), 1.22 (d, *J* = 6.9 Hz, 12H), 1.18 (d, *J* = 6.9 Hz, 12H), 1.13 (d, *J* = 6.9 Hz, 12H).

^{13}C NMR (101 MHz, $CDCl_3$) δ 160.0, 146.4, 146.1, 140.8, 139.9, 137.1, 136.8, 136.6, 136.4, 136.2, 128.6, 128.3, 128.2, 124.8, 123.7, 122.8, 30.9, 30.8, 24.7, 24.2, 23.6, 21.2, 21.1, 21.0.

HRMS (ESI+) (m/z): [M+H$^+$] calculated for $C_{59}H_{74}N$, 796.58158; found, 796.58026.

2,6-bis(3,5-diisopropyl-2',4',6'-trimethyl-[1,1'-biphenyl]-4-yl)-4-(2,6-diisopropylphenyl)-1-methylpyridin-1-ium tetrafluoroborate (C6$^+$BF$_4^-$)

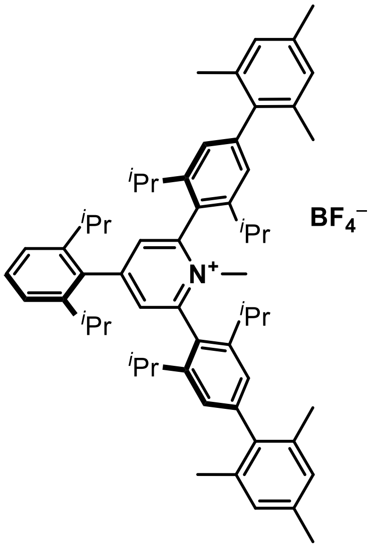

A 10 mL Schlenk flask was loaded with **C6_N** (156 mg, 0.19 mmol) and placed under an inert atmosphere with three vacuum/N_2 cycles. Dry CH_2Cl_2 (2 mL) was added and the mixture was cooled to 0 °C. Trimethyloxonium tetrafluoroborate (35 mg, 0.23 mmol, 1.2 equiv) was added in one portion and the resulting suspension was stirred at 0 °C for 1 h. Then, H_2O (3 mL) was added, and the mixture was extracted with CH_2Cl_2 (3 x 5 mL), the combined organic extracts were dried over $MgSO_4$, filtered, and the solvent was removed under reduced pressure. The residue was purified by column chromatography (10 g of silica gel, CH_2Cl_2, 5 CV; then 10% *i*PrOH in CH_2Cl_2, 5 CV), the collected fractions were washed with $NaBF_4$ (10% aq. soln., 50 mL), filtered through a filter paper, and the solvent was removed under reduced pressure to afford the title compound as a yellowish solid (165 mg, 94% yield).

^{1}H NMR (400 MHz, $CDCl_3$) δ 7.97 (s, 2H), 7.49 (t, *J* = 7.9 Hz, 1H), 7.31 (d, *J* = 7.9 Hz, 2H), 7.23 (s, 4H), 6.99 (s, 2H), 6.97 (s, 2H), 3.91 (s, 3H), 2.53 (hept, *J* = 6.9 Hz, 2H), 2.34 (s, 6H), 2.28 (hept, *J* = 6.7 Hz, 4H), 2.05 (s, 6H), 2.02 (s, 6H), 1.35 (d, *J* = 6.8 Hz, 12H), 1.23 (d, *J* = 6.7 Hz, 24H).

^{13}C NMR (101 MHz, $CDCl_3$) δ 160.0, 157.5, 146.1, 145.1, 137.7, 137.6, 136.2, 135.3, 132.0, 131.1, 128.7, 128.4, 126.2, 126.1, 123.7, 44.1, 32.9, 31.9, 25.0, 24.1, 23.4, 21.2, 20.9.

^{19}F NMR (376 MHz, $CDCl_3$) δ -154.26, -154.31.

HRMS (ESI+) (m/z): [M$^+$] calculated for $C_{60}H_{76}N$, 810.59723; found, 810.59171.

Synthesis of R^c-Br

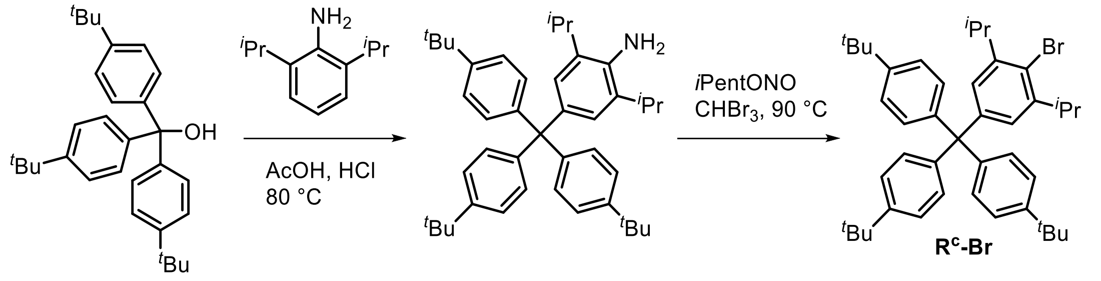

2,6-diisopropyl-4-(tris(4-(*tert*-butyl)phenyl)methyl)aniline

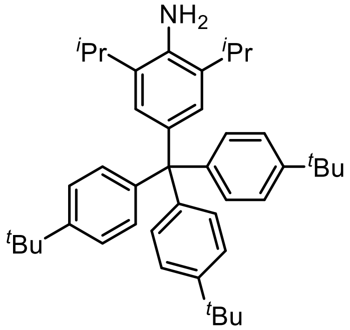

Tris(4-(*tert*-butyl)phenyl)methanol[7] (15.6 g, 36.4 mmol) was dissolved in acetic acid (220 mL) and the solution was degassed by purging with N_2. Then, 2,6-diisopropylaniline (9.68 g, 10.3 mL, 54.6 mmol, 1.5 equiv.) was added, followed by the addition of concentrated HCl (3.3 mL). The reaction mixture was stirred at 80 °C under an N_2 atmosphere for 8 h, then allowed to cool to room temperature and stirred for further 18 h. KOH (40 mL, 2 M aq. soln.) was added and the formed precipitate was isolated by filtration, washed with H_2O (3 x 100 mL), MeOH (50 mL, 2 x 25 mL), and dried under vacuum to afford the title compound as a colourless solid (18.3 g, 86% yield). The ^{1}H NMR analysis was in accordance with the previously reported data.[8]

4,4',4''-((4-bromo-3,5-diisopropylphenyl)methanetriyl)tris(*tert*-butylbenzene) (R^c-Br)

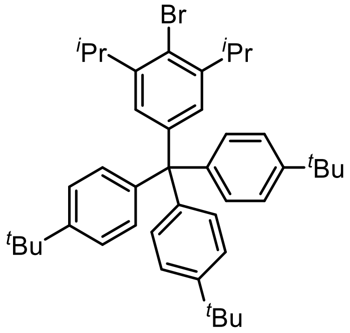

Isopentyl nitrite (1.17 g, 1.34 mL, 10 mmol, 5 equiv.) was added dropwise over 3 min to a stirred degassed solution of 2,6-diisopropyl-4-(tris(4-(*tert*-butyl)phenyl)methyl)aniline (1.18 g, 2 mmol) in $CHBr_3$ at 90 °C. The reaction mixture was stirred for 1 h and cooled to room temperature. Then, silica gel (2 g) was added and the mixture was concentrated under reduced pressure. The residue was loaded directly on a silica gel column (40 g) and purified by column chromatography (hexanes) to afford the title compound as a colourless solid (860 mg, 66% yield).

^{1}H NMR (400 MHz, $CDCl_3$) δ 7.26 – 7.22 (m, 6H), 7.11 – 7.05 (m, 6H), 6.86 (s, 2H), 3.41 (hept, *J* = 7.0 Hz, 2H), 1.31 (s, 27H), 1.04 (d, *J* = 6.8 Hz, 12H).

^{13}C NMR (101 MHz, $CDCl_3$) δ 148.7, 146.5, 146.0, 143.8, 130.9, 127.8, 124.1, 123.7, 63.8, 34.5, 33.5, 31.5, 23.0.

HRMS (EI+) (m/z): [M$^+$] calculated for $C_{43}H_{55}Br$, 650.34817; found, 650.34710.

2,6-bis(2,6-diisopropyl-4-(tris(4-(*tert*-butyl)phenyl)methyl)phenyl)-4-(2,6-diisopropylphenyl)pyridine (C7_N)

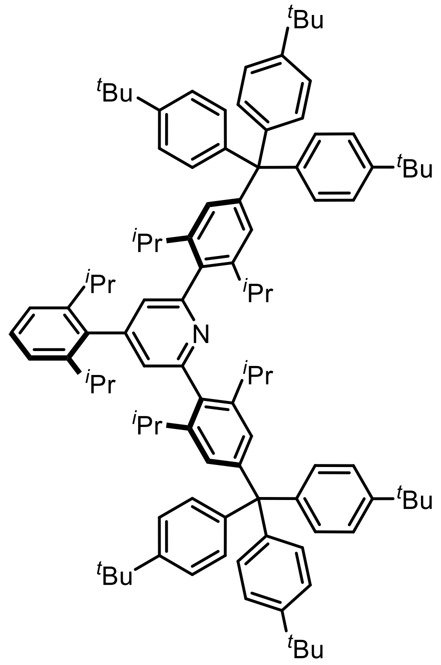

Part A. Magnesium (66 mg, 2.75 mmol, 3.6 equiv.) was placed in a Schlenk flask containing a stirring bar. The flask was closed with a septum, evacuated, flame-dried under vacuum for 1 min and left to cool down. Then, the flask was refilled with nitrogen and dry THF (0.3 mL) was added followed by slow addition of 1,2-dibromoethane (0.02 mL). To activate the magnesium, the mixture was repeatedly briefly heated to reflux with a heat gun. A solution of **R^c-Br** (1.17 g, 1.8 mmol, 2.4 equiv.) in dry THF (5 mL) was added dropwise while maintaining the reflux. After that, the flask was sealed and the resulting mixture was stirred at 60 °C for an additional 19 h.

Part B. A separate 25 mL Schlenk flask was loaded with 2,6-dichloro-4-(2,6-diisopropylphenyl)pyridine (231 mg, 0.75 mmol, prepared as above) and $Pd(PPh_3)_2Cl_2$ (26 mg, 0.0375 mmol, 0.05 equiv.) and was placed under an inert atmosphere with three vacuum/N_2 cycles. The Grignard reagent solution from *Part A* was added, followed by THF (1 mL), and the resulting suspension was stirred at 80 °C for 24 h. Then, the mixture was allowed to cool to room temperature, H_2O (10 mL) and CH_2Cl_2 (20 mL) were added, the layers were separated, and the aqueous layer was extracted with CH_2Cl_2 (2 x 10 mL). The combined organic extracts were dried over $MgSO_4$, filtered, and the solvent was removed under reduced pressure. The residue was purified by column chromatography (40 g of silica gel, hexanes/CH_2Cl_2 9:1) to give the title compound as a colourless solid (0.99 g, 96% yield).

^{1}H NMR (400 MHz, $CDCl_3$) δ 7.34 (t, *J* = 7.7 Hz, 1H), 7.25 – 7.21 (m, 12H), 7.20 (d, *J* = 7.7 Hz, 2H), 7.17 – 7.12 (m, 14H), 6.92 (s, 4H), 2.75 (d, *J* = 6.9 Hz, 2H), 2.66 (hept, *J* = 6.8 Hz, 4H), 1.30 (s, 54H), 1.12 (d, *J* = 6.8 Hz, 12H), 0.96 (d, *J* = 6.8 Hz, 12H), 0.93 (d, *J* = 6.8 Hz, 12H).

^{13}C NMR (101 MHz, $CDCl_3$) δ 160.0, 148.7, 148.3, 147.2, 146.1, 144.7, 144.3, 136.9, 136.1, 131.0, 128.5, 125.9, 124.6, 124.0, 122.7, 64.2, 34.4, 31.5, 30.8, 30.7, 24.5, 24.2, 23.3.

HRMS (ESI+) (m/z): [M+H$^+$] calculated for $C_{103}H_{130}N$, 1381.01978; found, 1381.01477.

2,6-bis(2,6-diisopropyl-4-(tris(4-(*tert*-butyl)phenyl)methyl)phenyl)-4-(2,6-diisopropylphenyl)-1-methylpyridin-1-ium tetrafluoroborate (C7$^+$BF$_4^-$)

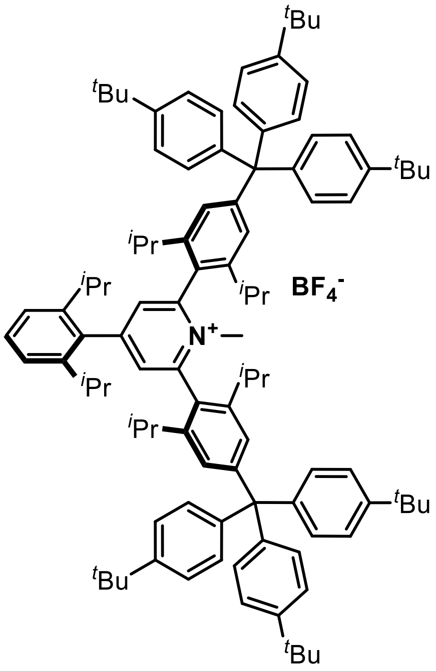

A 10 mL Schlenk flask was loaded with **C7_N** (276 mg, 0.2 mmol) and placed under an inert atmosphere with three vacuum/N_2 cycles. Dry CH_2Cl_2 (2 mL) was added and the mixture was cooled to 0 °C. Trimethyloxonium tetrafluoroborate (36 mg, 0.24 mmol, 1.2 equiv) was added in one portion and the resulting suspension was stirred at 0 °C for 1 h. Then, H_2O (3 mL) was added, the mixture was extracted with CH_2Cl_2 (3 x 5 mL), and the combined organic extracts were dried over $MgSO_4$, filtered, and the solvent was removed under reduced pressure. The residue was purified by column chromatography (10 g of silica gel, CH_2Cl_2, 5 CV; then 10% *i*PrOH in CH_2Cl_2, 5 CV), the collected fractions were washed with $NaBF_4$ (10% aq. soln., 50 mL), filtered through a filter paper, and the solvent was evaporated under reduced pressure to afford the title compound as a colourless solid (250 mg, 84% yield).

^{1}H NMR (400 MHz, $CDCl_3$) δ 7.85 (s, 2H), 7.46 (t, *J* = 7.8 Hz, 1H), 7.31 – 7.26 (m, 14H), 7.18 (s, 4H), 7.09 (d, *J* = 8.9 Hz, 12H), 3.83 (s, 3H), 2.45 (hept, *J* = 6.8 Hz, 2H), 2.15 (hept, *J* = 6.8 Hz, 4H), 1.30 (s, 54H), 1.17 (d, *J* = 6.8 Hz, 12H), 1.13 (d, *J* = 6.8 Hz, 12H), 1.01 (d, *J* = 6.7 Hz, 12H).

^{13}C NMR (101 MHz, $CDCl_3$) δ 159.8, 157.5, 152.9, 149.1, 145.0, 144.4, 143.0, 132.0, 131.02, 130.96, 130.8, 128.1, 125.1, 124.5, 123.7, 64.3, 43.9, 34.5, 32.7, 31.8, 31.5, 24.6, 24.1, 23.2.

^{19}F NMR (376 MHz, $CDCl_3$) δ -154.3, -154.4.

HRMS (ESI+) (m/z): [M$^+$] calculated for $C_{104}H_{132}N$, 1395.03543; found, 1395.03369.

2.5 Synthesis of anions present in 1-6

3',5'-dichloro-2,6-diisopropyl-1,1'-biphenyl

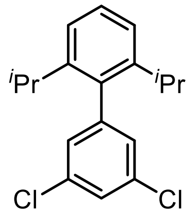

A 250 mL Schlenk flask was loaded with 3,5-dichloroiodobenzene (4.8 g, 17.6 mmol), (2,6-diisopropylphenyl)boronic acid (4 g, 19.4 mmol, 1.1 equiv.), $Pd(PPh_3)_2Cl_2$ (1.24 g, 1.76 mmol, 0.1 equiv.), and $Ba(OH)_2{\cdot}8H_2O$ (11.1 g, 35.2 mmol, 2 equiv.) and was placed under an inert atmosphere with three vacuum/N_2 cycles. Then, degassed 1,4-dioxane (70 mL) and degassed H_2O (30 mL) were added and the resulting suspension was stirred at 90 °C for 18 h. The reaction mixture was allowed to cool to room temperature, poured into a sat. aq. solution of NH_4Cl (150 mL), and extracted with CH_2Cl_2 (3 x 150 mL). The combined organic extracts were washed with brine (300 mL), dried over Na_2SO_4, filtered, and the solvent was removed under reduced pressure. The residue was purified by column chromatography (300 g of silica gel, hexanes) to give the title compound as a colourless solid (4.96 g, 92% yield).

^{1}H NMR (500 MHz, $CDCl_3$) δ 7.39 (t, *J* = 1.9 Hz, 1H, overlapped), 7.36 (t, *J* = 7.8 Hz, 1H, overlapped), 7.21 (d, *J* = 7.7 Hz, 2H), 7.09 (d, *J* = 1.9 Hz, 2H), 2.56 (hept, *J* = 6.9 Hz, 2H), 1.11 (d, *J* = 6.9 Hz, 12H).

^{13}C NMR (126 MHz, $CDCl_3$) δ 146.7, 144.0, 136.8, 134.8, 128.8, 128.1, 127.2, 122.9, 30.5, 24.3.

HRMS (EI+) (m/z): [M^+] calculated for $C_{18}H_{20}Cl_2$, 306.0942; found, 306.09327.

2'-bromo-5'-(2,6-diisopropylphenyl)-2,2'',4,4'',6,6''-hexaisopropyl-1,1':3',1''-terphenyl (A4_Br)

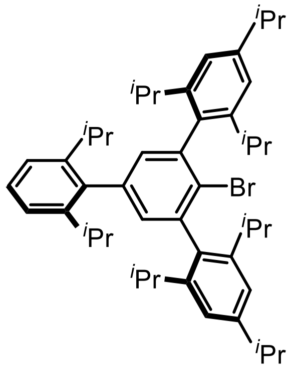

Part A. Magnesium (0.448 g, 18.4 mmol, 2.8 equiv.) was placed in a 100 mL Schlenk flask containing a stirring bar. The flask was closed with a septum, evacuated, dried with a heat-gun under vacuum for 1 min and left to cool to room temperature. Then, the flask was refilled with nitrogen and dry THF (6 mL) was added followed by slow addition of 1,2-dibromoethane (0.05 mL). To activate the magnesium, the mixture was repeatedly briefly heated to reflux with a heat gun. A solution of 1-bromo-2,4,6-triisopropylbenzene (4.06 g, 14.3 mmol, 2.2 equiv.) in dry THF (30 mL) was added dropwise while maintaining the reflux by heating the flask with a heat-gun occasionally. After that, the flask was sealed and the resulting mixture was stirred at 70 °C for an additional 24 h.

Part B. A separate 100 mL Schlenk flask was loaded with 3',5'-dichloro-2,6-diisopropyl-1,1'-biphenyl (2 g, 6.51 mmol) and was placed under an inert atmosphere with three vacuum/N_2 cycles. Dry THF (20 mL) was added, and the solution was cooled to -78 °C. *n*-BuLi (2.5 M in hexanes, 2.6 mL, 6.51 mmol, 1 equiv.) was added dropwise and the mixture was stirred at -78 °C for 1 h. Then, the Grignard reagent solution from *Part A* was quickly added through a cannula with vigorous stirring. The resulting solution was heated to 70 °C and stirred at that temperature for 3 h. Afterwards, it was cooled to -50 °C and bromine (1.25 g, 7.81 mmol, 1.2 equiv.)

was added. The mixture was stirred for 10 min at -50 °C, then poured into a sat. aq. solution of Na_2SO_3 (150 mL) and extracted with CH_2Cl_2 (3 x 100 mL). The combined organic layers were washed with brine (150 mL), dried over Na_2SO_4, filtered, and the solvent was removed under reduced pressure. The residue was purified by column chromatography (300 g of silica gel, hexanes to hexanes/CH_2Cl_2 9:1) to give the title compound as a colourless solid (3.55 g, 75% yield).

^{1}H NMR (400 MHz, $CDCl_3$) δ 7.31 (t, *J* = 7.7 Hz, 1H), 7.17 (d, *J* = 7.7 Hz, 2H), 7.06 (s, 4H), 7.04 (s, 2H), 2.95 (hept, *J* = 6.9 Hz, 2H), 2.86 (hept, *J* = 6.8 Hz, 2H), 2.72 (hept, *J* = 6.9 Hz, 4H), 1.31 (d, *J* = 7.0 Hz, 12H), 1.23 (d, *J* = 6.9 Hz, 12H), 1.11 (d, *J* = 6.9 Hz, 24H).

^{13}C NMR (101 MHz, $CDCl_3$) δ 148.5, 146.7, 146.0, 142.2, 139.3, 138.1, 136.4, 131.2, 128.1, 126.7, 122.5, 120.8, 34.3, 31.3, 30.7, 24.7, 24.3, 24.2, 23.7.

HRMS (EI+) (m/z): [M^+] calculated for $C_{48}H_{65}Br$, 720.426965; found, 720.42568.

5'-(2,6-diisopropylphenyl)-2,2'',4,4'',6,6''-hexaisopropyl-[1,1':3',1''-terphenyl]-2'-sulfonyl chloride ($A4\_SO_2Cl$)

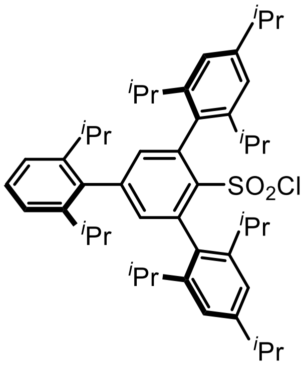

A4_Br (1 g, 1.39 mmol) was placed in a flame-dried 100 mL Schlenk flask under a nitrogen atmosphere. Dry THF (15 mL) was added and the mixture was cooled to -78 °C. *n*-BuLi (2.5 M in hexanes, 1.11 ml, 2.78 mmol, 2 equiv.) was added slowly with vigorous stirring. The reaction mixture was stirred for 0.5 h at -78 °C and then placed in an ice-bath, warmed to 0 °C and stirred at this temperature for 15 min. The resulting suspension was cooled to -94 °C and SO_2Cl_2 (0.563 g, 0.337 mL, 4.17 mmol, 3 equiv.) was added quickly in one portion. The reaction mixture was kept stirring at -94 °C for 10 min, then it was taken from the bath and allowed to warm to 0 °C. H_2O (40 mL) was added and the mixture was extracted with EtOAc (3 x 100 mL). The combined organic fractions were washed with $NaHCO_3$ (sat. aq. soln., 100 mL), brine (100 mL), dried over Na_2SO_4, filtered and concentrated under reduced pressure. The residue was purified by column chromatography (50 g of silica gel, hexanes/CH_2Cl_2 2:1) to give the title compound as a colourless solid (480 mg, 47% yield).

^{1}H NMR (400 MHz, $CDCl_3$) δ 7.31 (t, *J* = 7.9 Hz, 1H), 7.24 (s, 2H), 7.16 (d, *J* = 7.7 Hz, 2H), 7.03 (s, 4H), 2.93 (hept, *J* = 6.9 Hz, 2H), 2.74 – 2.55 (m, 6H), 1.29 (d, *J* = 6.8 Hz, 12H), 1.28 (d, *J* = 6.8 Hz, 12H), 1.11 (d, *J* = 6.8 Hz, 12H), 1.08 (d, *J* = 6.8 Hz, 12H).

^{13}C NMR (101 MHz, $CDCl_3$) δ 148.9, 146.7, 146.4, 146.0, 142.0, 141.1, 136.1, 134.4, 133.5, 128.8, 122.9, 120.8, 34.3, 31.8, 30.7, 25.9, 24.7, 24.2, 23.3.

HRMS (ESI+) (m/z): [$M+Na^+$] calculated for $C_{48}H_{65}ClO_2SNa$, 763.42860; found, 763.42627.

Sodium 5'-(2,6-diisopropylphenyl)-2,2'',4,4'',6,6''-hexaisopropyl-[1,1':3',1''-terphenyl]-2'-sulfonate (Na^+A4^-)

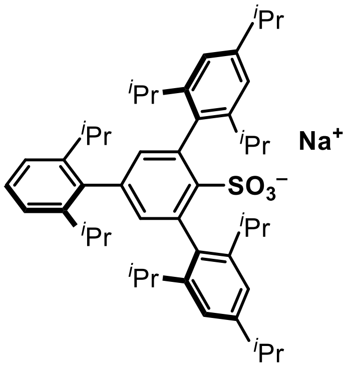

Magnesium (315 mg, 12.9 mmol, 20 equiv.) was placed in a 25 mL Schlenk flask containing a stirring bar. The flask was closed with a septum, evacuated, flame-dried under vacuum for 1 min and left to cool to room temperature. Then, the flask was refilled with nitrogen and dry THF (0.5 mL) was added followed by slow addition of 1,2-dibromoethane (0.02 mL). To activate the magnesium, the mixture was repeatedly briefly heated to reflux with a heat gun. Then, it was heated to 60 °C and a solution of **$A4\_SO_2Cl$** (480 mg, 0.647 mmol) in dry THF (5.5 mL) was added dropwise. The resulting mixture was stirred at 60 °C for an additional 2 h, before being poured into H_2O (20 mL) and extracted with EtOAc (3 x 20 mL). The combined organic extracts were washed with brine (50 mL), dried over Na_2SO_4, filtered, and concentrated under reduced pressure. The residue was dissolved in dry CH_2Cl_2 (7.5 mL) and the solution was cooled to 0 °C. Then, a solution of 3-chloroperoxybenzoic

acid (dried prior to use, approx. 80%, 154 mg, 0.712 mmol, 1.1 equiv.) in CH_2Cl_2 (7.5 mL) was added dropwise. The reaction mixture was stirred at 0 °C for 1 h, then it was diluted with CH_2Cl_2 (30 mL), washed with Na_2CO_3 (sat. aq. soln., 2 x 40 mL), $Na_2S_2O_3$ (sat. aq. soln., 40 mL), brine (40 mL), dried over Na_2SO_4, filtered, and concentrated under reduced pressure. The residue was purified by column chromatography (50 g of silica gel, CH_2Cl_2, 1 CV; then 10% *i*PrOH in CH_2Cl_2, 3 CV), the collected fractions were washed with NaOH (5% aq. soln., 2 x 50 mL), dried over Na_2SO_4, filtered, and concentrated under reduced pressure to give the title compound as a colourless solid (327 mg, 68% yield).

^{1}H NMR (500 MHz, $CDCl_3$) δ 7.25 (t, *J* = 7.8 Hz, 2H), 7.11 (d, *J* = 7.8 Hz, 2H), 6.91 (s, 2H), 6.90 (s, 4H), 2.85 (hept, *J* = 6.9 Hz, 2H), 2.68 (hept, *J* = 6.8 Hz, 2H + hept, *J* = 6.8 Hz, 4H), 1.22 (d, *J* = 6.9 Hz, 12H), 1.15 (d, *J* = 6.8 Hz, 12H), 1.06 (d, *J* = 6.9 Hz, 12H), 1.01 (d, *J* = 6.8 Hz, 12H).

^{13}C NMR (126 MHz, $CDCl_3$) δ 147.4, 146.8, 146.6, 141.7, 139.5, 138.8, 137.5, 137.1, 132.7, 128.1, 122.6, 120.1, 34.2, 31.3, 30.4, 25.5, 24.7, 24.3, 23.4.

HRMS (ESI-) (m/z): [M-Na^+] calculated for $C_{48}H_{65}O_3S$, 721.46599; found, 721.45795.

3',5'-dichloro-2,4,6-triisopropyl-1,1'-biphenyl

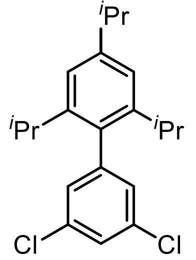

A 100 mL Schlenk flask was loaded with 3,5-dichloroiodobenzene (2 g, 7.32 mmol), 2,4,6-triisopropylphenylboronic acid (2 g, 8.05 mmol, 1.1 equiv.), $Pd(PPh_3)_2Cl_2$ (0.514 g, 0.732 mmol, 0.1 equiv.), and $Ba(OH)_2{\cdot}8H_2O$ (4.62 g, 14.6 mmol, 2 equiv.) and was placed under an inert atmosphere with three vacuum/N_2 cycles. Then, degassed 1,4-dioxane (25 mL) and degassed H_2O (10 mL) were added and the resulting suspension was stirred at 90 °C for 18 h. The reaction mixture was allowed to cool to room temperature, poured into a sat. aq. solution of NH_4Cl (100 mL), and extracted with CH_2Cl_2 (3 x 100 mL). The combined organic extracts were washed with brine (200 mL), dried over Na_2SO_4, filtered, and the solvent was removed under reduced pressure. The residue was purified by column chromatography (250 g of silica gel, hexanes) to give the title compound as a colourless solid (1.92 g, 75% yield).

^{1}H NMR (400 MHz, $CDCl_3$) δ 7.36 (t, *J* = 1.9 Hz, 1H), 7.09 (d, *J* = 1.9 Hz, 2H), 7.05 (s, 2H), 2.93 (hept, *J* = 6.9 Hz, 1H), 2.54 (hept, *J* = 6.9 Hz, 2H), 1.29 (d, *J* = 6.9 Hz, 6H), 1.10 (d, *J* = 6.9 Hz, 12H).

^{13}C NMR (101 MHz, $CDCl_3$) δ 149.0, 146.4, 144.3, 134.7, 134.4, 128.4, 127.0, 120.9, 34.5, 30.5, 24.3, 24.2.

HRMS (EI+) (m/z): [M^+] calculated for $C_{21}H_{26}Cl_2$, 348.14061; found, 348.14094.

2'-bromo-2,2'',4,4'',6,6''-hexaisopropyl-5'-(2,4,6-triisopropylphenyl)-1,1':3',1''-terphenyl (A5_Br)

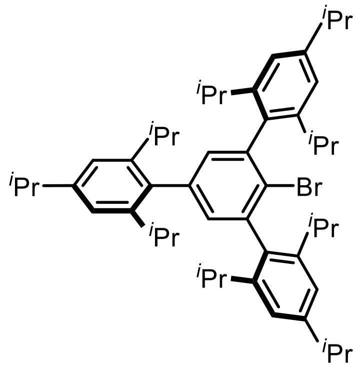

Part A. Magnesium (0.239 g, 9.83 mmol, 3.78 equiv.) was placed in a 100 mL Schlenk flask containing a stirring bar. The flask was closed with a septum, evacuated, dried with a heat-gun under vacuum for 1 min and left to cool to room temperature. Then, the flask was refilled with nitrogen and dry THF (3 mL) was added followed by slow addition of 1,2-dibromoethane (0.05 mL). To activate the magnesium, the mixture was repeatedly briefly heated to reflux with a heat gun. A solution of 1-bromo-2,4,6-triisopropylbenzene (1.62 g, 5.72 mmol, 2.2 equiv.) in dry THF (12 mL) was added dropwise while maintaining the reflux by occasionally heating the flask with a heat-gun. After that, the flask was sealed and the resulting mixture was stirred at 70 °C for an additional 24 h.

Part B. A separate 100 mL Schlenk flask was loaded with 3',5'-dichloro-2,4,6-triisopropyl-1,1'-biphenyl (0.91 g, 2.6 mmol) and was placed under an inert atmosphere with three vacuum/N_2 cycles. Dry THF (9 mL) was added, and the solution was cooled to -78 °C. *n*-BuLi (2.5M in hexanes, 1.04 mL, 2.6 mmol, 1 equiv.) was added drop-wise and the mixture was stirred at -78 °C for 1 h. Then, the Grignard reagent solution from *Part A* was quickly added through a cannula with vigorous stirring. The resulting solution was heated to 70 °C and stirred at that temperature for 3 h. Afterwards, it was cooled to -50 °C and bromine (0.499 g, 0.16 mL, 3.12 mmol, 1.2 equiv.) was added. The mixture was stirred for 10 min at -50 °C, then poured into a sat. aq. solution of Na_2SO_3 (100 mL) and extracted with CH_2Cl_2 (3 x 100 mL). The combined organic layers were washed with brine (150 mL), dried over Na_2SO_4, filtered, and the solvent was removed under reduced pressure. The residue was purified by column chromatography (200 g of silica gel, hexanes, 1 CV; then hexanes/CH_2Cl_2 8:1, 1 CV; then hexanes/CH_2Cl_2 5:1, 2 CV) to give the title compound with slight impurities (<10%) as a white solid (1.58 g, 79% yield), which was used as is in the next step.

^{1}H NMR (500 MHz, $CDCl_3$) δ 7.08 (s, 4H), 7.07 (s, 2H), 7.04 (s, 2H), 3.03 – 2.92 (hept, *J* = 6.8 Hz, 2H + hept, *J* = 6.8 Hz, 1H), 2.92 – 2.84 (hept, *J* = 6.9 Hz, 2H), 2.75 (hept, *J* = 6.9 Hz, 4H), 1.33 (d, *J* = 6.9 Hz, 12H), 1.30 (d, *J* = 6.9 Hz, 6H), 1.26 (d, *J* = 6.8 Hz, 12H), 1.16 – 1.11 (m, 24H, two overlapped doublets).

^{13}C NMR (126 MHz, $CDCl_3$) δ 148.4, 148.2, 146.4, 146.0, 142.1, 139.6, 136.5, 135.7, 131.5, 126.5, 120.7, 120.5, 34.5, 34.3, 31.2, 30.7, 24.7, 24.3, 24.23, 24.21, 23.7.

HRMS (APCI+) (m/z): [M+H$^+$] calculated for $C_{51}H_{72}Br$, 763.48119; found, 763.48075.

2,2'',4,4'',6,6''-hexaisopropyl-5'-(2,4,6-triisopropylphenyl)-[1,1':3',1''-terphenyl]-2'-sulfonyl chloride ($A5\_SO_2Cl$)

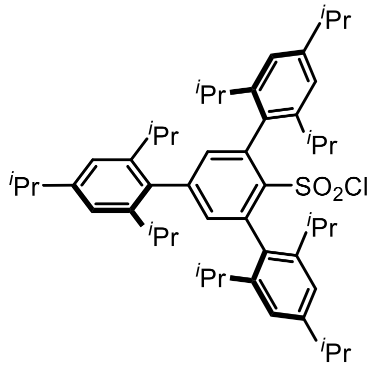

A5_Br (1.92 g, 2.51 mmol) was placed in a flame-dried 100 mL Schlenk flask under a nitrogen atmosphere. Dry THF (20 mL) was added, and the mixture was cooled to -78 °C. *n*-BuLi (2.5 M in hexanes, 2.01 ml, 5.02 mmol, 2 equiv.) was added slowly with vigorous stirring. The reaction mixture was stirred for 0.5 h at -78 °C and then placed in an ice-bath, warmed to 0 °C and stirred at this temperature for 15 min. The resulting suspension was cooled to -94 °C in an acetone/liquid N_2 bath and SO_2Cl_2 (1.02 g, 0.61 mL, 7.53 mmol, 3 equiv.) was added quickly in one portion. The reaction mixture was kept stirring at -94 °C for 10 min, then it was taken from the bath and allowed to warm to 0 °C. H_2O (100 mL) was added and the mixture was extracted with EtOAc (3 x 100 mL). The combined organic fractions were washed with $NaHCO_3$ (sat. aq. soln., 100 mL), brine (100 mL), dried over Na_2SO_4, filtered, and concentrated under reduced pressure. The residue was purified by column chromatography (200 g of silica gel, hexanes/CH_2Cl_2 2:1), the fractions containing impurity were concentrated and purified again by column chromatography (200 g of silica gel, hexanes/CH_2Cl_2 3:1), to provide with the title compound (1.1 g, 56%) as a yellowish solid.

^{1}H NMR (500 MHz, $CDCl_3$) δ 7.23 (s, 2H), 7.02 (s, 4H), 7.00 (s, 2H), 2.97 – 2.83 (hept, *J* = 6.9 Hz, 2H + hept, *J* = 6.9 Hz, 1H), 2.71 – 2.56 (hept, *J* = 6.8 Hz, 2H + hept, *J* = 6.8 Hz, 4H), 1.31 – 1.26 (m, 24H, two overlapped doublets), 1.24 (d, *J* = 6.9 Hz, 6H), 1.10 (d, *J* = 6.8 Hz, 12H), 1.08 (d, *J* = 6.8 Hz, 12H).

^{13}C NMR (126 MHz, $CDCl_3$) δ 149.1, 148.8, 147.1, 146.2, 146.0, 141.9, 140.9, 134.7, 133.7, 133.6, 120.8, 120.7, 34.4, 34.3, 31.8, 30.7, 25.9, 24.7, 24.2, 24.1, 23.3.

HRMS (ESI+) (m/z): [$M+NH_4^+$] calculated for $C_{51}H_{75}O_2NClS$, 800.52016; found, 800.52022.

Sodium 2,2'',4,4'',6,6''-hexaisopropyl-5'-(2,4,6-triisopropylphenyl)-[1,1':3',1''-terphenyl]-2'-sulfonate (Na^+A5^-)

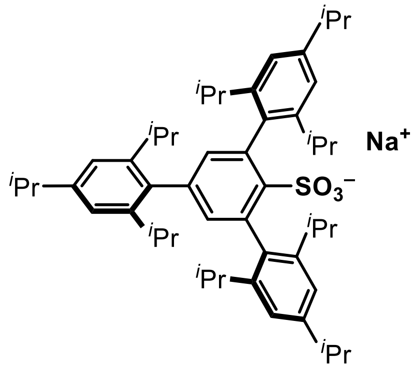

Magnesium (143 mg, 5.88 mmol, 20 equiv.) was placed in a 10 mL Schlenk flask containing a stirring bar. The flask was closed with a septum, evacuated, flame-dried under vacuum for 1 min and left to cool down to room temperature. Then, the flask was refilled with nitrogen and dry THF (0.5 mL) was added followed by slow addition of 1,2-dibromoethane (0.02 mL). To activate the magnesium, the mixture was repeatedly briefly

heated to reflux with a heat gun. Then, it was heated to 60 °C and a solution of **A5_SO$_2$Cl** (230 mg, 0.294 mmol) in dry THF (2 mL) was added dropwise. The resulting mixture was stirred at 60 °C for an additional 2 h, before being poured into H_2O (10 mL) and extracted with EtOAc (3 x 10 mL). The combined organic extracts were washed with brine (20 mL), dried over Na_2SO_4, filtered and concentrated under reduced pressure. The residue was dissolved in dry CH_2Cl_2 (3 mL) and the solution was cooled to 0 °C. Then, a solution of 3-chloroperoxybenzoic acid (dried prior to use, approx. 80%, 69.8 mg, 0.323 mmol, 1.1 equiv.) in CH_2Cl_2 (1 mL) was added dropwise. The reaction mixture was stirred at 0 °C for 1 h, then it was diluted with CH_2Cl_2 (30 mL), washed with NaOH (5%. aq. soln., 20 mL), $Na_2S_2O_3$ (sat. aq. soln., 20 mL), brine (20 mL), dried over Na_2SO_4, filtered, and concentrated under reduced pressure. The residue was purified by column chromatography (25 g of silica gel, CH_2Cl_2, 1 CV, then 10% *i*PrOH in CH_2Cl_2, 2 CV), the collected fractions were washed with NaOH (5% aq. soln., 2 x 50 mL), dried over Na_2SO_4, filtered, and concentrated under reduced pressure to give the title compound as an off-white solid (140 mg, 61% yield).

^{1}H NMR (500 MHz, $CDCl_3$) δ 6.95 (s, 2H), 6.92 (s, 6H), 2.86 (hept, *J* = 6.9 Hz, 2H + hept, *J* = 6.9 Hz, 1H), 2.75 – 2.65 (hept, *J* = 6.8 Hz, 2H + hept, *J* = 6.8 Hz, 4H), 1.26 – 1.21 (two overlapped doublets, 18H), 1.17 (d, *J* = 6.8 Hz, 12H), 1.05 (d, *J* = 6.8 Hz, 12H), 0.99 (d, *J* = 6.9 Hz, 12H).

^{13}C NMR (126 MHz, $CDCl_3$) δ 148.2, 147.4, 147.1, 146.5, 141.6, 139.6, 139.2, 137.6, 135.2, 132.6, 120.5, 119.8, 34.4, 34.2, 31.3, 30.4, 25.5, 24.7, 24.3, 24.2, 23.3.

HRMS (ESI-) (m/z): [M-Na$^+$] calculated for $C_{51}H_{71}O_3S$, 763.51294; found, 763.51264.

2''-bromo-5''-(2,6-diisopropylphenyl)-2''',3',5',6'''-tetraisopropyl-2,2'''',4,4'''',6,6''''-hexamethyl-1,1':4',1'':3'',1''':4''',1''''-quinquephenyl (A6_Br)

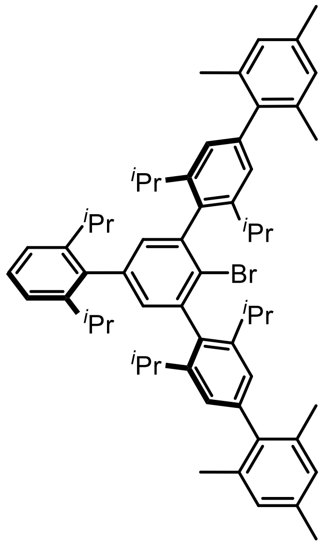

Part A. Magnesium (108 mg, 4.5 mmol, 3.1 equiv.) was placed in a 10 mL Schlenk flask containing a stirring bar. The flask was closed with a septum, evacuated, dried with a heat-gun under vacuum for 1 min and left to cool to room temperature. Then, the flask was refilled with nitrogen and dry THF (0.3 mL) was added followed by slow addition of 1,2-dibromoethane (0.02 mL). To activate the magnesium, the mixture was repeatedly briefly heated to reflux with a heat gun. A solution of **R^b-Br** (1.29 g, 3.6 mmol, 2.5 equiv.) in dry THF (3 mL) was added dropwise while maintaining the reflux by heating the flask with a heat-gun. After that, the flask was sealed and the resulting mixture was stirred at 60 °C for an additional 5 h.

Part B. A 25 mL Schlenk flask was loaded with 3',5'-dichloro-2,6-diisopropyl-1,1'-biphenyl (442 mg, 1.44 mmol, prepared as described above) and was placed under an inert atmosphere with three vacuum/N_2 cycles. Dry

THF (4 mL) was added, and the solution was cooled to -78 °C. *n*-BuLi (2.5M in hexanes, 0.58 mL, 1.44 mmol, 1 equiv.) was added dropwise and the mixture was stirred at -78 °C for 1.5 h. Then, the Grignard reagent solution from *Part A* was added quickly with a syringe with vigorous stirring. The resulting solution was heated to 66 °C and stirred at that temperature for 3 h. After that, it was cooled to -30 °C and bromine (0.118 mL, 368 mg, 2.3 mmol, 1.6 equiv.) was added. The mixture was stirred for 5 min at -30 °C before being warmed to 0 °C. Na_2SO_3 (sat. aq. soln., 8 mL) was added with vigorous stirring, the mixture was separated between H_2O (10 mL) and EtOAc (50 mL), the aqueous phase was extracted with EtOAc (2 x 30 mL), the combined organic layers were dried over $MgSO_4$, filtered, and the solvent was removed under reduced pressure. The residue was subjected to column chromatography (100 g of silica gel, dry-loaded with 2 g of silica gel, hexanes/CH_2Cl_2 98:2, 2.5 CV; then 96:4, 2.5 CV; then 94:6, 2.5 CV). The obtained material (773 mg) was contaminated with a side product in which there is H instead of Br (product/side product 0.78:0.22).

^{1}H NMR (400 MHz, $CDCl_3$) δ 7.33 (t, *J* = 7.7 Hz, 1H^{product} + 1H$^{side\ product}$), 7.21 – 7.20 (m, 3H$^{side\ product}$, overlapped), 7.20 (d, *J* = 7.8 Hz, 2H^{product}), 7.17 (s, 2H^{product}), 7.12 (d, *J* = 1.6 Hz, 2H$^{side\ product}$), 6.98 – 6.97 (m, 8H^{product} + 4H$^{side\ product}$), 6.97 (s, 4H$^{side\ product}$), 2.98 – 2.86 (m, 2H^{product} + 6H$^{side\ product}$), 2.77 (hept, *J* = 6.9 Hz, 4H^{product}), 2.35 (s, 6H^{product} + 6H$^{side\ product}$), 2.08 (s, 6H^{product}), 2.06 (s, 6H^{product} + 12H$^{side\ product}$), 1.22 (d, *J* = 6.9 Hz, 12H^{product}), 1.16 (d, J = 6.9 Hz, 12H^{product} + 12H$^{side\ product}$), 1.14 – 1.10 (m, 12H^{product} + 24H$^{side\ product}$).

^{13}C NMR (101 MHz, $CDCl_3$) δ 146.83, 146.76, 146.7, 146.3, 142.2, 140.59, 140.55, 140.3, 140.1, 139.5, 139.4, 138.1, 137.4, 136.9, 136.6, 136.43, 136.37, 136.3, 136.1, 131.1, 129.5, 129.2, 128.23, 128.20, 128.16, 127.9, 126.6, 123.8, 123.5, 122.6, 122.5, 31.3, 30.8, 24.7, 24.3, 24.2, 23.6, 21.2, 21.99, 21.95, 21.81, 20.8. Includes signals from the product and the side product.

HRMS (APCI+) (m/z): [M+H$^+$]^{product} calculated for $C_{60}H_{74}Br$, 873.49684; found, 873.49649.

HRMS (APCI+) (m/z): [M+H$^+$]$^{side\ product}$ calculated for $C_{60}H_{75}$, 795.58633; found, 795.58592.

5''-(2,6-diisopropylphenyl)-2''',3',5',6'''-tetraisopropyl-2,2'''',4,4'''',6,6''''-hexamethyl-[1,1':4',1'':3'',1''':4''',1''''-quinquephenyl]-2''-sulfonyl chloride (A6_SO$_2$Cl)

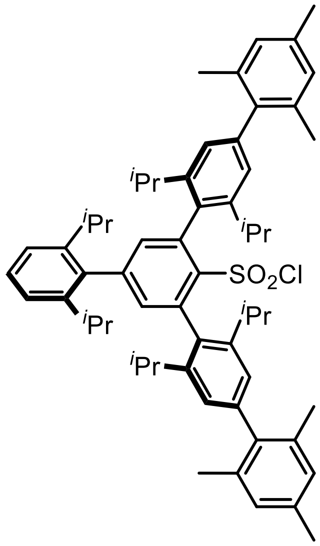

A6_Br (730 mg, contaminated with a side product as described above) was placed in a flame-dried Schlenk flask under a nitrogen atmosphere. Dry THF (8 mL) was added and the mixture was cooled to -78 °C. *n*-BuLi (2.5 M in hexanes, 0.84 ml, 2.09 mmol, 2.5 equiv.) was added slowly with vigorous stirring. The reaction mixture was stirred for 20 min at -78 °C and then placed in an ice-bath, warmed to 0 °C and stirred at this temperature for 15 min. The resulting suspension was cooled to -94 °C and SO_2Cl_2 (339 mg, 0.204 mL, 2.51 mmol, 3 equiv.) was added quickly in one portion. The reaction mixture was kept stirring at -94 °C for 20 min, then it was taken from the bath and warmed to 0 °C. H_2O (15 mL) was added and the mixture was extracted with EtOAc (3 x 20 mL). The combined organic fractions were washed with $NaHCO_3$ (sat. aq. soln., 10 mL), dried over $MgSO_4$, filtered, and concentrated under reduced pressure. The residue was purified by column chromatography (30 g of silica gel, dry-loaded with 2 g of silica gel, hexanes/CH_2Cl_2 90:10, 2 CV; then 90:10, 2 CV; then 85:15, 2 CV; then 80:20, 2 CV) to give the title compound as a colourless solid (193 mg, 15% total yield over two steps, based on 3',5'-dichloro-2,6-diisopropyl-1,1'-biphenyl).

^{1}H NMR (400 MHz, $CDCl_3$) δ 7.38 – 7.33 (m, 3H), 7.21 (d, *J* = 7.7 Hz, 2H), 6.99 (s, 6H), 6.97 (s, 2H), 2.80 – 2.61 (m, 6H), 2.35 (s, 6H), 2.07 (s, 12H), 1.28 (d, *J* = 6.7 Hz, 12H), 1.16 (d, *J* = 6.8 Hz, 12H), 1.10 (d, *J* = 6.8 Hz, 12H).

^{13}C NMR (101 MHz, $CDCl_3$) δ 146.7, 146.5, 141.8, 141.6, 141.1, 139.8, 136.8, 136.7, 136.3, 135.9, 134.4, 134.0, 129.0, 128.3, 128.1, 123.8, 123.0, 31.9, 30.8, 25.9, 24.7, 23.2, 21.2, 20.94, 20.88.

HRMS (ESI+) (m/z): [M+Na$^+$] calculated for $C_{60}H_{73}SO_2ClNa$, 915.49120; found, 915.48969.

Sodium 5''-(2,6-diisopropylphenyl)-2''',3',5',6'''-tetraisopropyl-2,2'''',4,4'''',6,6''''-hexamethyl-[1,1':4',1'':3'',1''':4''',1''''-quinquephenyl]-2''-sulfonate (Na^+A6^-)

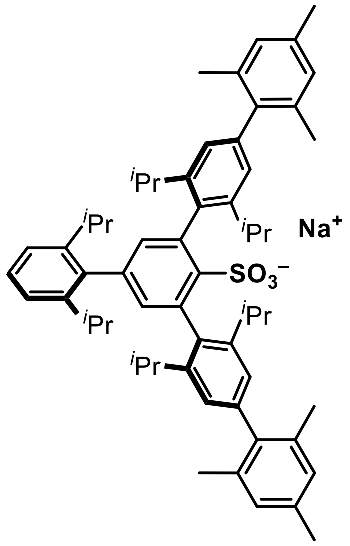

Magnesium (29.2 mg, 1.2 mmol, 6.3 equiv.) was placed in a 10 mL Schlenk flask containing a stirring bar. The flask was closed with a septum, evacuated, flame-dried under vacuum for 1 min and left to cool to room temperature. Then, the flask was refilled with nitrogen and dry THF (0.3 mL) was added followed by slow addition of 1,2-dibromoethane (0.02 mL). To activate the magnesium, the mixture was repeatedly briefly heated to reflux with a heat gun. Then, it was heated to 60 °C and a solution of **A6_SO_2Cl** (170 mg, 0.19 mmol) in dry THF (3 mL) was added dropwise. The resulting mixture was stirred at 60 °C for an additional 1 h, then H_2O (2 mL) was added, and the mixture was separated between H_2O (10 mL) and EtOAc (20 mL). The aqueous phase was extracted with EtOAc (2 x 10 mL), and the combined organic extracts were washed with brine (5 mL), dried over $MgSO_4$, filtered, and concentrated under reduced pressure. The residue was dissolved in dry CH_2Cl_2 (5 mL) and the solution was cooled to 0 °C. Then, a solution of 3-chloroperoxybenzoic acid (77%, 46.8 mg, 0.21 mmol, 1.1 equiv.) in CH_2Cl_2 (5 mL) was added dropwise. The reaction mixture was stirred at 0 °C for 1 h, then it was diluted with hexanes (50 mL), washed with NaOH (1M aq. soln., 3 x 10 mL), dried over $MgSO_4$, filtered, and concentrated under reduced pressure. The residue was purified by column chromatography (10 g of silica gel, 5% *i*PrOH in CH_2Cl_2, 5 CV; then 10% *i*PrOH in CH_2Cl_2, 1 CV), the collected fractions were washed with NaOH (5% aq. soln., 2 x 50 mL), filtered through a filter paper, and concentrated under reduced pressure to give the title compound as a colourless solid (154 mg, 90% yield).

^{1}H NMR (400 MHz, $CDCl_3$) δ 7.31 (t, *J* = 7.7 Hz, 1H), 7.17 (d, *J* = 7.8 Hz, 2H), 7.02 (s, 2H), 6.97 (s, 2H), 6.85 (s, 4H), 6.73 (s, 2H), 2.87 – 2.65 (m, 6H), 2.33 (s, 6H), 1.99 (s, 6H), 1.93 (s, 6H), 1.12 (d, *J* = 6.8 Hz, 12H), 1.08 (d, *J* = 6.7 Hz, 12H), 0.99 (d, *J* = 6.8 Hz, 12H).

^{13}C NMR (101 MHz, $CDCl_3$) δ 146.9, 146.8, 141.4, 139.9, 139.7, 139.6, 139.5, 137.8, 137.7, 136.4, 136.3, 135.8, 132.5, 128.3, 128.3, 123.3, 122.8, 31.3, 30.5, 25.5, 24.7, 23.3, 21.1, 21.0, 20.9.

HRMS (ESI-) (m/z): [$(M-Na^+)^-$] calculated for $C_{60}H_{73}SO_3$, 873.52749; found, 873.52618.

2'-bromo-5'-(2,6-diisopropylphenyl)-2,2'',6,6''-tetraisopropyl-4,4''-bis(tris(4-(*tert*-butyl)phenyl)methyl)-1,1':3',1''-terphenyl (A7_Br)

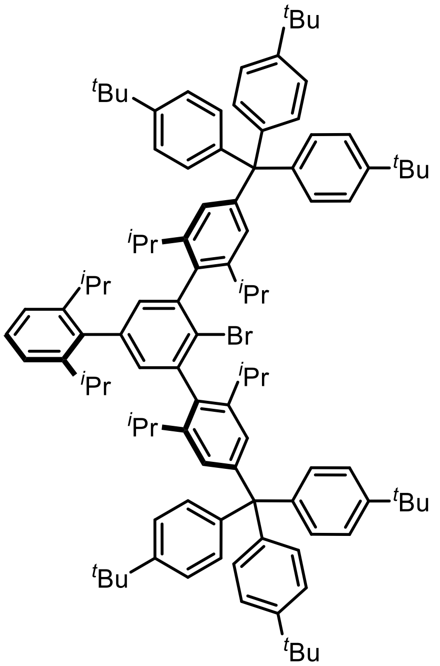

Part A. Magnesium (64 mg, 2.67 mmol, 3.7 equiv.) was placed in a 10 mL Schlenk flask containing a stirring bar. The flask was closed with a septum, evacuated, dried with a heat gun under vacuum for 1 min and left to cool down to room temperature. Then, the flask was refilled with nitrogen and dry THF (0.3 mL) was added followed by slow addition of 1,2-dibromoethane (0.02 mL). To activate the magnesium, the mixture was repeatedly briefly heated to reflux with a heat gun. A solution of **R^c-Br** (1.17 g, 1.8 mmol, 2.5 equiv.) in dry THF (5.5 mL) was added dropwise while maintaining the reflux by occasionally heating the flask with a heat gun. After that, the flask was sealed and the resulting mixture was stirred at 60 °C for an additional 23 h.

Part B. A 25 mL Schlenk flask was loaded with 3',5'-dichloro-2,6-diisopropyl-1,1'-biphenyl (224 mg, 0.72 mmol, prepared as described above) and was placed under an inert atmosphere with three vacuum/N_2 cycles. Dry THF (2 mL) was added and the solution was cooled to -78 °C. *n*-BuLi (2.5M in hexanes, 0.288 mL, 0.72 mmol, 1 equiv.) was added dropwise and the mixture was stirred at -78 °C for 1.5 h. Then, the Grignard reagent solution from *Part A* was added quickly with a syringe with vigorous stirring. The resulting solution was heated to 66 °C and stirred at that temperature for 3 h. After that, it was cooled to -30 °C and bromine (0.05 mL, 184 mg, 1.15 mmol, 1.6 equiv.) was added. The mixture was stirred for 5 min before being warmed to 0 °C. Na_2SO_3 (sat. aq. soln., 4 mL) was added with vigorous stirring, the mixture was separated between H_2O (10 mL) and EtOAc (30 mL) and the aqueous phase was extracted with EtOAc (2 x 30 mL). The combined organic layers were dried over $MgSO_4$, filtered, and the solvent was removed under reduced pressure. The residue was subjected to column chromatography (100 g of silica gel, hexanes/CH_2Cl_2 98:2, 5 CV; then 97:3, 2.5 CV; then 94:6, 2.5 CV; then 94:6, 2.5 CV; then 92:8, 2.5 CV), to afford the title compound as a colourless solid (761 mg, 73%).

^{1}H NMR (400 MHz, $CDCl_3$) δ 7.30 (t, *J* = 7.7 Hz, 1H), 7.27 – 7.23 (m, 14H), 7.17 (d, *J* = 7.7 Hz, 2H), 7.13 (m, 12H), 7.06 (s, 2H), 6.89 (s, 4H), 2.83 (hept, *J* = 6.6 Hz, 2H), 2.62 (hept, *J* = 6.9 Hz, 4H), 1.31 (s, 54H), 1.10 (d, *J* = 6.9 Hz, 12H), 1.01 (d, *J* = 6.8 Hz, 12H), 0.88 (d, *J* = 6.8 Hz, 12H).

^{13}C NMR (101 MHz, $CDCl_3$) δ 148.5, 146.8, 146.7, 144.5, 144.3, 142.2, 139.2, 138.2, 136.0, 131.1, 131.0, 128.1, 126.5, 126.3, 124.0, 122.5, 64.2, 34.5, 31.5, 31.1, 30.7, 24.4, 24.2, 23.4.

HRMS (APCI+) (m/z): [$M+NH_4^+$] calculated for $C_{104}H_{133}NBr$, 1474.96159; found, 1474.96126.

5'-(2,6-diisopropylphenyl)-2,2'',6,6''-tetraisopropyl-4,4''-bis(tris(4-(*tert*-butyl)phenyl)methyl)-[1,1':3',1''-terphenyl]-2'-sulfonyl chloride (A7_SO_2Cl)

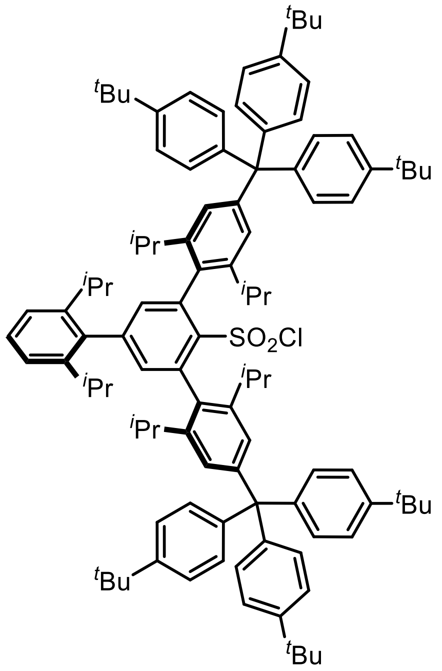

A7_Br (700 mg, 0.48 mmol) was placed in a flame-dried Schlenk flask under a nitrogen atmosphere. Dry THF (10 mL) was added and the mixture was cooled to -78 °C. *n*-BuLi (2.5 M in hexanes, 0.48 ml, 1.2 mmol, 2.5 equiv.) was added slowly with vigorous stirring. The reaction mixture was stirred for 20 min at -78 °C and then placed in an ice-bath, warmed to 0 °C, and stirred at this temperature for 15 min. The resulting suspension was cooled to -94 °C and SO_2Cl_2 (194 mg, 0.117 mL, 1.44 mmol, 3 equiv.) was quickly added in one portion. The reaction mixture was kept stirring at -94 °C for 20 min, then it was taken from the bath and warmed to 0 °C. H_2O (15 mL) was added and the mixture was extracted with EtOAc (3 x 20 mL). The combined organic fractions were washed with $NaHCO_3$ (sat. aq. soln., 10 mL), dried over $MgSO_4$, filtered, and concentrated under reduced pressure. The residue was purified by column chromatography (30 g of silica gel, dry-loaded with 2 g of silica gel, hexanes/CH_2Cl_2 95:5, 2 CV; then 90:10, 2 CV; then 85:15, 1.5 CV; then 80:20, 1.5 CV) to give the title compound as a colourless solid (338 mg, 48%).

^{1}H NMR (400 MHz, $CDCl_3$) δ 7.33 (t, *J* = 7.8 Hz, 1H), 7.28 – 7.22 (m, 14H), 7.18 (d, *J* = 7.8 Hz, 2H), 7.15 – 7.10 (m, 12H), 6.88 (s, 4H), 2.66 (hept, *J* = 6.9 Hz, 2H), 2.54 (hept, *J* = 7.0 Hz, 4H), 1.31 (s, 54H), 1.10 (d, *J* = 6.8 Hz, 12H), 1.06 (d, *J* = 6.7 Hz, 12H), 0.87 (d, *J* = 6.8 Hz, 12H).

^{13}C NMR (101 MHz, $CDCl_3$) δ 148.5, 147.4, 146.5, 146.4, 144.7, 144.2, 141.9, 141.6, 136.4, 134.2, 133.2, 131.1, 128.9, 126.2, 124.0, 123.0, 64.1, 34.5, 31.7, 31.5, 30.7, 25.6, 24.6, 22.9.

HRMS (ESI+) (m/z): [M+Na^+] calculated for $C_{104}H_{129}SO_2ClNa$, 1499.92940; found, 1499.92505.

Sodium 5'-(2,6-diisopropylphenyl)-2,2'',6,6''-tetraisopropyl-4,4''-bis(tris(4-(tert-butyl)phenyl)methyl)-[1,1':3',1''-terphenyl]-2'-sulfonate (Na+A7−)

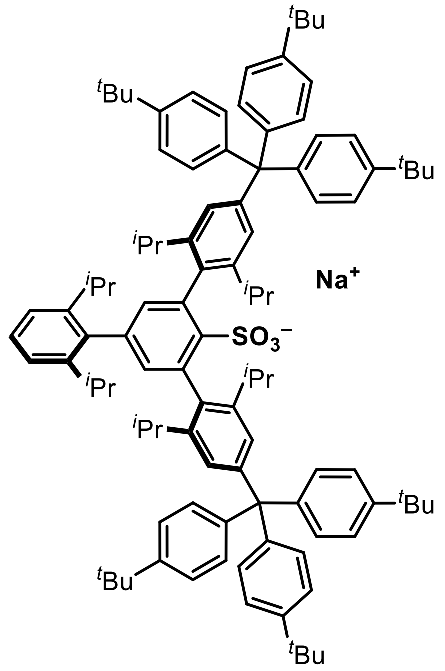

Magnesium (29.6 mg, 1.23 mmol, 6.2 equiv.) was placed in a 10 mL Schlenk flask containing a stirring bar. The flask was closed with a septum, evacuated, flame-dried under vacuum for 1 min and left to cool down to room temperature. Then, the flask was refilled with nitrogen and dry THF (0.3 mL) was added followed by slow addition of 1,2-dibromoethane (0.02 mL). To activate the magnesium, the mixture was repeatedly briefly heated to reflux with a heat gun. Then, it was heated to 60 °C and a solution of **A7_SO$_2$Cl** (291 mg, 0.20 mmol) in dry THF (3 mL) was added dropwise. The resulting mixture was stirred at 60 °C for an additional 1 h. Then, H_2O (2 mL) was added, the mixture was separated between H_2O (10 mL) and EtOAc (20 mL), and the aqueous phase was extracted with EtOAc (2 x 10 mL). The combined organic extracts were washed with brine (5 mL), dried over $MgSO_4$, filtered, and concentrated under reduced pressure. The residue was dissolved in dry CH_2Cl_2 (5 mL) and the solution was cooled to 0 °C. Then, a solution of 3-chloroperoxybenzoic acid (77%, 48.6 mg, 0.22 mmol, 1.1 equiv.) in CH_2Cl_2 (5 mL) was added dropwise. The reaction mixture was stirred at 0 °C for 1 h, then it was diluted with hexanes (50 mL), washed with NaOH (1M aq. soln., 3 x 10 mL), dried over $MgSO_4$, filtered, and concentrated under reduced pressure. The residue was purified by column chromatography (10 g of silica gel, CH_2Cl_2, 5 CV; then 5% *i*PrOH in CH_2Cl_2, 4 CV), the collected fractions were washed with NaOH (5% aq. soln., 2 x 50 mL), filtered through a filter paper, and concentrated under reduced pressure to give the title compound as a colourless solid (272 mg, 93% yield).

^{1}H NMR (400 MHz, $CDCl_3$) δ 7.32 (t, *J* = 7.7 Hz, 1H), 7.19 – 7.12 (m, 26H), 6.93 (s, 4H), 6.89 (s, 2H), 2.75 (hept, *J* = 6.9 Hz, 2H), 2.67 (hept, *J* = 6.7 Hz, 4H), 1.24 (s, 54H), 1.08 (d, *J* = 6.8 Hz, 12H), 0.94 (d, *J* = 6.7 Hz, 12H), 0.81 (d, *J* = 6.8 Hz, 12H).

^{13}C NMR (101 MHz, $CDCl_3$) δ 148.2, 147.0, 146.4, 145.6, 144.7, 140.7, 140.5, 139.6, 138.0, 137.4, 132.3, 130.7, 128.2, 125.2, 124.3, 122.8, 64.2, 34.4, 31.5, 31.2, 30.4, 25.5, 24.7, 23.1.

HRMS (ESI-) (m/z): [(M-Na$^+$)$^-$] calculated for $C_{104}H_{129}SO_3$, 1457.96570; found, 1457.96497.

2.6 Synthesis of cations present in 7-10

4-(2,6-diisopropyl-4-(4,4,5,5-tetramethyl-1,3,2-dioxaborolan-2-yl)phenyl)-2,6-bis(2,4,6-triisopropylphenyl)pyridine (Bpin_C4_N)

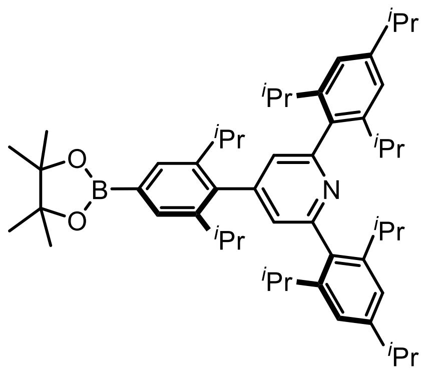

A heat-gun dried 100 mL Schlenk flask was loaded with 4,4'-di-tert-butyl-2,2'-bipyridyl (50 mg, 0.187 mmol, 0.06 equiv.), (1,5-cyclooctadiene)(methoxy)iridium(I) dimer (62 mg, 0.093 mmol, 0.03 equiv.), bis(pinacolato)diboron (0.948 g, 3.73 mmol, 1.2 equiv.), and **C4_N** (2 g, 3.11 mmol), and was placed under an inert atmosphere with three vacuum/N_2 cycles. THF (20 mL) was added, the flask was sealed and the resulting mixture was stirred at 80 °C for 12 h. Then, the mixture was allowed to cool to room temperature, hexane (20 mL) was added, and the solvent was removed under reduced pressure. The crude material was purified by column chromatography (150 g of silica gel, hexanes/CH_2Cl_2 1:1) to give the title compound as a colourless solid (1.98 g, 83% yield).

^{1}H NMR (500 MHz, $CDCl_3$) δ 7.67 (s, 2H), 7.11 (s, 2H), 7.03 (s, 4H), 2.91 (hept, *J* = 6.8 Hz, 2H), 2.82 – 2.69 (hept, *J* = 6.8 Hz, 2H + hept, *J* = 6.9 Hz, 4H), 1.37 (s, 12H), 1.25 (d, *J* = 6.9 Hz, 12H), 1.20 – 1.14 (d, *J* = 6.9 Hz, 12H + d, *J* = 6.9 Hz, 12H), 1.11 (d, *J* = 6.9 Hz, 12H).

^{13}C NMR (126 MHz, $CDCl_3$) δ 160.0, 148.8, 148.7, 146.1, 145.3, 140.1, 136.7, 129.3, 124.1, 120.6, 83.9, 34.6, 30.9, 30.8, 25.1, 24.7, 24.3, 24.1, 23.6.

HRMS (ESI+) (m/z): [M+H$^+$] calculated for $C_{53}H_{77}O_2NB$, 770.60528; found, 770.60455.

4,4'-(3,3'',5,5''-tetraisopropyl-[1,1':4',1''-terphenyl]-4,4''-diyl)bis(2,6-bis(2,4,6-triisopropylphenyl)pyridine) (C8_N)

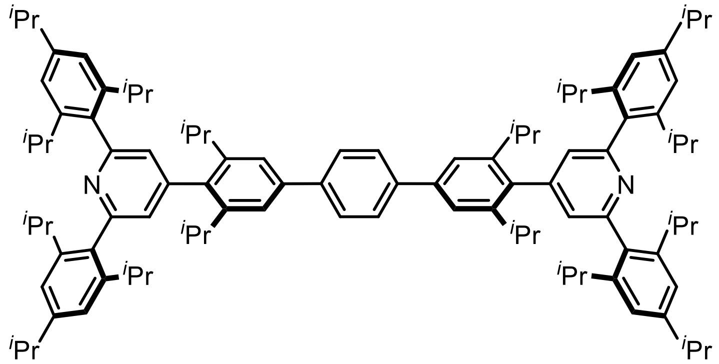

A 25 mL Schlenk flask was loaded with **Bpin_C4_N** (549 mg, 0.713 mmol, 2.4 equiv.), 1,4-dibromobenzene (70 mg, 0.297 mmol), $Pd(PPh_3)_4$ (68.6 mg, 0.059 mmol, 0.2 equiv.), and K_2CO_3 (197 mg, 1.43 mmol, 4.8 equiv.) and was placed under an inert atmosphere with three vacuum/N_2 cycles. Then, degassed toluene (3 mL), degassed 1,4-dioxane (3 mL), and degassed H_2O (3 mL) were added and the resulting suspension was stirred at 100 °C for 18 h. The reaction mixture was allowed to cool to room temperature and NH_4Cl (sat.

aq. soln. 15 mL) and CH_2Cl_2 (15 mL) were added. The layers were separated and the aqueous layer was extracted with CH_2Cl_2 (3 x 15 mL). The combined organic extracts were washed with brine (30 mL), dried over Na_2SO_4, filtered, and the solvent was removed under reduced pressure. The residue was purified by column chromatography (125 g of silica gel, hexanes/CH_2Cl_2 1:1, 2 CV; hexanes/CH_2Cl_2 1:2, 1 CV) to give the title compound as a colourless solid (230 mg, 57% yield).

^{1}H NMR (500 MHz, $CDCl_3$) δ 7.74 (s, 4H), 7.47 (s, 4H), 7.20 (s, 4H), 7.04 (s, 8H), 2.95 – 2.82 (hept, J = 6.9 Hz, 4H + hept, J = 6.9 Hz, 4H), 2.80 – 2.69 (m, 8H), 1.26 (d, *J* = 6.9 Hz, 24H), 1.24 – 1.16 (m, 48H, two overlapped doublets), 1.13 (d, *J* = 6.9 Hz, 24H).

^{13}C NMR (126 MHz, $CDCl_3$) δ 160.0, 148.9, 148.6, 146.7, 146.1, 141.1, 140.8, 136.5, 136.3, 127.7, 124.6, 121.8, 120.6, 34.6, 31.0, 30.9, 24.7, 24.3, 24.2, 23.6.

HRMS (ESI+) (m/z): [M+2H$^+$] calculated for $C_{100}H_{134}N_2$, 681.5268; found, 681.52735.

4,4'-(3,3'',5,5''-tetraisopropyl-[1,1':4',1''-terphenyl]-4,4''-diyl)bis(1-methyl-2,6-bis(2,4,6-triisopropylphenyl)pyridin-1-ium) tetrafluoroborate (C8^{2+}(BF_4^-)$_2$)

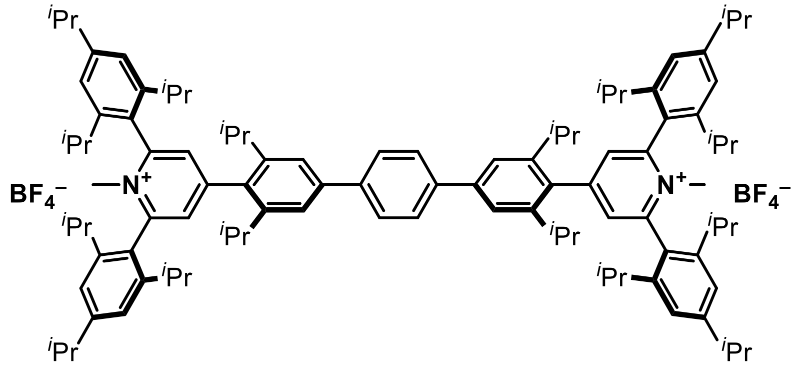

A 10 mL Schlenk flask was loaded with **C8_N** (201 mg, 0.148 mmol) and placed under an inert atmosphere with three vacuum/N_2 cycles. Dry CH_2Cl_2 (2.5 mL) was added and the mixture was cooled to 0 °C. Trimethyloxonium tetrafluoroborate (219 mg, 1.48 mmol, 10 equiv.) was added in one portion and the resulting suspension was stirred at 0 °C for 2.5 h. Then, *N,N*-diisopropylethylamine (191 mg, 0.258 mL, 1.48 mmol, 10 equiv.) was added, followed by the addition of trimethyloxonium tetrafluoroborate (219 mg, 1.48 mmol, 10 equiv.), and the mixture was warmed to room temperature, and stirred for another 30 min. *N,N*-Diisopropylethylamine (191 mg, 0.258 mL, 1.48 mmol, 10 equiv.) was added again, followed by the addition of trimethyloxonium tetrafluoroborate (219 mg, 1.48 mmol, 10 equiv.), and the reaction mixture was stirred for 30 min. After that, it was separated between CH_2Cl_2 (20 mL) and H_2O (20 mL), and the aqueous layer was extracted with CH_2Cl_2 (2 x 20 mL). The combined organic extracts were dried over Na_2SO_4, filtered, and the solvent was removed under reduced pressure. The residue was purified by column chromatography (40 g of silica gel, CH_2Cl_2, 1 CV; then 10% *i*PrOH in CH_2Cl_2, 2 CV), the collected fractions were washed with $NaBF_4$ (10% aq. soln., 50 mL), filtered through a filter paper and the solvent was removed under reduced pressure. The title compound was obtained as a yellowish solid (180 mg, 78% yield).

^1H NMR (500 MHz, $CDCl_3$) δ 7.83 (s, 4H), 7.71 (s, 4H), 7.51 (s, 4H), 7.24 (s, 8H), 3.77 (s, 6H), 3.00 (hept, *J* = 6.9 Hz, 4H), 2.56 (hept, *J* = 6.8 Hz, 4H), 2.22 (hept, *J* = 6.9 Hz, 8H), 1.33 (d, *J* = 6.8 Hz, 24H), 1.31 (d, *J* = 6.9 Hz, 24H), 1.25 (d, *J* = 6.8 Hz, 24H), 1.22 (d, *J* = 6.8 Hz, 24H).

^{13}C NMR (126 MHz, $CDCl_3$) δ 159.5, 157.7, 154.2, 145.8, 145.7, 143.4, 140.3, 131.3, 131.2, 128.0, 125.5, 123.1, 122.6, 64.6, 43.8, 34.7, 32.9, 31.9, 25.5, 24.9, 24.2, 23.9, 23.5.

^{19}F NMR (471 MHz, $CDCl_3$) δ -154.36, -154.41.

HRMS (ESI+) (m/z): [M^{2+}] calculated for $C_{102}H_{138}N_2$, 695.54245; found, 695.54346.

4,4'-(3,3'',5,5''-tetraisopropyl-[1,1':3',1''-terphenyl]-4,4''-diyl)bis(2,6-bis(2,4,6-triisopropylphenyl)pyridine) (C9_N)

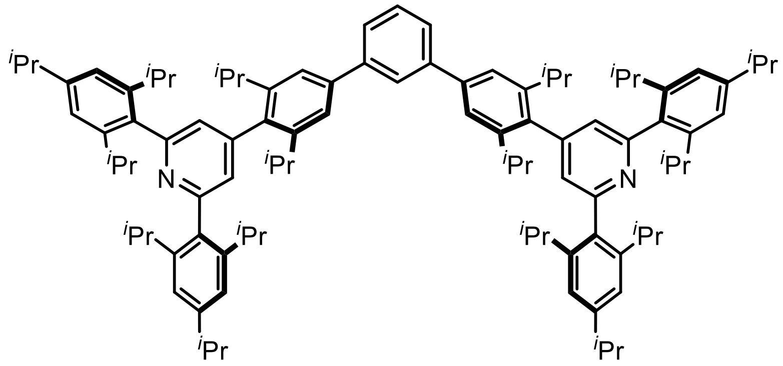

A 10 mL Schlenk flask was loaded with **Bpin_C4_N** (235 mg, 0.305 mmol, 2.4 equiv.), 1,3-dibromobenzene (30 mg, 0.127 mmol), $Pd(PPh_3)_4$ (29.4 mg, 0.025 mmol, 0.2 equiv.), and K_2CO_3 (84.3 mg, 0.61 mmol, 4.8 equiv.) and was placed under an inert atmosphere with three vacuum/N_2 cycles. Then, degassed toluene (1 mL), degassed 1,4-dioxane (1 mL), and degassed H_2O (1 mL) were added and the resulting suspension was stirred at 100°C for 18 h. The reaction mixture was allowed to cool to room temperature and NH_4Cl (sat. aq. soln. 10 mL) and CH_2Cl_2 (10 mL) were added. The layers were separated and the aqueous layer was extracted with CH_2Cl_2 (3 x 10 mL). The combined organic extracts were washed with brine (15 mL), dried over Na_2SO_4, filtered, and the solvent was removed under reduced pressure. The residue was purified by column chromatography (25 g of silica gel, hexanes/CH_2Cl_2 1:1, 2 CV) to give the title compound as a white solid (129 mg, 75% yield).

^1H NMR (400 MHz, $CDCl_3$) δ 7.85 (t, *J* = 1.8 Hz, 1H), 7.65 – 7.60 (m, 2H), 7.56 (dd, *J* = 8.8, 6.3 Hz, 1H), 7.48 (s, 4H), 7.19 (s, 4H), 7.04 (s, 8H), 2.97 – 2.82 (m, 8H), 2.76 (hept, *J* = 6.9 Hz, 8H), 1.26 (d, *J* = 7.0 Hz, 24H), 1.20 (d, *J* = 7.0 Hz, 24H, overlapped), 1.19 (d, *J* = 7.0 Hz, 24H, overlapped), 1.13 (d, *J* = 6.9 Hz, 24H).

^{13}C NMR (101 MHz, $CDCl_3$) δ 160.0, 148.9, 146.8, 146.1, 142.5, 141.6, 136.6, 136.4, 129.3, 126.56, 126.51, 124.54, 124.52, 122.1, 120.6, 34.6, 31.0, 30.8, 24.7, 24.3, 24.2, 23.6.

HRMS (ESI+) (m/z): [$M+2H^+$] calculated for $C_{100}H_{134}N_2$, 681.52680; found, 681.52679.

4,4'-(3,3'',5,5''-tetraisopropyl-[1,1':3',1''-terphenyl]-4,4''-diyl)bis(1-methyl-2,6-bis(2,4,6-triiso-propylphenyl)pyridin-1-ium) tetrafluoroborate ($C9^{2+}(BF_4^-)_2$)

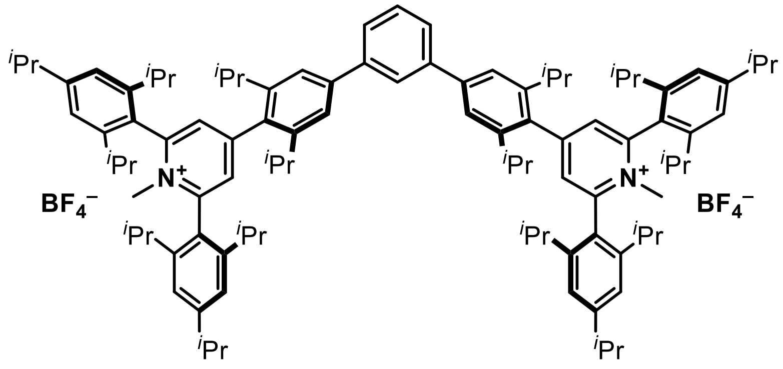

A 10 mL Schlenk flask was loaded with **C9_N** (84 mg, 0.062 mmol) and placed under an inert atmosphere with three vacuum/N_2 cycles. Dry CH_2Cl_2 (2 mL) was added and the mixture was cooled to 0 °C. Trimethyloxonium tetrafluoroborate (45.6 mg, 0.31 mmol, 5 equiv.) was added in one portion and the resulting suspension was stirred at 0 °C for 20 min and then at 25 °C for 10 min. Then, the reaction mixture was cooled to 0 °C and *N,N*-diisopropylethylamine (40 mg, 0.05 mL, 0.31 mmol, 5 equiv.) was added, followed by the addition of trimethyloxonium tetrafluoroborate (45.6 mg, 0.31 mmol, 5 equiv.), and the mixture was warmed to room temperature, and stirred for another 30 min. After that, it was separated between CH_2Cl_2 (10 mL) and H_2O (10 mL) and the aqueous layer was extracted with CH_2Cl_2 (2 x 5 mL). The combined organic extracts were dried over Na_2SO_4, filtered, and the solvent was removed under reduced pressure. The residue was purified by column chromatography (10 g of silica gel, CH_2Cl_2, 1 CV; then 10% *i*PrOH in CH_2Cl_2, 2 CV), the collected fractions were washed with $NaBF_4$ (10% aq. soln., 50 mL), filtered through a filter paper, and the solvent was evaporated under reduced pressure. The title compound was obtained as a yellowish solid (91 mg, 93% yield).

^{1}H NMR (400 MHz, $CDCl_3$) δ 7.83 (s, 4H), 7.67 (s, 1H), 7.62 – 7.58 (m, 3H), 7.49 (s, 4H), 7.24 (s, 8H), 3.75 (s, 6H), 2.99 (hept, *J* = 6.9 Hz, 4H), 2.55 (hept, *J* = 6.8 Hz, 4H), 2.21 (hept, *J* = 6.8 Hz, 8H), 1.33 (d, *J* = 6.9 Hz, 24H), 1.30 (d, *J* = 6.9 Hz, 24H), 1.24 (d, *J* = 6.9 Hz, 24H, overlapped), 1.22 (d, *J* = 6.9 Hz, 24H, overlapped).

^{13}C NMR (101 MHz, $CDCl_3$) δ 159.5, 157.7, 154.2, 145.9, 145.7, 143.9, 141.6, 131.4, 131.2, 129.8, 127.1, 126.4, 125.4, 123.0, 122.8, 43.7, 34.6, 32.8, 31.9, 24.9, 24.1, 23.9, 23.4.

^{19}F NMR (377 MHz, $CDCl_3$) δ -154.34, -154.39.

HRMS (ESI+) (m/z): [M^{2+}] calculated for $C_{102}H_{138}N_2$, 695.54245; found, 695.54266.

4,4'-(5'-(4-(2,6-bis(2,4,6-triisopropylphenyl)pyridin-4-yl)-3,5-diisopropylphenyl)-3,3'',5,5''-tetraisopropyl-[1,1':3',1''-terphenyl]-4,4''-diyl)bis(2,6-bis(2,4,6-triisopropylphenyl)pyridine) (C10_N)

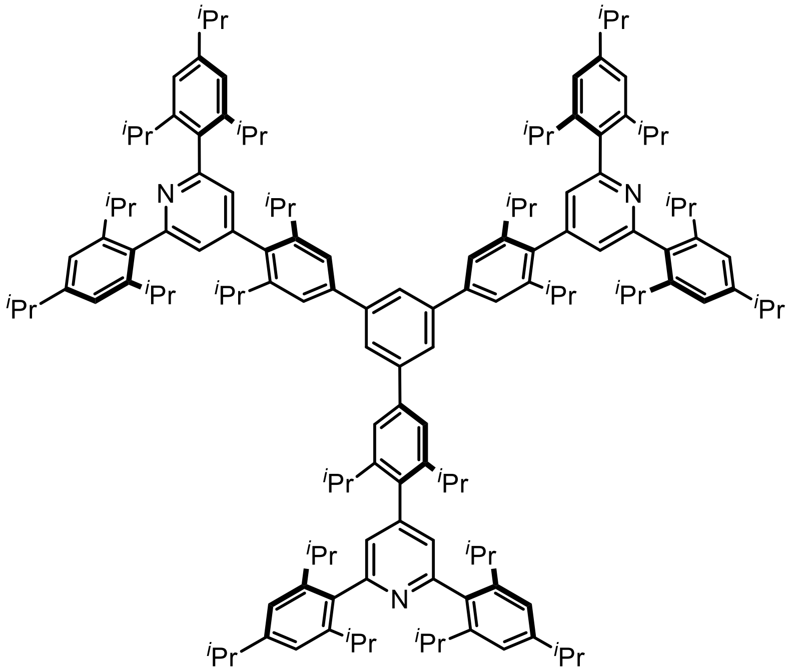

A 25 mL Schlenk flask was loaded with **Bpin_C4_N** (693 mg, 0.9 mmol, 3.6 equiv.), 1,3,5-tribromobenzene (80 mg, 0.249 mmol), $Pd(PPh_3)_4$ (43.3 mg, 0.037 mmol, 0.15 equiv.), and K_2CO_3 (249 mg, 1.8 mmol, 7.2 equiv.) and was placed under an inert atmosphere with three vacuum/N_2 cycles. Then, degassed toluene (3 mL), degassed 1,4-dioxane (3 mL), and degassed H_2O (3 mL) were added and the resulting suspension was stirred at 100 °C for 18 h. The reaction mixture was allowed to cool to room temperature and NH_4Cl (sat. aq. soln. 15 mL) and CH_2Cl_2 (15 mL) were added. The layers were separated and the aqueous layer was extracted with CH_2Cl_2 (3 x 15 mL). The combined organic extracts were washed with brine (30 mL), dried over Na_2SO_4, filtered, and the solvent was removed under reduced pressure. The residue was purified by column chromatography (100 g of silica gel, hexanes/CH_2Cl_2 1:1, 2 CV; hexanes/CH_2Cl_2 1:2, 1 CV) to give the title compound as a white solid (300 mg, 60% yield).

^{1}H NMR (500 MHz, $CDCl_3$) δ 7.82 (s, 3H), 7.55 (s, 6H), 7.21 (s, 6H), 7.04 (s, 12H), 2.96 – 2.83 (hept, J = 6.9 Hz, 6H + hept, J = 6.9 Hz, 6H), 2.79 – 2.68 (m, 12H), 1.26 (d, *J* = 6.9 Hz, 36H), 1.20 (d, *J* = 6.9 Hz, 36H, overlapped), 1.19 (d, *J* = 6.9 Hz, 36H, overlapped), 1.13 (d, *J* = 6.9 Hz, 36H).

^{13}C NMR (126 MHz, $CDCl_3$) δ 160.0, 149.0, 148.6, 146.9, 146.1, 143.1, 141.79, 141.77, 136.5, 125.9, 124.6, 122.3, 120.7, 34.6, 31.1, 30.9, 24.7, 24.25, 24.23, 23.6.

HRMS (ESI+) (m/z): [M+3H$^+$] calculated for $C_{147}H_{198}N_3$, 668.51898; found, 668.52039.

C10^{3+}(BF$_4^-$)$_3$

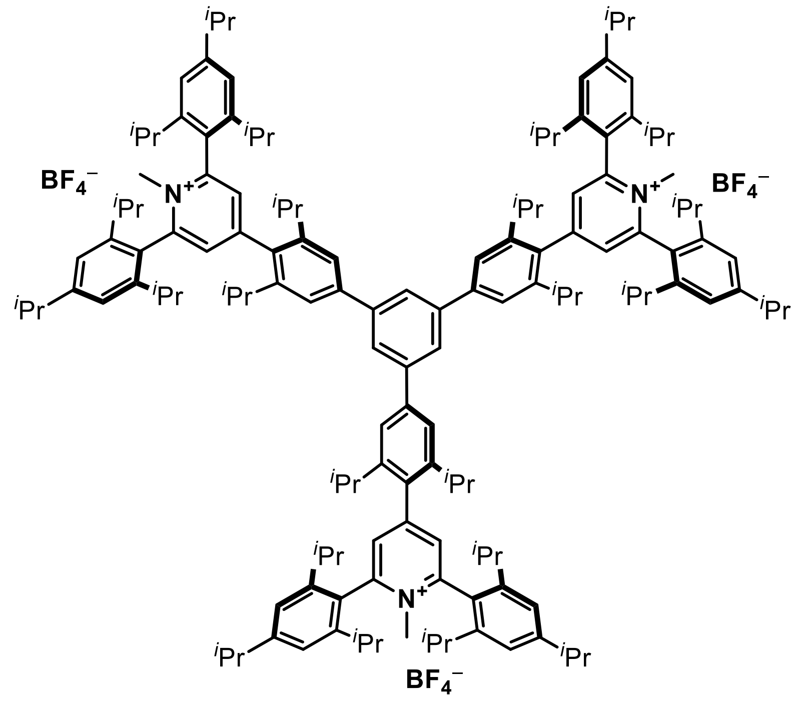

A 10 mL Schlenk flask was loaded with **C10_N** (268 mg, 0.134 mmol) and placed under an inert atmosphere with three vacuum/N_2 cycles. Dry CH_2Cl_2 (3.5 mL) was added and the mixture was cooled to 0 °C. Trimethyloxonium tetrafluoroborate (313 mg, 2.01 mmol, 15 equiv.) was added in one portion and the resulting suspension was stirred at 0 °C for 2 h. Then, *N,N*-diisopropylethylamine (260 mg, 0.35 mL, 2.01 mmol, 15 equiv.) was added, followed by the addition of trimethyloxonium tetrafluoroborate (313 mg, 2.01 mmol, 15 equiv.), and the mixture was warmed to room temperature, and stirred for 30 min. *N,N*-diisopropylethylamine (260 mg, 0.35 mL, 2.01 mmol, 15 equiv.) was added again, followed by the addition of trimethyloxonium tetrafluoroborate (313 mg, 2.01 mmol, 15 equiv.), and the reaction mixture was stirred for another 30 min. Then, *N,N*-diisopropylethylamine (260 mg, 0.35 mL, 2.01 mmol, 15 equiv.) was added once again, followed by the addition of trimethyloxonium tetrafluoroborate (313 mg, 2.01 mmol, 15 equiv.), and the reaction mixture was stirred for 5 min. After that, it was separated between CH_2Cl_2 (20 mL) and H_2O (20 mL) and the aqueous layer was extracted with CH_2Cl_2 (2 x 20 mL). The combined organic extracts were dried over Na_2SO_4, filtered, and the solvent was removed under reduced pressure. The residue was purified by column chromatography (50 g of silica gel, CH_2Cl_2, 1 CV; then 10% *i*PrOH in CH_2Cl_2, 3 CV), the collected fractions were washed with $NaBF_4$ (10% aq. soln., 50 mL), filtered through a filter paper, and the solvent was evaporated under reduced pressure. The title compound was obtained as greenish crystals (235 mg, 76% yield).

^{1}H NMR (500 MHz, $CDCl_3$) δ 7.84 (s, 6H), 7.66 (s, 3H), 7.52 (s, 6H), 7.23 (s, 12H), 3.72 (s, 9H), 2.99 (hept, *J* = 6.9 Hz, 6H), 2.57 (hept, *J* = 6.9 Hz, 6H), 2.19 (hept, *J* = 6.7 Hz, 12H), 1.31 (d, *J* = 6.8 Hz, 36H, overlapped), 1.30 (d, *J* = 6.8 Hz, 36H, overlapped), 1.24 (d, *J* = 6.8 Hz, 36H), 1.21 (d, *J* = 6.8 Hz, 36H).

^{13}C NMR (126 MHz, $CDCl_3$) δ 159.5, 157.7, 154.2, 146.0, 145.7, 143.7, 142.6, 131.7, 131.2, 126.2, 125.4, 123.05, 123.00, 43.6, 34.6, 32.8, 31.9, 24.9, 24.1, 23.9, 23.4.

^{19}F NMR (471 MHz, $CDCl_3$) δ -154.36, -154.41.

HRMS (ESI+) (m/z): [M^{3+}] calculated for $C_{150}H_{204}N_3$, 682.53463; found, 682.5351.

2.7 Synthesis of anions present in 9-10

Ethyl 5'-(2,6-diisopropylphenyl)-2,2'',4,4'',6,6''-hexaisopropyl-[1,1':3',1''-terphenyl]-2'-sulfonate (A4_SO_3Et)

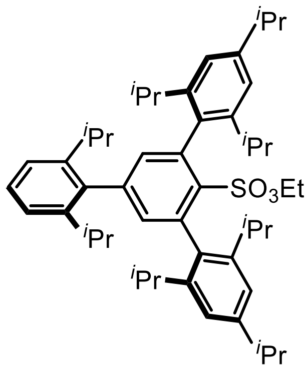

A 25 mL Schlenk flask was loaded with **Na^+A4$^-$** (280 mg, 0.376 mmol) and placed under an inert atmosphere with three vacuum/N_2 cycles. Dry CH_2Cl_2 (5 mL) was added and the mixture was cooled to 0 °C. Triethyloxonium tetrafluoroborate (357 mg, 1.88 mmol, 5 equiv.) was added in one portion and the resulting suspension was stirred at 0 °C for 2 h. Then, H_2O (10 mL) was added to the reaction mixture, it was extracted with CH_2Cl_2 (3 x 15 mL), and the combined organic extracts were washed with brine (10 mL), dried over Na_2SO_4, filtered, and the solvent was removed under reduced pressure. The residue was purified by column chromatography (20 g of silica gel, hexanes/CH_2Cl_2 1:1), to afford the title compound as a colourless solid (235 mg, 83% yield).

^{1}H NMR (400 MHz, $CDCl_3$) δ 7.30 (t, *J* = 7.8 Hz, 1H), 7.15 (d, *J* = 7.8 Hz, 2H), 7.13 (s, 2H), 7.02 (s, 4H), 3.50 (q, *J* = 7.1 Hz, 2H), 2.92 (hept, *J* = 6.9 Hz, 2H), 2.77 – 2.62 (hept, *J* = 6.9 Hz, 2H + hept, *J* = 6.9 Hz, 4H), 1.29 (d, *J* = 6.9 Hz, 12H), 1.27 (d, *J* = 6.9 Hz, 12H), 1.10 (d, *J* = 6.9 Hz, 12H), 1.07 (d, *J* = 6.9 Hz, 12H), 0.91 (t, *J* = 7.2 Hz, 3H).

^{13}C NMR (101 MHz, $CDCl_3$) δ 148.3, 146.6, 146.2, 144.2, 141.2, 136.8, 135.5, 135.1, 133.3, 128.5, 122.7, 120.5, 64.4, 34.3, 31.5, 30.6, 25.7, 24.6, 24.2, 23.2, 15.4.

HRMS (ESI+) (m/z): [M+H^+] calculated for $C_{50}H_{71}O_3S$, 751.51184; found, 751.51123.

Ethyl 5'-(2,6-diisopropyl-4-(4,4,5,5-tetramethyl-1,3,2-dioxaborolan-2-yl)phenyl)-2,2'',4,4'',6,6''-hexaisopropyl-[1,1':3',1''-terphenyl]-2'-sulfonate (Bpin_A4_SO_3Et)

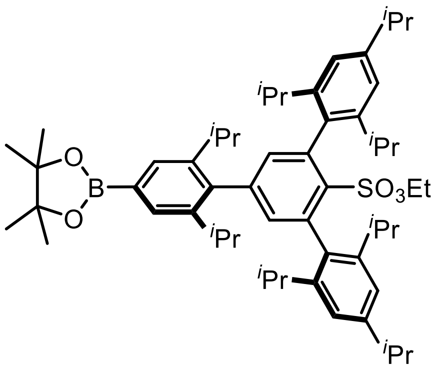

A flame dried 10 mL Schlenk flask was loaded with 4,4'-di-tert-butyl-2,2'-bipyridyl (4.93 mg, 0.018 mmol, 0.06 equiv.), (1,5-cyclooctadiene)(methoxy)iridium(I) dimer (6.09 mg, 0.009 mmol, 0.03 equiv.), bis(pinacolato)diboron (117 mg, 0.459 mmol, 1.5 equiv.), and **A4_SO_3Et** (230 mg, 0.306 mmol) and was placed under an inert atmosphere with three vacuum/N_2 cycles. THF (4 mL) was added, the flask was sealed, and the resulting mixture was stirred at 80 °C for 12 h. Then, the mixture was allowed to cool to room temperature, hexane (5 mL) was added, and the solvent was removed under reduced pressure. The residue was purified by column chromatography (25 g of silica gel, hexanes/CH_2Cl_2 1:1) to give the title compound as a colourless solid (195 mg, 73% yield).

^{1}H NMR (500 MHz, $CDCl_3$) δ 7.61 (s, 2H), 7.10 (s, 2H), 7.02 (s, 4H), 3.49 (q, *J* = 7.1 Hz, 2H), 2.92 (hept, *J* = 6.9 Hz, 2H), 2.72 (hept, *J* = 6.9 Hz, 2H), 2.66 (hept, *J* = 6.9 Hz, 4H), 1.36 (s, 12H), 1.29 (d, *J* = 6.8 Hz, 12H), 1.27 (d, *J* = 6.8 Hz, 12H), 1.13 (d, *J* = 6.9 Hz, 12H), 1.06 (d, *J* = 6.8 Hz, 12H), 0.91 (t, *J* = 7.1 Hz, 3H).

^{13}C NMR (126 MHz, $CDCl_3$) δ 148.2, 146.2, 145.7, 144.2, 141.3, 139.9, 135.6, 135.0, 133.0, 129.2, 120.5, 83.8, 64.4, 34.3, 31.5, 30.6, 25.7, 25.0, 24.6, 24.2, 23.1, 15.4.

HRMS (ESI+) (m/z): [M+H$^+$] calculated for $C_{56}H_{82}BO_5S$, 876.60068; found, 876.59814.

Ethyl 2,2'',4,6,6''-pentaisopropyl-5'-(2,4,6-triisopropylphenyl)-4'''-(5,15,20-triphenylporphyrin-10-yl)-[1,1':3',1'':4'',1'''-quaterphenyl]-6'-sulfonate (A8_SO₃Et)

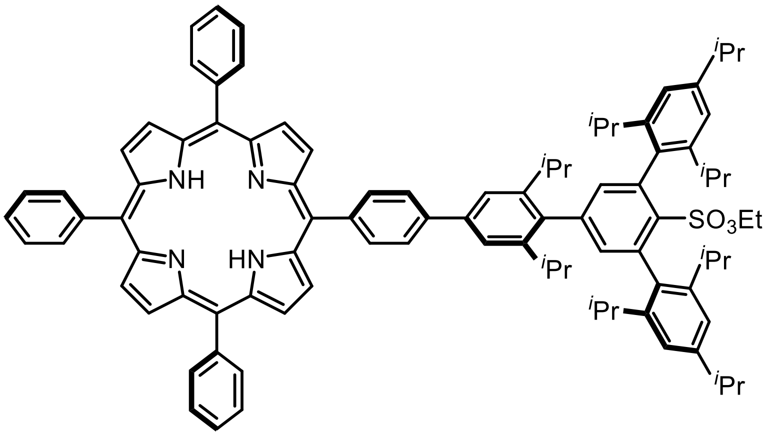

A 10 mL Schlenk flask was loaded with **Bpin_A4_SO₃Et** (100 mg, 0.114 mmol), 5-(4-iodophenyl)-10,15,20-triphenylporphyrin[9] (92.9 mg, 0.125 mmol, 1.1 equiv.), $Pd(PPh_3)_4$ (26.3 mg, 0.023 mmol, 0.2 equiv.), and Na_2CO_3 (12.1 mg, 0.114 mmol, 1 equiv.) and was placed under an inert atmosphere with three vacuum/N_2 cycles. Then, degassed toluene (0.5 mL), degassed 1,4-dioxane (1 mL), and degassed H_2O (0.5 mL) were added and the resulting suspension was stirred at 75 °C for 18 h. The reaction mixture was allowed to cool to room temperature and NH_4Cl (sat. aq. soln. 10 mL) was added, it was extracted with CH_2Cl_2 (3 x 10 mL), and the combined organic extracts were washed with brine (30 mL), dried over Na_2SO_4, filtered, and the solvent was removed under reduced pressure. The residue was purified by column chromatography (25 g of silica gel, hexanes/CH_2Cl_2 1:1) to give the title compound as a dark-purple solid (80 mg, 52% yield).

^{1}H NMR (500 MHz, C_6D_6) δ 9.11 (d, *J* = 4.8 Hz, 2H), 9.01 (d, *J* = 4.8 Hz, 2H), 8.94 (s, 4H), 8.34 (d, *J* = 8.0 Hz, 2H), 8.16 – 8.09 (m, 6H), 8.06 (d, *J* = 8.1 Hz, 2H), 7.88 (s, 2H), 7.52 – 7.40 (m, 9H), 7.27 (s, 2H), 7.24 (s, 4H), 3.47 (q, *J* = 7.1 Hz, 2H), 3.12 – 3.00 (m, 6H), 2.89 (hept, *J* = 6.9 Hz, 2H), 1.56 (d, *J* = 6.7 Hz, 12H), 1.32 (d, *J* = 6.8 Hz, 12H), 1.29 (d, *J* = 6.9 Hz, 12H), 1.26 (d, *J* = 6.8 Hz, 12H), 0.67 (t, *J* = 7.1 Hz, 3H), -2.04 (s, 2H).

^{13}C NMR (126 MHz, C_6D_6) δ 148.8, 148.6, 147.7, 146.77, 146.75, 146.2, 144.4, 144.2, 142.9, 142.8, 142.2, 141.8, 141.72, 141.67, 141.5, 137.0, 136.7, 136.0, 135.7, 134.9, 133.9, 133.5, 132.5, 132.4, 131.59, 131.57, 129.9, 128.4, 127.0, 126.0, 122.4, 120.84, 120.81, 120.7, 120.4, 64.3, 34.8, 32.0, 31.1, 26.0, 24.9, 24.4, 23.6, 15.3.

HRMS (ESI+) (m/z): [M+H$^+$] calculated for $C_{94}H_{99}O_3N_4S$, 1363.74324; found, 1363.74494.

Sodium 2,2'',4,6,6''-pentaisopropyl-5'-(2,4,6-triisopropylphenyl)-4'''-(5,15,20-triphenylporphyrin-10-yl)-[1,1':3',1'':4'',1'''-quaterphenyl]-6'-sulfonate (Na^+A8^-)

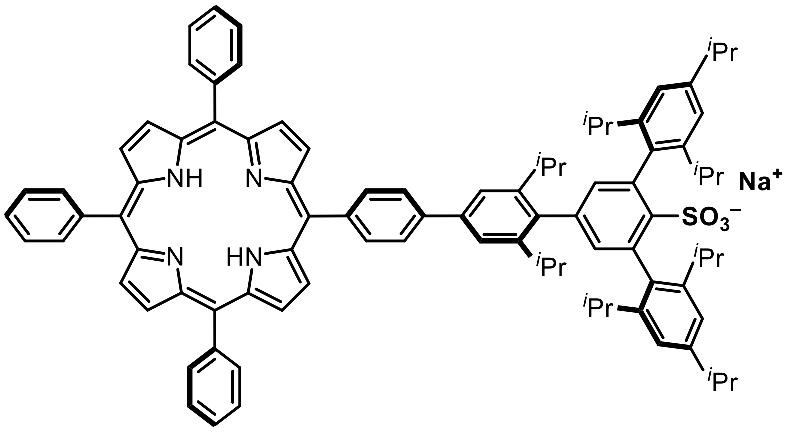

The reaction was performed in an N_2-filled glovebox. **A8_SO₃Et** (70 mg, 0.051 mmol) was dissolved in THF (1 mL) and *t*-BuOK (23 mg, 0.205 mmol, 4 equiv.) was added at room temperature. The reaction mixture was stirred for 12 h, then taken out of the glovebox and 3 drops of water and CH_2Cl_2 (2 mL) were added. The suspension was loaded directly on a silica gel column (20 g of silica gel) and purified by column chromatography (CH_2Cl_2, 1 CV; then 10% *i*PrOH in CH_2Cl_2, 3 CV), the collected fractions were washed with NaOH (5% aq. soln., 2 x 50 mL), dried over Na_2SO_4, filtered, and concentrated under reduced pressure to give the title compound as a dark-red solid (49 mg, 70% yield).

^{1}H NMR (500 MHz, $CDCl_3$) δ 8.99 (d, *J* = 4.8 Hz, 2H), 8.93 – 8.84 (m, 6H), 8.32 (d, *J* = 7.9 Hz, 2H), 8.29 – 8.21 (m, 6H), 8.03 (d, *J* = 7.9 Hz, 2H), 7.83 – 7.74 (m, 9H), 7.71 (s, 2H), 7.12 (s, 2H), 7.02 (s, 4H), 3.02 – 2.89 (m, 4H), 2.81 (hept, *J* = 6.8 Hz, 4H), 1.33 (d, *J* = 6.9 Hz, 12H), 1.30 – 1.28 (m, 12H), 1.26 (d, *J* = 6.8 Hz, 12H), 1.12 (d, *J* = 6.7 Hz, 12H), -2.71 (s, 2H).

^{13}C NMR (126 MHz, $CDCl_3$) δ 147.7, 147.4, 147.3, 147.1, 147.0, 146.0, 142.35, 142.33, 141.2, 141.1, 141.0, 140.4, 140.2, 139.6, 139.4, 137.6, 137.4, 135.2, 134.7, 132.5, 132.3, 132.2, 132.0, 131.3 (broad), 128.7, 128.6, 127.9, 126.8, 125.6, 121.8, 120.31, 120.29, 120.1, 119.8, 119.7, 34.2, 31.4, 30.7, 25.7, 24.9, 24.4, 23.4.

HRMS (ESI-) (m/z): [M-Na$^+$] calculated for $C_{92}H_{93}O_3N_4S$, 1333.69629; found, 1333.69748.

Palladium (II) sodium 10-(2,2'',4,6,6''-pentaisopropyl-6'-sulfonato-5'-(2,4,6-triisopropylphenyl)-[1,1':3',1'':4'',1'''-quaterphenyl]-4'''-yl)-5,15,20-triphenylporphyrin-21,23-diide (Na^{+}A9^{-})

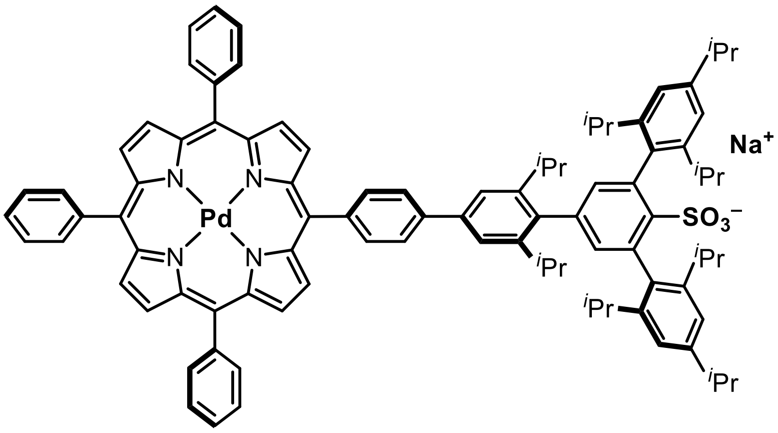

The reaction was performed in an N_2-filled glovebox. **Na^{+}A8^{-}** (42 mg, 0.031 mmol) was dissolved in C_6D_6 (1 mL) and bis(benzonitrile)palladium (II) 1,1,1,3,3,3-hexafluoro-2-(trifluoromethyl)propan-2-olate[10] (29 mg, 0.037 mmol, 1.2 equiv.) was added in one portion. The reaction mixture was stirred for 12 h at room temperature (until the starting material was consumed based on the absence of the characteristic signal from porphyrin N-*H* protons at -2.7 ppm in the ^{1}H NMR spectrum). Then, the mixture was loaded on a silica gel column (15 g of silica gel) and purified by column chromatography (10% *i*PrOH in CH_2Cl_2 3 CV), the collected fractions were washed with NaOH (5% aq. soln., 2 x 10 mL), dried over Na_2SO_4, filtered, and concentrated under reduced pressure to give the title compound as a red solid (42 mg, 93% yield).

^{1}H NMR (500 MHz, $CDCl_3$) δ 8.93 (d, *J* = 4.9 Hz, 2H), 8.85 – 8.77 (m, 6H), 8.25 (d, *J* = 8.2 Hz, 2H), 8.21 – 8.14 (m, 6H), 7.98 (d, *J* = 8.2 Hz, 2H), 7.80 – 7.72 (m, 9H), 7.67 (s, 2H), 7.08 (s, 2H), 6.99 (s, 4H), 2.98 – 2.88 (m, 4H), 2.84 – 2.74 (m, 4H), 1.32 – 1.25 (m, 36H, overlapped with the signal from grease), 1.11 (d, *J* = 6.9 Hz, 12H).

^{13}C NMR (126 MHz, $CDCl_3$) δ 147.96, 147.65, 147.43, 146.83, 141.94, 141.76, 141.74, 141.18, 140.76, 140.44, 139.68, 139.65, 137.36, 137.29, 134.69, 134.27, 132.78, 131.25, 131.16, 128.47, 128.22, 127.92, 126.87, 125.57, 121.91, 121.78, 121.71, 120.04, 119.97, 34.30, 31.38, 30.73, 25.60, 24.88, 24.36, 23.40.

HRMS (ESI-) (m/z): [M-Na^{+}] calculated for $C_{92}H_{91}N_4O_3PdS$, 1437.5852; found, 1437.5892.

A10_SO$_3$Et

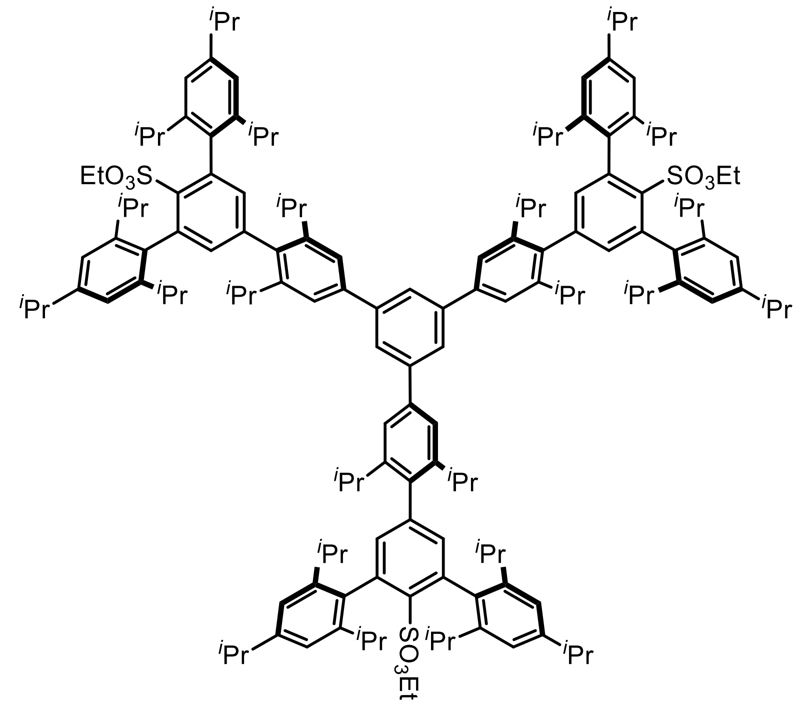

A 10 mL Schlenk flask was loaded with **Bpin_A4_SO$_3$Et** (95.5 mg, 0.109 mmol, 3.5 equiv.), 1,3,5-tribromobenzene (10 mg, 0.031 mmol), Pd(PPh$_3$)$_4$ (10.8 mg, 0.009 mmol, 0.3 equiv.), and Na_2CO_3 (23.1 mg, 0.218 mmol, 7 equiv.) and was placed under an inert atmosphere with three vacuum/N_2 cycles. Then, degassed toluene (1 mL), degassed 1,4-dioxane (1 mL), and degassed H_2O (1 mL) were added and the resulting suspension was stirred at 85 °C for 18 h. The reaction mixture was allowed to cool to room temperature and NH_4Cl (sat. aq. soln. 10 mL) was added, it was extracted with CH_2Cl_2 (3 x 10 mL), and the combined organic extracts were washed with brine (30 mL), dried over Na_2SO_4, filtered, and the solvent was removed under reduced pressure. The residue was dissolved in dry CH_2Cl_2 (1 mL) under an N_2 atmosphere and the mixture was cooled to 0 °C. Triethyloxonium tetrafluoroborate (88.6 mg, 0.466 mmol, 15 equiv.) was added in one portion and the resulting suspension was stirred at 25 °C for 24 h. Then, H_2O (10 mL) was added to the reaction mixture, it was extracted with CH_2Cl_2 (3 x 15 mL), and the combined organic extracts were washed with brine (10 mL), dried over Na_2SO_4, filtered, and the solvent was removed under reduced pressure. The residue was purified by column chromatography (20 g of silica gel, hexanes/CH_2Cl_2 1:1), to afford the title compound as a colourless solid (55 mg, 76% yield).

^{1}H NMR (500 MHz, CDCl$_3$) δ 7.68 (s, 3H), 7.43 (s, 6H), 7.16 (s, 6H), 7.01 (s, 12H), 3.50 (q, *J* = 7.2 Hz, 6H), 2.91 (hept, *J* = 6.8 Hz, 6H), 2.79 (hept, *J* = 6.8 Hz, 6H), 2.66 (hept, *J* = 6.9 Hz, 12H), 1.28 (d, *J* = 6.9 Hz, 36H, overlapped), 1.27 (d, *J* = 6.9 Hz, 36H, overlapped), 1.14 (d, *J* = 6.8 Hz, 36H), 1.06 (d, *J* = 6.8 Hz, 36H), 0.91 (t, *J* = 7.2 Hz, 9H).

^{13}C NMR (126 MHz, CDCl$_3$) δ 148.3, 147.3, 146.2, 143.8, 143.1, 141.6, 141.4, 136.5, 135.7, 135.0, 133.4, 125.6, 122.2, 120.5, 64.4, 34.3, 31.6, 30.8, 25.7, 24.7, 24.2, 23.1, 15.4.

HRMS (ESI+) (m/z): [M+2Na$^+$] calculated for $C_{156}H_{210}O_9S_3$, 1184.74607; found, 1184.75537.

$(Na^+)_3A10^{3-}$

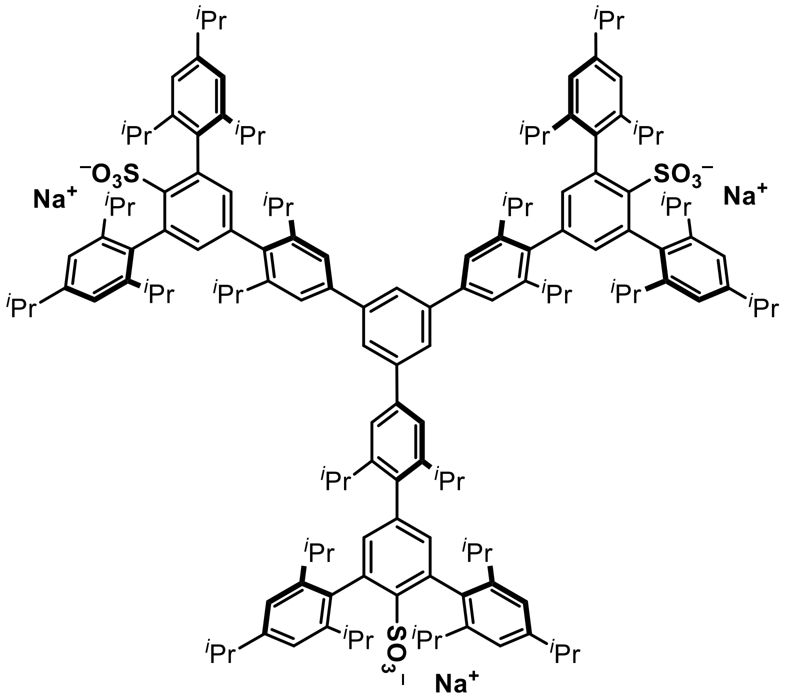

The reaction was performed in an N_2-filled glovebox. **A10_SO$_3$Et** (55 mg, 0.024 mmol) was dissolved in THF (1 mL) and *t*BuOK (39.9 mg, 0.356 mmol, 15 equiv.) was added at room temperature. The reaction mixture was stirred for 18 h, then taken out of the glovebox, and 3 drops of water and CH_2Cl_2 (2 mL) were added. The suspension was loaded directly on a silica gel column (10 g of silica gel) and purified by column chromatography (CH_2Cl_2, 1 CV; then 10% *i*PrOH in CH_2Cl_2, 3 CV; then 20% *i*PrOH in CH_2Cl_2, 3 CV), the collected fractions were washed with NaOH (5% aq. soln., 2 x 50 mL), dried over Na_2SO_4, filtered, and concentrated under reduced pressure to give the title compound as an off-white solid (30 mg, 55% yield).

^{1}H NMR (400 MHz, $CDCl_3$-MeOD) δ 7.65 (s, 3H), 7.38 (s, 6H), 6.95 (s, 6H), 6.93 (s, 12H), 2.81 (m, 24H), 1.23 (d, *J* = 6.8 Hz, 36H, overlapped), 1.22 (d, *J* = 6.8 Hz, 36H, overlapped), 1.09 (d, *J* = 6.8 Hz, 36H), 1.01 (d, *J* = 6.9 Hz, 36H).

^{13}C NMR (101 MHz, $CDCl_3$-MeOD) δ 147.8, 147.2, 146.8, 143.4, 141.2, 140.9, 140.8, 140.1, 138.1, 137.7, 132.9, 125.7, 122.2, 120.1, 78.0, 77.7, 77.4, 34.5, 31.5, 25.7, 24.9, 24.2, 23.2.

HRMS (ESI-) (m/z): [M-3Na$^+$] calculated for $C_{150}H_{195}O_9S_3$, 745.46599; found, 745.46581.

2.8 Synthesis and characterization of the ion pairs

A general procedure for ion pair formation

A solution of the cation BF_4^- salt (1 equiv.) in CH_2Cl_2 (1.5 mL) was added to a solution of the anion Na^+ salt (1 equiv.; or 2 equiv. for **7-9**) in CH_2Cl_2 (1 mL) under air at room temperature. After 10 min at room temperature (with occasional swirling of the solution), the solvent was removed under reduced pressure and the residue was re-dissolved in $CDCl_3$ (0.6 mL). The ratio of both components was confirmed by ^1H NMR analysis and the amounts were adjusted if necessary. Then, the NMR sample was diluted with CH_2Cl_2 (5 mL), H_2O (5 mL) was added, and the mixture was stirred for 10 min. After that, the layers were separated, the organic layer was washed with H_2O (2 x 5 mL), filtered through a CH_2Cl_2-soaked filter paper, the solvent was removed under reduced pressure, and the product was dried under vacuum.

C2$^+$A4$^-$

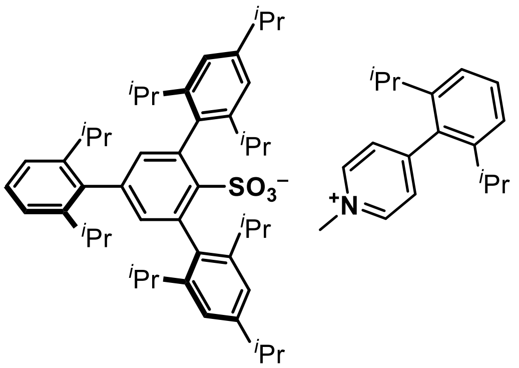

Prepared following the general procedure from **C2$^+$BF$_4^-$** (3 mg, 0.0088 mmol) and **Na$^+$A4$^-$** (6.55 mg, 0.0088 mmol). The ion pair was obtained as a yellowish crystalline solid (7.1 mg, 83%). Single crystals of **C2$^+$A4$^-$** were obtained by slow diffusion of hexane into a solution of the ion pair in chlorobenzene (0.2 mL) at room temperature.

^1H NMR (500 MHz, C_6D_6) δ 8.59 (d, *J* = 6.0 Hz, 2H), 7.30 (t, *J* = 7.7 Hz, 1H), 7.26 (d, *J* = 5.9 Hz, 2H), 7.22 (t, *J* = 7.7 Hz, 1H), 7.18 (d, 2H), 7.12 (s, 4H), 7.07 (d, *J* = 7.8 Hz, 2H), 7.04 (s, 2H), 4.20 (s, 3H), 3.27 (hept, *J* = 7.1 Hz, 4H), 3.08 (hept, *J* = 7.0 Hz, 2H), 2.75 (hept, *J* = 7.0 Hz, 2H), 2.24 (hept, *J* = 7.0 Hz, 2H), 1.50 (d, *J* = 6.6 Hz, 12H), 1.24 (d, *J* = 6.8 Hz, 12H), 1.19 (d, *J* = 6.7 Hz, 12H), 1.14 (d, *J* = 6.8 Hz, 12H), 1.04 (d, *J* = 6.7 Hz, 12H).

^{13}C NMR (126 MHz, C_6D_6) δ 157.4, 147.5, 147.1, 147.0, 146.2, 145.7, 144.2, 140.4, 140.0, 139.7, 138.7, 134.4, 132.5, 130.3, 123.5, 122.8, 119.7, 48.1, 34.7, 31.7, 30.8, 30.6, 26.0, 25.0, 24.5, 24.3, 24.0.

C4⁺A2⁻

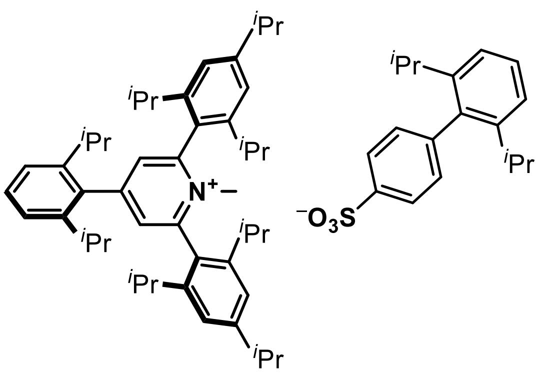

Prepared following the general procedure from **$C4^+BF_4^-$** (3 mg, 0.0088 mmol) and **Na^+A2^-** (3.9 mg, 0.0088 mmol). The ion pair was obtained as a yellowish crystalline solid (7.1 mg, 83%). Single crystals of **$C4^+A2^-$** were obtained by slow evaporation of a solution in Et_2O/hexanes/p-xylene at room temperature.

^{1}H NMR (500 MHz, C_6D_6/$CDCl_3$ 9:1) δ 8.01 (d, *J* = 8.0 Hz, 2H), 7.23 (t, *J* = 7.8 Hz, 2H), 7.12 (d, *J* = 7.7 Hz, 2H), 7.09 (s, 2H), 7.07 (d, *J* = 7.9 Hz, 2H), 7.02 (d, *J* = 7.9 Hz, 2H), 3.93 (s, 3H), 2.86 (hept, *J* = 7.0 Hz, 2H), 2.74 (hept, *J* = 6.9 Hz, 2H), 2.23 (hept, *J* = 6.9 Hz, 2H), 2.07 (hept, *J* = 6.8 Hz, 4H), 1.31 (d, *J* = 6.8 Hz, 12H), 1.25 (d, *J* = 6.8 Hz, 12H), 1.05 (d, *J* = 6.8 Hz, 12H), 1.01 (d, *J* = 6.7 Hz, 12H), 0.97 (d, *J* = 6.8 Hz, 12H). One 4H singlet signal is likely overlapped with the residual solvent signal.

^{13}C NMR (101 MHz, $CDCl_3$) δ 159.9, 157.5, 154.3, 147.2, 146.0, 145.6, 144.9, 140.7, 140.0, 131.9, 131.1, 128.7, 127.6, 126.5, 125.4, 123.7, 123.1, 122.4, 43.8, 34.6, 32.8, 31.8, 30.0, 24.8, 24.3, 24.1, 23.9, 23.5.

C3⁺A3⁻

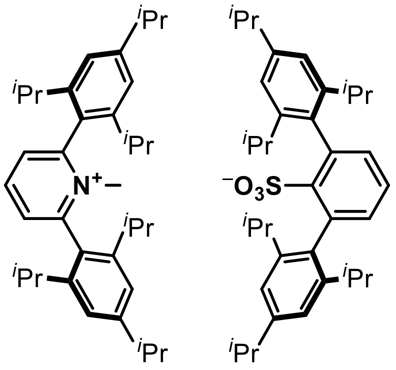

Prepared following the general procedure from **$C3^+BF_4^-$** (10 mg, 0.0171 mmol) and **Na^+A3^-** (10 mg, 0.0171 mmol). The ion pair was obtained as a colourless crystalline solid (17 mg, 94%). Single crystals of **$C3^+A3^-$** were obtained by slow evaporation of a solution in hexane at room temperature.

^{1}H NMR (500 MHz, C_6D_6) δ 8.87 (t, *J* = 7.9 Hz, 1H), 7.86 (d, *J* = 7.9 Hz, 2H), 7.25 (s, 4H), 7.18 (d, *J* = 7.3 Hz, 2H), 7.09 (t, *J* = 7.5 Hz, 1H), 7.05 (s, 4H), 3.38 (hept, *J* = 6.9 Hz, 4H), 2.91 (hept, *J* = 6.9 Hz, 2H), 2.71 (hept, *J* = 6.9 Hz, 2H), 2.63 (s, 3H), 2.04 (hept, *J* = 6.9 Hz, 4H), 1.62 (d, *J* = 6.9 Hz, 12H), 1.30 (d, *J* = 6.9 Hz, 12H, overlapped), 1.29 (d, *J* = 6.9 Hz, 12H, overlapped), 1.16 (d, *J* = 6.9 Hz, 12H), 1.05 (d, *J* = 6.6 Hz, 12H), 0.94 (d, *J* = 6.9 Hz, 12H).

^{13}C NMR (126 MHz, C_6D_6) δ 155.0, 152.7, 151.7, 147.2, 146.8, 146.7, 146.0, 141.1, 140.2, 131.5, 131.5, 127.4, 126.5, 122.3, 119.9, 41.6, 34.8, 34.7, 31.8, 31.7, 25.8, 24.9, 24.7, 24.0, 23.9, 23.3.

Additional NMR spectra available in section 3.1 NMR spectra: COSY, HSQC, HMBC, and ROESY in C_6D_6.

1 ($C4^{+}A3^{-}$)

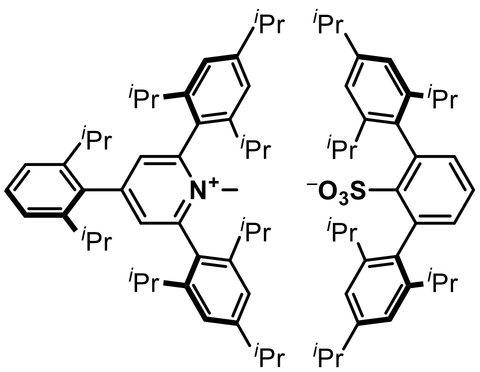

Prepared following the general procedure from **$C4^{+}BF_4^{-}$** (3 mg, 0.0088 mmol) and **$Na^{+}A3^{-}$** (6.55 mg, 0.0088 mmol). The ion pair was obtained as a yellowish crystalline solid (7.1 mg, 83%). Single crystals of **1** were obtained by slow evaporation of a solution in hexane at room temperature.

^{1}H NMR (500 MHz, C_6D_6) δ 7.21 (s, 8H), 7.18 (t, *J* = 7.6 Hz, 1H), 7.09 (d, *J* = 7.0 Hz, 2H), 7.02 (t, *J* = 7.0 Hz, 1H), 6.99 (d, *J* = 7.9 Hz, 2H), 6.86 (s, 2H), 3.88 (s, 3H), 3.20 (hept, *J* = 6.8 Hz, 4H), 3.04 – 2.97 (m, 2H), 2.97 – 2.91 (m, 2H), 2.23 (hept, *J* = 6.9 Hz, 2H), 2.06 (hept, *J* = 6.8 Hz, 4H), 1.51 (d, *J* = 6.7 Hz, 12H), 1.43 (d, *J* = 7.0 Hz, 12H), 1.36 (d, *J* = 6.8 Hz, 12H), 1.32 (d, *J* = 6.8 Hz, 12H), 1.25 (d, *J* = 6.8 Hz, 12H), 0.99 (d, *J* = 6.8 Hz, 12H), 0.94 (d, *J* = 6.8 Hz, 12H).

^{13}C NMR (126 MHz, C_6D_6) δ 157.9, 157.4, 153.3, 146.9, 145.8, 145.6, 145.2, 141.0, 140.0, 133.2, 131.3, 130.6, 130.4, 126.4, 126.2, 123.3, 122.8, 119.7, 46.3, 34.90, 34.87, 32.7, 31.5, 31.3, 25.7, 25.0, 24.8, 24.2, 24.1, 24.0, 23.6.

2 ($C4^{+}A4^{-}$)

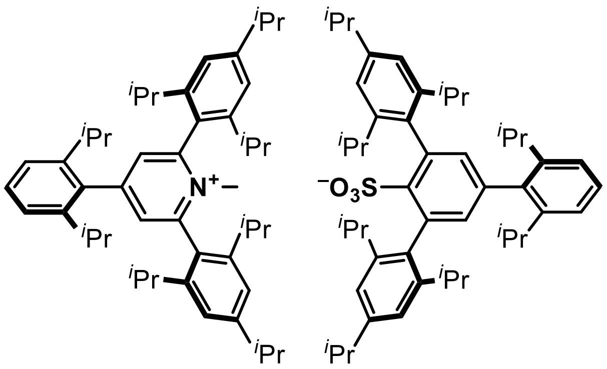

Prepared following the general procedure from **$C4^{+}BF_4^{-}$** (12.7 mg, 0.017 mmol) and **$Na^{+}A4^{-}$** (12.7 mg, 0.017 mmol). The ion pair was obtained as a yellowish crystalline solid (21 mg, 87%). Single crystals of **2** were obtained by slow evaporation of a solution in hexane at room temperature.

^{1}H NMR (400 MHz, $CDCl_3$) δ 7.81 (s, 2H), 7.48 (t, *J* = 7.8 Hz, 1H), 7.28 (d, *J* = 7.9 Hz, 2H), 7.23 (s, 4H), 7.18 (t, *J* = 7.5 Hz, 1H), 7.07 (d, *J* = 7.7 Hz, 2H), 6.91 (s, 4H), 6.85 (s, 2H), 3.70 (s, 3H), 3.06 – 2.80 (m, 10H), 2.44 (hept, *J* = 6.7 Hz, 2H), 2.17 (hept, *J* = 7.1 Hz, 4H), 1.33 – 1.27 (three overlapped doublets, 36H), 1.25 (d, *J* = 6.9 Hz, 12H), 1.21 (d, *J* = 6.7 Hz, 12H), 1.17 (d, *J* = 6.8 Hz, 12H), 1.05 (d, *J* = 6.8 Hz, 12H), 1.01 (d, *J* = 6.9 Hz, 12H).

^{13}C NMR (101 MHz, $CDCl_3$) δ 160.1, 157.5, 154.5, 147.2, 146.2, 145.6, 145.3, 144.8, 144.4, 139.6, 139.1, 139.0, 138.5, 131.9, 131.8, 131.2, 131.1, 127.3, 125.3, 123.7, 123.1, 122.1, 119.7, 43.5, 34.6, 34.0, 32.8, 31.8, 31.2, 30.1, 29.8, 25.7, 24.8, 24.8, 24.3, 24.1, 23.9, 23.8, 23.6.

3 ($C5^+A5^-$)

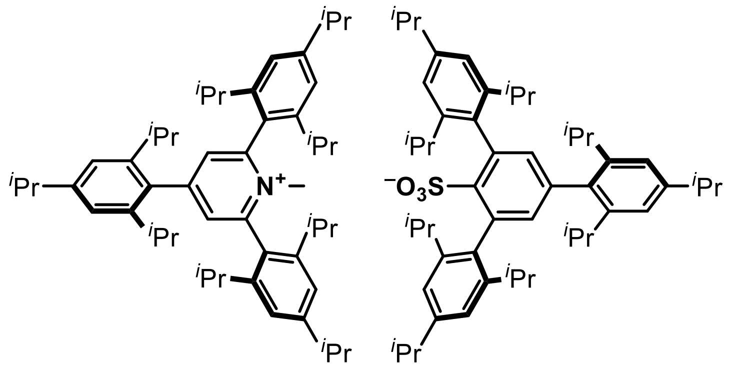

Prepared following the general procedure from $\mathbf{C5^+BF_4^-}$ (20 mg, 0.025 mmol) and $\mathbf{Na^+A5^-}$ (20 mg, 0.025 mmol). The ion pair was obtained as a yellowish crystalline solid (35 mg, 94%). Single crystals of **3** were obtained by a slow evaporation of a solution in hexane/benzene at room temperature.

^{1}H NMR (500 MHz, C_6D_6) δ 7.22 (s, 4H), 7.19 (s, 4H), 7.14 (s, 2H), 7.04 (s, 2H), 6.97 (s, 2H), 6.86 (s, 2H), 3.94 (s, 3H), 3.27 (hept, *J* = 6.9 Hz, 4H), 3.16 (hept, *J* = 6.9 Hz, 2H), 3.06 – 2.92 (m, 4H), 2.87 (hept, *J* = 6.9 Hz, 1H), 2.77 (hept, *J* = 6.8 Hz, 1H), 2.26 (hept, *J* = 6.7 Hz, 2H), 2.04 (hept, *J* = 6.7 Hz, 4H), 1.53 (d, *J* = 6.7 Hz, 12H), 1.46 (d, *J* = 6.9 Hz, 12H), 1.37 (d, *J* = 6.8 Hz, 12H), 1.33 – 1.29 (m, 30H), 1.23 (d, *J* = 6.8 Hz, 12H), 1.21 (d, *J* = 6.9 Hz, 6H), 0.99 (d, *J* = 6.8 Hz, 12H), 0.97 (d, *J* = 6.7 Hz, 12H).

^{13}C NMR (126 MHz, C_6D_6) δ 157.8, 157.6, 153.3, 151.2, 147.7, 147.3, 146.9, 145.7, 145.3, 145.3, 140.8, 139.7, 138.8, 137.3, 132.4, 131.1, 130.8, 126.5, 122.8, 121.2, 120.4, 119.5, 46.8, 34.9, 34.9, 34.9, 34.8, 32.8, 31.6, 31.4, 30.6, 26.0, 25.1, 24.9, 24.8, 24.5, 24.4, 24.2, 24.0, 23.6.

Additional NMR spectra available in section 3.1 NMR spectra: COSY, HSQC, HMBC, ROESY in C_6D_6; ^{1}H NMR, COSY, and ROESY in toluene-d_8; ^{1}H NMR, COSY, and ROESY in DMSO-d_6.

4 ($C6^+A6^-$)

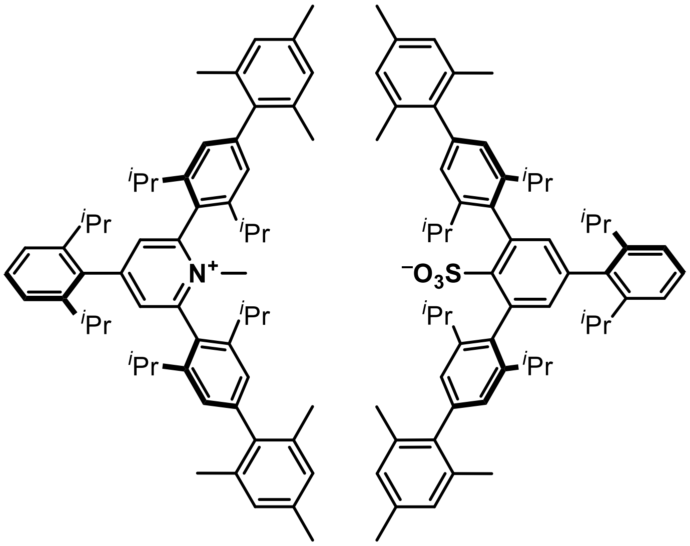

Prepared following the general procedure from $\mathbf{C6^+BF_4^-}$ (15.6 mg, 0.017 mmol) and $\mathbf{Na^+A6^-}$ (16 mg, 0.017 mmol). The ion pair was obtained as a yellowish crystalline solid (28.5 mg, 99%). Single crystals of **4** were obtained by slow evaporation of a solution in benzene/heptane at room temperature.

^{1}H NMR (400 MHz, $CDCl_3$) δ 8.02 (s, 2H), 7.51 (t, *J* = 7.9 Hz, 1H), 7.31 (d, *J* = 7.9 Hz, 2H), 7.27 (t, *J* = 7.6 Hz, 1H, overlapped), 7.24 (s, 4H), 7.15 (d, *J* = 7.6 Hz, 2H), 6.99 (s, 4H), 6.90 (s, 4H), 6.88 (s, 2H), 6.84 (s, 4H), 3.82 (s, 3H), 3.07 – 2.89 (m, 6H), 2.49 (hept, *J* =

6.9 Hz, 2H), 2.35 (s, 6H), 2.32 – 2.22 (m, 12H), 2.18 (s, 6H), 2.02 (s, 12H), 1.99 (s, 6H), 1.34 – 1.28 (two overlapped doublets, 24H), 1.25 – 1.19 (two overlapped doublets, 24H), 1.11 (d, *J* = 6.8 Hz, 12H), 1.01 (d, *J* = 6.9 Hz, 12H).

^{13}C NMR (101 MHz, $CDCl_3$) δ 160.8, 157.3, 147.4, 146.6, 146.6, 146.0, 144.8, 141.3, 140.0, 139.6, 139.3, 138.2, 138.0, 137.7, 137.4, 137.4, 136.5, 135.5, 135.4, 135.3, 131.9, 131.7, 131.4, 131.3, 128.7, 128.7, 128.0, 127.7, 127.5, 126.4, 125.8, 123.8, 122.7, 122.5, 43.6, 32.9, 32.0, 31.2, 30.2, 25.6, 24.9, 24.8, 24.1, 23.8, 23.5, 21.6, 21.1, 21.0, 20.9, 20.9.

5 ($C7^+A7^-$)

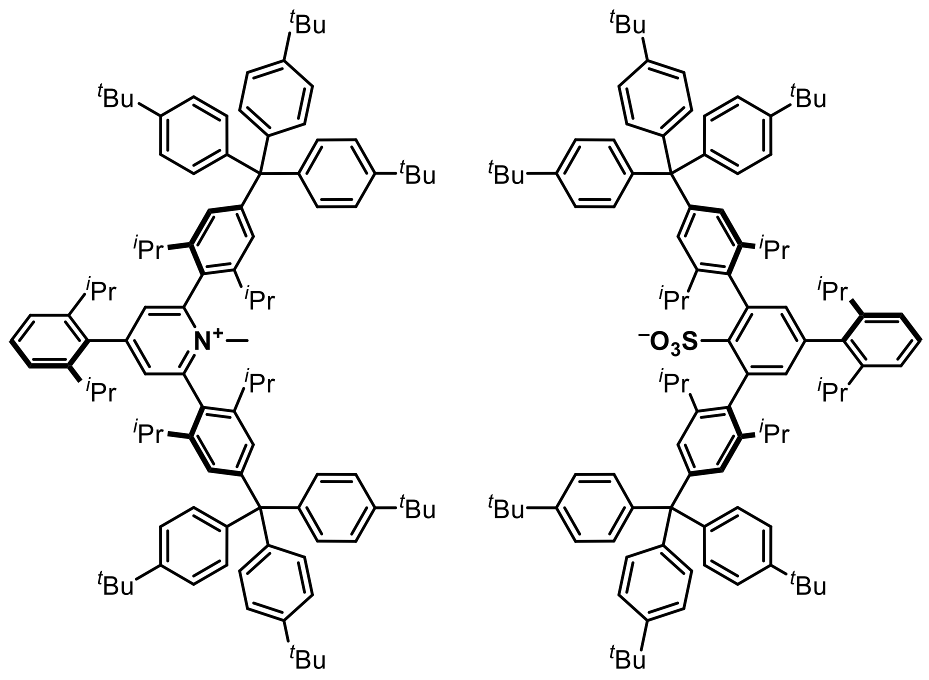

Prepared following the general procedure from **$C7^+BF_4^-$** (18.7 mg, 0.0126 mmol) and **Na^+A7^-** (18.7 mg, 0.0126 mmol). The ion pair was obtained as a yellowish crystalline solid (33.8 mg, 94%). Single crystals of **5** were obtained by slow evaporation of a solution in hexane at room temperature.

^{1}H NMR (500 MHz, $CDCl_3$) δ 7.94 (s, 2H), 7.49 (t, *J* = 7.9 Hz, 1H), 7.32 – 7.25 (m, 14H), 7.23 (s, 4H), 7.23 – 7.17 (m, 25H), 7.14 (d, *J* = 7.7 Hz, 2H), 7.06 (d, *J* = 8.6 Hz, 12H), 6.83 (s, 2H), 6.77 (s, 4H), 3.73 (s, 3H), 2.93 – 2.80 (m, 6H), 2.42 (hept, *J* = 6.8 Hz, 2H), 2.14 (hept, *J* = 6.9 Hz, 4H), 1.31 (s, 54H), 1.28 (s, 54H), 1.18 (d, *J* = 6.8 Hz, 12H), 1.13 (d, *J* = 6.5 Hz, 12H), 1.10 (d, *J* = 6.8 Hz, 12H), 1.08 – 1.03 (two overlapped doublets, 24H), 0.81 (d, *J* = 6.9 Hz, 12H).

^{13}C NMR (126 MHz, $CDCl_3$) δ 160.9, 157.3, 153.4, 149.4, 147.7, 147.4, 145.1, 144.9, 144.7, 144.2, 142.8, 139.8, 139.4, 138.9, 138.5, 131.9, 131.6, 131.5, 131.3, 131.2, 130.6, 128.2, 127.5, 125.2, 124.8, 124.6, 123.9, 123.8, 122.5, 64.3, 64.1, 43.2, 34.5, 34.3, 32.7, 32.0, 31.6, 31.5, 31.1, 30.2, 29.8, 25.3, 24.8, 24.5, 24.0, 23.4, 23.3.

6 (C7$^+$A4$^-$)

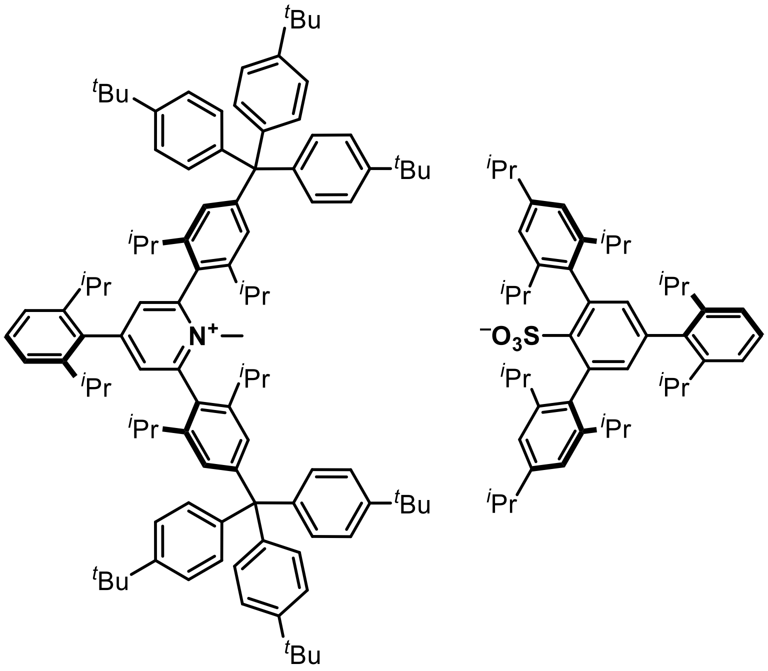

Prepared following the general procedure from **C7$^+$BF$_4^-$** (18.7 mg, 0.0126 mmol) and **Na$^+$A4$^-$** (9.4 mg, 0.0126 mmol). The ion pair was obtained as a yellowish crystalline solid (25.2 mg, 94%). Single crystals of **6** were obtained by leaving a solution in hot hexane at room temperature overnight.

^{1}H NMR (400 MHz, $CDCl_3$) δ 7.91 (s, 2H), 7.49 (t, *J* = 7.8 Hz, 1H), 7.31 – 7.26 (m, 14H), 7.21 (s, 4H), 7.18 (t, *J* = 7.7 Hz, 2H), 7.08 – 7.03 (m, 14H), 6.93 (s, 4H), 6.86 (s, 2H), 3.72 (s, 3H), 3.01 – 2.81 (m, 8H), 2.41 (hept, *J* = 6.8 Hz, 2H), 2.19 – 2.08 (m, 8H), 1.35 (d, *J* = 6.7 Hz, 12H), 1.31 (s, 54H), 1.26 (d, *J* = 6.9 Hz, 12H), 1.17 (d, *J* = 6.8 Hz, 12H), 1.09 (d, *J* = 6.8 Hz, 12H), 1.07 – 1.01 (three overlapped doublets, 36H).

^{13}C NMR (101 MHz, $CDCl_3$) δ 160.7, 157.3, 153.3, 149.4, 147.3, 146.3, 145.4, 144.7, 144.2, 142.8, 139.7, 139.2, 139.1, 138.5, 131.9, 131.7, 131.4, 131.2, 130.7, 128.2, 127.3, 124.8, 124.5, 123.8, 122.1, 119.7, 64.3, 43.3, 34.5, 34.1, 32.7, 31.9, 31.5, 31.2, 30.2, 29.8, 25.7, 24.8, 24.5, 24.3, 24.0, 23.8, 23.3.

7 ($C8^{2+}(A4^{-})_2$)

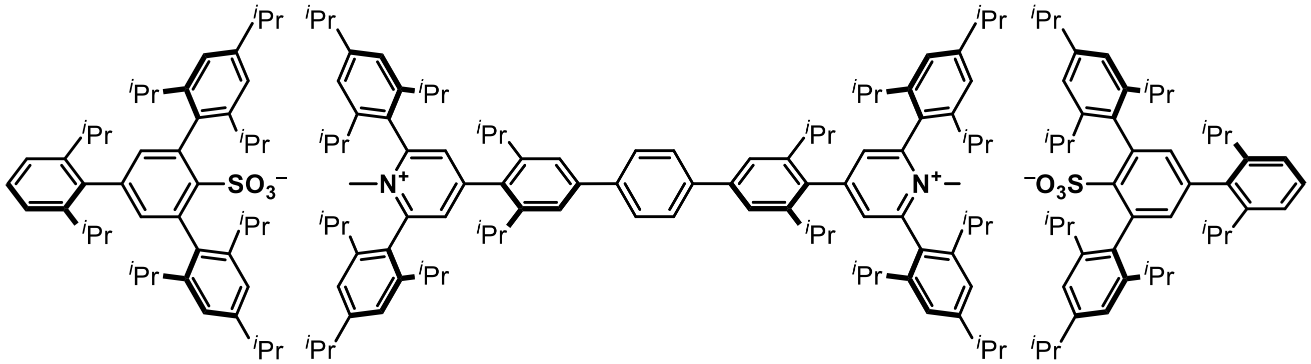

Prepared following a modified general procedure from **$C8^{2+}(BF_4^{-})_2$** (9.5 mg, 0.0061 mmol) and **$Na^{+}A4^{-}$** (9.05 mg, 0.0121 mmol) in the mixture of CH_2Cl_2/*i*PrOH 3:1. The ion pair was obtained as a yellowish crystalline solid (14.3 mg, 83%). Single crystals of **7** were obtained by slow evaporation of a solution in mesitylene/n-hexane/n-pentane/benzene.

^{1}H NMR (500 MHz, $C_6D_6/CDCl_3$ 9:1) δ 7.75 (s, 4H), 7.54 (s, 4H), 7.22 (t, *J* = 7.7 Hz, 2H), 7.14 (s, 4H), 7.11 – 7.09 (m, 12H), 6.93 (s, 4H), 3.77 (s, 6H), 3.19 (hept, *J* = 6.9 Hz, 8H), 3.06 (hept, *J* = 6.4 Hz, 4H), 2.95 (hept, *J* = 14.1, 7.1 Hz, 4H), 2.88 (hept, *J* = 6.9 Hz, 4H), 2.35 (hept, *J* = 7.0 Hz, 4H), 2.06 (hept, *J* = 6.6 Hz, 8H), 1.45 (d, *J* = 6.7 Hz, 24H), 1.38 (d, *J* = 6.9 Hz, 24H), 1.28 (d, *J* = 6.9 Hz, 24H), 1.23 (d, *J* = 6.8 Hz, 24H, overlapped), 1.22 (d, *J* = 6.9 Hz, 24H, overlapped), 1.14 (d, *J* = 6.9 Hz, 24H), 1.09 (d, *J* = 6.8 Hz, 24H), 1.02 (d, *J* = 6.7 Hz, 24H). One signal (s, 8H) is overlapped with the residual solvent peak at 7.16 ppm.

^{13}C NMR (126 MHz, $C_6D_6/CDCl_3$ 9:1) δ 158.0, 157.7, 153.7, 147.3, 146.8, 146.2, 146.0, 145.5, 143.3, 141.0, 140.4, 139.8, 139.4, 138.8, 132.4, 132.2, 131.0, 126.3, 123.0, 122.6, 122.4, 119.8, 77.4, 45.6, 34.8, 34.8, 32.8, 31.7, 31.6, 30.5, 30.2, 26.0, 25.1, 25.0, 24.8, 24.3, 24.2, 24.1, 23.7.

Additional NMR spectra available in section 3.1 NMR spectra: COSY, ROESY in $C_6D_6/CDCl_3$.

8 ($C9^{2+}(A4^{-})_2$)

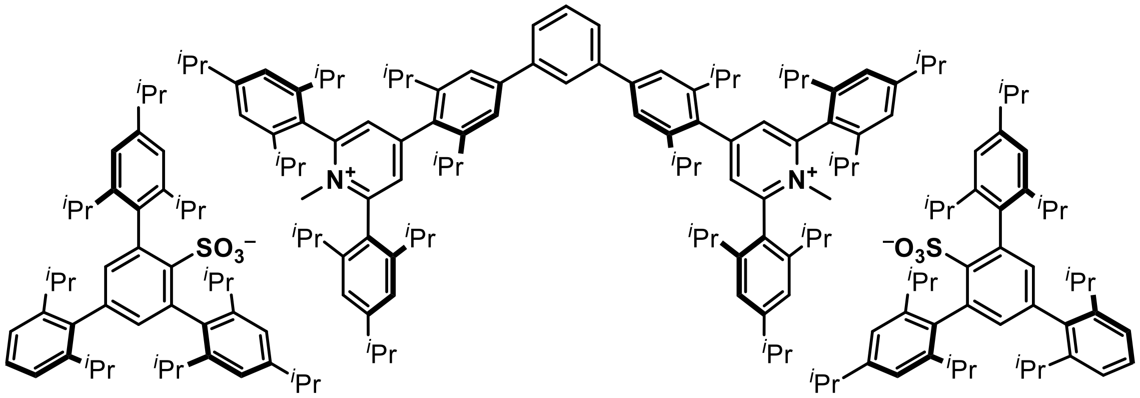

Prepared following the general procedure from **$C9^{2+}(BF_4^{-})_2$** (9.2 mg, 0.0059 mmol) and **$Na^{+}A4^{-}$** (8.8 mg, 0.0118 mmol). The ion pair was obtained as a yellowish crystalline solid (14.2 mg, 85%).

^{1}H NMR (500 MHz, C_6D_6) δ 8.13 (t, *J* = 2.0 Hz, 1H), 7.70 (dd, *J* = 7.7, 1.8 Hz, 2H), 7.60 (s, 4H), 7.50 (t, *J* = 7.7 Hz, 1H), 7.28 (t, *J* = 7.7 Hz, 2H), 7.20 (s, 8H), 7.19 (s, 8H), 7.18 (s, 2H), 7.05 (s, 4H), 6.99 (s, 4H), 3.74 (s, 6H), 3.30 (hept, *J* = 6.8 Hz, 8H), 3.17 (hept, *J* = 6.8 Hz, 4H), 3.03 (hept, *J* = 6.9 Hz, 4H), 2.92 (hept, *J* = 6.8 Hz, 4H), 2.37 (hept, *J* = 6.8 Hz, 4H), 2.05 (hept, *J* = 6.8 Hz, 8H), 1.55 (d, *J* = 6.7 Hz, 24H), 1.46 (d, *J* = 6.9 Hz, 24H), 1.33 (d, *J* = 6.9 Hz, 48H), 1.27 (d, *J* = 6.8 Hz, 24H), 1.20 (d, *J* = 6.8 Hz, 24H), 1.07 (d, *J* = 6.7 Hz, 24H), 1.04 (d, *J* = 6.7 Hz, 24H).

^{13}C NMR (126 MHz, C_6D_6) δ 157.8, 157.5, 153.4, 147.4, 146.8, 146.6, 146.4, 145.9, 145.7, 145.1, 143.7, 142.8, 140.9, 139.7, 139.7, 138.4, 132.8, 132.1, 130.8, 128.6, 127.2, 127.1, 126.3, 122.8, 122.6, 122.6, 119.4, 46.2, 34.9, 34.8, 32.8, 31.6, 31.6, -30.5, 26.0, 25.0, 24.9, 24.9, 24.5, 24.1, 24.0, 23.6.

Additional NMR spectra available in section 3.1 NMR spectra: COSY and ROESY in C_6D_6.

9 ($C8^{2+}(A9^-)_2$)

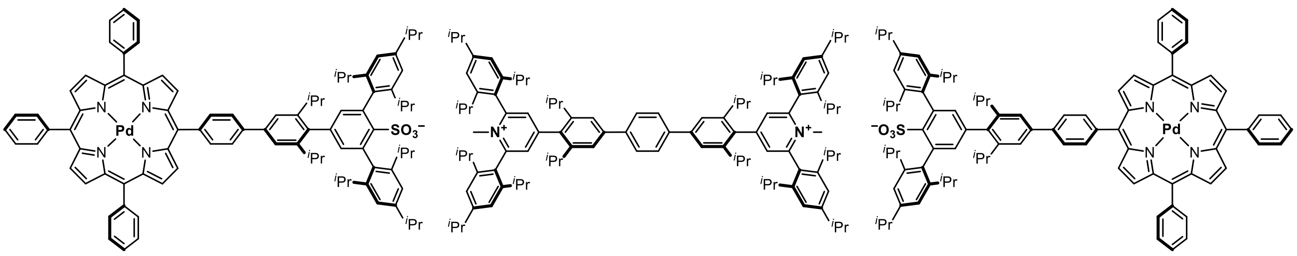

Prepared following the general procedure from **$C8^{2+}(BF_4^-)_2$** (4.1 mg, 0.0026 mmol) and **Na^+A9^-** (7.7 mg, 0.0052 mmol). The ion pair was obtained as dark-red crystals (7.5 mg, 67%). Partial loss of around 20% of Pd was observed by ^{1}H NMR analysis. Single crystals of **9** were obtained from a solution in toluene/*n*-heptane at room temperature.

^{1}H NMR (500 MHz, C_6D_6) δ 9.12 (d, *J* = 5.0 Hz, 4H), 8.98 (d, *J* = 4.9 Hz, 4H), 8.94 – 8.92 (m, 8H), 8.29 (d, *J* = 8.2 Hz, $4H^{free}$), 8.21 (d, *J* = 8.2 Hz, $4H^{Pd}$), 8.14 – 8.11 (m, $12H^{free}$), 8.07 – 8.02 (m, $16H^{Pd}$ + $4H^{free}$), 7.88 (s, $4H^{free}$), 7.86 (s, $4H^{Pd}$), 7.84 (s, 4H), 7.63 (s, 4H), 7.49 – 7.36 (m, 18H), 7.27 (s, 8H), 7.25 (s, 8H), 7.17 (assigned as s, 4H, overlapped with the residual solvent signal), 7.08 (s, 4H), 3.83 (s, 6H), 3.43 – 3.33 (m, 12H), 3.06 (hept, *J* = 6.9 Hz, 4H), 2.96 (hept, *J* = 6.9 Hz, 4H), 2.41 (hept, *J* = 6.7 Hz, 4H), 2.13 – 2.06 (m, 8H, overlapped with a signal from residual toluene), 1.62 (d, *J* = 6.6 Hz, 24H), 1.50 (d, *J* = 6.8 Hz, 24H), 1.43 (d, *J* = 6.9 Hz, 24H), 1.41 – 1.34 (two overlapped doublets, 48H, overlapped with residual heptane), 1.31 (d, *J* = 6.8 Hz, 24H), 1.12 (d, *J* = 6.8 Hz, 24H), 1.08 (d, *J* = 6.8 Hz, 24H), -2.06 (s, $2H^{free}$, N-*H*). The signals from the product and the side-product in which Pd is not present in the porphyrin are marked as H^{Pd} and H^{free}, respectively, where separable.

^{13}C NMR (126 MHz, C_6D_6) δ 158.1, 157.3, 153.6, 148.3, 147.0, 146.3, 145.9, 143.2, 143.0, 142.53, 142.46, 142.42, 142.36, 142.3, 140.0, 137.9, 135.6, 135.2, 135.0, 134.5, 132.8, 132.5, 131.8, 131.6, 131.5, 130.8, 129.3, 128.6, 127.5, 127.0, 126.4, 126.0, 125.7, 122.9, 122.6, 122.5, 122.4, 122.3, 122.0, 119.8, 53.3, 35.7, 34.9, 34.9, 32.9, 32.3, 31.7, 26.1, 25.1, 25.0, 24.8, 24.4, 24.2, 24.1, 23.7. Contains signals belonging to residual heptane and toluene.

10 ($C10^{3+}A10^{3-}$)

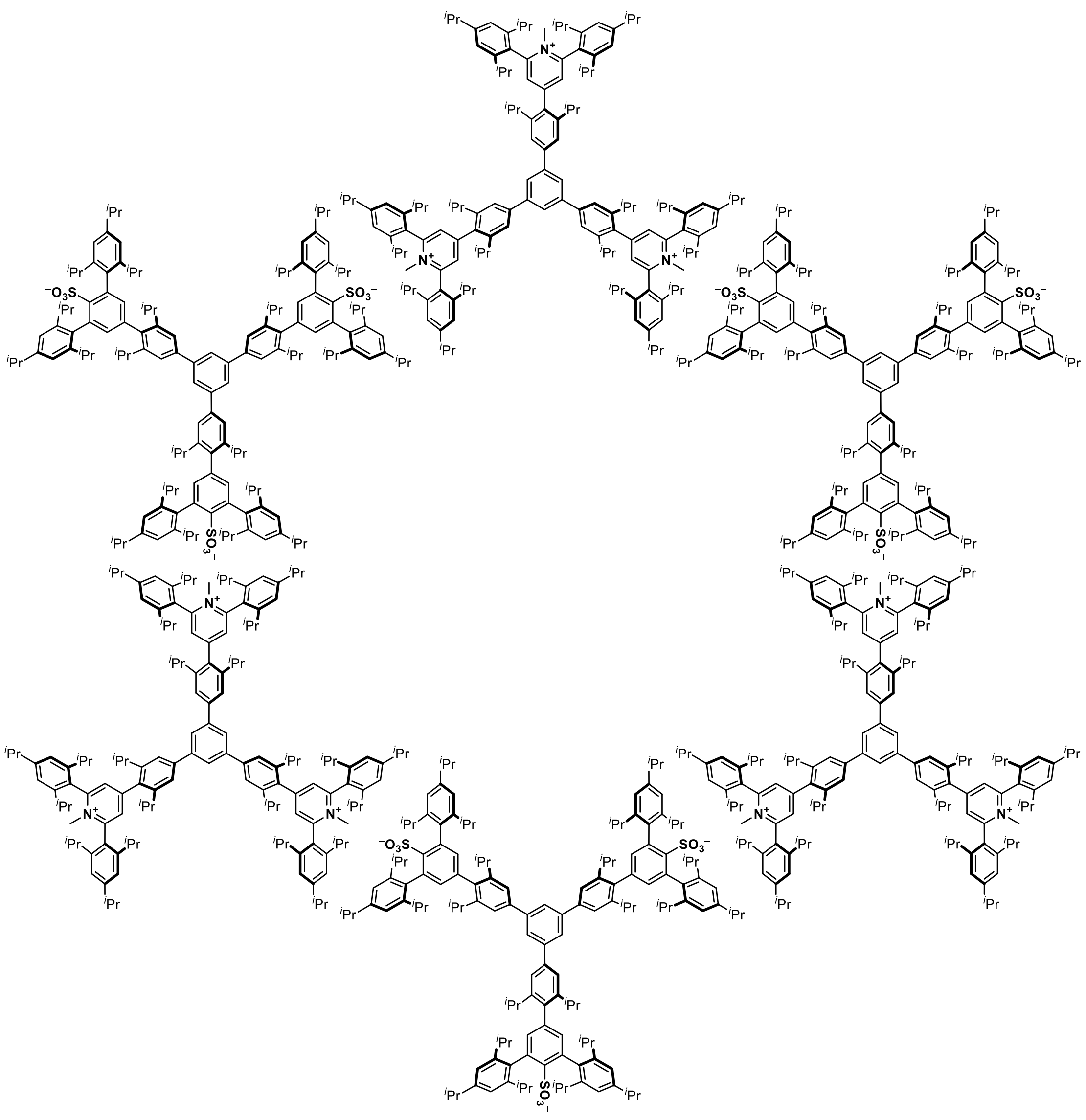

Prepared following a modified general procedure from **$C10^{3+}(BF_4^-)_3$** (9 mg, 0.0039 mmol) and **$(Na^+)_3A10^{3-}$** (9 mg, 0.0039 mmol) in CH_2Cl_2/*i*PrOH 4:1 (5 mL). The ion pair was obtained as an off-white solid (15 mg, 90%). Single crystals of **10** were obtained by slow evaporation of a solution in CH_2Cl_2/$PhCF_3$.

^{1}H NMR (500 MHz, MeOD-$CDCl_3$) δ 7.89 (s, 6H), 7.63 (s, 3H), 7.62 (s, 3H), 7.50 (s, 6H), 7.34 (s, 6H), 7.25 (s, 12H), 6.89 (s, 18H), 3.68 (s, 9H), 2.98 (hept, *J* = 6.9 Hz, 6H), 2.82 (m, 24H), 2.54 (hept, *J* = 6.6 Hz, 6H), 2.16 (hept, *J* = 6.6 Hz, 12H), 1.29 – 1.26 (m, *J* = 6.5 Hz, 72H), 1.23 – 1.18 (m, 144H), 1.04 (d, *J* = 6.8 Hz, 36H), 0.96 (d, *J* = 6.9 Hz, 36H). Contains residual ^{i}PrOH.

^{13}C NMR (126 MHz, MeOD-$CDCl_3$) δ 159.7, 157.8, 154.8, 147.7, 147.6, 146.6, 146.5, 146.1, 145.7, 143.9, 143.3, 142.7, 141.7, 141.1, 140.0, 138.2, 137.8, 132.5, 131.9, 131.4, 126.3, 125.5, 125.3, 123.2, 123.1, 122.0, 119.9, 64.1, 43.5, 34.7, 34.4, 33.0, 32.0, 31.3, 30.5, 25.6, 24.9, 24.8, 24.2, 24.1, 23.9, 23.4, 23.2. Contains residual ^{i}PrOH.

2.9 Analysis of the directionality of the ionic bonds in solution

2.9.1 Analysis of C3⁺A3⁻ by ROESY

^{1}H NMR (C_6D_6) spectrum of **C3⁺A3⁻** with assignments:

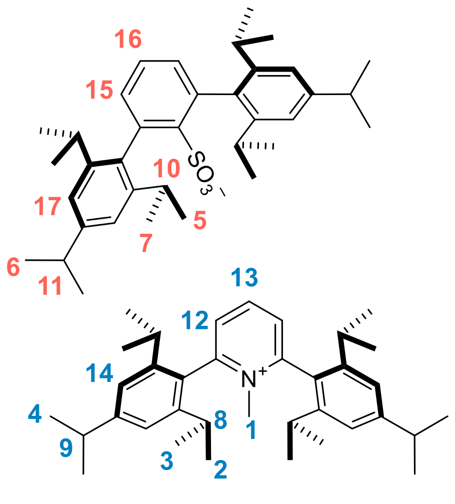

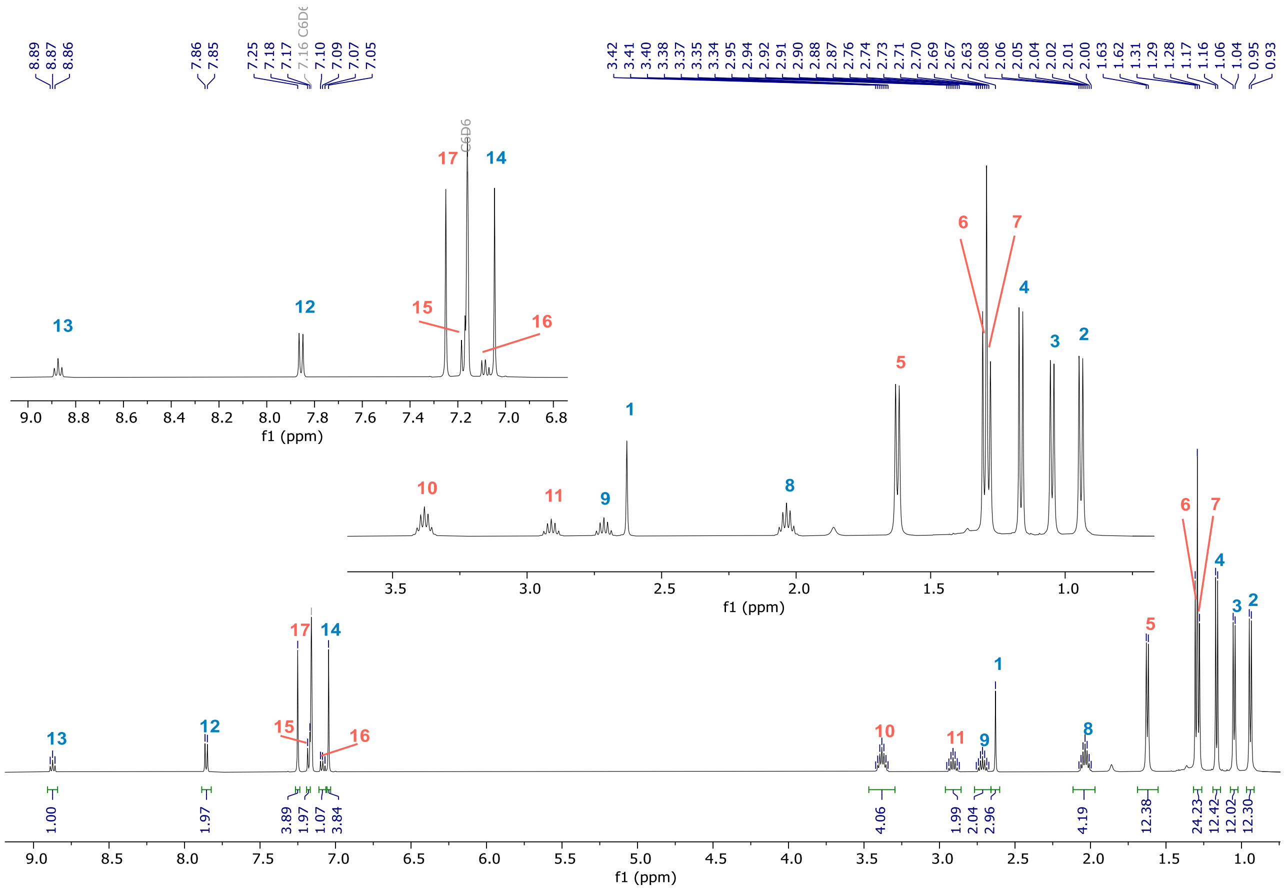

Excerpts from the ^{1}H-^{1}H ROESY NMR spectrum of **C3⁺A3⁻** in C_6D_6:

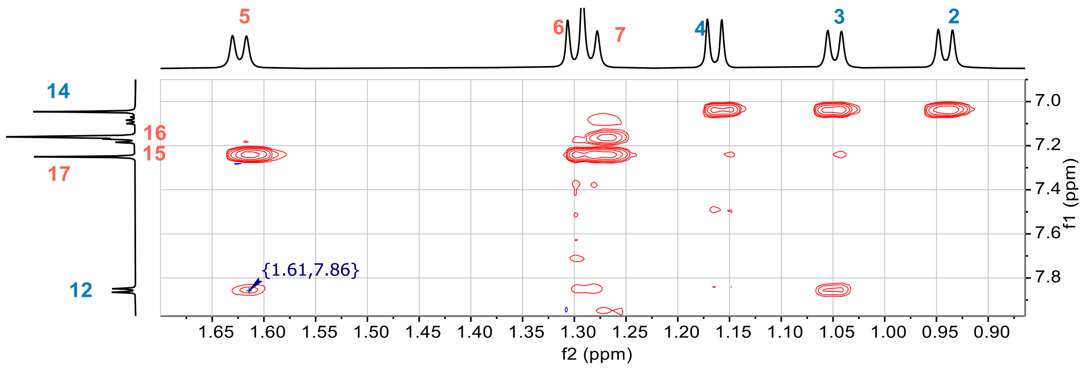

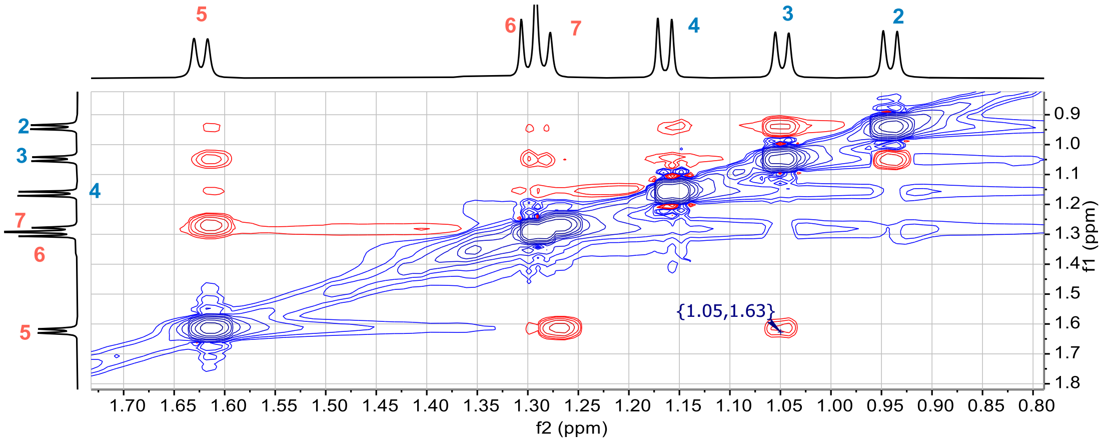

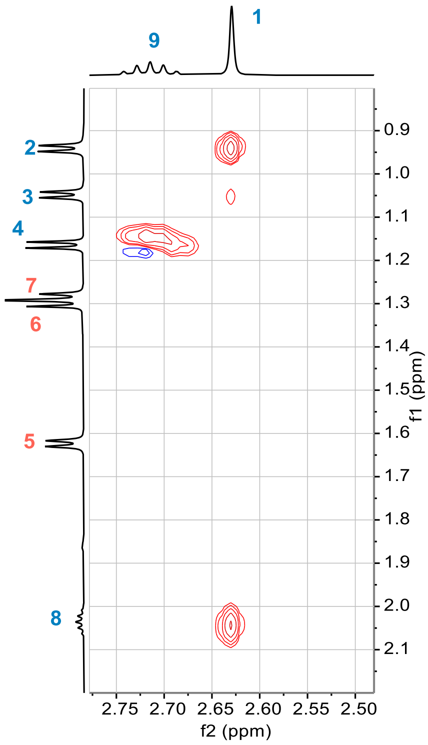

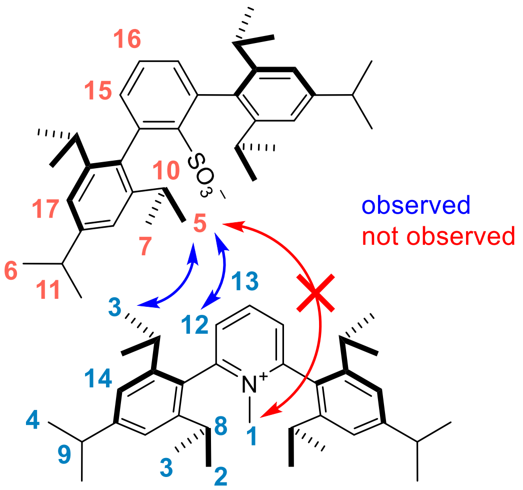

a

Comparison of the directionality in **C3⁺A3⁻** in solution in C_6D_6 and in the solid-state:

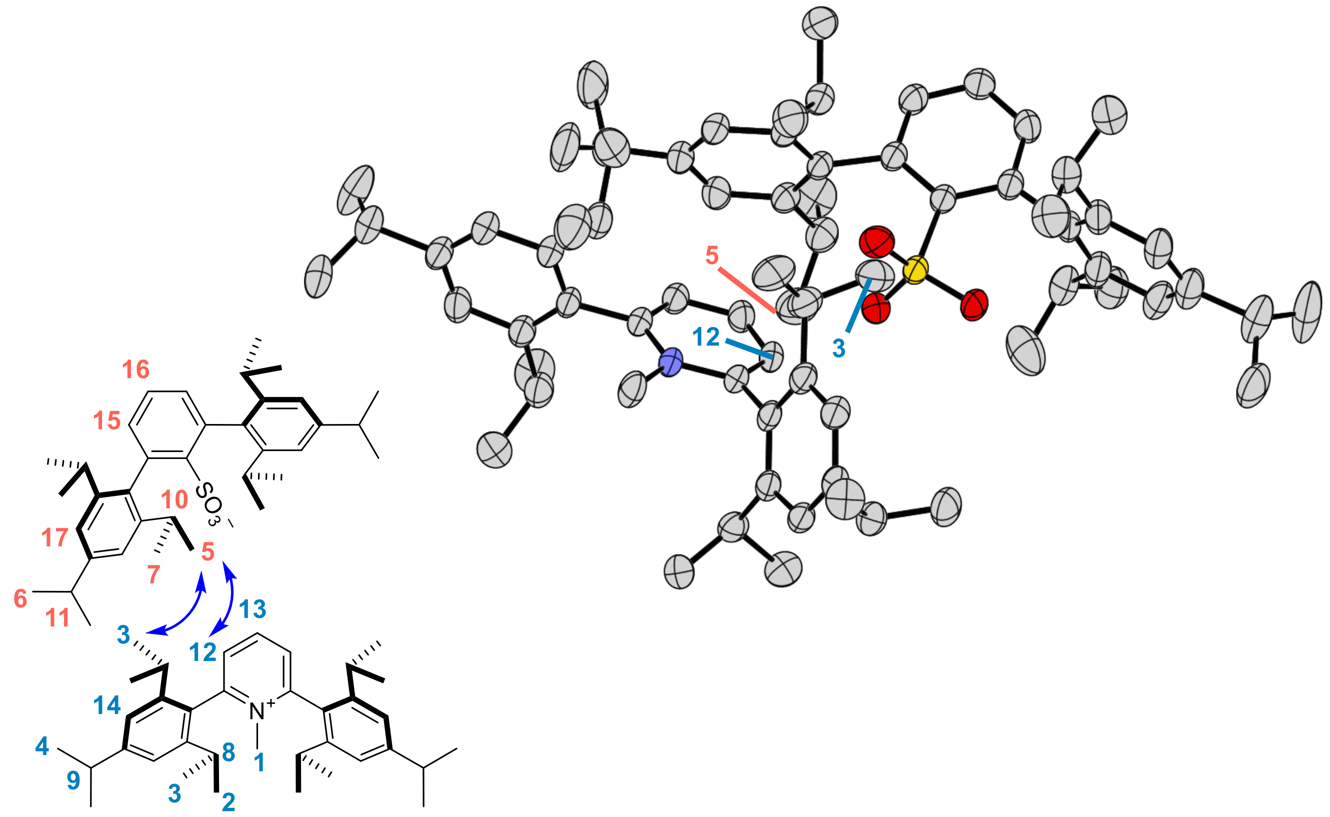

2.9.2 Analysis of 3 by ROESY

^{1}H NMR (C_6D_6) spectrum of **3** with assignments:

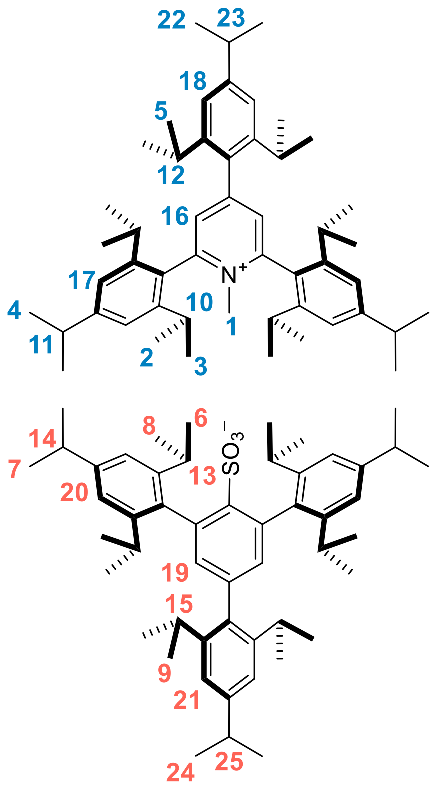

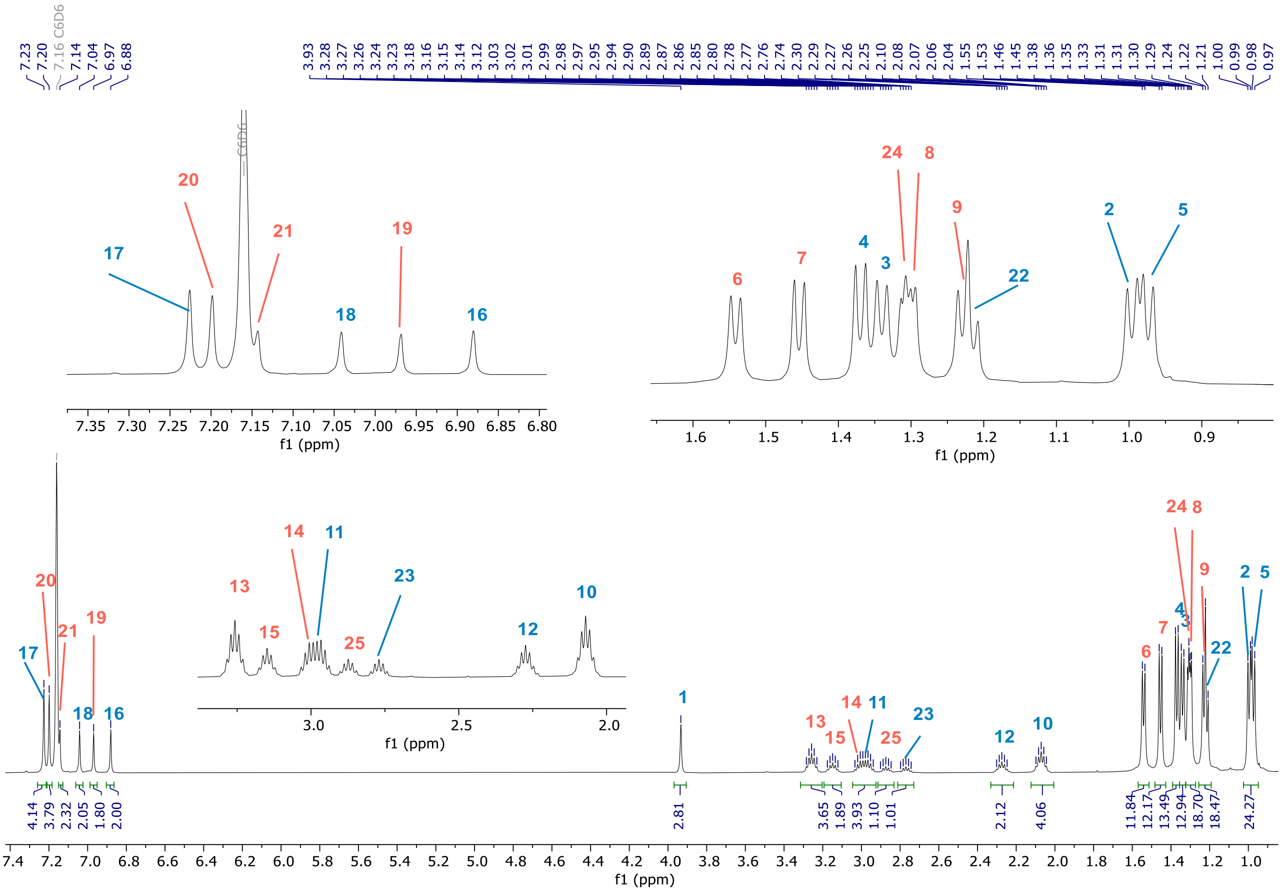

Excerpts from the ^{1}H-^{1}H ROESY NMR spectrum of **3** in C_6D_6:

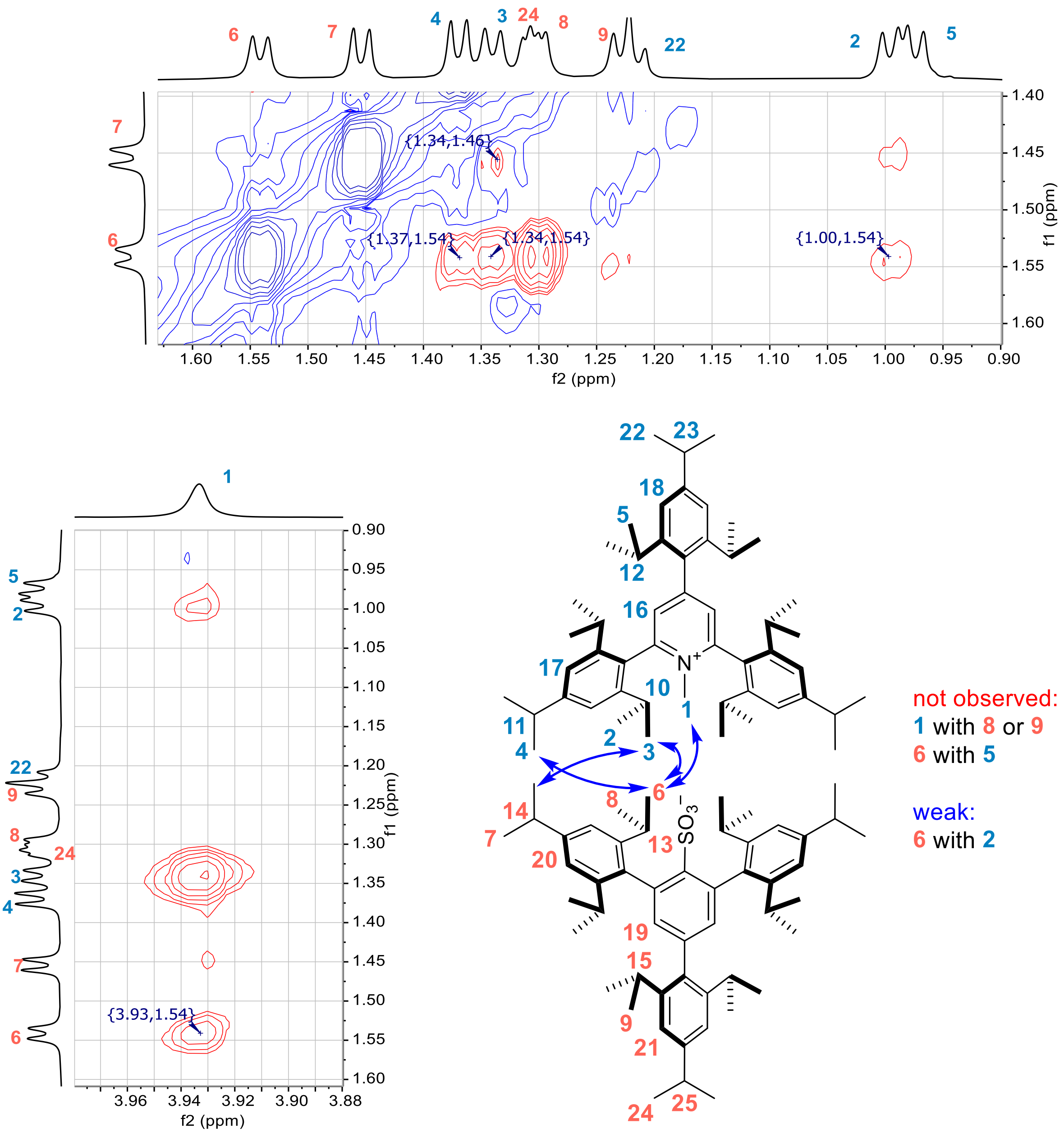

Comparison of the directionality in **3** in solution in C_6D_6 and in the solid-state:

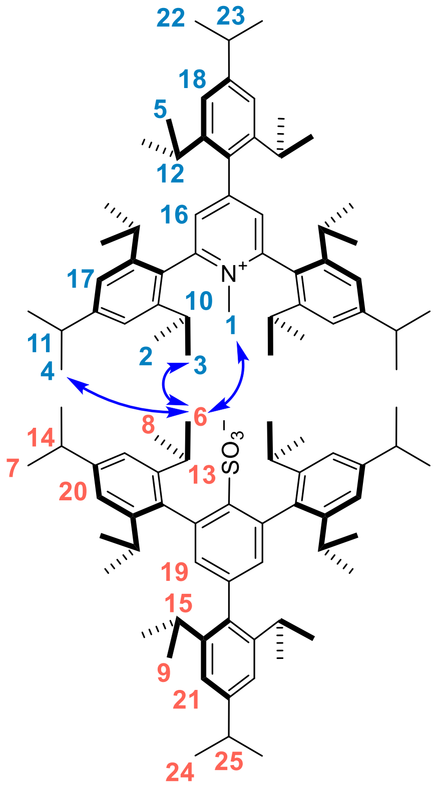

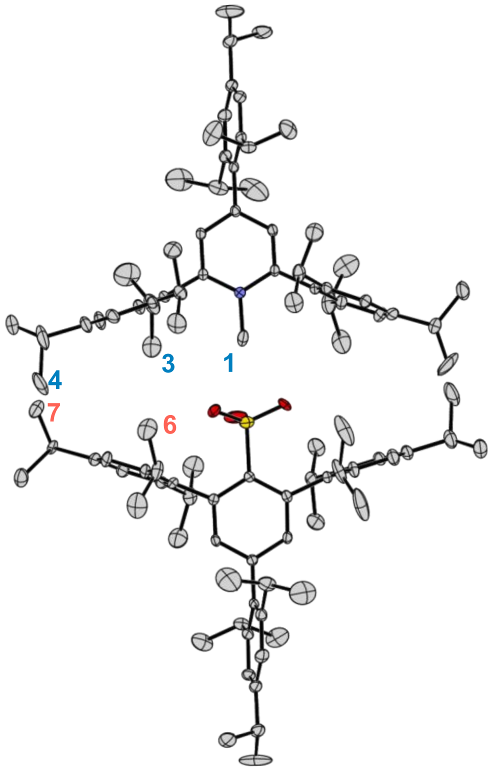

Selected ROE correlations and the corresponding distances, calculated from the integral intensities in **3** in C_6D_6:

Correlation	**F1, ppm**	**F2, ppm**	**Distance, Å**	**Closest distance in X-ray, Å**
1-3*	1.34	3.93	2.57	2.57
1-6	1.54	3.93	3.47	2.68
2-6	0.99	1.53	3.30	5.28
2-7	0.99	1.45	3.45	3.71
3-6	1.34	1.54	2.35	2.96
4-6	1.37	1.54	2.64	2.23
3-7	1.34	1.45	3.38	2.50
6-17	1.54	7.24	3.02	3.88
7-17	1.45	7.24	3.34	2.65
6-22	1.54	1.22	–	12.39

*The distance between the two closest protons was taken from the crystal structure of **3** and used as a reference.

Correlation	**F1, ppm**	**F2, ppm**	**Distance, Å**	**Closest distance in X-ray, Å**
1-10*	3.93	2.07	2.42	2.42
1-6	1.54	3.93	3.25	2.68
2-6	0.99	1.53	3.22	5.28
2-7	0.99	1.45	3.37	3.71
3-6	1.34	1.54	2.28	2.96
4-6	1.37	1.54	2.55	2.23
3-7	1.34	1.45	3.28	2.50
6-17	1.54	7.24	2.96	3.88
7-17	1.45	7.24	3.27	2.65
6-22	1.54	1.22	–	12.39

*The distance between the two closest protons was taken from the crystal structure of **3** and used as a reference.

Correlation	**F1, ppm**	**F2, ppm**	**Distance, Å**	**Closest distance in X-ray, Å**
10-12*	2.07	2.27	2.95	2.95
1-6	1.54	3.93	3.70	2.68
2-6	0.99	1.53	3.67	5.28
2-7	0.99	1.45	3.83	3.71
3-6	1.34	1.54	2.59	2.96
4-6	1.37	1.54	2.90	2.23
3-7	1.34	1.45	3.72	2.50
6-17	1.54	7.24	3.37	3.88
7-17	1.45	7.24	3.72	2.65
6-22	1.54	1.22	–	12.39

*The distance between the two closest protons was taken from the crystal structure of **3** and used as a reference.

^{1}H NMR (toluene-d_8) spectrum of **3** with assignments:

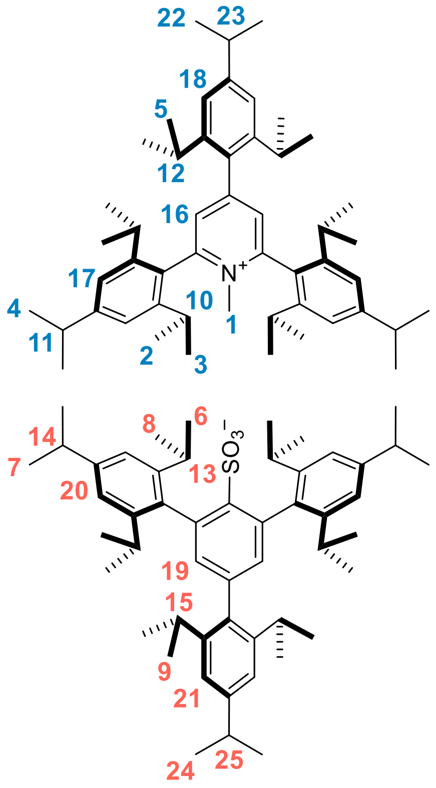

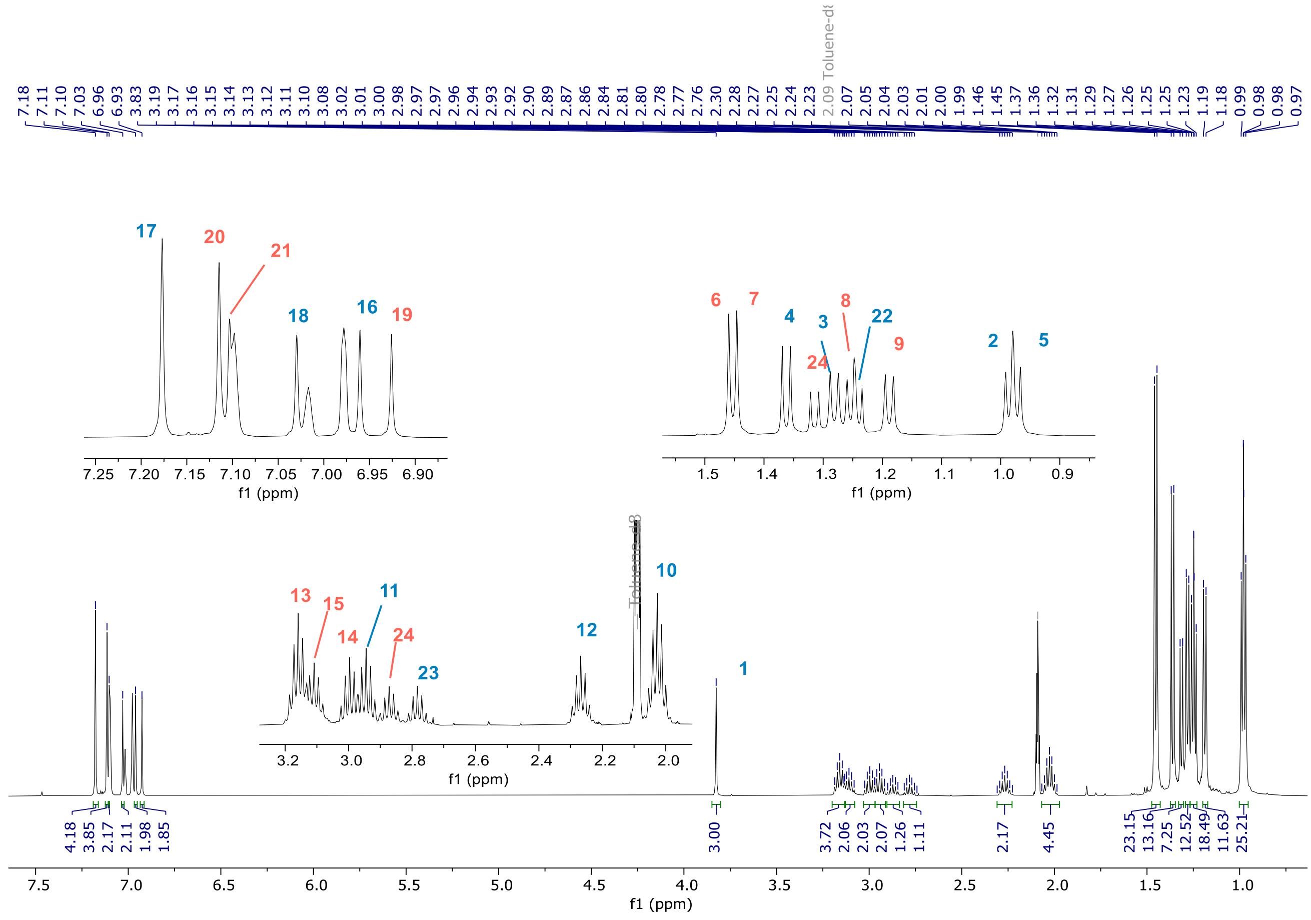

Excerpts from the ^{1}H-^{1}H ROESY NMR spectrum of **3** in toluene-d_8:

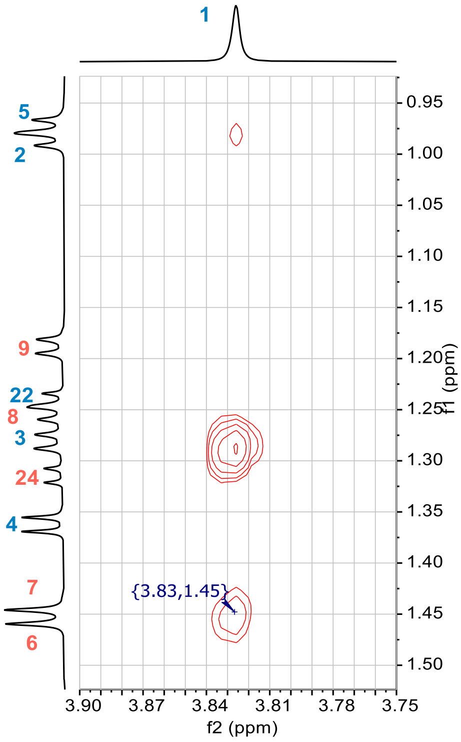

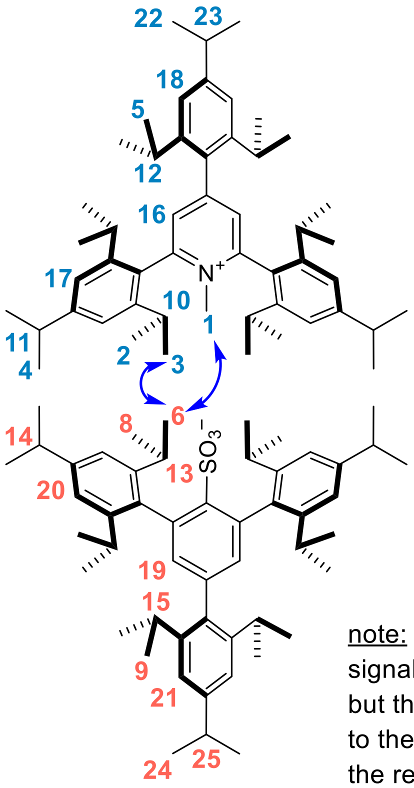

not observed:
1 with **8** or **9**

weak:
6/7 with **5** or **2**

note:
signals of **6** and **7** are overlapped, but the ROE signals are assigned to the interactions with **6**, based on the results of the analysis in C_6D_6

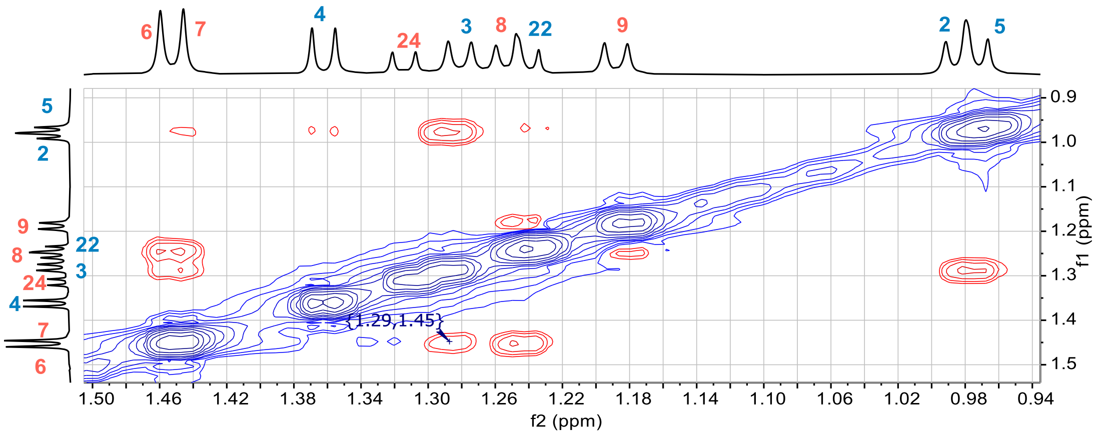

^{1}H NMR (DMSO-d_6) spectrum of **3** with assignments:

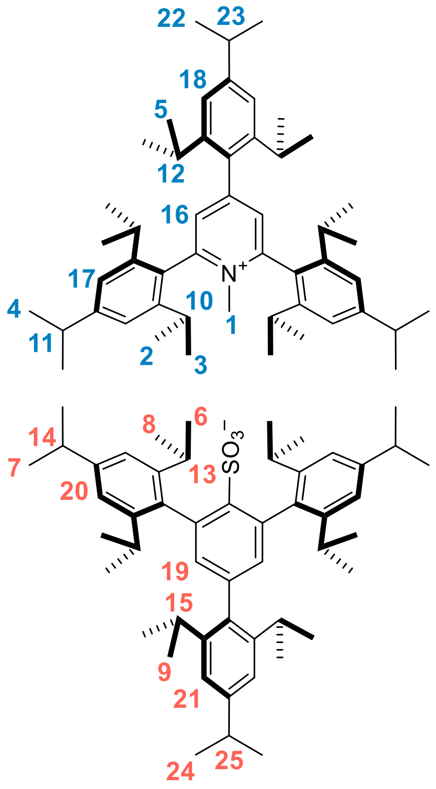

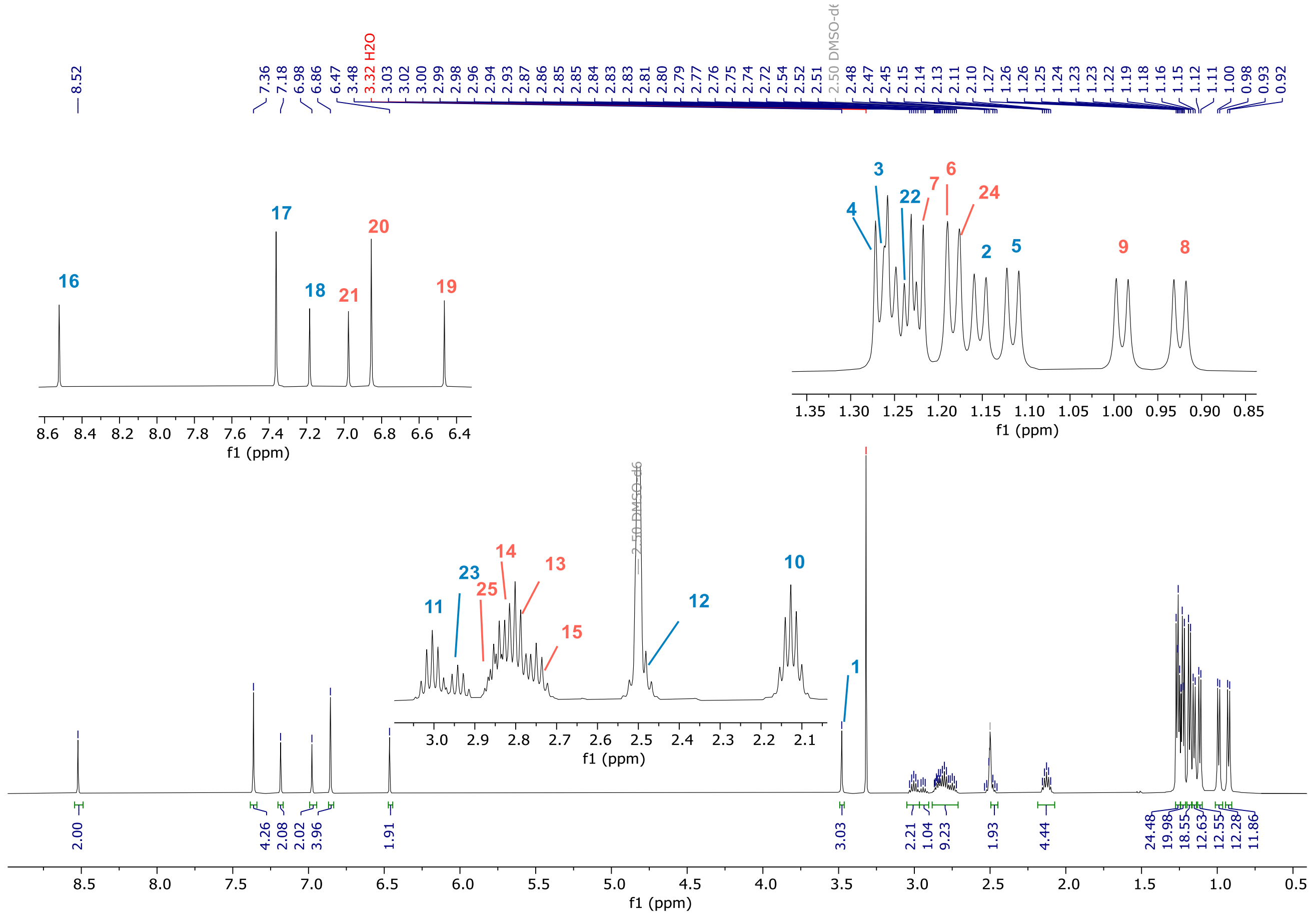

Excerpts from the ^1H-^1H ROESY NMR spectrum of **3** in DMSO-d_6:

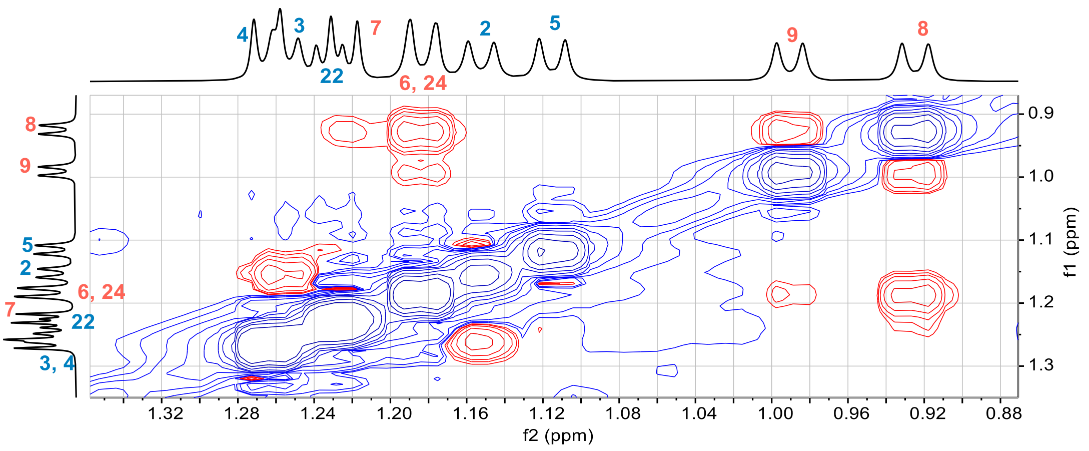

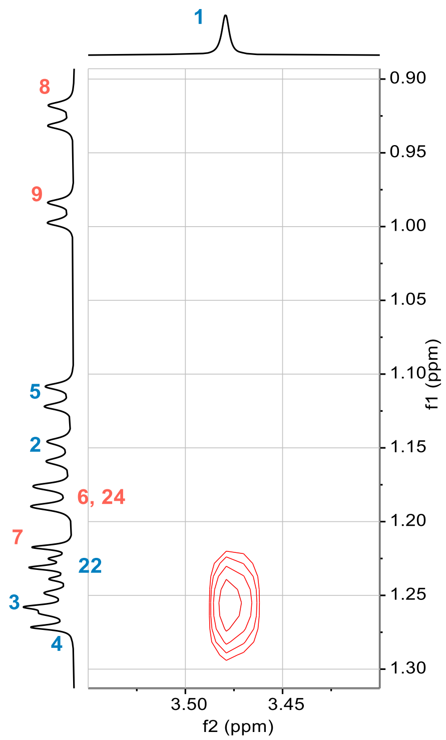

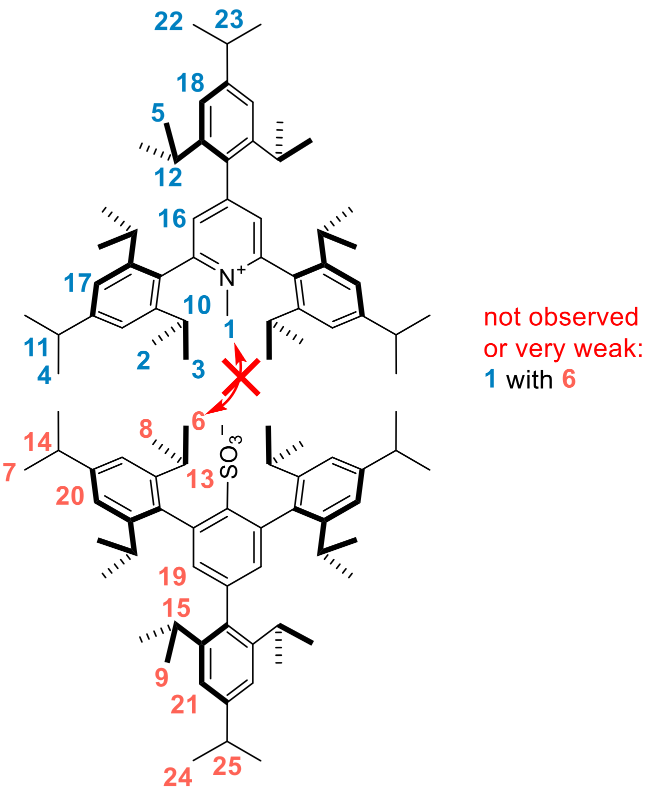

2.9.3 Analysis of 7 by ROESY

^{1}H NMR (C_6D_6/$CDCl_3$ 9:1) spectrum of **7** with assignments:

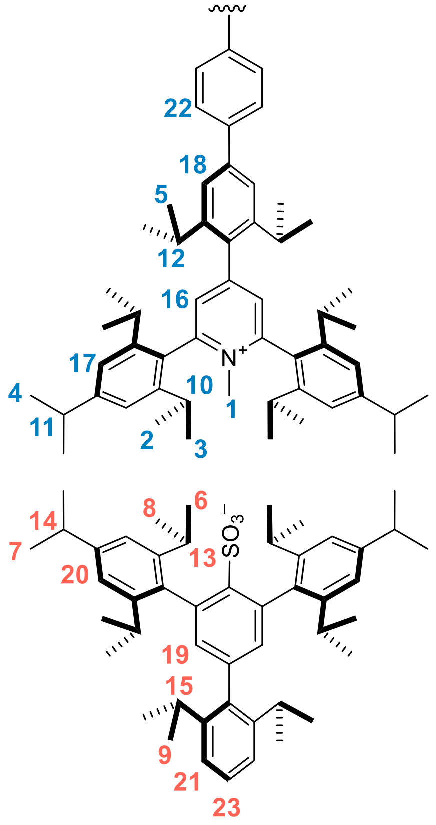

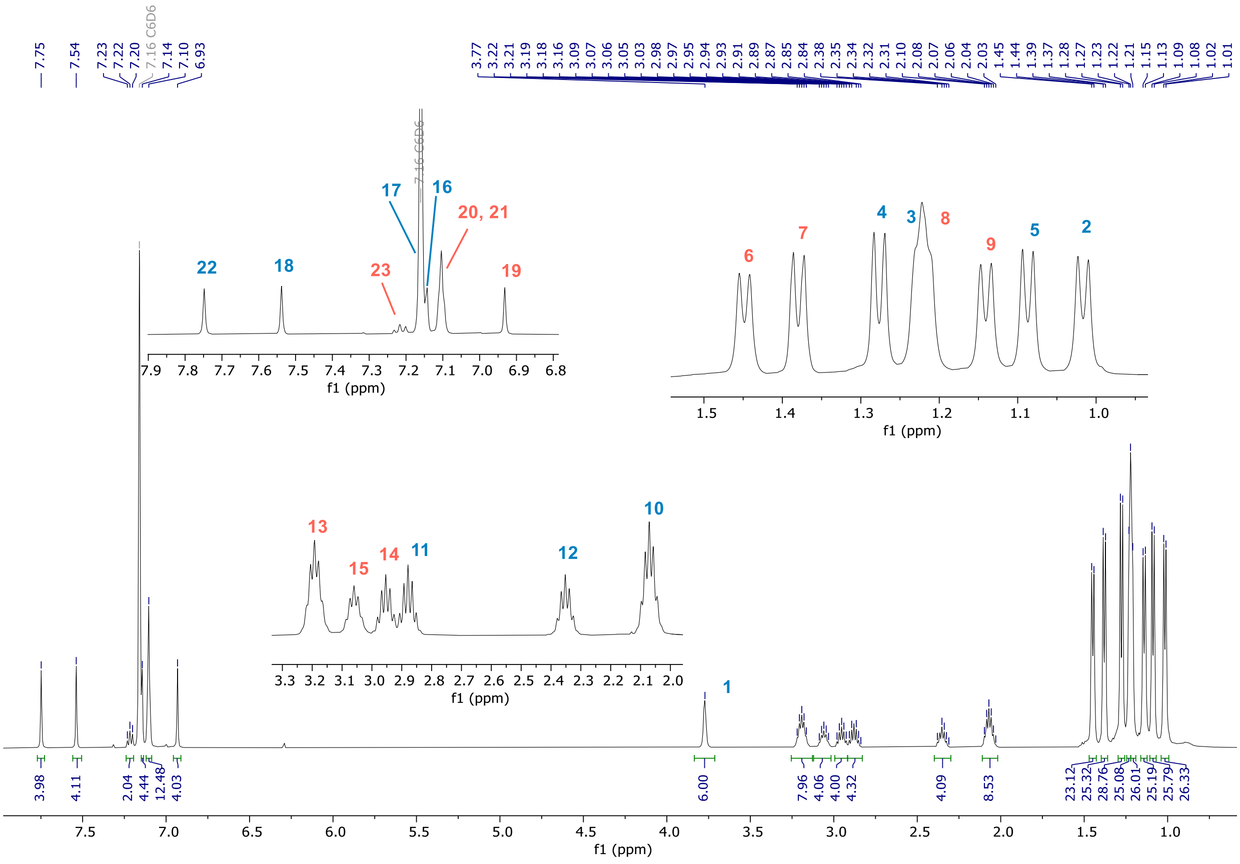

Excerpts from the ^{1}H-^{1}H ROESY NMR spectrum of **7** in $C_6D_6/CDCl_3$ 9:1:

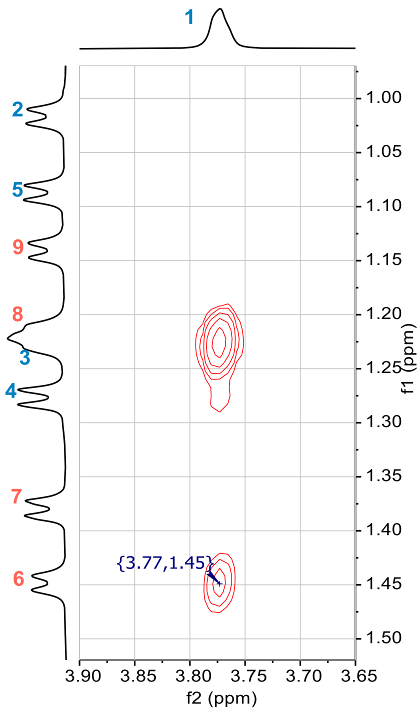

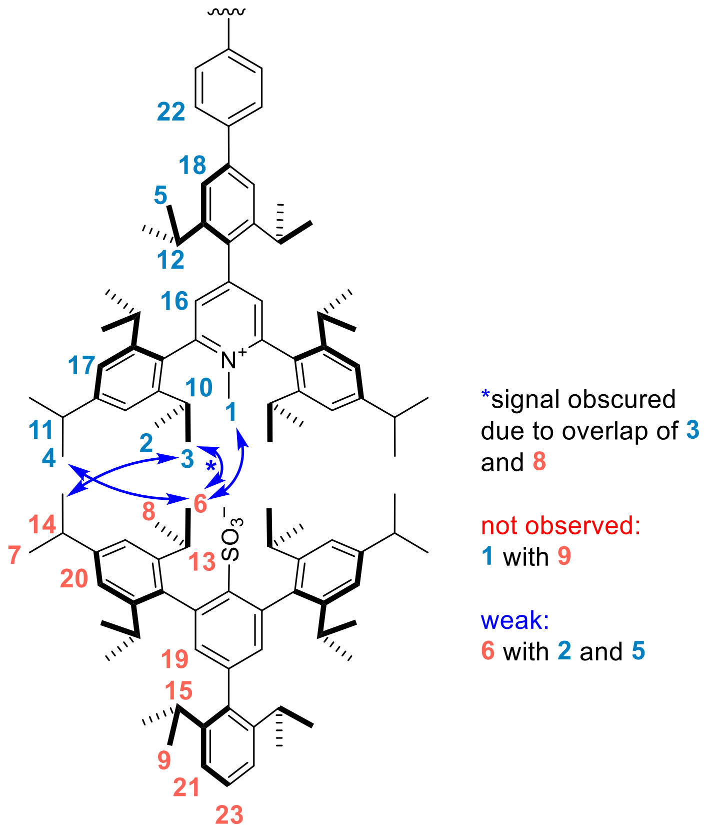

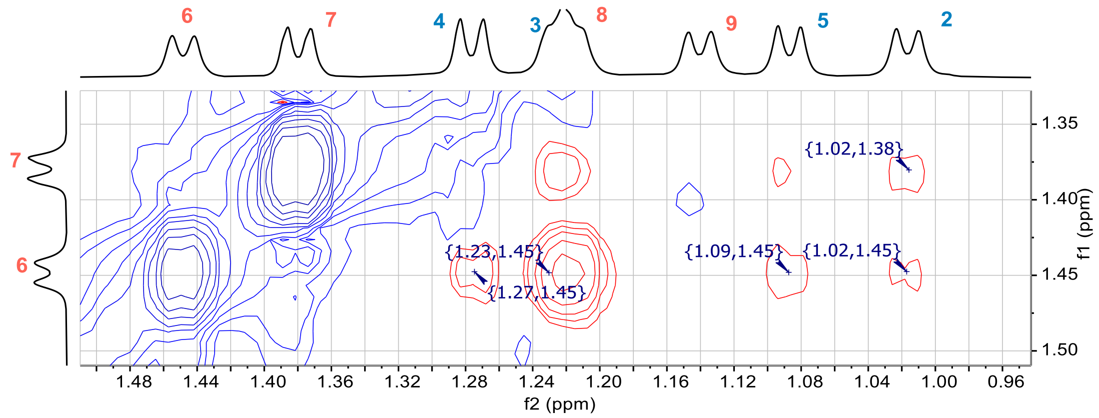

Comparison of the directionality in **7** in solution in $C_6D_6/CDCl_3$ 9:1 and in the solid state:

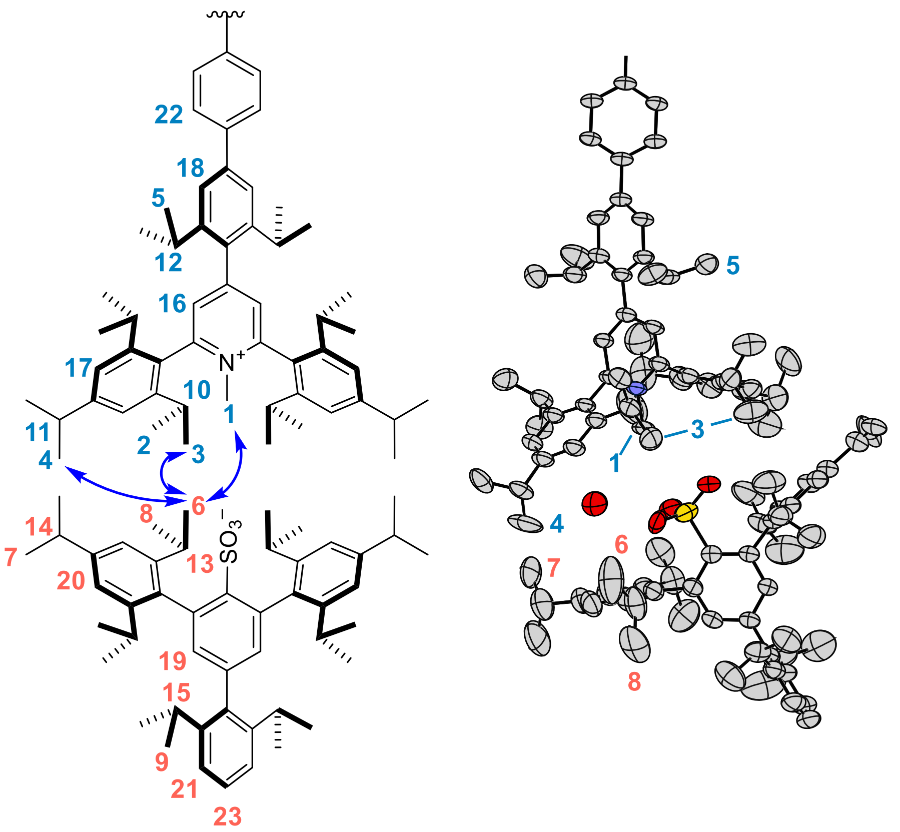

Selected ROE correlations and the corresponding distances, calculated from the integral intensities in **7** in $C_6D_6/CDCl_3$ 9:1:

Correlation	**F1, ppm**	**F2, ppm**	**Distance, Å**	**Closest distance in X-ray, Å**
1-3*	1.23	3.77	2.53	2.53
1-6	1.45	3.77	3.21	3.36
2-6	1.01	1.45	3.35	5.49
3-6**	1.23	1.45	2.57	2.85
4-6	1.45	1.27	2.62	2.52
5-6	1.08	1.45	3.25	7.11
2-7	1.02	1.38	4.30	4.13
6-10	1.44	2.07	2.91	4.68
6-22	1.44	7.75	–	12.36

*The distance between the two closest protons was taken from the crystal structure of **7** and used as a reference.

The integral intensity of signal ***3-6* was obtained as a difference between the integral intensities of ***3-2*** and ***6-(3&8)*** due to the overlap of signals ***3*** and ***8***.

Correlation	**F1, ppm**	**F2, ppm**	**Distance, Å**	**Closest distance in X-ray, Å**
1-10*	3.77	2.07	2.62	2.68
1-6	1.45	3.77	3.44	3.36
2-6	1.01	1.45	3.45	5.49
3-6**	1.23	1.45	2.63	2.85
4-6	1.45	1.27	2.71	2.52
5-6	1.08	1.45	3.34	7.11
2-7	1.02	1.38	4.41	4.13
6-10	1.44	2.07	3.05	4.68
6-22	1.44	7.75	–	12.36

* The distance between the two closest protons was taken from the crystal structure of **7** and used as a reference.

The integral intensity of signal ***3-6* was obtained as a difference between the integral intensities of ***3-2*** and ***6-(3&8)*** due to the overlap of signals ***3*** and ***8***.

Correlation	**F1, ppm**	**F2, ppm**	**Distance, Å**	**Closest distance in X-ray, Å**
10-12*	2.07	2.35	3.12	3.12
1-6	1.45	3.77	4.17	3.36
2-6	1.01	1.45	4.35	5.49
3-6**	1.23	1.45	3.33	2.85
4-6	1.45	1.27	3.40	2.52
5-6	1.08	1.45	4.22	7.11
2-7	1.02	1.38	5.58	4.13
6-10	1.44	2.07	3.77	4.68
6-22	1.44	7.75	–	12.36

* The distance between the two closest protons was taken from the crystal structure of **7** and used as a reference.

The integral intensity of ***3-6* was obtained as a difference between the integral intensities of ***3-2*** and ***6-(3&8)*** due to the overlap of signals ***3*** and ***8***.

2.9.4 Analysis of 8 by ROESY

^{1}H NMR (C_6D_6-$CDCl_3$) spectrum of **8** with assignments:

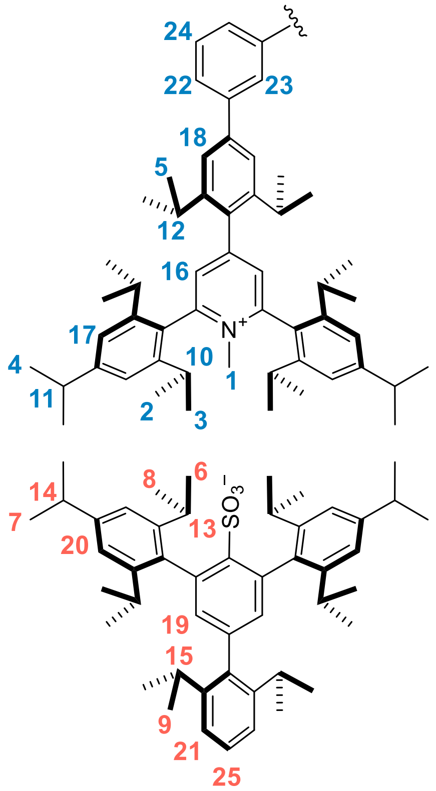

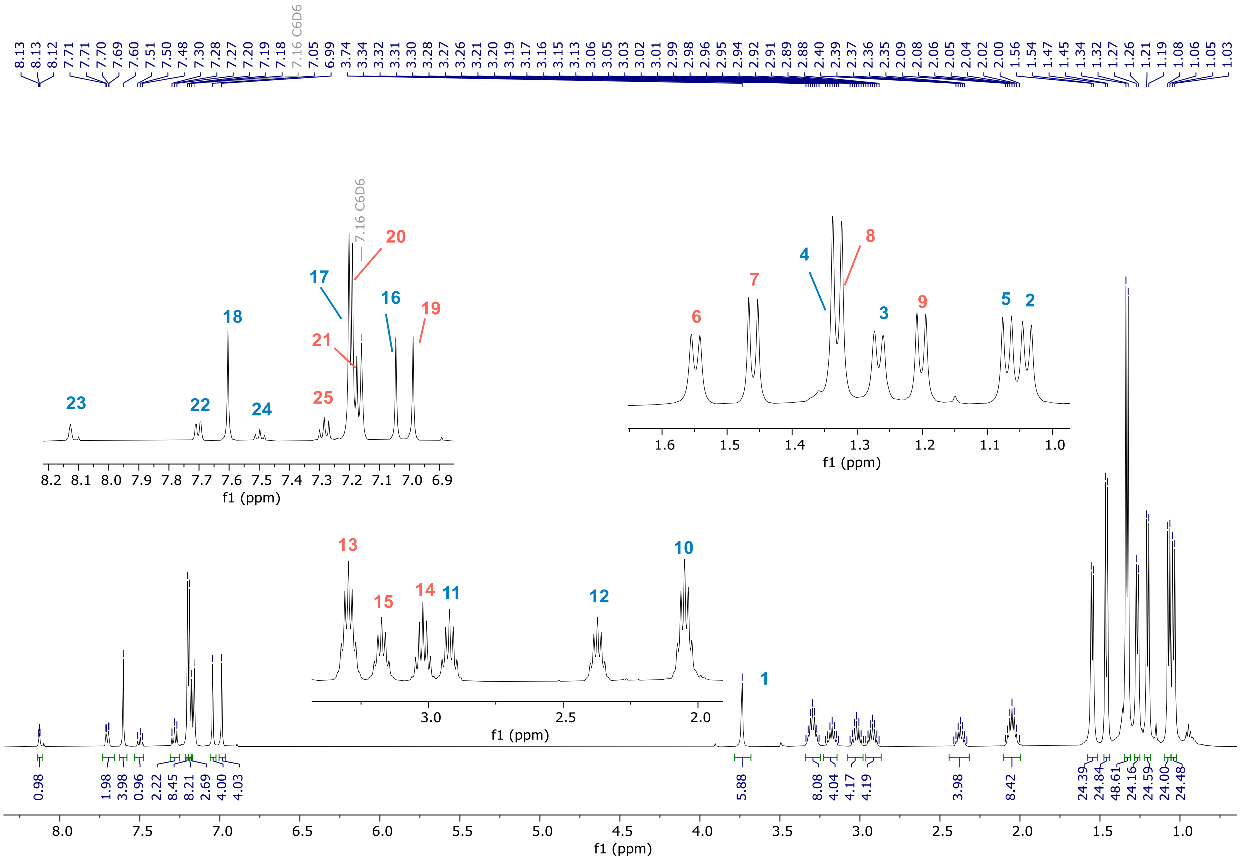

Excerpts from the ^{1}H-^{1}H ROESY NMR spectrum of **8** in C_6D_6:

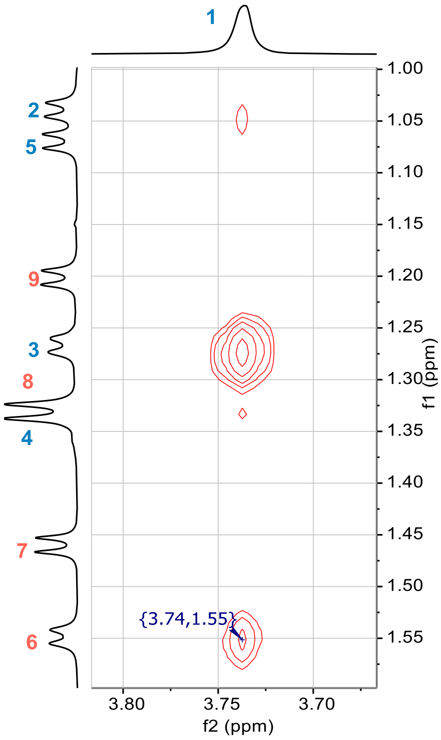

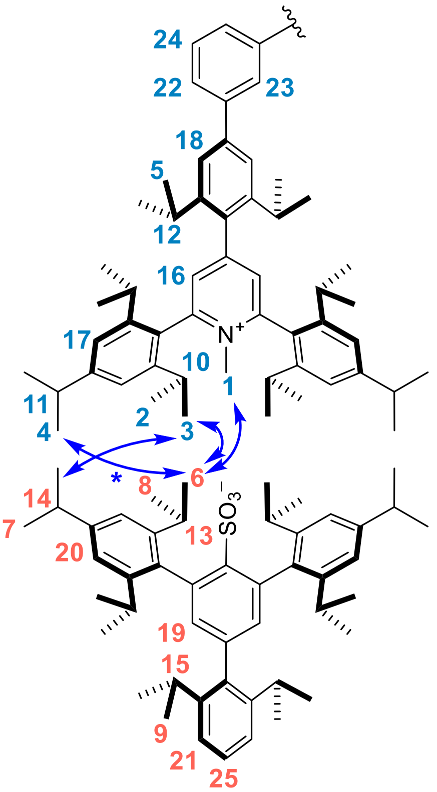

*signal obsured due to overlap of 4 and 8

not observed:
1 with 8 or 9

weak:
6 with 2 and 5

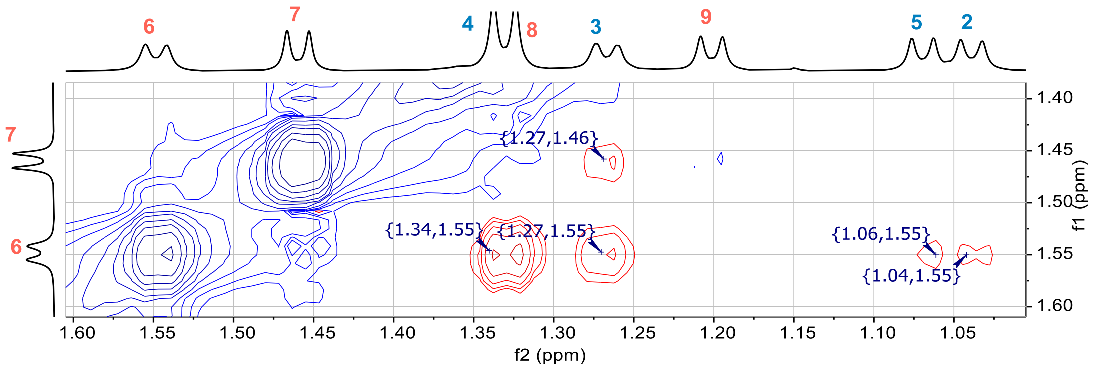

Selected ROE correlations and the corresponding distances, calculated from the integral intensities in **8** in C_6D_6:

Correlation	F1, ppm	F2, ppm	Distance, Å
1-3*	1.27	3.75	2.53
1-6	1.55	3.75	3.22
2-6	1.04	1.55	3.06
3-6	1.25	1.55	2.53
3-7	1.46	1.26	2.75
6-5	1.55	1.06	2.91
6-22	1.55	7.71	–

* The distance between the two closest protons was taken from the crystal structure of **7** and used as a reference.

Correlation	F1, ppm	F2, ppm	Distance, Å
1-10*	3.75	2.06	2.62
1-6	1.55	3.75	3.33
2-6	1.04	1.55	3.17
3-6	1.25	1.55	2.62
3-7	1.46	1.26	2.85
6-5	1.55	1.06	3.01
6-22	1.55	7.71	–

* The distance between the two closest protons was taken from the crystal structure of **7** and used as a reference.

Correlation	F1, ppm	F2, ppm	Distance, Å
10-12*	2.06	2.37	3.12
1-6	1.55	3.75	3.90
2-6	1.04	1.55	3.71
3-6	1.25	1.55	3.07
3-7	1.46	1.26	3.33
6-5	1.55	1.06	3.52
6-22	1.55	7.71	–

* The distance between the two closest protons was taken from the crystal structure of **7** and used as a reference.

2.9.5 Analysis of 3 in the presence of $^nBu_4N^+BF_4^-$

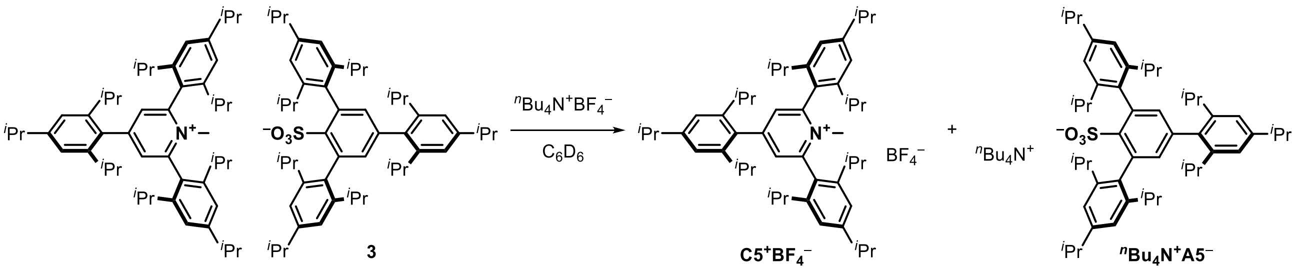

Procedure: $^nBu_4N^+BF_4^-$ (ca. 1.7 equiv.) was added to an NMR tube containing a solution of **3** in C_6D_6 at room temperature in an N_2 filled glovebox. The mixture was shaken and analyzed by NMR spectroscopy.

^{1}H NMR (C_6D_6) spectrum of **3** + $^nBu_4N^+BF_4^-$ with assignments:

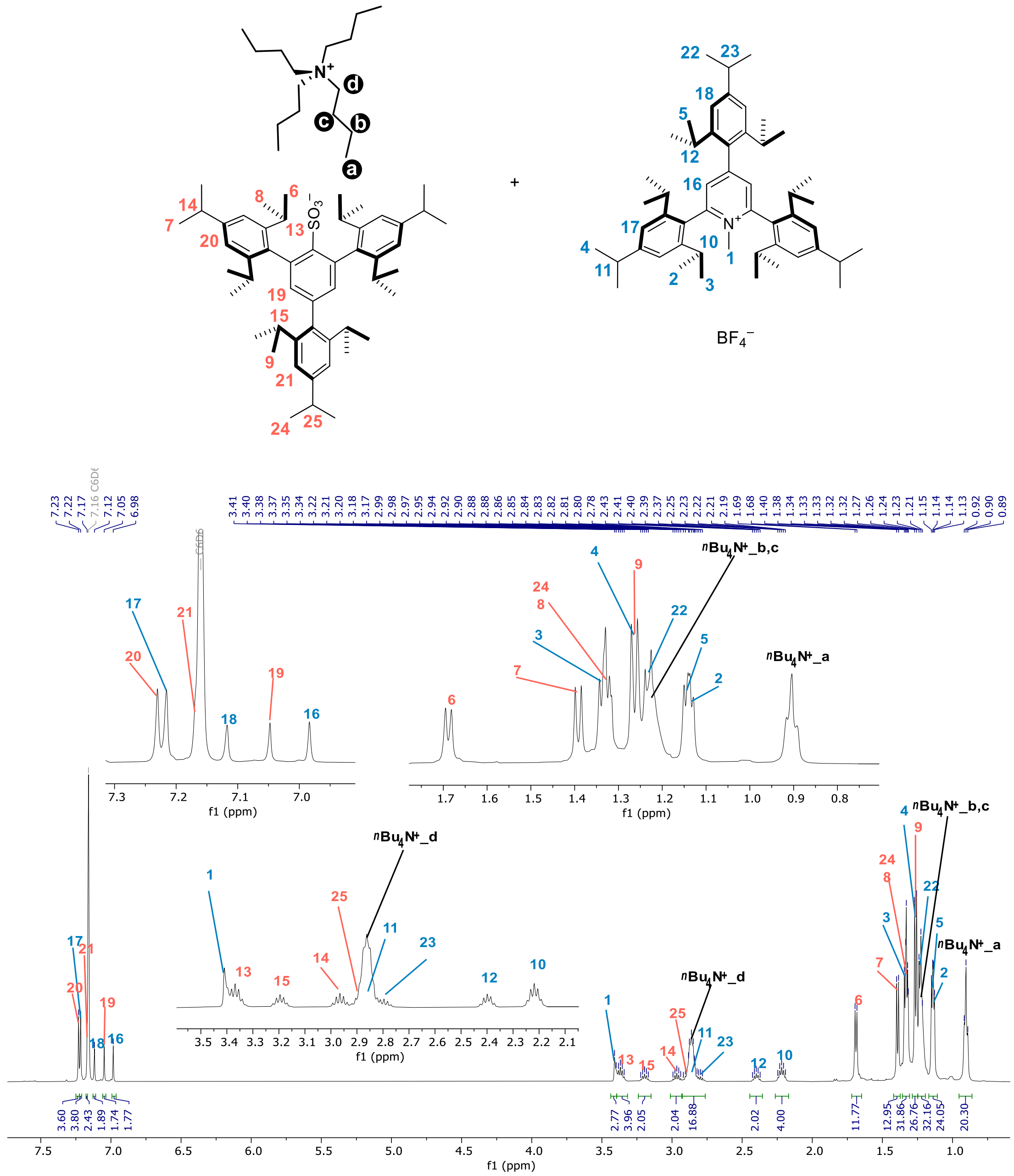

Excerpts from ^{1}H-^{1}H ROESY NMR of **3** + $^{n}Bu_4N^+BF_4^-$ in C_6D_6:

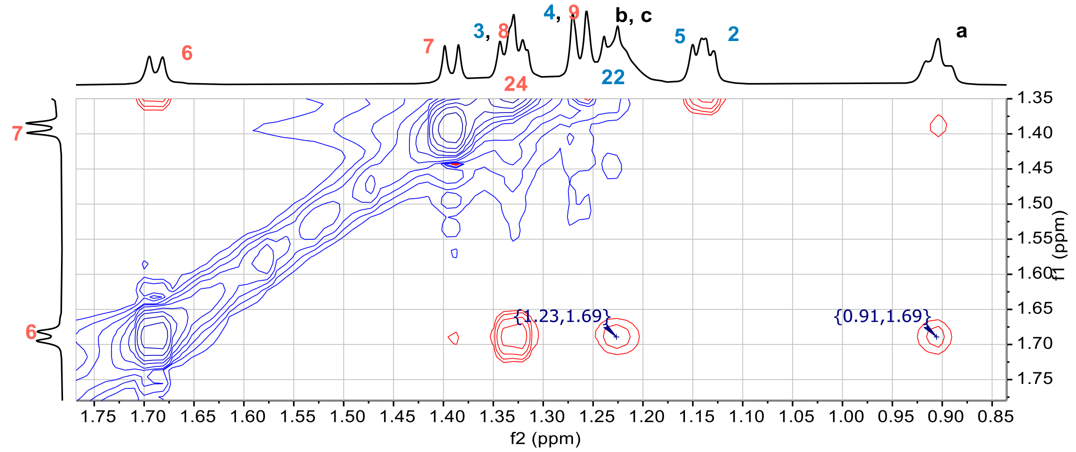

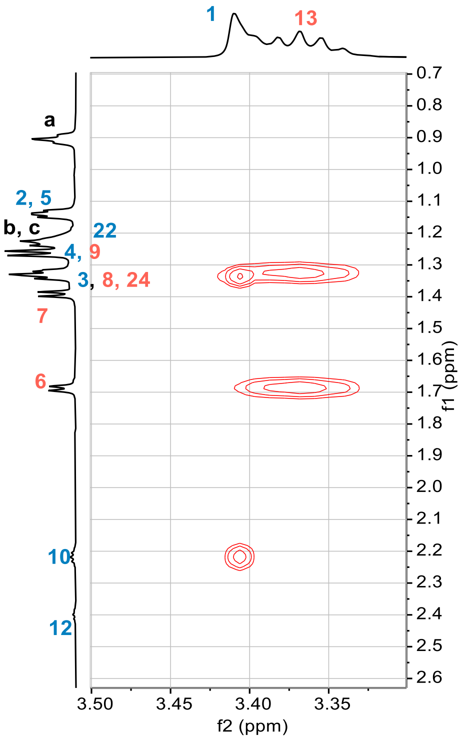

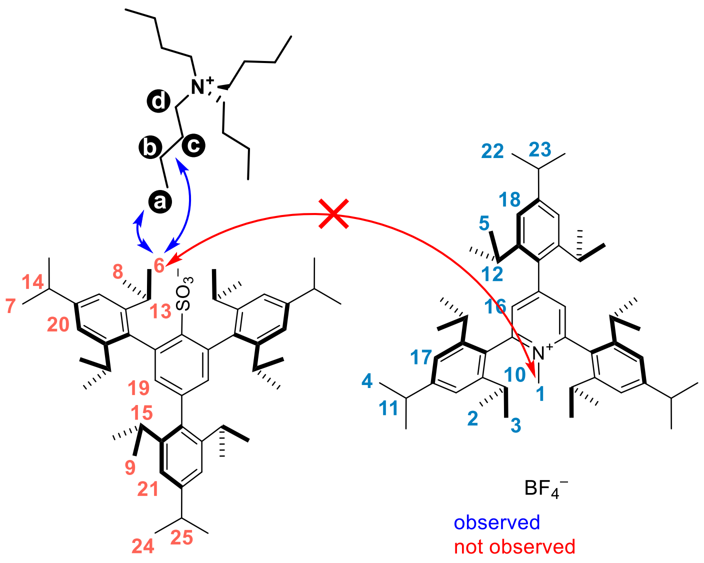

ROESY (C_6D_6) of **3** + $^nBu_4N^+BF_4^-$:

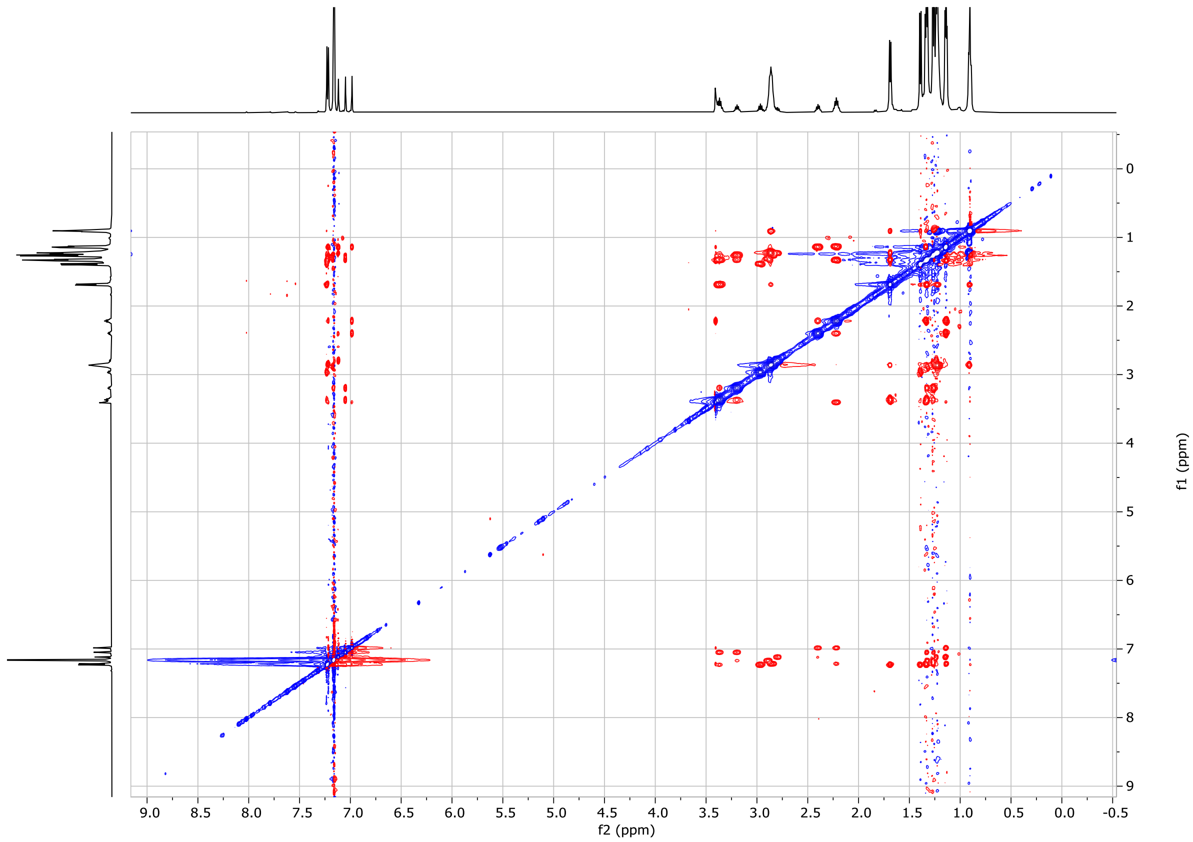

2.10 ^{1}H-^{1}H DOSY NMR experiments

The diffusion coefficients of ion pairs were determined in specified solvents at 298 K. For this purpose, bipolar gradient pulse sequence *ledbpgp2s* was employed on a Bruker Avance III 500 MHz spectrometer. The sigmoidal decay of intensity was fitted to the Stejskal-Tanner equation using the routine DOSY algorithm implemented in TopSpin 4.1.1. The hydrodynamic radii were calculated solving a modified Stokes-Einstein equation[11]:

$$D = \frac{k_B T}{\frac{6}{1 + 0.695\left(\frac{r_s}{r_h}\right)^{2.234}} \times \pi \eta r_h}$$

Where, D – diffusion coefficient [m^2 s^{-1}]; k_B – Boltzmann constant 1.3806485 × 10^{-23} [m^2 kg s^{-2} K^{-1}]; T – temperature [K]; r_s – hydrodynamic radius of the solvent [m]; r_h – hydrodynamic radius of the pair [m]; η – viscosity of the solvent at temperature T [kg m^{-1} s^{-1}]. Values for viscosity and solvent hydrodynamic radii were taken from the respective literature sources.[12,13] This approach has been previously used to estimate the size of various supramolecular systems.[14–16]

Calculated hydrodynamic parameters of **3** in different solvents using ^{1}H-^{1}H DOSY NMR:

Solvent	D × 10^{10} m^2 s^{-1}	r_s, nm	η × 10^4, kg m^{-1} s^{-1}	r_h, nm	d_h, nm
**C_6D_6**	5.618	0.270	6.03	0.70	1.40
**PhMe-*d*$_8$**	5.803	0.287	5.51	0.74	1.48
**$CDCl_3$**	4.860	0.260	5.42	0.87	1.74
**CD_2Cl_2**	8.314	0.249	4.13	0.68	1.36
**DMSO-*d*$_6$**	1.590	0.263	19.9	0.74	1.48

Calculated hydrodynamic parameters of different ion pairs in C_6D_6 by ^{1}H-^{1}H DOSY NMR.

Pair	D × 10^{10} m^2 s^{-1}	r_h, nm	d_h, nm
**2**	5.350	0.73	1.46
**3**	5.620	0.70	1.40
**C3$^+$A3$^-$**	7.260	0.56	1.12
**C2$^+$A4$^-$**	4.589	0.83	1.67
**C4$^+$A2$^-$***	5.080	0.76	1.52
**7***	2.868	1.29	2.58
**8**	3.488	1.07	2.14
**9**	2.250	1.63	2.26

*10% of $CDCl_3$ was added to cope with solubility issues in C_6D_6. It was assumed that this amount is not enough to compromise the estimation of the hydrodynamic radii.

Comparison of the hydrodynamic diameter obtained from the DOSY NMR data with the molecular size of **7** (the geometry of **7** is the experimental solid-state geometry):

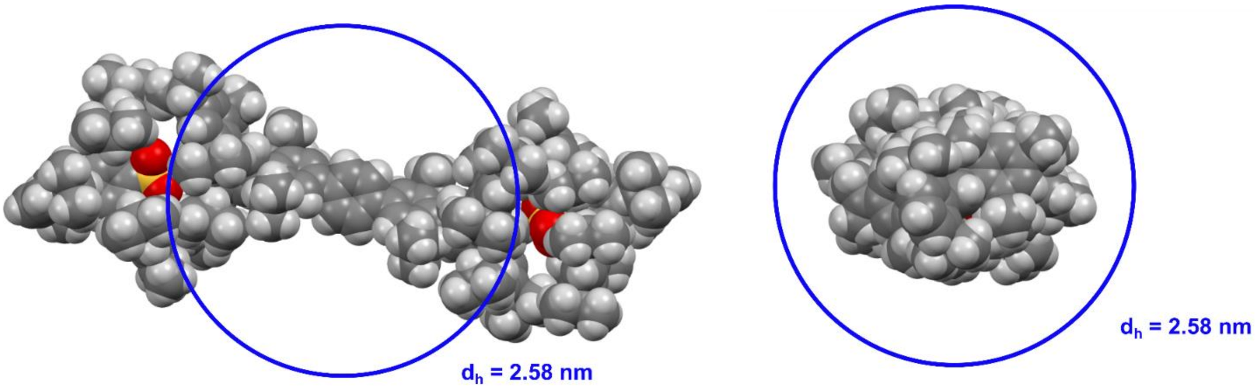

Comparison of the hydrodynamic diameter obtained from the DOSY NMR data with the molecular size of **8** (the geometry of **8** was optimized using the PM6 semi-empirical method by using fragments from separate geometries of **C5**$^+$ and **A5**$^-$ that were optimized at the M06-2X/6-31+G(d,p) level of theory):

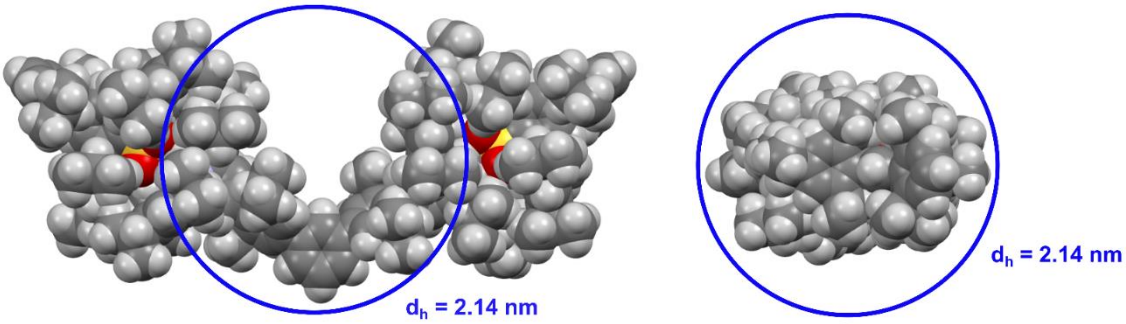

^{1}H-^{1}H DOSY NMR of **C2^{+}A4$^{–}$** in C_6D_6:

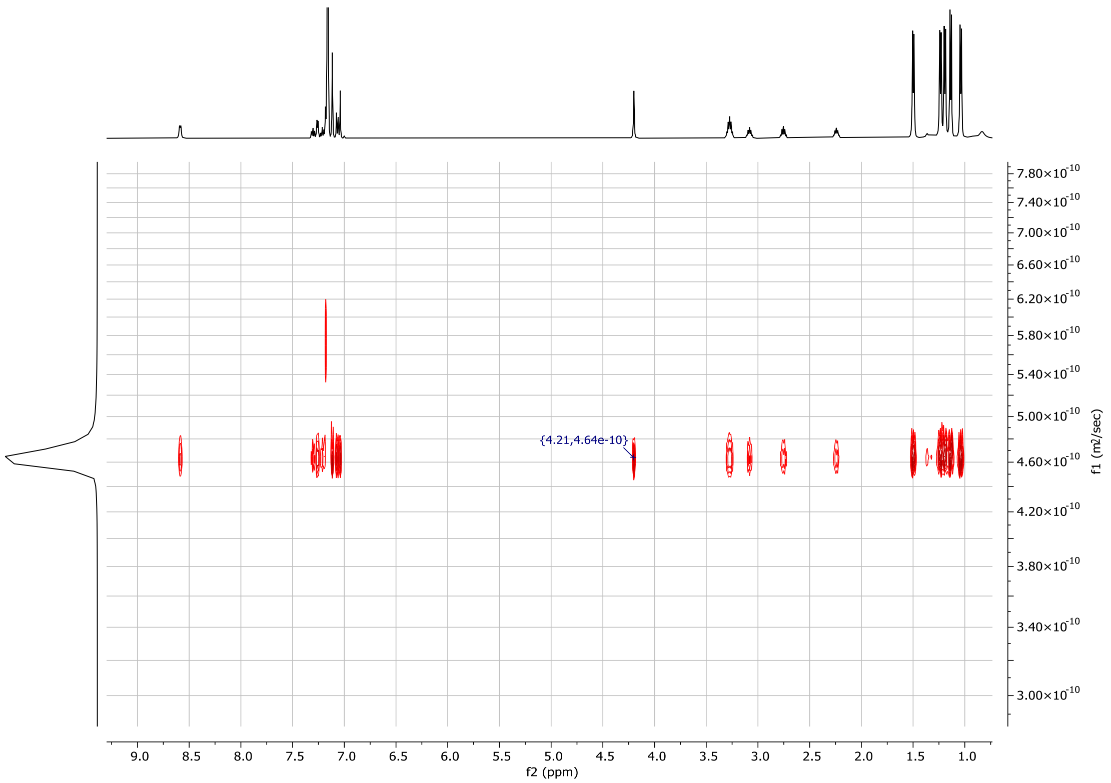

^{1}H-^{1}H DOSY NMR of **C4^{+}A2$^{–}$** in C_6D_6/$CDCl_3$ 9:1:

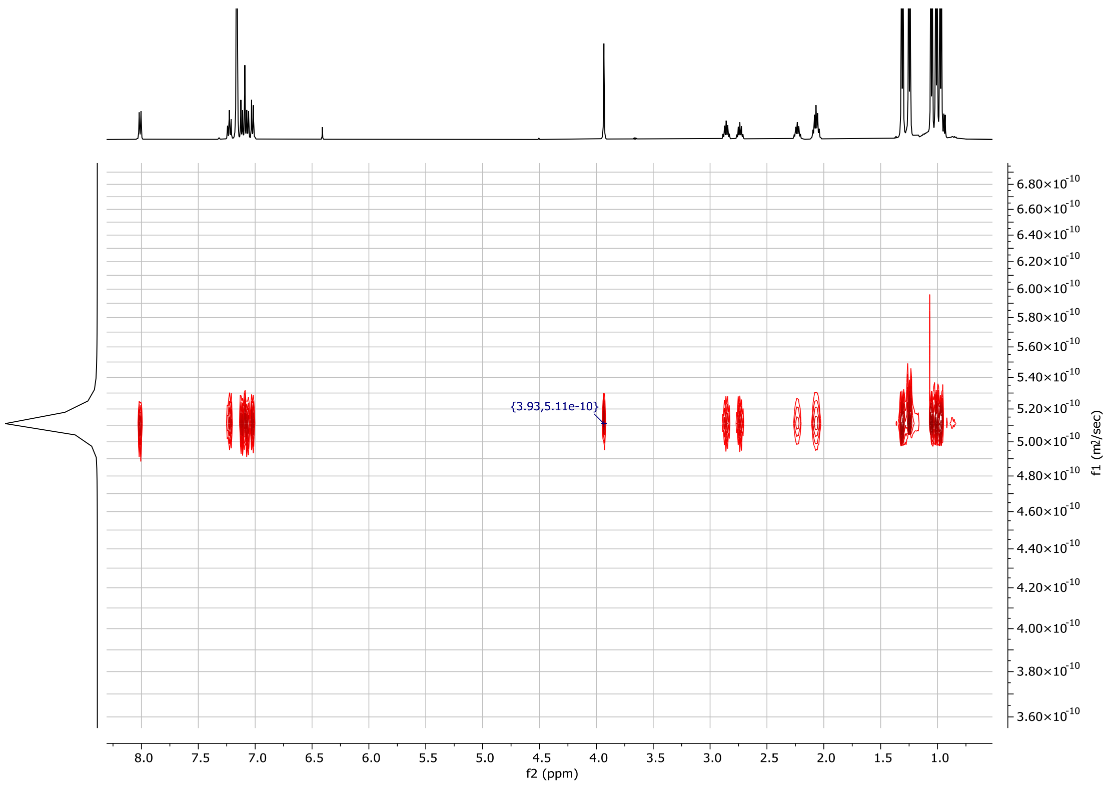

^{1}H-^{1}H DOSY NMR of **C3⁺A3⁻** in C_6D_6:

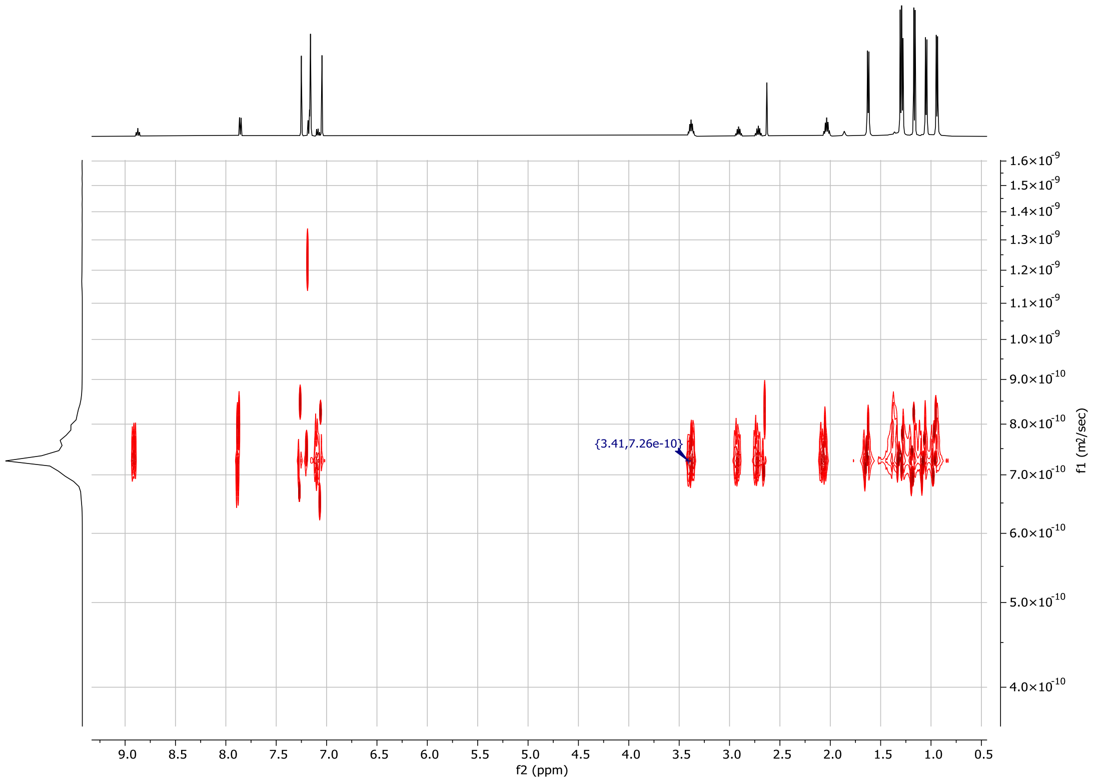

^{1}H-^{1}H DOSY NMR of **2** in C_6D_6:

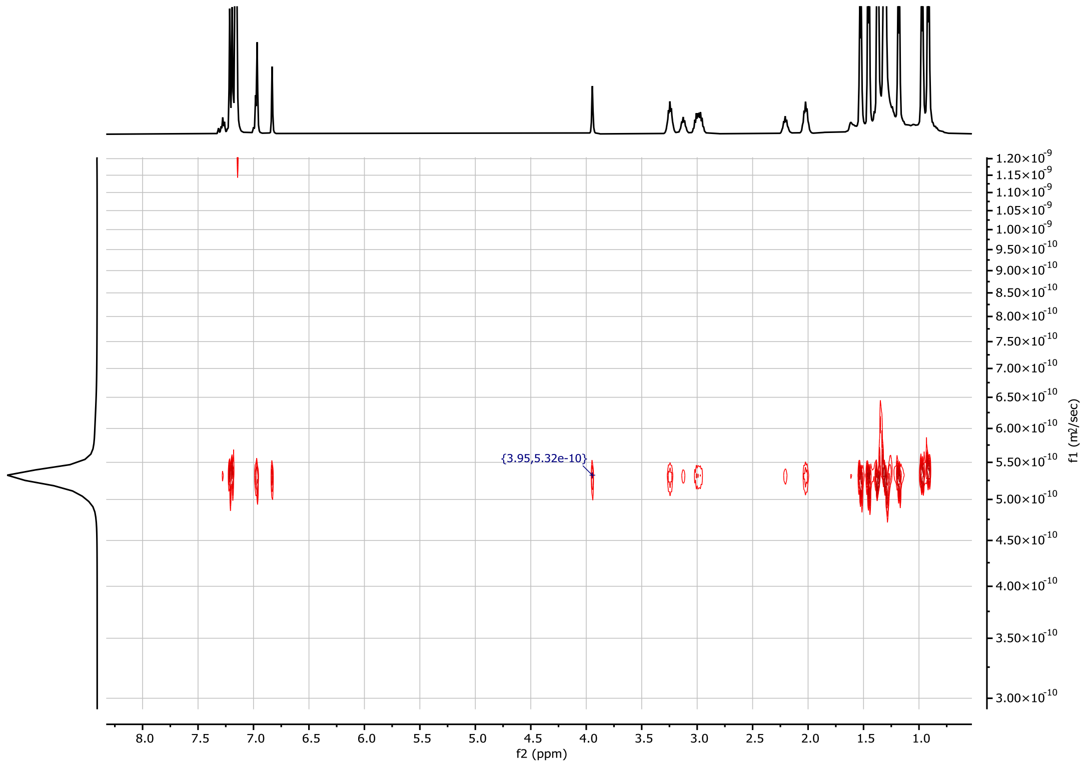

^{1}H-^{1}H DOSY NMR of **3** in C_6D_6:

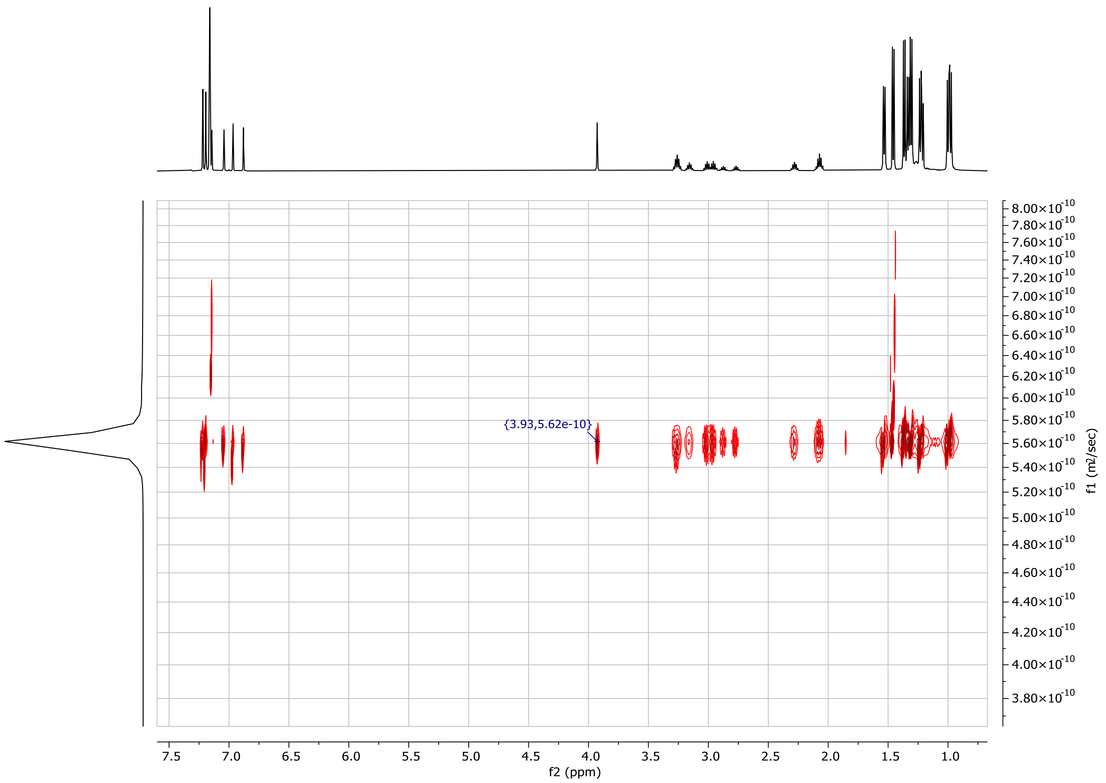

^{1}H-^{1}H DOSY NMR of **3** in toluene-d_8:

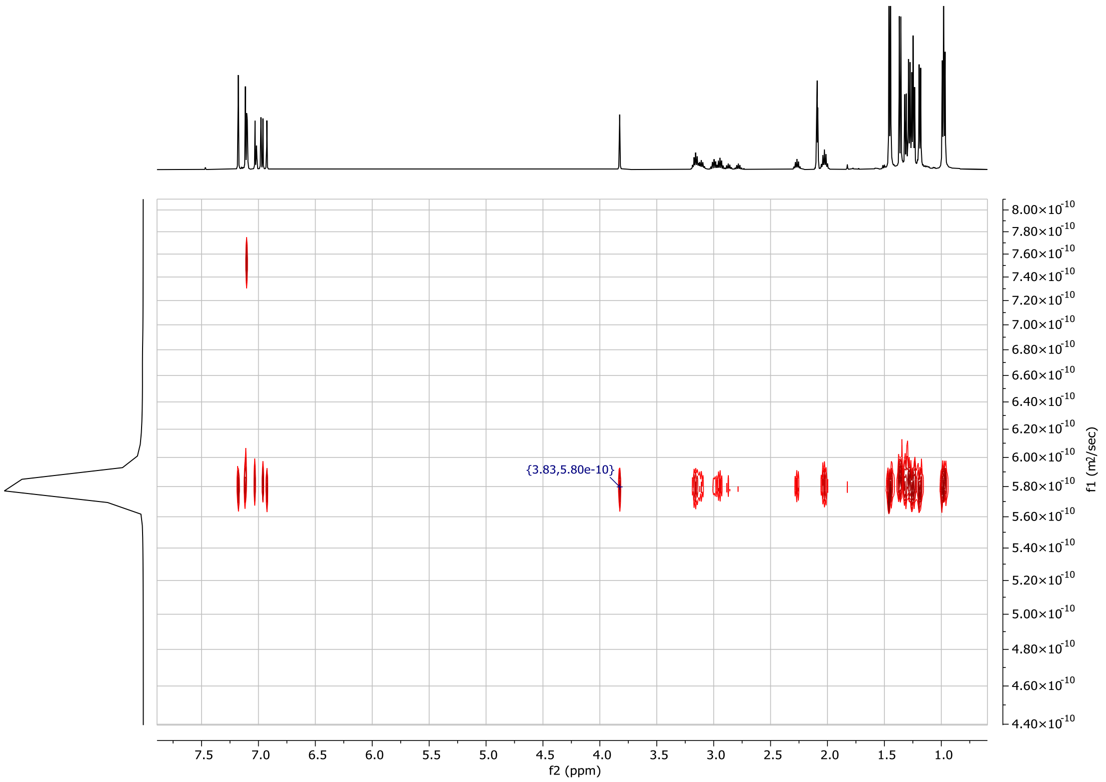

^1H-^1H DOSY NMR of **3** in $CDCl_3$:

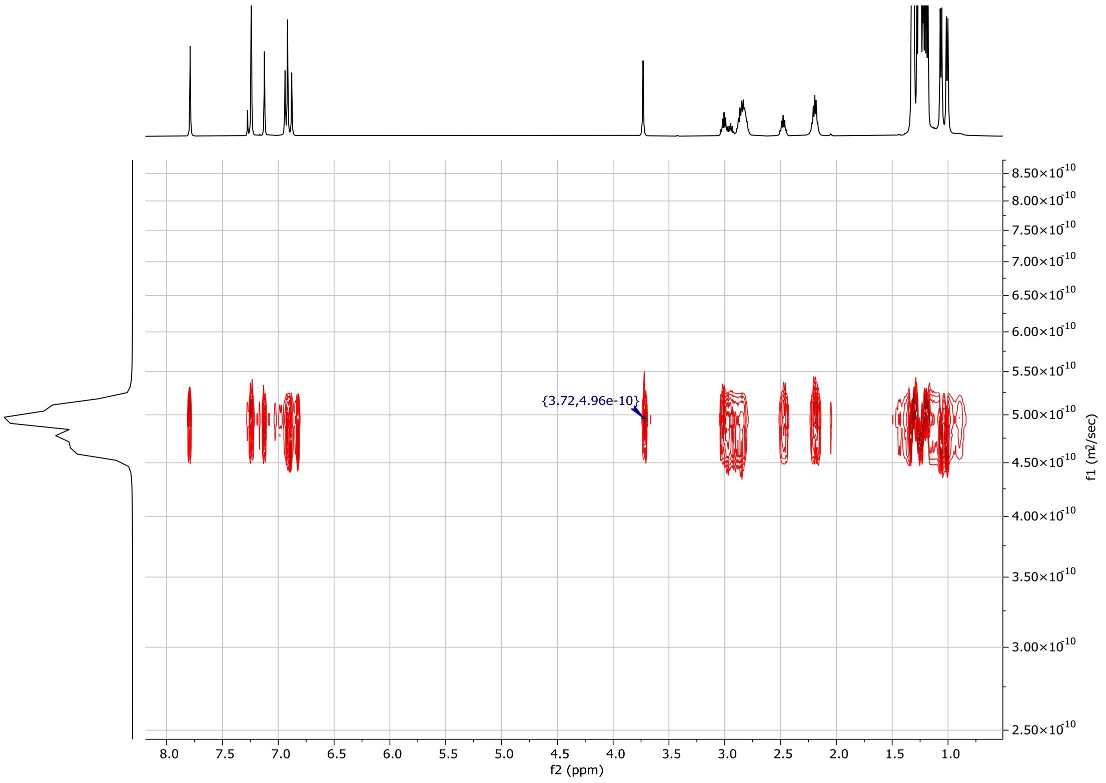

^1H-^1H DOSY NMR of **3** in CD_2Cl_2:

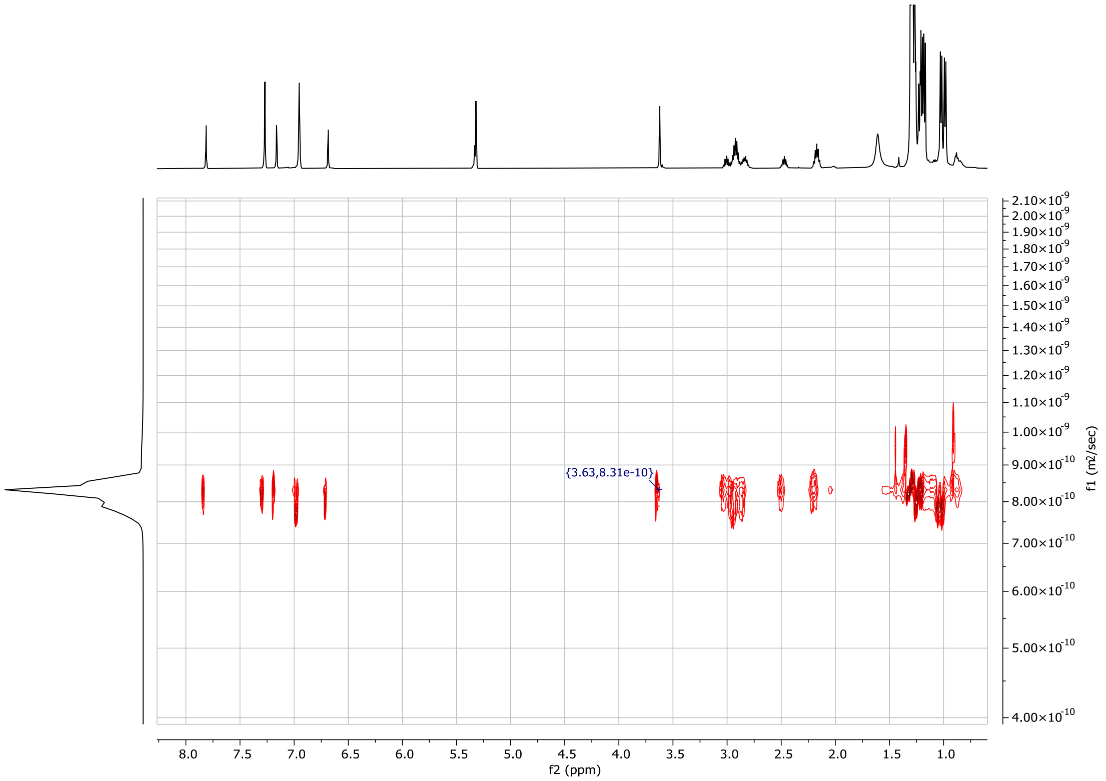

^{1}H-^{1}H DOSY NMR of **3** in DMSO-d_6:

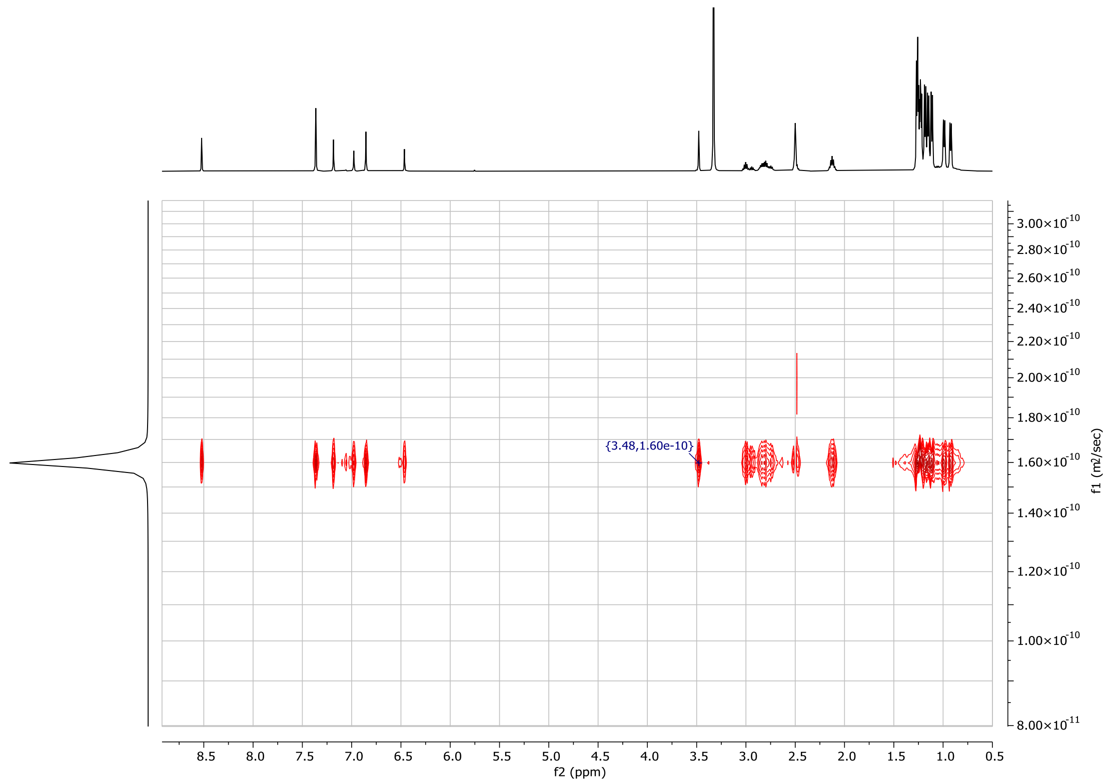

^{1}H-^{1}H DOSY NMR of **7** in C_6D_6/$CDCl_3$ 9:1:

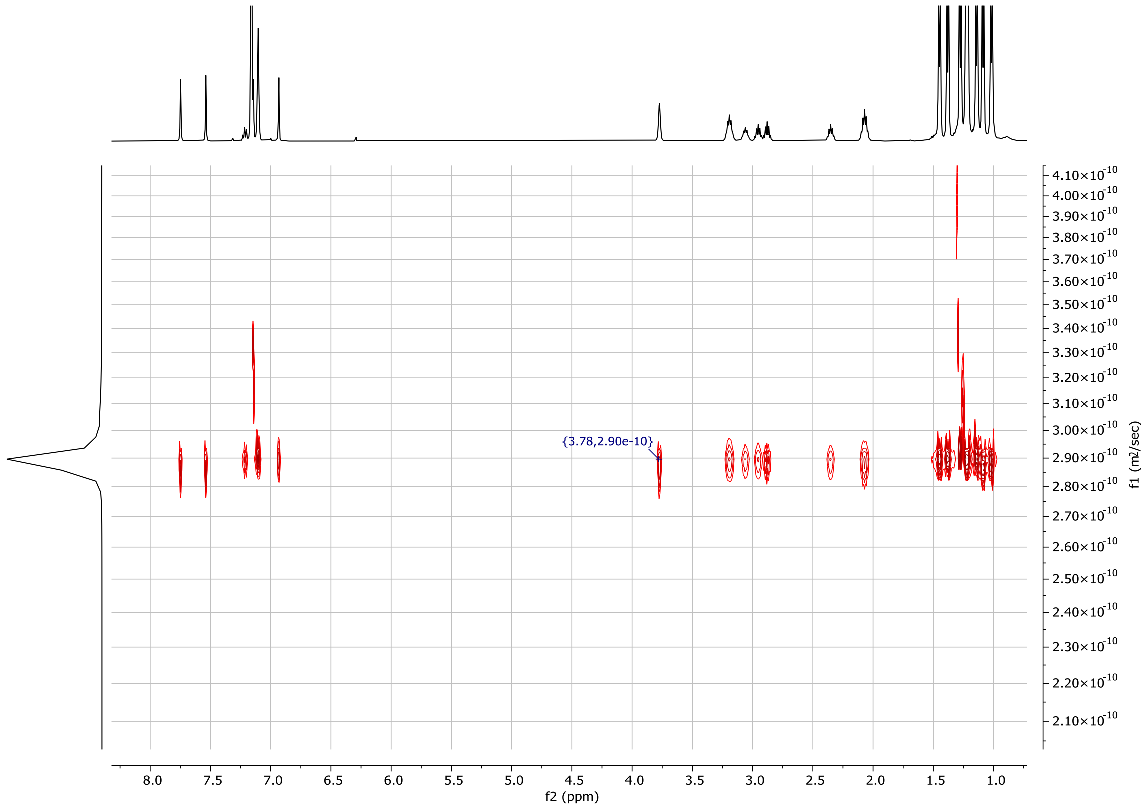

^{1}H-^{1}H DOSY NMR of **8** in C_6D_6:

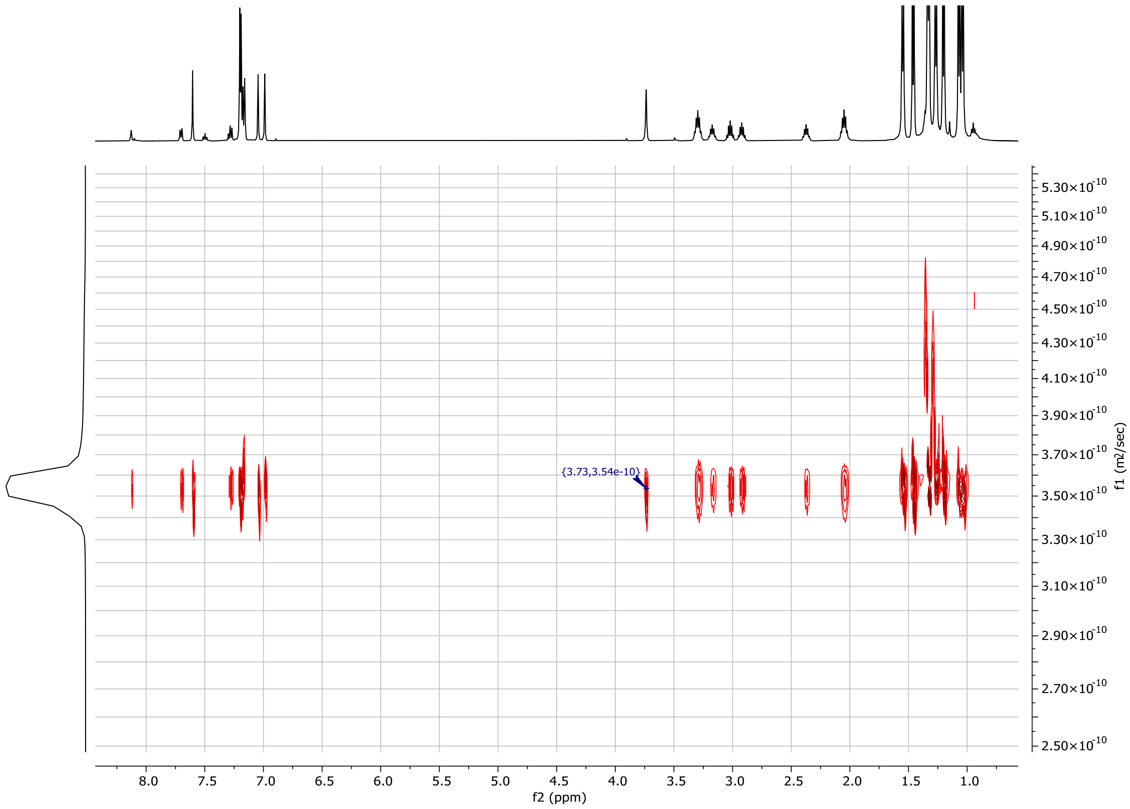

^{1}H-^{1}H DOSY NMR of **9** in C_6D_6:

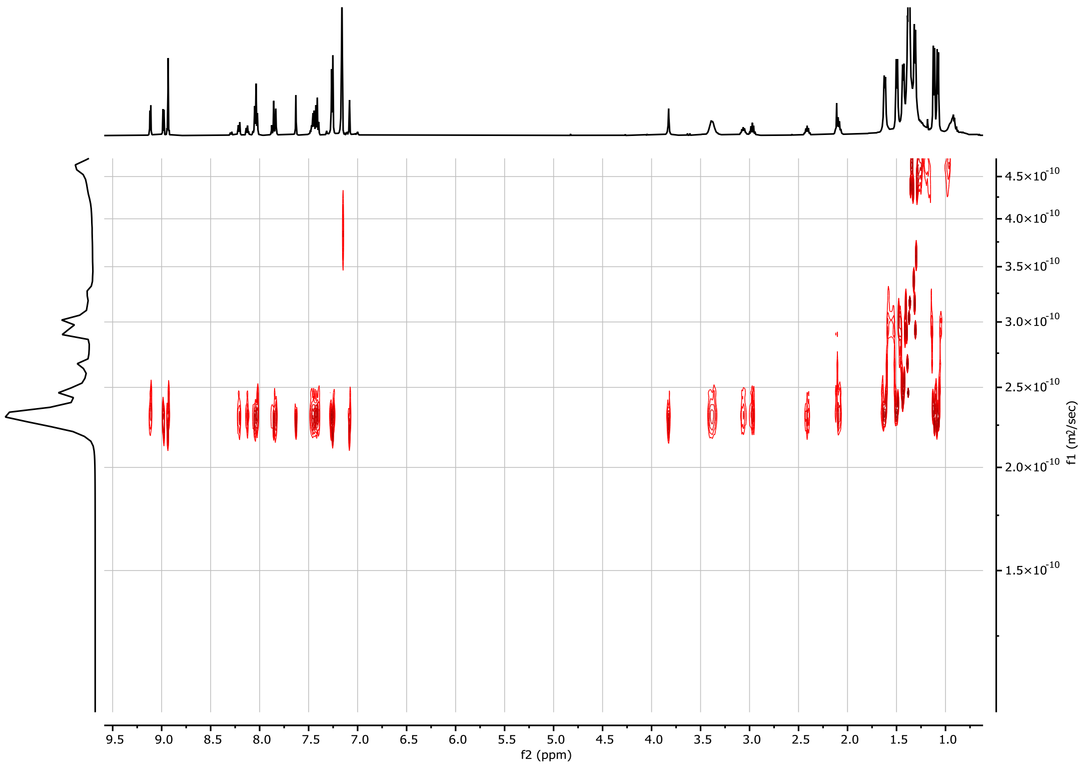

2.11 Thermal stability analysis

2.11.1 Thermogravimetric analysis (TGA) and melting points

Compound	$\mathbf{C5^{+}BF_4^{-}}$	$\mathbf{Na^{+}A5^{–}}$	**3**	$\mathbf{C7^{+}BF_4^{-}}$	$\mathbf{Na^{+}A7^{–}}$	**5**
Melting point	180 °C	211 °C	213 °C	Solid at 300 °C	285 °C	Solid at 300 °C

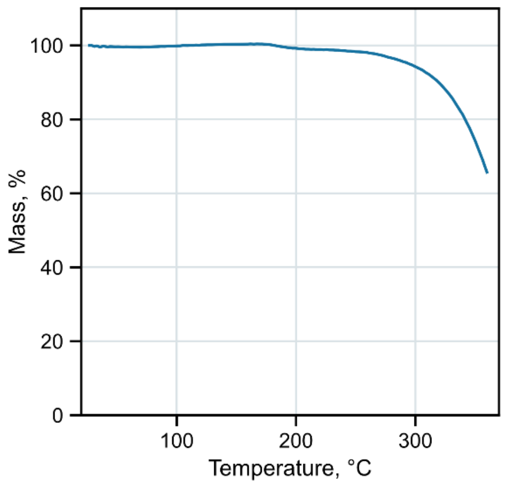

TGA trace of **3**

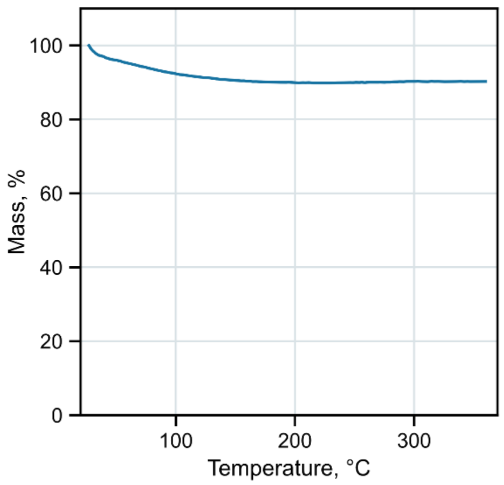

TGA trace of **5**

Compound	$\mathbf{C10^{3+}(BF_4^{-})_3}$	$\mathbf{(Na^{+})_3A10^{3–}}$	**10**
Melting point	Solid at 300 °C	Solid at 300 °C	Solid at 300 °C

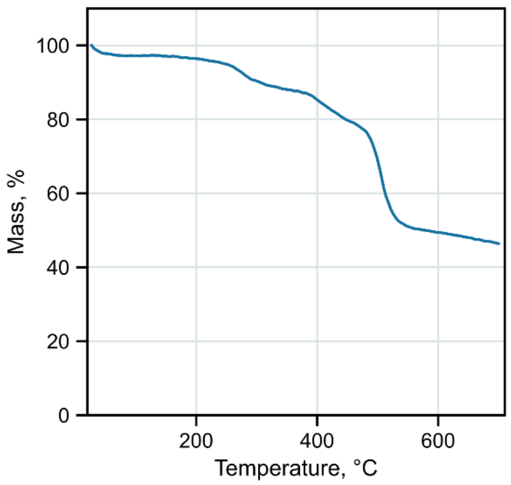

TGA trace of **10**

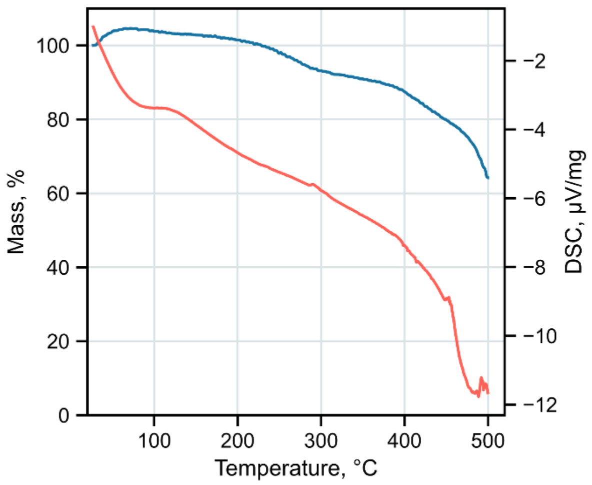

TGA (blue) and DSC (red) traces of **10**

2.11.2 NMR studies of thermal stability of 10

^{1}H NMR spectrum ($CDCl_3$, 400 MHz) of **$C10^{3+}(BF_4^-)_3$** before heating (blue), and after 1 h at 250 °C (red):

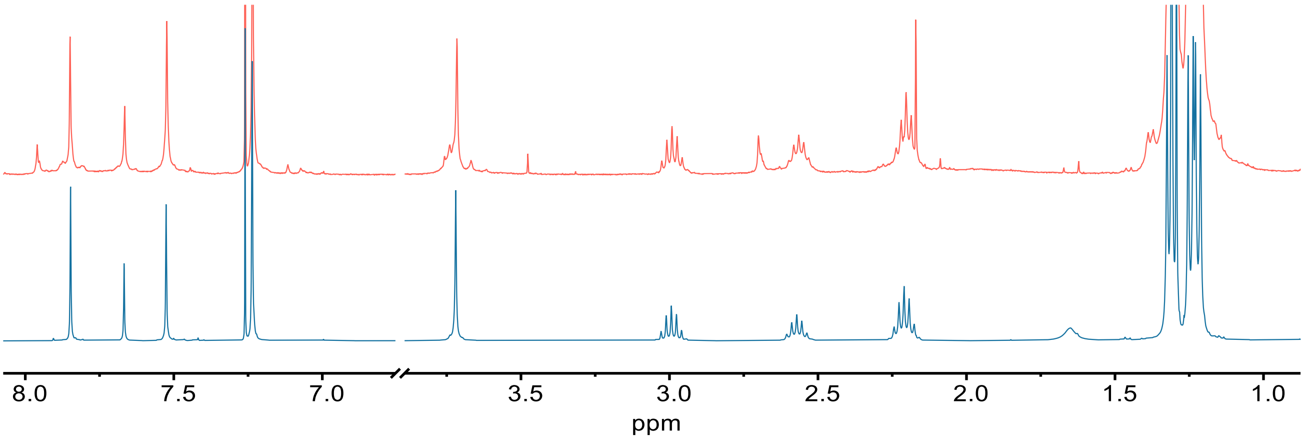

^{1}H NMR spectrum ($CDCl_3/CD_3OD$, 400 MHz) of **$(Na^+)_3A10^{3-}$** before heating (blue), and after 1 h at 250 °C (red):

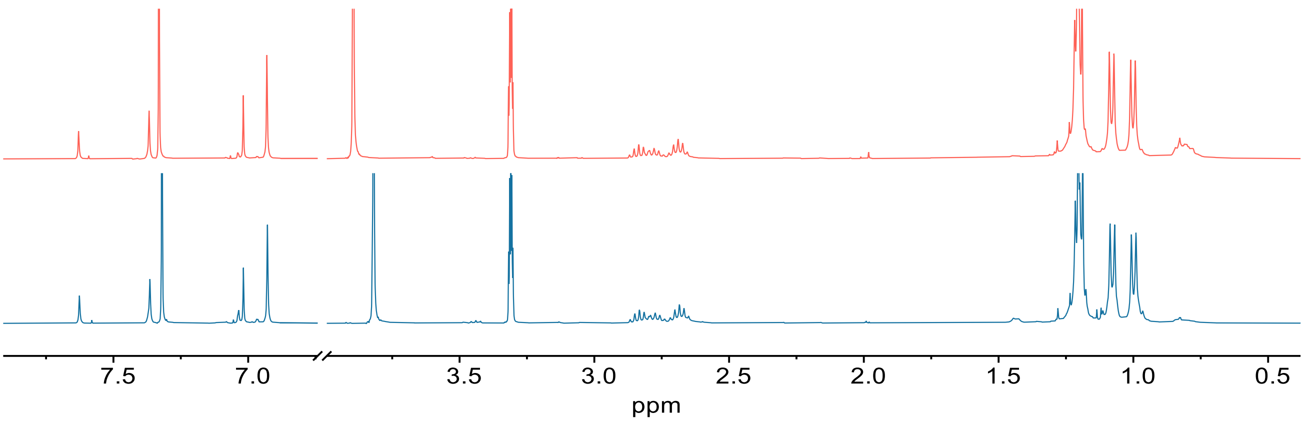

^{1}H NMR spectrum ($CDCl_3/CD_3OD$, 400 MHz) of **10** before heating (blue), and after 1 h at 250 °C (red):

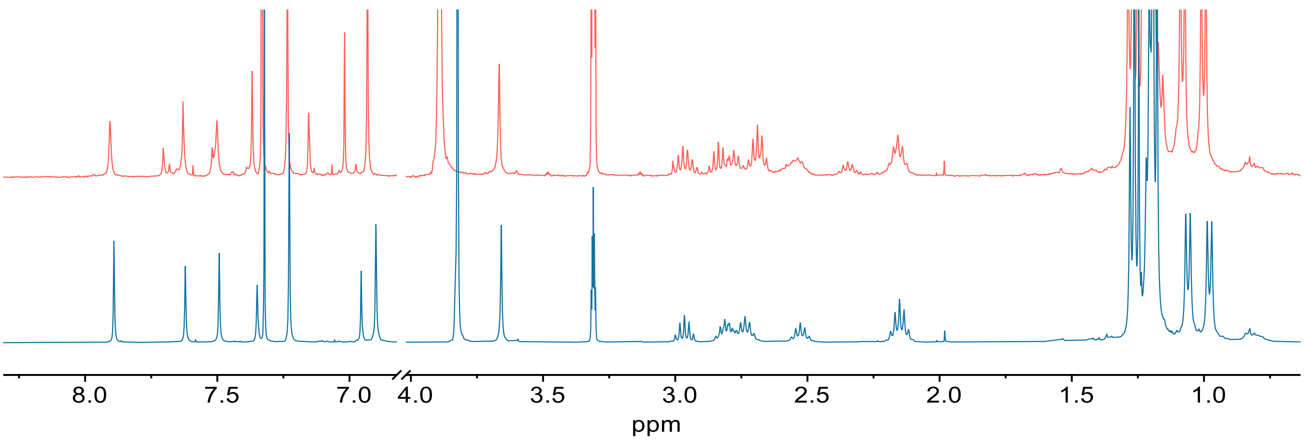

2.12 Computational studies

Density functional theory (DFT) calculations were performed using the Gaussian 16 (revision C.01) program.[17] The initial geometries of the cations **C2⁺**, **C3⁺**, **C4⁺** and **C5⁺**, anions **A2⁻**, **A3⁻**, **A4⁻**, **A5⁻**, and pair **3** were taken from the X-ray crystal structures of the corresponding ion pairs **C2⁺A4⁻**, **C3⁺A3⁻**, **C4⁺A2⁻** and **3**. Geometry optimizations were performed at the M06-2X level of theory with the 6-31+G(d,p) basis set in the gas phase. Harmonic vibrational frequencies were evaluated for the optimized geometries, with minima characterized by the absence of imaginary frequencies. Quasiharmonic corrections to the entropy for frequencies below 100 cm^{-1} were calculated with the Goodvibes[18] program by employing the method of Grimme.[19] Single point energies were calculated at M06-2X level of theory with the 6-311++G(3df,2p) basis set in the gas phase or with implicit solvation model CPCM.[20,21] The employed level of theory has been used previously for anionic supramolecular systems.[22] The cube files, containing the electrostatic potential and the SCF density data, were generated by a standalone Gaussian utility *cubegen*. The electrostatic potential maps were visualized in VMD.[23] The optimized structures were visualized in CYLView.[24]

	ΔG_{qh}	G_{qh}	ΔG	G	E_{elec}
	M06-2X/6-311++G(3df,2p)//M06-2X/6-31+G(d,p)				
**C5⁺**	-	-2041.28953	-	-2041.30580	-2042.34402
**A5⁻**	-	-2608.92386	-	-2608.93809	-2609.95116
**3**	-51.8	-4650.29594	-49.2	-4650.32232	-4652.41850
	CPCM(benzene)/M06-2X/6-311++G(3df,2p)//M06-2X/6-31+G(d,p)				
**C5⁺**	-	-2041.31791	-	-2041.33417	-2042.37239
**A5⁻**	-	-2608.96744	-	-2608.98167	-2609.99474
**3**	-15.4	-4650.30990	-12.8	-4650.33628	-4652.43246
	CPCM(CH_2Cl_2)/M06-2X/6-311++G(3df,2p)//M06-2X/6-31+G(d,p)				
**C5⁺**	-	-2041.33544	-	-2041.35171	-2042.38993
**A5⁻**	-	-2608.99711	-	-2609.01134	-2610.02441
**3**	8.0	-4650.31978	10.6	-4650.34617	-4652.44234
	CPCM(H_2O)/M06-2X/6-311++G(3df,2p)//M06-2X/6-31+G(d,p)				
**C5⁺**	-	-2041.34091	-	-2041.35718	-2042.39540
**A5⁻**	-	-2609.00693	-	-2609.02115	-2610.03422
**3**	15.5	-4650.32312	18.1	-4650.34951	-4652.44568

Electronic and free energies are in Hartree, ΔG and ΔG_{qh} values are in kcal/mol and represent the differences in free energies of the ion pair compared with the separated ions, ΔG_{qh} and G_{qh} include quasiharmonic corrections to the entropy.

Energy decomposition analysis

The decomposition analyses of interaction energies were performed using symmetry-adapted perturbation theory (SAPT)[25,26] employing the SAPT0 algorithm[27,28] implemented in the Psi4 software.[29] The total energy (E_{total}) by the SAPT0 analysis is divided into contributions from electrostatics (E_{elst}), induction (E_{ind}), dispersion (E_{disp}), and exchange (E_{exch}). The DFT optimized geometry of **3** was used to generate truncated geometries **3$_{trunc}$A-D**. For **3$_{trunc}$B**, the SO_3 group was replaced with the H atom, while N-CH_3 group was replaced with a C-H group. The other structures were obtained by replacing the 2,4,6-triisopropylphenyl-substituents at the 2- and 6-positions of the central aromatic rings of both ions (**3$_{trunc}$A**), of the anion only (**3$_{trunc}$C**), or of the cation only (**3$_{trunc}$D**) with H atoms. The truncated geometries **1$_{trunc}$**, **4$_{trunc}$**, and **6$_{trunc}$** were obtained from the X-ray crystal structures of **1**, **4**, and **6**, respectively, by replacing the 2,6-diisopropylphenyl substituents from the 4-positions of the central aromatic rings of the ions with H atoms. Additionally, to reduce the computational time in the case of **6$_{trunc}$**, one of the *p*-^{t}Bu-phenyl groups (which points away from the anion) was removed from each of the two shielding groups, and replaced with an H atom. Then, geometry optimizations were performed at the M06-2X/6-31+G(d,p) (for **1$_{trunc}$**) or M06-2X/6-31G(d) (**4$_{trunc}$** and **6$_{trunc}$**) levels of theory. Structures **4$_{trunc}$A** and **6$_{trunc}$A** were obtained by replacing the substituents at the 2- and 6-positions of the central aromatic rings of **4$_{trunc}$** and **6$_{trunc}$**, respectively, with H atoms. The SAPT0 energy decomposition analyses were then performed with the jun-cc-pVDZ basis set.

The overview of the results of the SAPT calculations:

	E_{elst}	E_{ind}	E_{exch}	E_{disp}	E_{total}
**1$_{trunc}$**	-74.80	-18.73	52.42	-44.48	-85.59
**3**	-79.96	-21.04	65.44	-53.91	-89.46
**3$_{trunc}$A**	-76.70	-10.85	15.15	-5.79	-78.20
**3 - 3$_{trunc}$A**	-3.26	-10.19	50.29	-48.12	**-11.26 (13%)**
**3$_{trunc}$B**	-12.10	-3.68	37.11	-38.24	-16.91
**3$_{trunc}$C**	-72.52	-18.64	30.74	-16.44	-76.87
**3$_{trunc}$D**	-71.24	-12.35	16.59	-8.96	-75.96
**4$_{trunc}$**	-76.10	-19.48	57.64	-50.56	-88.51
**4$_{trunc}$A**	-77.73	-7.73	15.25	-4.74	-74.96
**4 - 4$_{trunc}$A**	1.63	-11.75	42.39	-45.82	**-13.55 (15%)**
**6$_{trunc}$**	-82.89	-23.32	79.17	-71.69	-98.73
**6$_{trunc}$A**	-75.44	-7.86	13.09	-4.01	-74.23
**6 - 6$_{trunc}$A**	-7.45	-15.46	66.08	-67.68	**-24.50 (25%)**

All energies are in kcal/mol.

The geometries based on **1$_{trunc}$** with different N···S distances (Figure S2c) were generated by incremental increase of the distance between carbons at the 4-positions of the central arenes of the cation and the anion and were not further optimized. The SAPT0 calculations were performed by using the jun-cc-pVDZ basis set.

E_{elst}	E_{exch}	E_{disp}	E_{ind}	E_{total}	*Distance*
-74.80	52.42	-44.48	-18.73	-85.59	4.70
-69.97	41.24	-39.99	-17.12	-85.84	4.79
-63.02	26.12	-32.57	-14.58	-84.05	4.98
-58.35	17.02	-26.71	-12.65	-80.69	5.18
-55.02	11.40	-22.04	-11.12	-76.78	5.37
-51.41	6.48	-16.68	-9.30	-70.90	5.66
-47.18	2.59	-10.70	-7.05	-62.34	6.14
-44.03	0.98	-7.03	-5.44	-55.51	6.63
-41.47	0.34	-4.71	-4.25	-50.09	7.12
-39.26	0.11	-3.23	-3.37	-45.76	7.61
-37.30	0.03	-2.27	-2.70	-42.24	8.11
-35.52	0.01	-1.63	-2.18	-39.32	8.60
-33.90	0.00	-1.19	-1.79	-36.87	9.09
-32.40	0.00	-0.88	-1.47	-34.76	9.59
-31.03	0.00	-0.67	-1.23	-32.92	10.08
-29.75	0.00	-0.51	-1.03	-31.29	10.58
-28.57	0.00	-0.40	-0.87	-29.83	11.07
-27.47	0.00	-0.31	-0.74	-28.52	11.57

All energies are in kcal/mol, distances are in Å.

The geometries of the model systems shown in Figure S2d for ionic, hydrogen, and halogen bonds were generated using a coordinate scan (the distance between carbon atoms at the 4-positions of the aromatic groups was increased in 0.1 Å steps) in Gaussian 16 and are optimized with the distance constraint at the M06-2X/6-31+G(d,p) level of theory with the LANL2DZ basis set with the ECP being used for the iodine atom.[30–33] The SAPT0 calculations were performed by using the jun-cc-pVDZ basis set for the ionic and hydrogen-bonded systems, while for the halogen-bonded system, aug-cc-pVDZ and aug-cc-pVDZ-PP (for the I atom) were used. The distances used for the graphs are: between nitrogen and sulfur atoms for the ionic bond, between nitrogen and hydrogen (of the -OH group) atoms for the hydrogen-bond, and between nitrogen and iodine atoms for the halogen-bonded system.

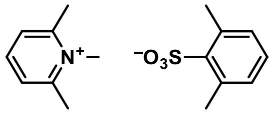

E_{elst}	E_{exch}	E_{disp}	E_{ind}	E_{total}	*Distance*
-86.41	25.07	-8.44	-12.26	-82.03	4.39
-85.34	23.08	-8.03	-11.79	-82.07	4.41
-83.91	20.71	-7.54	-11.23	-81.97	4.44
-82.31	18.27	-7.01	-10.64	-81.70	4.48
-80.68	15.99	-6.48	-10.04	-81.21	4.52
-78.98	13.87	-5.94	-9.43	-80.48	4.56
-77.24	11.96	-5.41	-8.84	-79.53	4.61
-75.66	10.47	-4.94	-8.29	-78.42	4.66

-74.22	9.31	-4.52	-7.79	-77.23	4.72
-72.70	8.02	-4.10	-7.31	-76.09	4.78
-71.24	6.84	-3.70	-6.84	-74.94	4.84
-69.78	5.71	-3.30	-6.36	-73.74	4.91
-68.01	4.24	-2.87	-5.85	-72.49	5.00
-66.31	3.09	-2.47	-5.38	-71.07	5.10
-64.70	2.20	-2.13	-4.93	-69.56	5.20
-63.17	1.55	-1.83	-4.53	-67.99	5.30
-61.76	1.09	-1.58	-4.17	-66.42	5.40
-60.44	0.76	-1.37	-3.85	-64.89	5.51
-59.20	0.53	-1.19	-3.56	-63.41	5.61
-58.04	0.37	-1.04	-3.30	-62.01	5.71
-56.94	0.26	-0.91	-3.07	-60.66	5.82
-55.90	0.19	-0.80	-2.86	-59.38	5.92
-54.90	0.13	-0.71	-2.67	-58.15	6.02
-53.95	0.09	-0.63	-2.50	-56.99	6.13
-53.04	0.07	-0.56	-2.34	-55.87	6.23
-52.16	0.05	-0.50	-2.19	-54.81	6.33
-51.31	0.03	-0.45	-2.06	-53.78	6.43
-50.49	0.02	-0.41	-1.94	-52.81	6.53
-49.70	0.02	-0.37	-1.82	-51.88	6.64
-48.94	0.01	-0.33	-1.72	-50.98	6.74
-48.20	0.01	-0.30	-1.62	-50.11	6.84
-47.49	0.01	-0.27	-1.53	-49.28	6.94
-46.80	0.00	-0.25	-1.45	-48.49	7.04
-46.13	0.00	-0.23	-1.37	-47.72	7.14
-45.47	0.00	-0.21	-1.29	-46.97	7.25
-44.84	0.00	-0.19	-1.23	-46.26	7.35
-44.23	0.00	-0.17	-1.16	-45.57	7.45
-43.63	0.00	-0.16	-1.10	-44.90	7.55
-43.05	0.00	-0.15	-1.05	-44.25	7.65
-42.49	0.00	-0.14	-1.00	-43.62	7.75
-41.94	0.00	-0.13	-0.95	-43.01	7.85
-41.41	0.00	-0.12	-0.90	-42.43	7.95
-40.88	0.00	-0.11	-0.86	-41.85	8.05
-40.38	0.00	-0.10	-0.82	-41.30	8.16
-39.88	0.00	-0.09	-0.78	-40.76	8.26
-39.40	0.00	-0.09	-0.75	-40.23	8.36
-38.93	0.00	-0.08	-0.71	-39.72	8.46
-38.47	0.00	-0.08	-0.68	-39.23	8.56
-38.02	0.00	-0.07	-0.65	-38.75	8.66
-37.58	0.00	-0.07	-0.63	-38.27	8.76
-37.16	0.00	-0.06	-0.60	-37.82	8.86
-36.74	0.00	-0.06	-0.57	-37.37	8.96

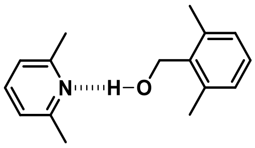

E_{elst}	E_{exch}	E_{disp}	E_{ind}	E_{total}	*Distance*
-15.04	17.31	-6.56	-5.54	-9.82	1.88
-14.62	16.47	-6.26	-5.37	-9.78	1.89
-13.86	14.99	-5.81	-5.02	-9.70	1.91
-12.98	13.24	-5.25	-4.60	-9.59	1.94
-11.61	10.93	-4.68	-3.91	-9.28	2.00
-10.23	8.73	-4.14	-3.23	-8.87	2.07
-8.91	6.78	-3.64	-2.62	-8.38	2.14
-7.73	5.16	-3.18	-2.09	-7.84	2.23
-6.65	3.82	-2.77	-1.64	-7.24	2.32
-5.71	2.78	-2.40	-1.28	-6.62	2.42
-4.91	1.99	-2.08	-1.00	-6.00	2.53
-4.23	1.40	-1.80	-0.77	-5.40	2.63
-3.66	0.98	-1.56	-0.60	-4.84	2.75
-3.18	0.68	-1.36	-0.47	-4.32	2.86
-2.79	0.48	-1.19	-0.37	-3.87	2.97
-2.46	0.33	-1.04	-0.30	-3.47	3.08
-2.19	0.23	-0.92	-0.24	-3.11	3.19
-1.95	0.16	-0.81	-0.20	-2.80	3.30
-1.76	0.12	-0.72	-0.17	-2.53	3.40
-1.59	0.08	-0.65	-0.14	-2.29	3.51
-1.44	0.06	-0.58	-0.12	-2.08	3.62
-1.31	0.04	-0.52	-0.10	-1.89	3.72
-1.19	0.03	-0.47	-0.09	-1.72	3.83
-1.09	0.02	-0.42	-0.07	-1.57	3.93
-1.00	0.01	-0.38	-0.06	-1.44	4.04
-0.92	0.01	-0.35	-0.06	-1.32	4.14
-0.85	0.01	-0.32	-0.05	-1.21	4.24
-0.79	0.01	-0.29	-0.04	-1.12	4.35
-0.73	0.00	-0.26	-0.04	-1.03	4.45
-0.68	0.00	-0.24	-0.03	-0.95	4.56
-0.63	0.00	-0.22	-0.03	-0.88	4.66
-0.59	0.00	-0.20	-0.03	-0.82	4.76
-0.55	0.00	-0.19	-0.02	-0.76	4.87
-0.51	0.00	-0.17	-0.02	-0.71	4.97
-0.48	0.00	-0.16	-0.02	-0.66	5.07
-0.45	0.00	-0.15	-0.02	-0.62	5.18
-0.42	0.00	-0.14	-0.02	-0.58	5.28
-0.40	0.00	-0.13	-0.01	-0.54	5.38
-0.38	0.00	-0.12	-0.01	-0.51	5.49
-0.35	0.00	-0.11	-0.01	-0.48	5.59
-0.34	0.00	-0.10	-0.01	-0.45	5.69
-0.32	0.00	-0.09	-0.01	-0.42	5.79

-0.30	0.00	-0.09	-0.01	-0.40	5.90
-0.28	0.00	-0.08	-0.01	-0.37	6.00
-0.27	0.00	-0.08	-0.01	-0.35	6.10

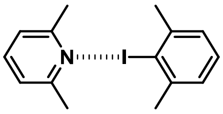

E_{elst}	E_{exch}	E_{disp}	E_{ind}	E_{total}	*Distance*
-7.71	12.05	-7.48	-2.67	-5.81	3.12
-6.74	10.23	-6.89	-2.32	-5.72	3.18
-5.47	7.92	-6.11	-1.85	-5.50	3.27
-4.40	6.06	-5.40	-1.46	-5.20	3.36
-3.62	4.63	-4.69	-1.15	-4.83	3.46
-2.92	3.48	-4.13	-0.91	-4.47	3.56
-2.34	2.59	-3.63	-0.71	-4.09	3.66
-1.88	1.92	-3.19	-0.56	-3.71	3.76
-1.52	1.43	-2.81	-0.44	-3.35	3.86
-1.23	1.05	-2.48	-0.35	-3.01	3.96
-1.00	0.77	-2.18	-0.28	-2.70	4.07
-0.81	0.57	-1.93	-0.23	-2.41	4.17
-0.67	0.41	-1.71	-0.19	-2.15	4.27
-0.55	0.30	-1.52	-0.15	-1.92	4.37
-0.45	0.23	-1.36	-0.13	-1.71	4.48
-0.38	0.16	-1.21	-0.10	-1.52	4.58
-0.31	0.12	-1.08	-0.09	-1.36	4.68
-0.26	0.09	-0.97	-0.07	-1.21	4.78
-0.21	0.07	-0.87	-0.06	-1.08	4.88
-0.17	0.05	-0.79	-0.05	-0.96	4.99
-0.14	0.05	-0.73	-0.04	-0.87	5.12
-0.11	0.05	-0.68	-0.04	-0.78	5.25
-0.08	0.04	-0.63	-0.03	-0.70	5.37
-0.06	0.07	-0.65	-0.02	-0.66	5.61
-0.03	0.12	-0.75	-0.02	-0.67	6.30
-0.02	0.09	-0.65	-0.01	-0.60	6.37
0.01	0.07	-0.62	-0.01	-0.55	6.49
0.02	0.05	-0.55	-0.01	-0.50	6.60
0.02	0.03	-0.49	-0.01	-0.45	6.70
0.02	0.02	-0.44	-0.01	-0.40	6.78
0.02	0.02	-0.40	0.00	-0.36	6.88
0.03	0.01	-0.36	0.00	-0.32	6.98
0.00	0.01	-0.27	0.00	-0.26	7.14
0.01	0.01	-0.26	0.00	-0.25	7.21
0.01	0.00	-0.24	0.00	-0.23	7.29
0.01	0.00	-0.20	0.00	-0.19	7.44

All energies are in kcal/mol, distances are in Å.

3 Supplementary Data

3.1 NMR spectra

^{1}H NMR (400 MHz, $CDCl_3$) of **C2_N**

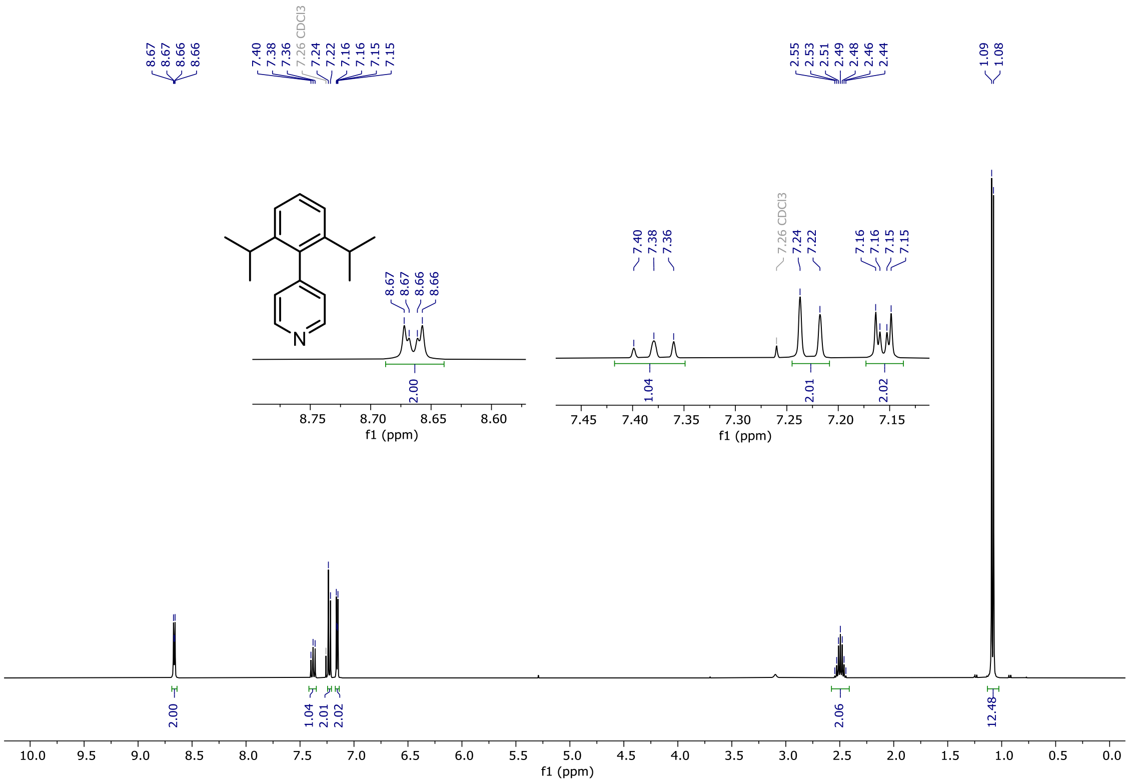

^{13}C NMR (101 MHz, $CDCl_3$) of **C2_N**

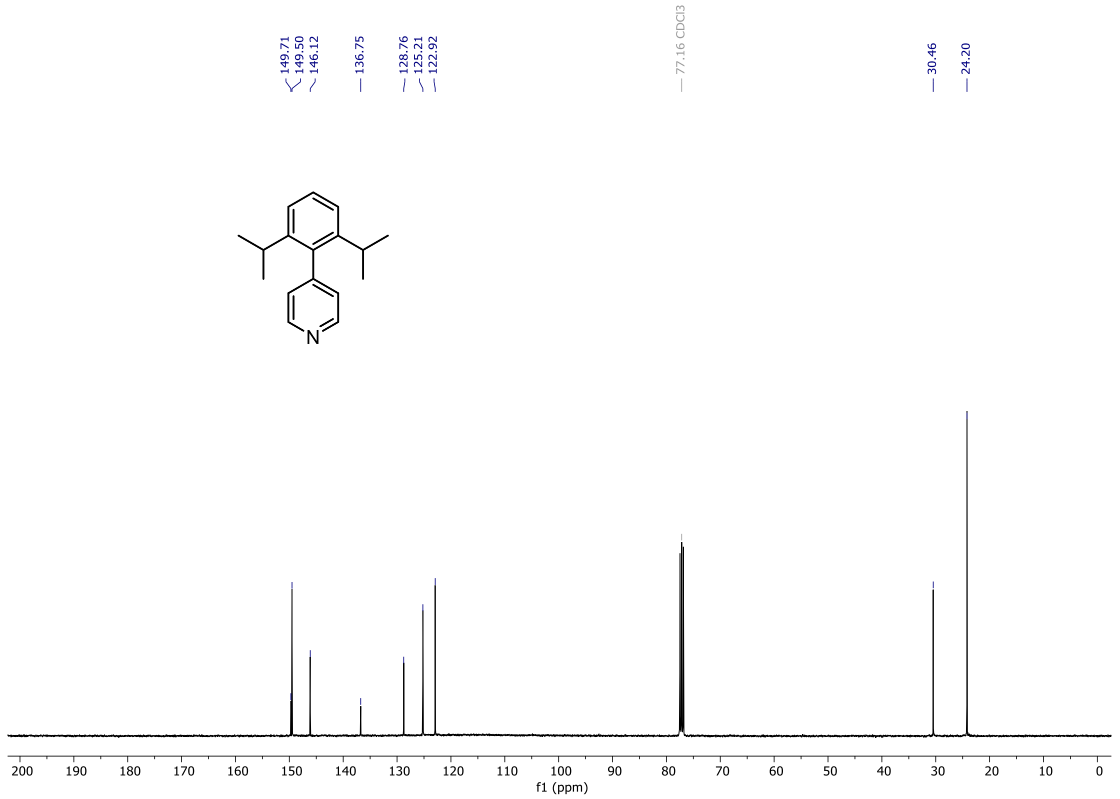

^{1}H NMR (400 MHz, CDCl$_3$) of **C2^{+}BF$_4^{-}$**

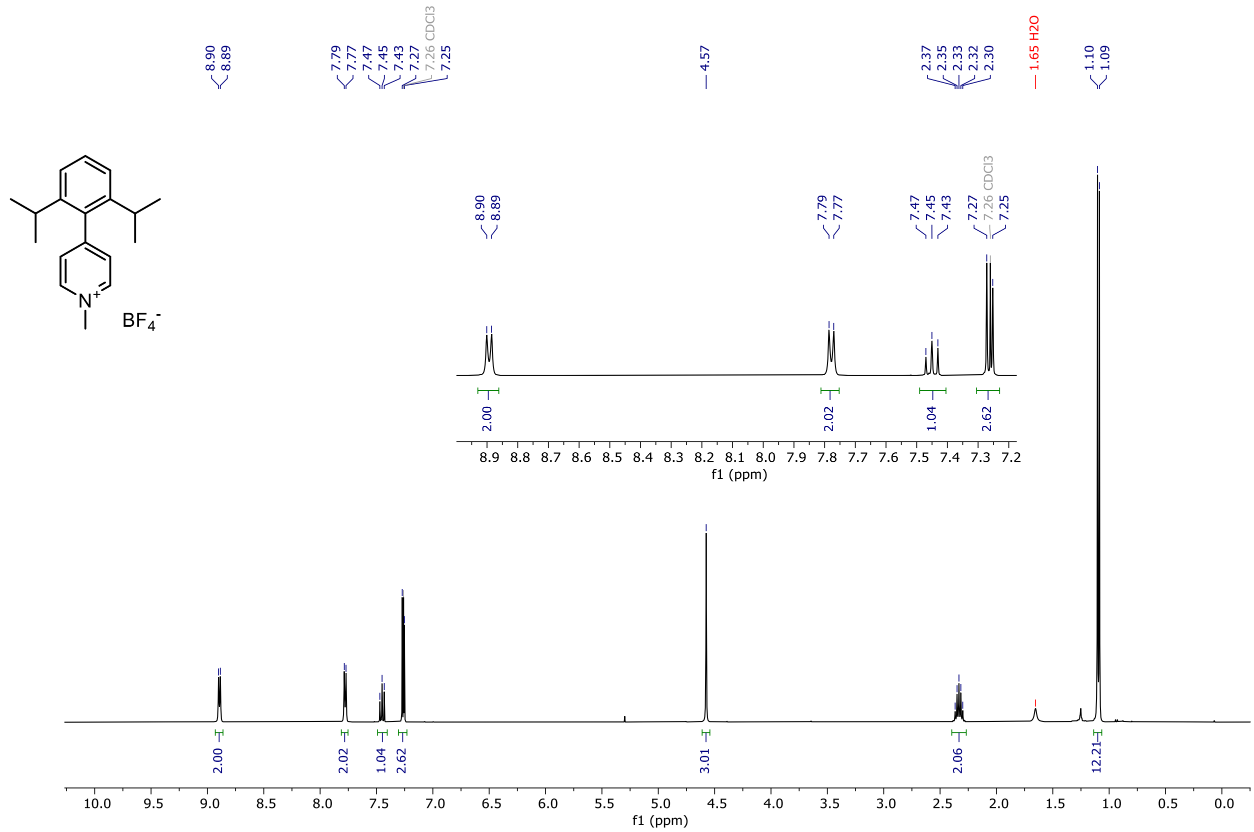

^{13}C NMR (101 MHz, CDCl$_3$) of **C2^{+}BF$_4^{-}$**

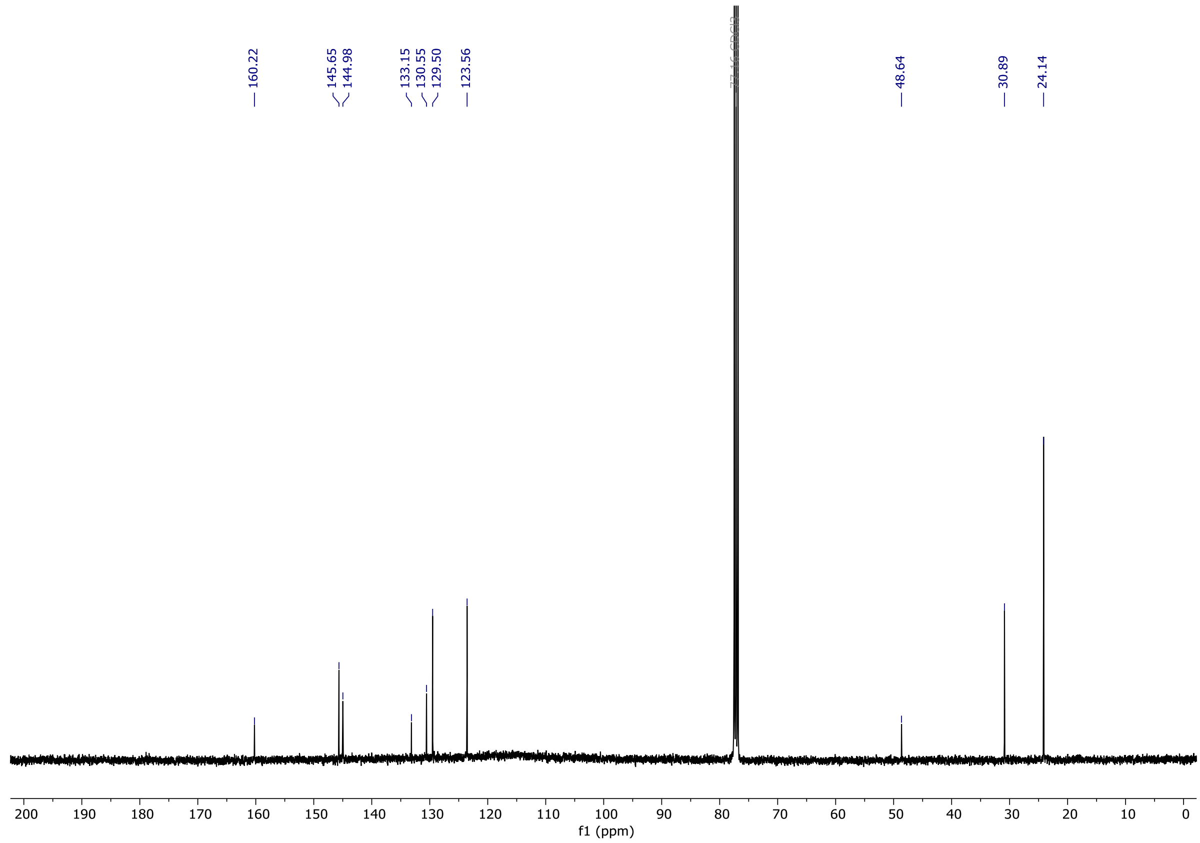

^{19}F NMR (376 MHz, $CDCl_3$) of **$C2^{+}BF_4^{-}$**

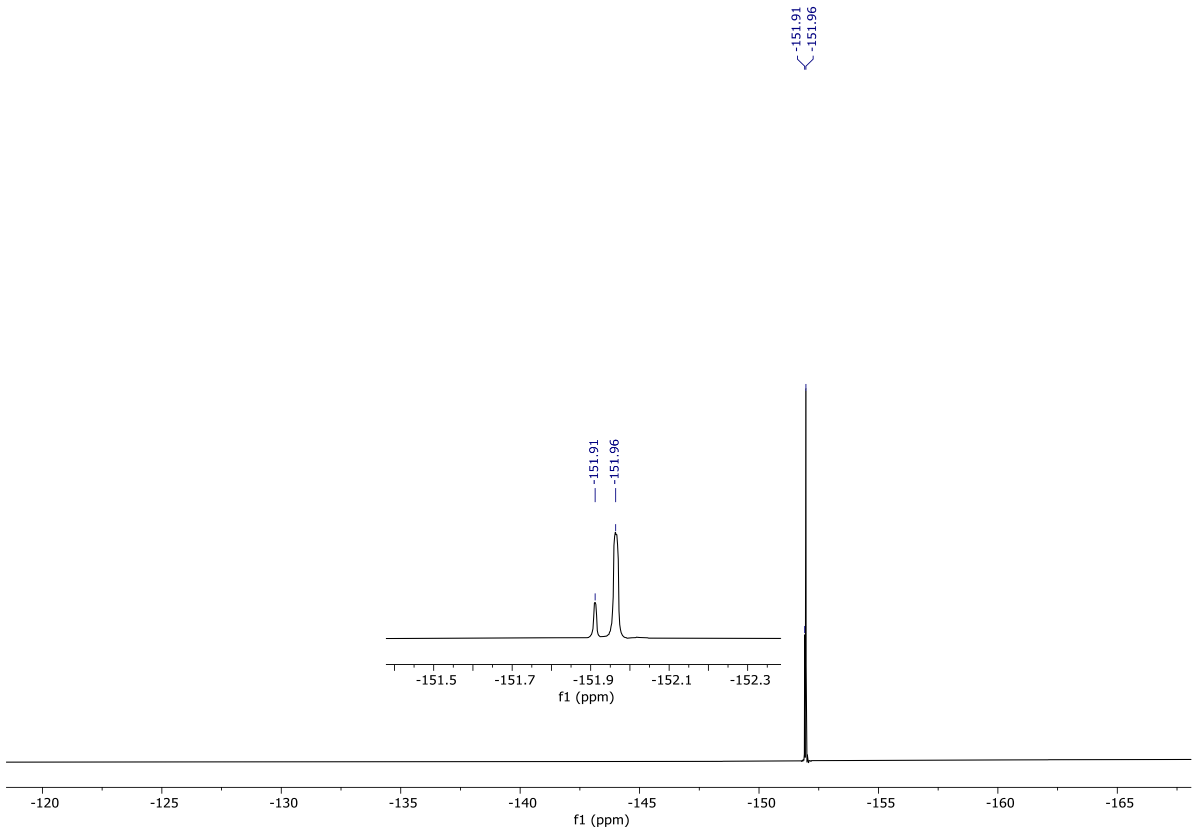

^{1}H NMR (400 MHz, $CDCl_3$) of **C3_N**

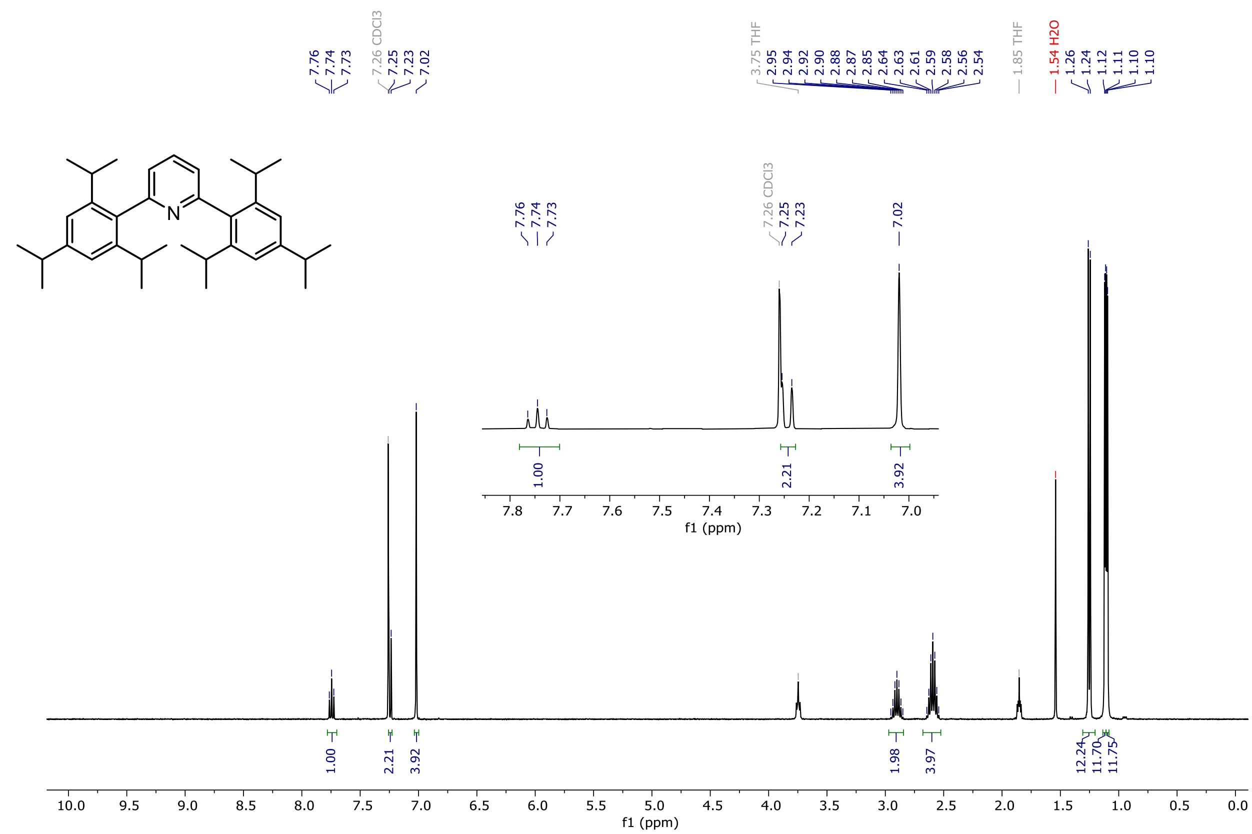

^{13}C NMR (101 MHz, $CDCl_3$) of **C3_N**

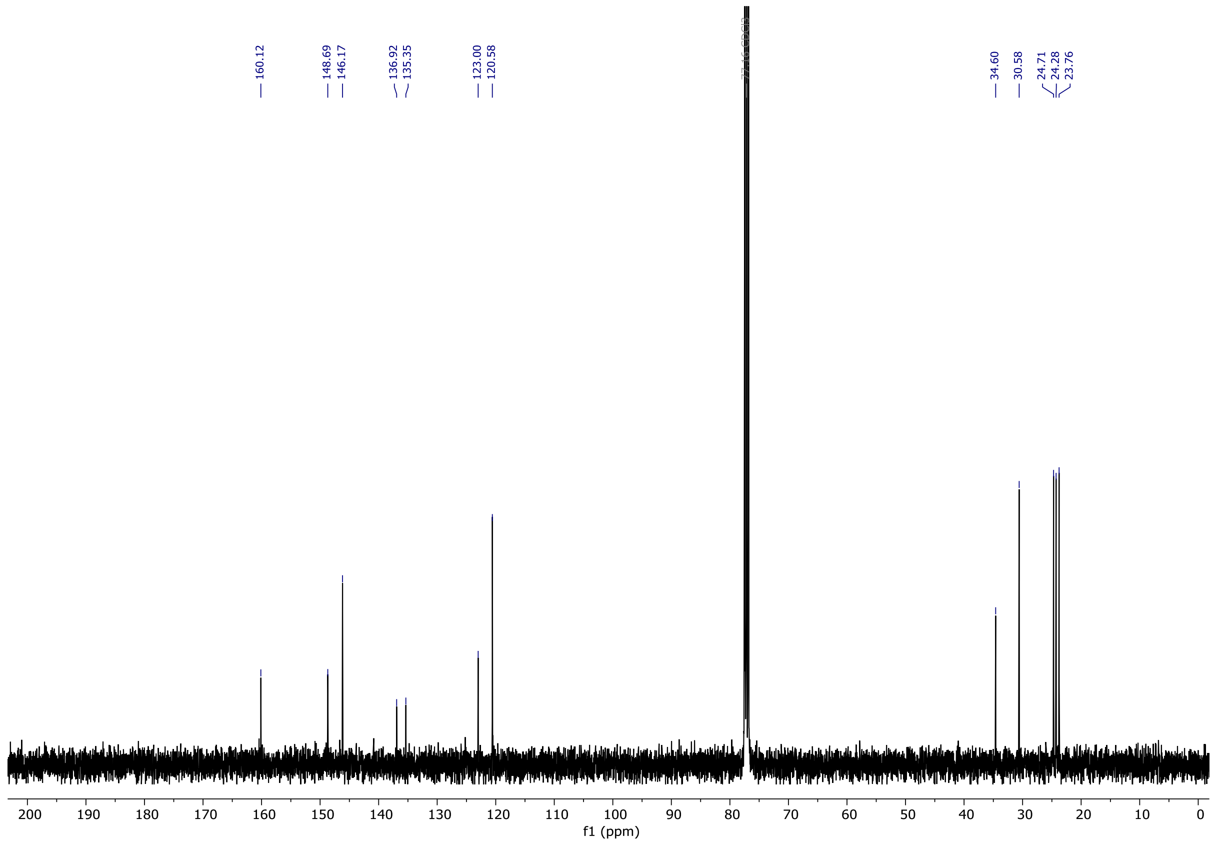

^{1}H NMR (400 MHz, $CDCl_3$) of **C3⁺BF₄⁻**

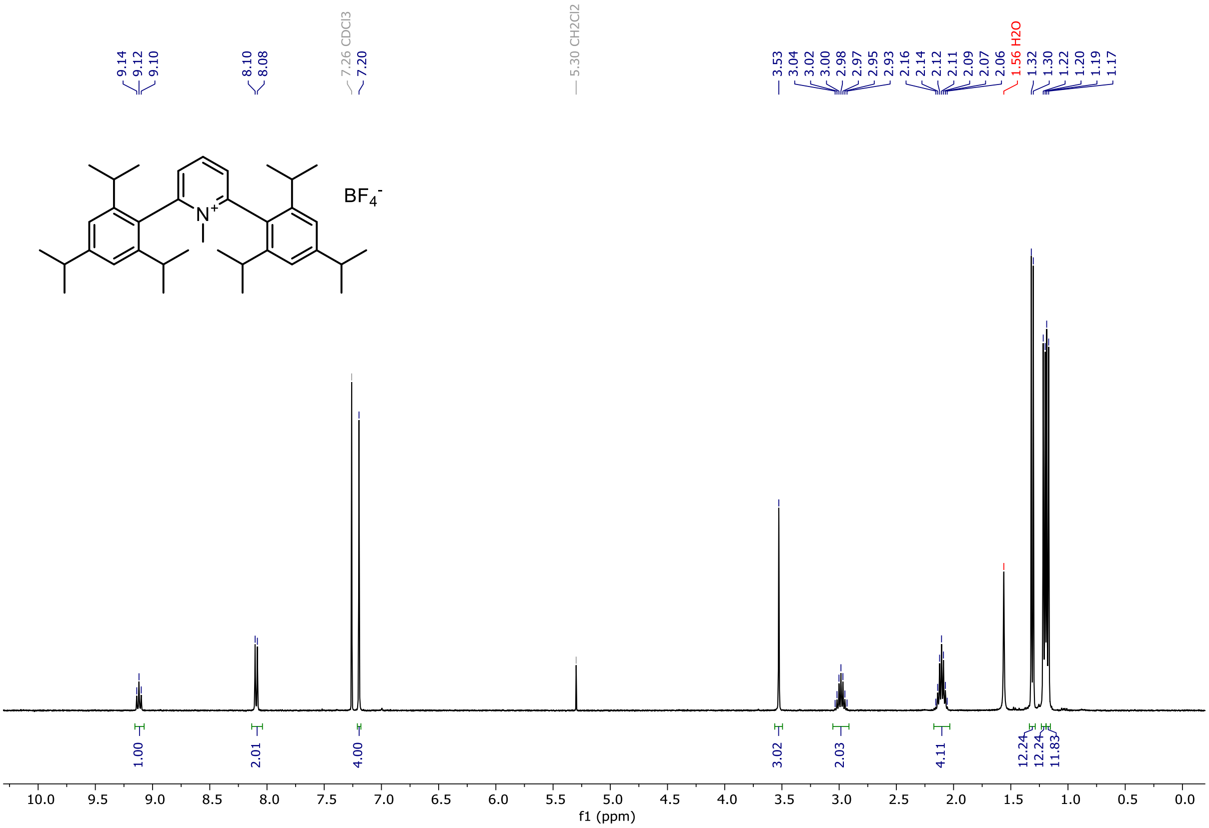

^{13}C NMR (101 MHz, $CDCl_3$) of **C3⁺BF₄⁻**

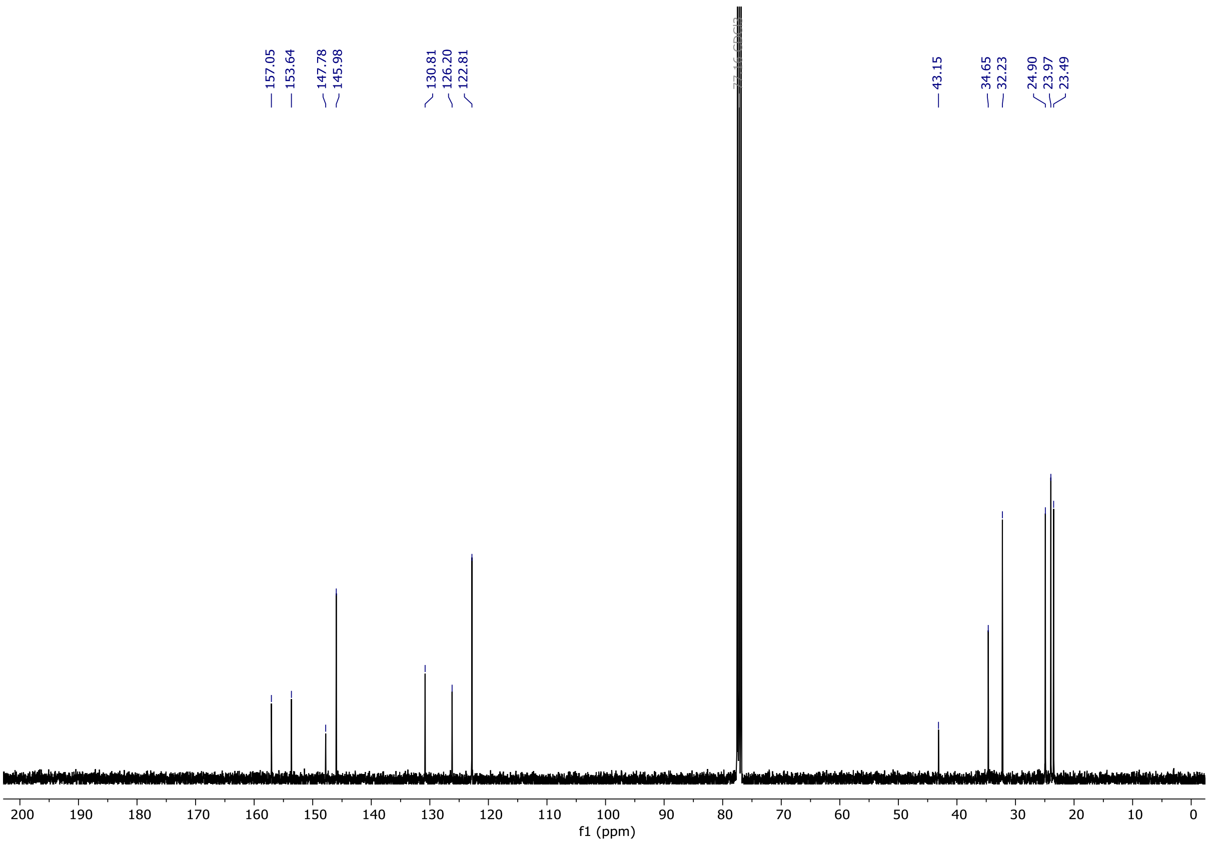

^{19}F NMR (376 MHz, $CDCl_3$) of **C3$^+$BF$_4^-$**

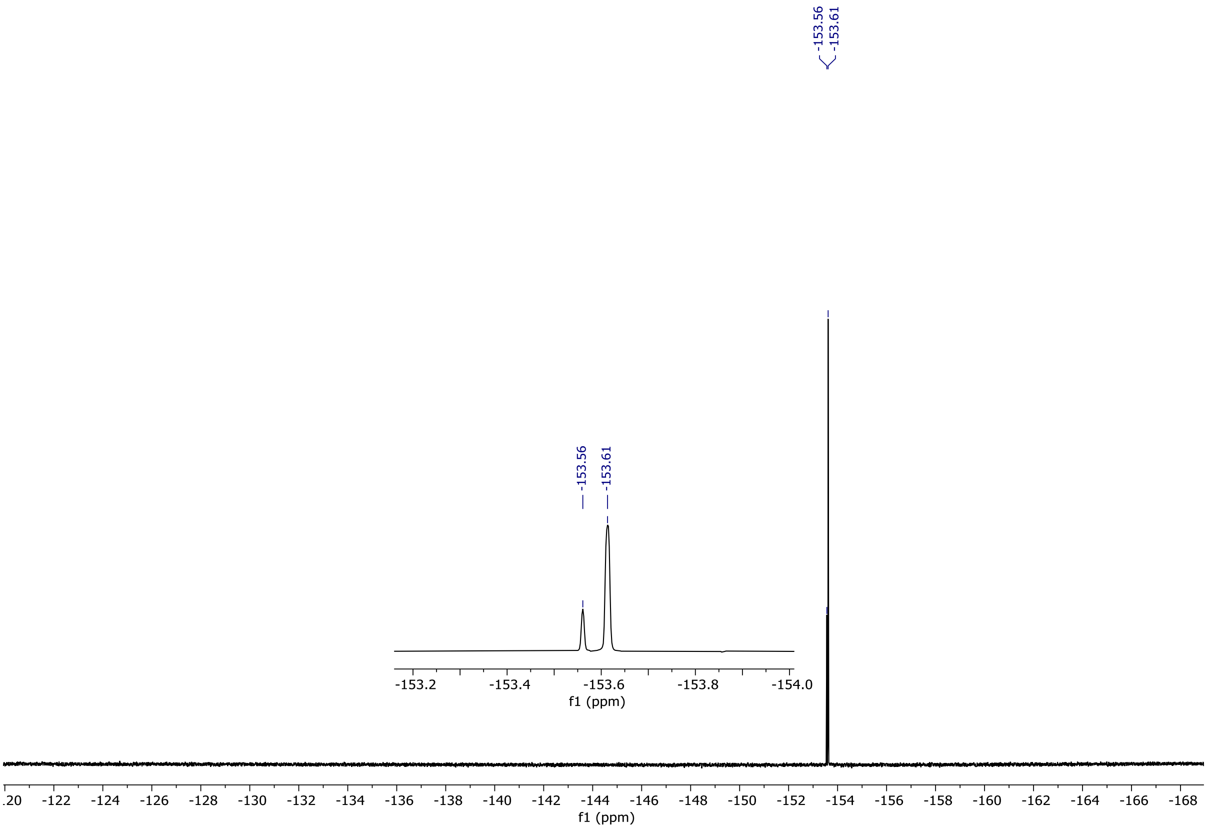

^{1}H NMR (400 MHz, $CDCl_3$) of **A2_Br**

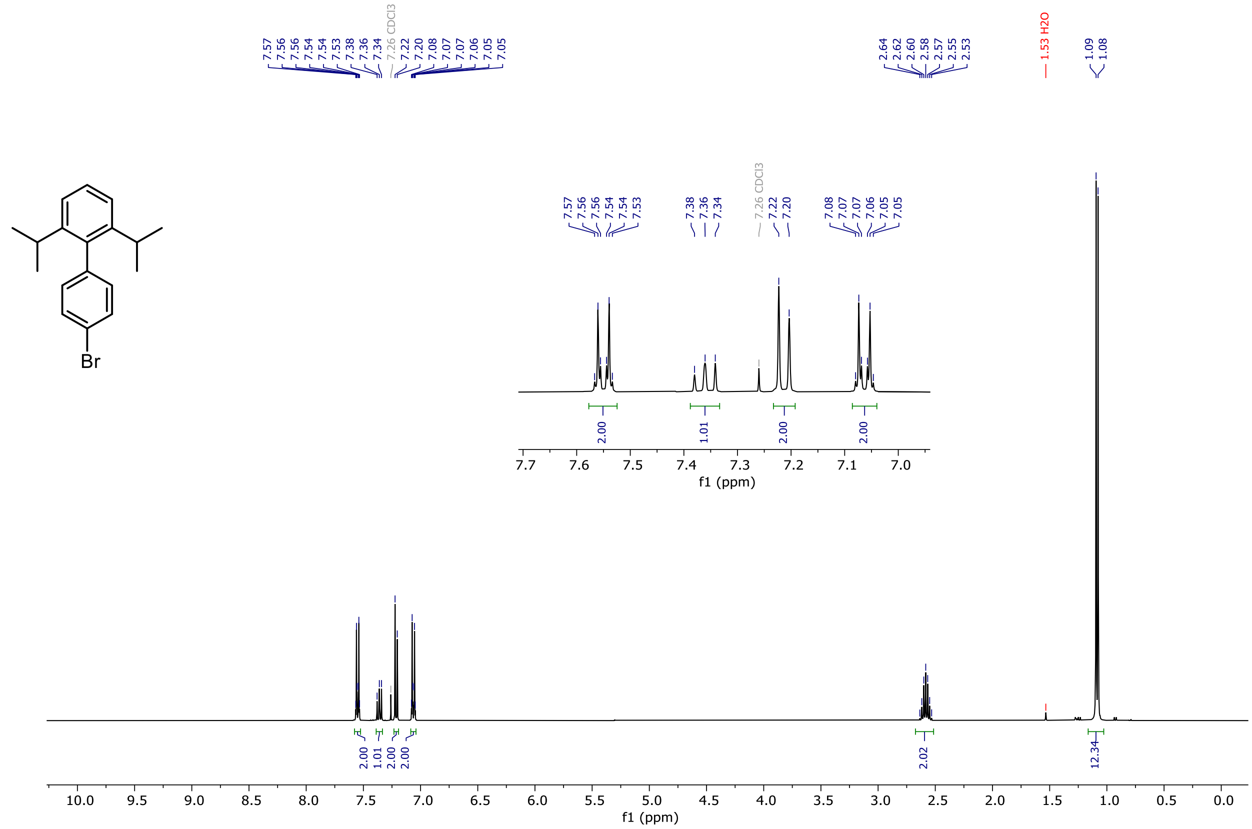

^{13}C NMR (101 MHz, $CDCl_3$) of **A2_Br**

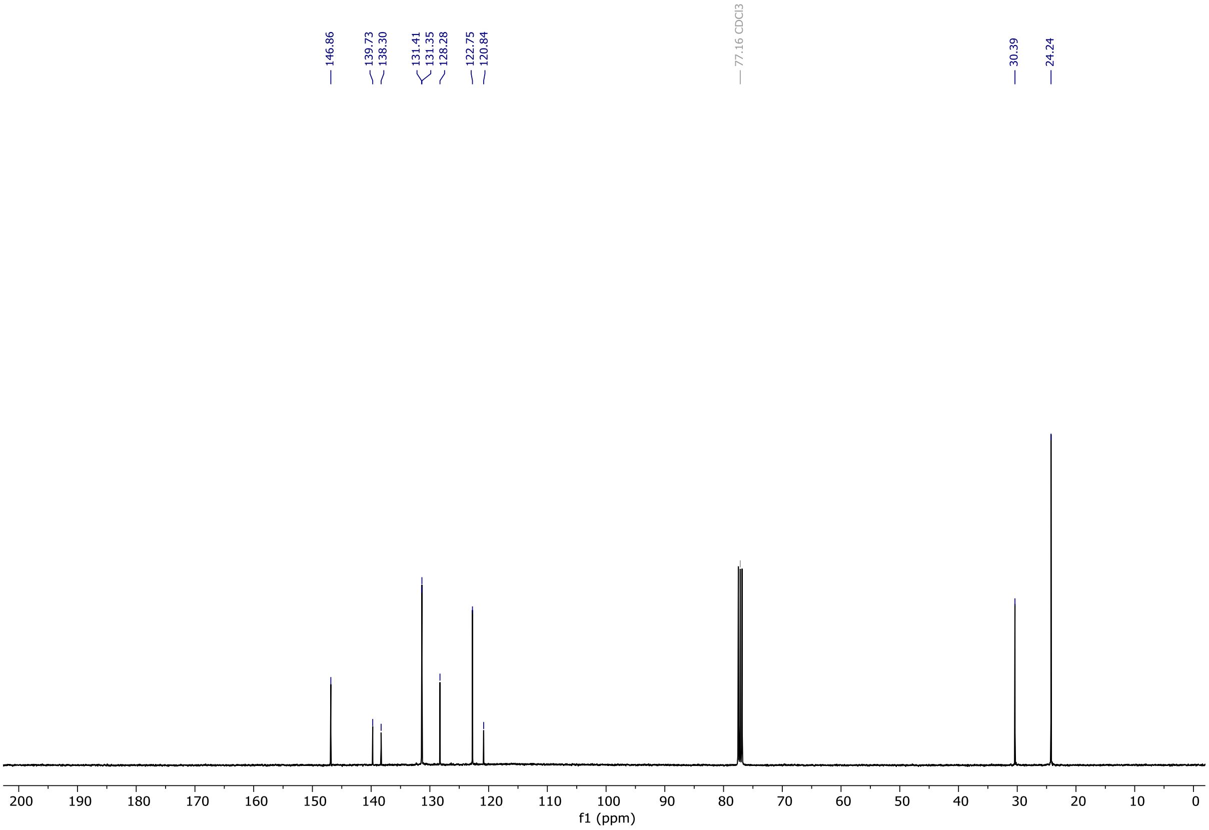

^1H NMR (400 MHz, $CDCl_3$) of **A2_SO₂Cl**

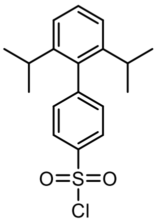

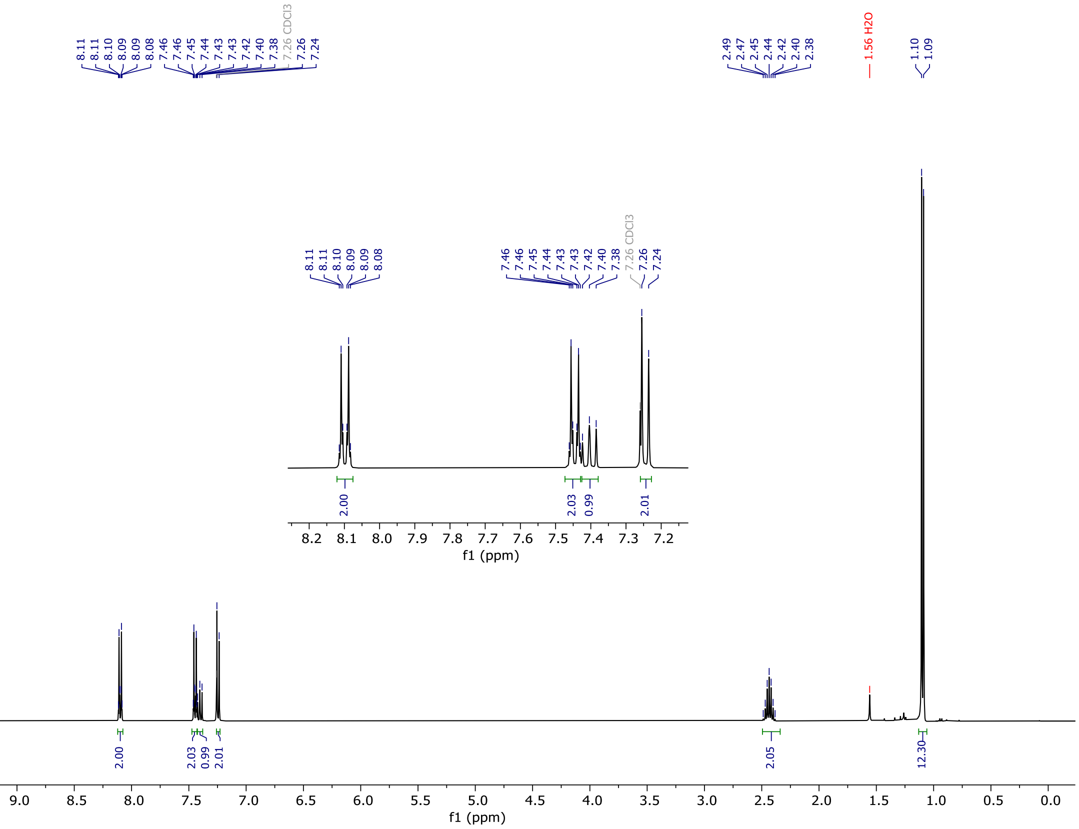

^{13}C NMR (101 MHz, $CDCl_3$) of **A2_SO₂Cl**

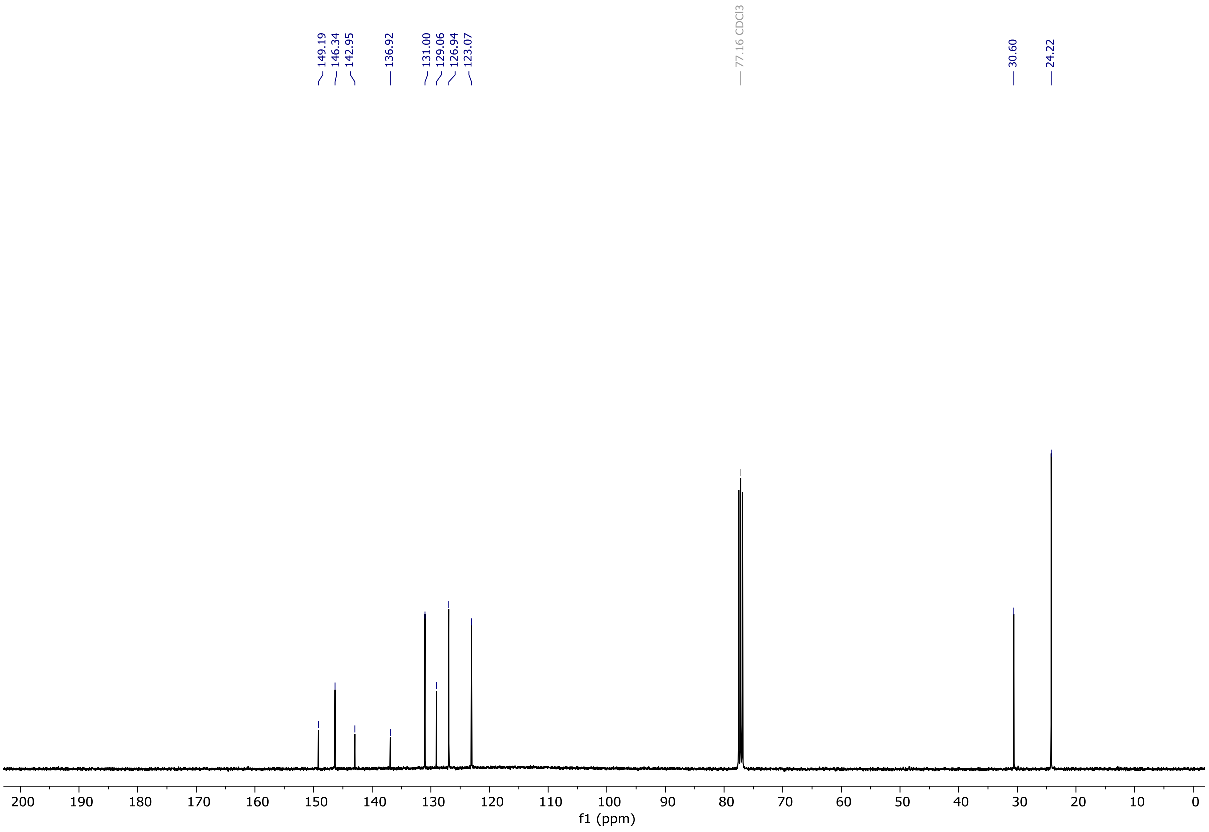

^{1}H NMR (500 MHz, CD_3OD) of **Na^{+}A2^{-}**

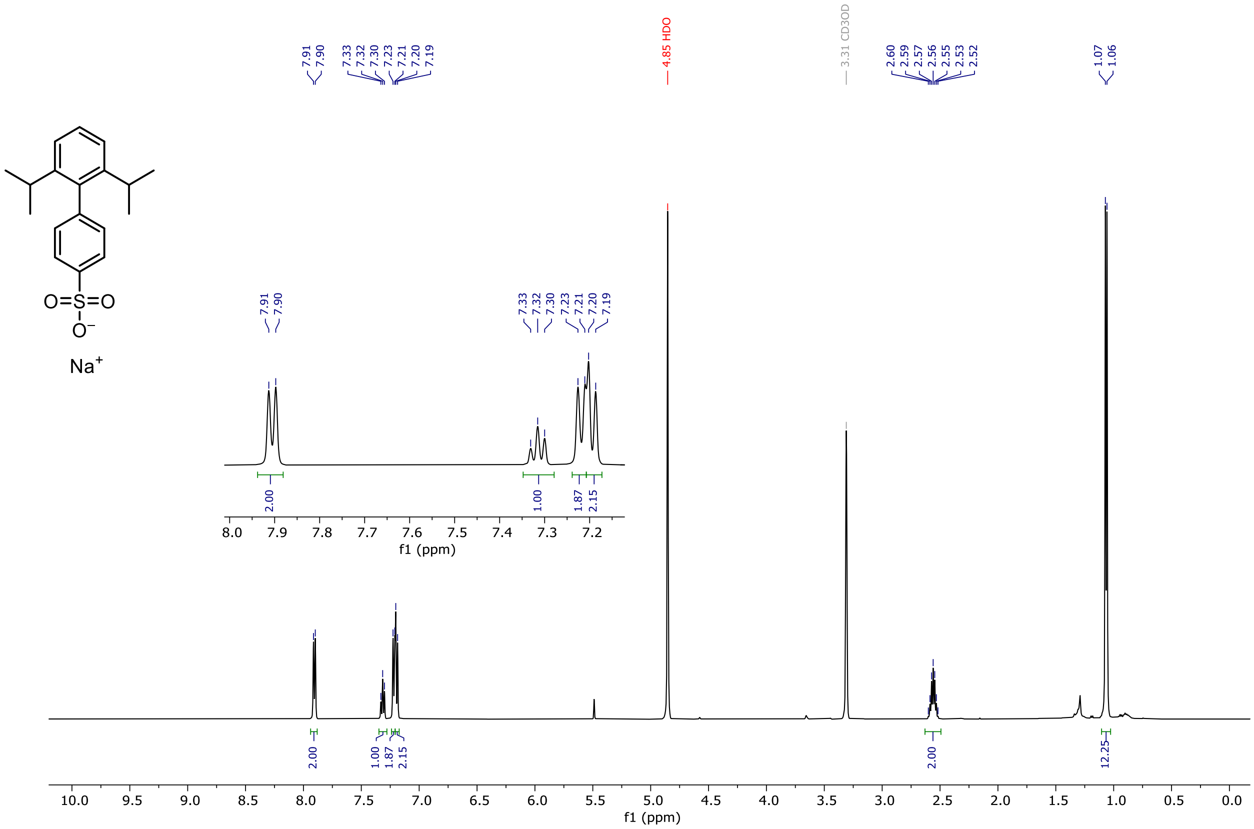

^{13}C NMR (126 MHz, CD_3OD) of **Na^{+}A2^{-}**

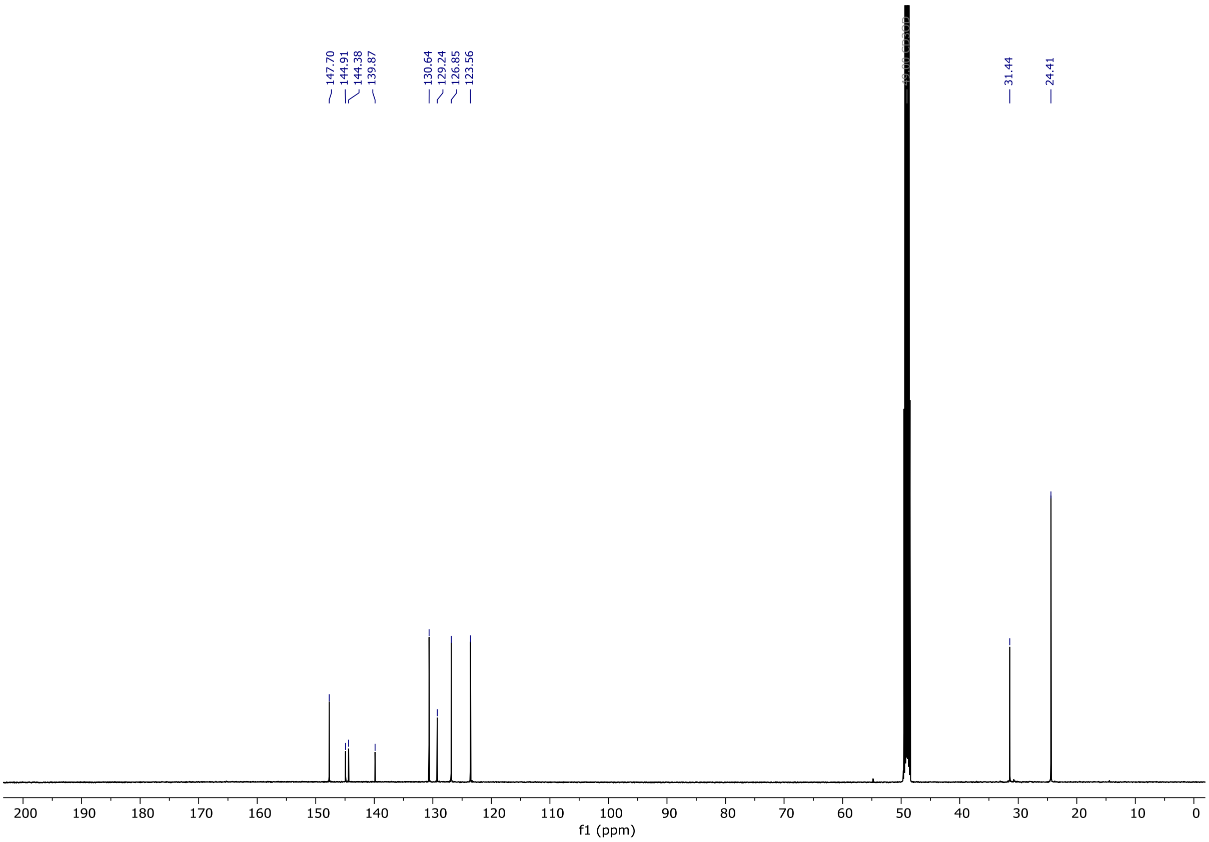

^{1}H NMR (400 MHz, $CDCl_3$) of **A3_Br**

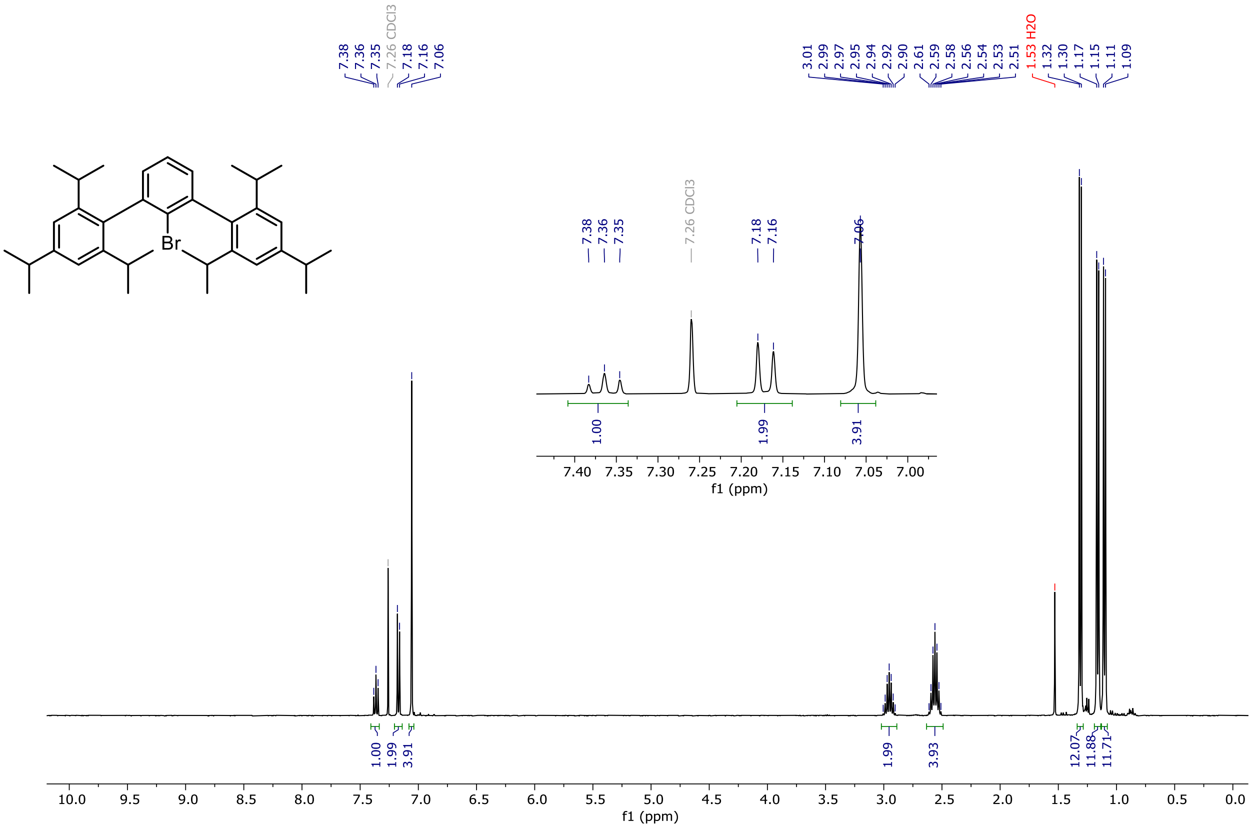

^{13}C NMR (101 MHz, $CDCl_3$) of **A3_Br**

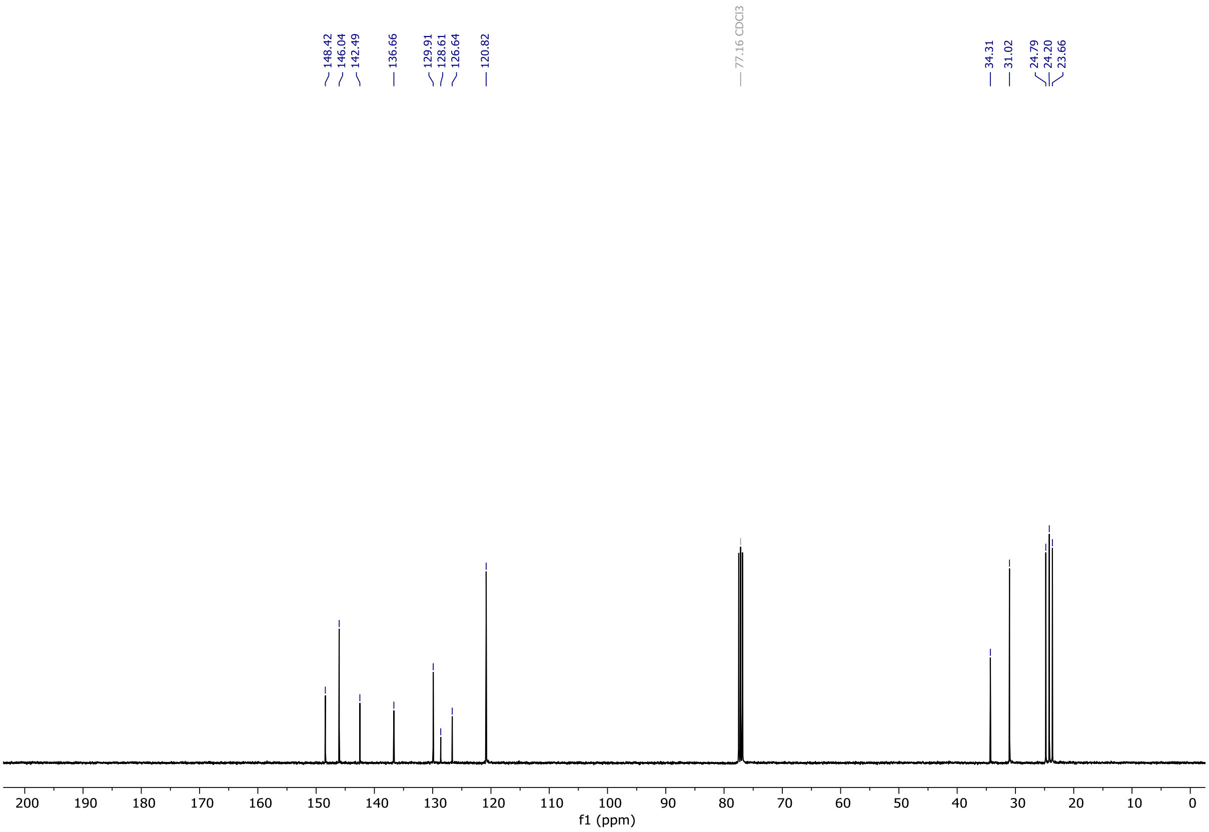

^{1}H NMR (400 MHz, $CDCl_3$) of **A3_SO₂Cl**

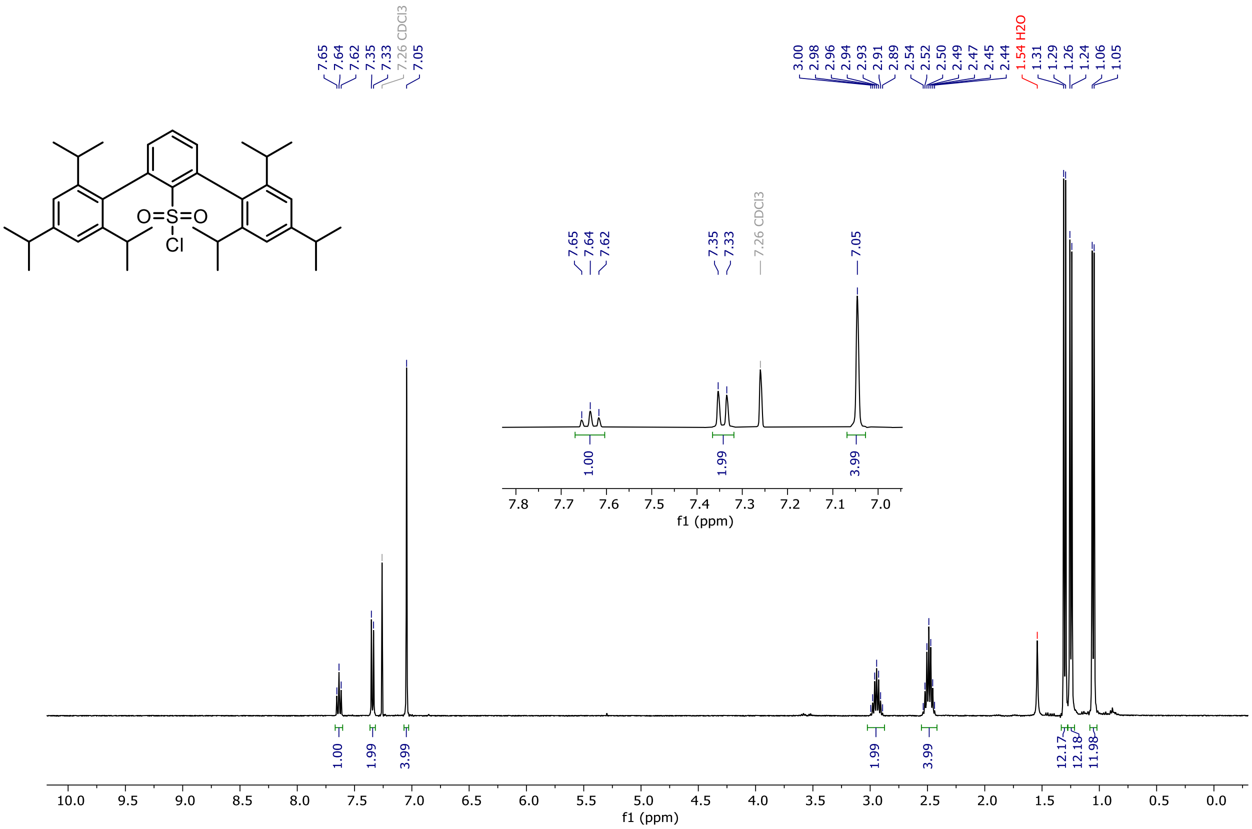

^{13}C NMR (101 MHz, $CDCl_3$) of **A3_SO₂Cl**

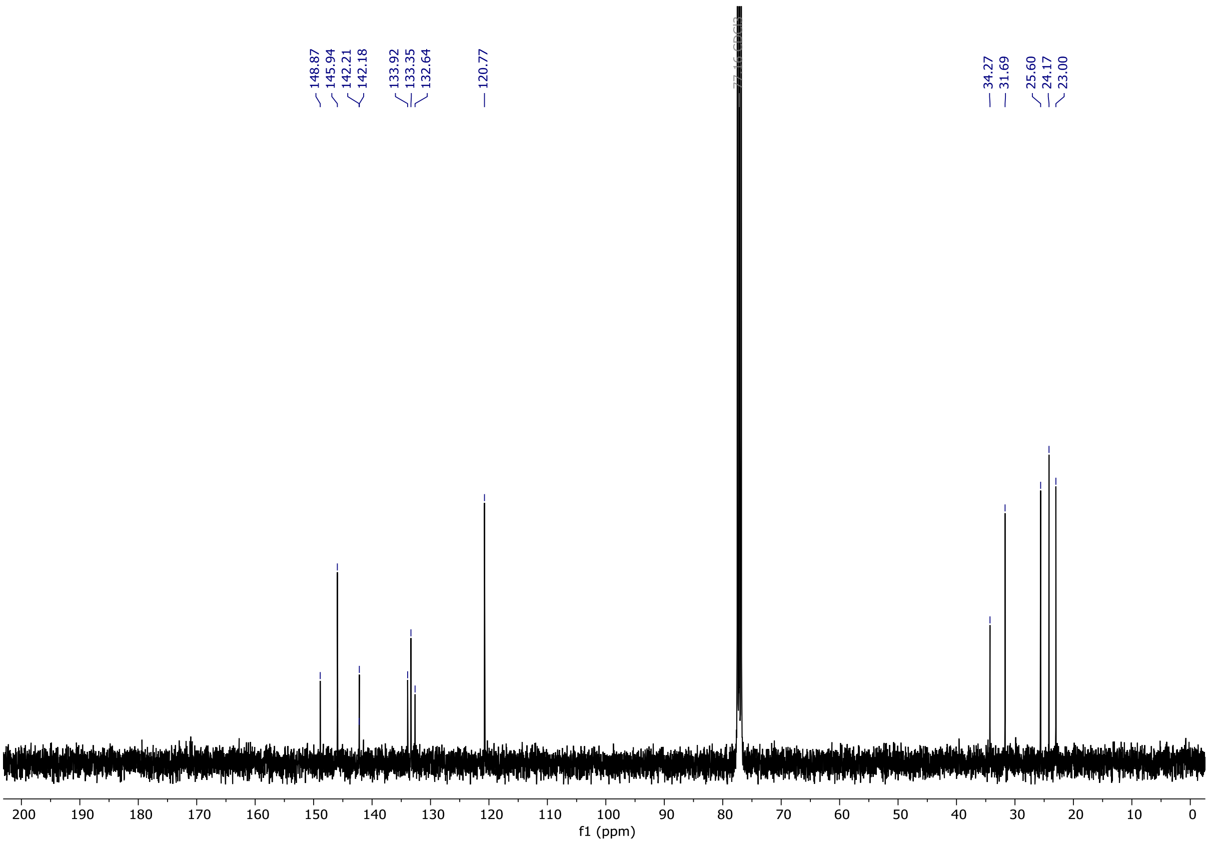

^{1}H NMR (400 MHz, $CDCl_3$) of **Na^{+}A3^{-}**

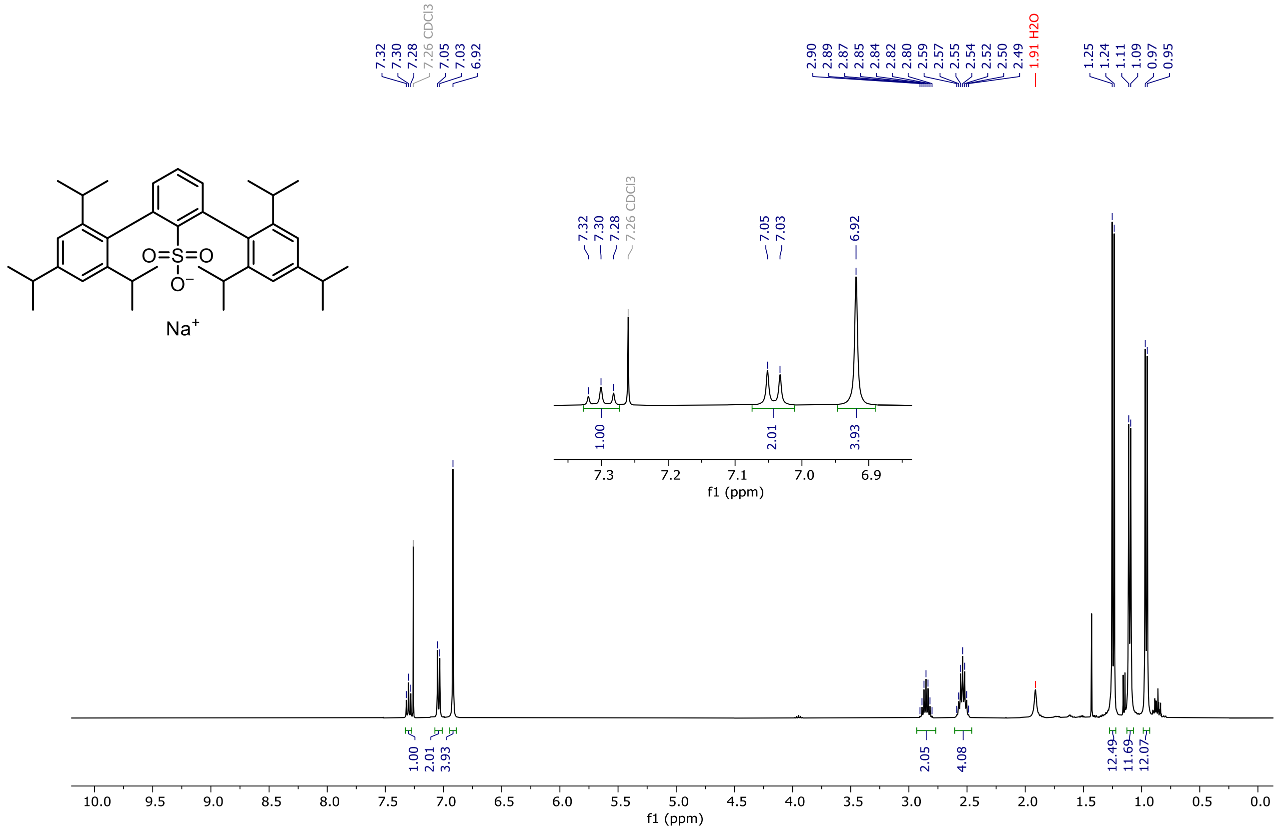

^{13}C NMR (101 MHz, $CDCl_3$) of **Na^{+}A3^{-}**

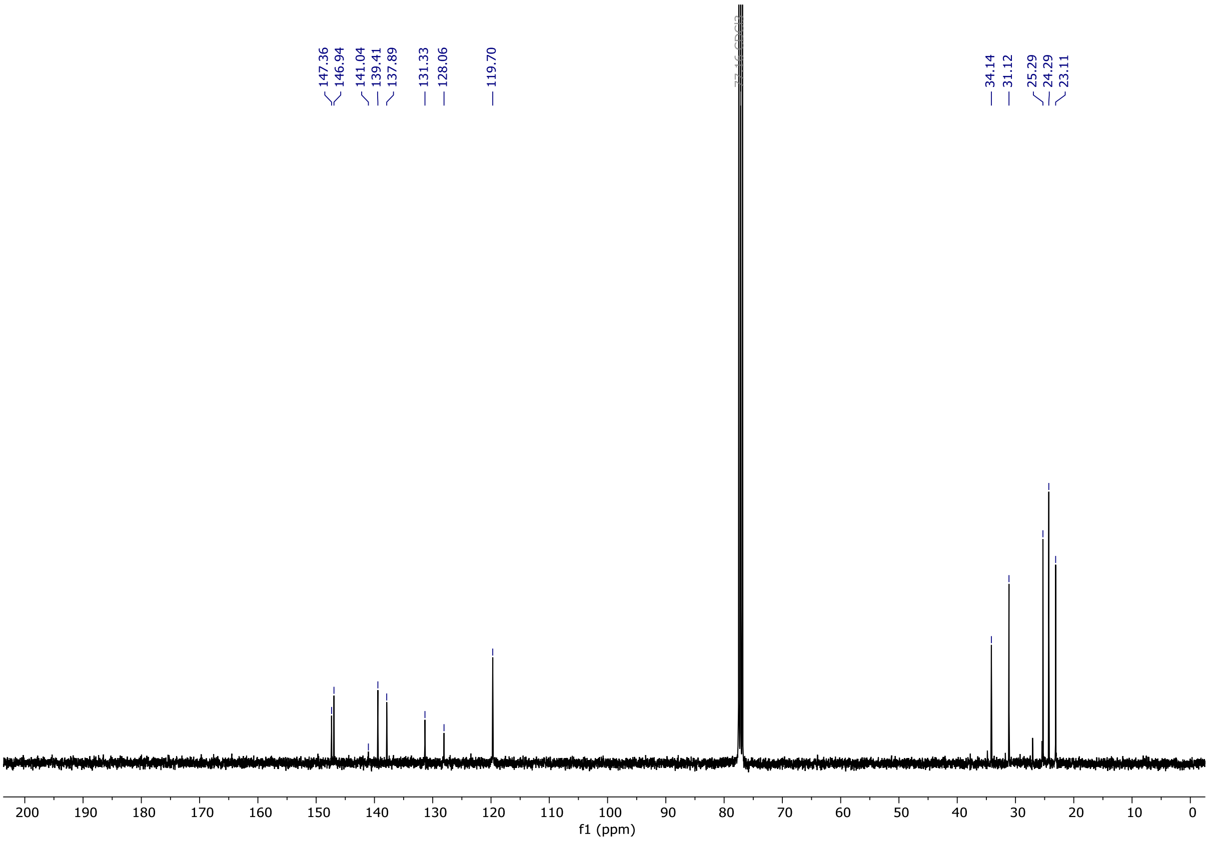

^{1}H NMR (500 MHz, $CDCl_3$) of 2,6-dichloro-4-(2,6-diisopropylphenyl)pyridine

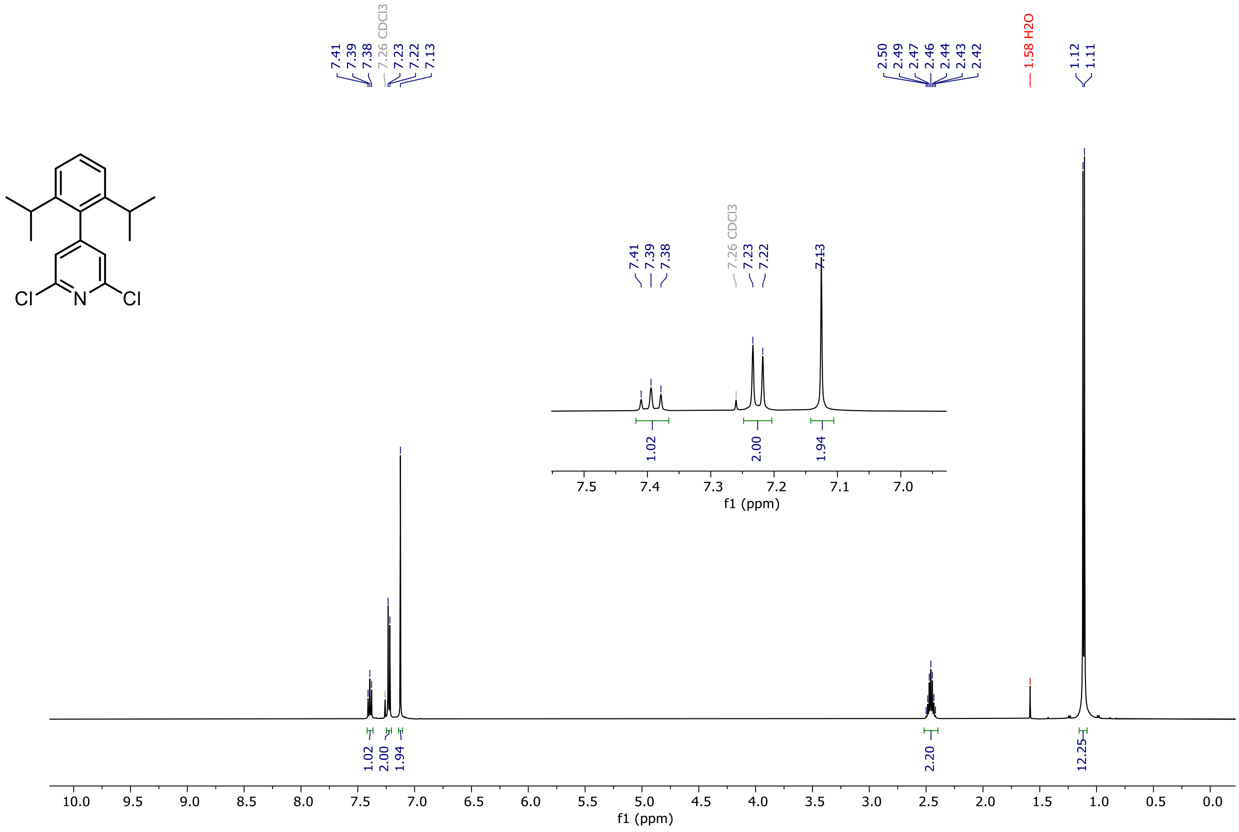

^{13}C NMR (126 MHz, $CDCl_3$) of 2,6-dichloro-4-(2,6-diisopropylphenyl)pyridine

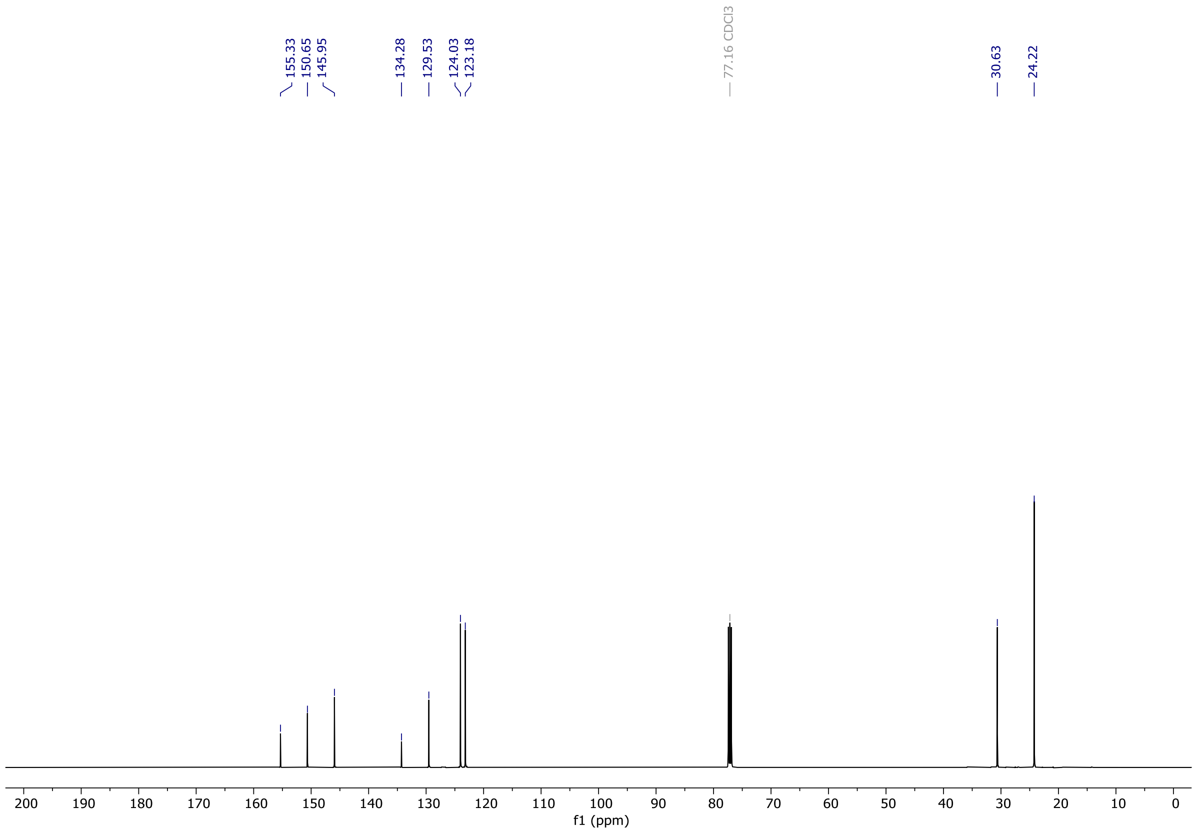

^{1}H NMR (500 MHz, $CDCl_3$) of **C4_N**

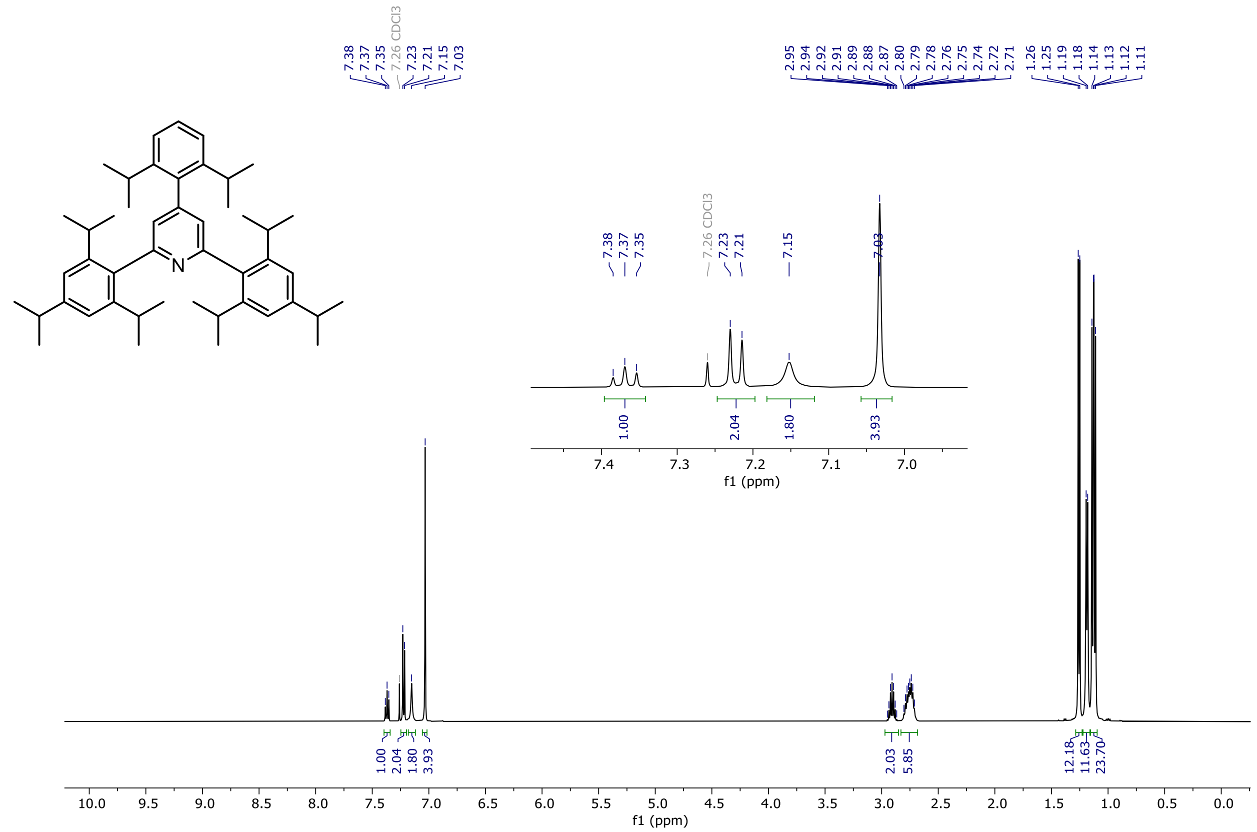

^{13}C NMR (126 MHz, $CDCl_3$) of **C4_N**

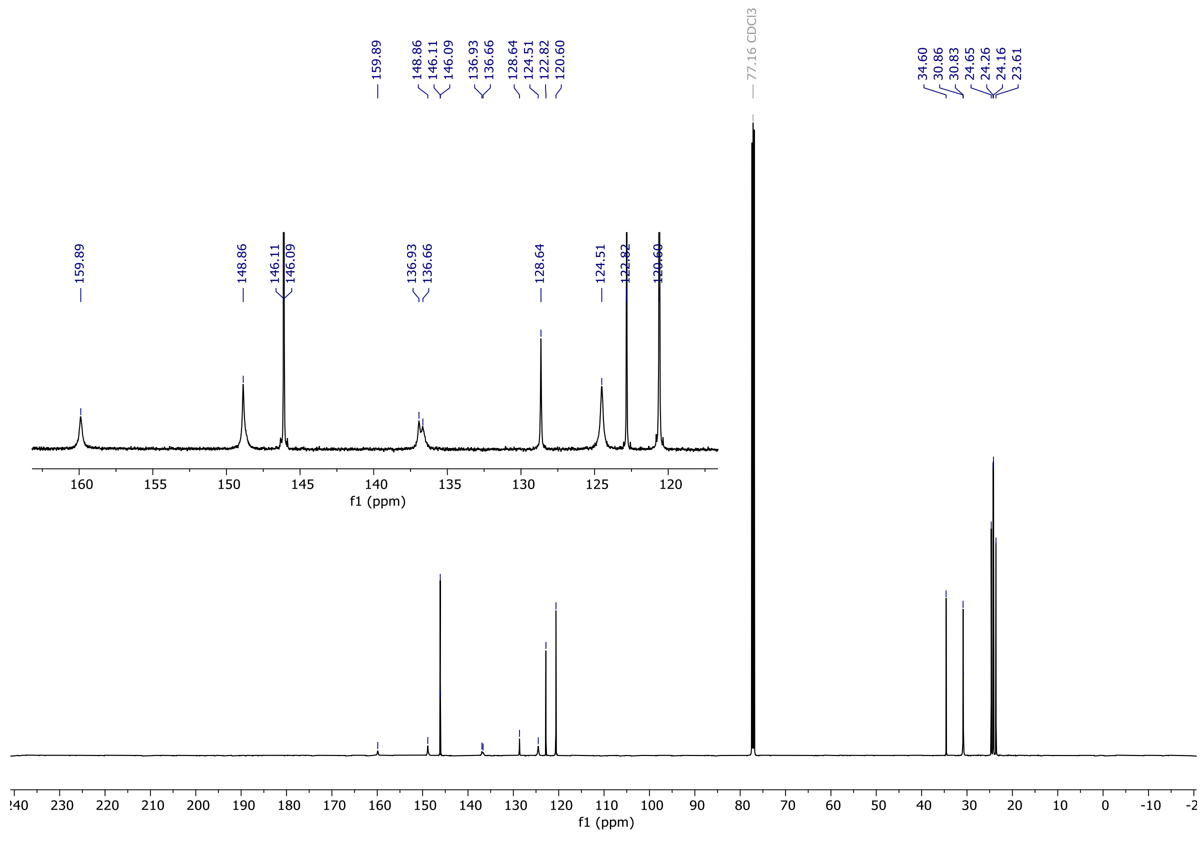

^{1}H NMR (500 MHz, $CDCl_3$) of **C4^{+}BF$_4^{-}$**

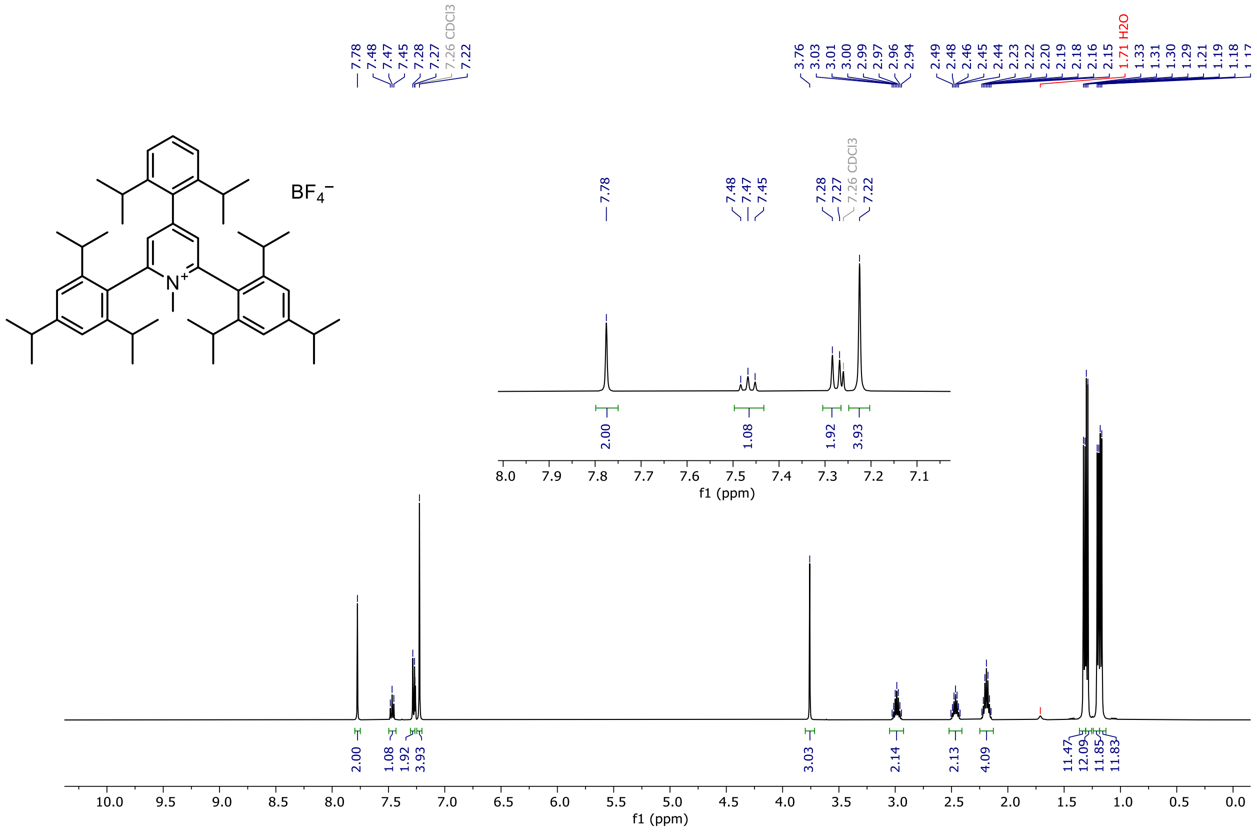

^{13}C NMR (126 MHz, $CDCl_3$) of **C4^{+}BF$_4^{-}$**

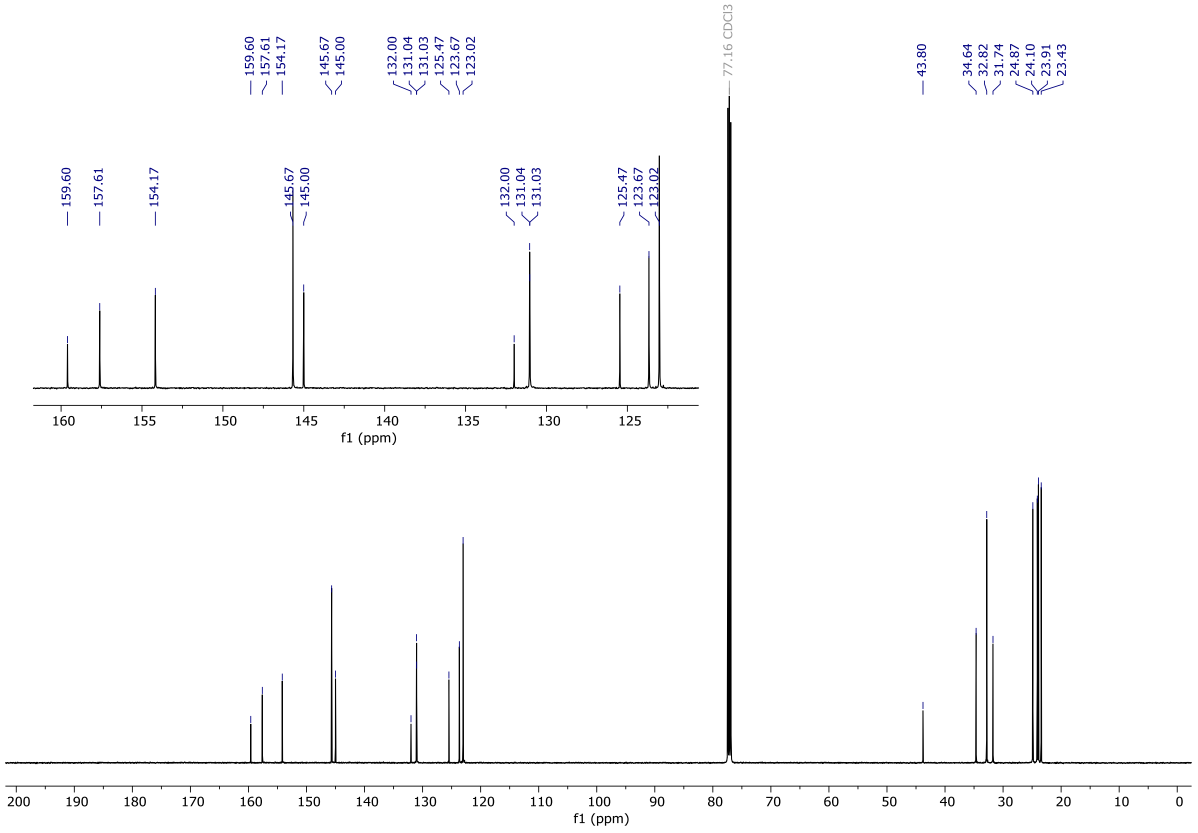

^{19}F NMR (471 MHz, $CDCl_3$) of **$C4^{+}BF_4^{-}$**

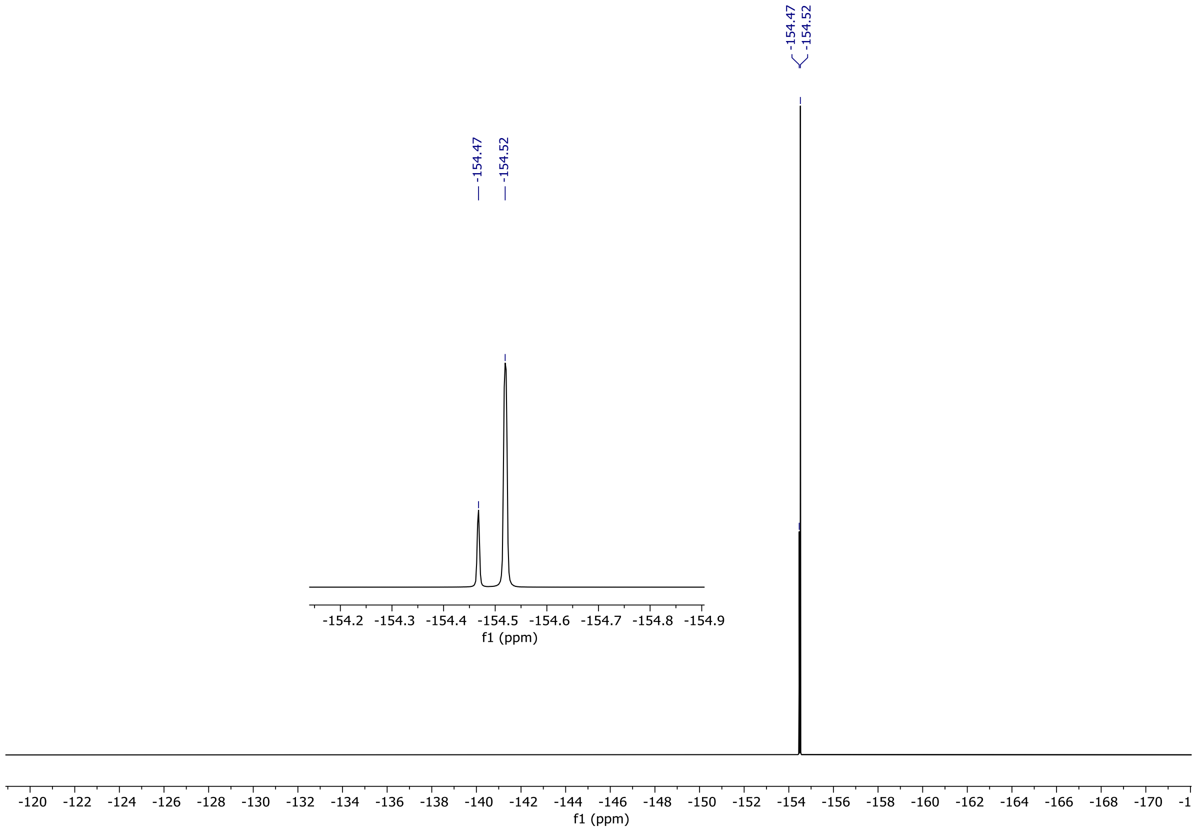

^{1}H NMR (400 MHz, $CDCl_3$) of **C5_N**

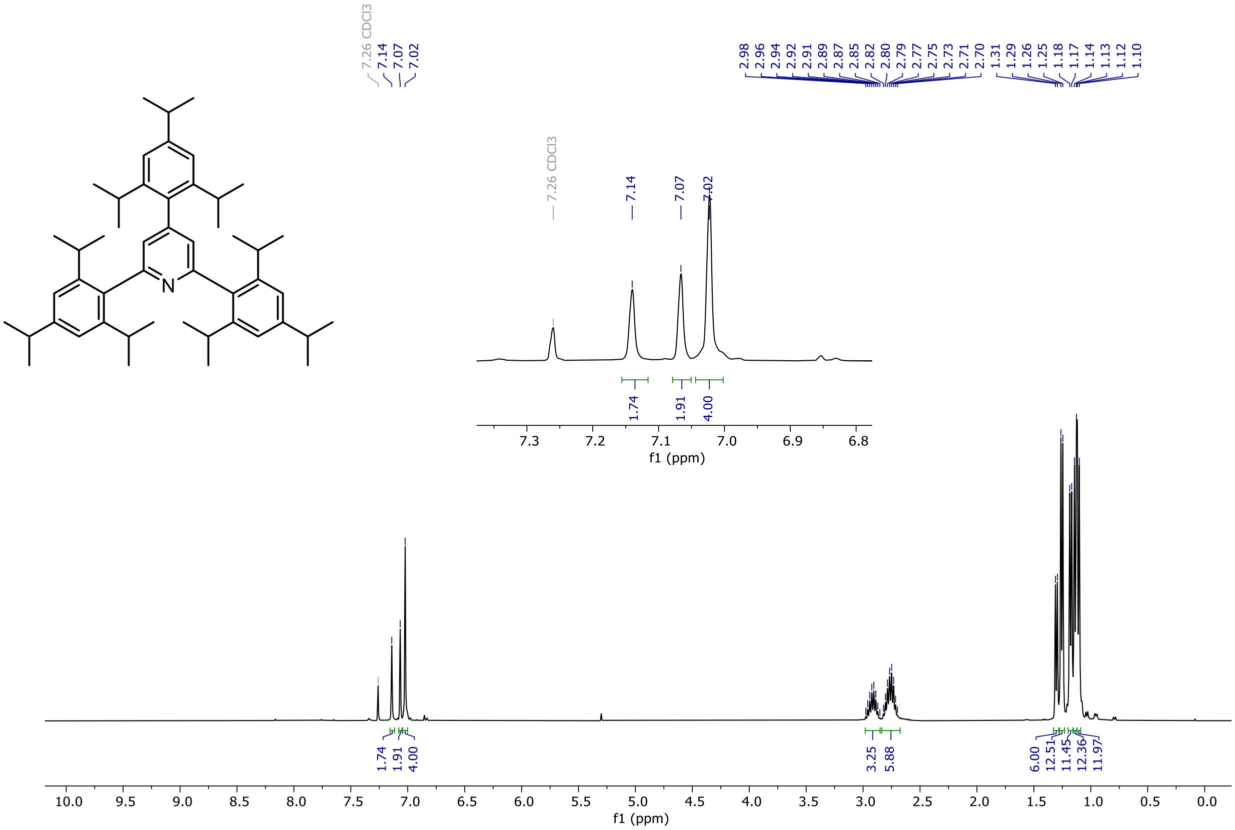

^{13}C NMR (101 MHz, $CDCl_3$) of **C5_N**

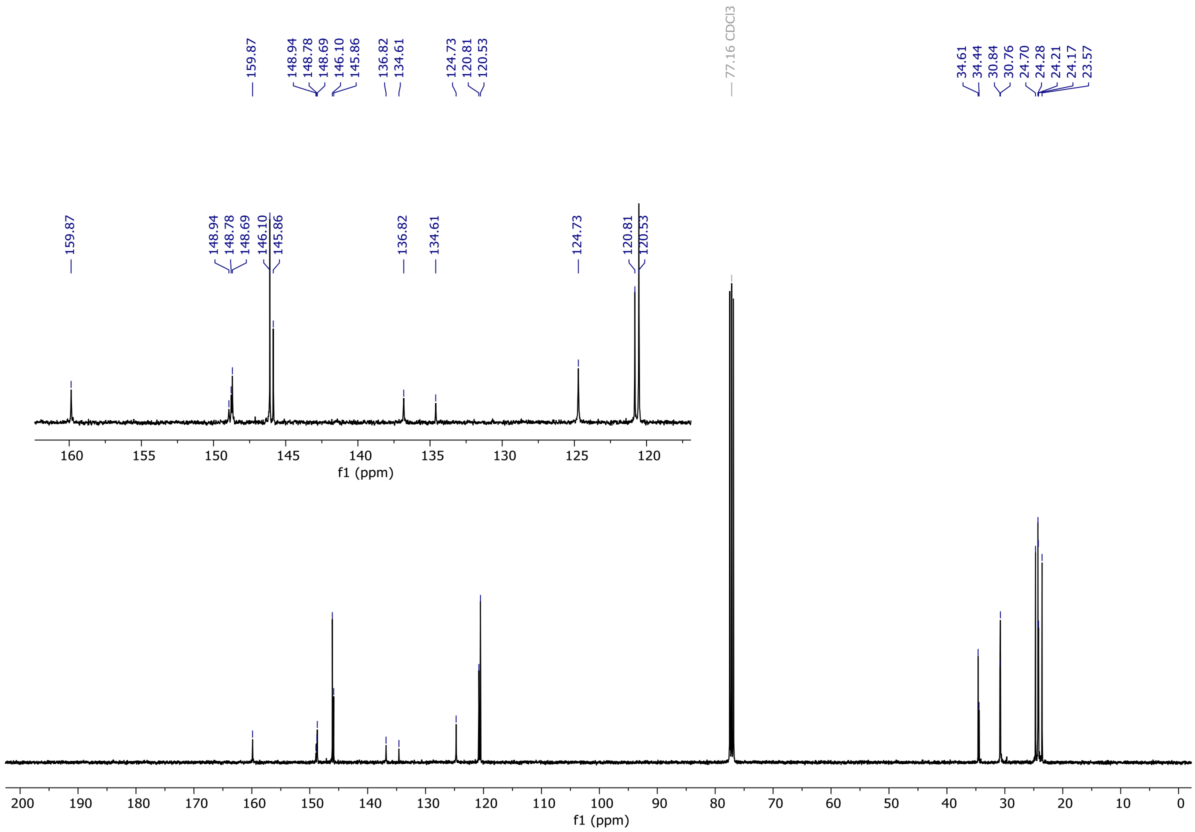

^{1}H NMR (500 MHz, $CDCl_3$) of **C5^{+}BF$_4^{-}$**

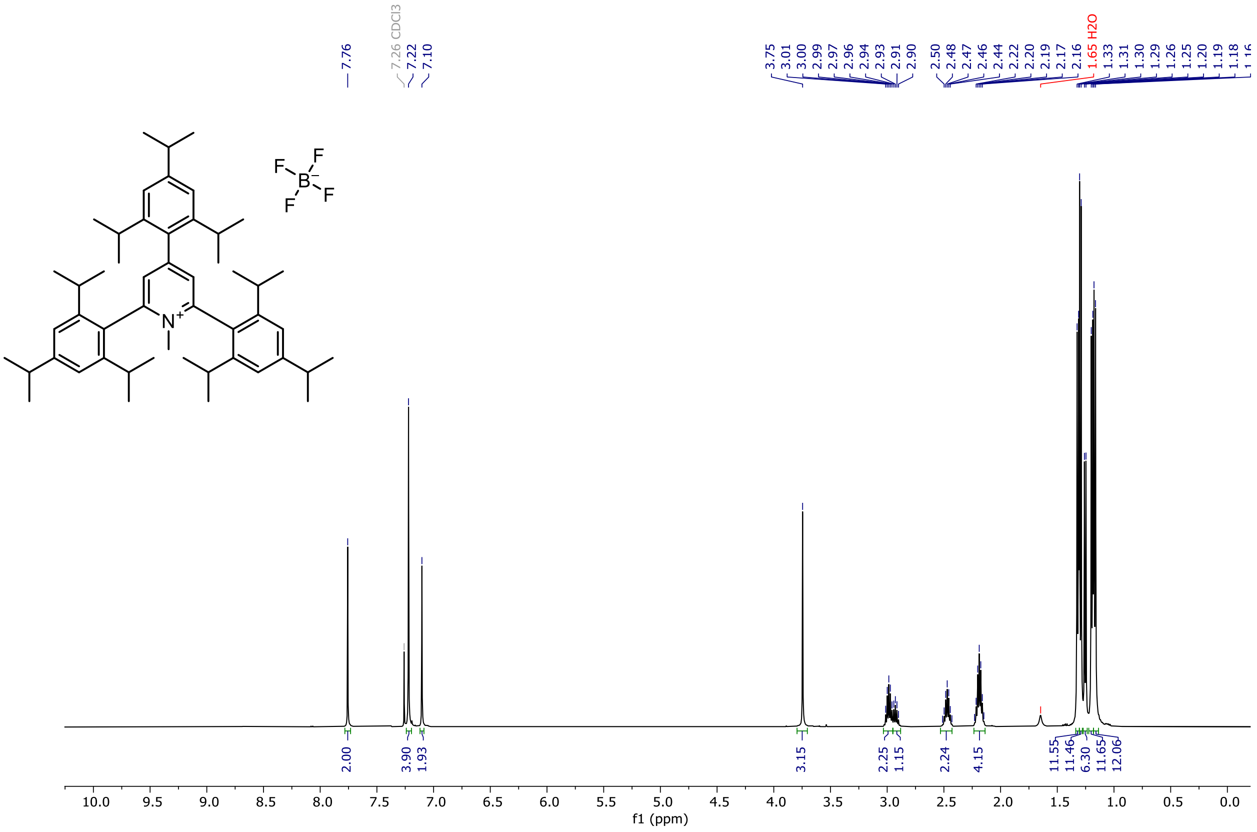

^{13}C NMR (126 MHz, $CDCl_3$) of **C5^{+}BF$_4^{-}$**

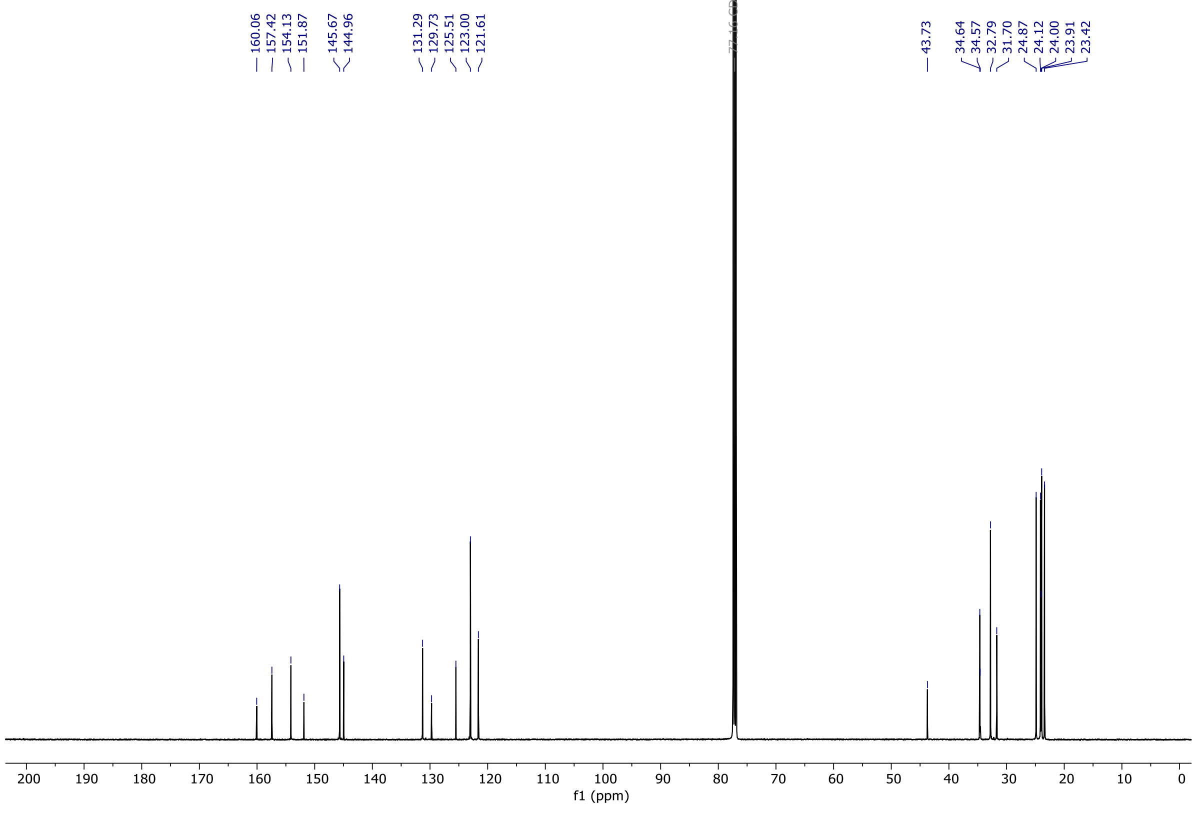

^{19}F NMR (471 MHz, CDCl$_3$) of **C5$^+$BF$_4$$^-$**

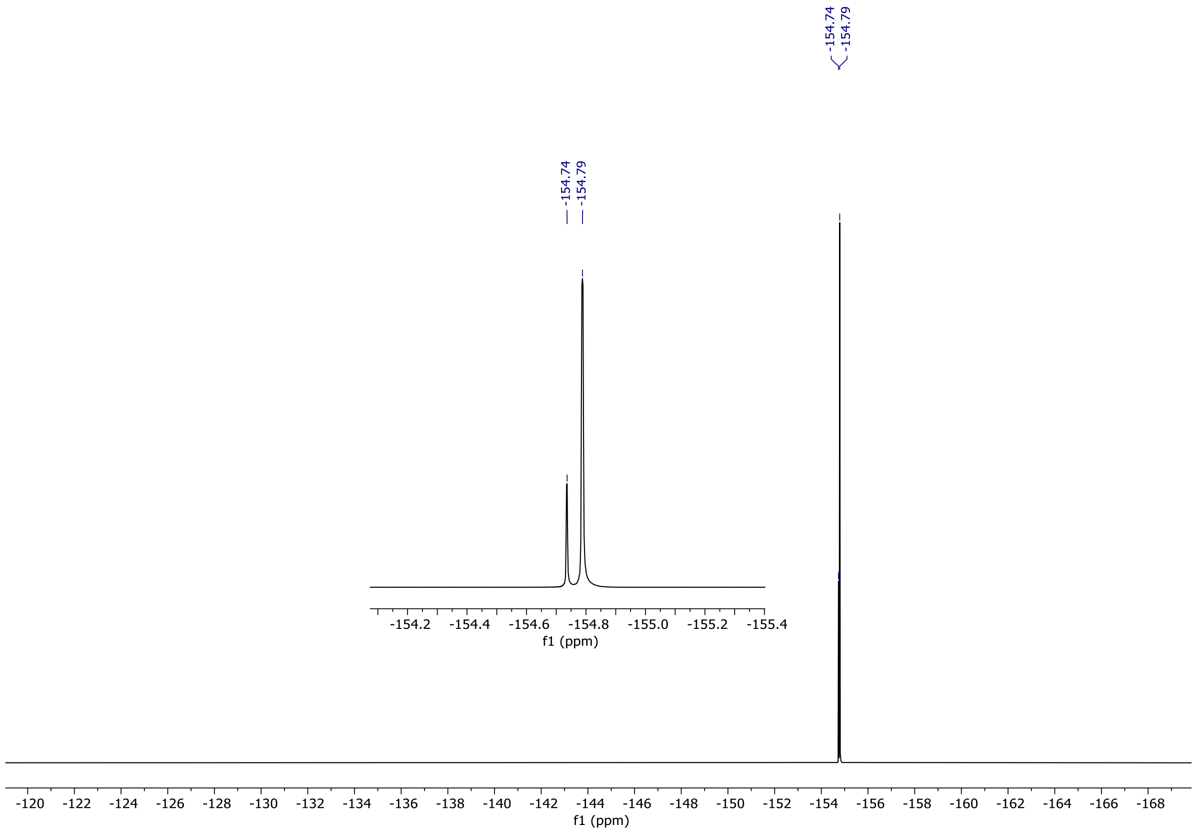

^{1}H NMR (400 MHz, $CDCl_3$) of 2-(4-bromo-3,5-diisopropylphenyl)-4,4,5,5-tetramethyl-1,3,2-dioxaborolane

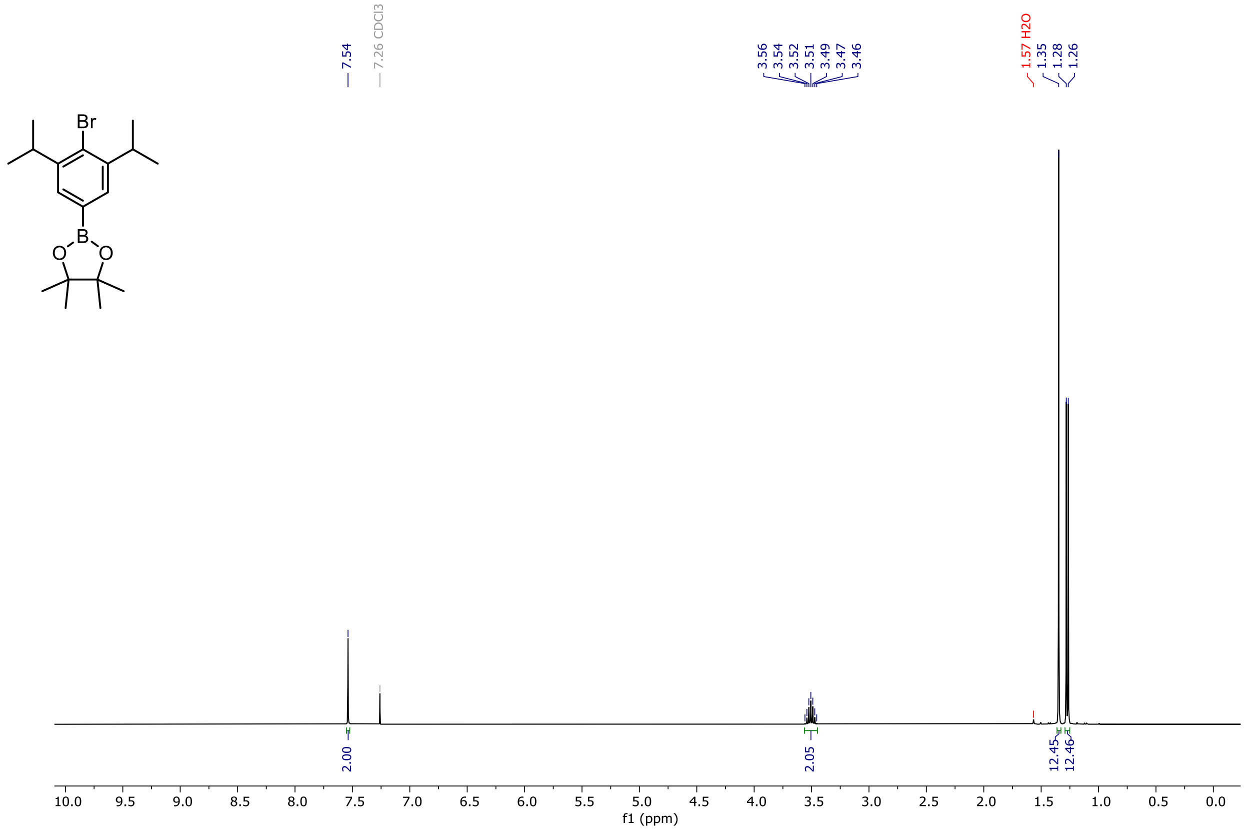

^{13}C NMR (101 MHz, $CDCl_3$) of 2-(4-bromo-3,5-diisopropylphenyl)-4,4,5,5-tetramethyl-1,3,2-dioxaborolane

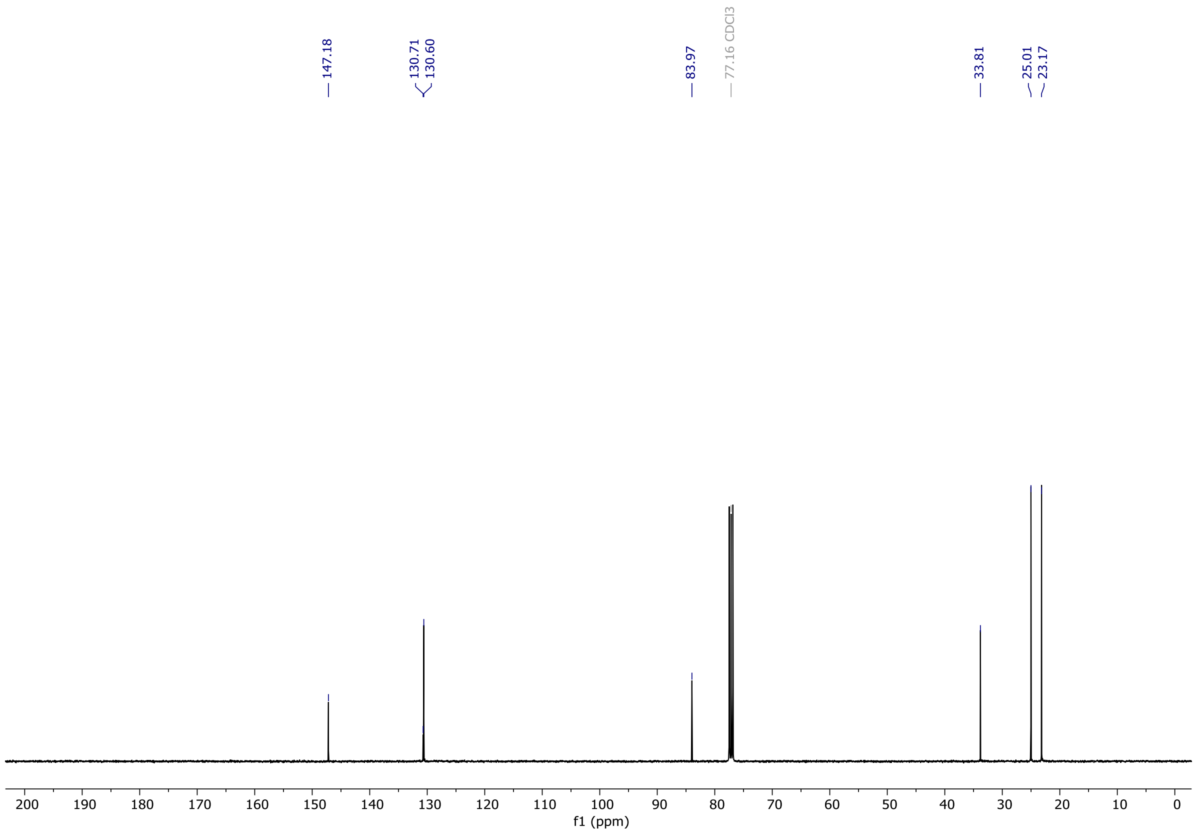

^{1}H NMR (400 MHz, $CDCl_3$) of **R^{b}-Br**

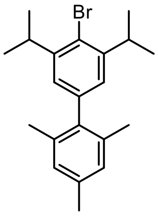

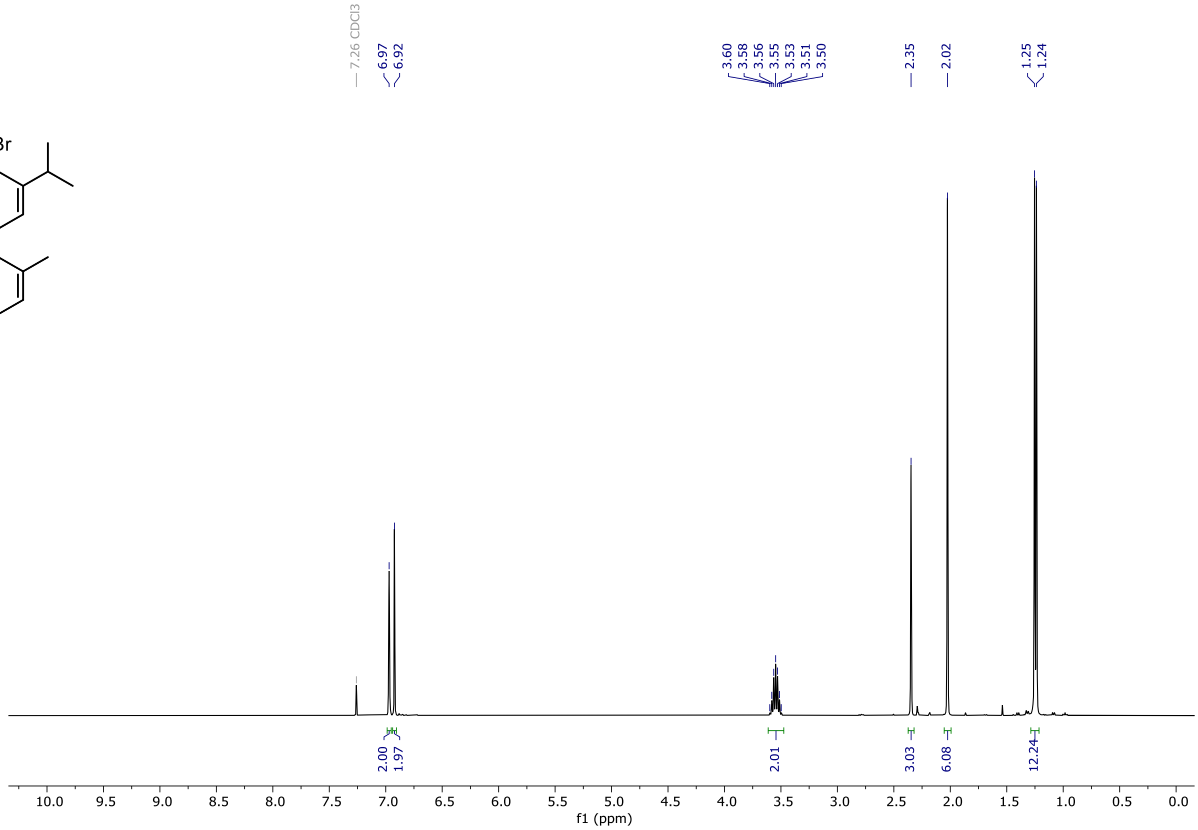

^{13}C NMR (101 MHz, $CDCl_3$) of **R^{b}-Br**

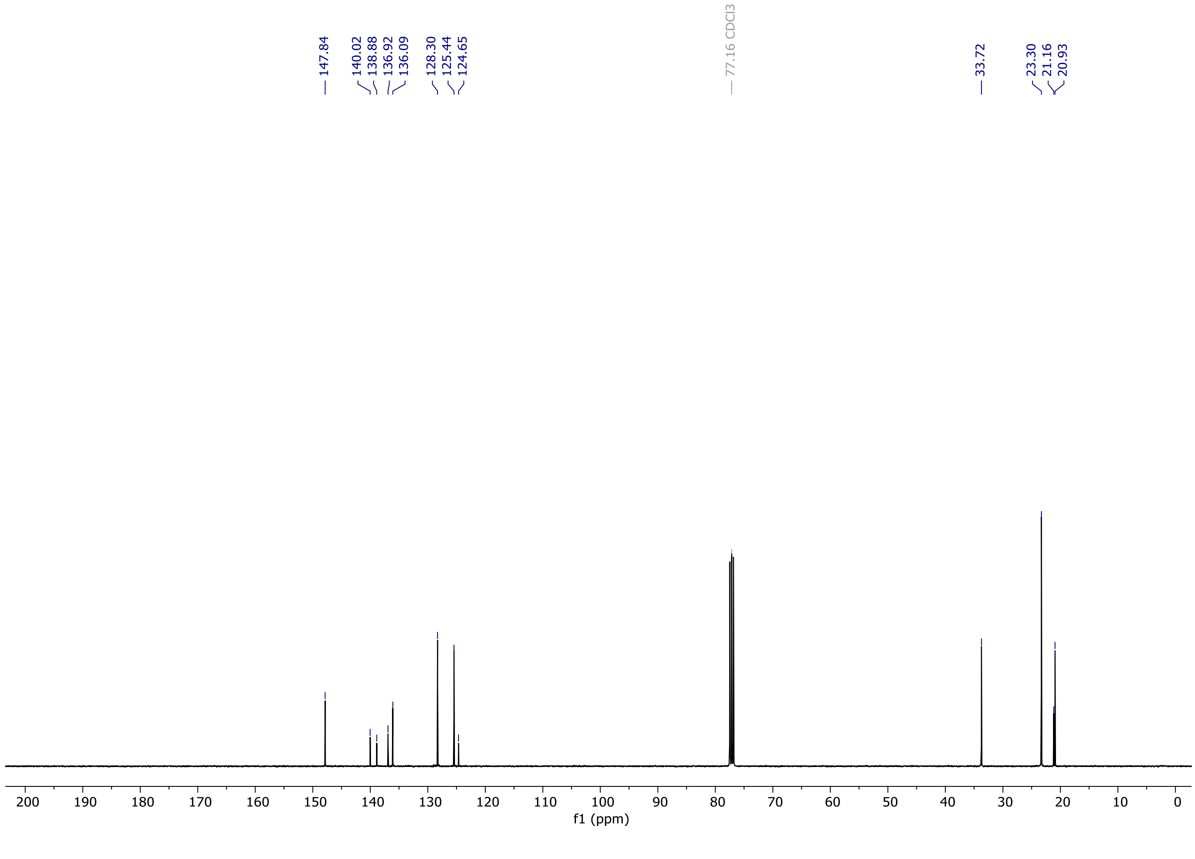

^{1}H NMR (400 MHz, $CDCl_3$) of **C6_N**

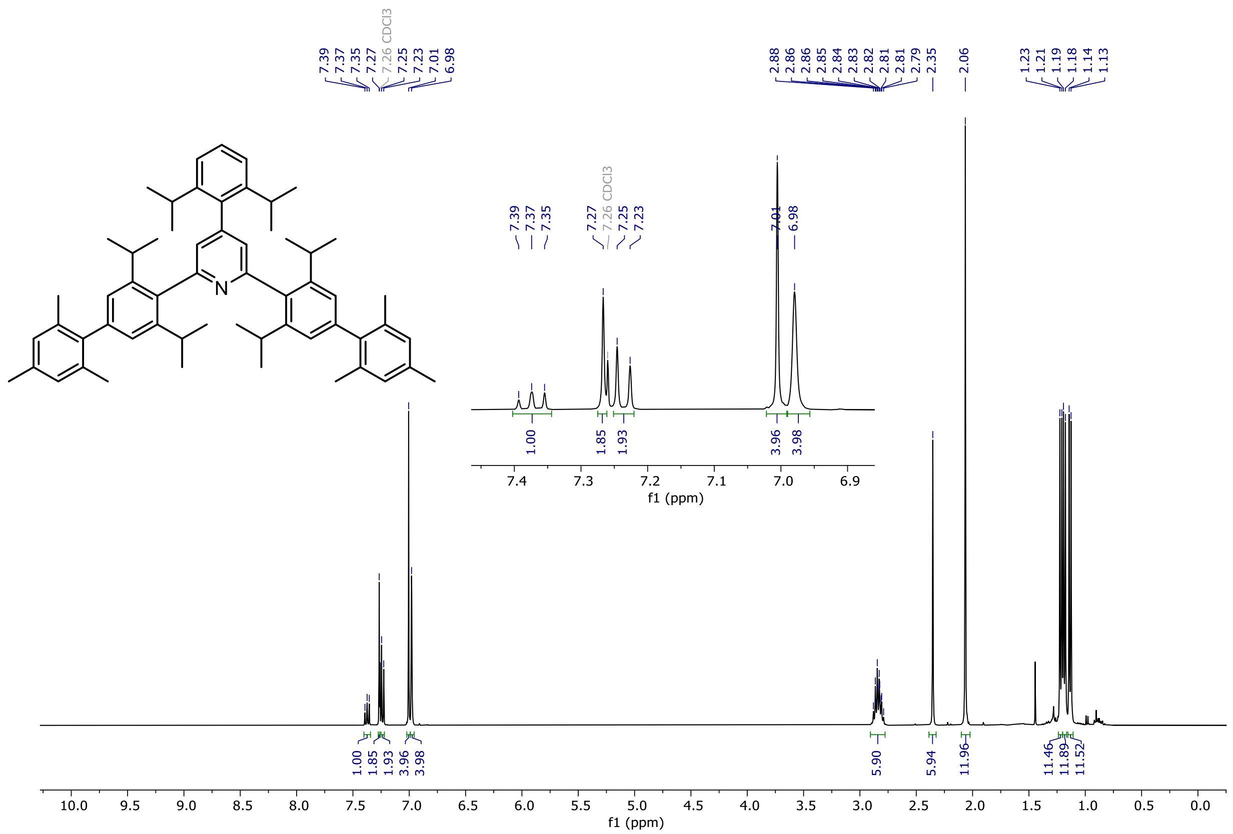

^{13}C NMR (101 MHz, $CDCl_3$) of **C6_N**

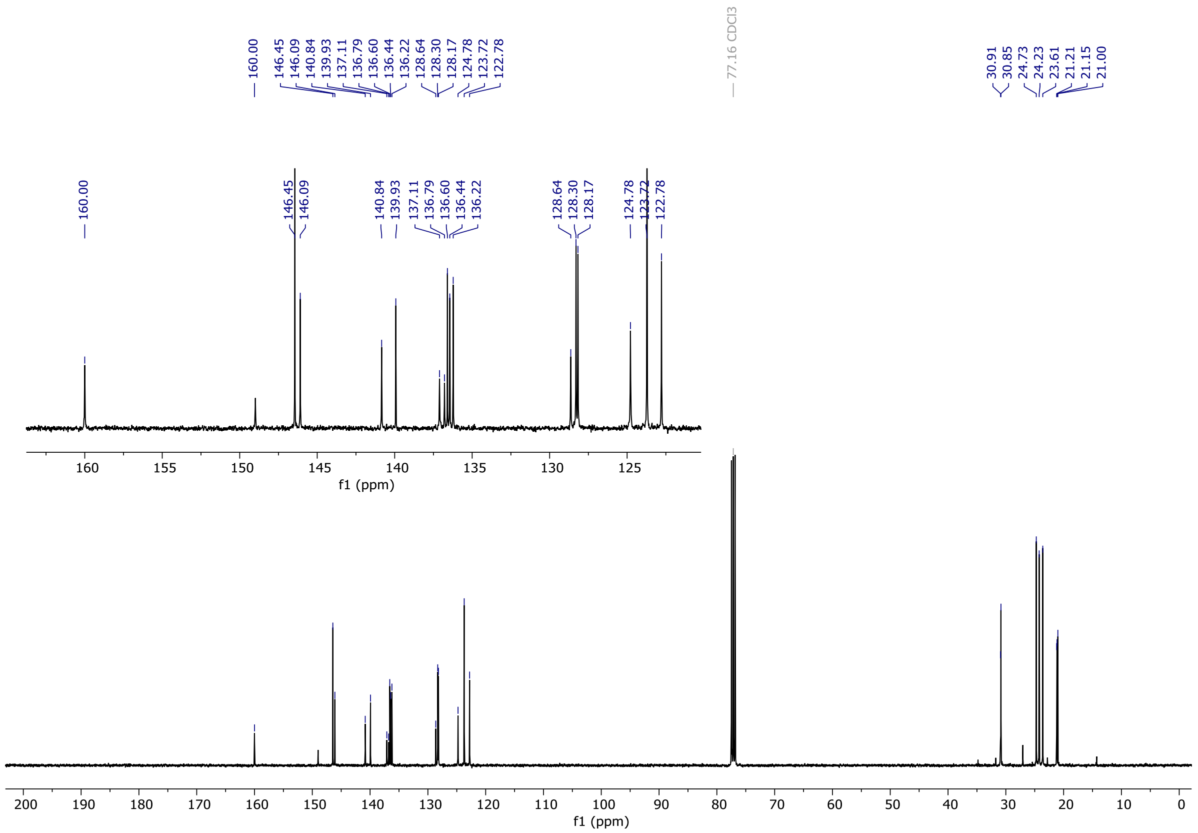

^{1}H NMR (400 MHz, $CDCl_3$) of **C6^{+}BF$_4^{-}$**

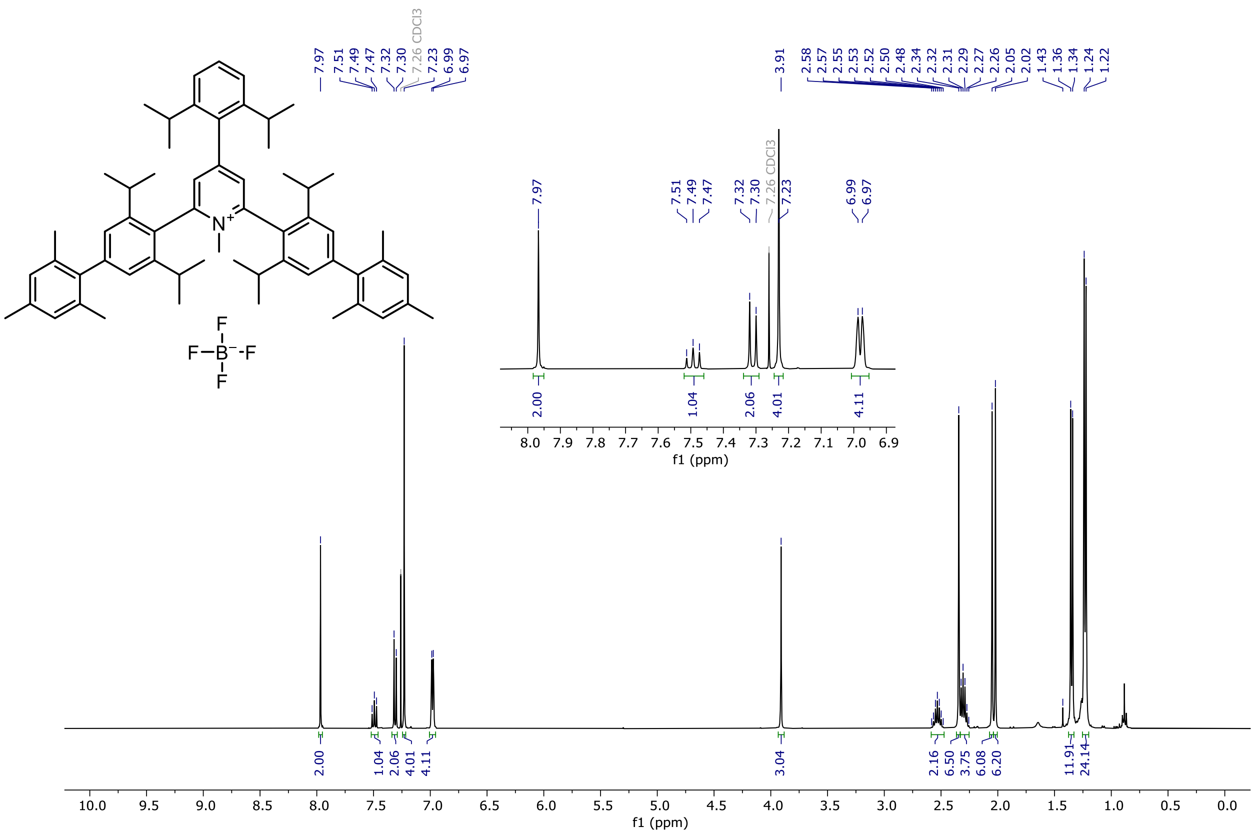

^{13}C NMR (101 MHz, $CDCl_3$) of **C6^{+}BF$_4^{-}$**

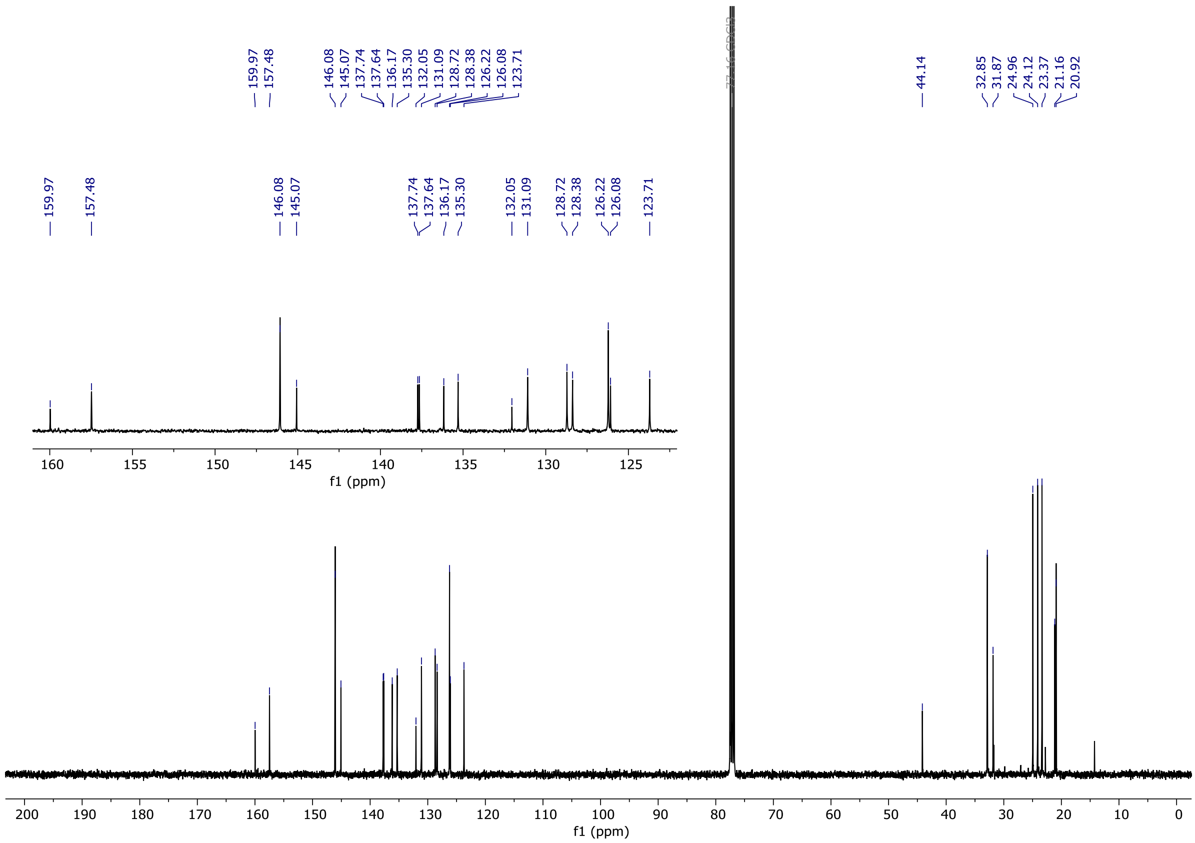

^{19}F NMR (376 MHz, $CDCl_3$) of **C6^{+}BF$_4^{-}$**

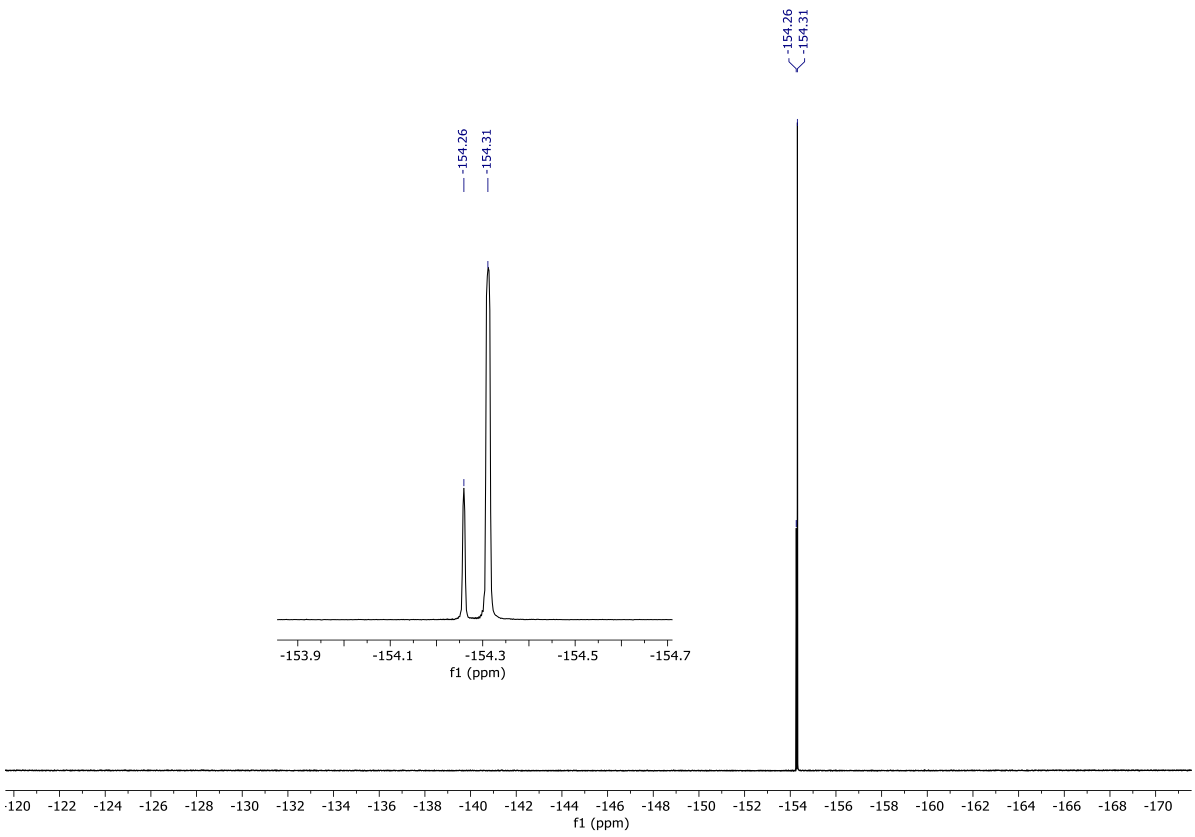

^{1}H NMR (400 MHz, $CDCl_3$) of **R^c-Br**

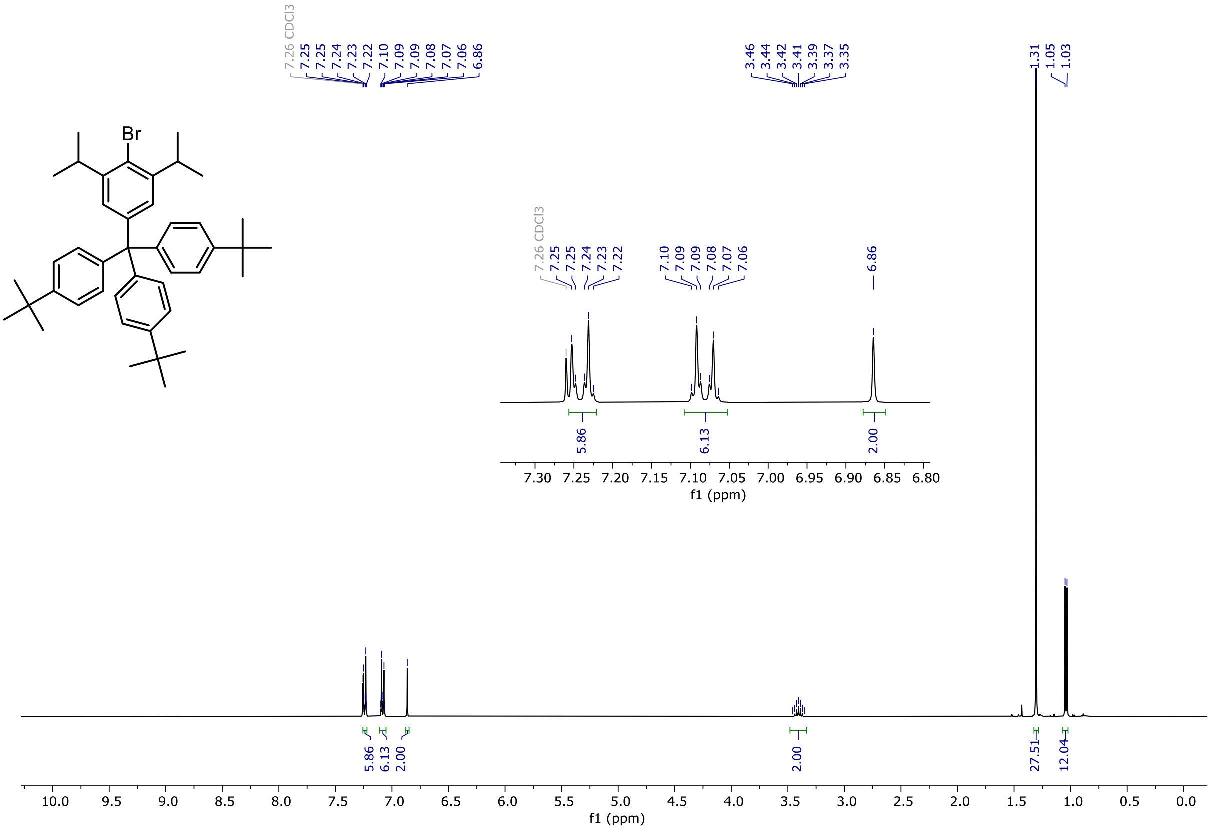

^{13}C NMR (101 MHz, $CDCl_3$) of **R^c-Br**

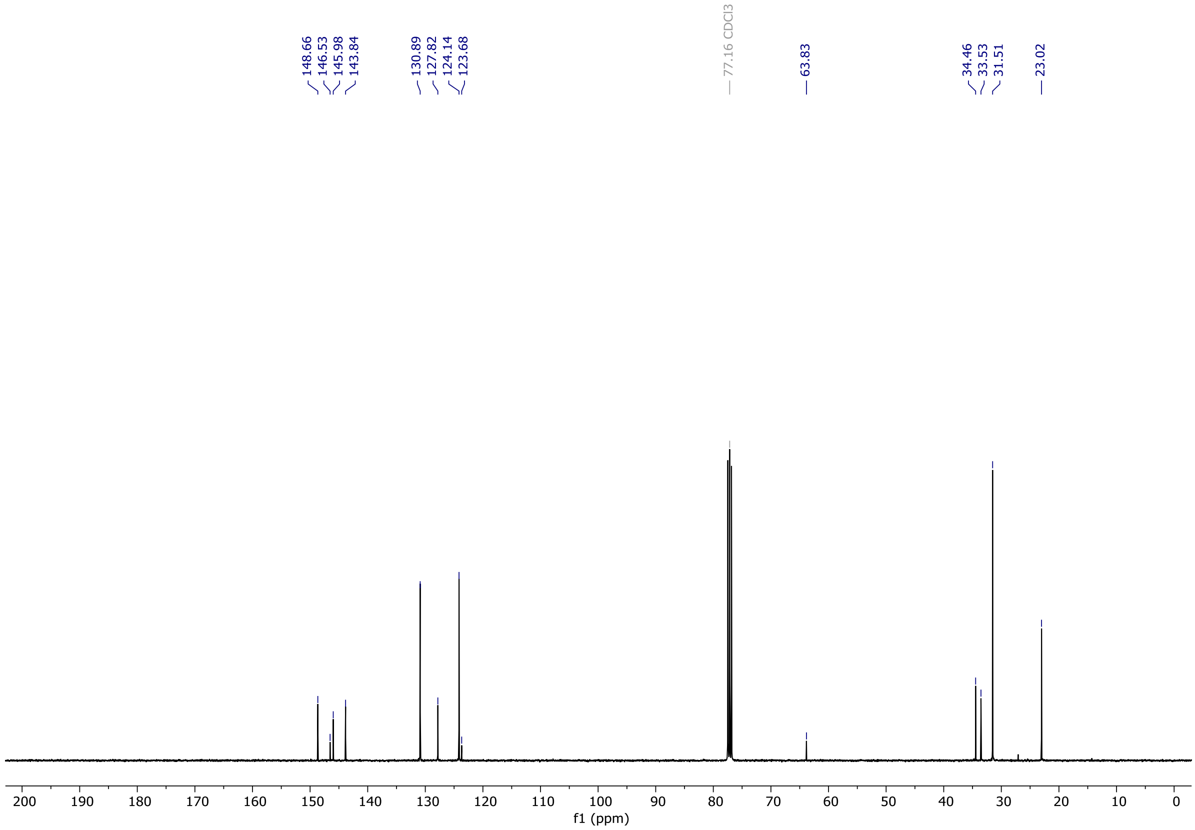

^{1}H NMR (400 MHz, $CDCl_3$) of **C7_N**

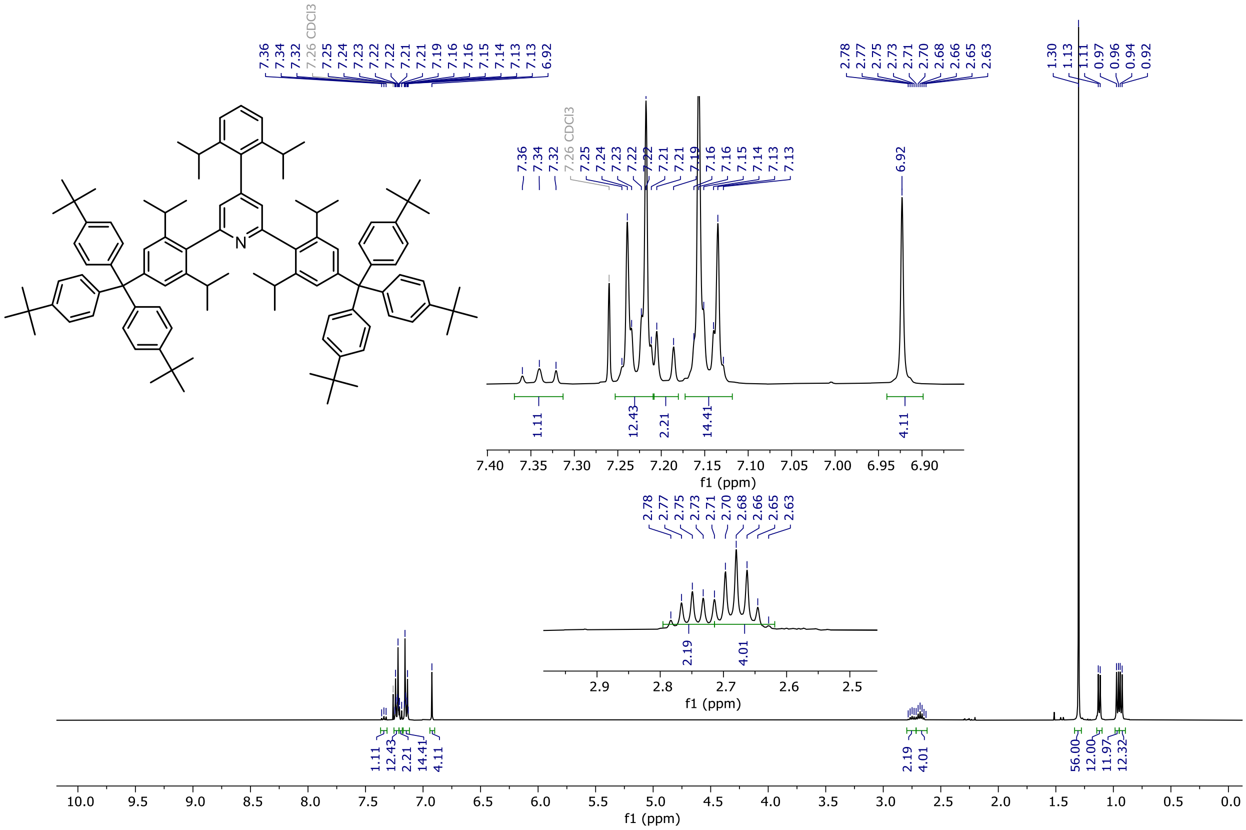

^{13}C NMR (101 MHz, $CDCl_3$) of **C7_N**

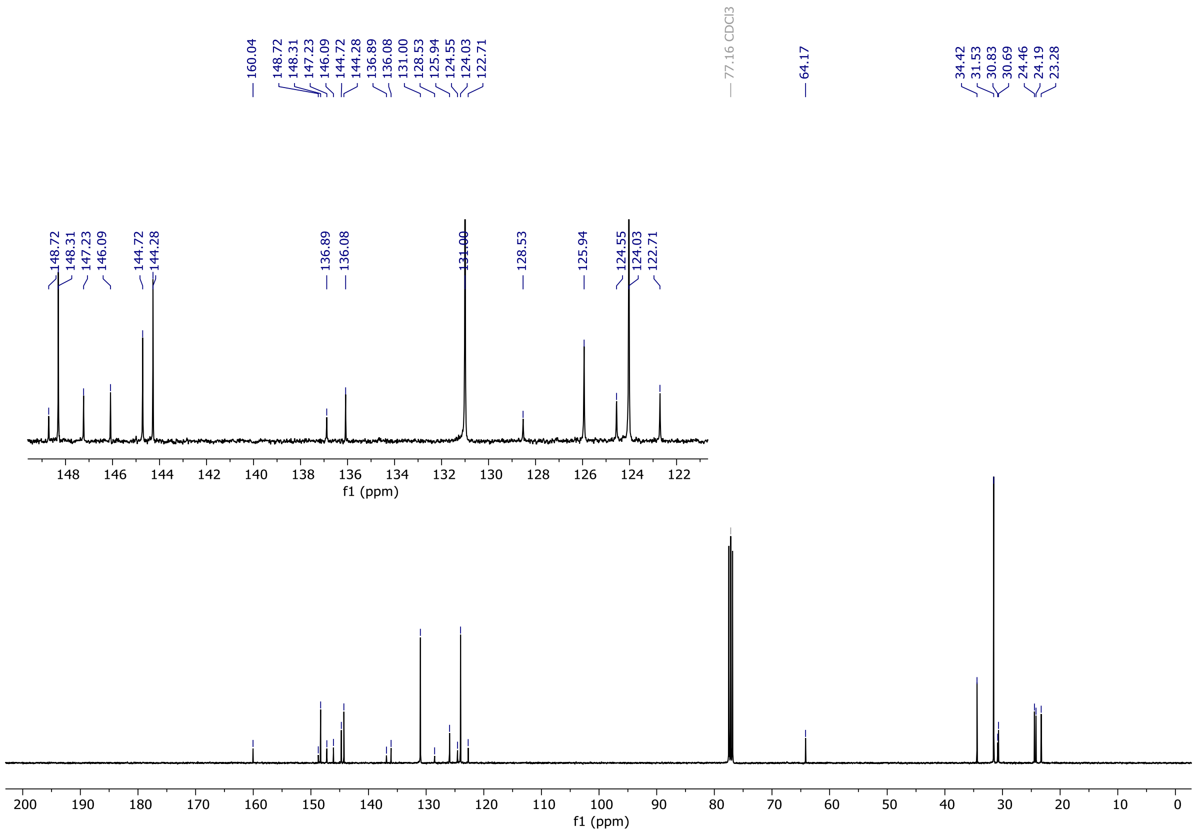

^{1}H NMR (400 MHz, $CDCl_3$) of **C7$^+$BF$_4^-$**

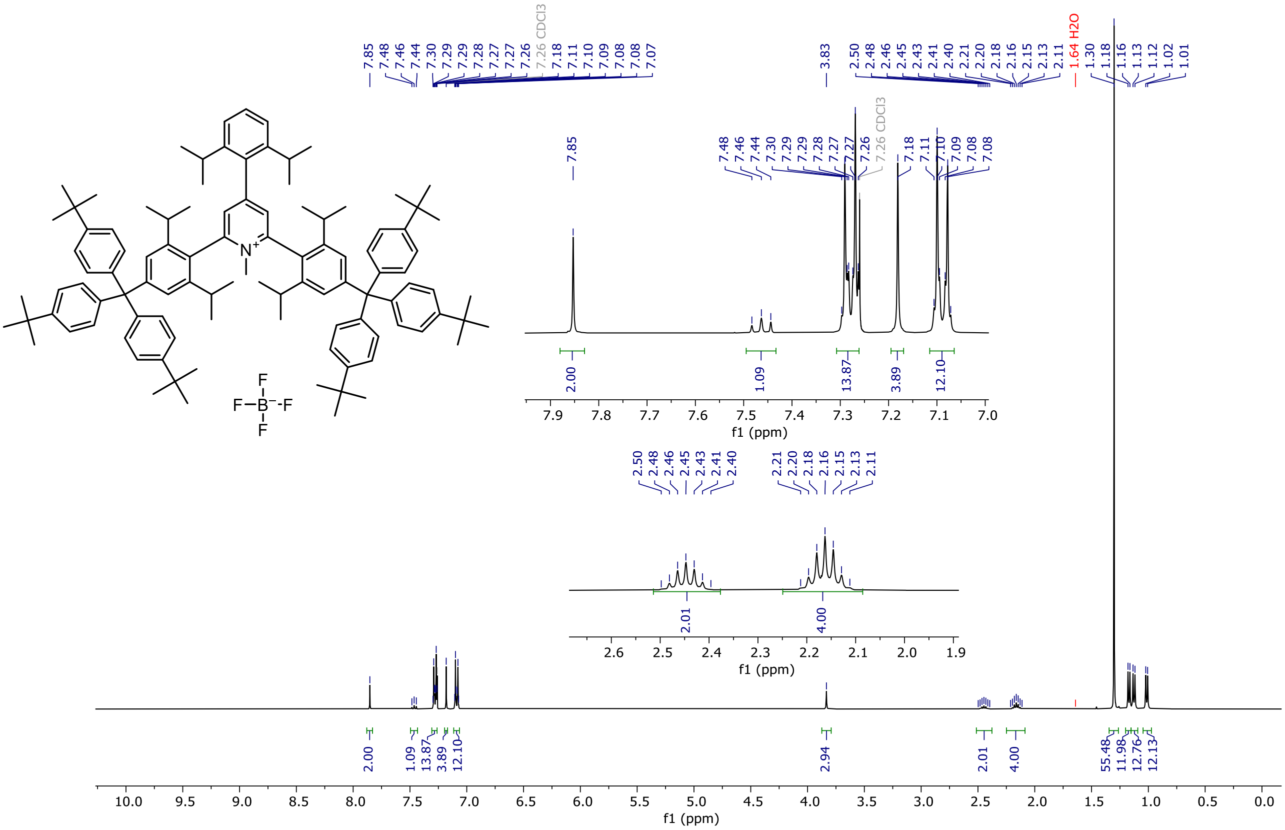

^{13}C NMR (101 MHz, $CDCl_3$) of **C7$^+$BF$_4^-$**

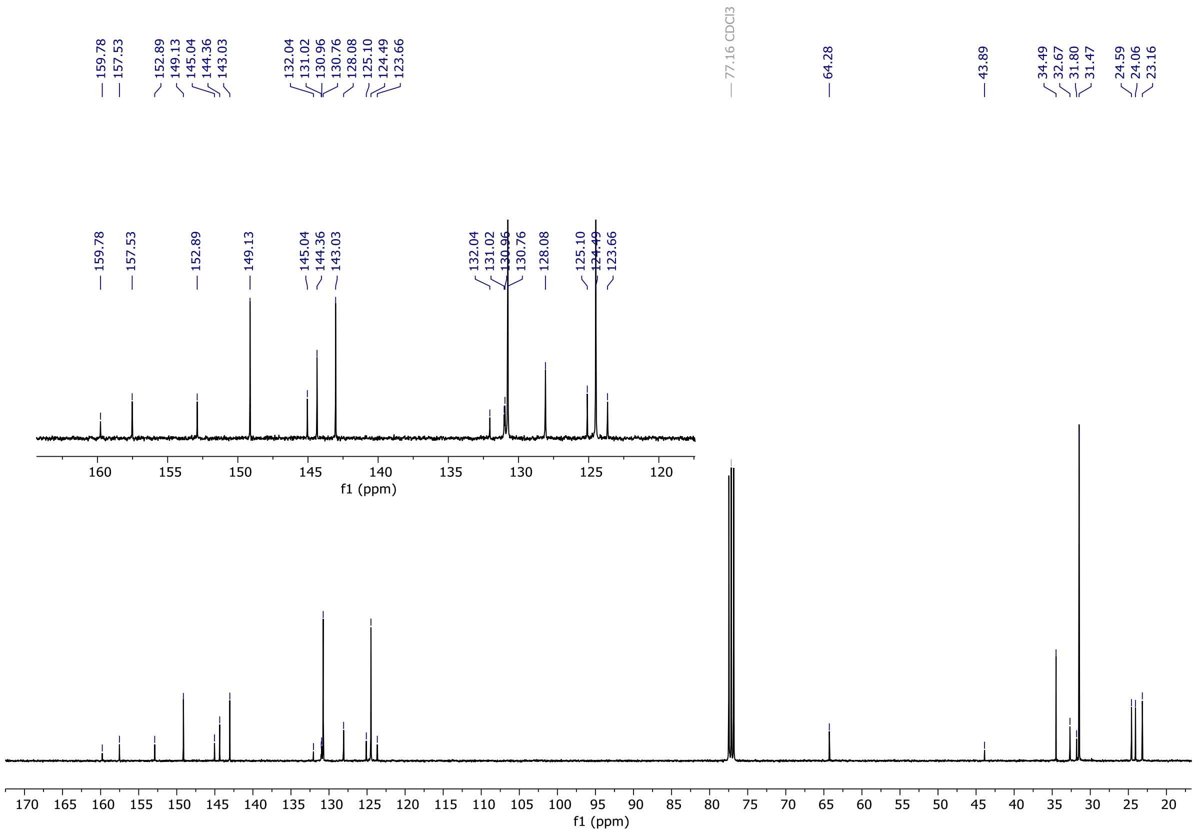

^{19}F NMR (376 MHz, $CDCl_3$) of **C7^{+}BF$_4^{-}$**

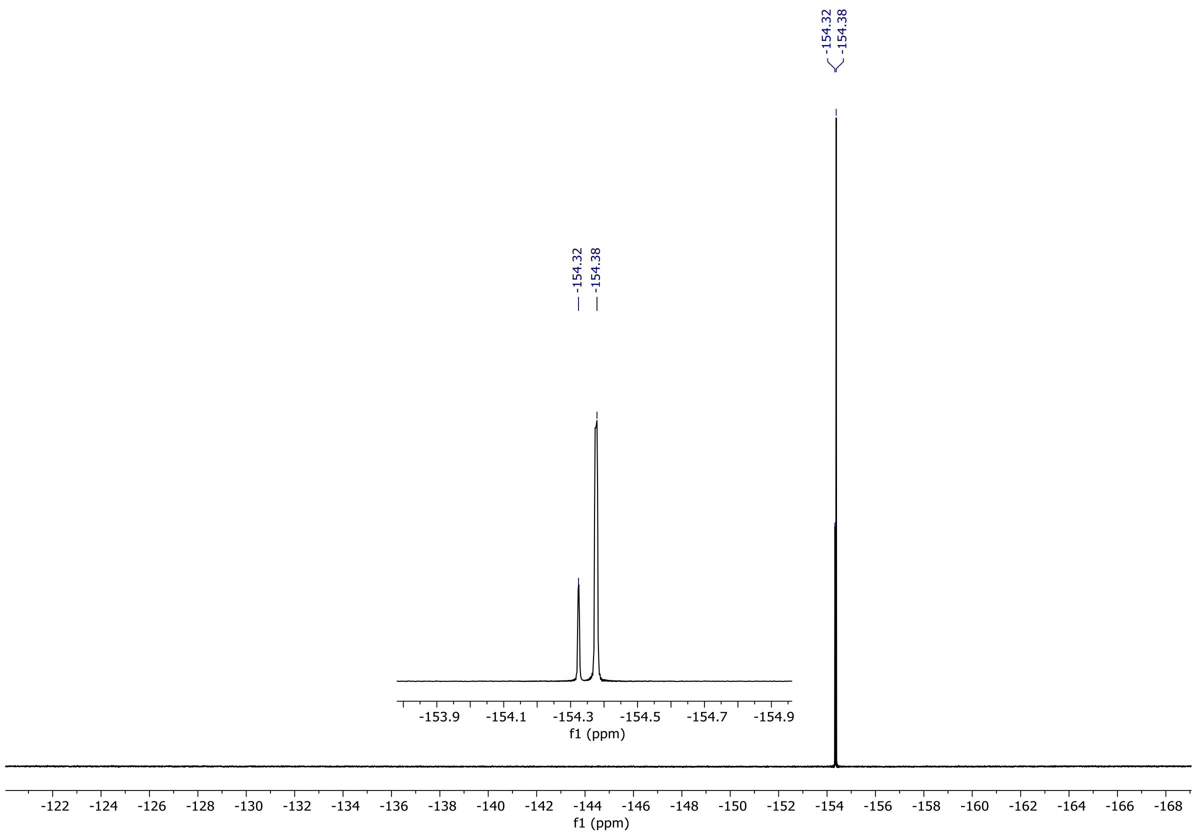

^1H NMR (500 MHz, $CDCl_3$) of 3',5'-dichloro-2,6-diisopropyl-1,1'-biphenyl

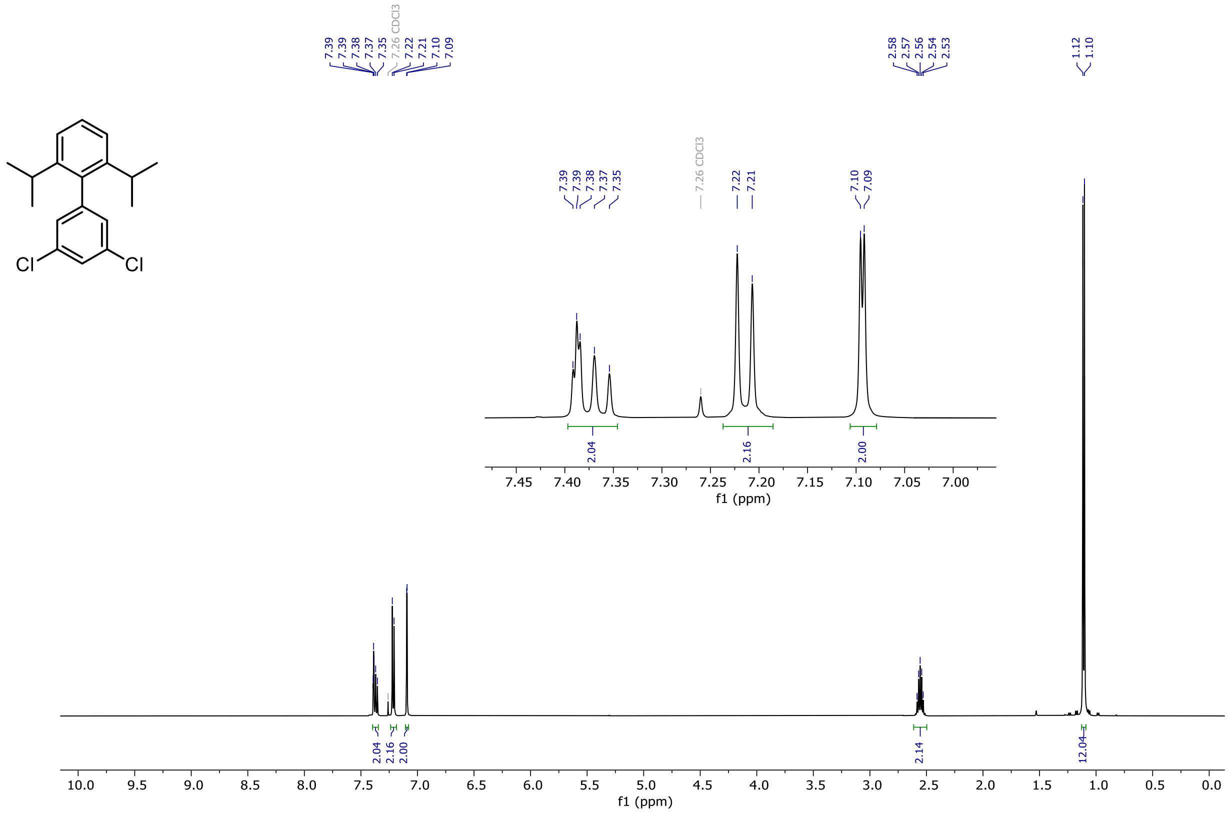

^{13}C NMR (126 MHz, $CDCl_3$) of 3',5'-dichloro-2,6-diisopropyl-1,1'-biphenyl

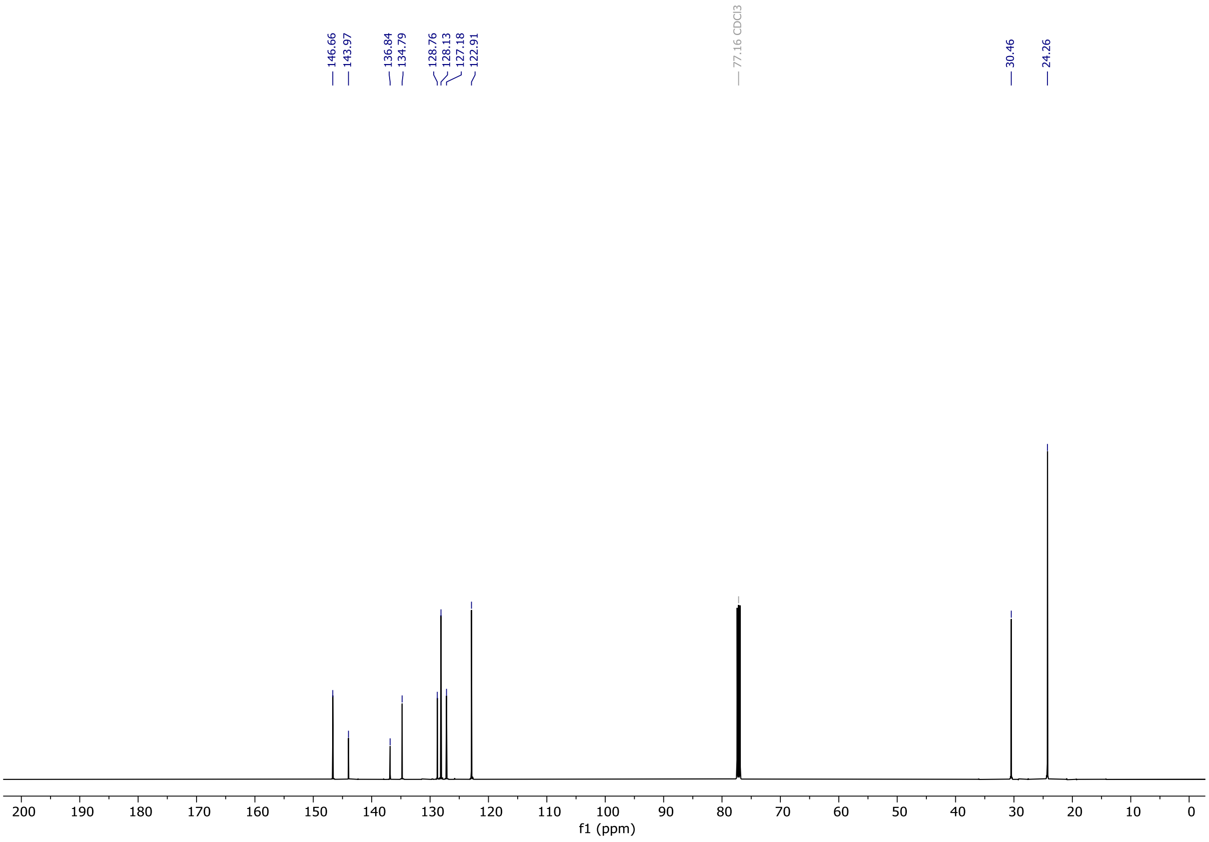

^{1}H NMR (400 MHz, $CDCl_3$) of **A4_Br**

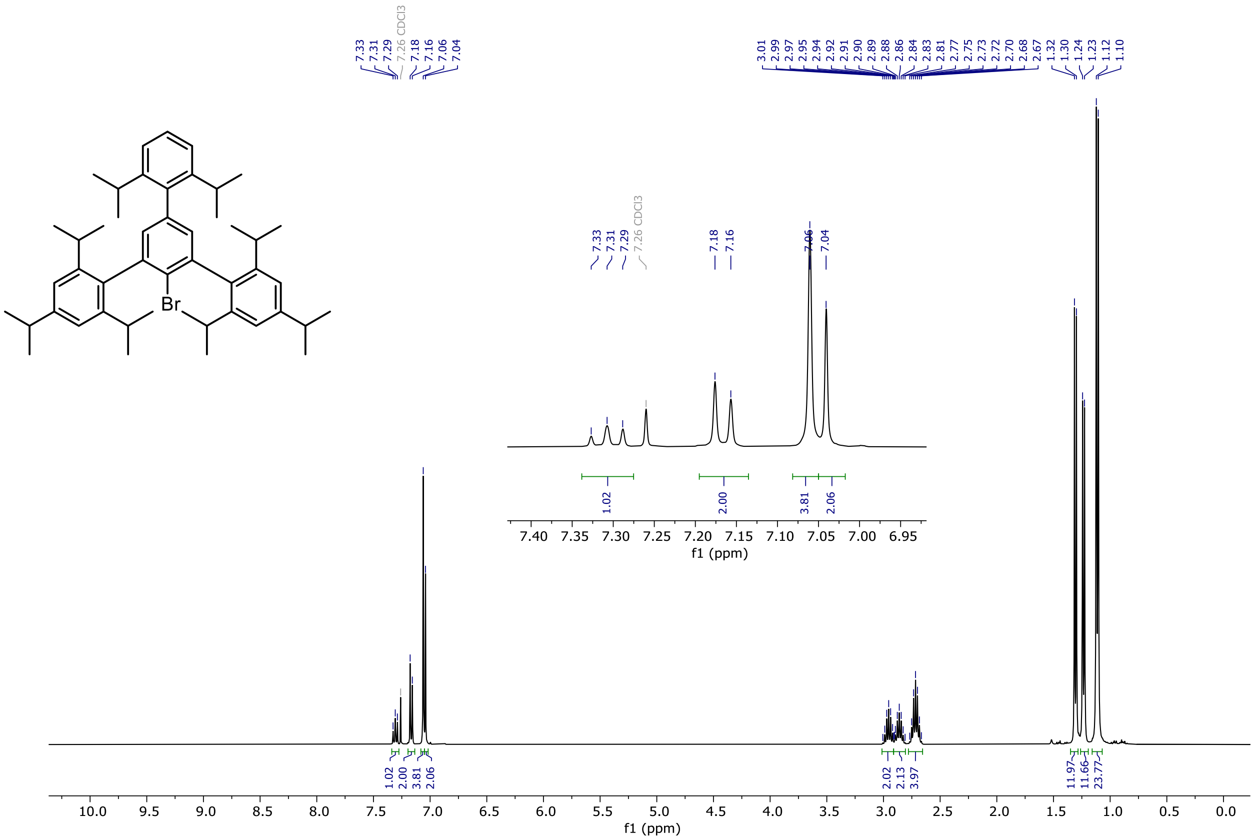

^{13}C NMR (101 MHz, $CDCl_3$) of **A4_Br**

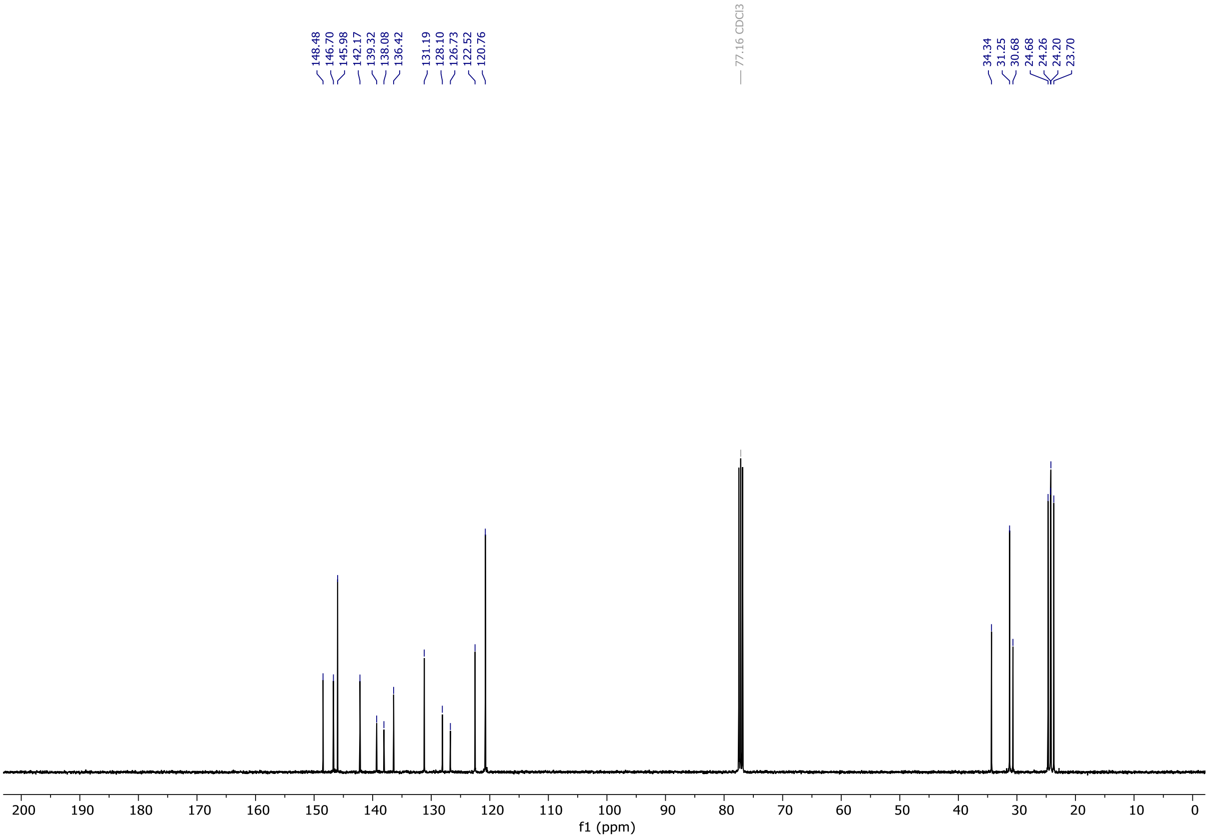

^{1}H NMR (400 MHz, $CDCl_3$) of **A4_SO₂Cl**

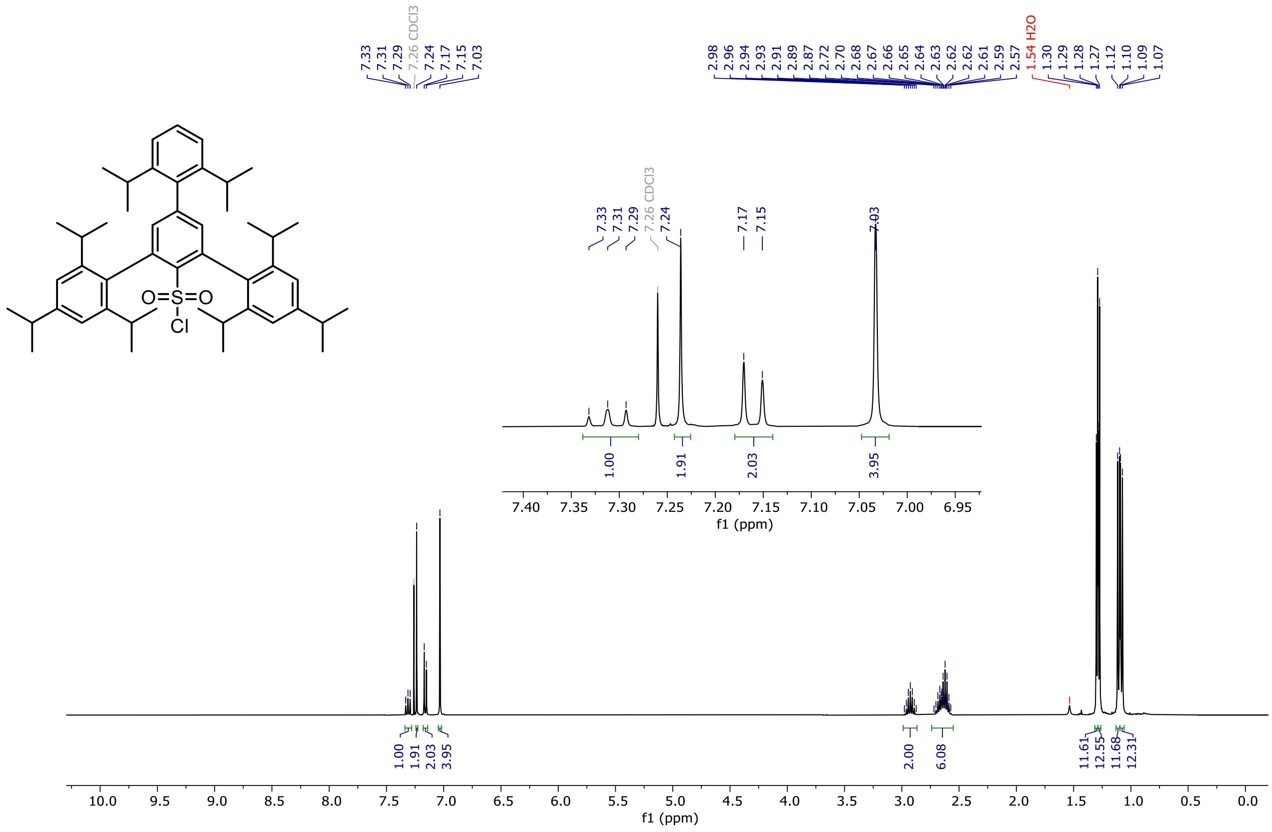

^{13}C NMR (101 MHz, $CDCl_3$) of **A4_SO₂Cl**

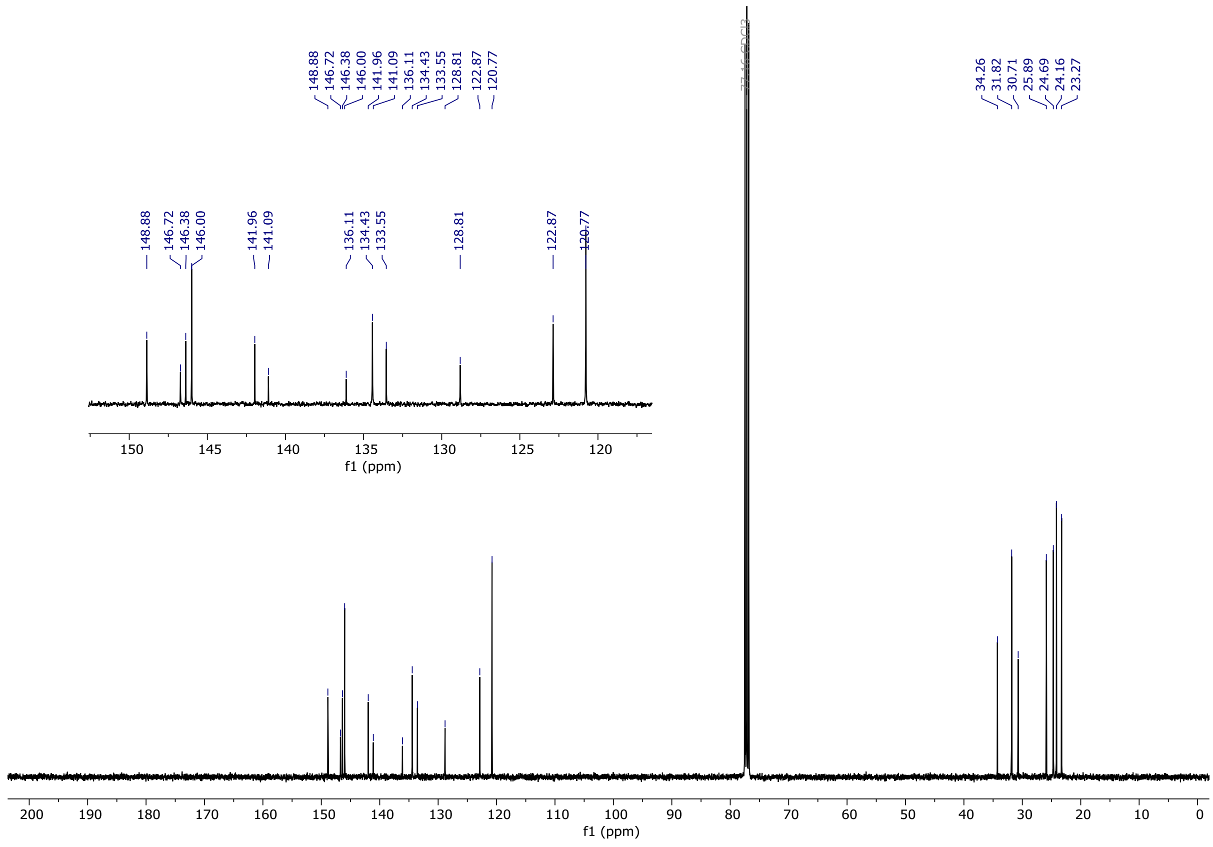

^{1}H NMR (500 MHz, $CDCl_3$) of **Na^{+}A4^{-}**

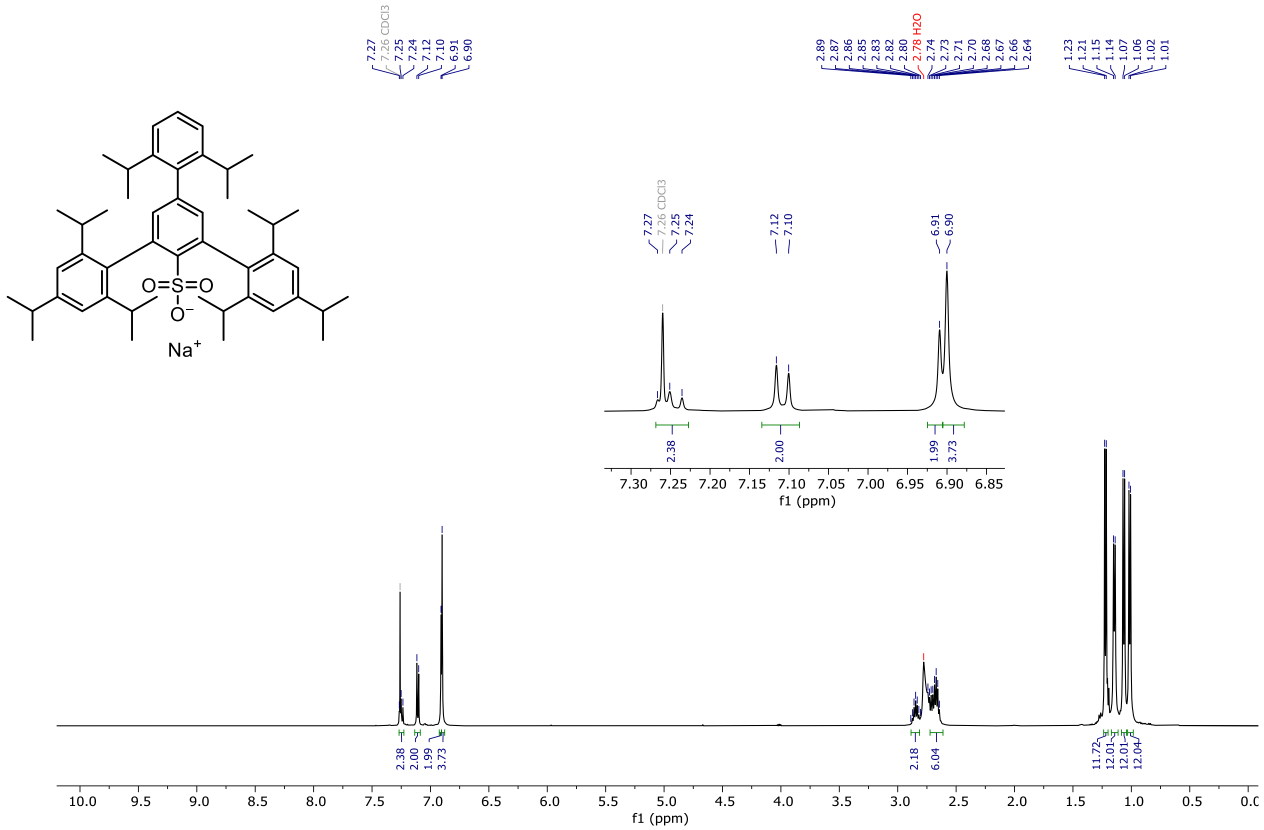

^{13}C NMR (126 MHz, $CDCl_3$) of **Na^{+}A4^{-}**

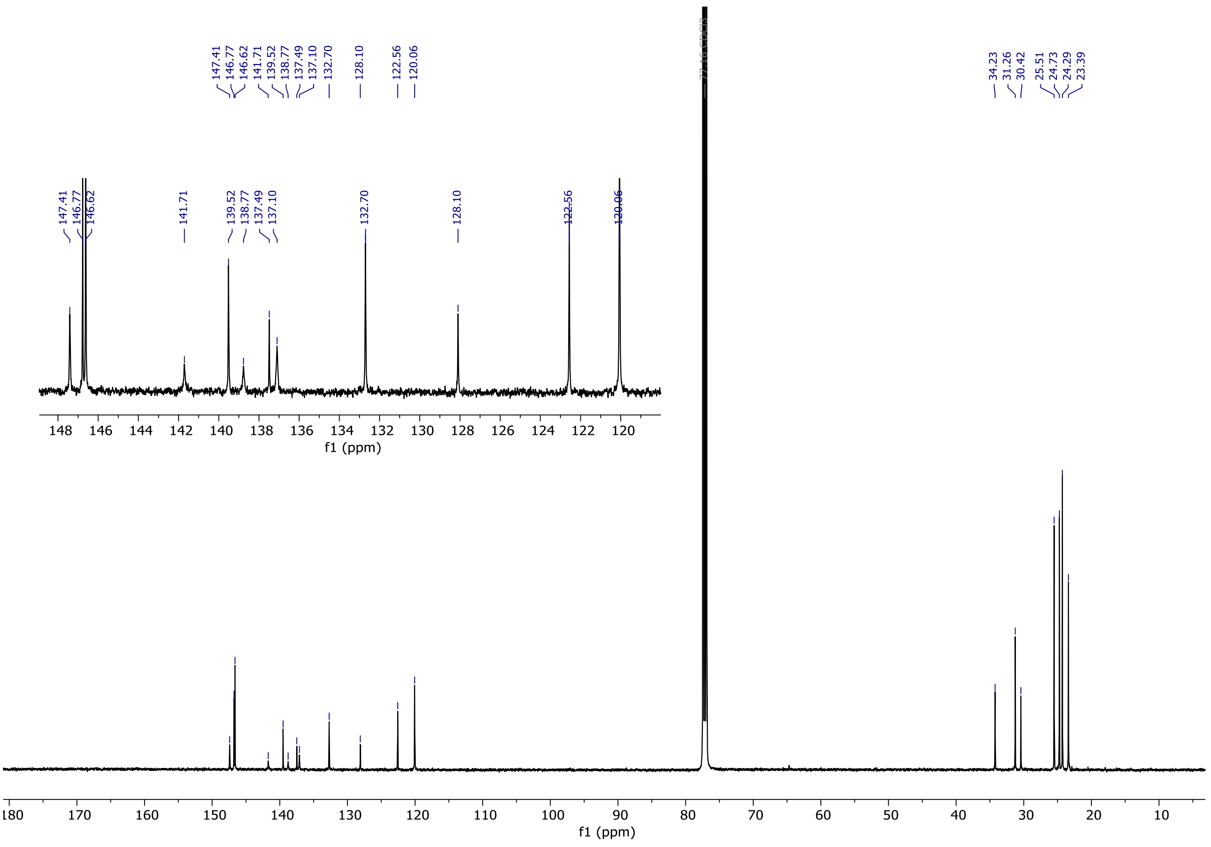

^{1}H NMR (400 MHz, $CDCl_3$) of 3',5'-dichloro-2,4,6-triisopropyl-1,1'-biphenyl

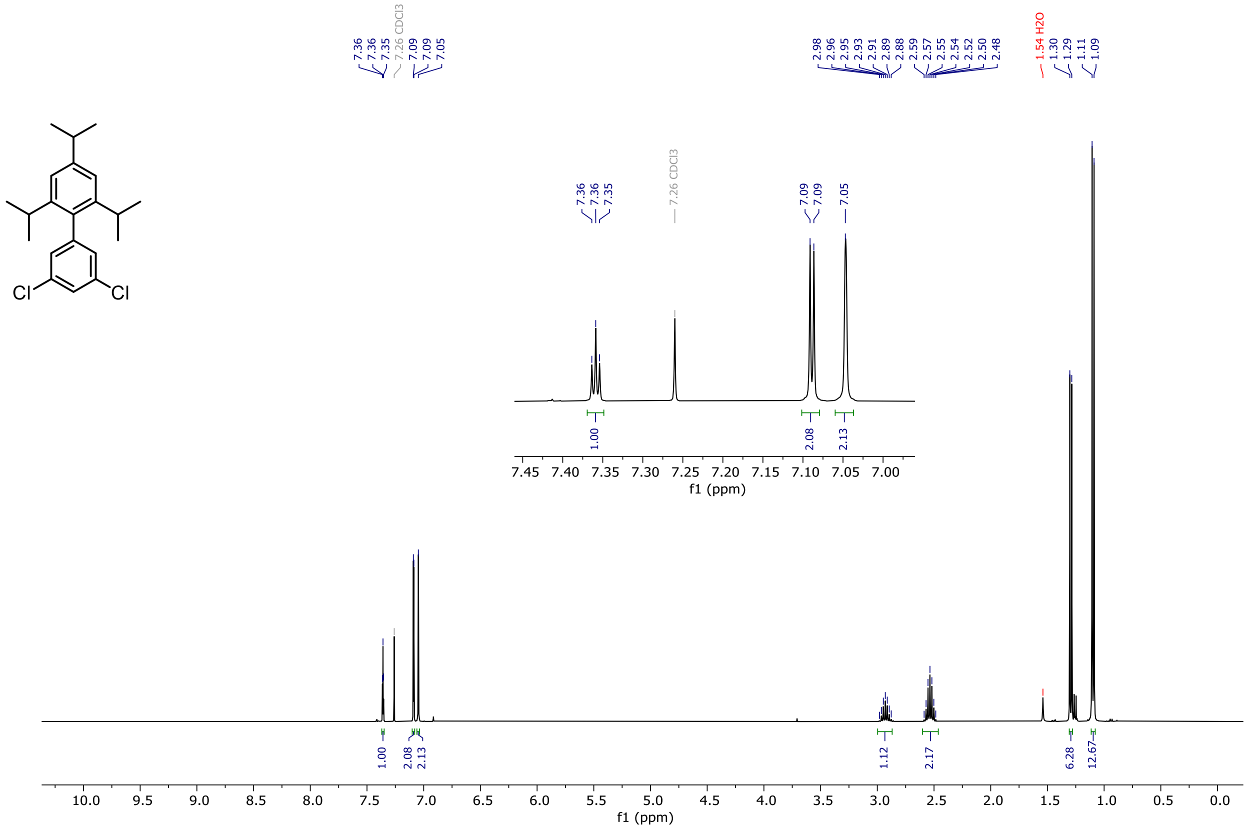

^{13}C NMR (101 MHz, $CDCl_3$) of 3',5'-dichloro-2,4,6-triisopropyl-1,1'-biphenyl

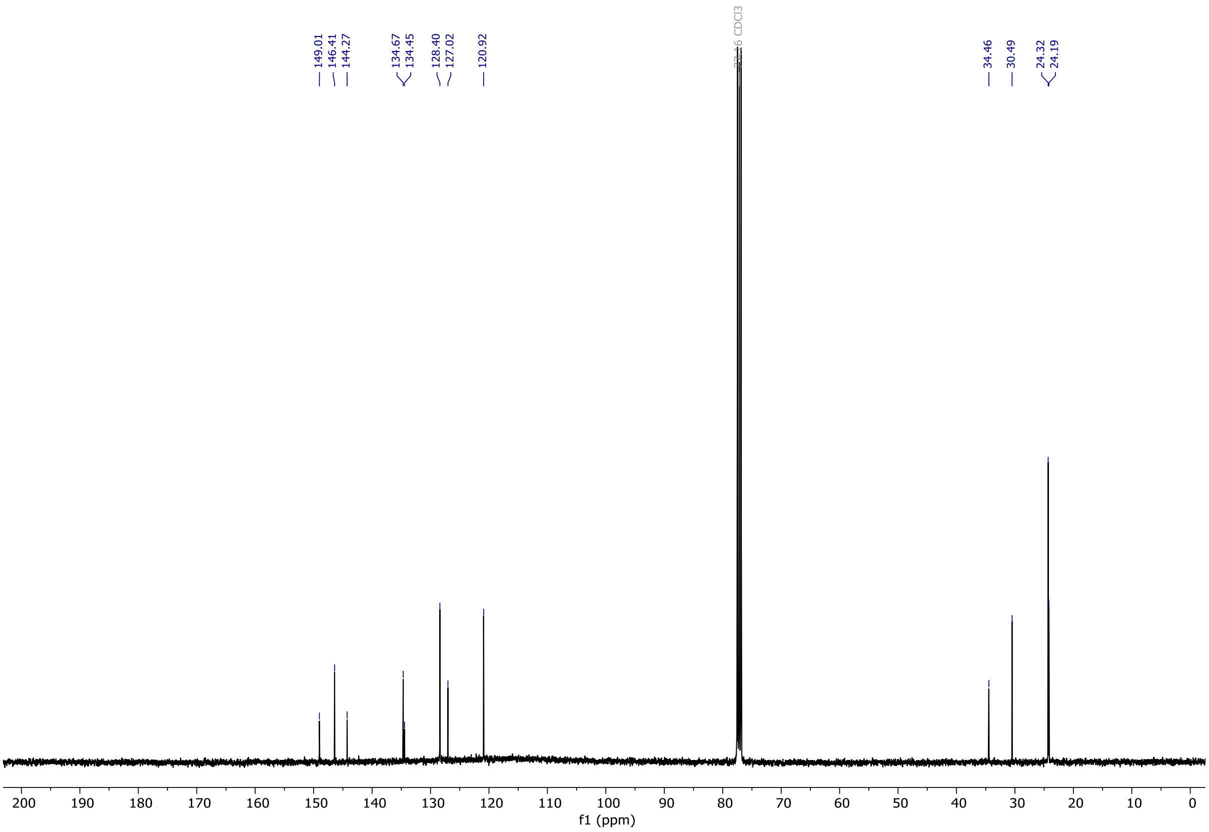

^{1}H NMR (500 MHz, $CDCl_3$) of **A5_Br**

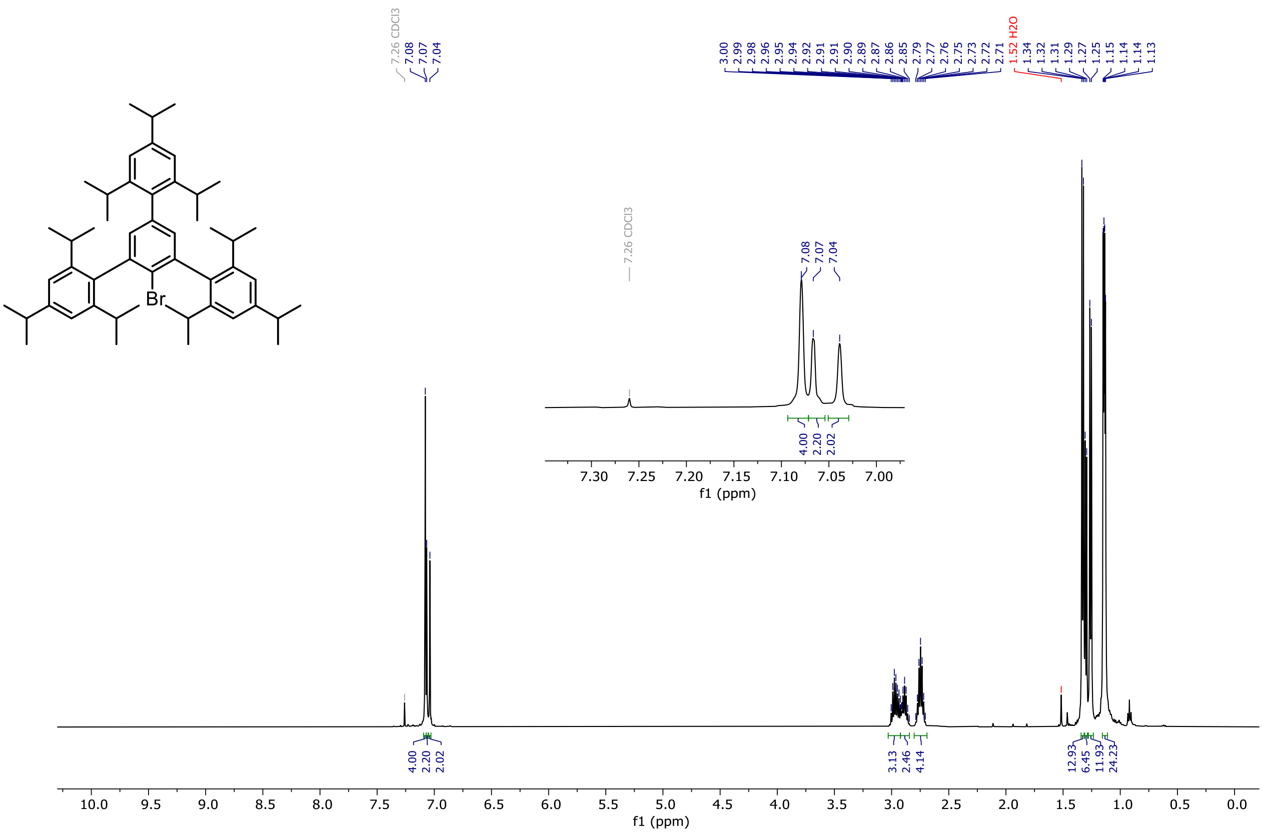

^{13}C NMR (126 MHz, $CDCl_3$) of **A5_Br**

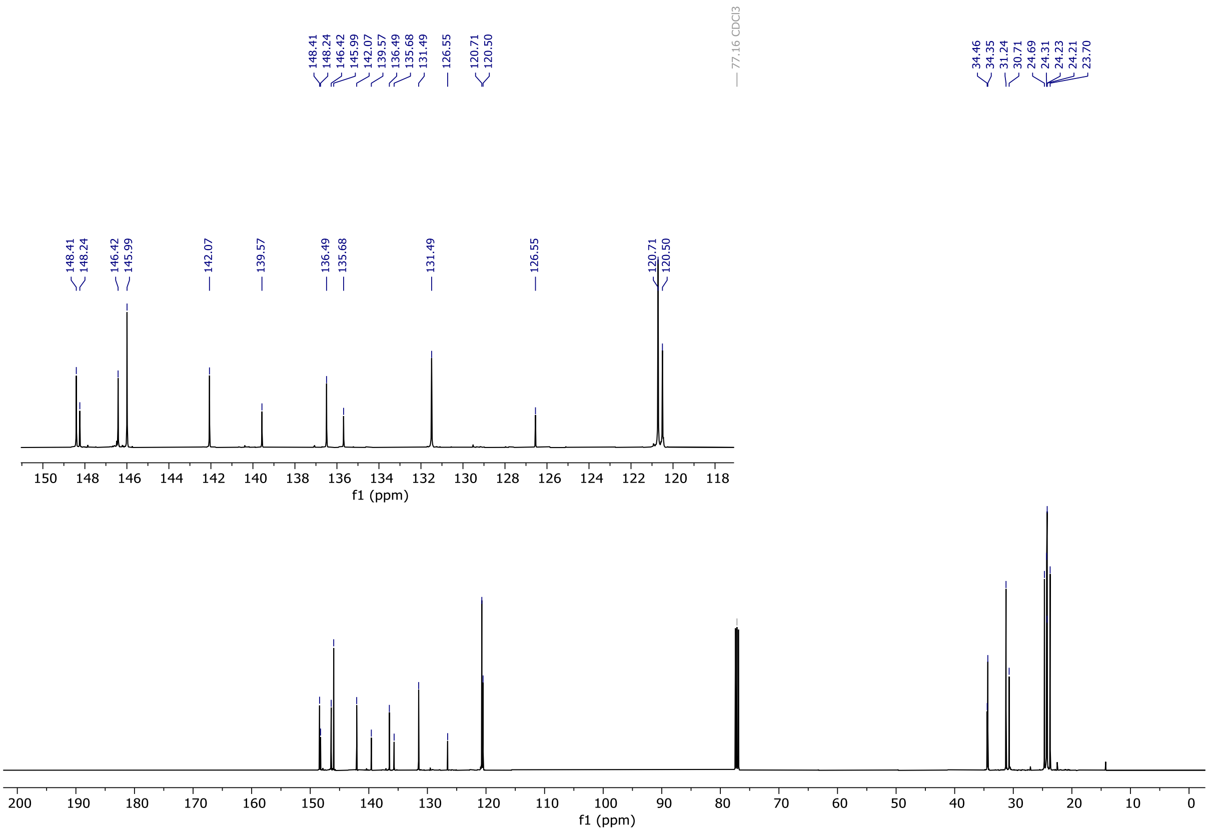

^{1}H NMR (500 MHz, $CDCl_3$) of **A5_SO₂Cl**

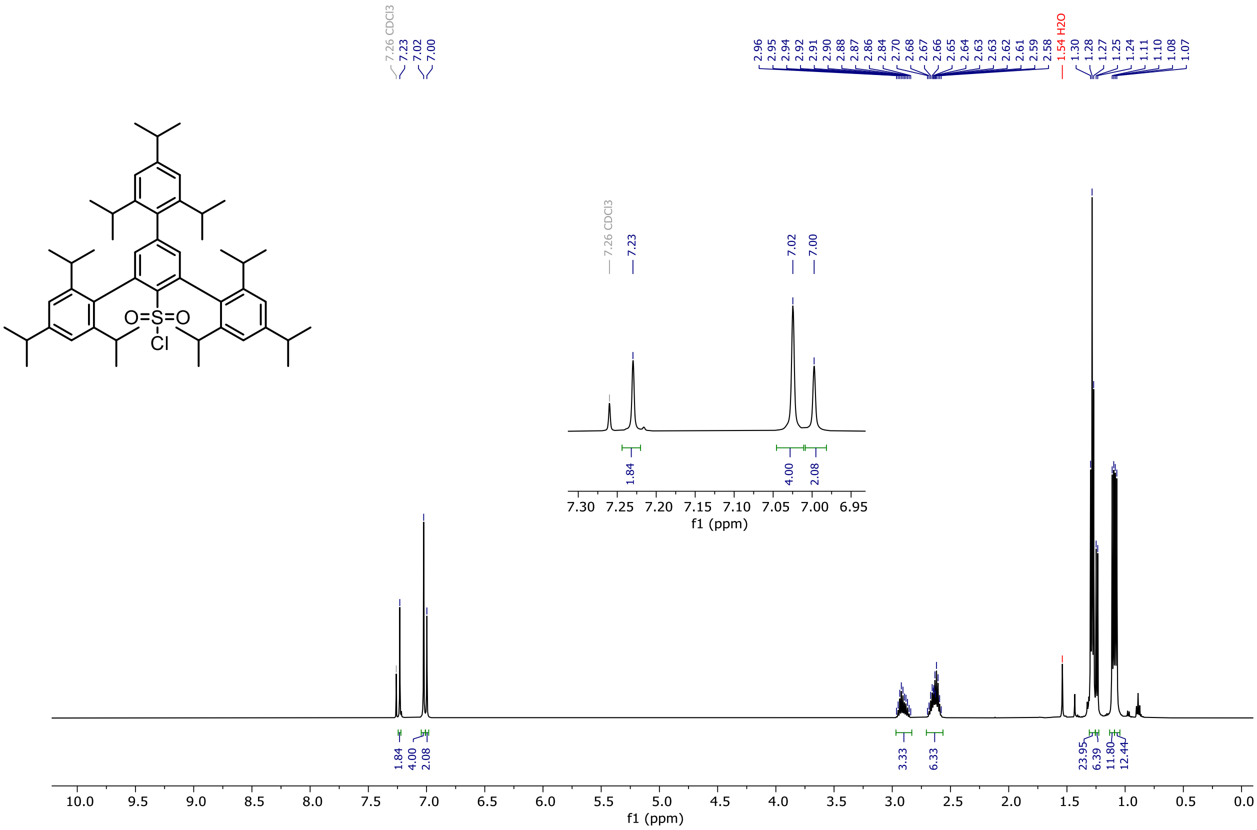

^{13}C NMR (126 MHz, $CDCl_3$) of **A5_SO₂Cl**

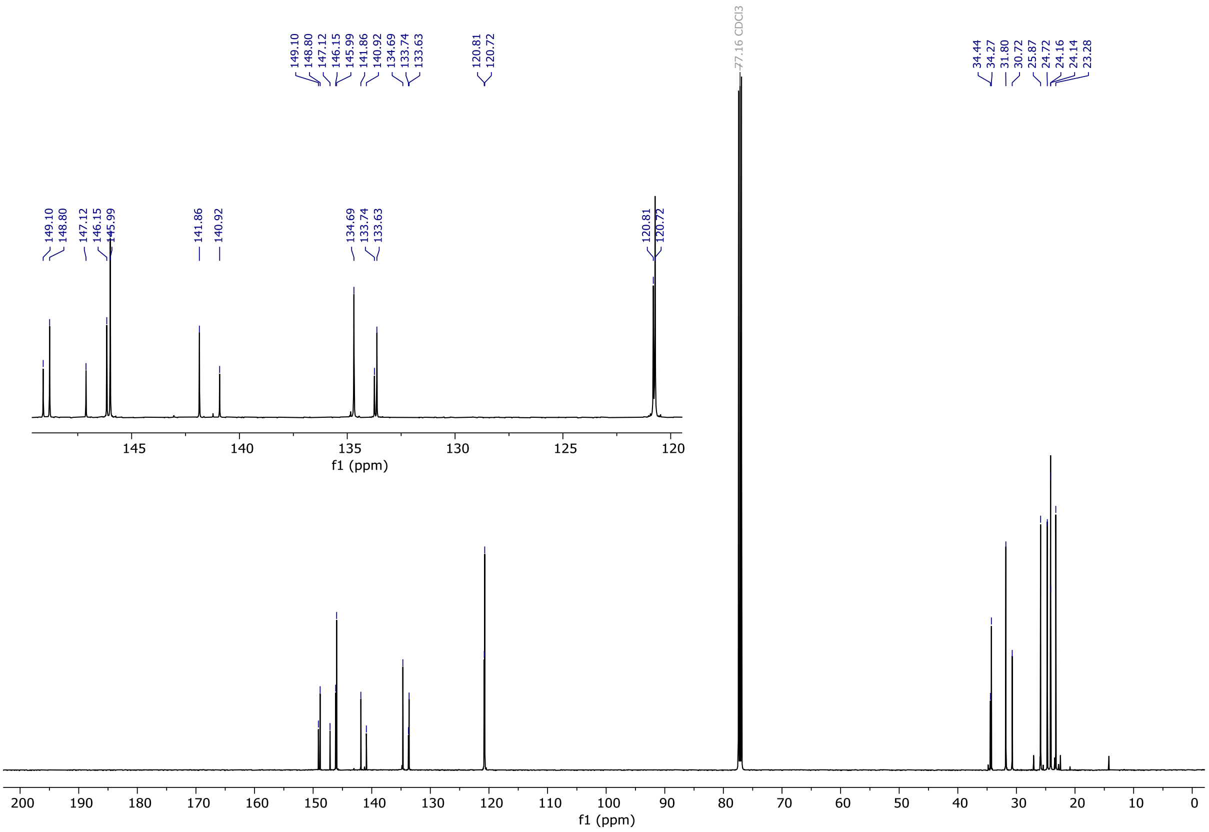

^{1}H NMR (500 MHz, $CDCl_3$) of **Na^{+}A5^{-}**

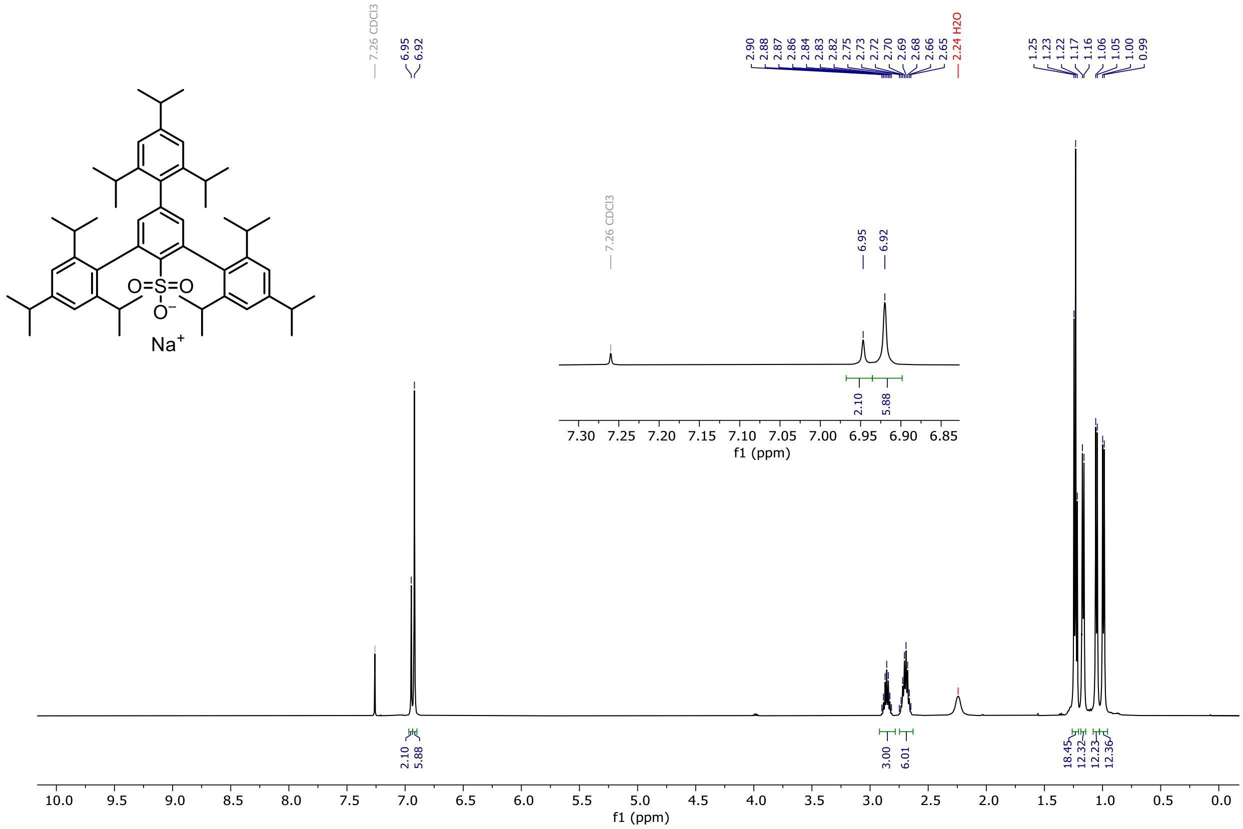

^{13}C NMR (126 MHz, $CDCl_3$) of **Na^{+}A5^{-}**

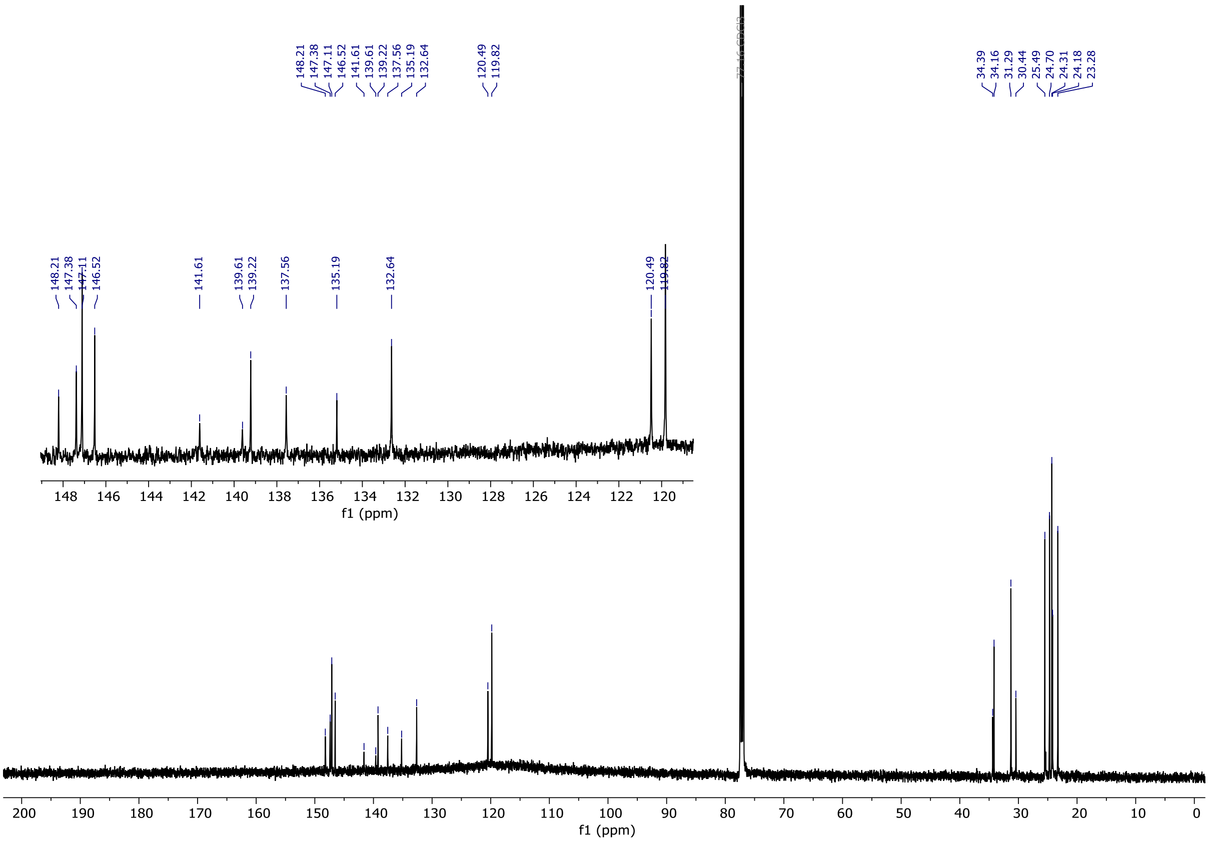

^{1}H NMR (400 MHz, $CDCl_3$) of **A6_Br** (in a mixture with a side product in which there is H instead of Br)

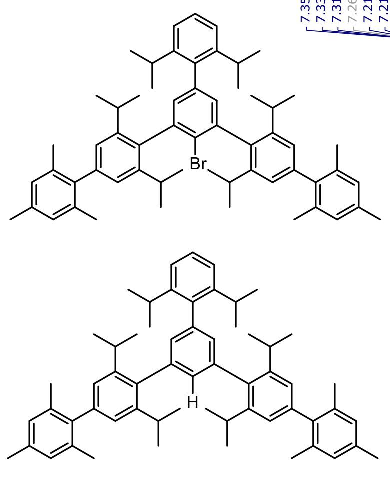

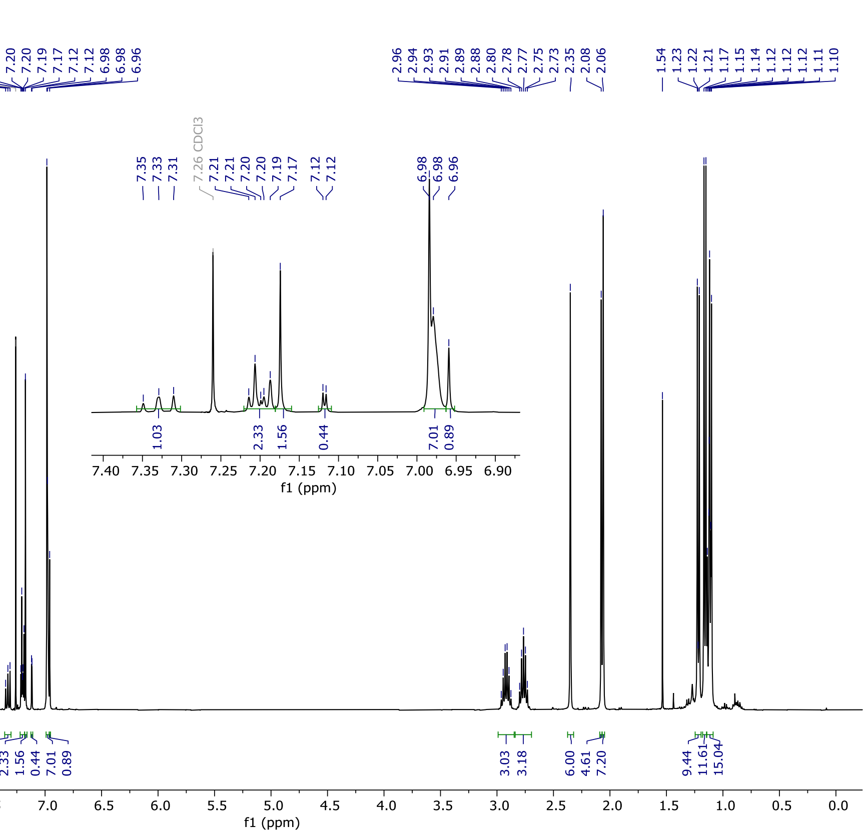

^{13}C NMR (101 MHz, $CDCl_3$) of **A6_Br** (in a mixture with a side product in which there is H instead of Br)

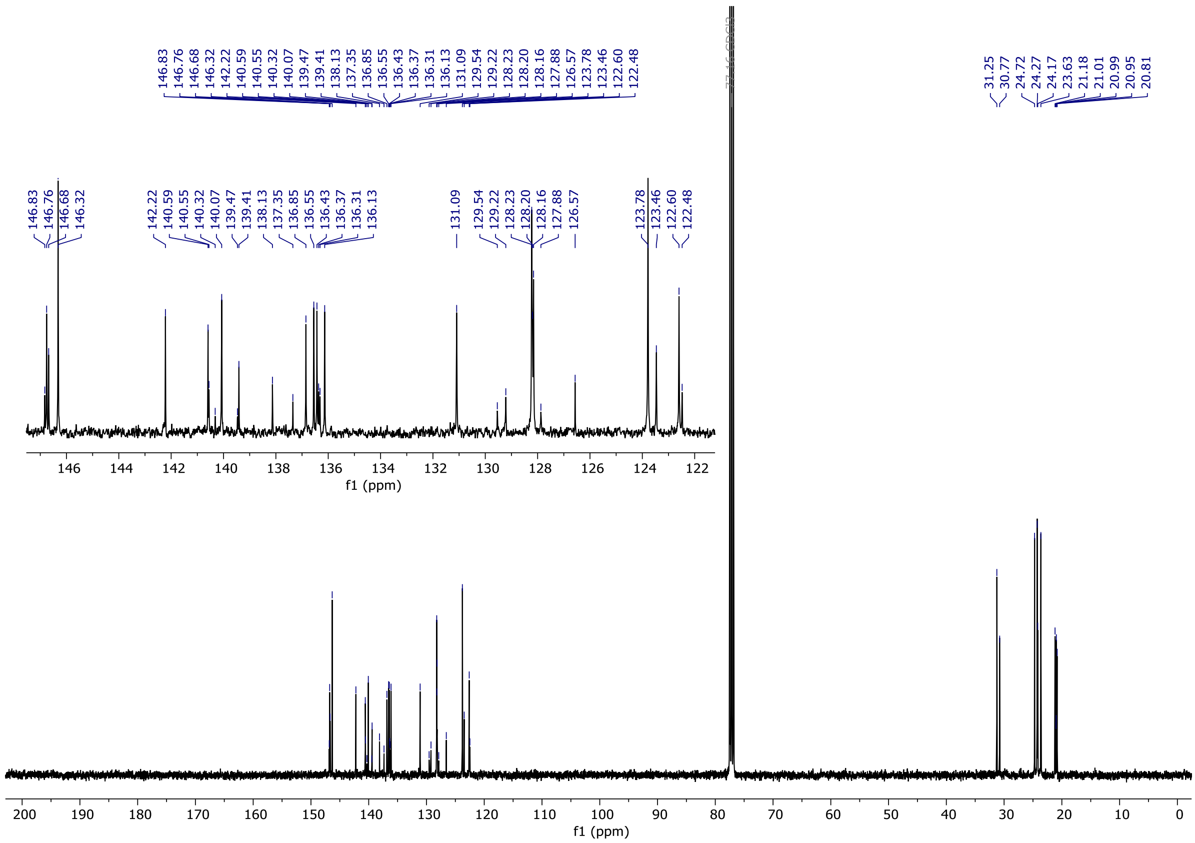

^{1}H NMR (400 MHz, $CDCl_3$) of **A6_SO₂Cl**

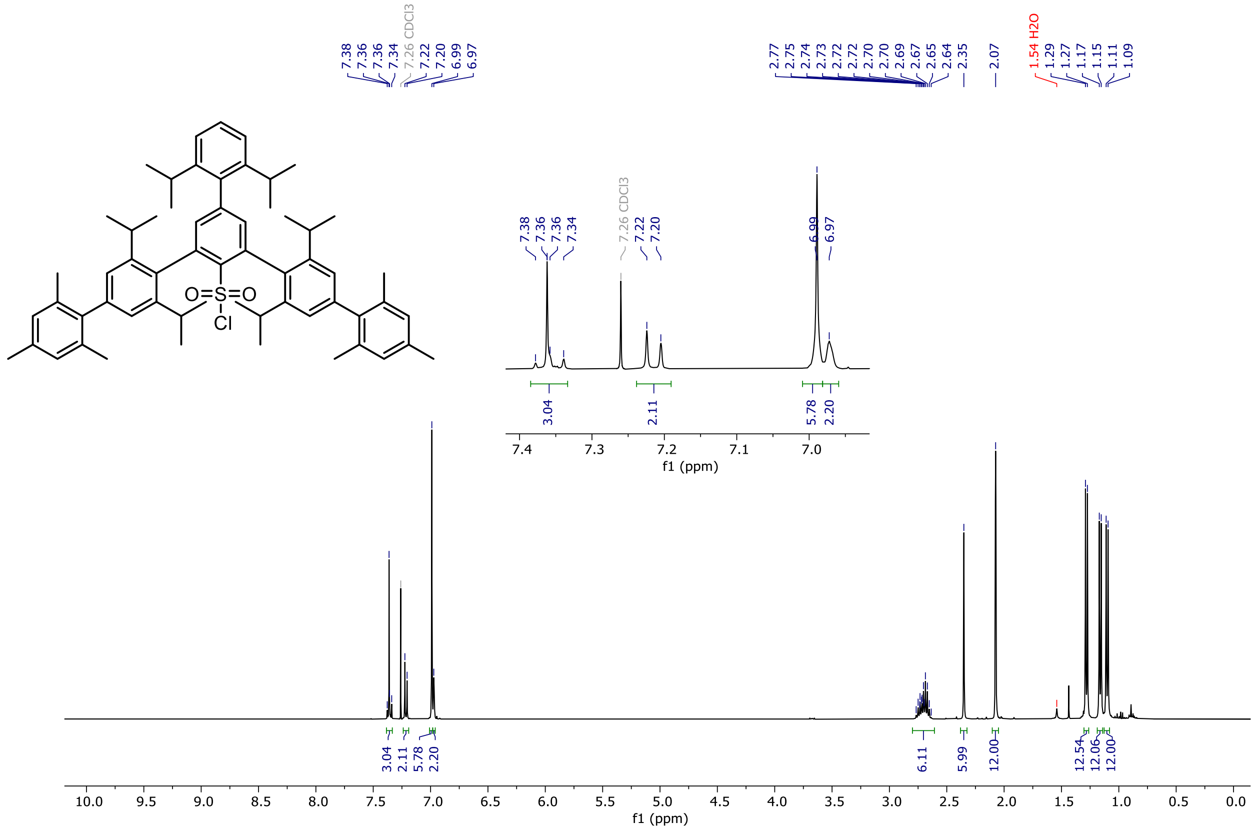

^{13}C NMR (101 MHz, $CDCl_3$) of **A6_SO₂Cl**

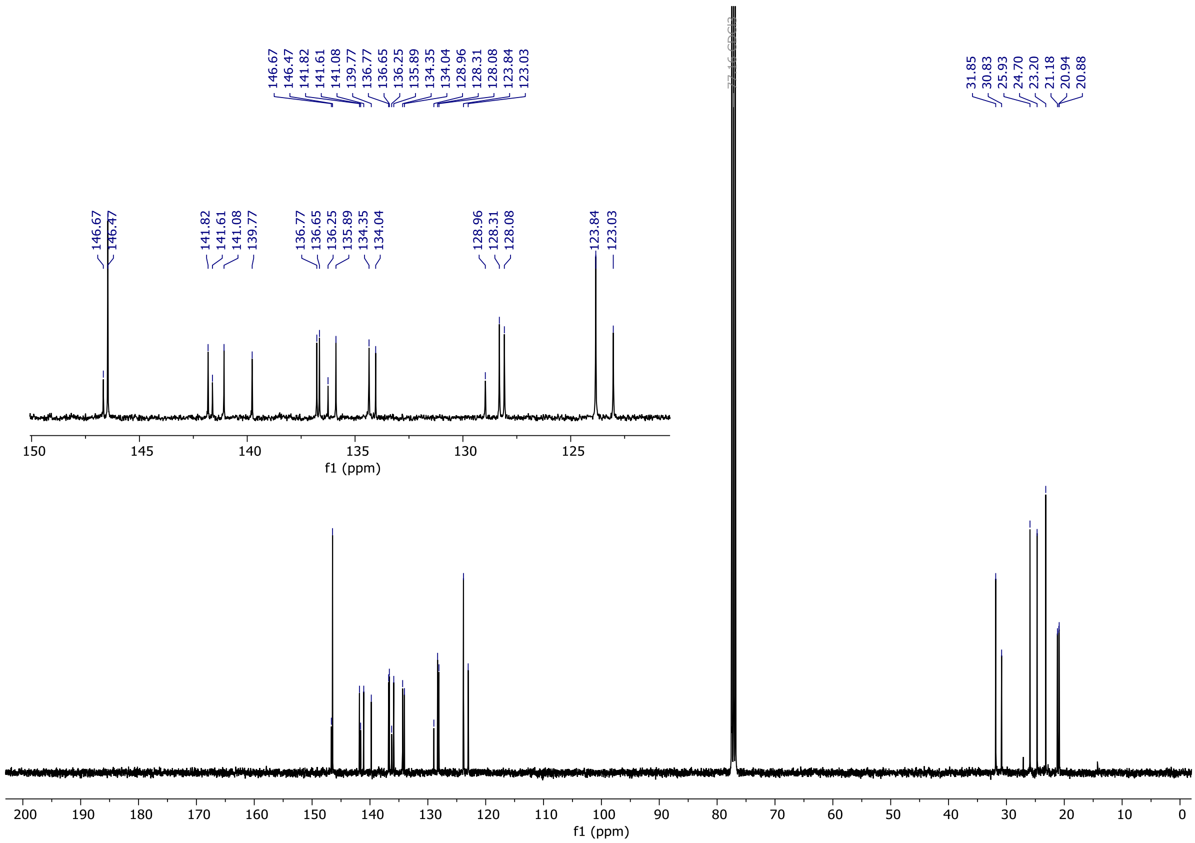

^{1}H NMR (400 MHz, $CDCl_3$) of **Na^+A6$^-$**

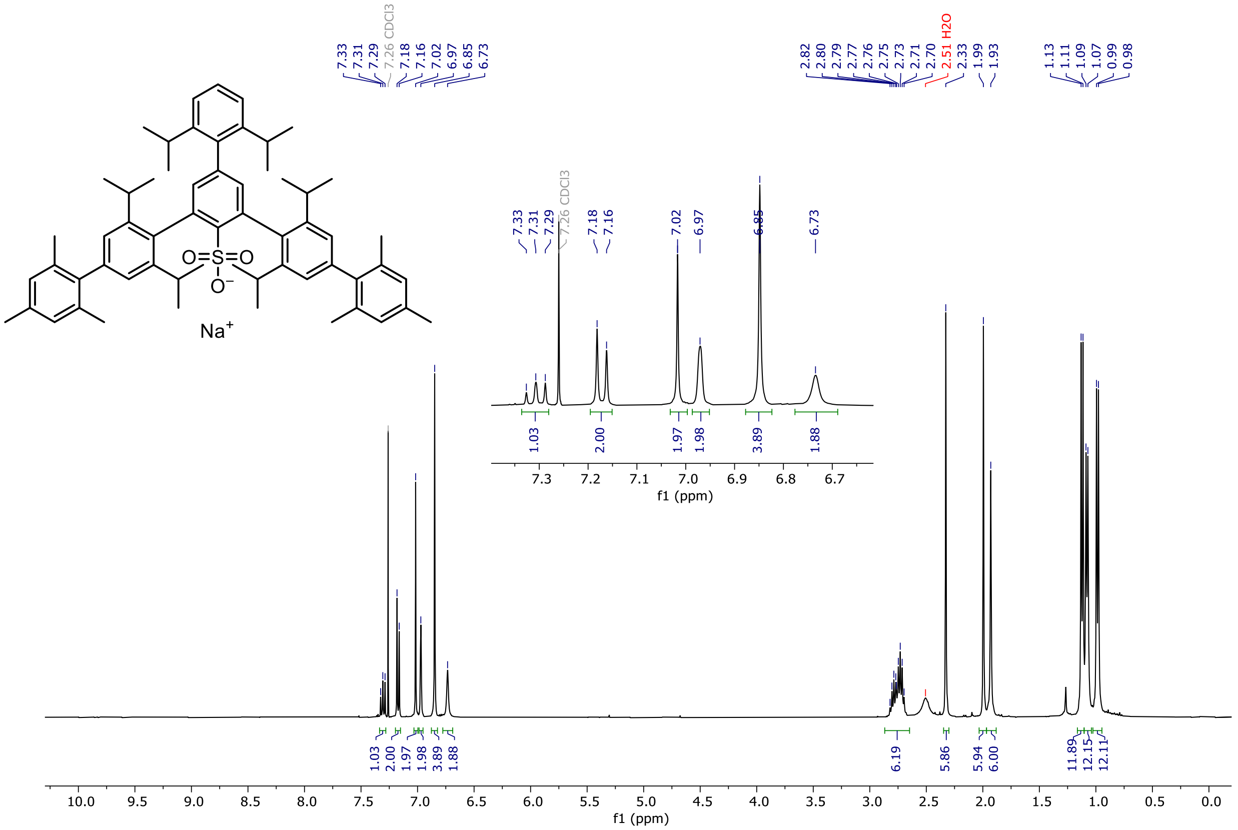

^{13}C NMR (101 MHz, $CDCl_3$) of **Na^+A6$^-$**

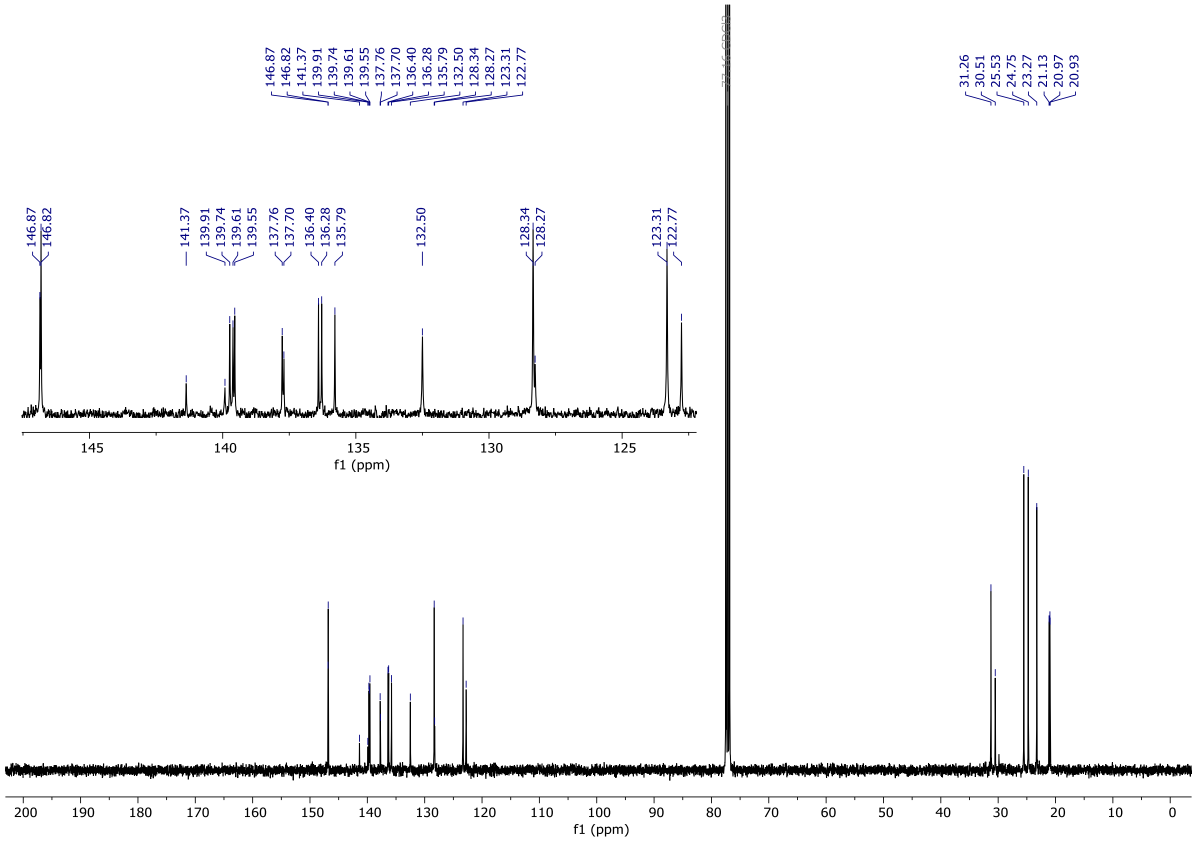

^{1}H NMR (400 MHz, $CDCl_3$) of **A7_Br**

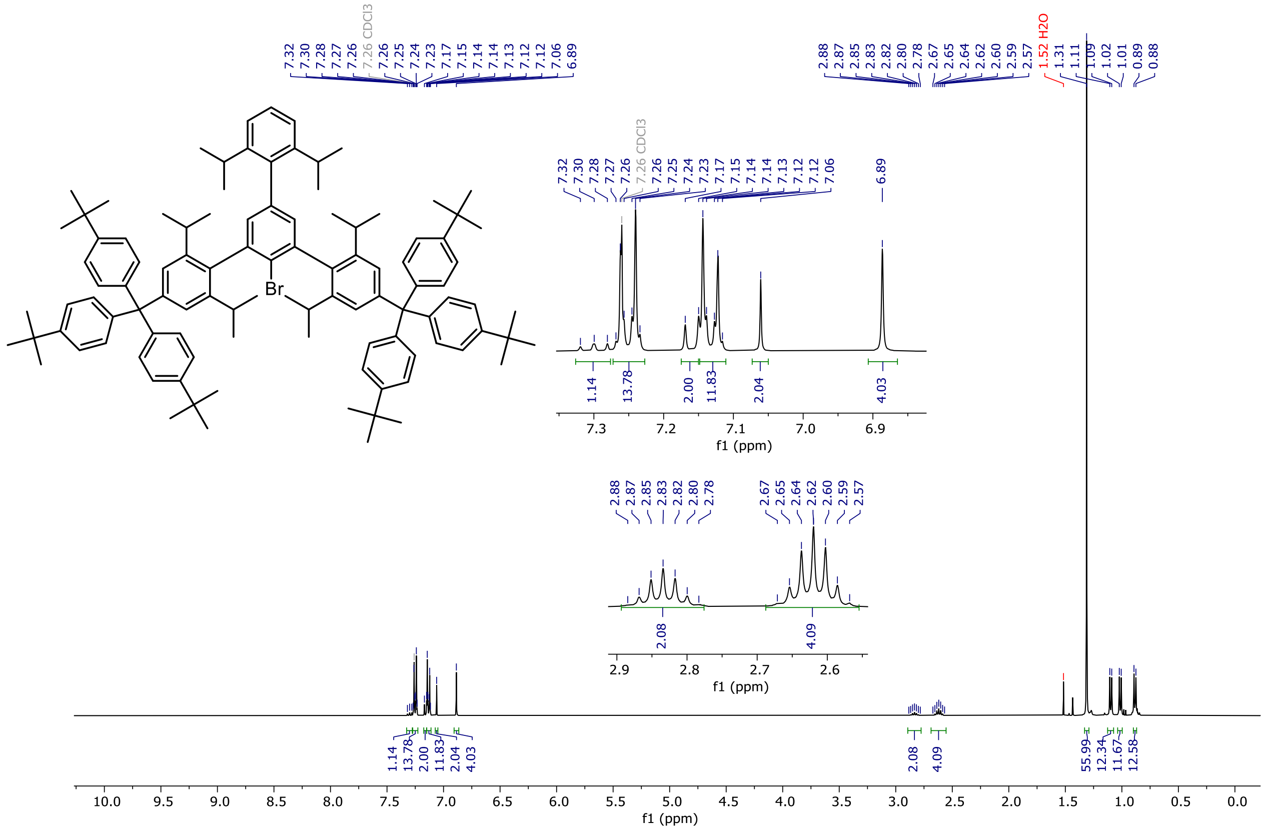

^{13}C NMR (101 MHz, $CDCl_3$) of **A7_Br**

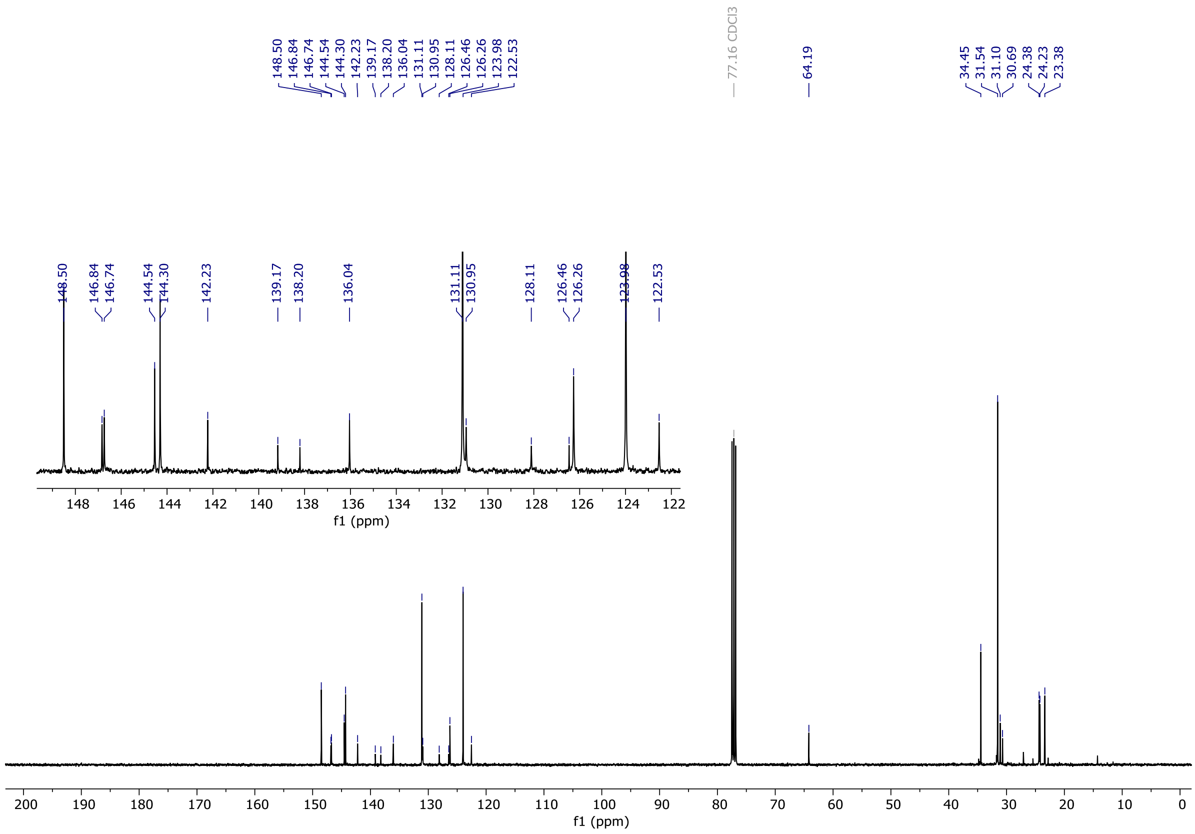

^{1}H NMR (400 MHz, $CDCl_3$) of **A7_SO₂Cl**

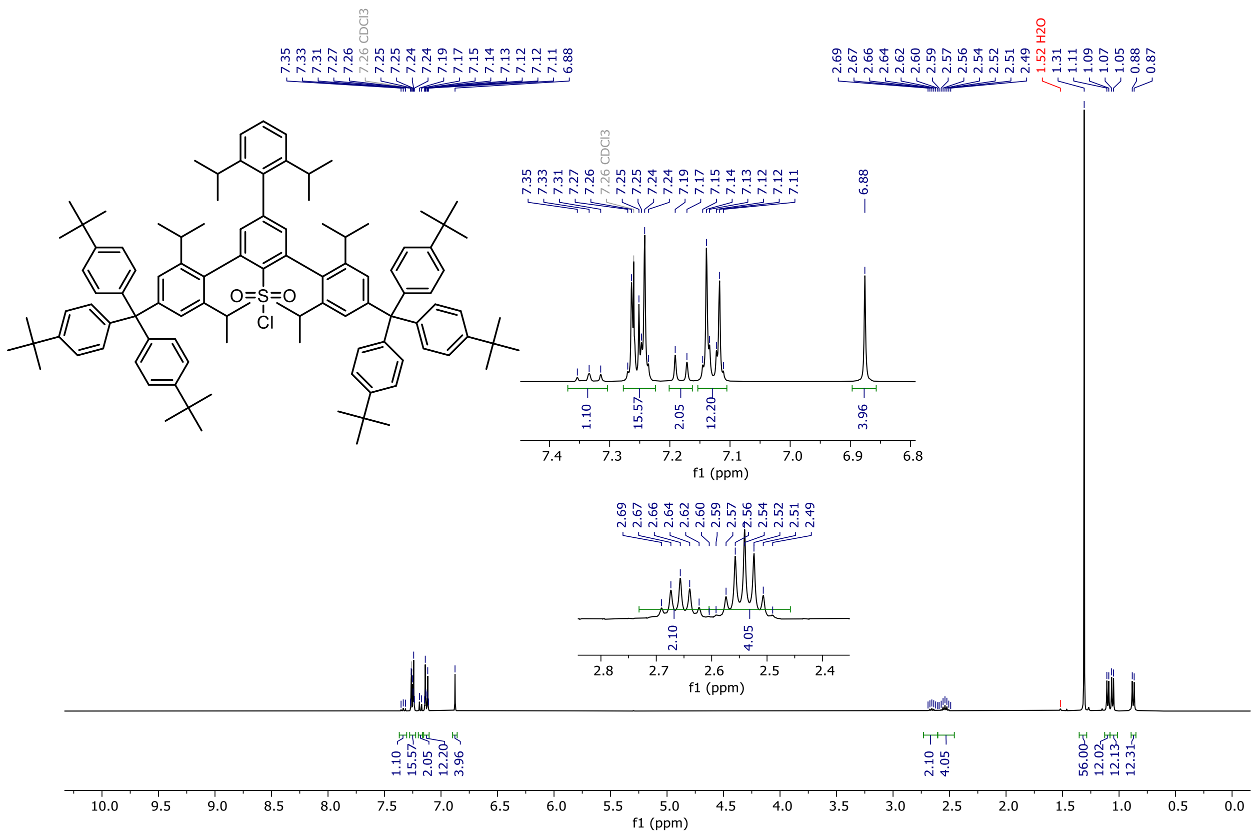

^{13}C NMR (101 MHz, $CDCl_3$) of **A7_SO₂Cl**

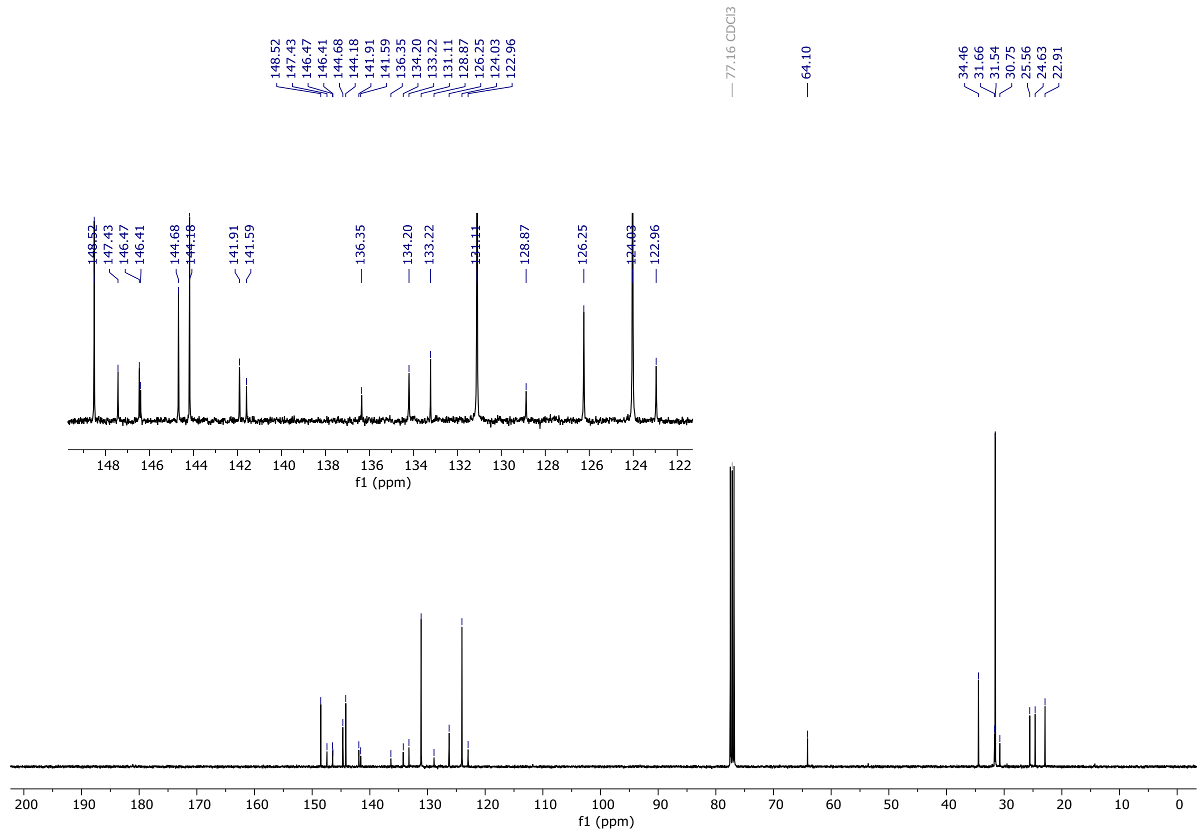

^{1}H NMR (400 MHz, $CDCl_3$) of **Na⁺A7⁻**

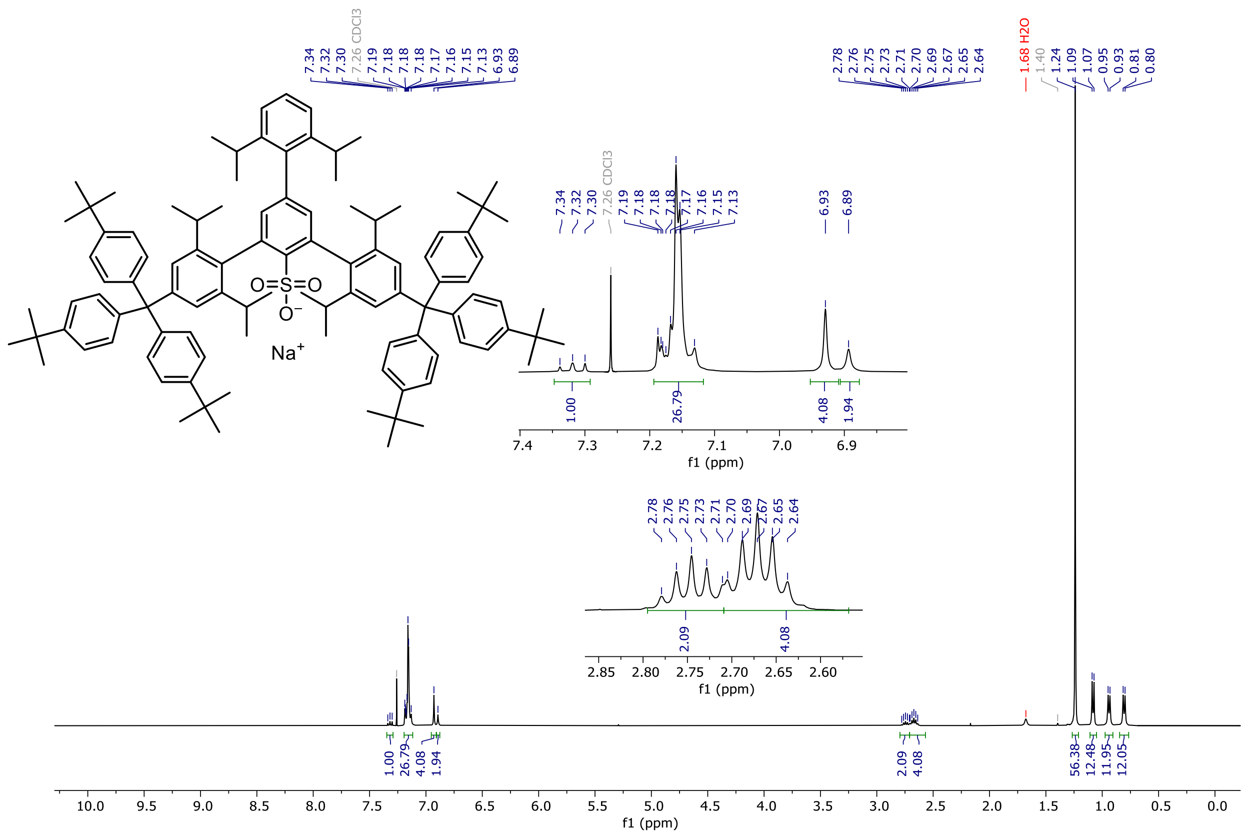

^{13}C NMR (101 MHz, $CDCl_3$) of **Na⁺A7⁻**

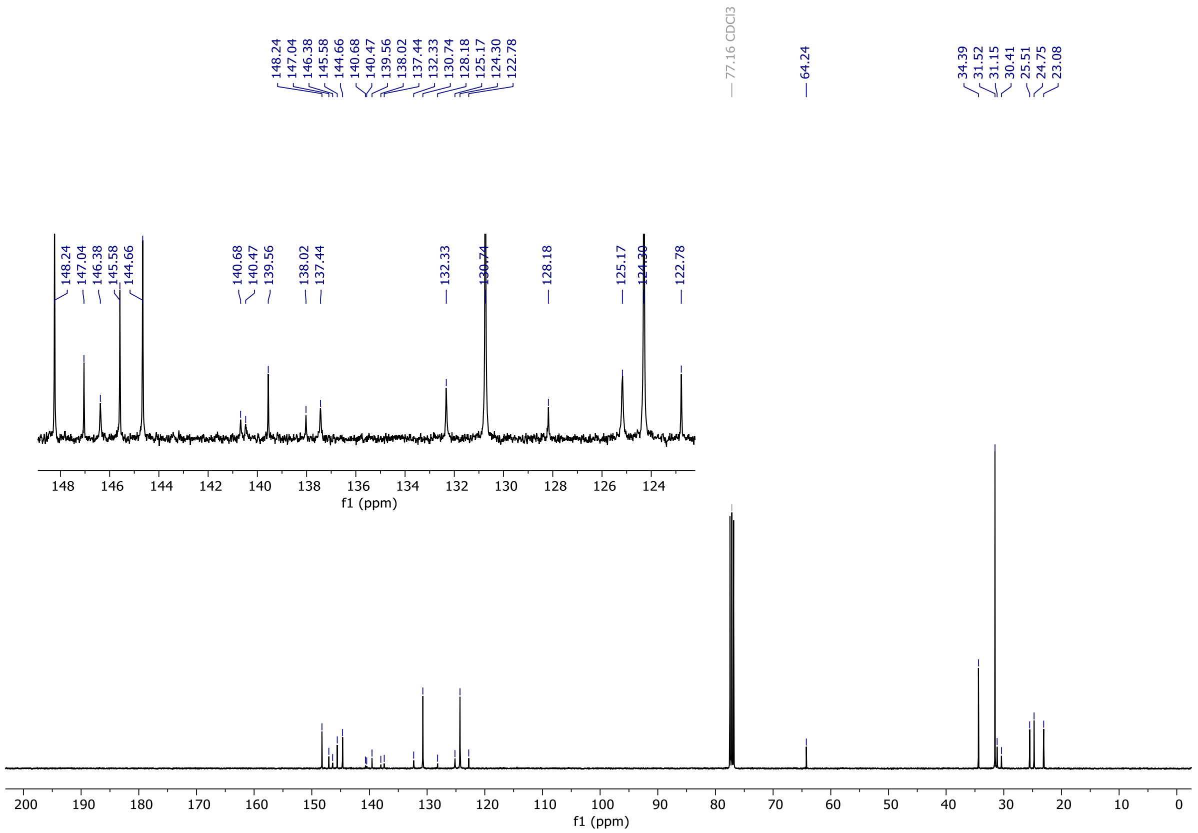

^{1}H NMR (500 MHz, $CDCl_3$) of **Bpin_C4_N**

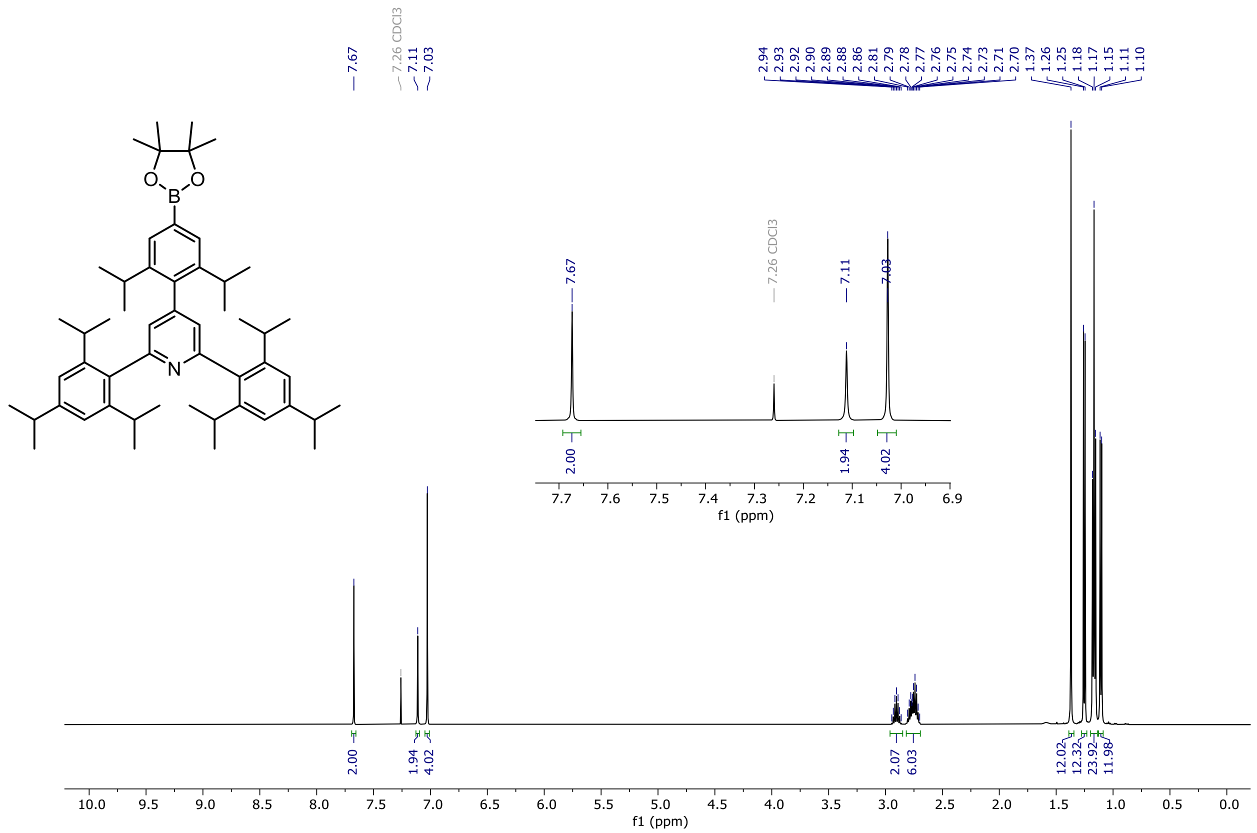

^{13}C NMR (126 MHz, $CDCl_3$) of **Bpin_C4_N**

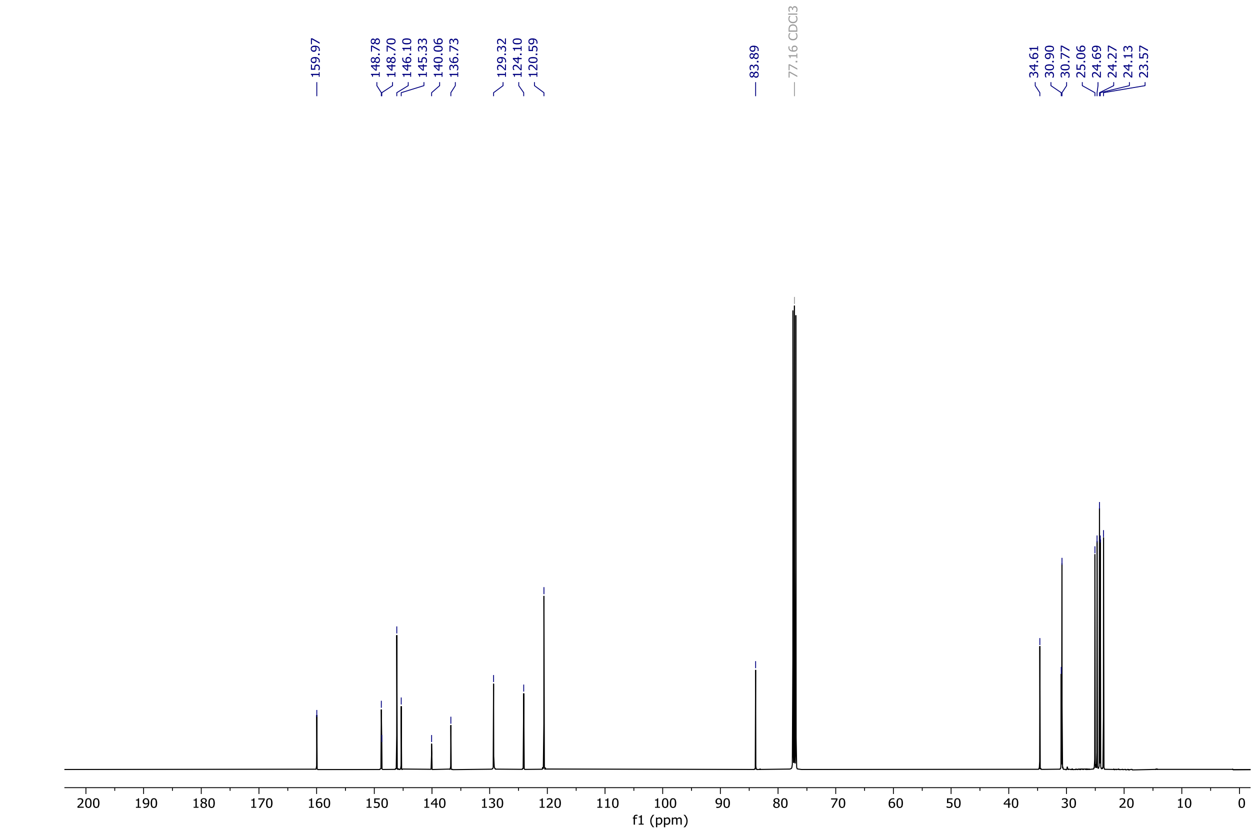

^{1}H NMR (500 MHz, $CDCl_3$) of **C8_N**

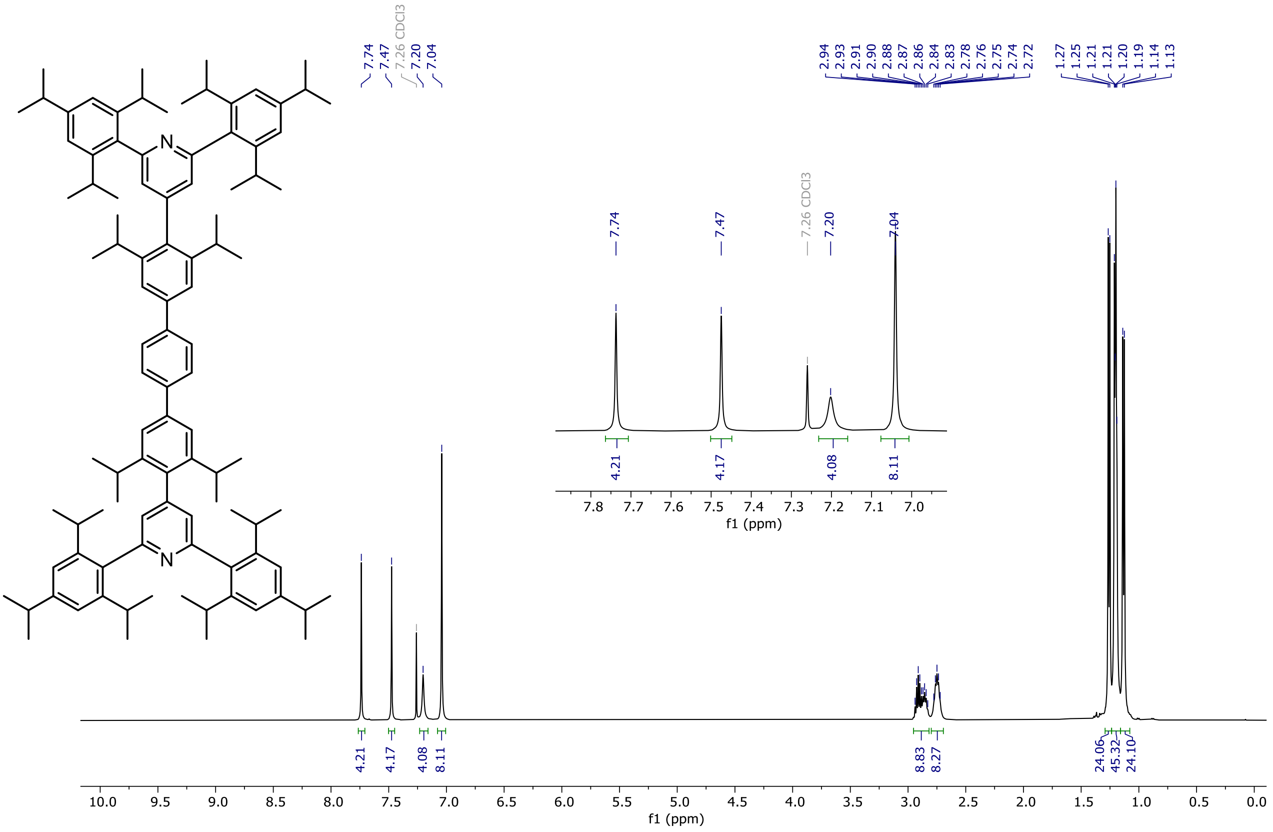

^{13}C NMR (126 MHz, $CDCl_3$) of **C8_N**

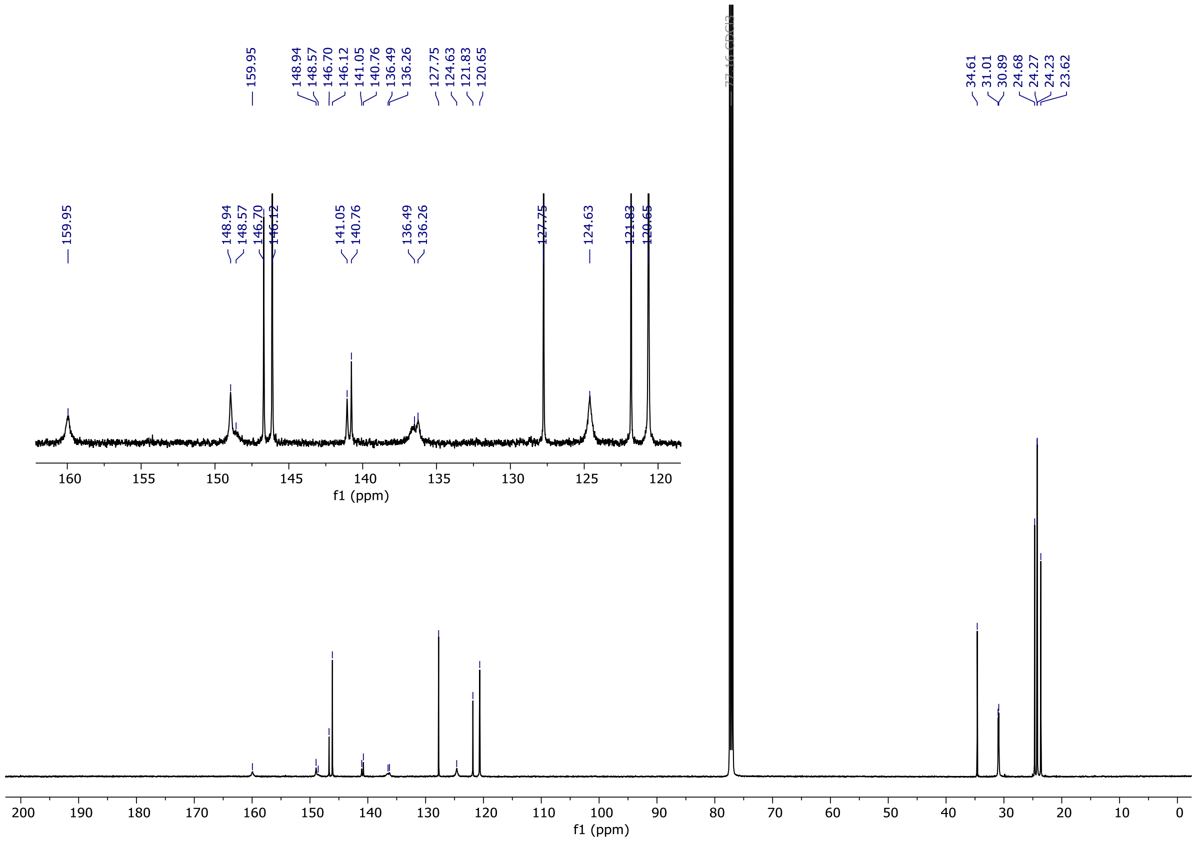

^{1}H NMR (500 MHz, $CDCl_3$) of **$C8^{2+}(BF_4^-)_2$**

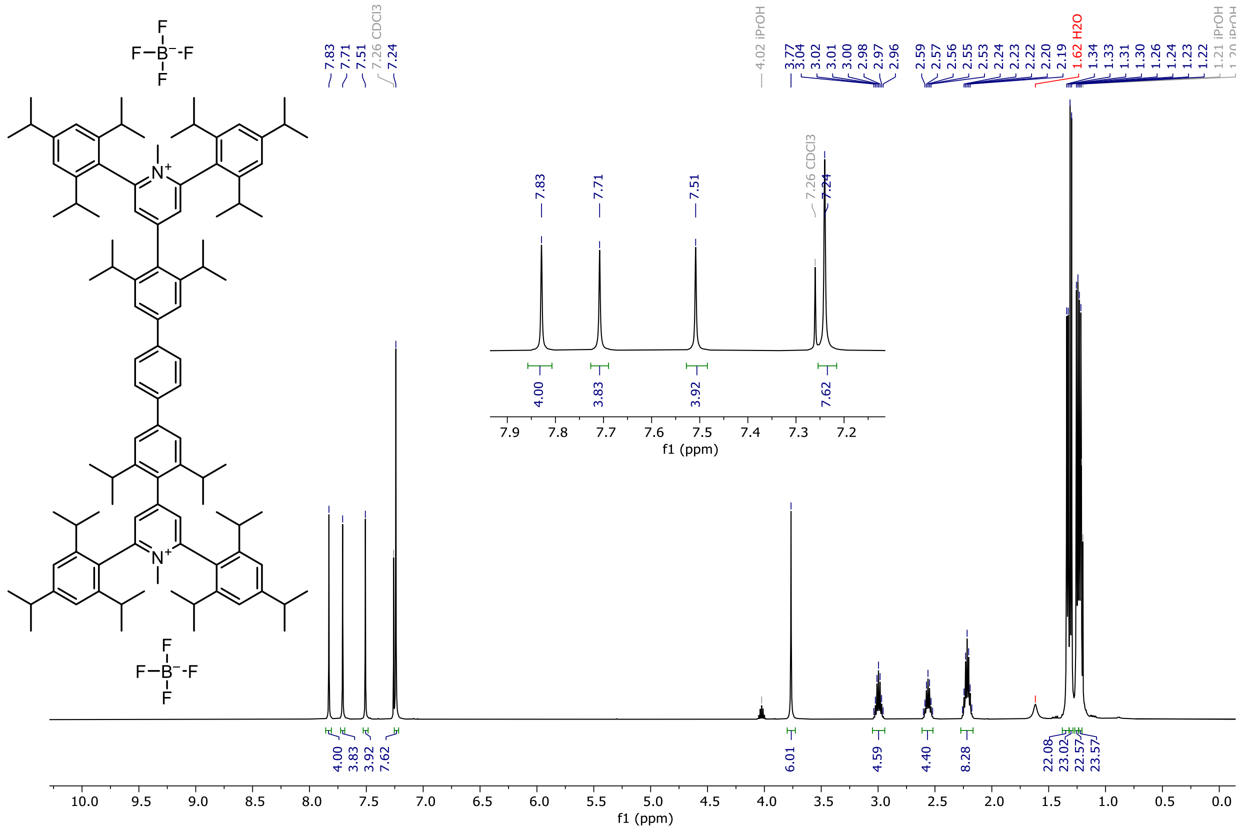

^{13}C NMR (126 MHz, $CDCl_3$) of **$C8^{2+}(BF_4^-)_2$**

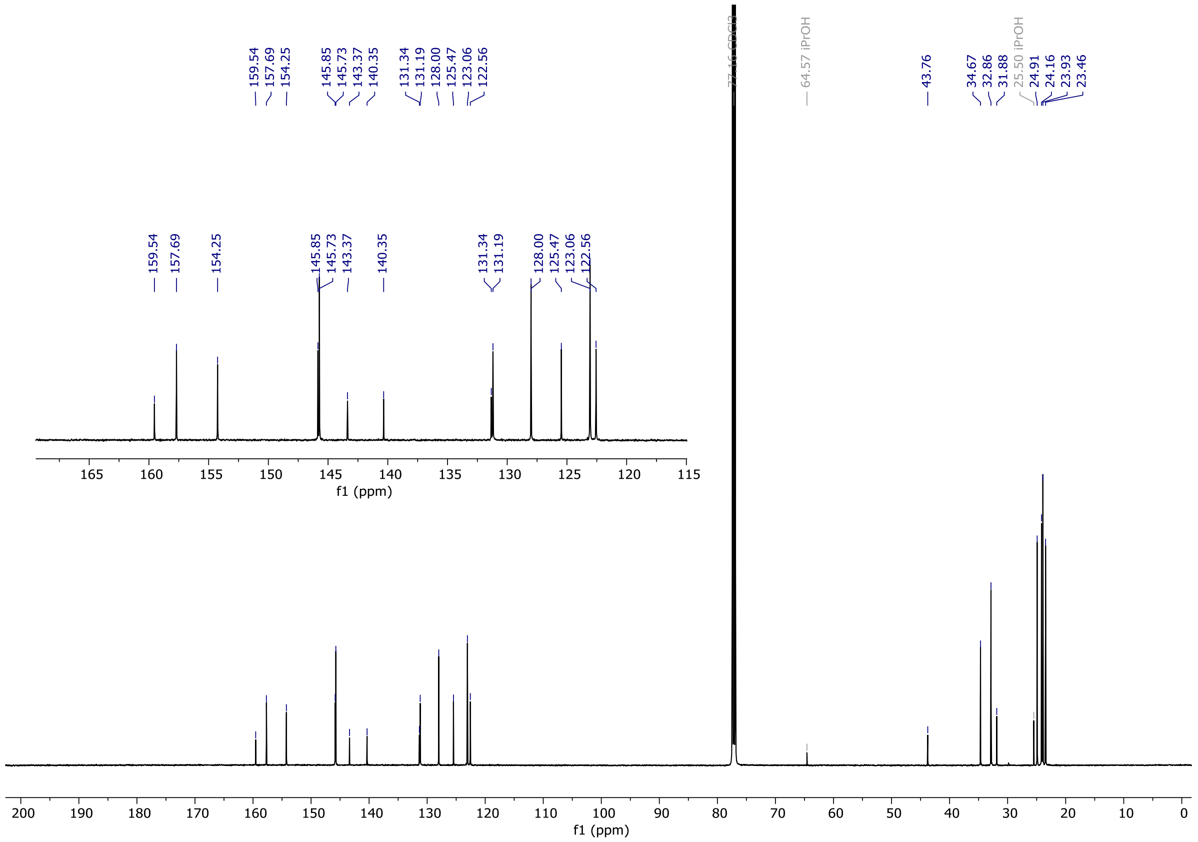

^{19}F NMR (471 MHz, CDCl$_3$) of **C8^{2+}(BF$_4^-$)$_2$**

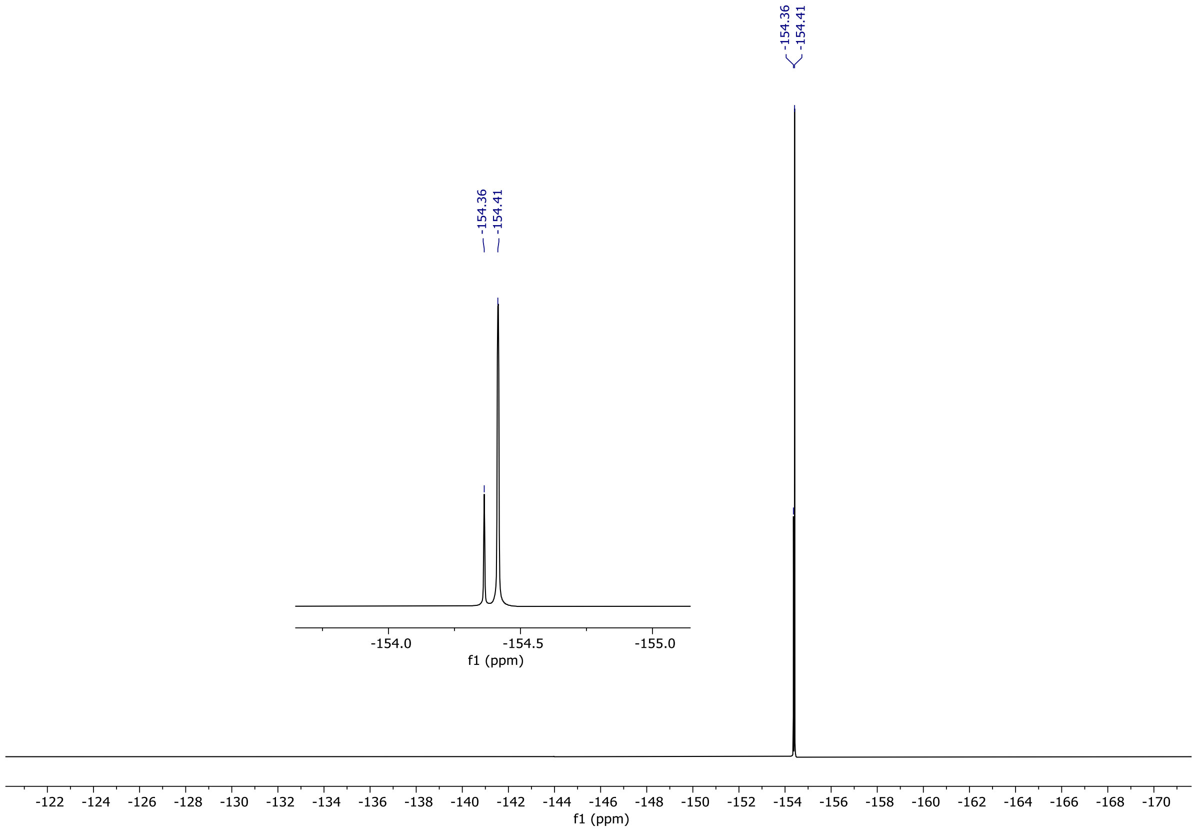

^{1}H NMR (400 MHz, $CDCl_3$) of **C9_N**

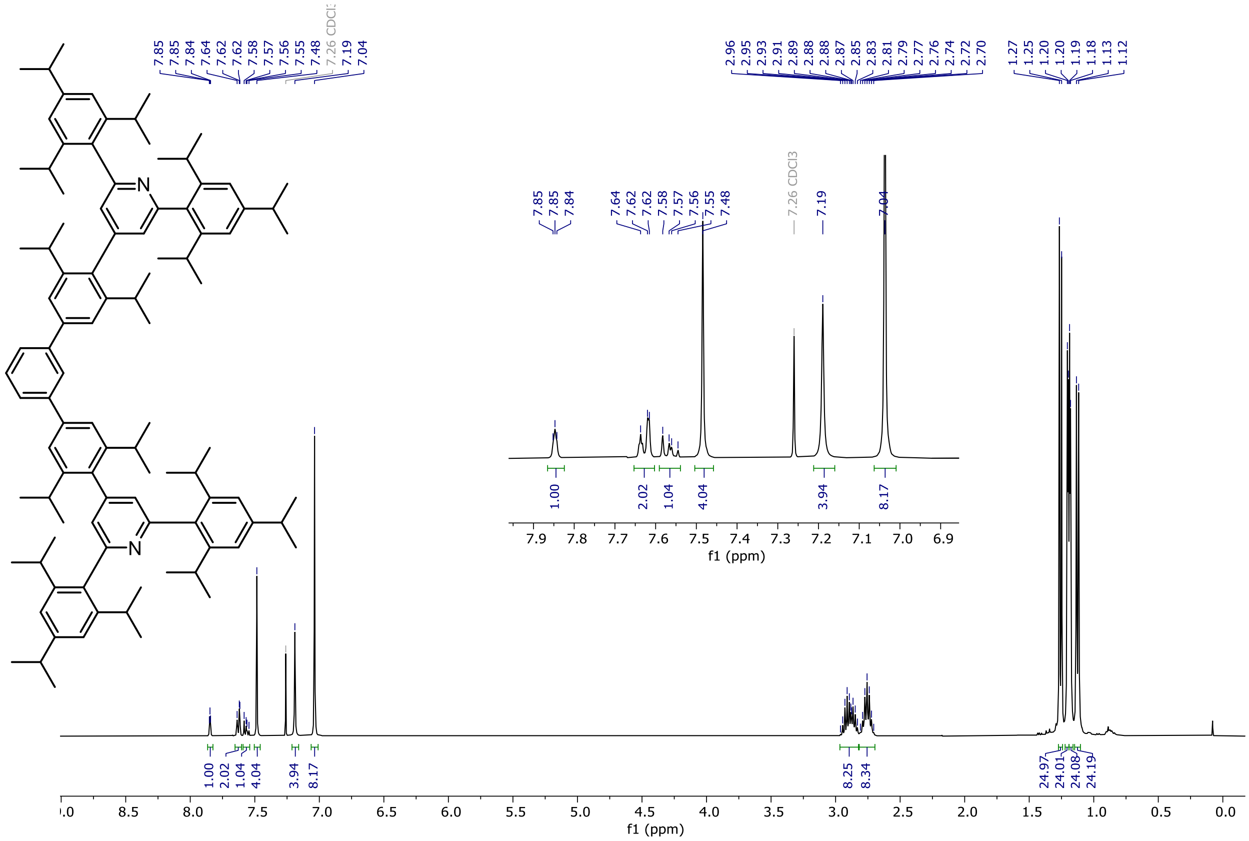

^{13}C NMR (101 MHz, $CDCl_3$) of **C9_N**

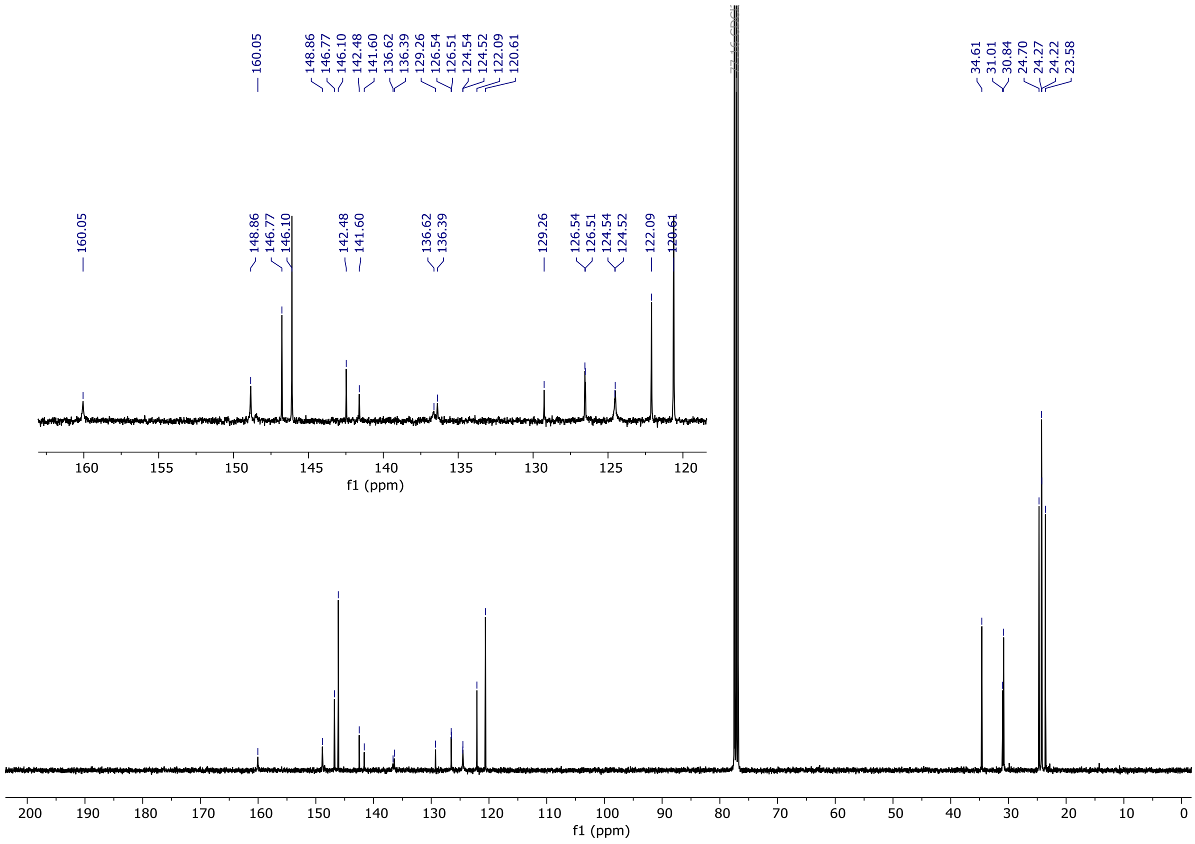

^{1}H NMR (400 MHz, $CDCl_3$) of **$C9^{2+}(BF_4^-)_2$**

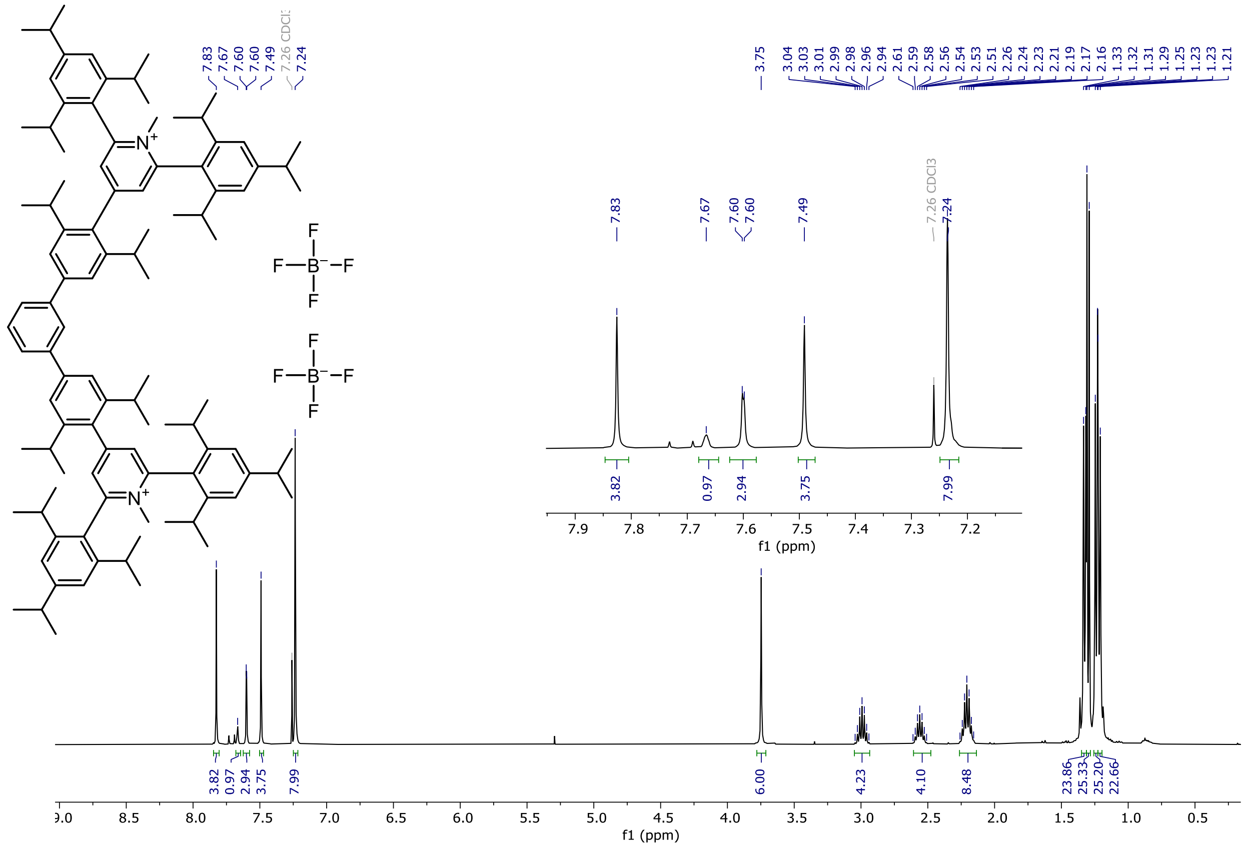

^{13}C NMR (101 MHz, $CDCl_3$) of **$C9^{2+}(BF_4^-)_2$**

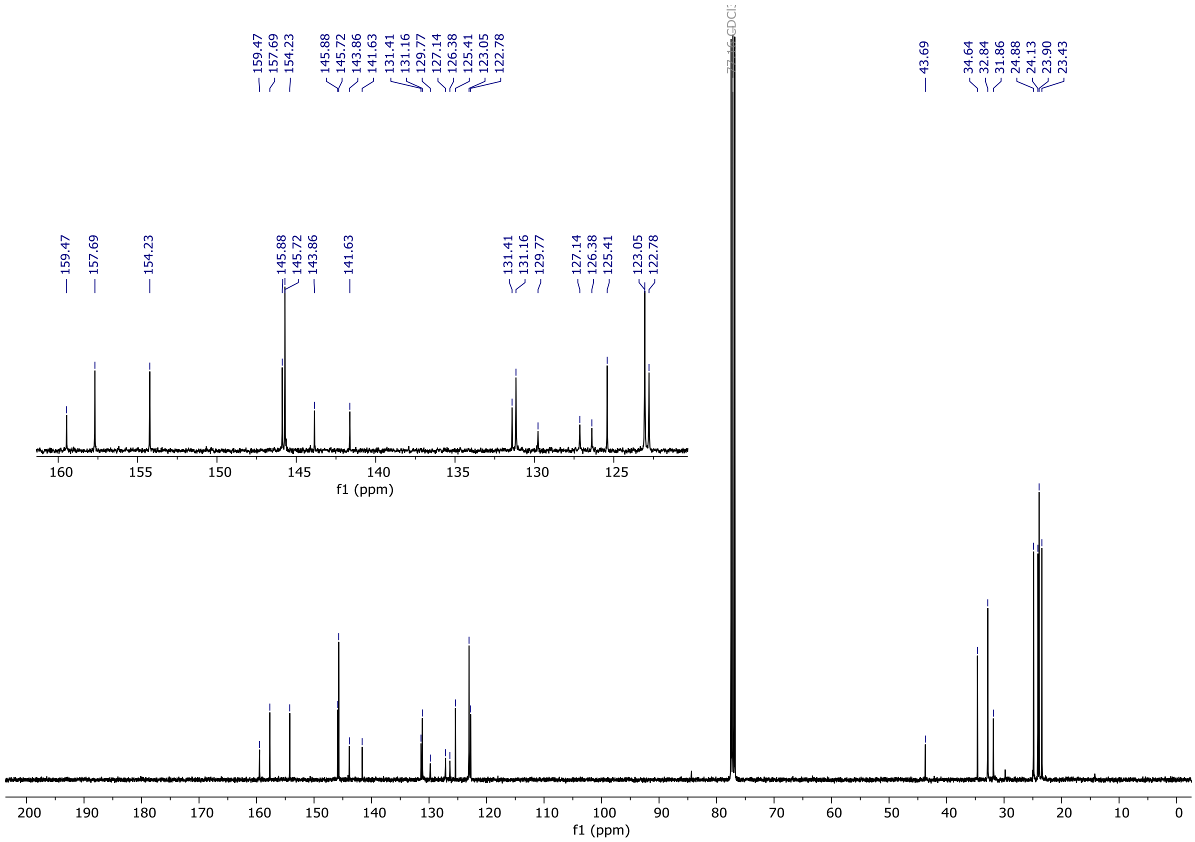

^{19}F NMR (376 MHz, $CDCl_3$) of **C9^{2+}(BF_4^-)$_2$**

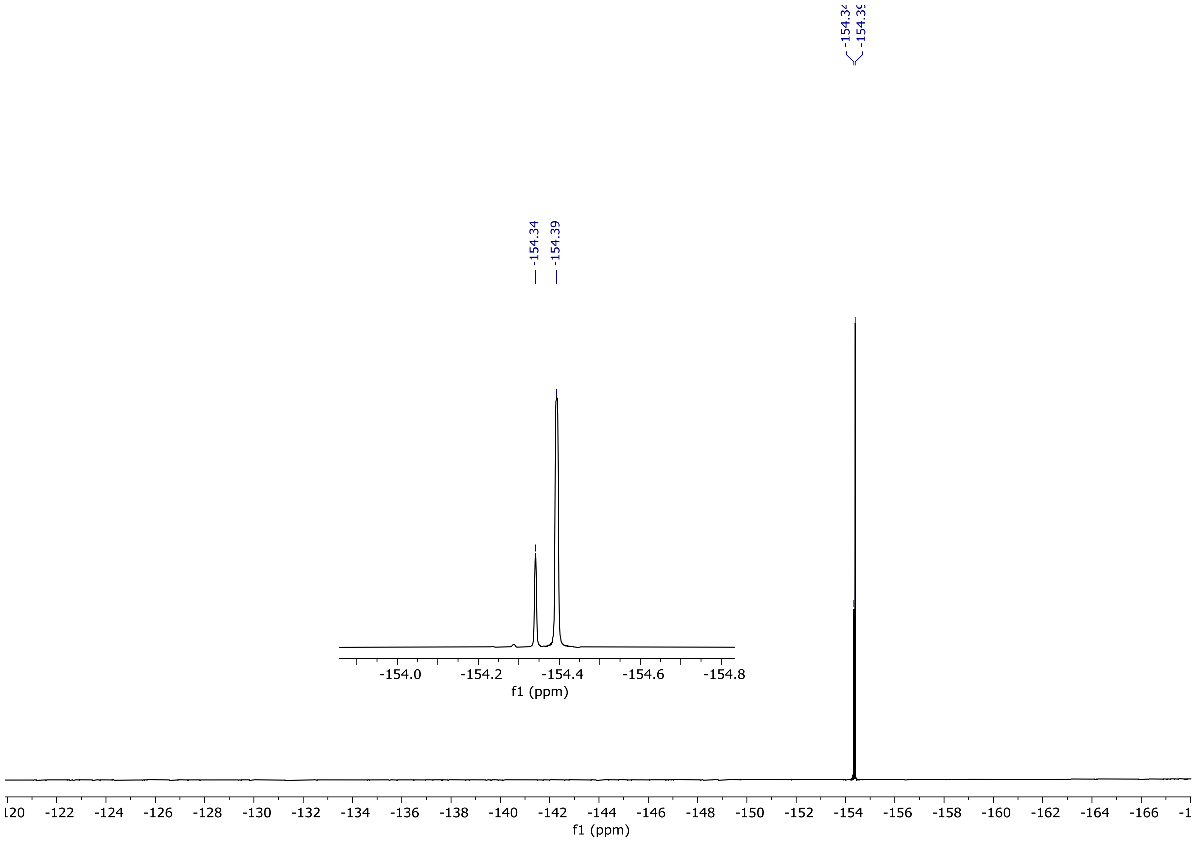

^{1}H NMR (500 MHz, $CDCl_3$) of **C10_N**

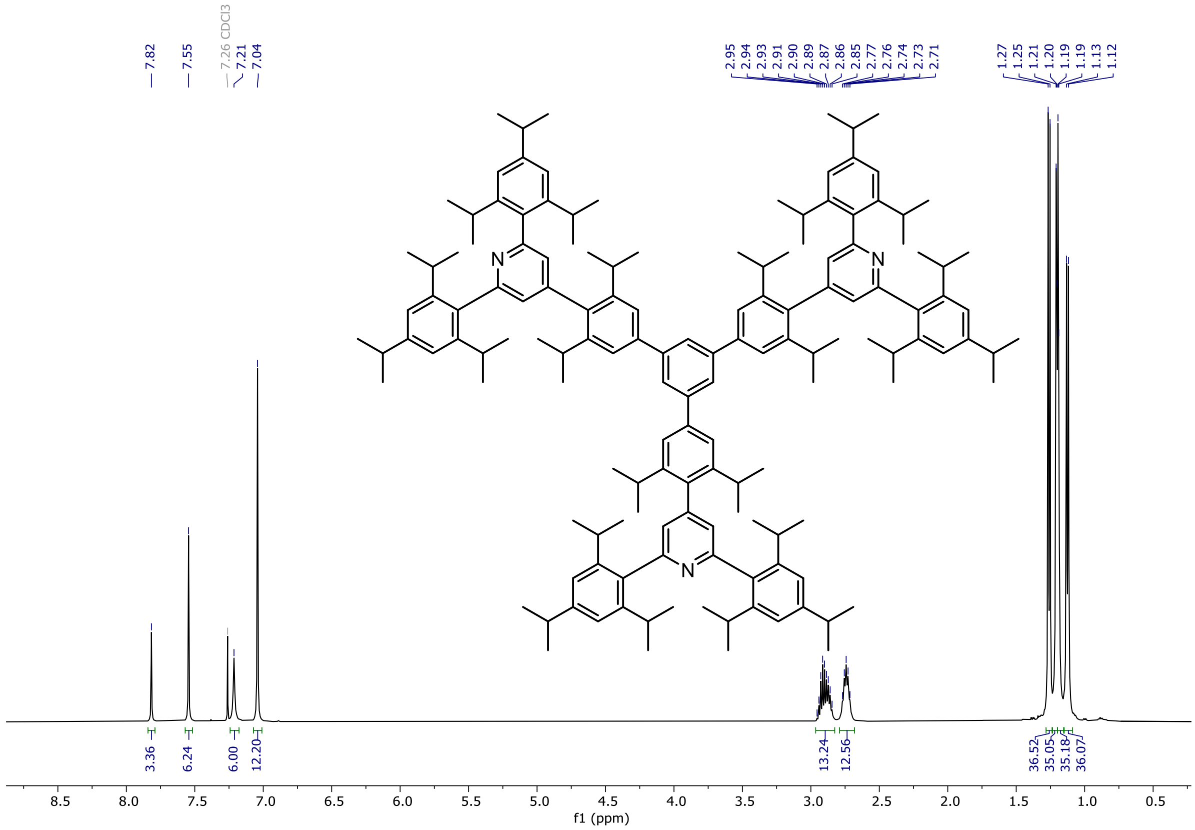

^{13}C NMR (126 MHz, $CDCl_3$) of **C10_N**

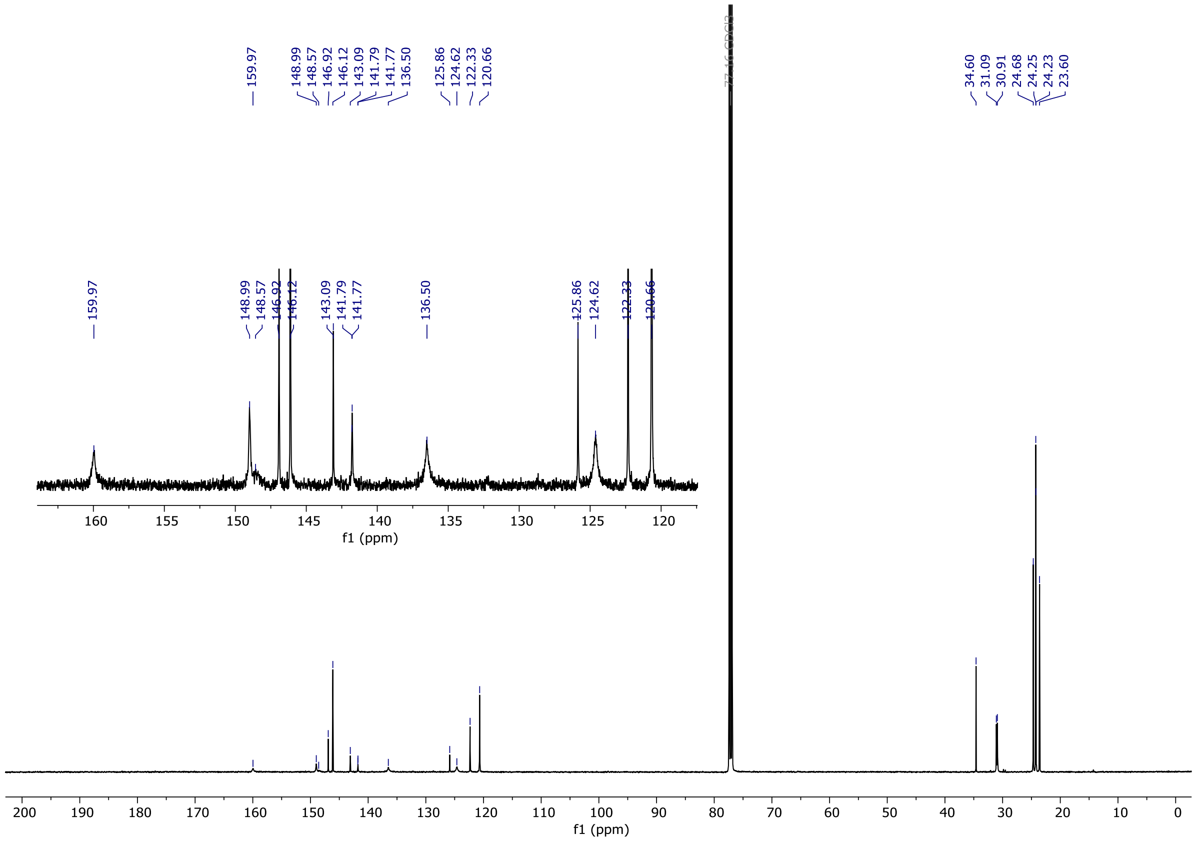

^{1}H NMR (500 MHz, $CDCl_3$) of **C10^{3+}(BF$_4^-$)$_3$**

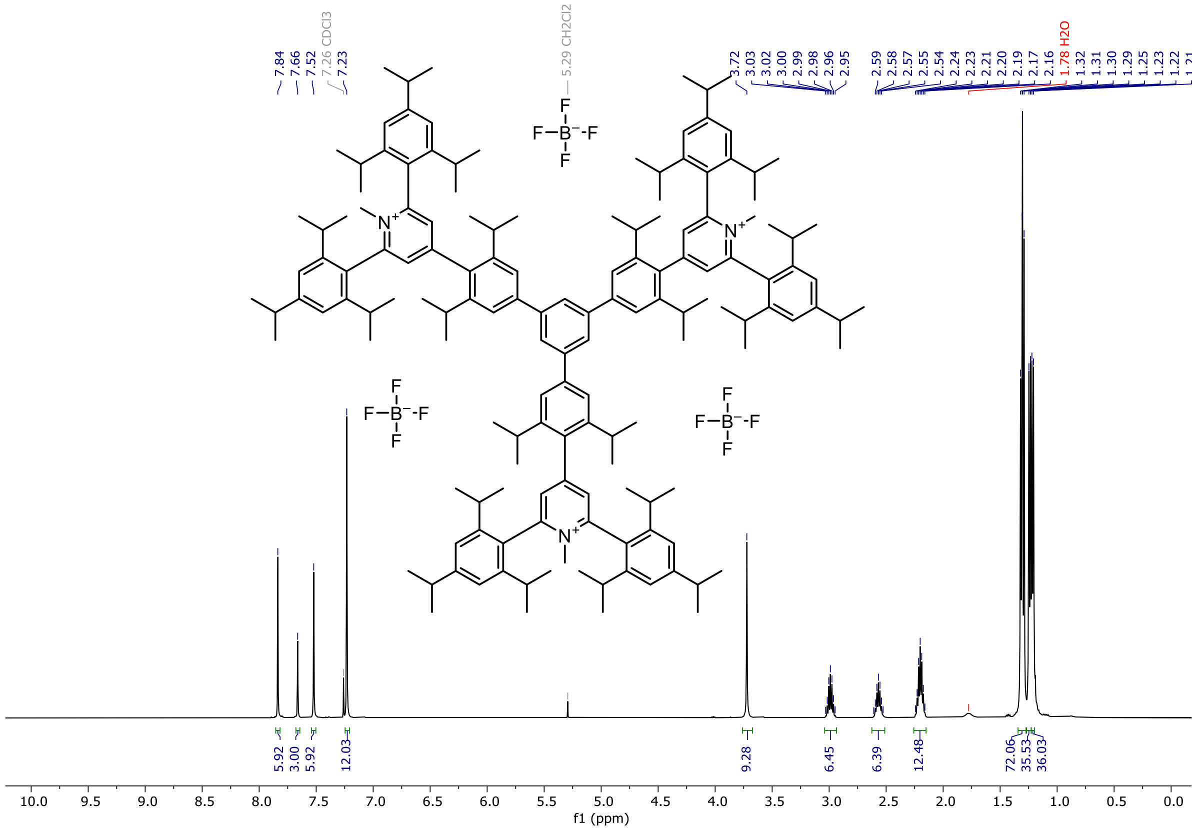

^{13}C NMR (126 MHz, $CDCl_3$) of **C10^{3+}(BF$_4^-$)$_3$**

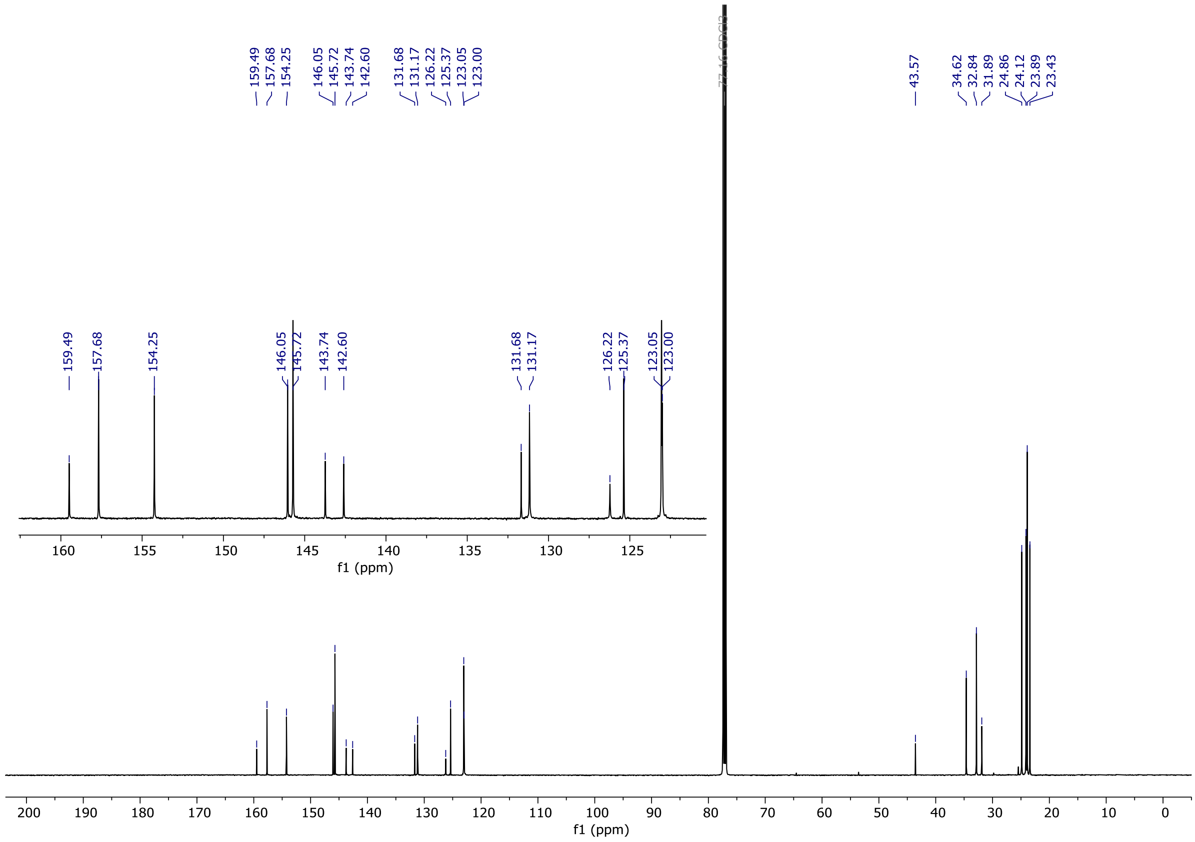

^{19}F NMR (471 MHz, $CDCl_3$) of **C10^{3+}(BF_4^-)$_3$**

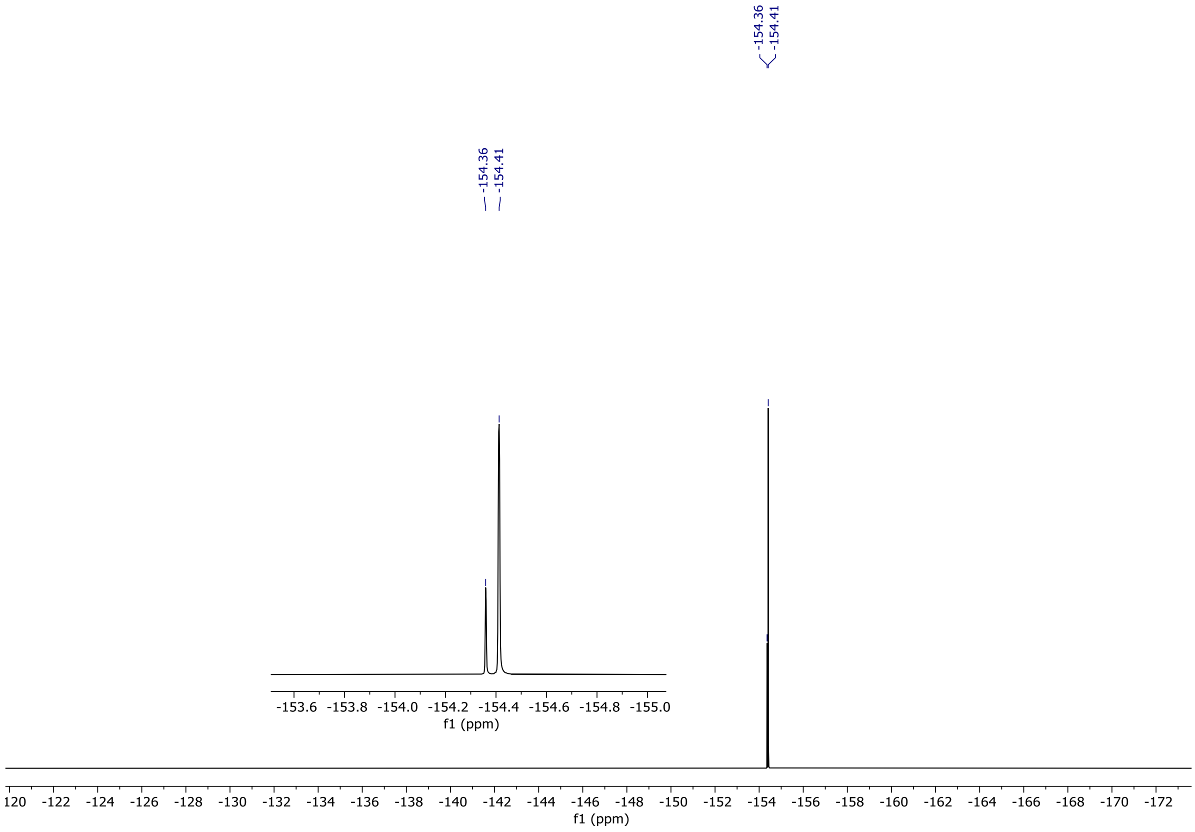

^{1}H NMR (400 MHz, $CDCl_3$) of **A4_SO₃Et**

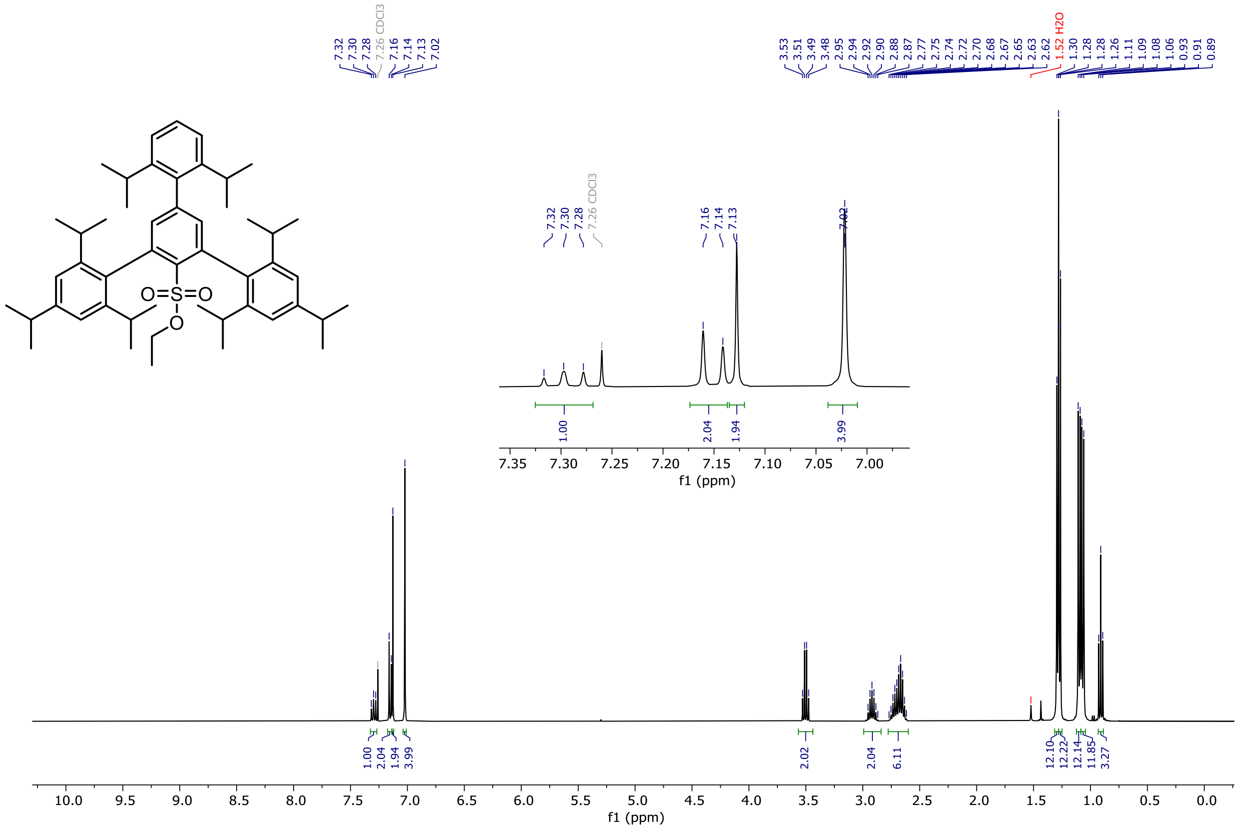

^{13}C NMR (101 MHz, $CDCl_3$) of **A4_SO₃Et**

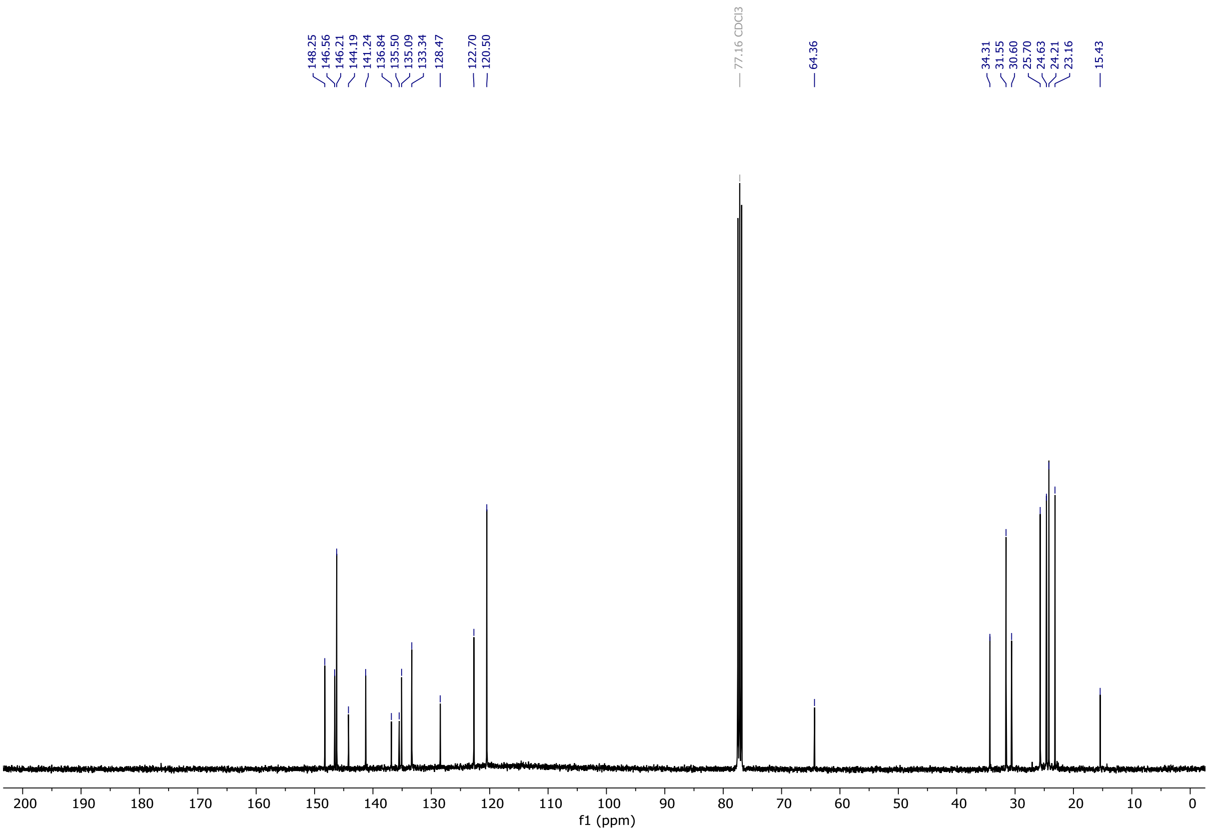

^{1}H NMR (500 MHz, $CDCl_3$) of **Bpin_A4_SO₃Et**

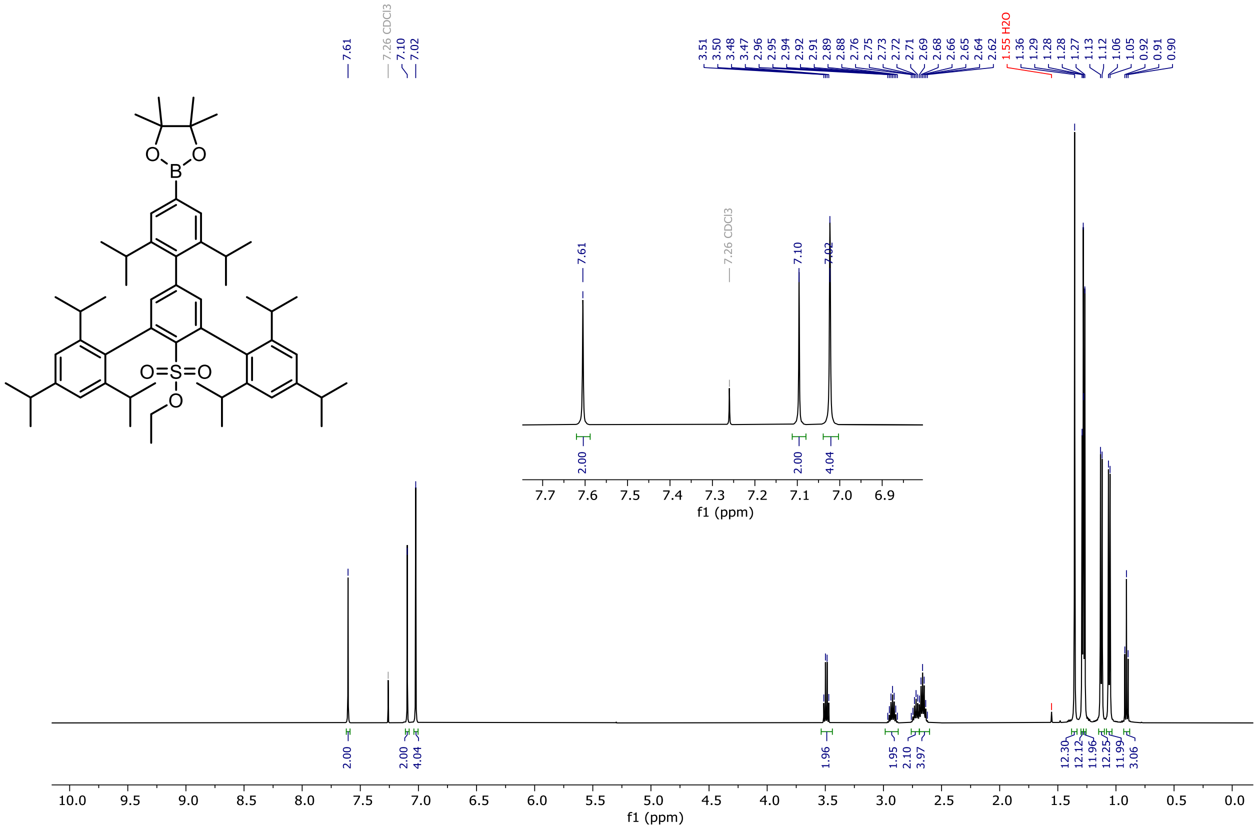

^{13}C NMR (126 MHz, $CDCl_3$) of **Bpin_A4_SO₃Et**

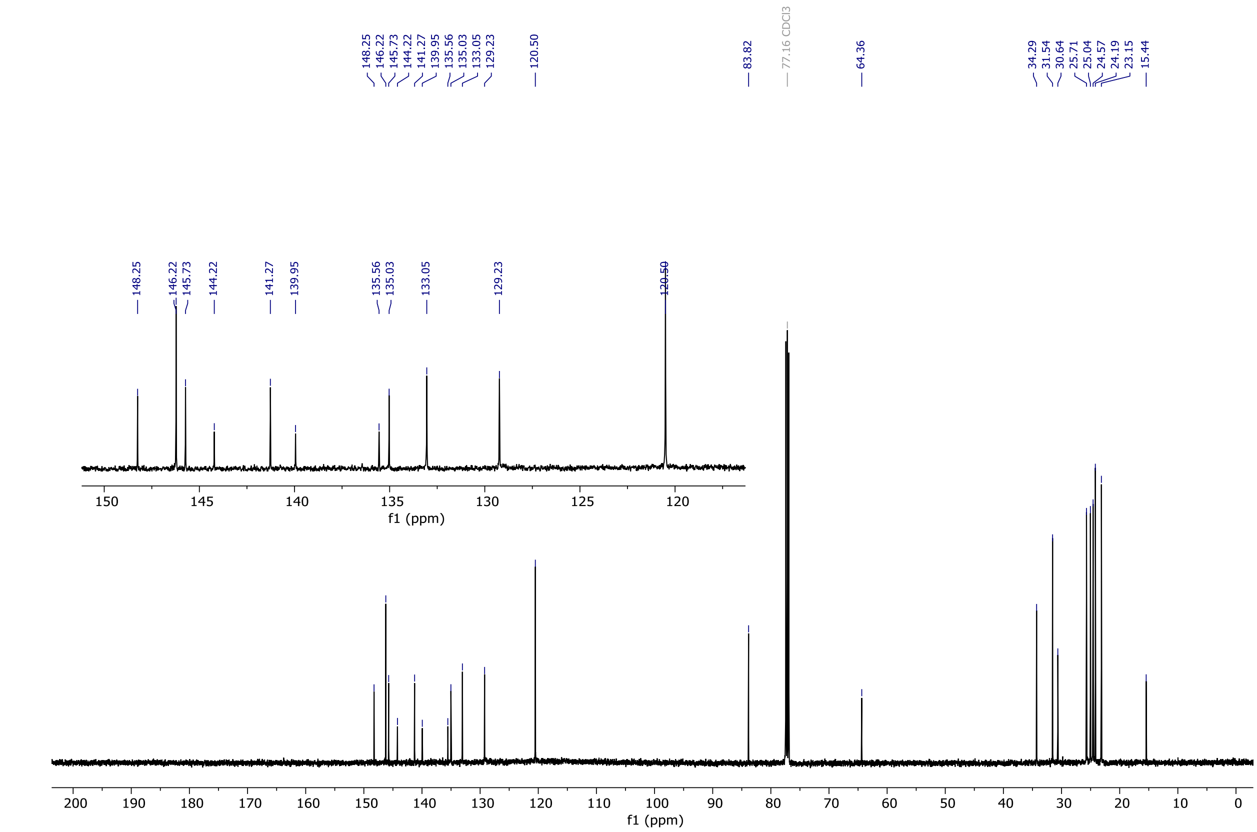

^{1}H NMR (500 MHz, C_6D_6) of **A8_SO$_3$Et**

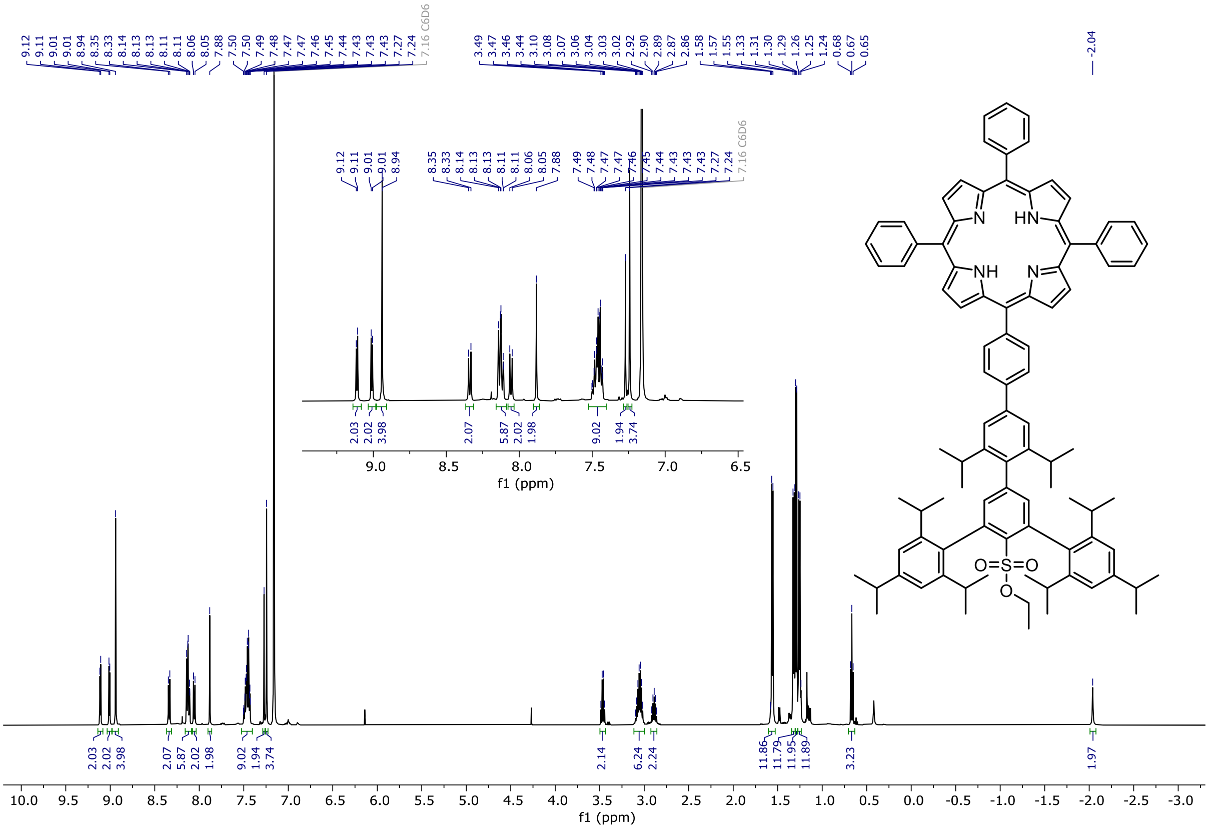

^{13}C NMR (126 MHz, C_6D_6) of **A8_SO$_3$Et**

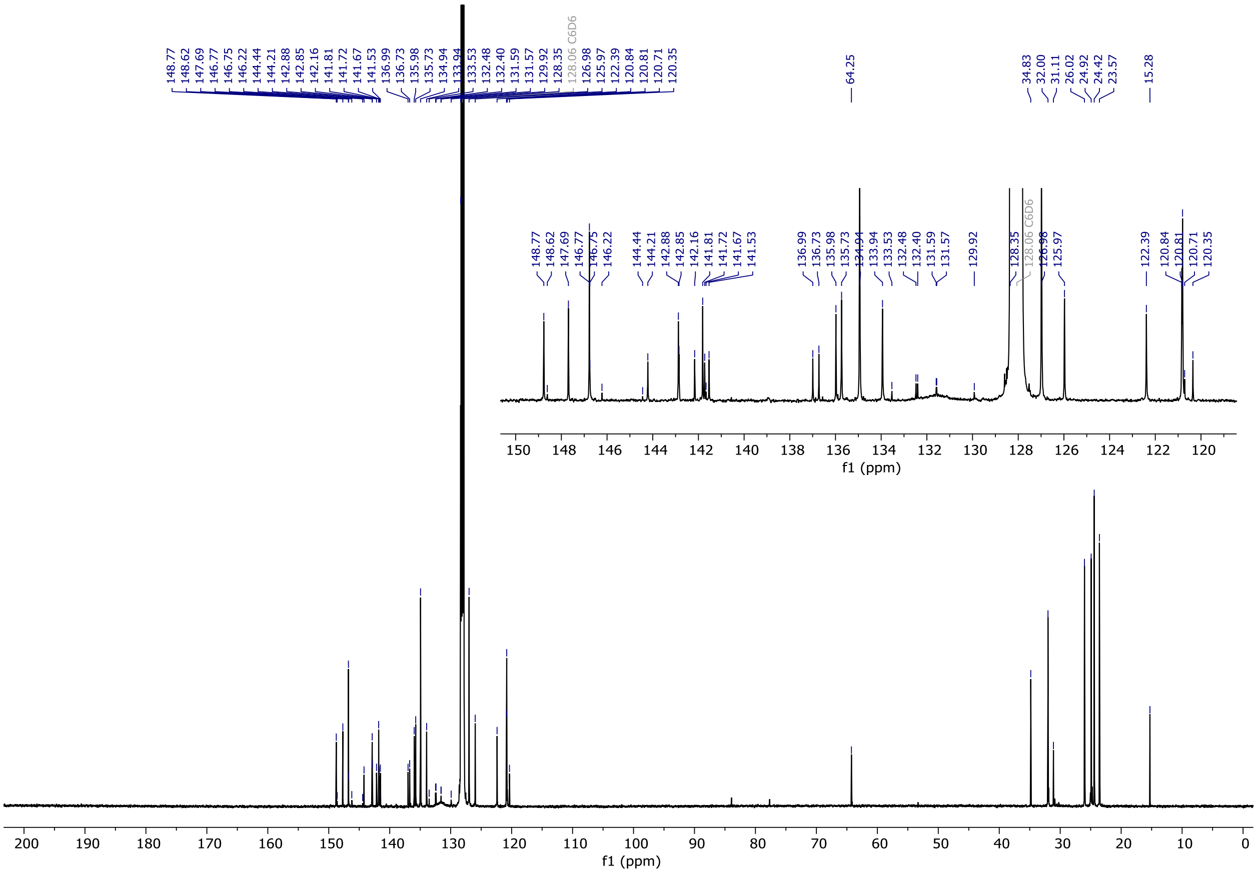

^{1}H NMR (500 MHz, $CDCl_3$) of **Na$^+$A8$^-$**

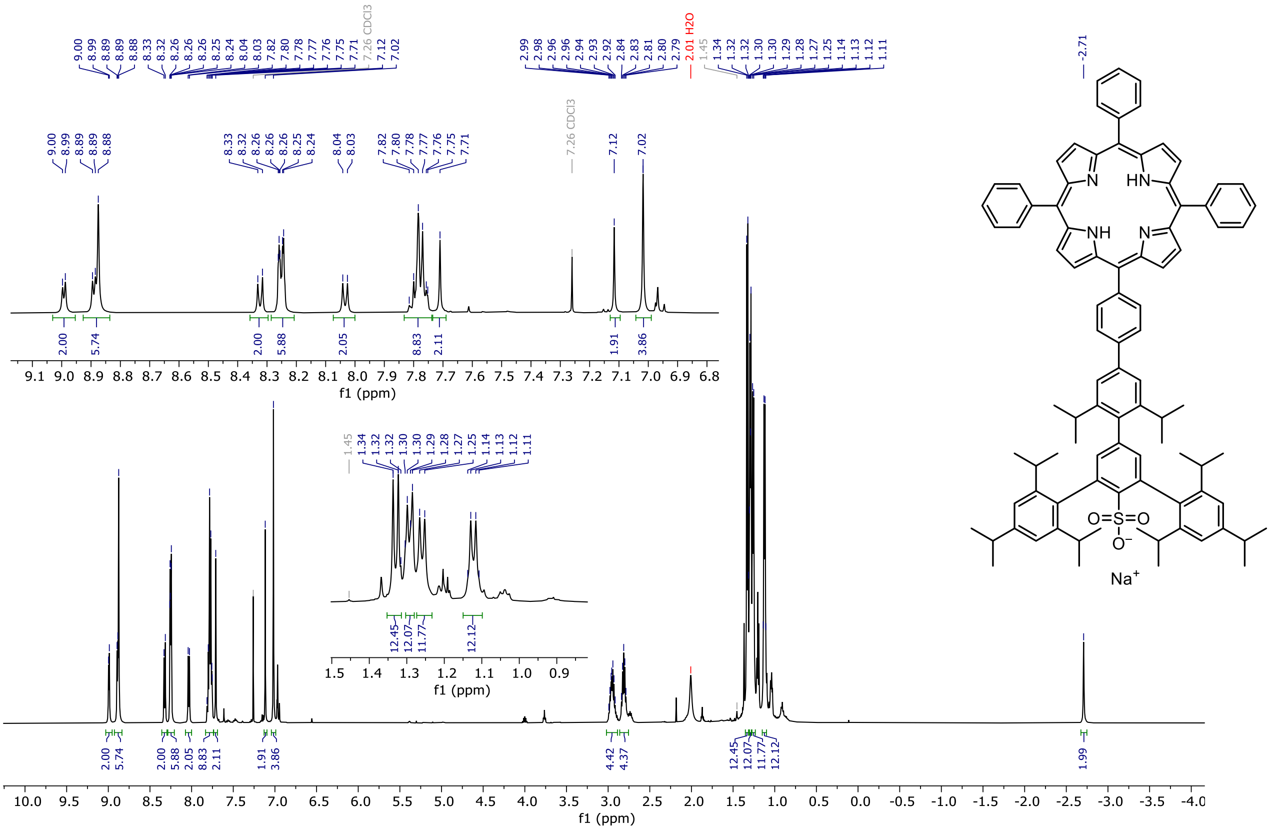

^{13}C NMR (126 MHz, $CDCl_3$) of **Na$^+$A8$^-$**

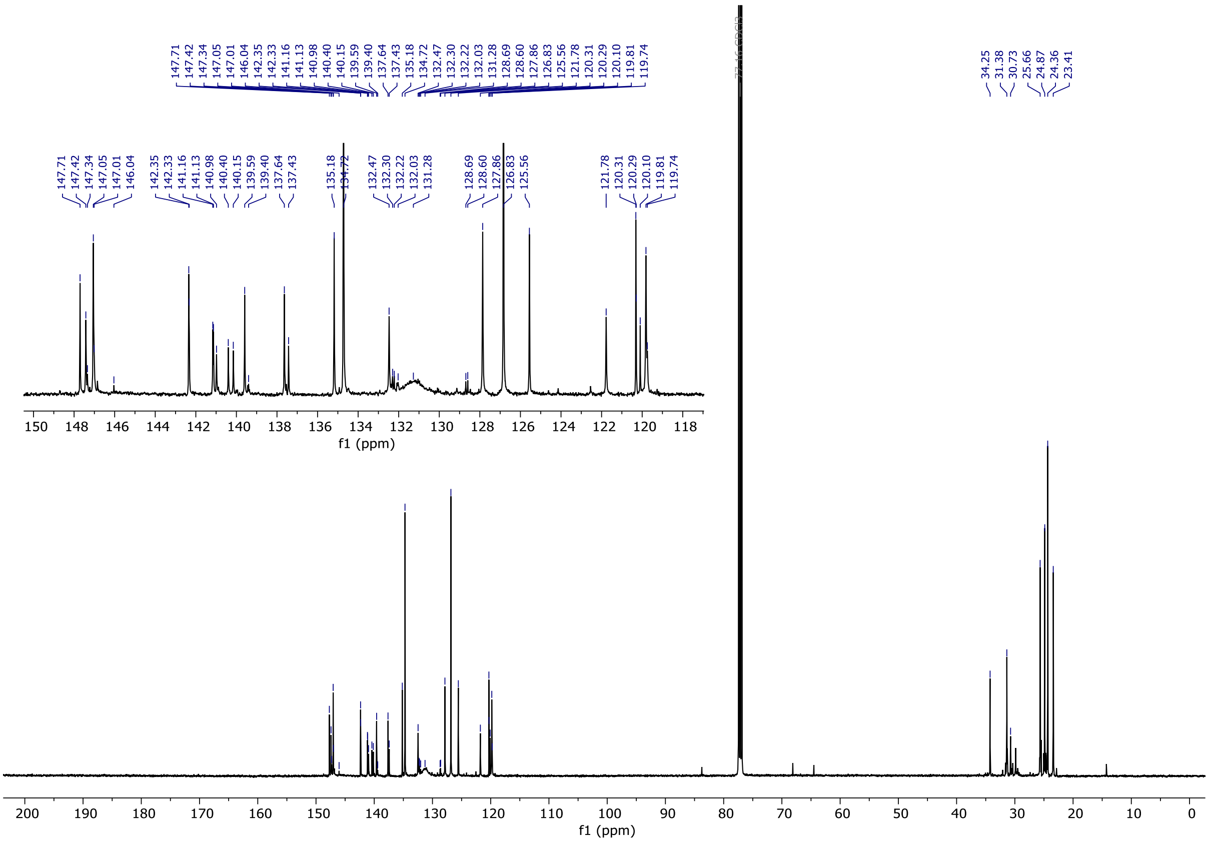

^1H NMR (500 MHz, $CDCl_3$) of **Na⁺A9⁻**

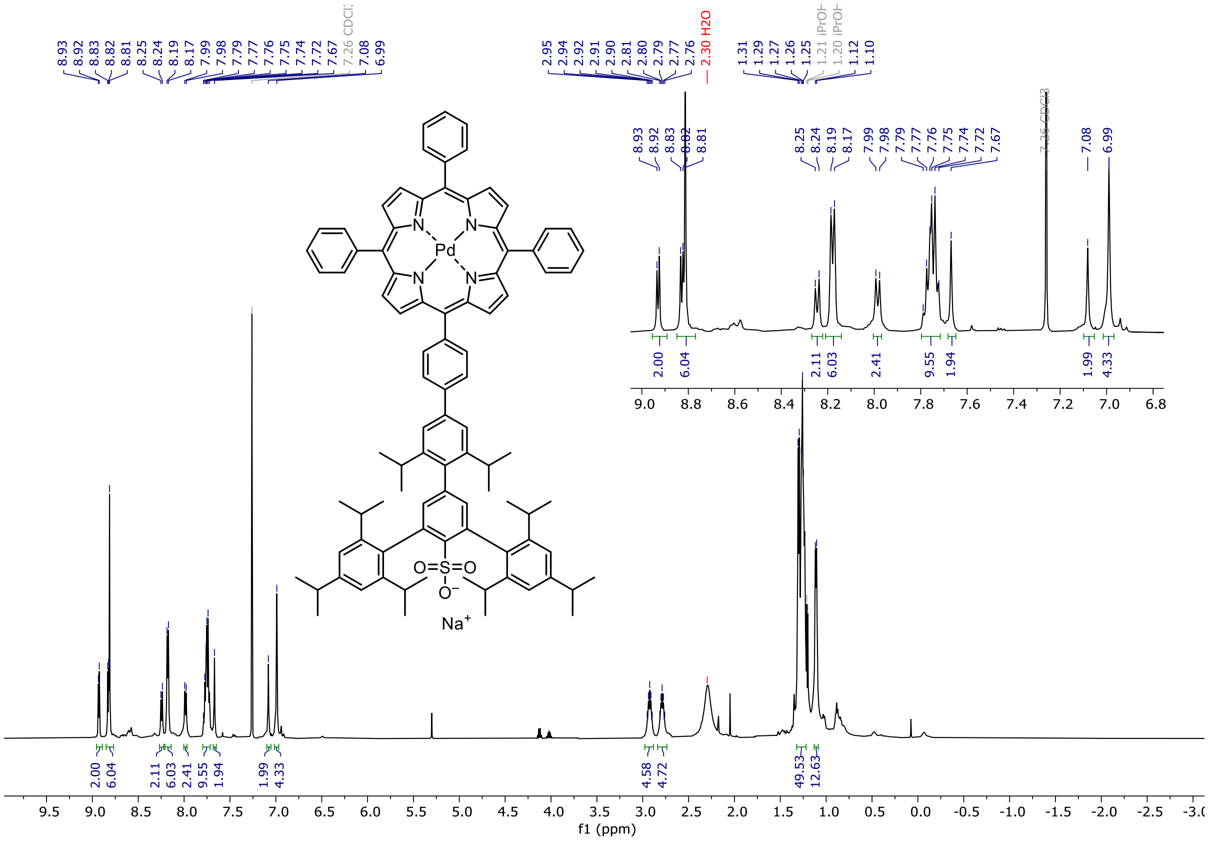

^{13}C NMR (126 MHz, $CDCl_3$) of **Na⁺A9⁻**

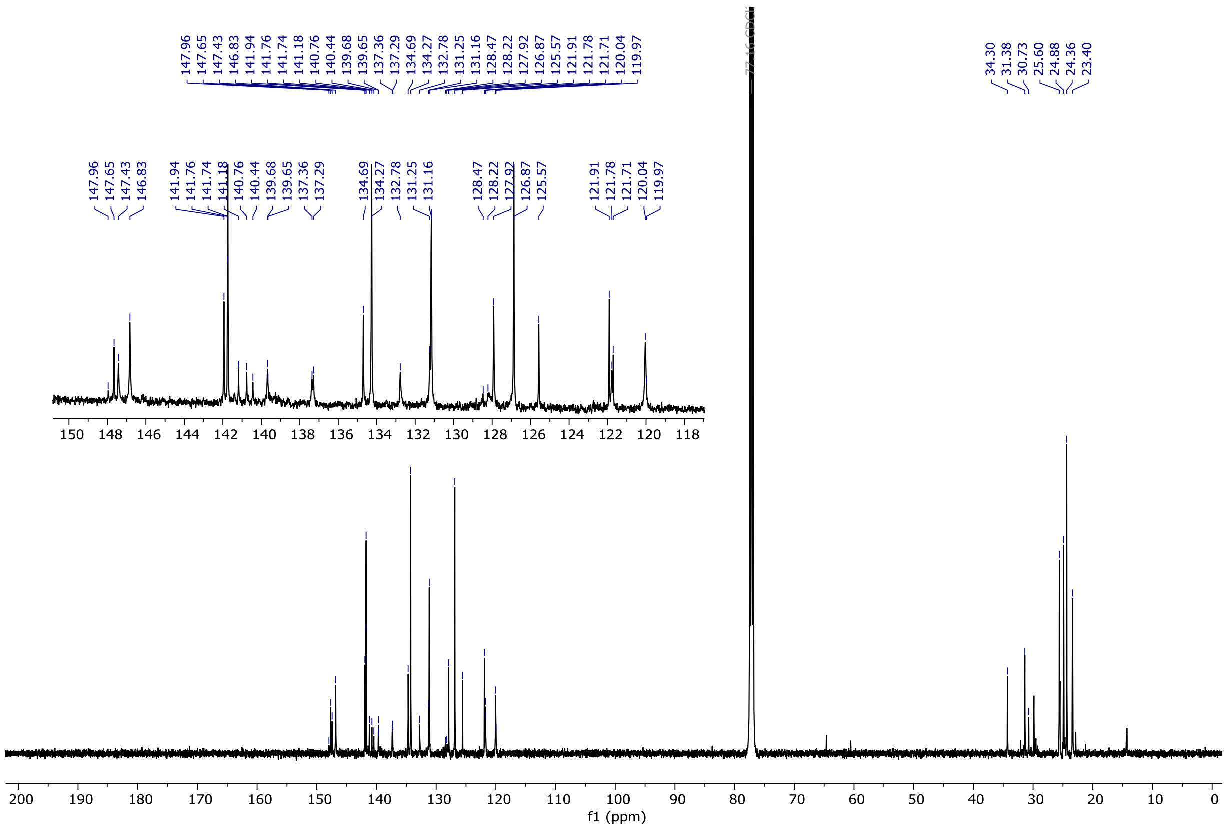

^{1}H NMR (500 MHz, CDCl$_3$) of **A10_SO$_3$Et**

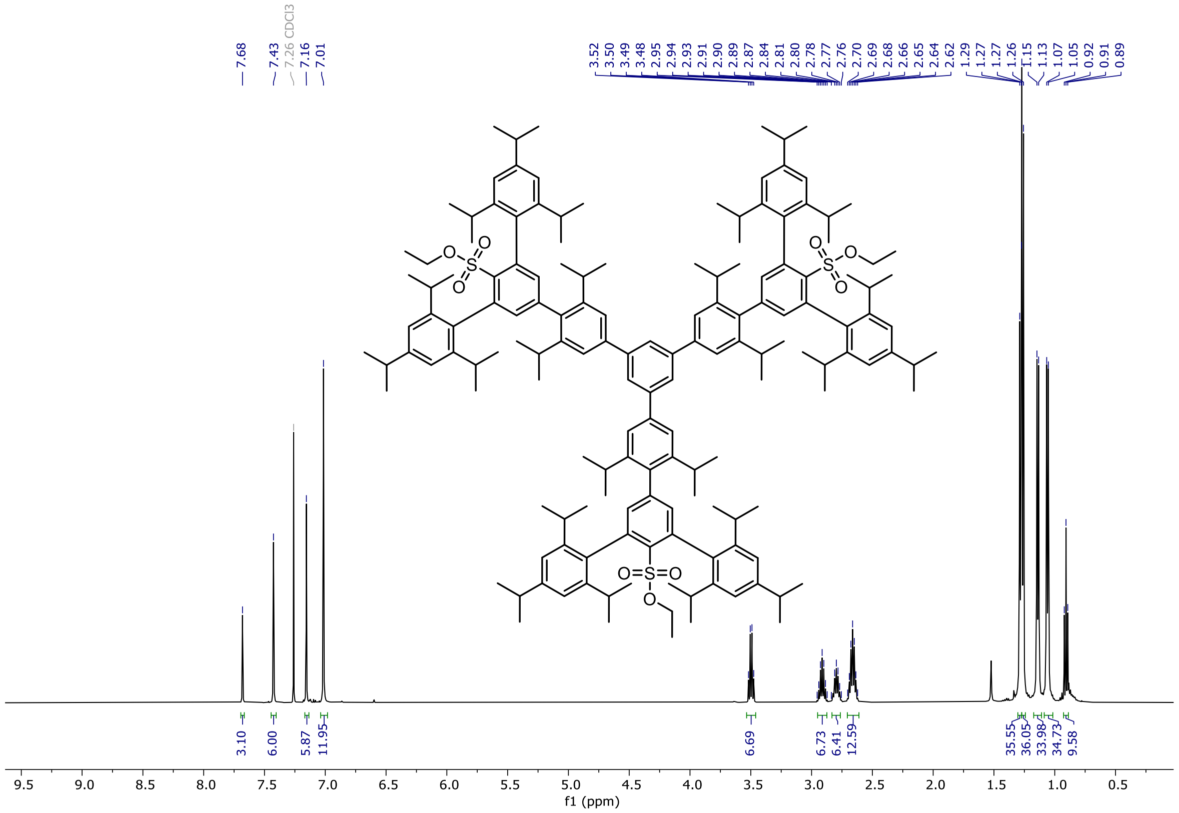

^{13}C NMR (126 MHz, CDCl$_3$) of **A10_SO$_3$Et**

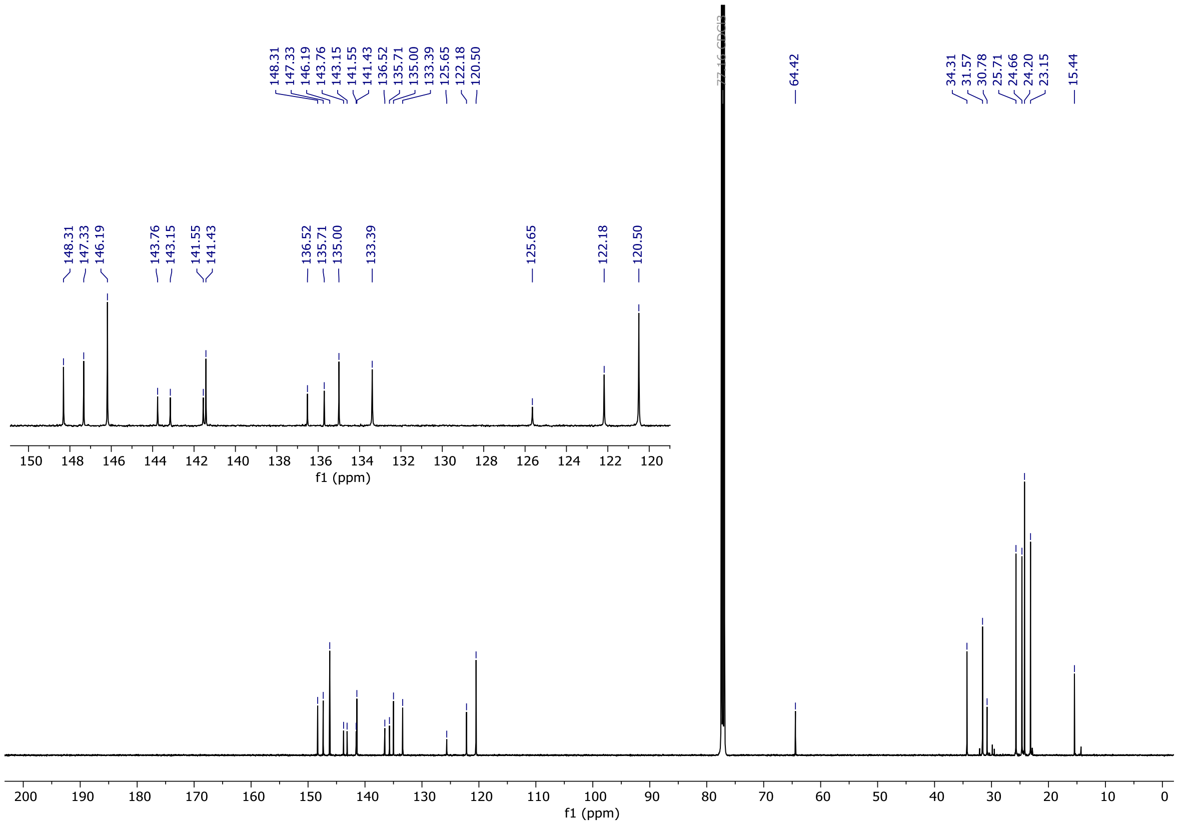

^{1}H NMR (400 MHz, $CDCl_3$-CD_3OD) of **$(Na^+)_3$A10^{3-}**

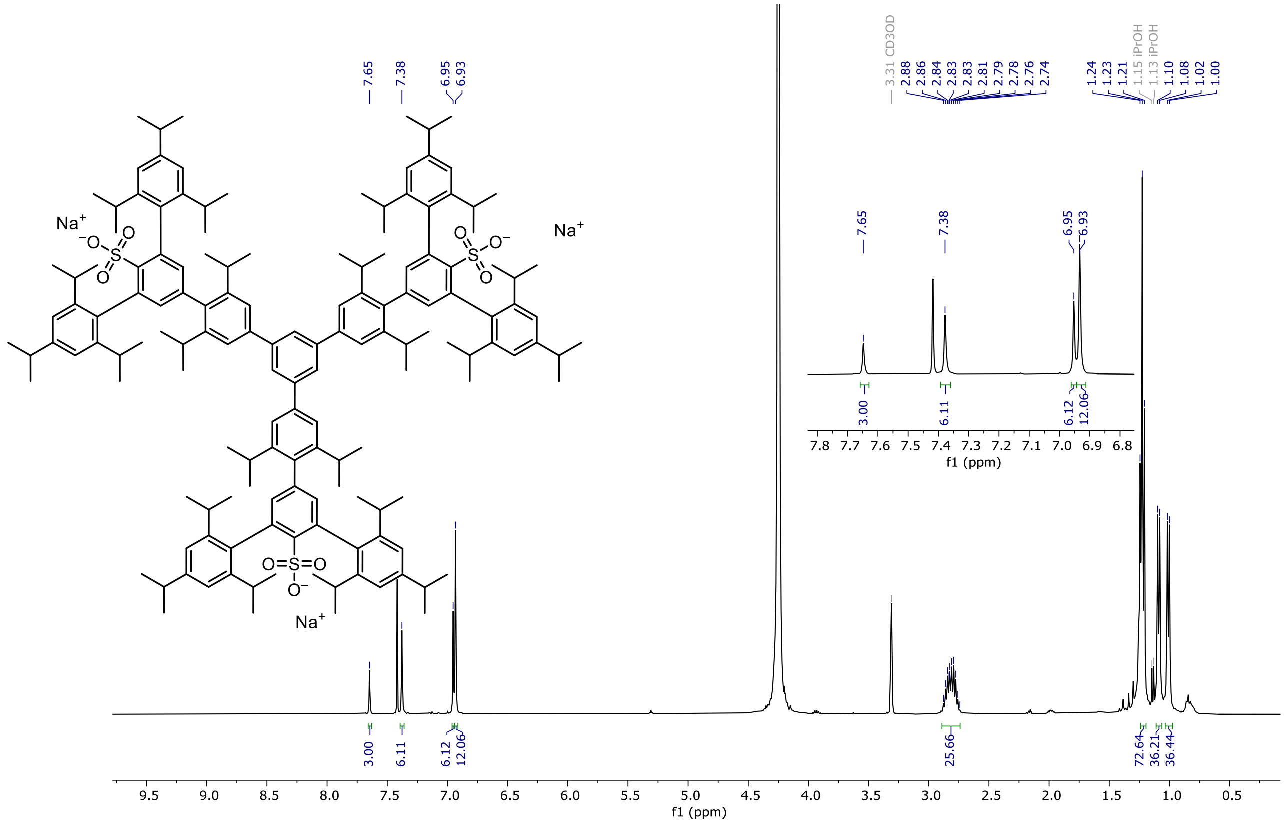

^{13}C NMR (101 MHz, $CDCl_3$-CD_3OD) of **$(Na^+)_3$A10^{3-}**

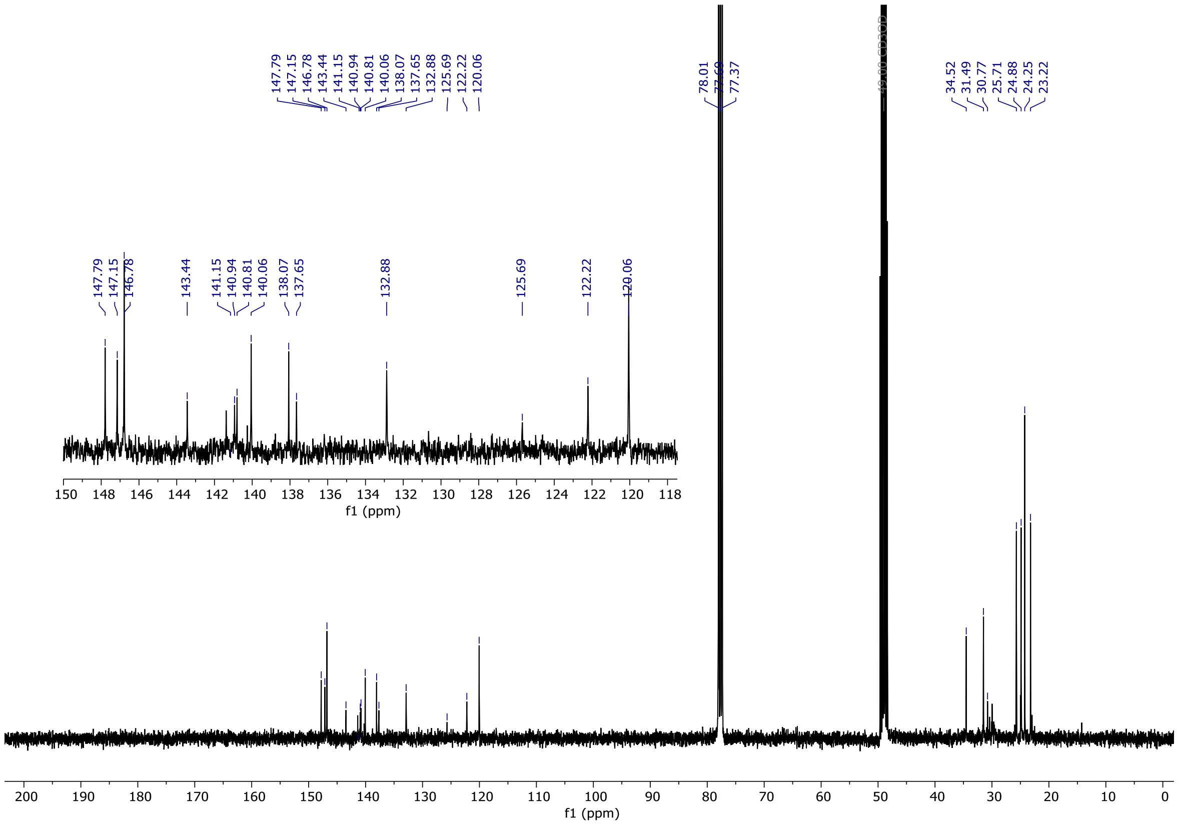

^{1}H NMR (500 MHz, C_6D_6) of **C2^{+}A4^{-}**

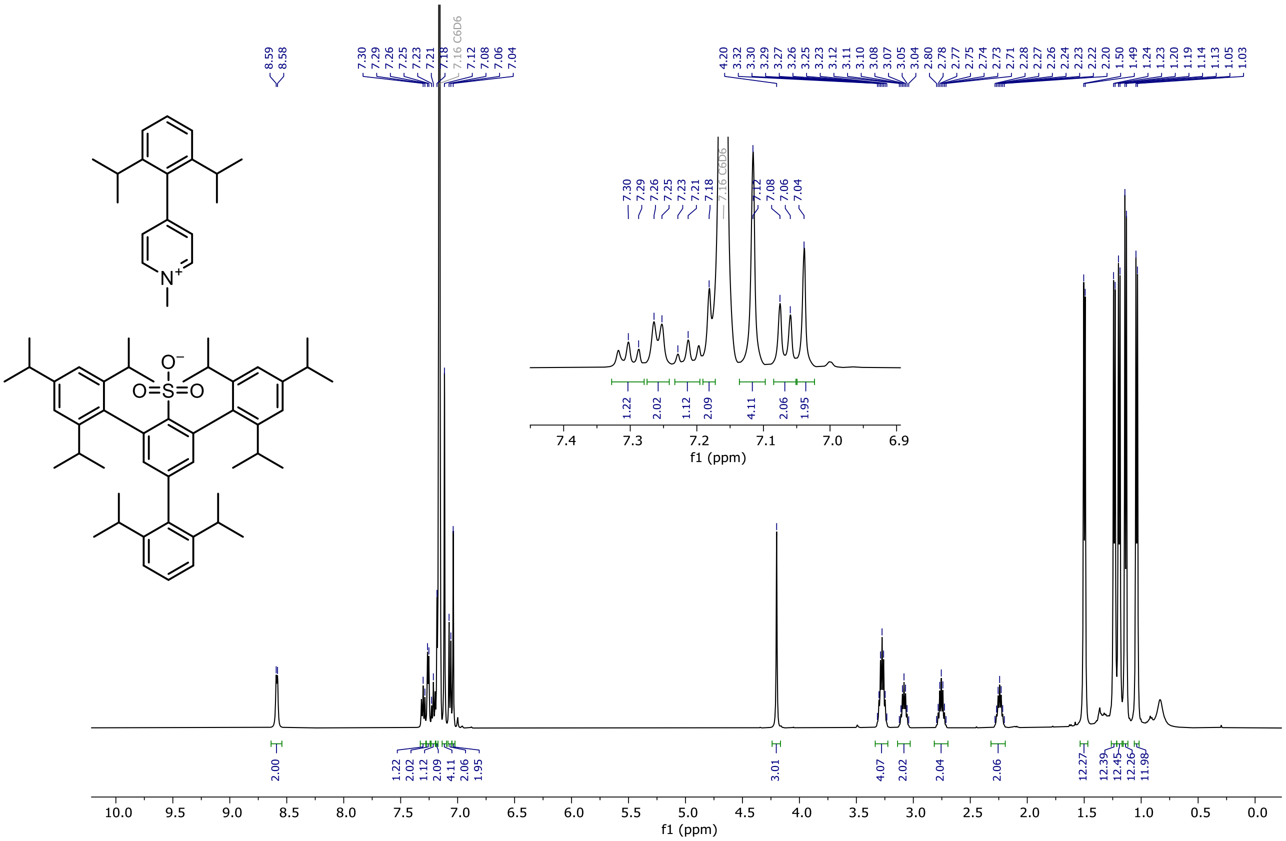

^{13}C NMR (126 MHz, C_6D_6) of **C2^{+}A4^{-}**

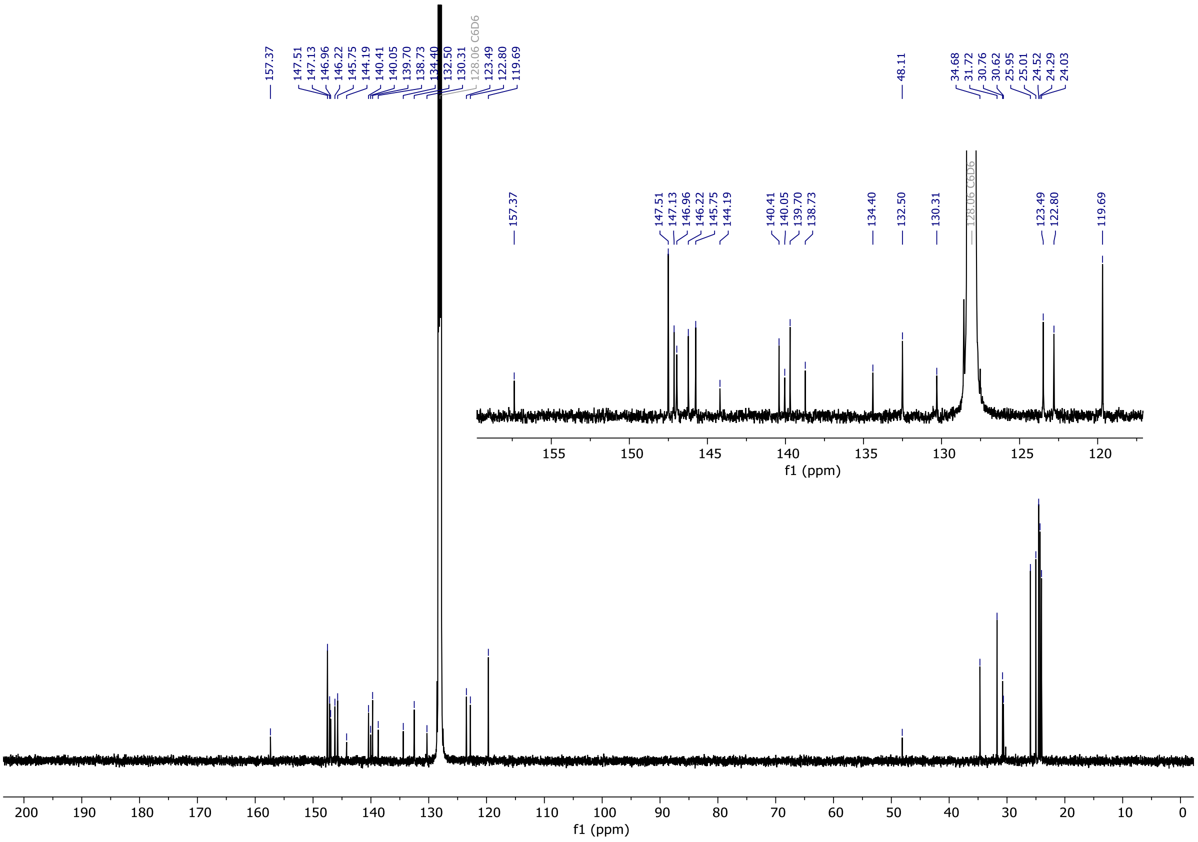

^{1}H NMR (500 MHz, C_6D_6/$CDCl_3$ 9:1) of **C4$^+$A2$^-$**

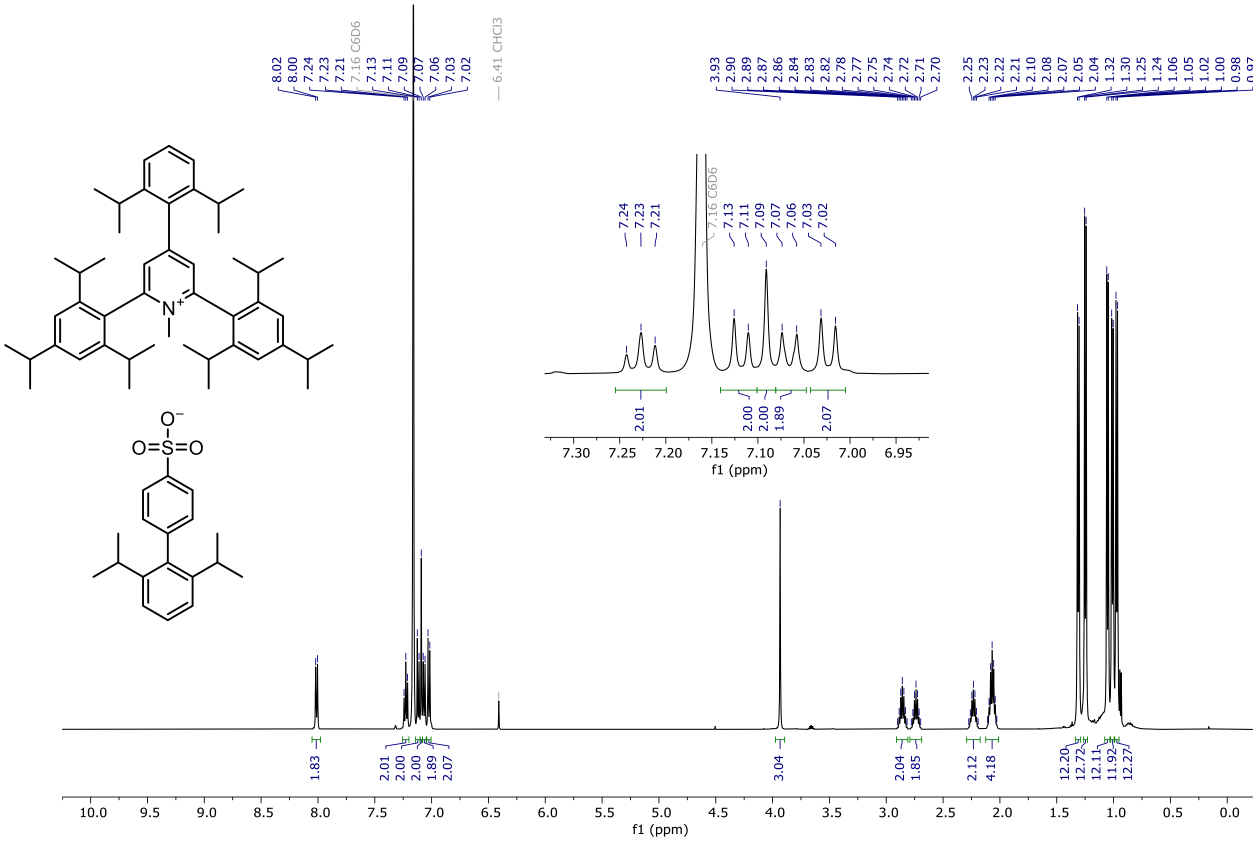

^{13}C NMR (101 MHz, $CDCl_3$) of **C4$^+$A2$^-$**

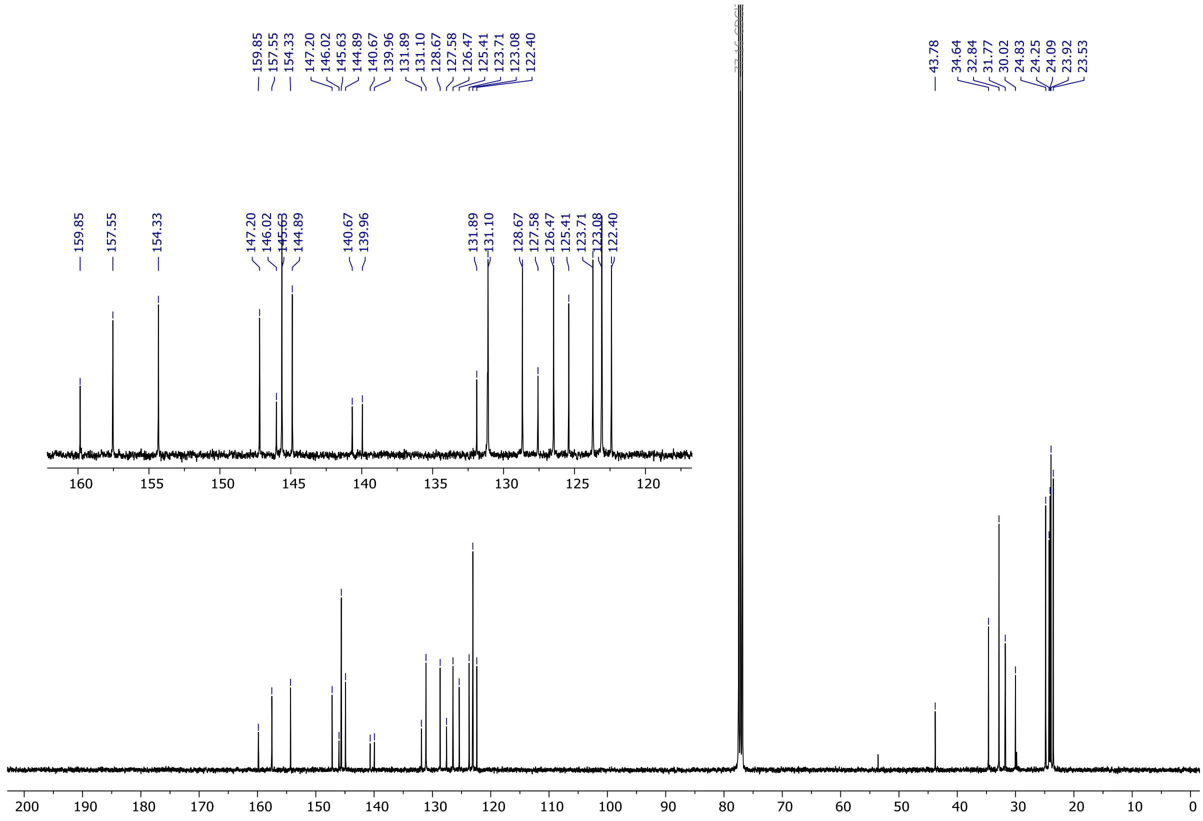

^{1}H NMR (500 MHz, C_6D_6) of **C3^{+}A3^{-}**

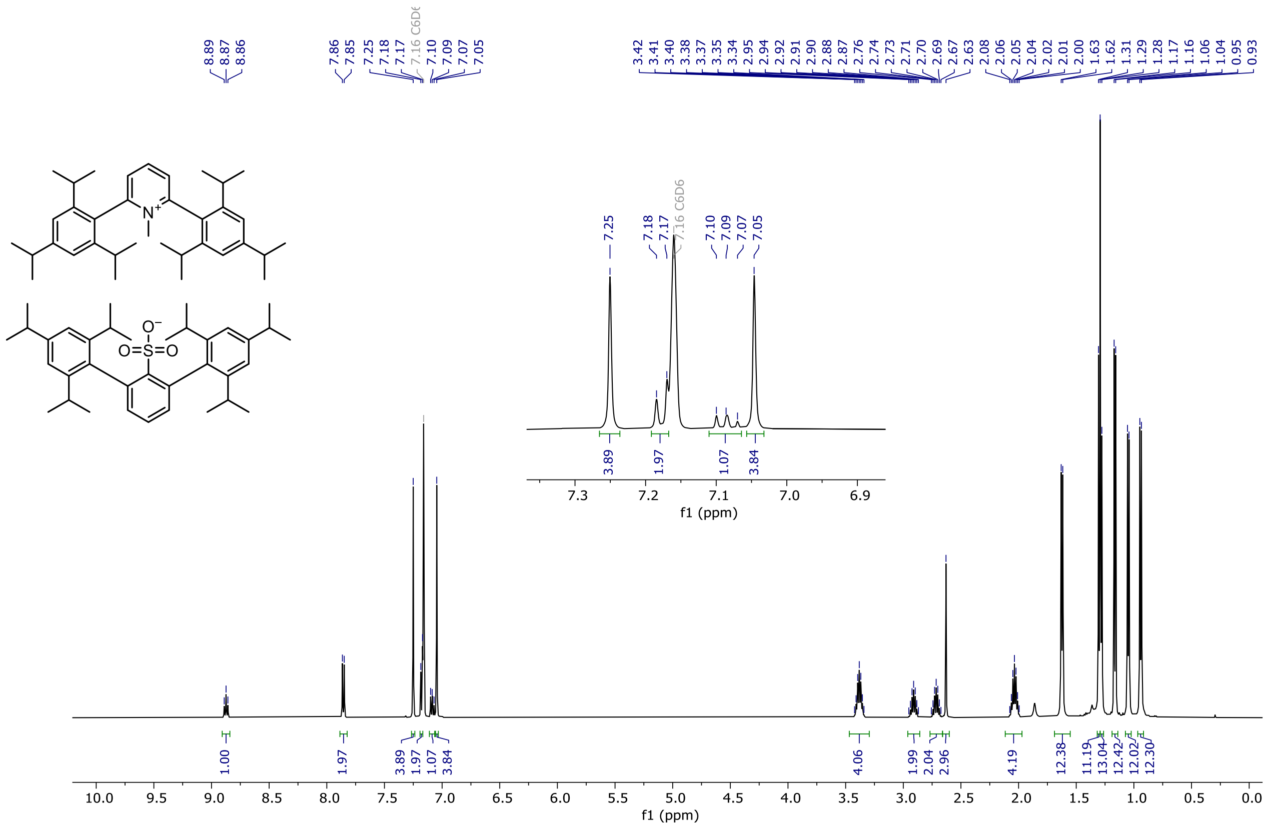

^{13}C NMR (126 MHz, C_6D_6) of **C3^{+}A3^{-}**

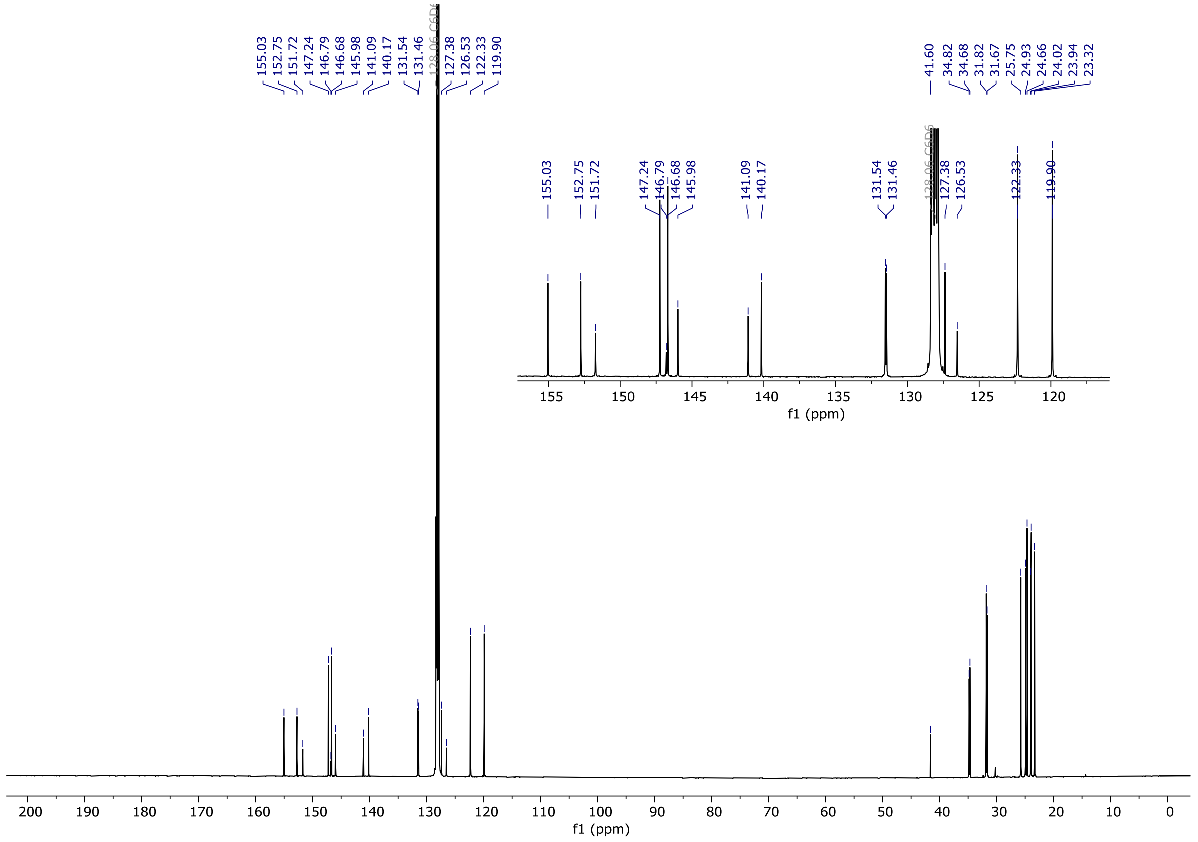

COSY (C_6D_6) of **C3⁺A3⁻**

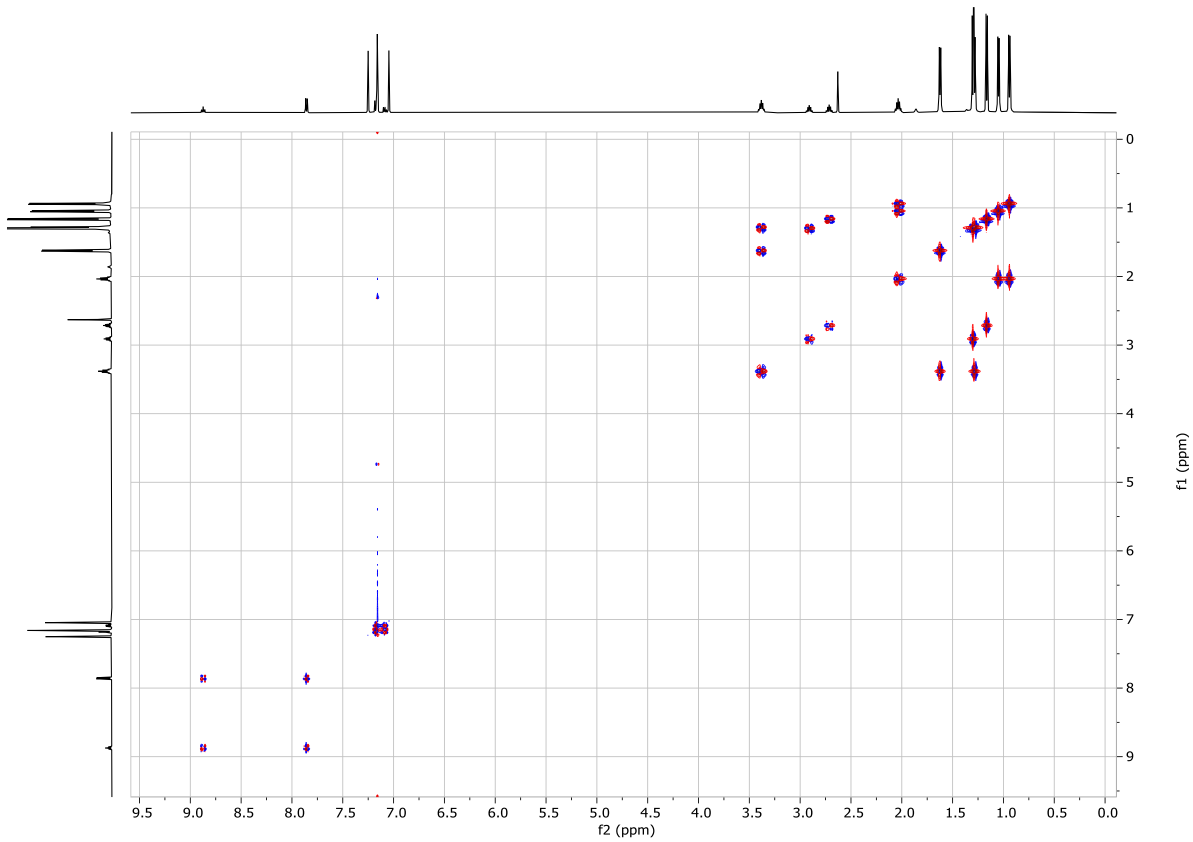

Excerpt from COSY of **C3⁺A3⁻**

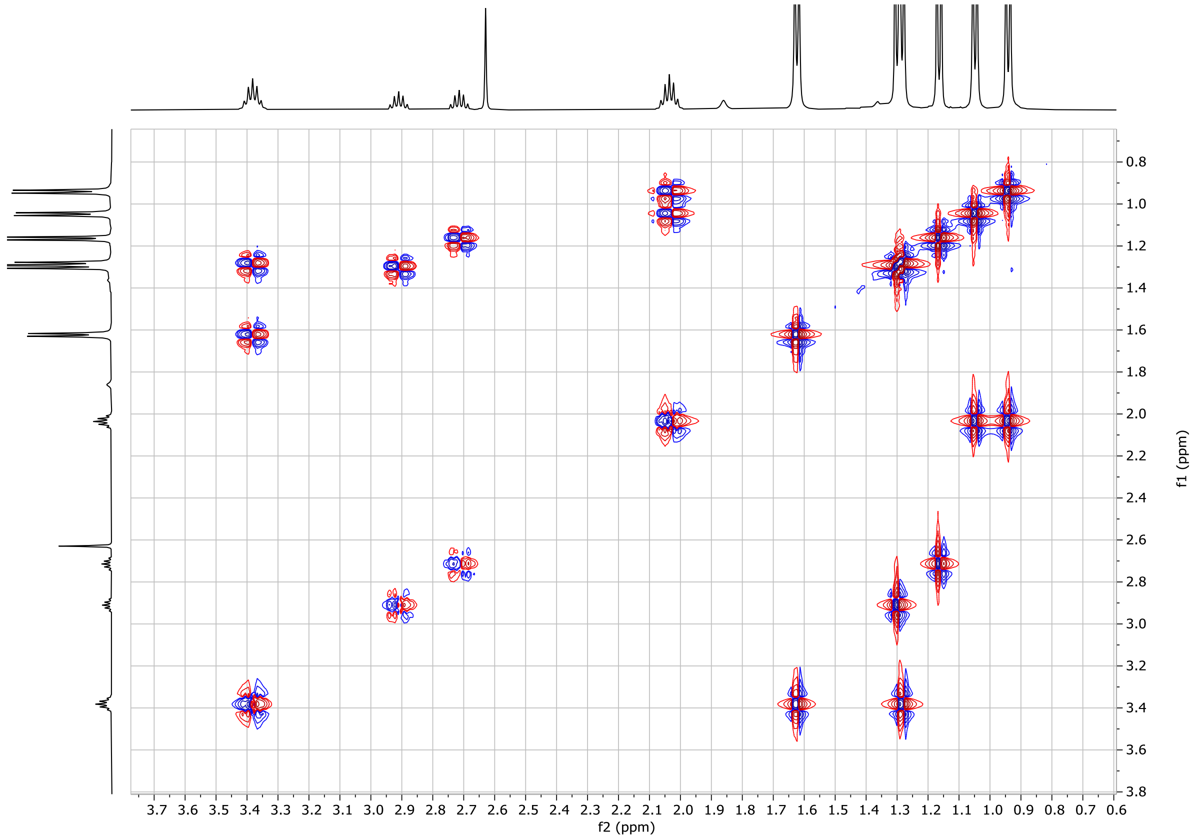

HSQC (C_6D_6) of **C3⁺A3⁻**

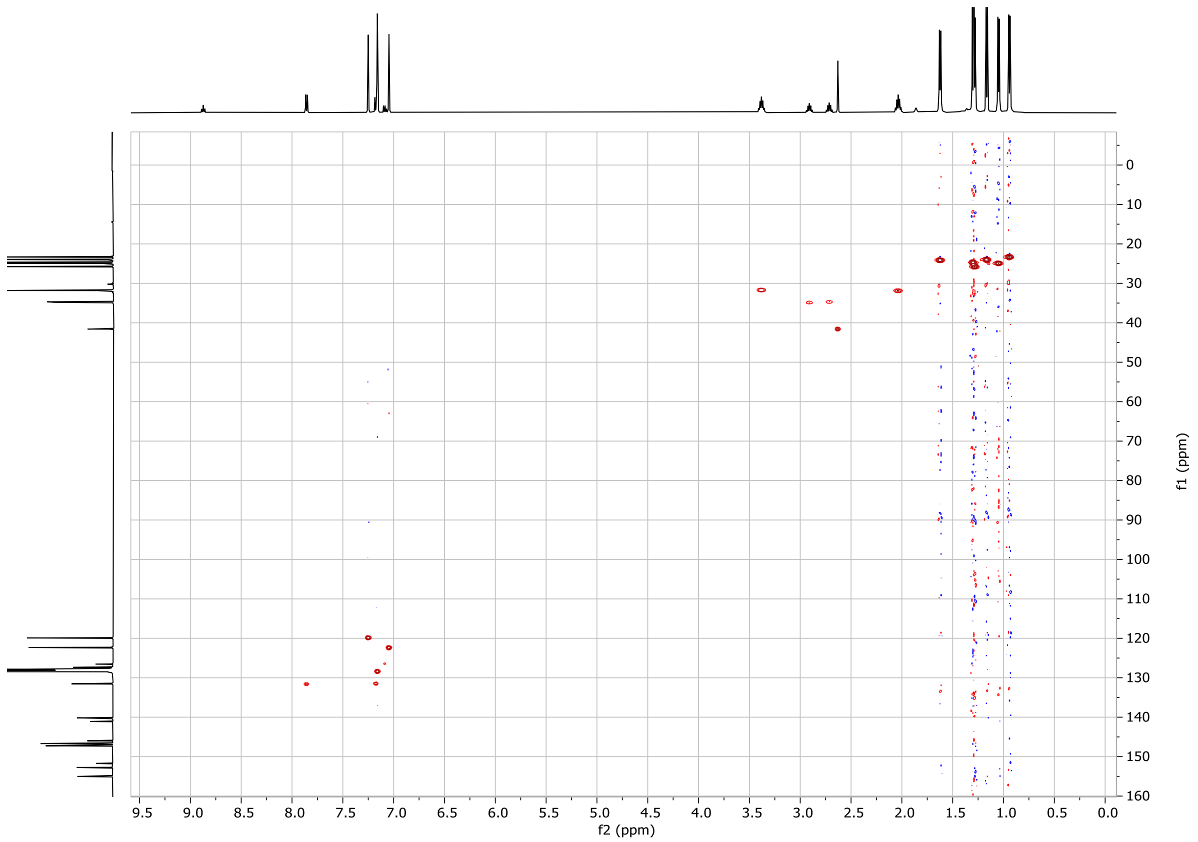

Excerpt from HSQC of **C3⁺A3⁻**

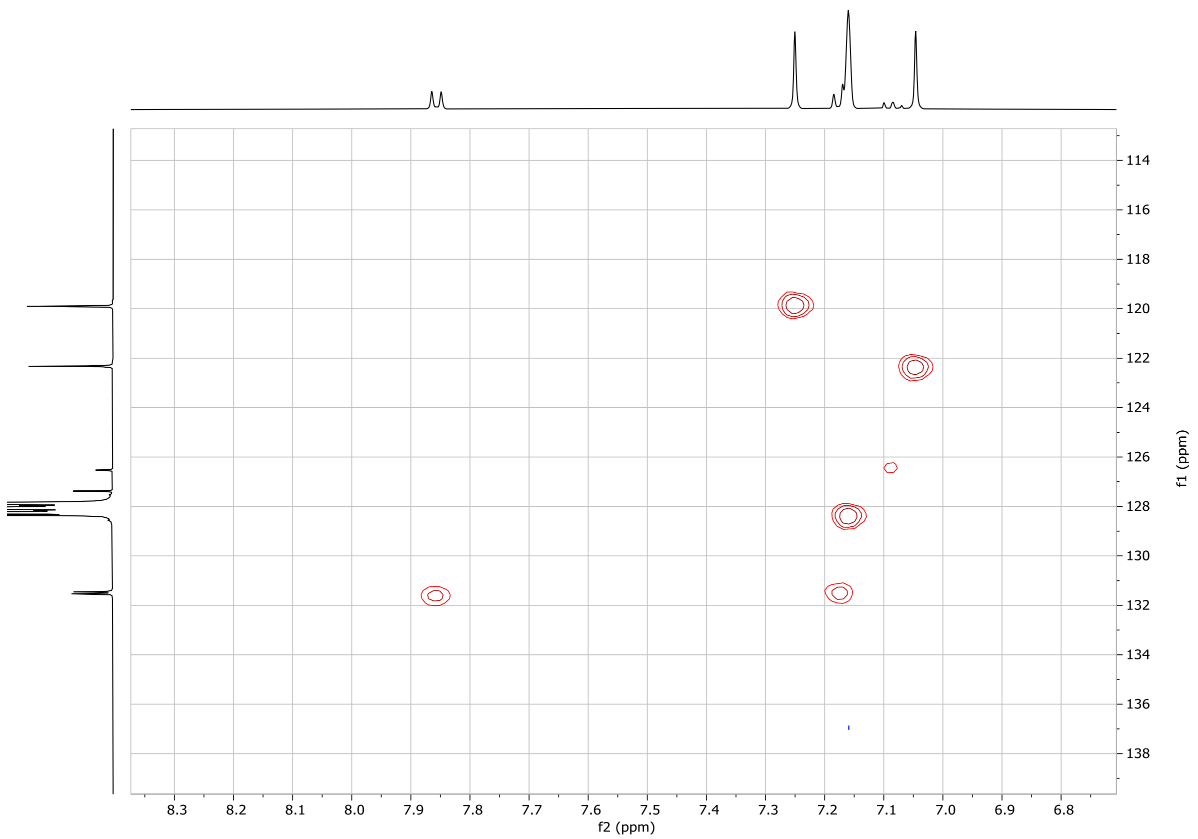

Excerpt from HSQC of **C3⁺A3⁻**

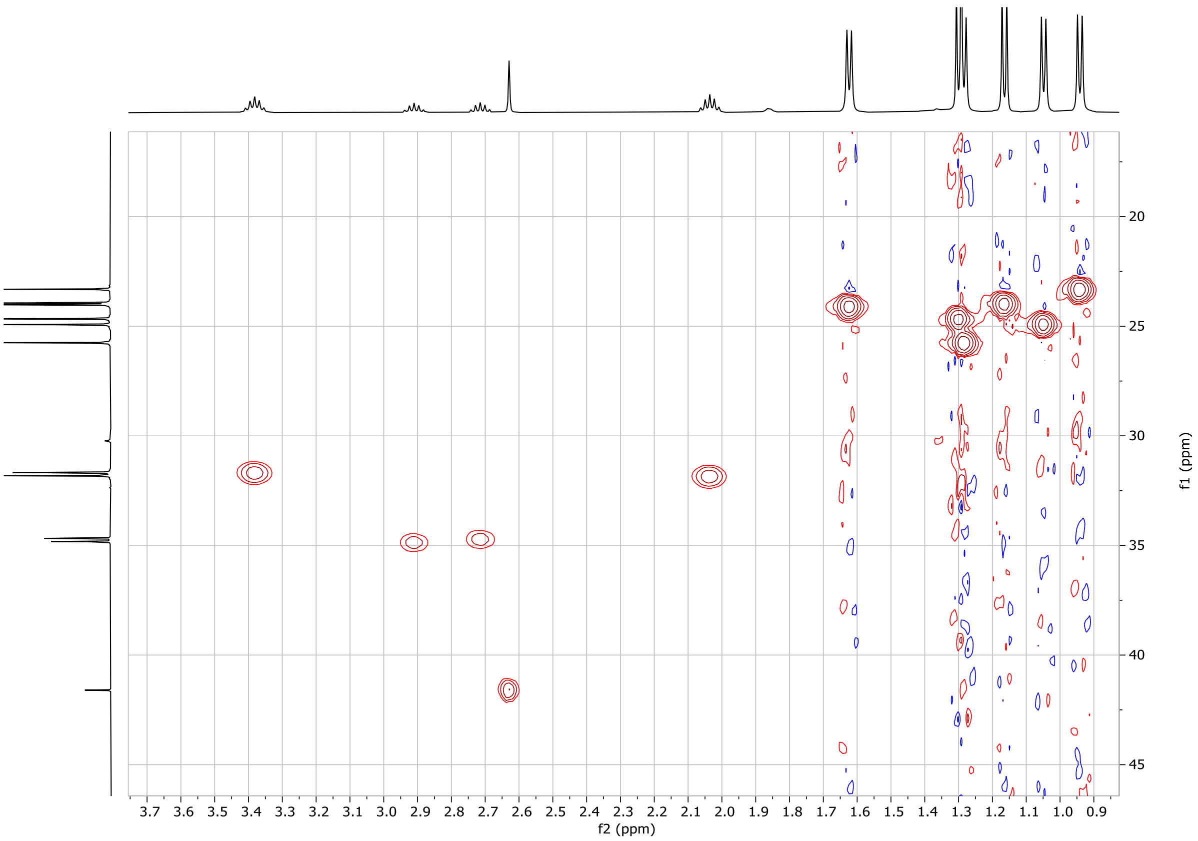

HMBC (C_6D_6) of **C3⁺A3⁻**

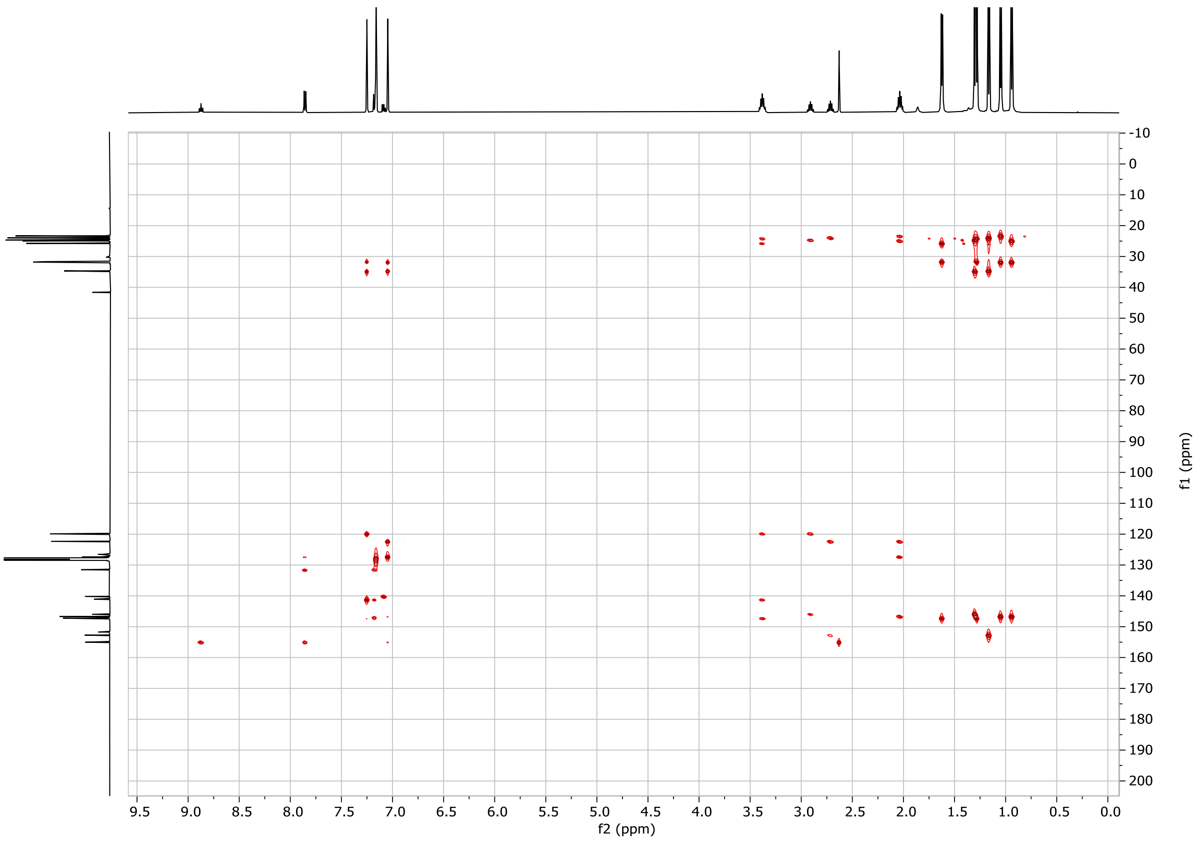

Excerpt from HMBC of **C3⁺A3⁻**

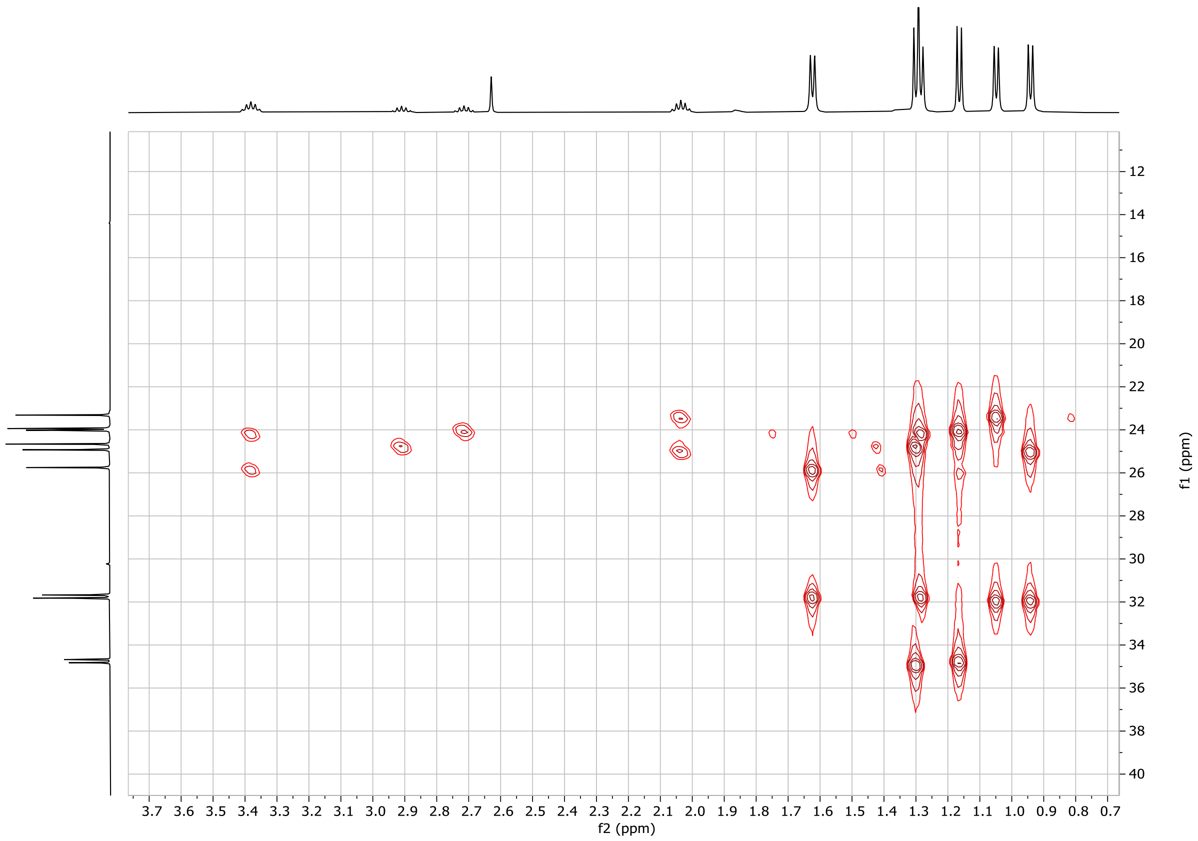

Excerpt from HMBC of **C3⁺A3⁻**

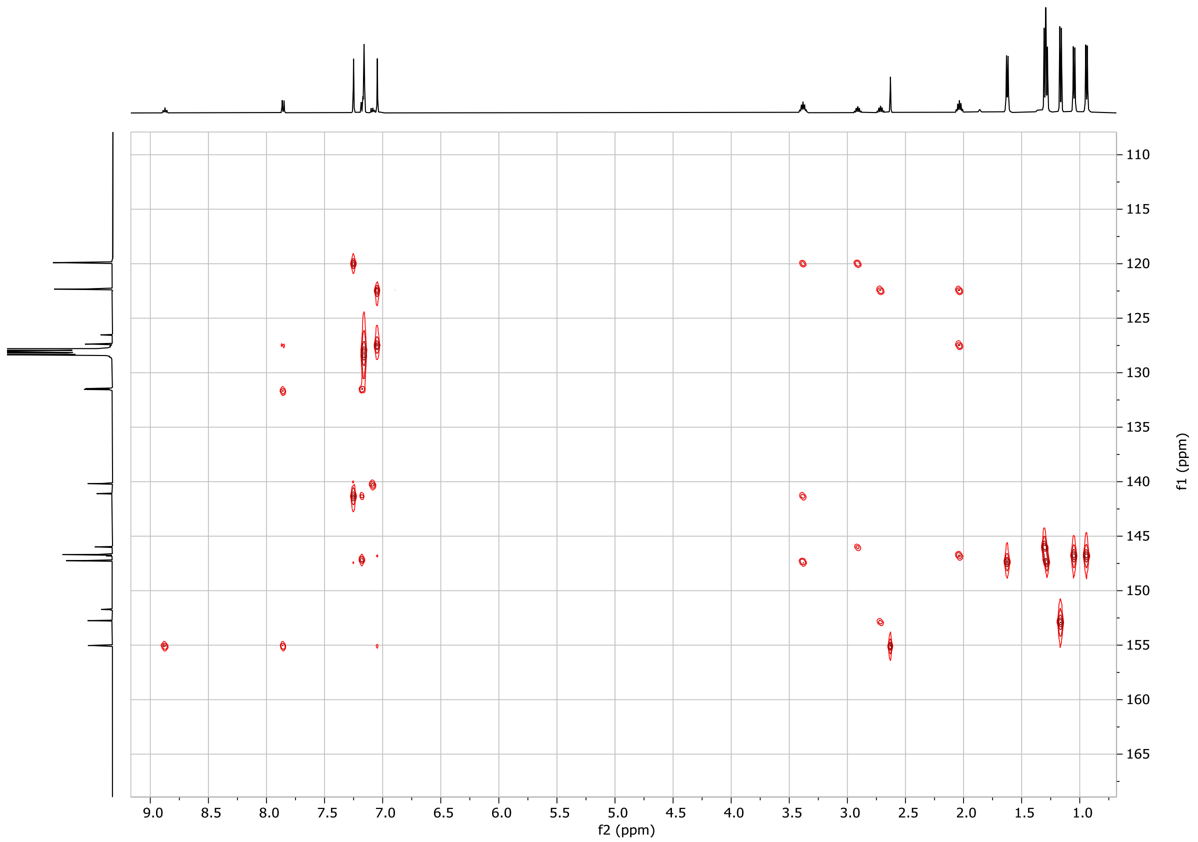

ROESY of **C3**$^{+}$**A3**$^{-}$

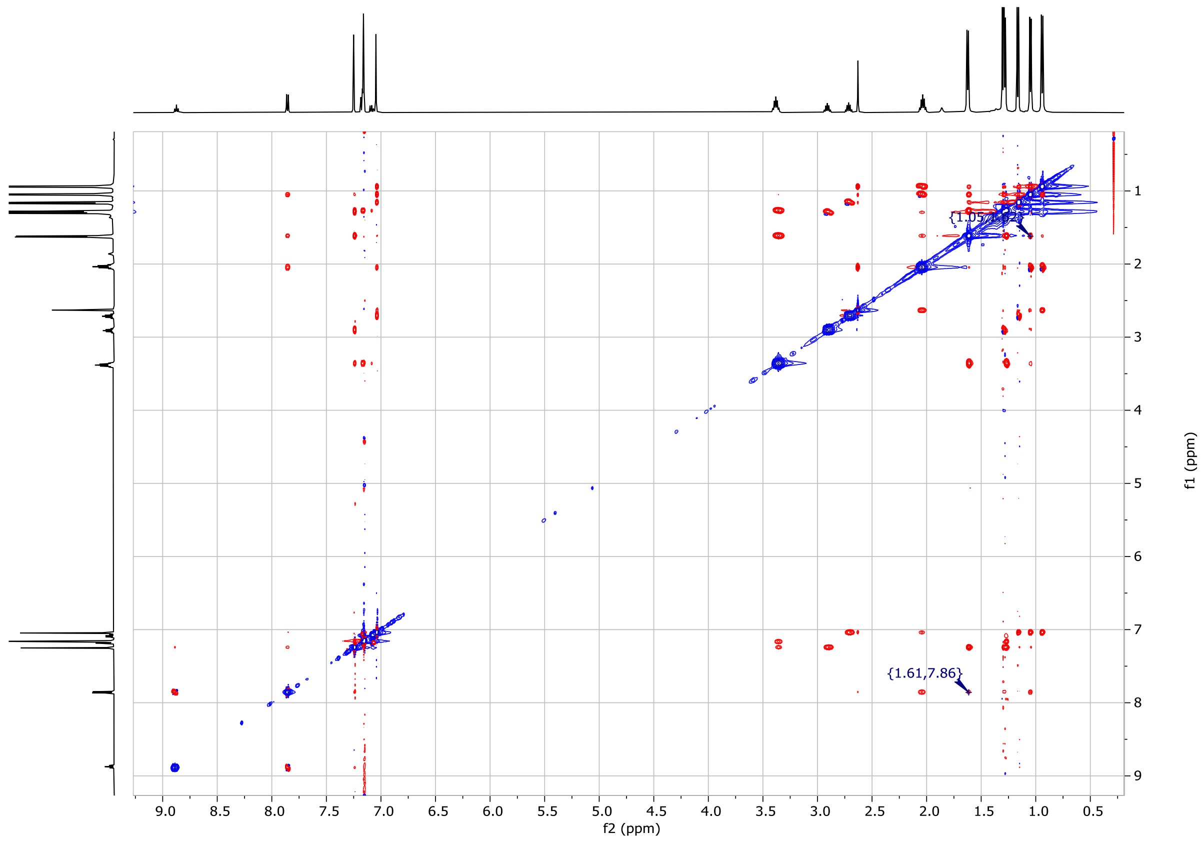

^{1}H NMR (500 MHz, C_6D_6) of **1**

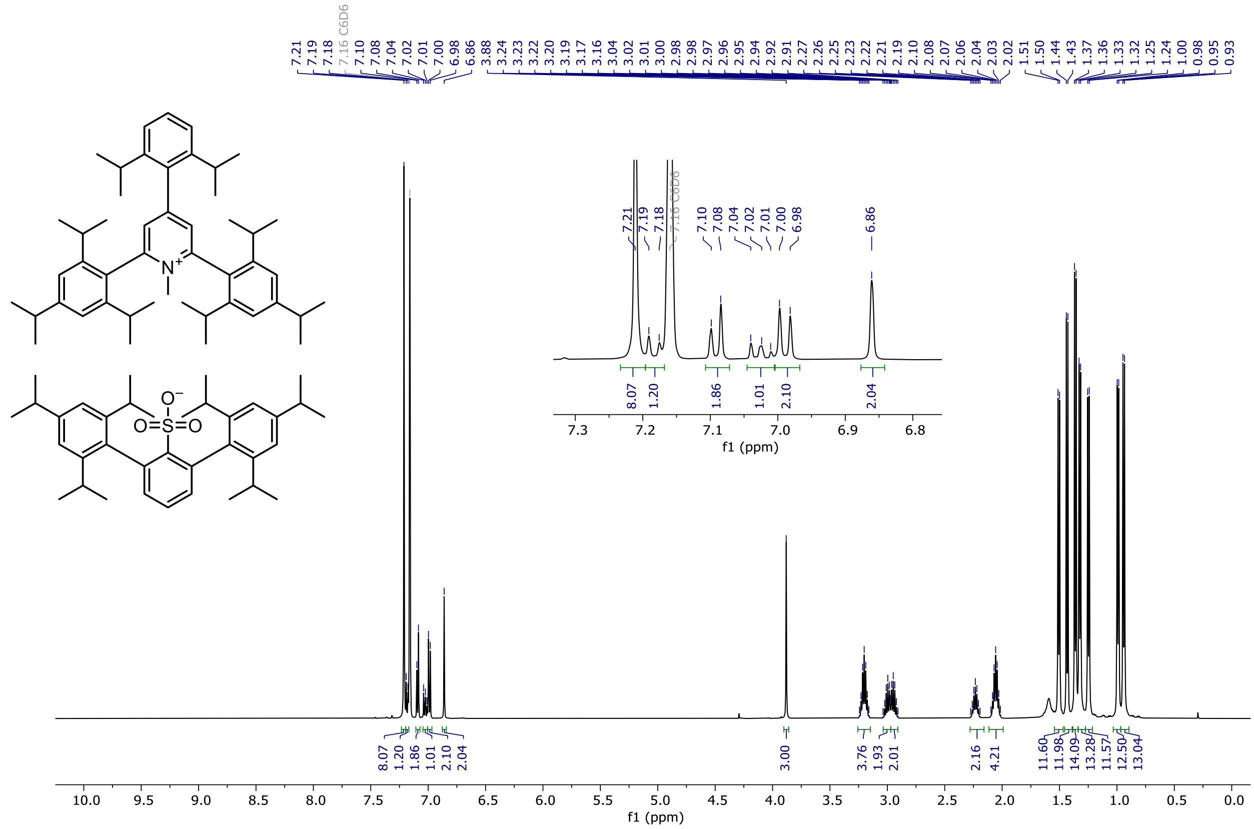

^{13}C NMR (126 MHz, C_6D_6) of **1**

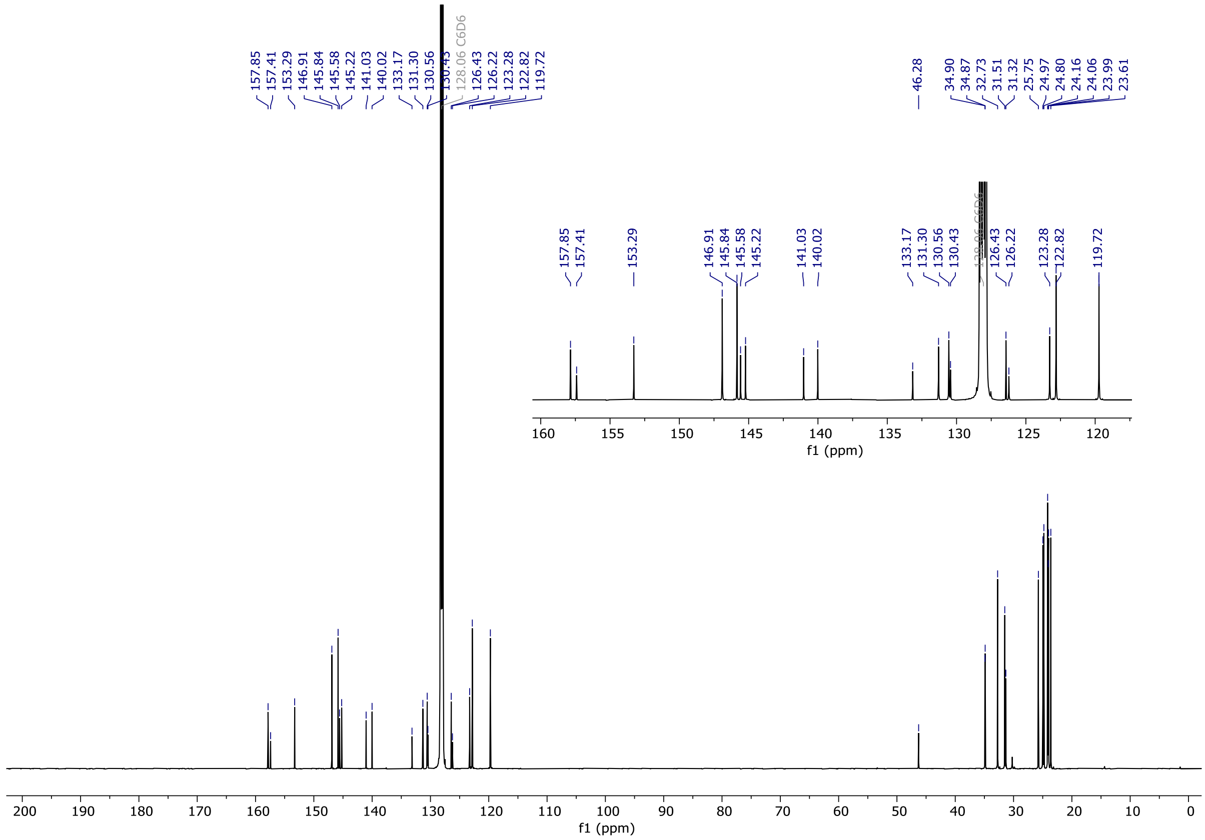

^{1}H NMR (400 MHz, $CDCl_3$) of **2**

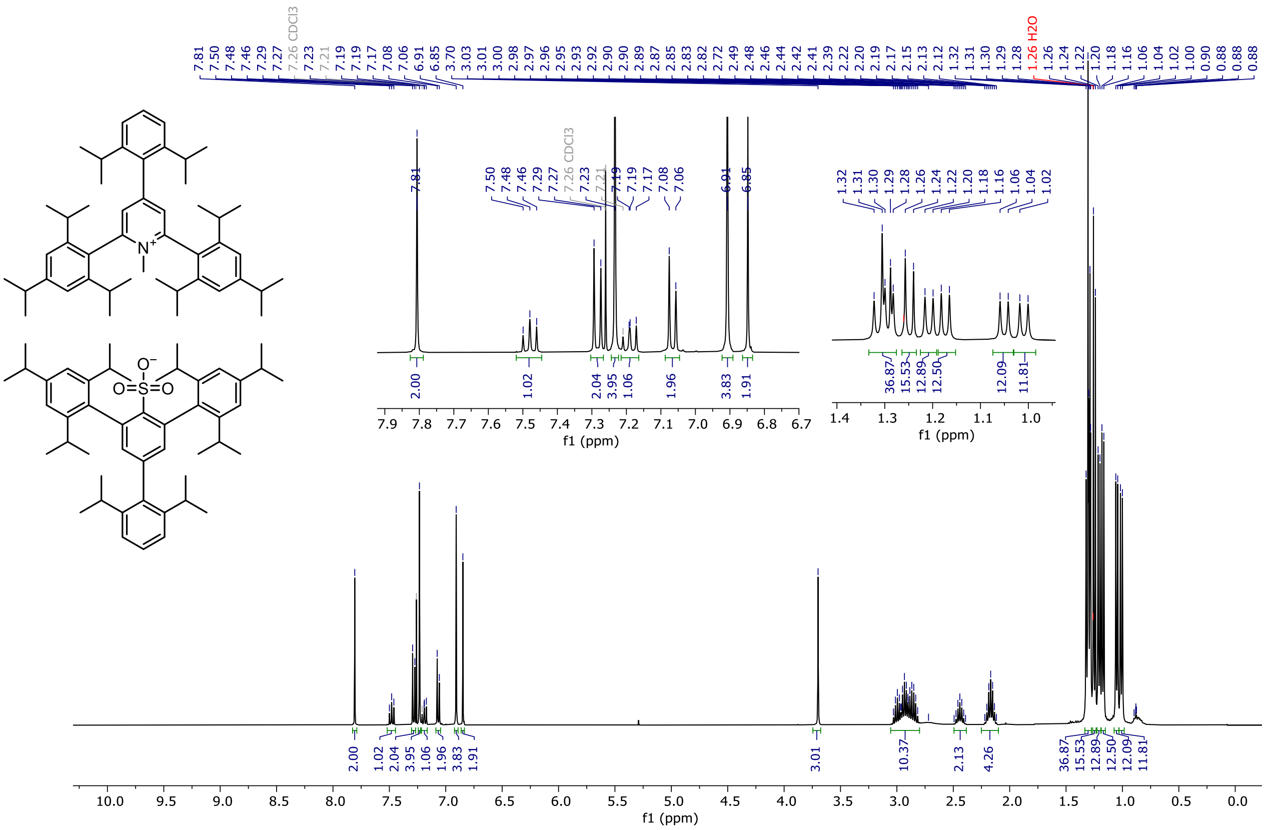

^{13}C NMR (101 MHz, $CDCl_3$) of **2**

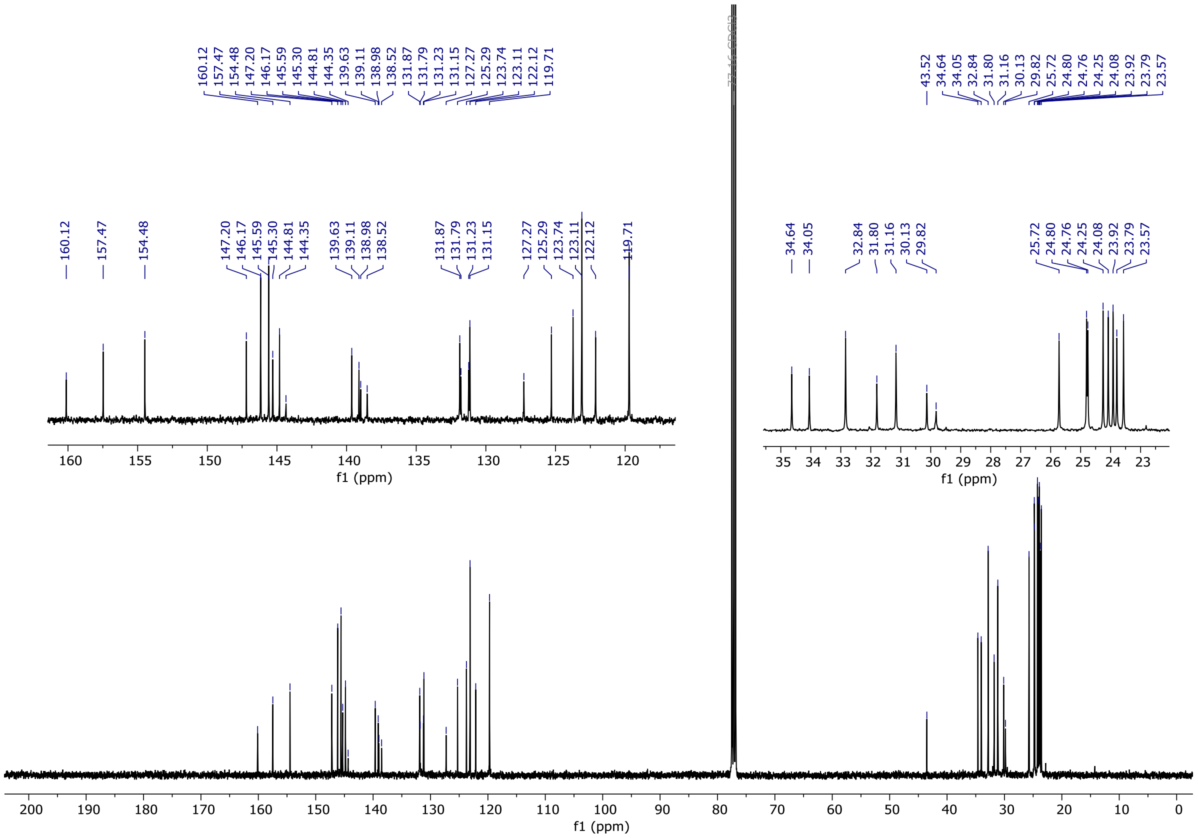

^{1}H NMR (500 MHz, C_6D_6) of **3**

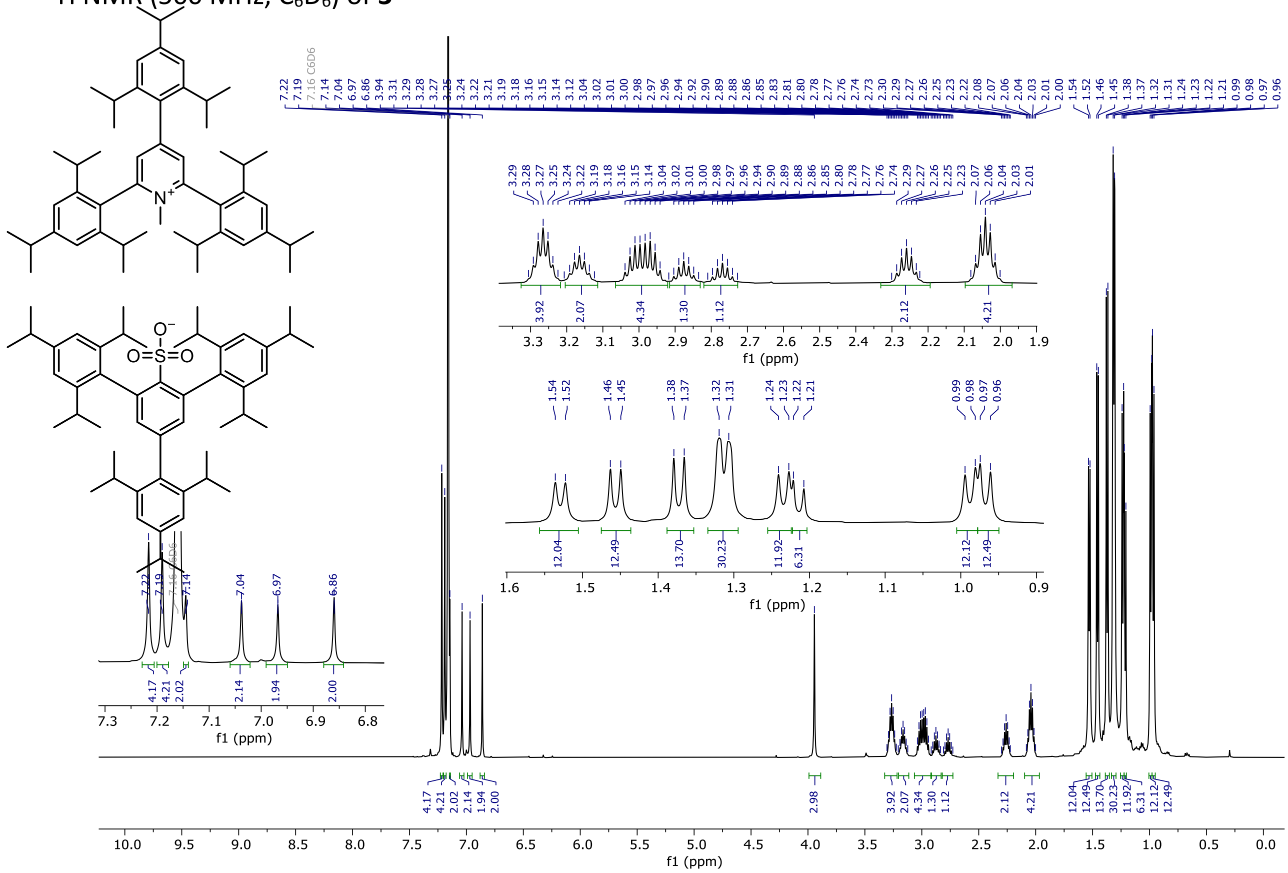

^{13}C NMR (126 MHz, C_6D_6) of **3**

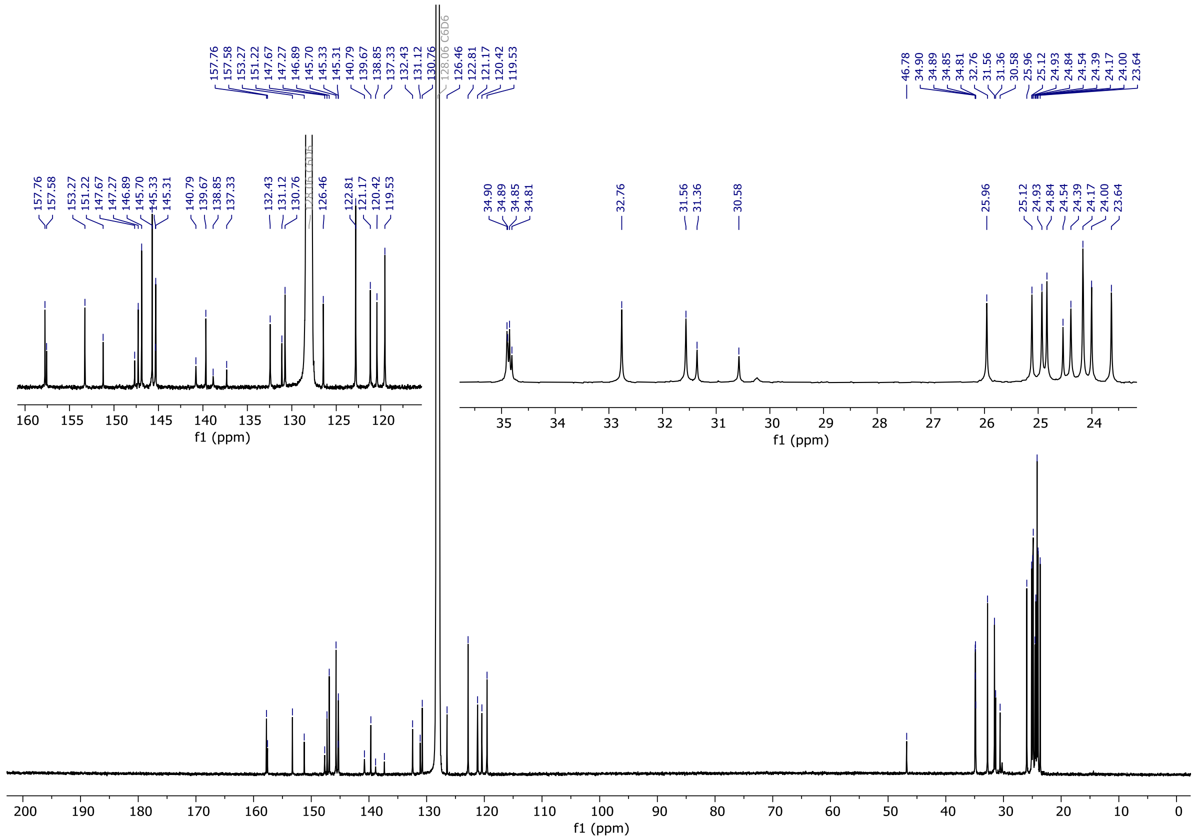

COSY (C_6D_6) of **3**

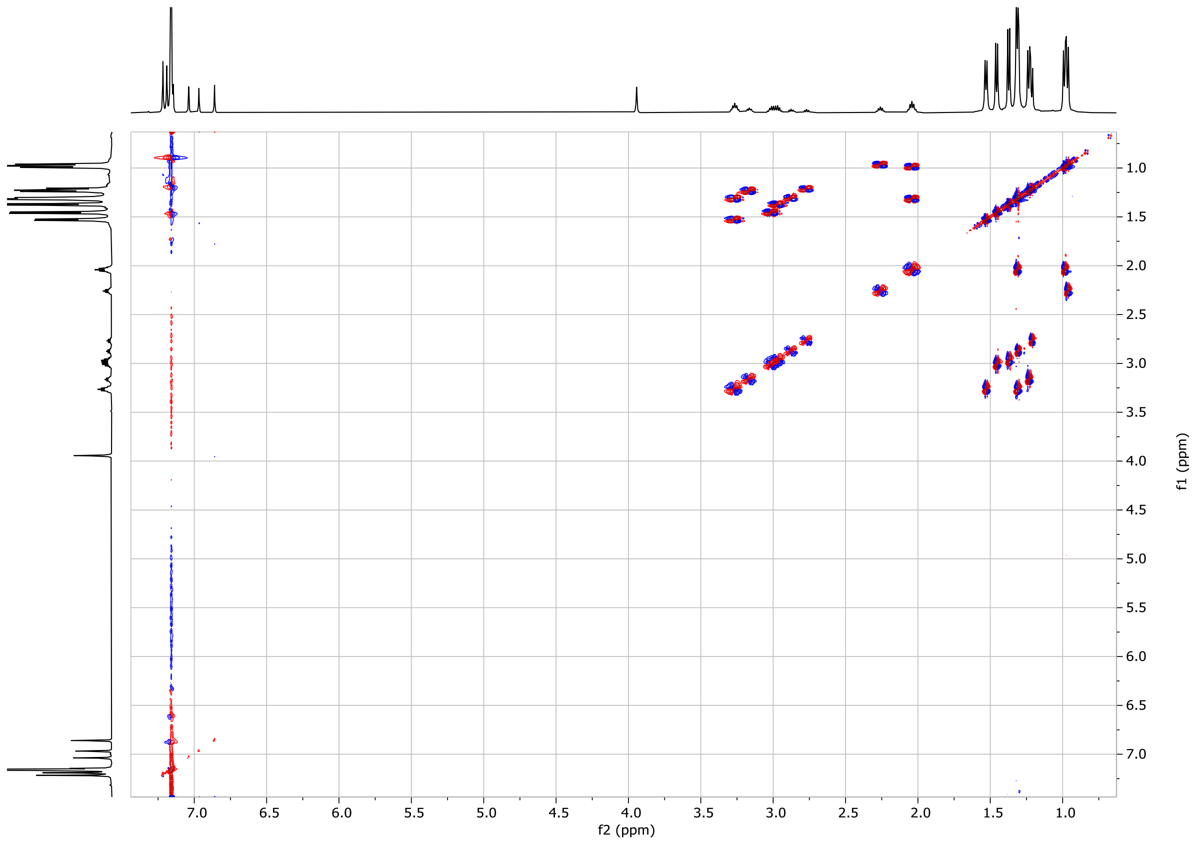

Excerpt from COSY (C_6D_6) of **3**

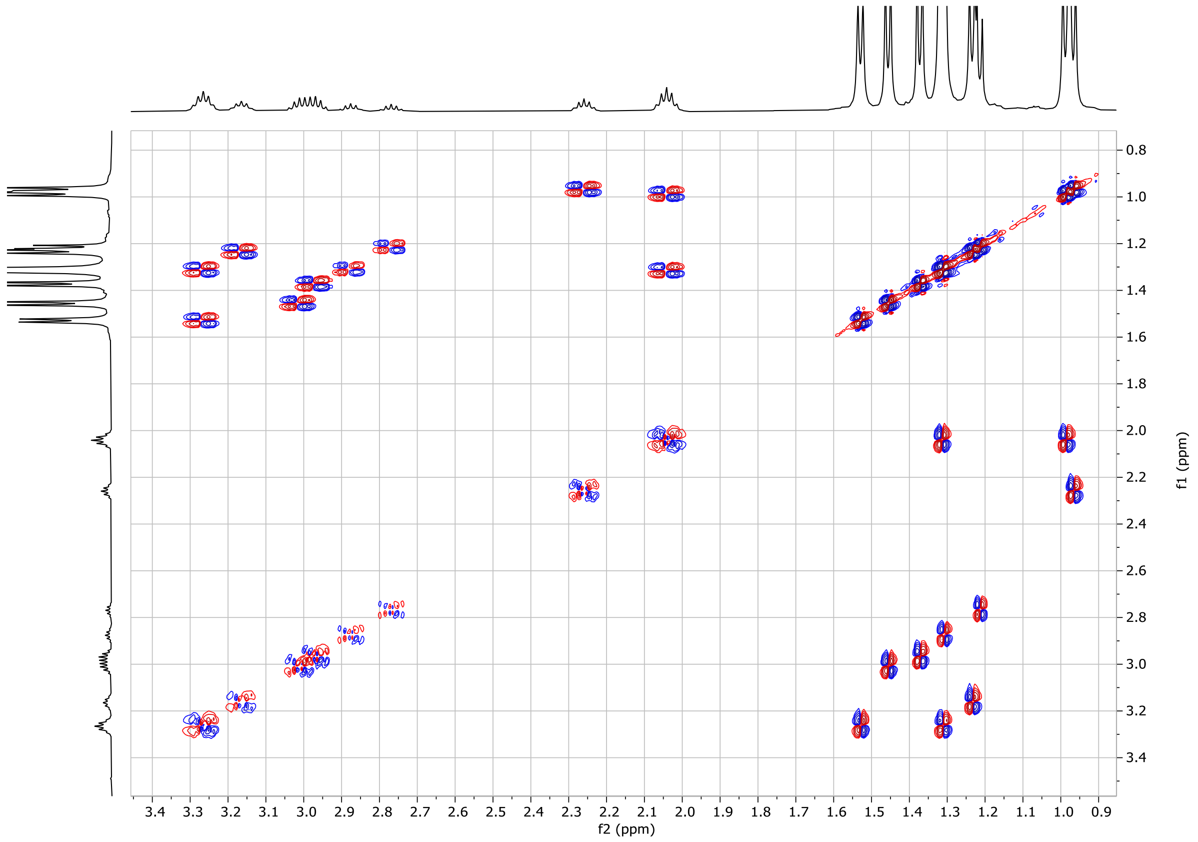

HSQC (C_6D_6) of **3**

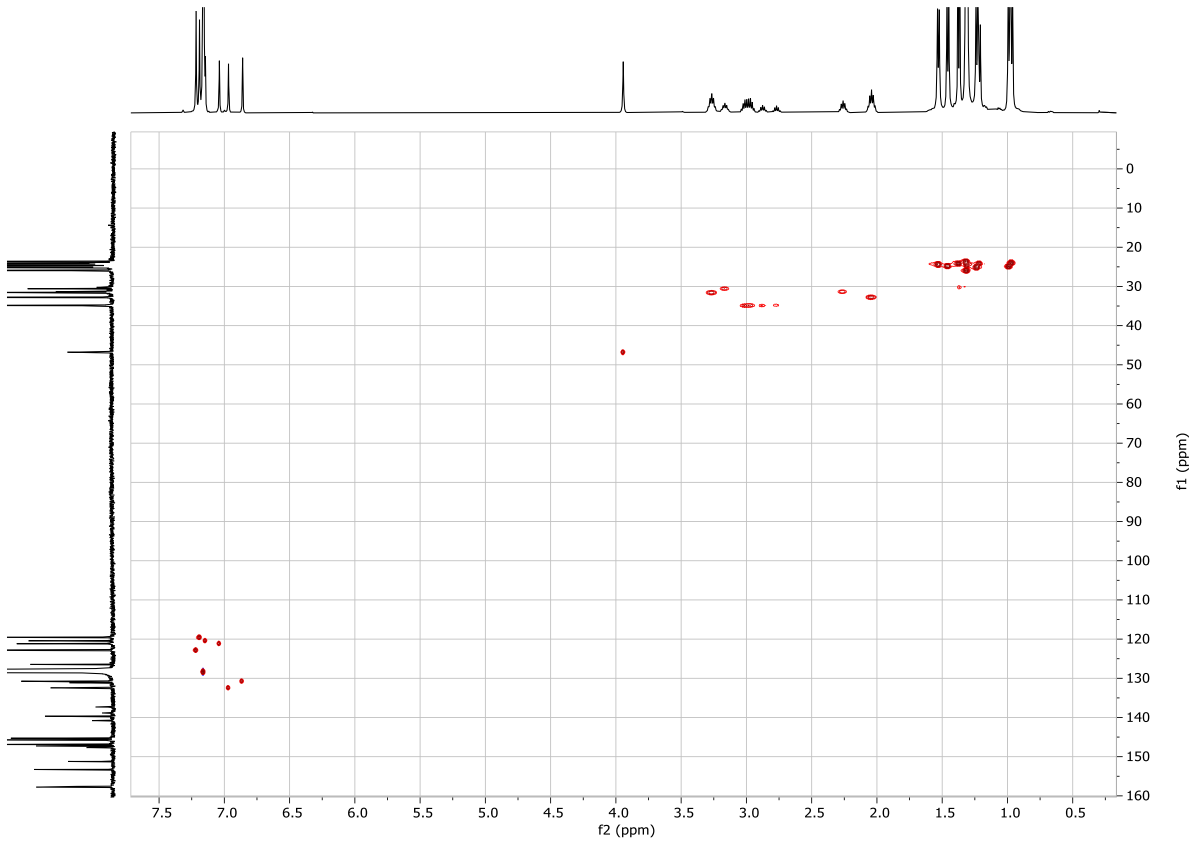

Excerpt from HSQC(C_6D_6) of **3**

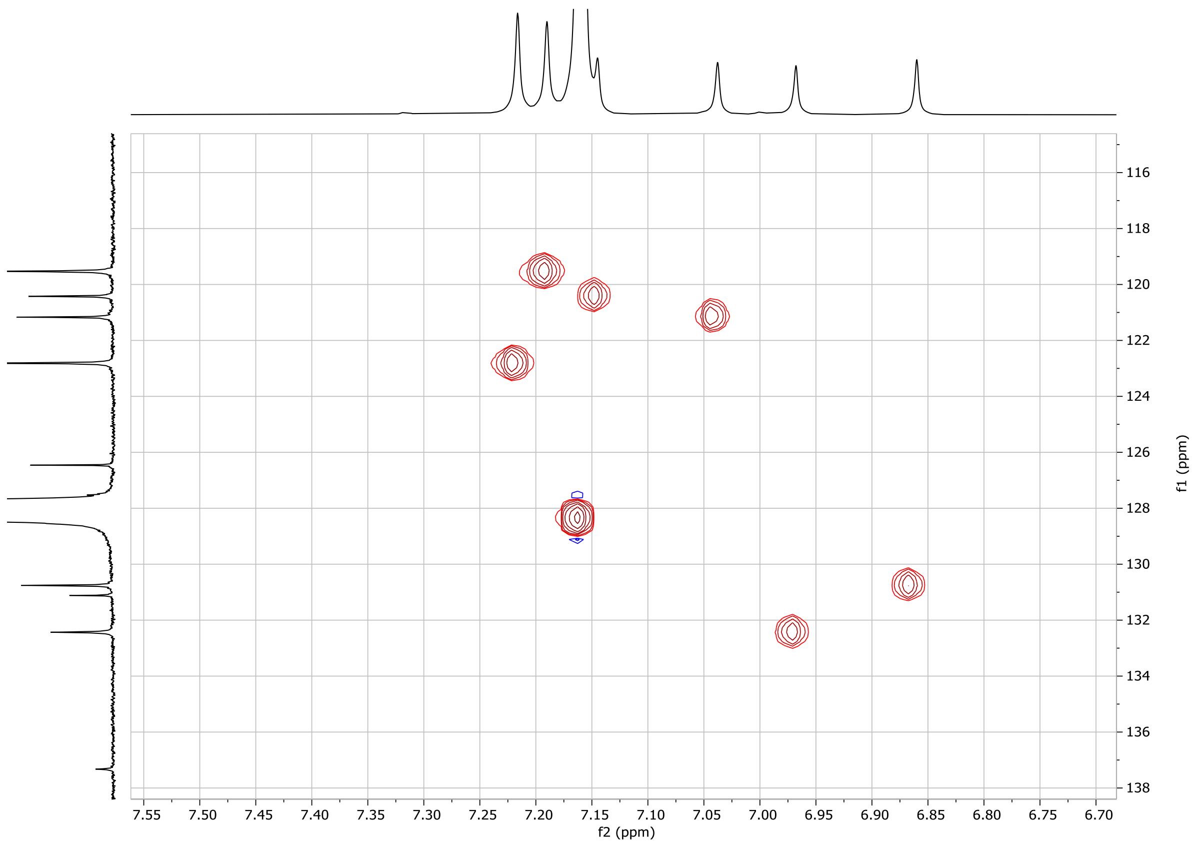

Excerpt from HSQC(C_6D_6) of **3**

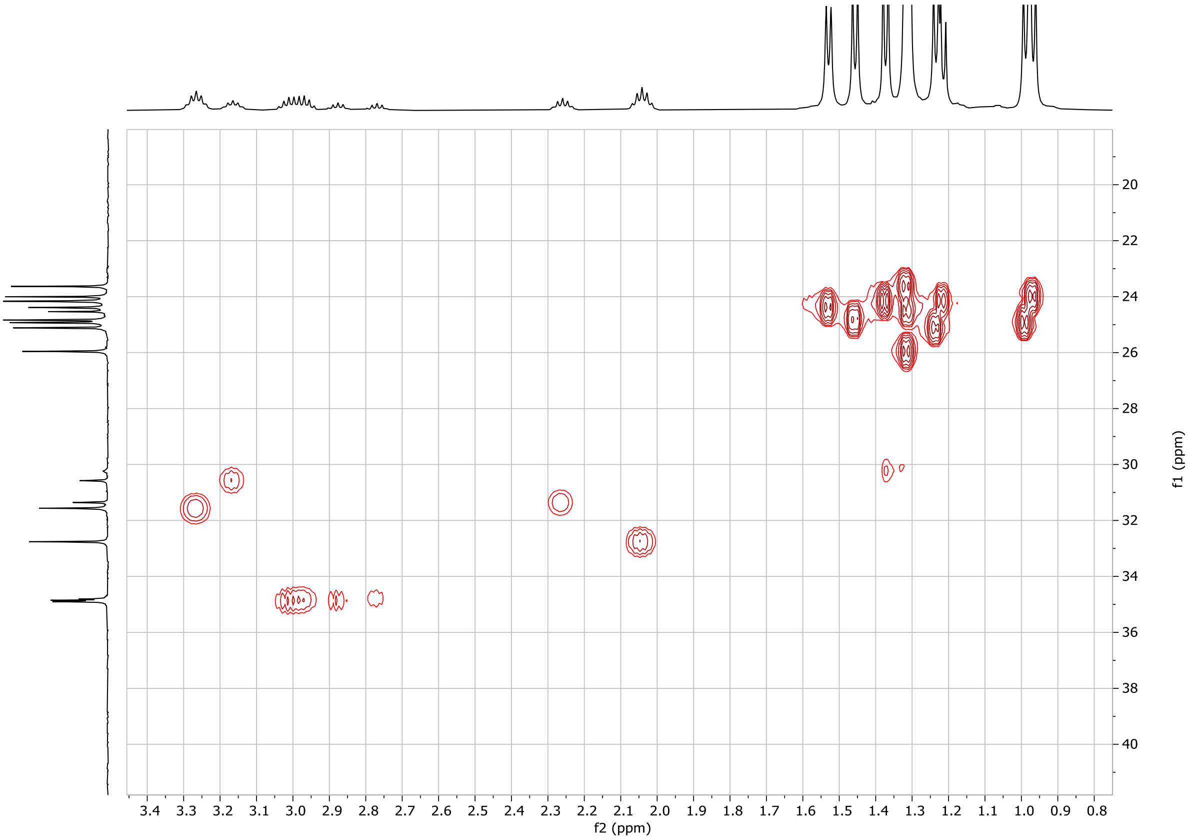

HMBC(C_6D_6) of **3**

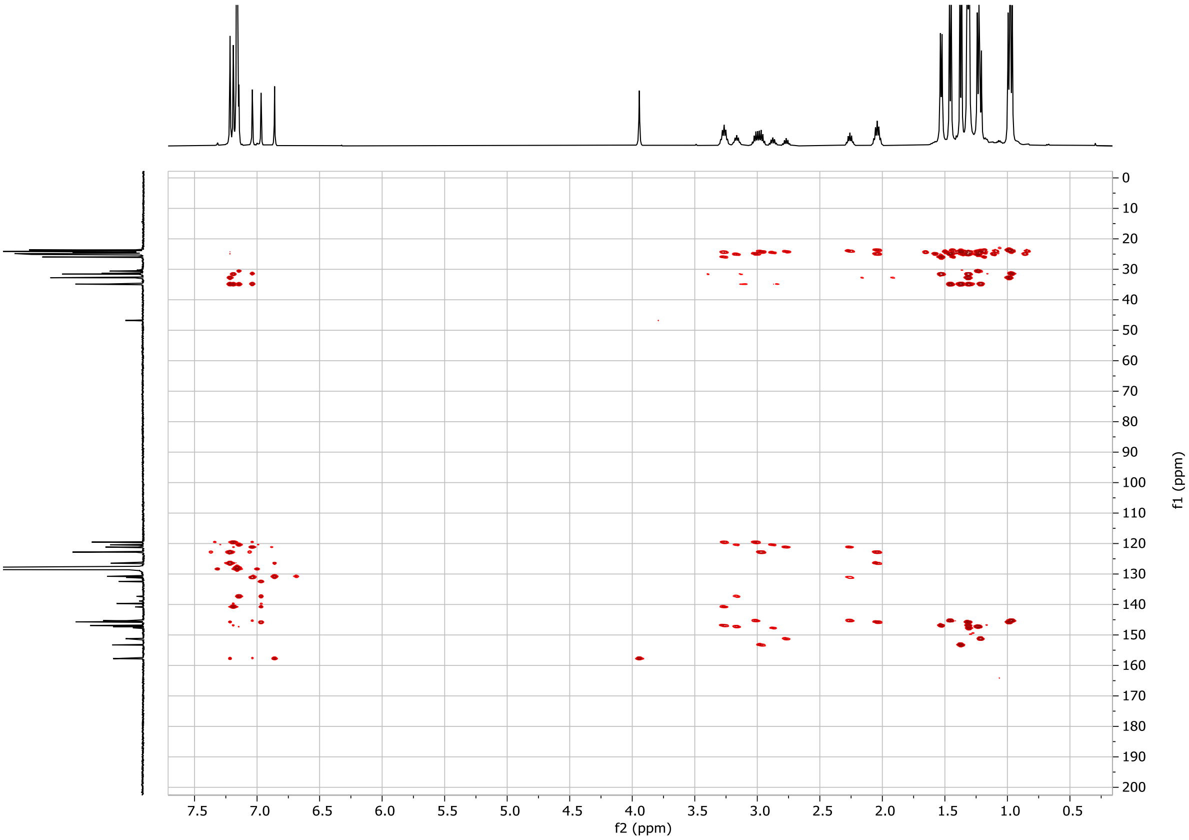

Excerpt from HMBC (C_6D_6) of **3**

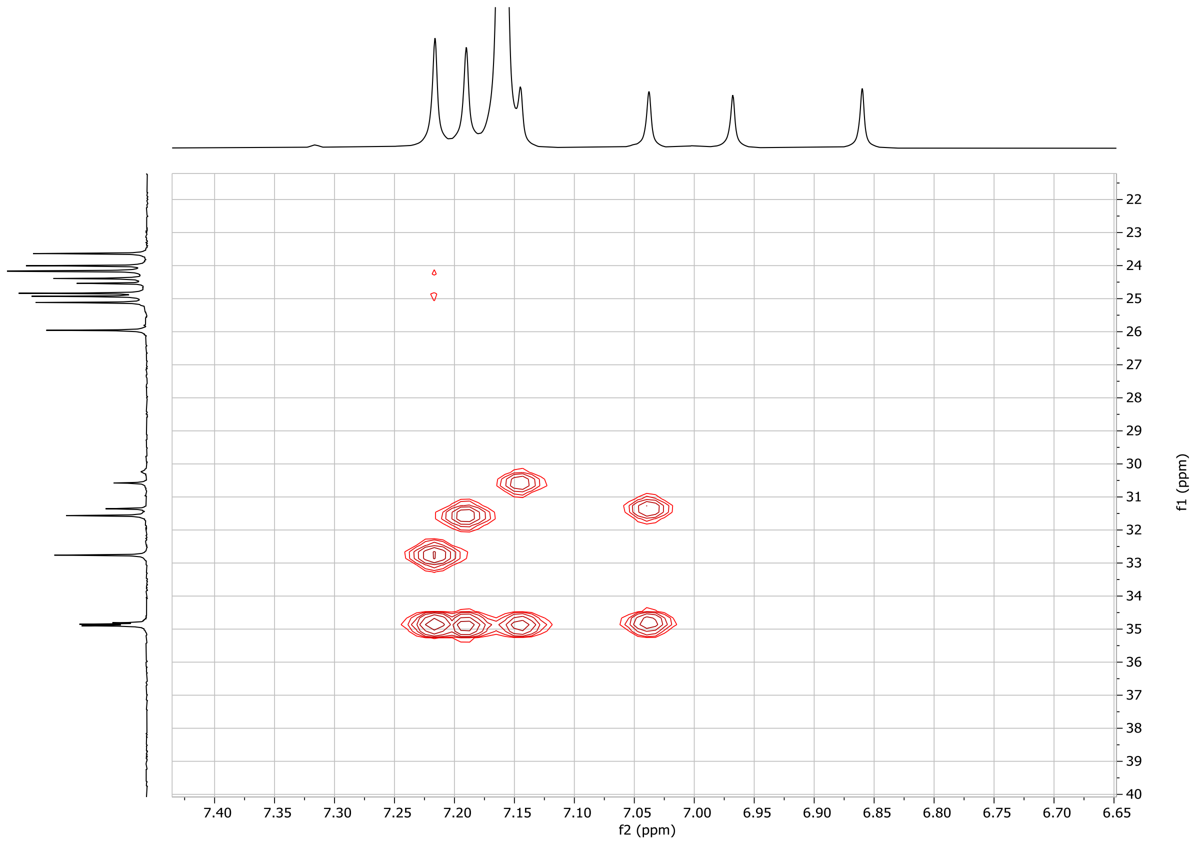

Excerpt from HMBC (C_6D_6) of **3**

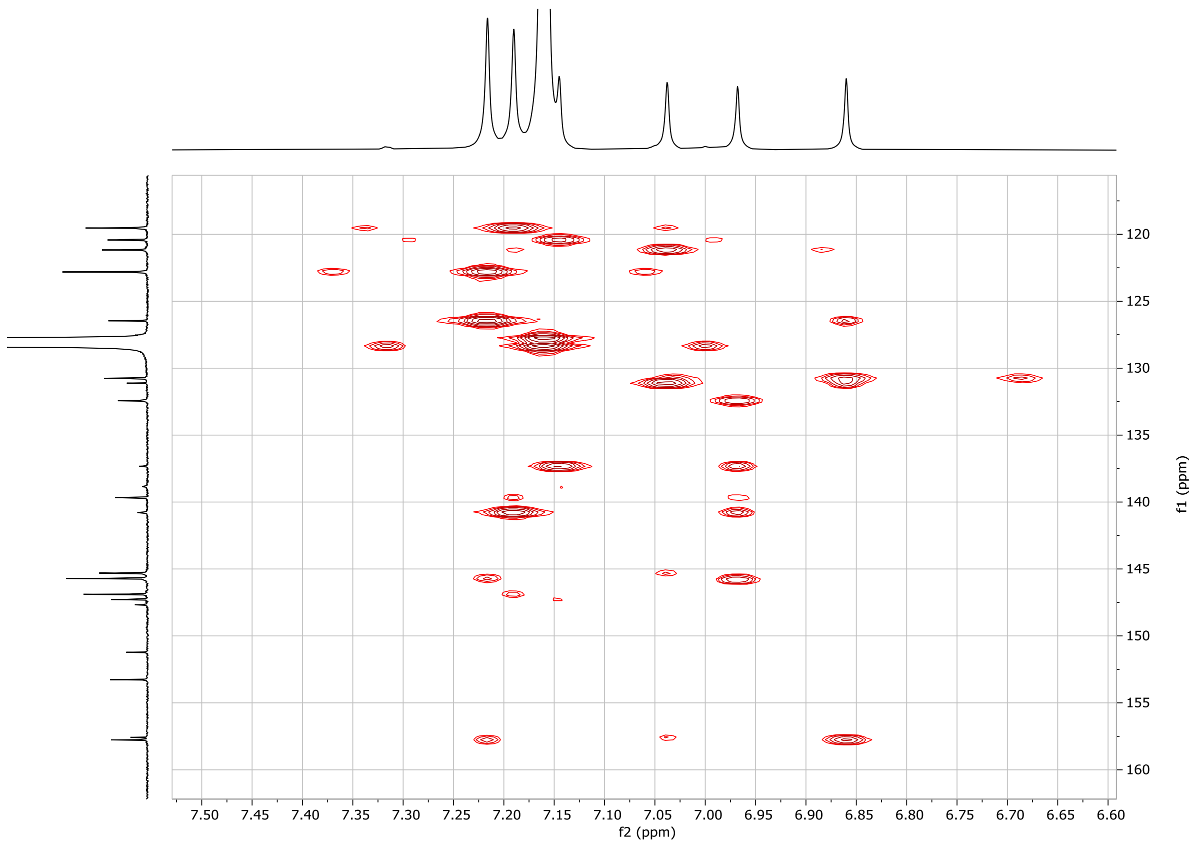

ROESY (C_6D_6) of **3**

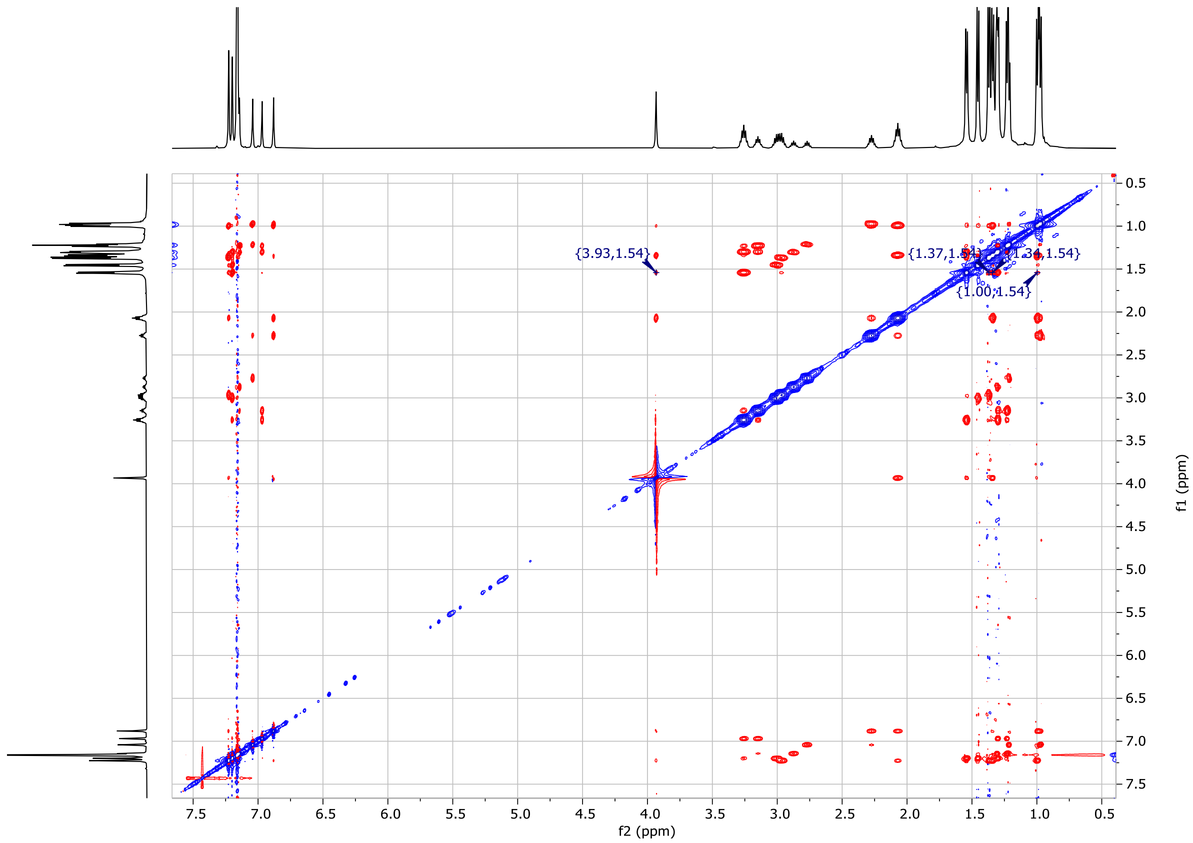

Excerpt from ROESY (C_6D_6) of **3**

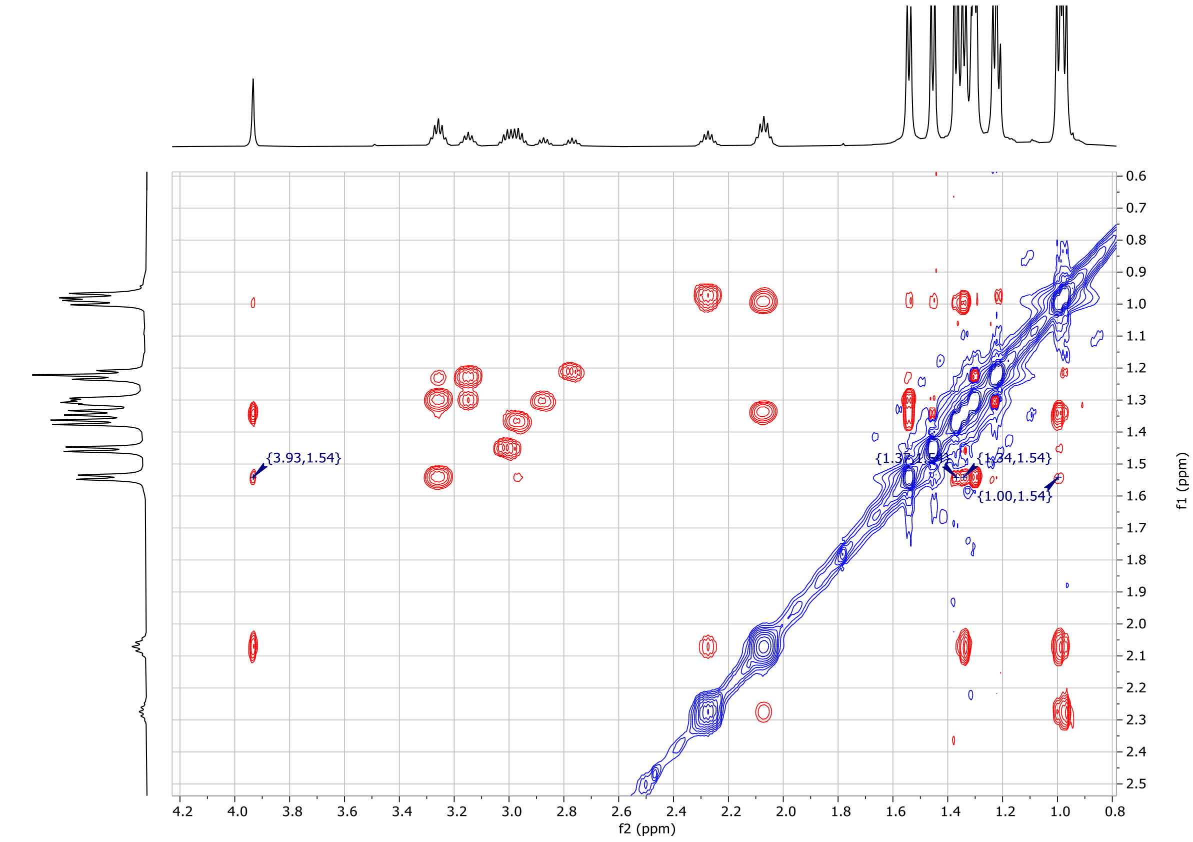

Excerpt from ROESY (C_6D_6) of **3**

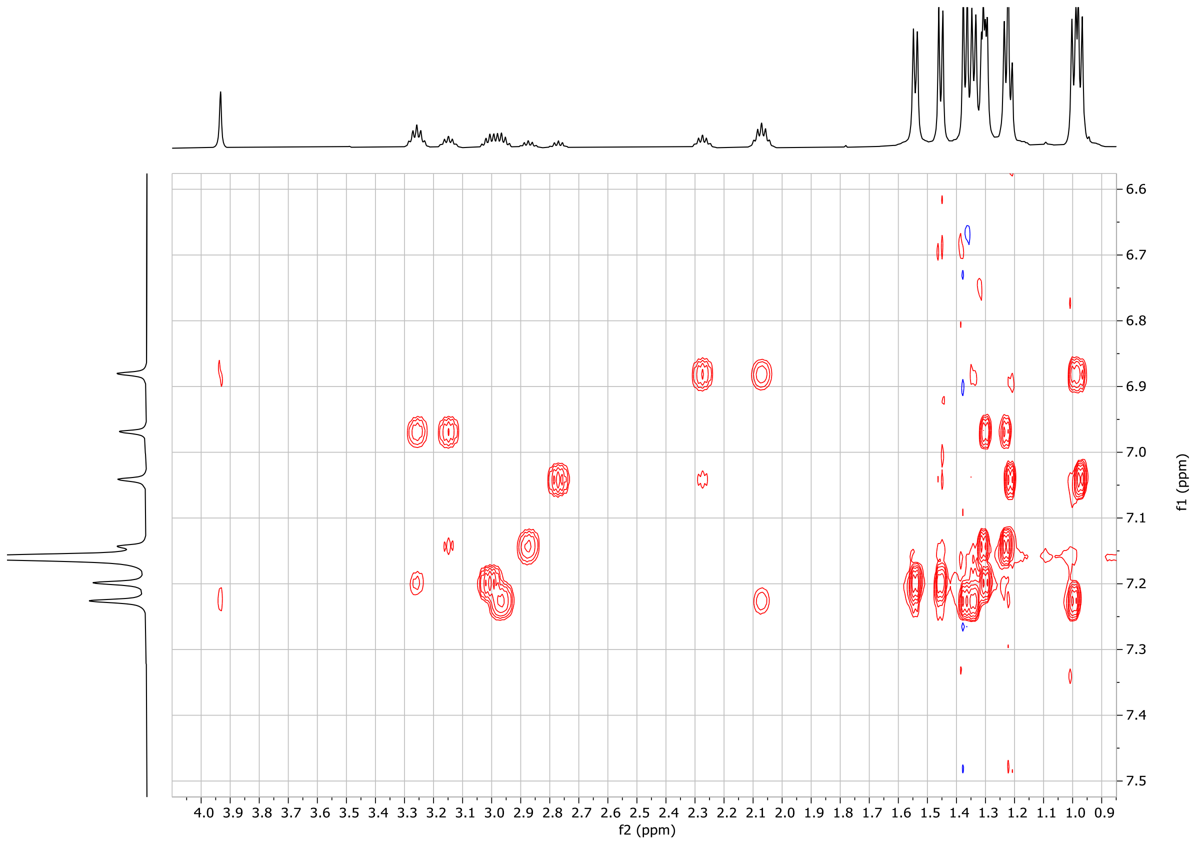

¹H NMR (500 MHz, toluene-d_8) of **3**

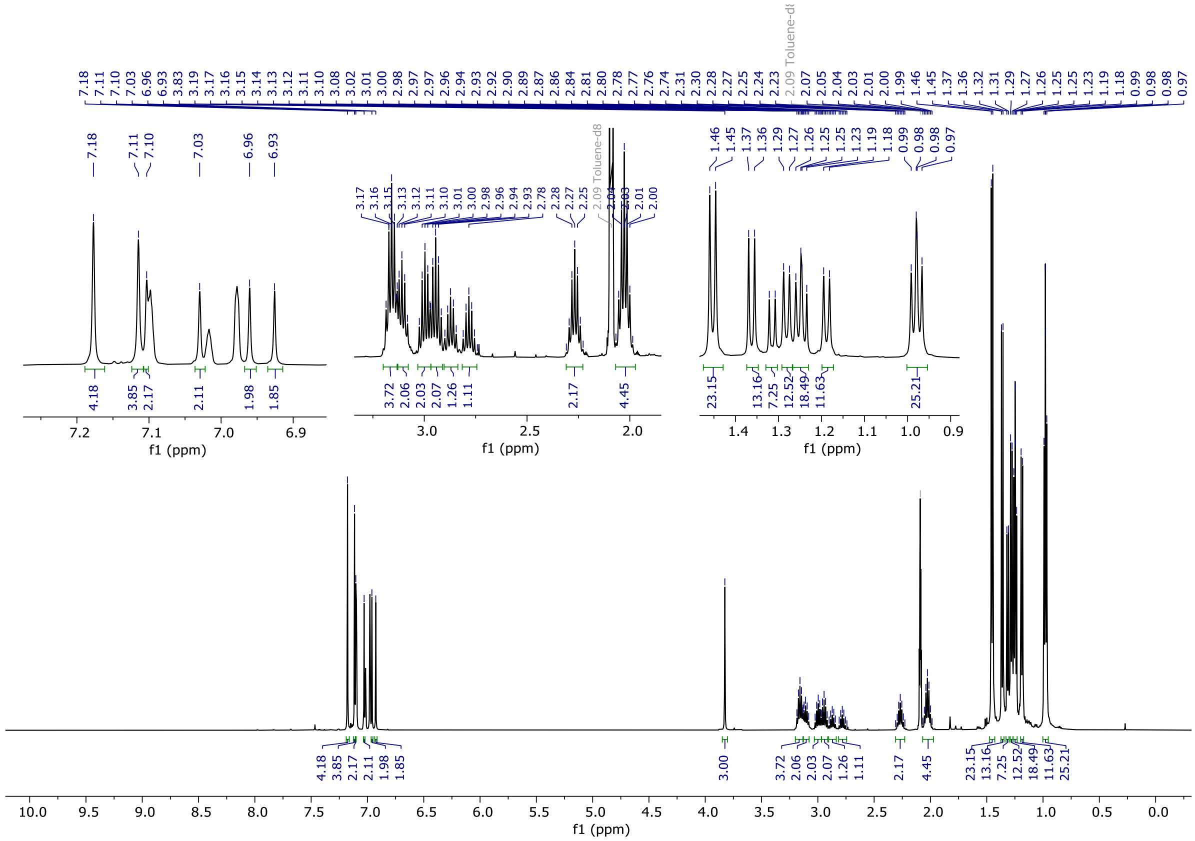

COSY (toluene-d_8) of **3**

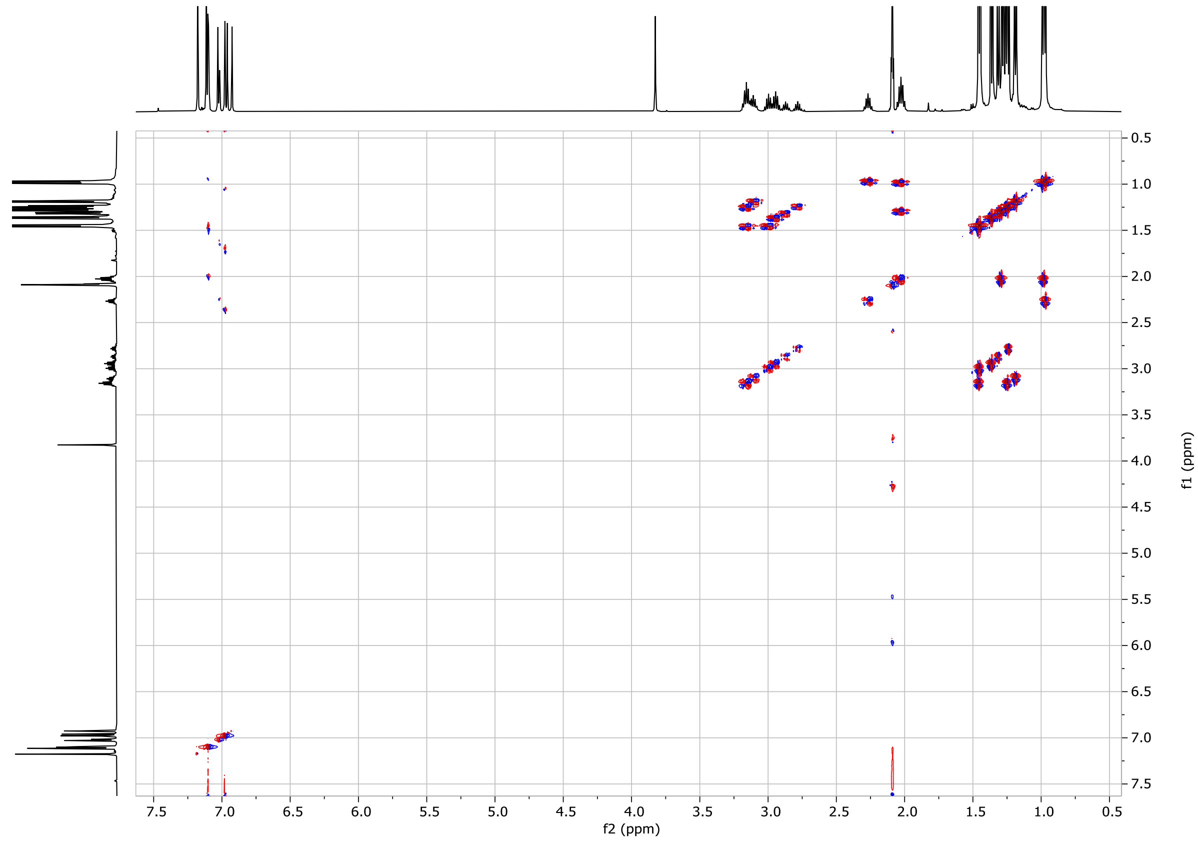

Excerpt from COSY (toluene-d_8) of **3**

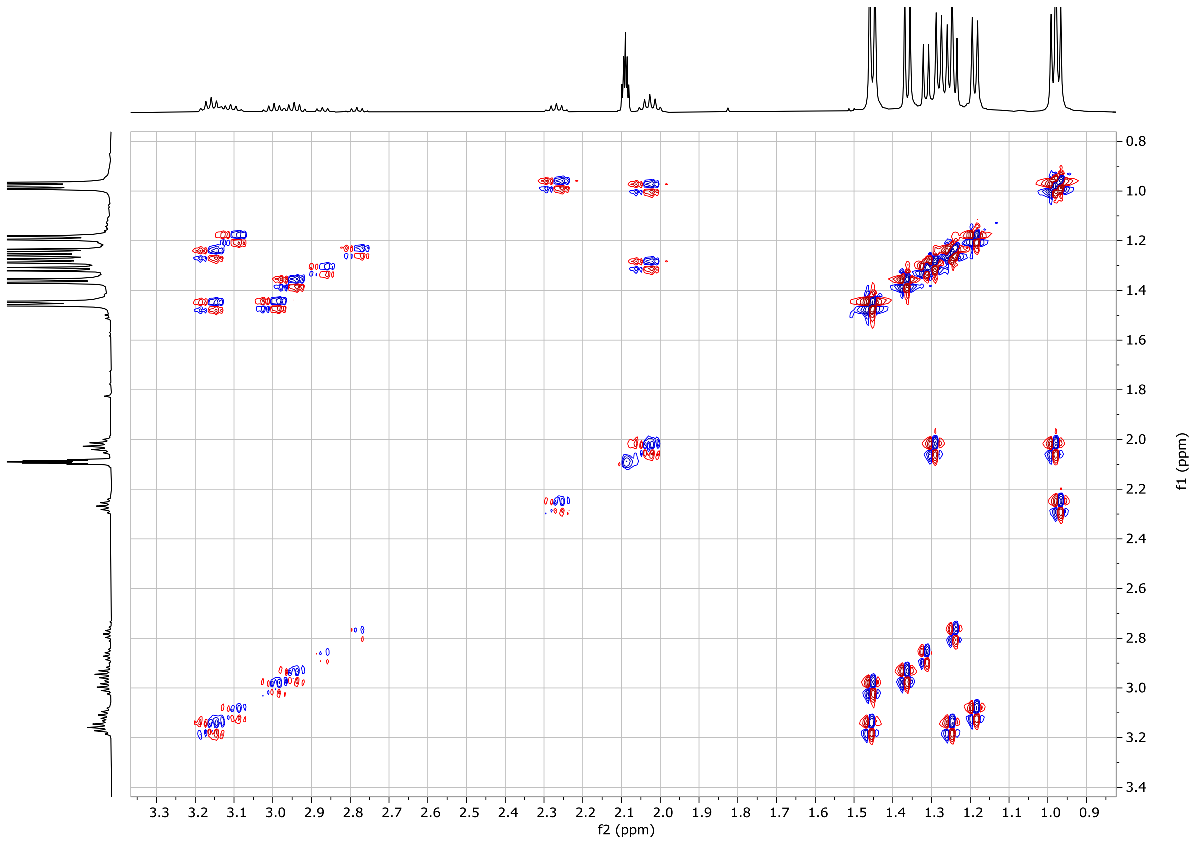

ROESY (toluene-d_8) of **3**

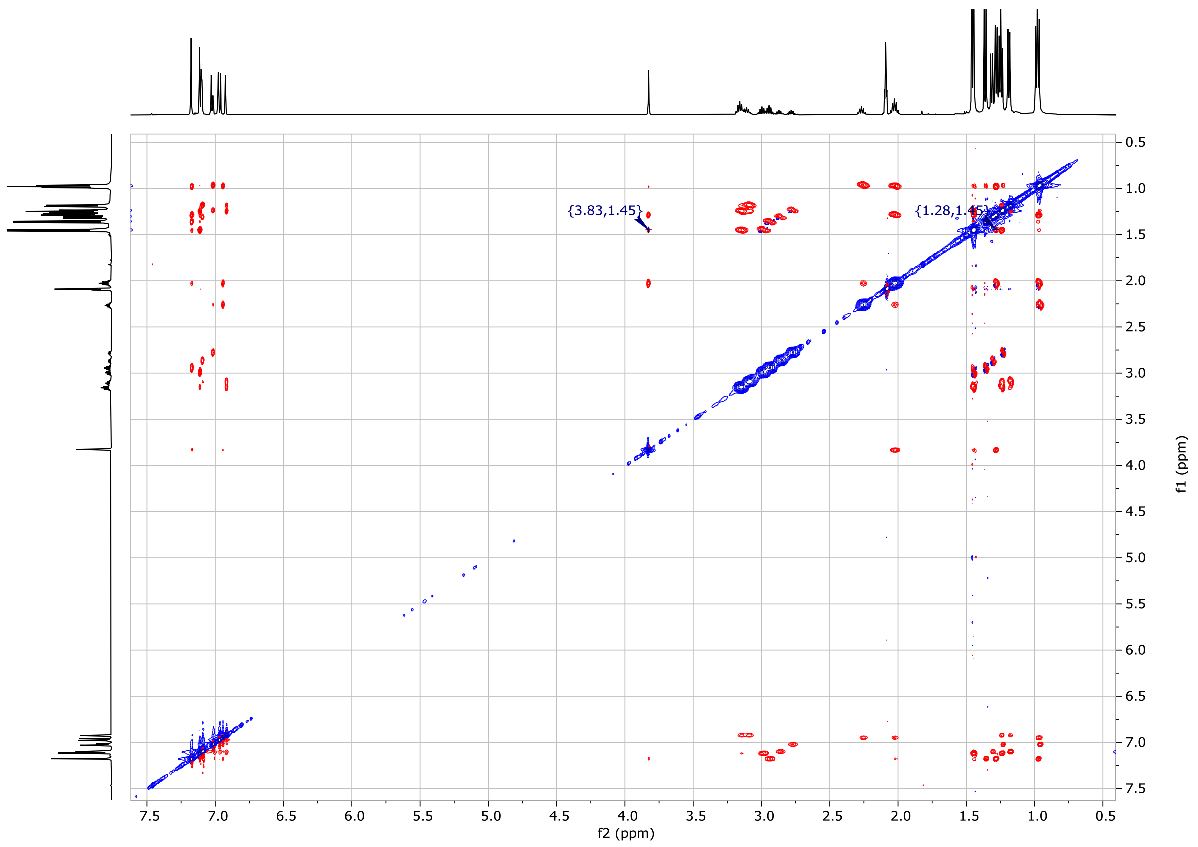

Excerpt from ROESY (toluene-d_8) of **3**

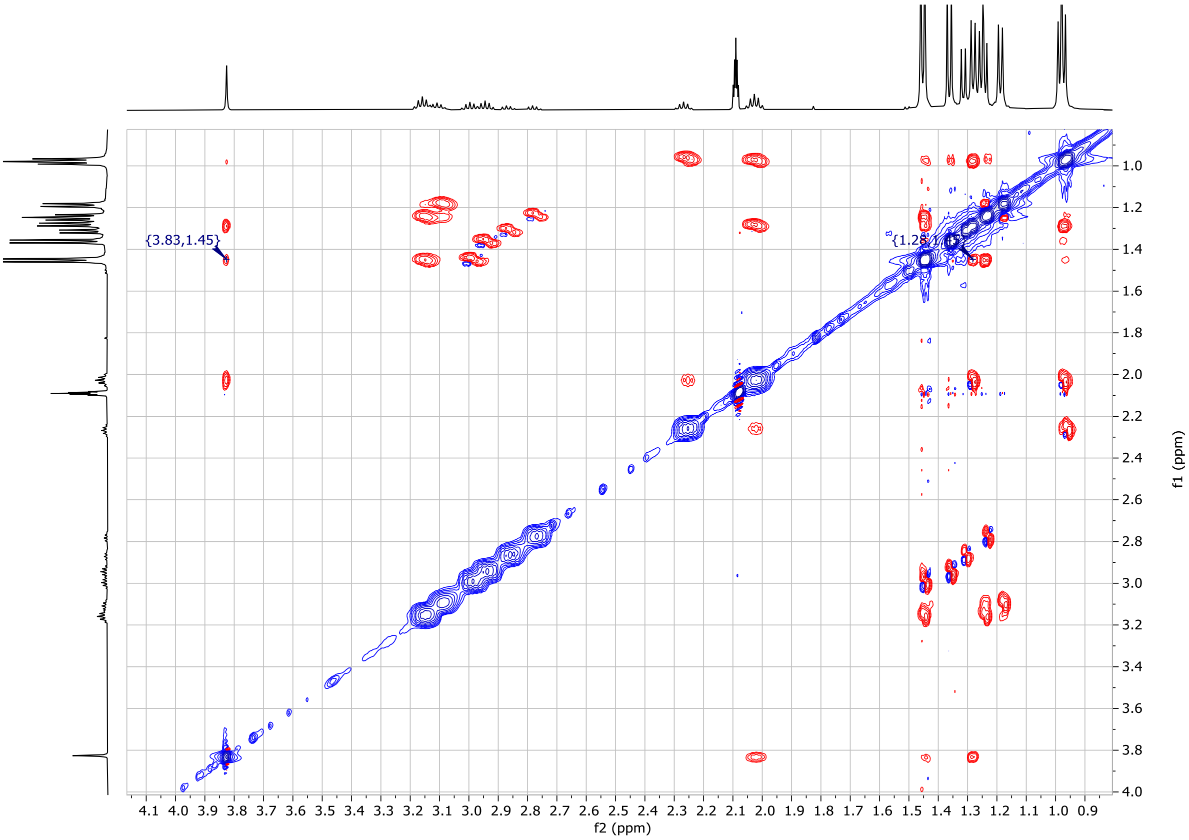

Excerpt from ROESY (toluene-d_8) of **3**

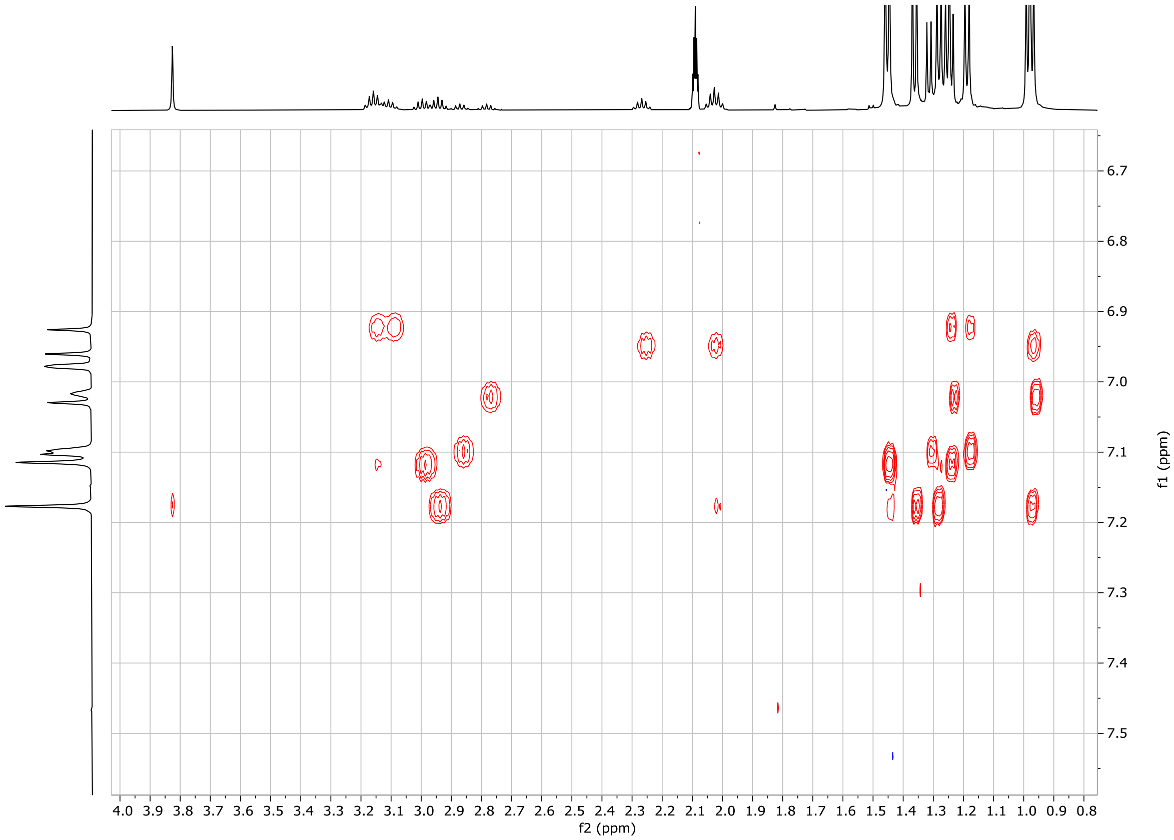

^1H NMR (500 MHz, DMSO-d_6) of **3**

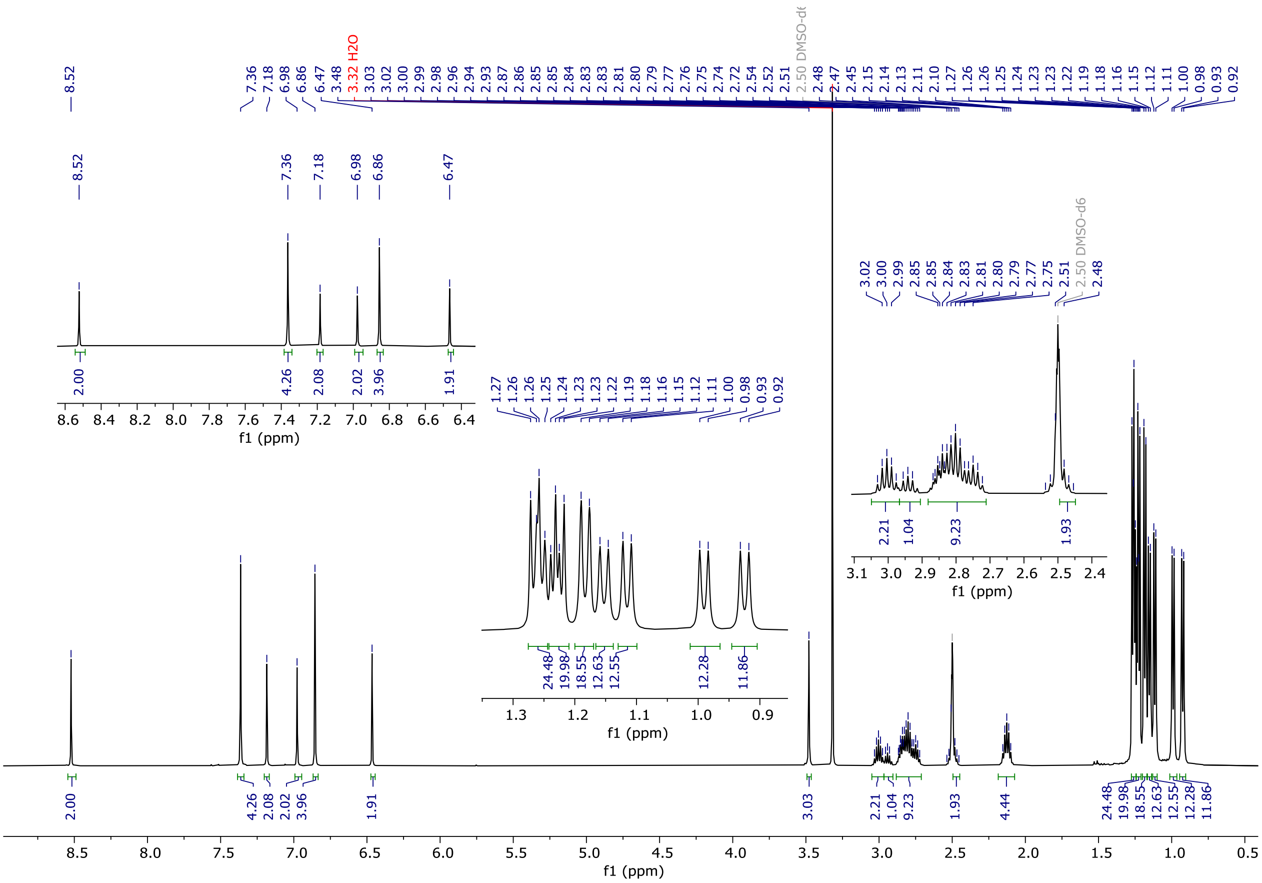

COSY (DMSO-d_6) of **3**

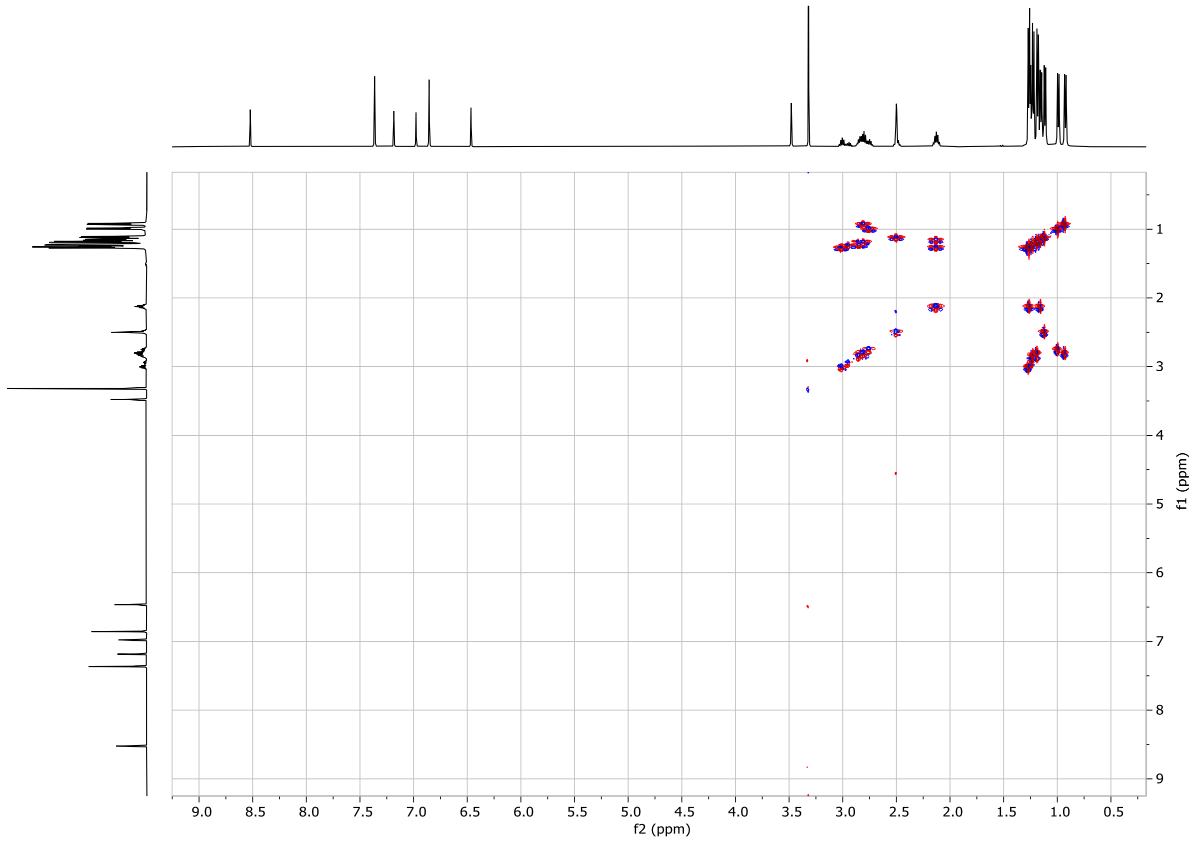

Excerpt from COSY (DMSO-d_6) of **3**

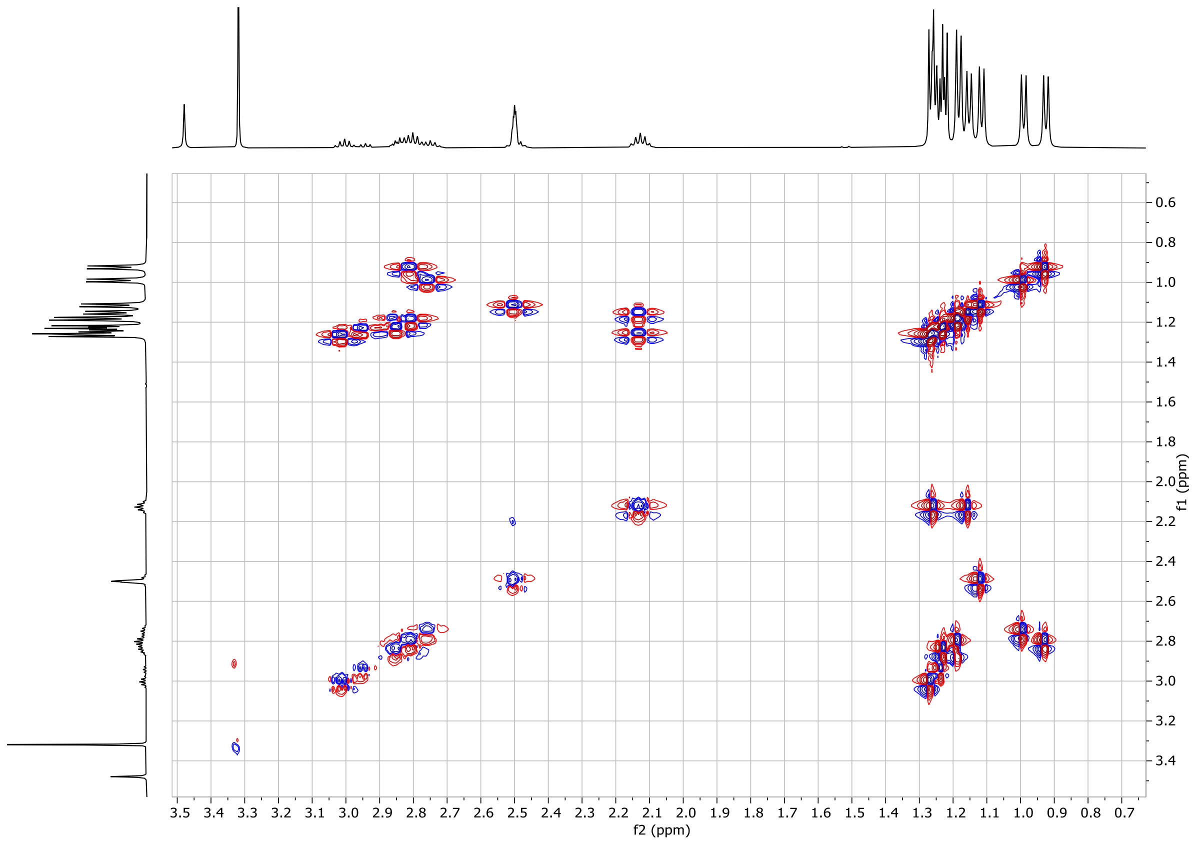

ROESY (DMSO-d_6) of **3**

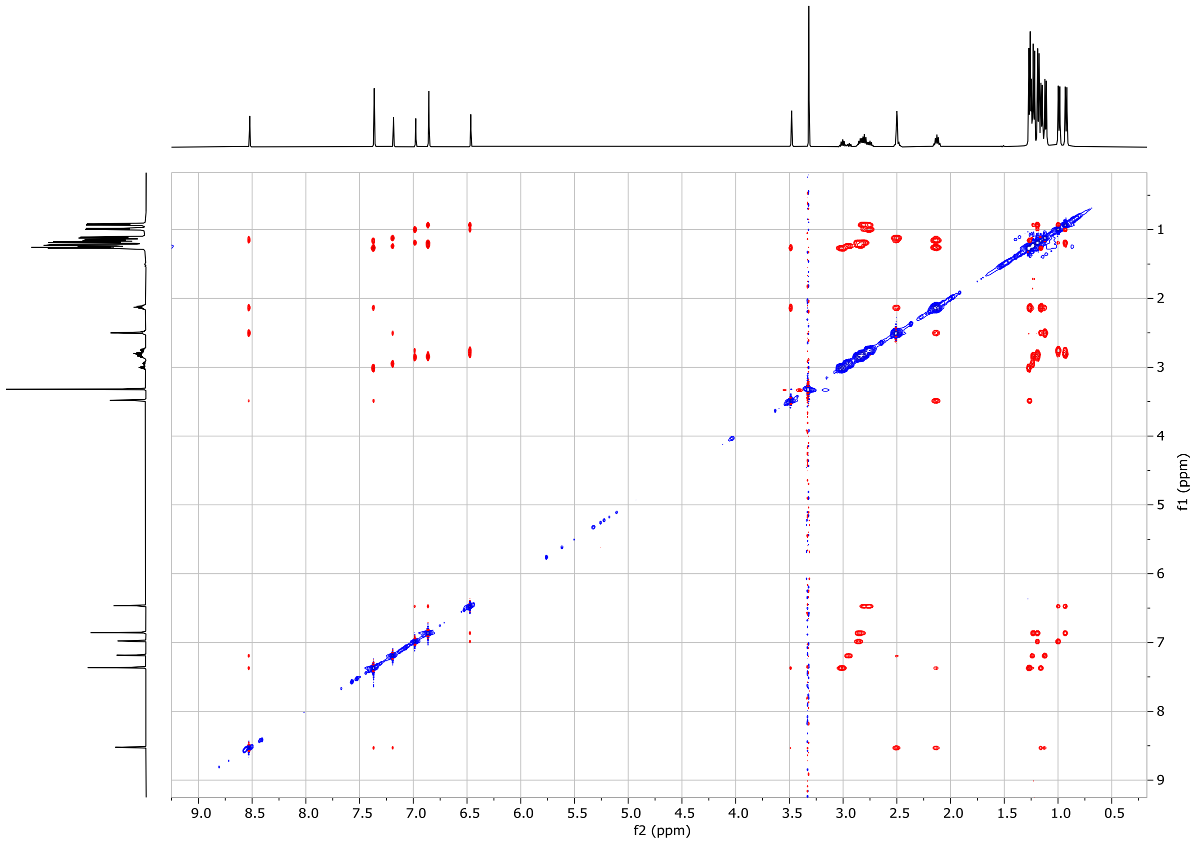

Excerpt from ROESY (DMSO-d_6) of **3**

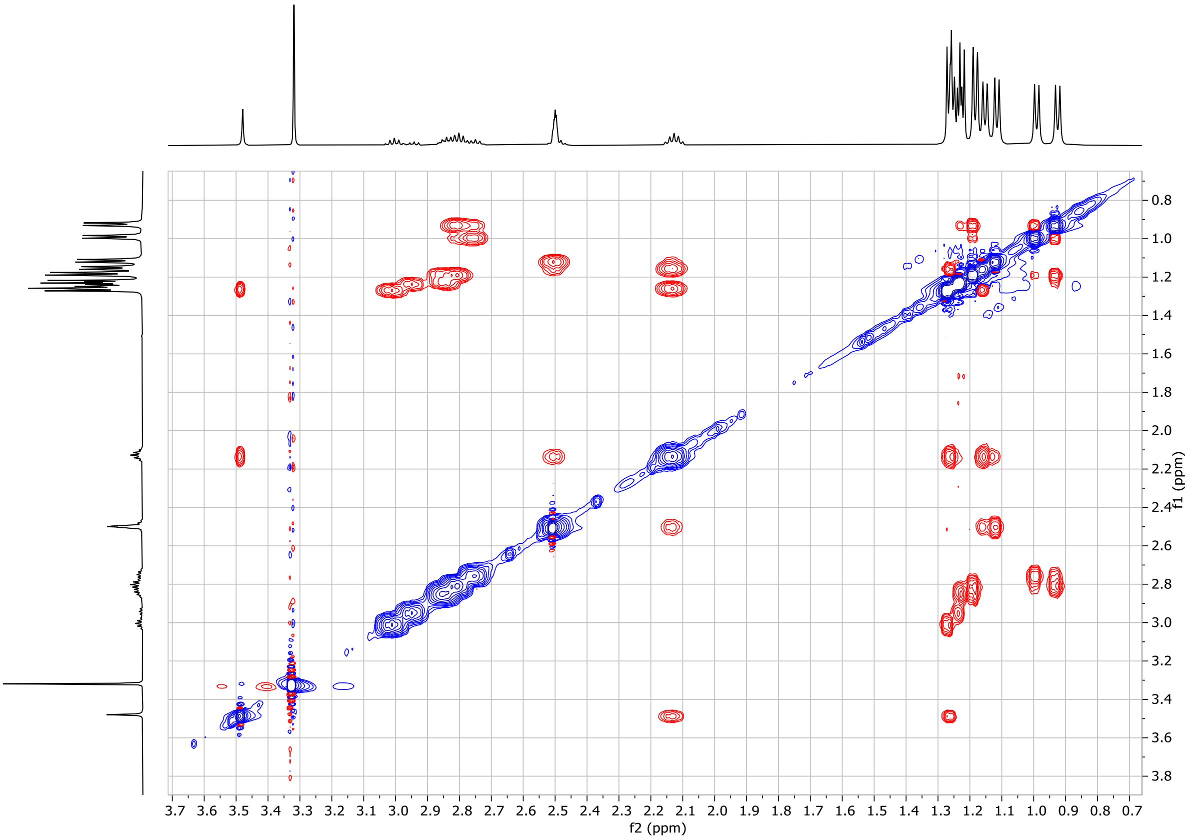

Excerpt from ROESY (DMSO-d6) of **3**

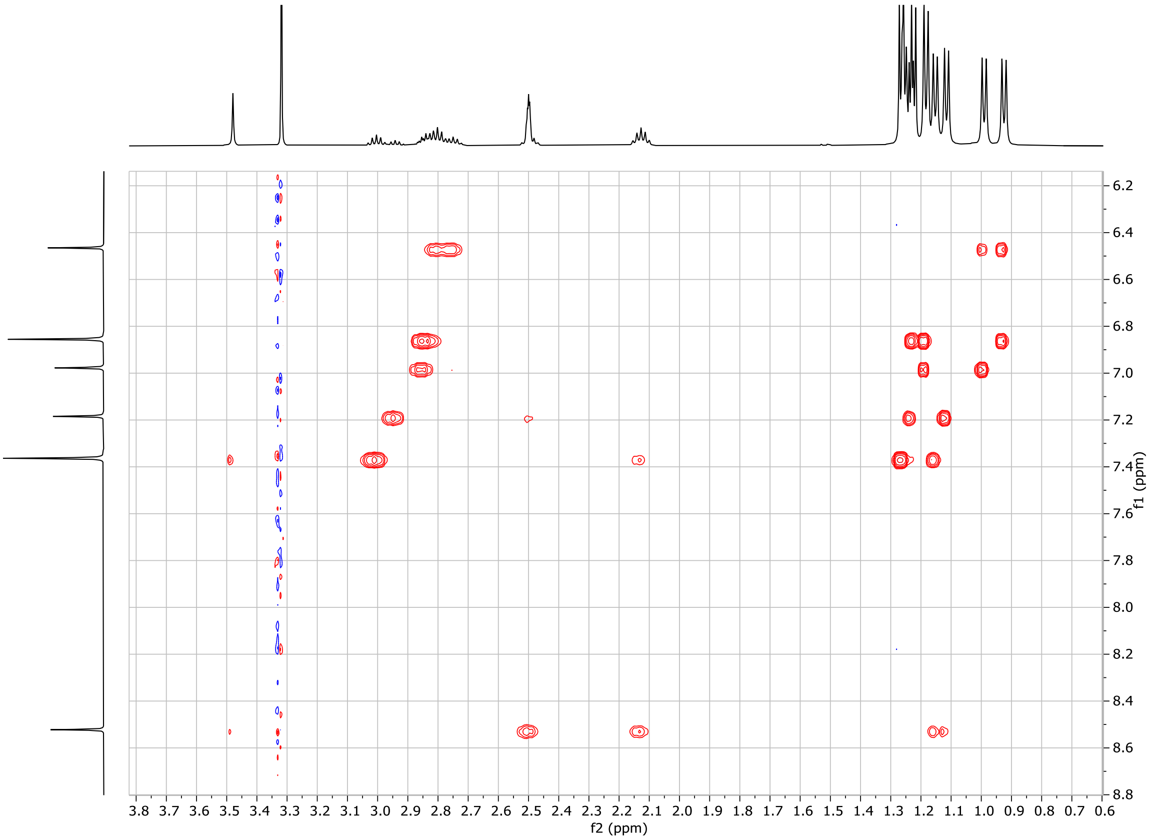

^{1}H NMR (400 MHz, $CDCl_3$) of **4**

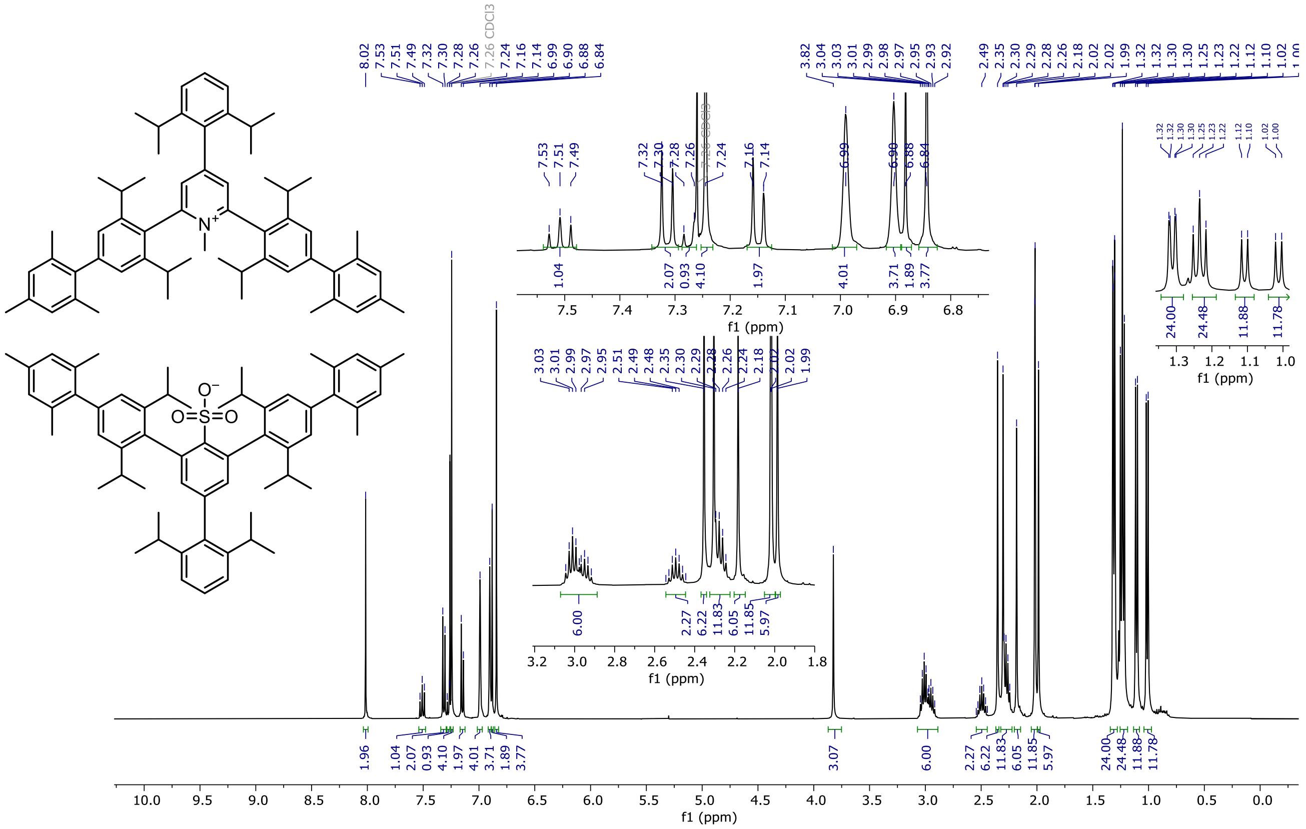

^{13}C NMR (101 MHz, $CDCl_3$) of **4**

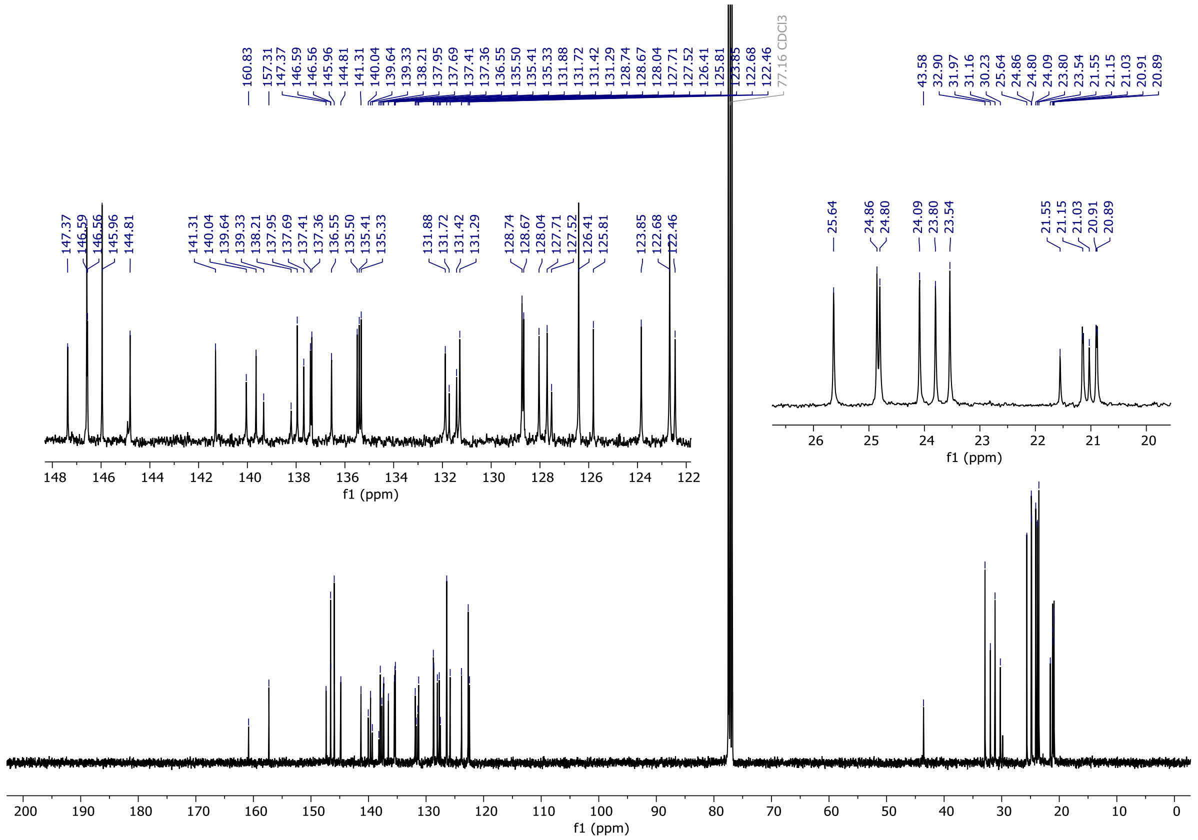

^{1}H NMR (500 MHz, $CDCl_3$) of **5**

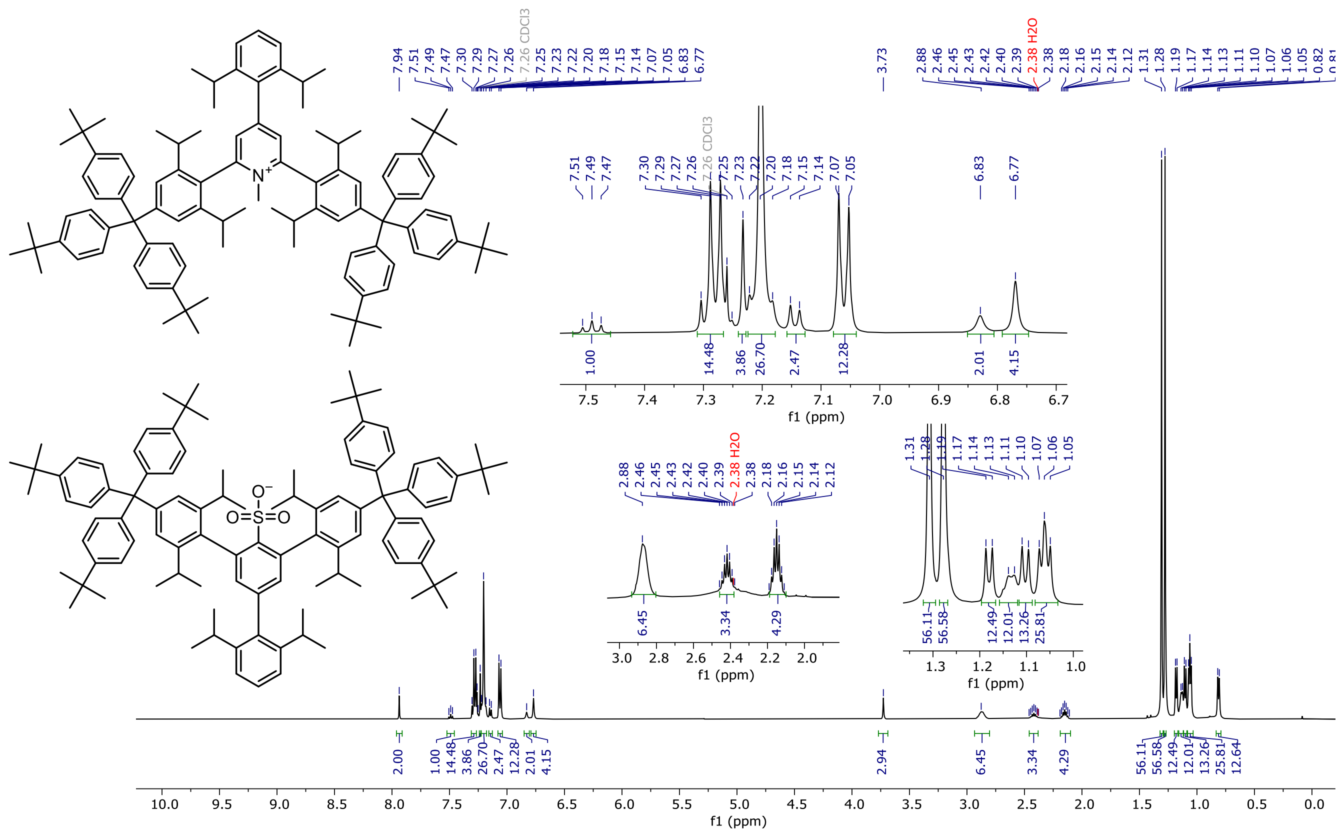

^{13}C NMR (126 MHz, $CDCl_3$) of **5**

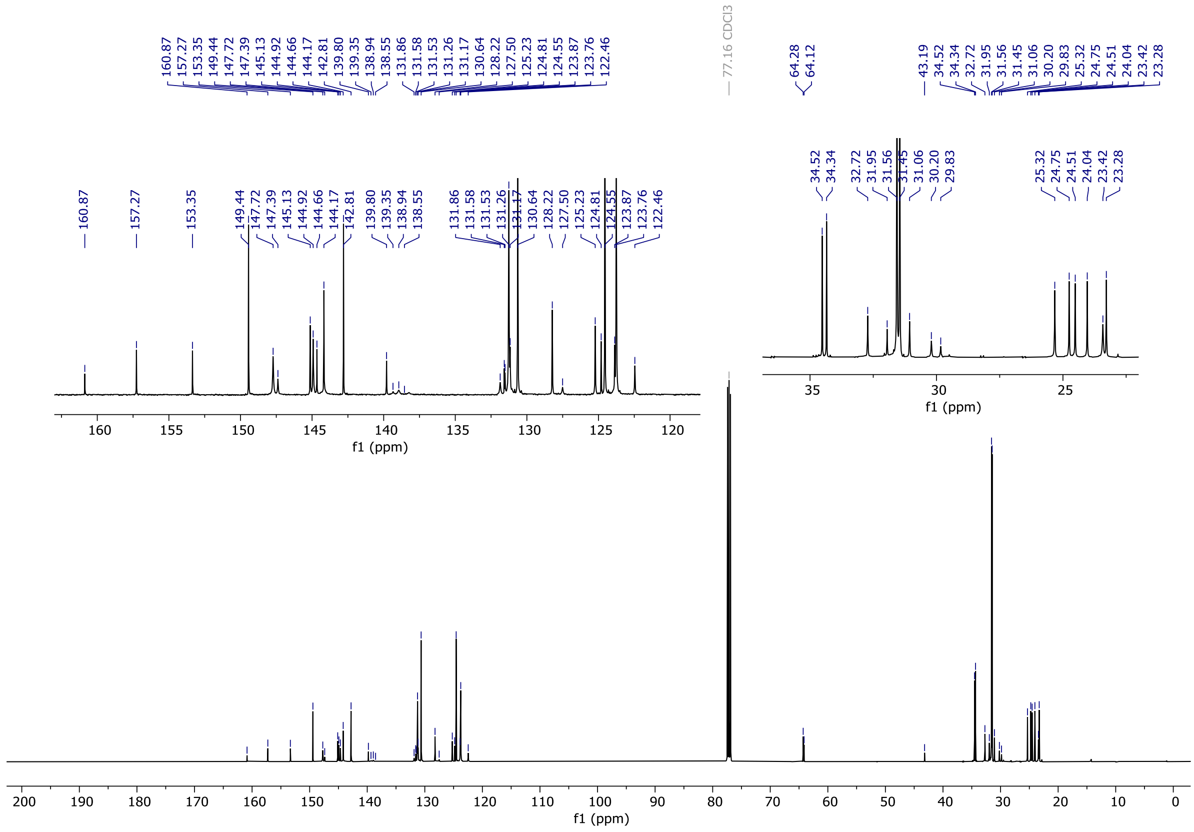

^{1}H NMR (400 MHz, $CDCl_3$) of **6**

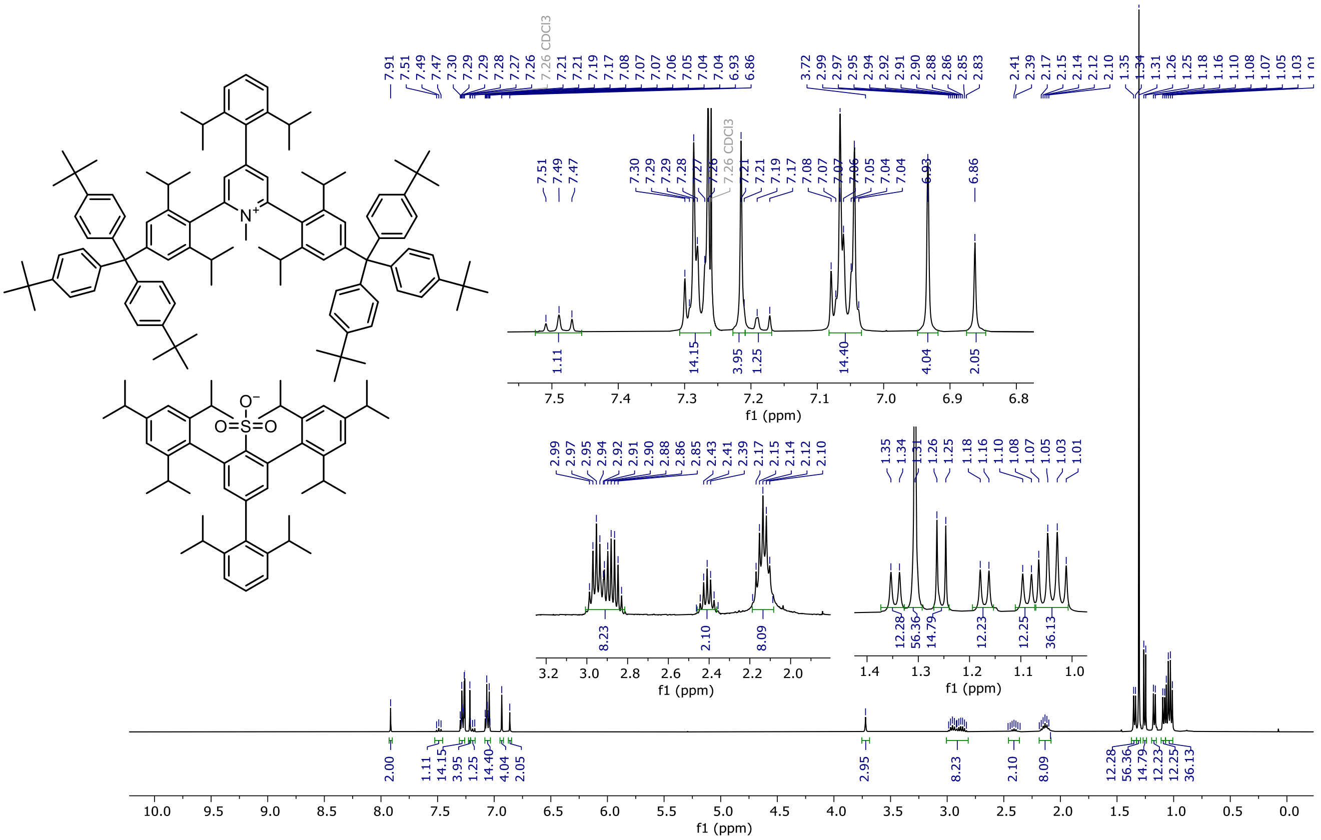

^{13}C NMR (101 MHz, $CDCl_3$) of **6**

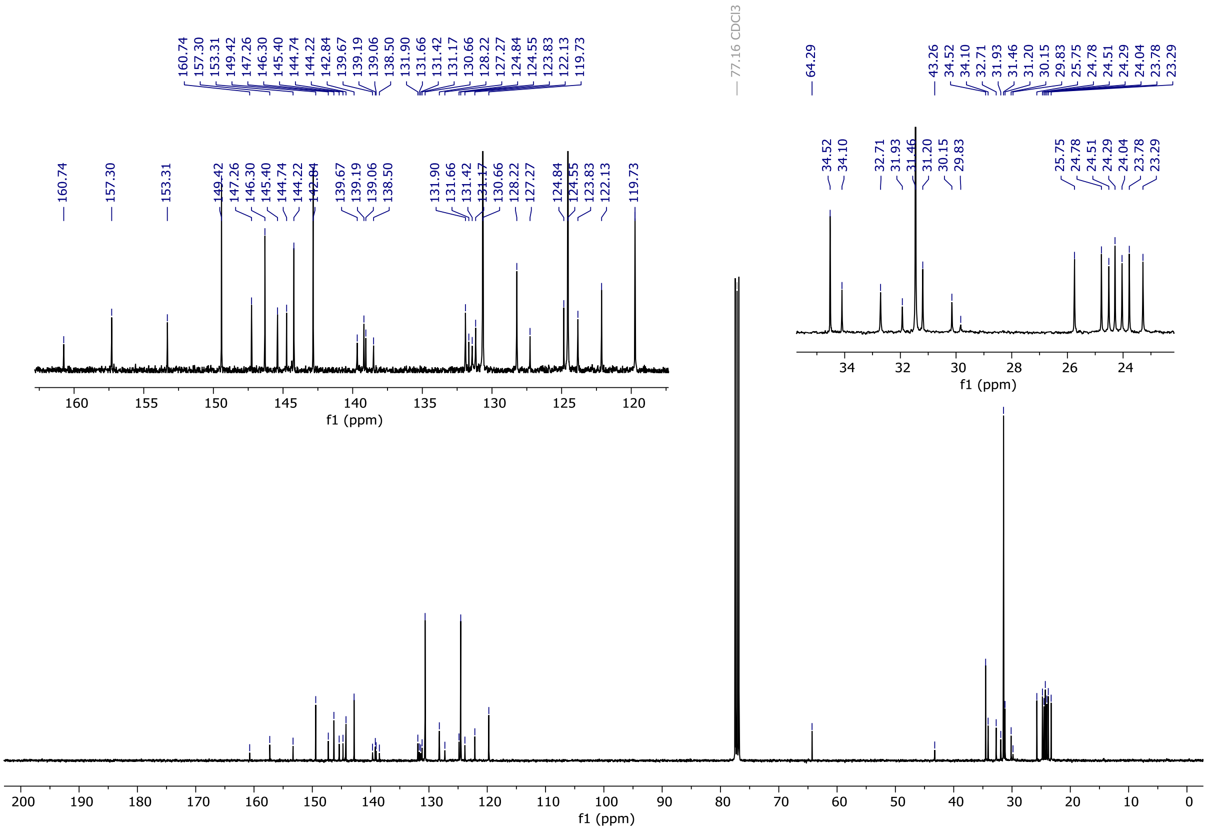

^{1}H NMR (500 MHz, $C_6D_6/CDCl_3$ 9:1) of **7**

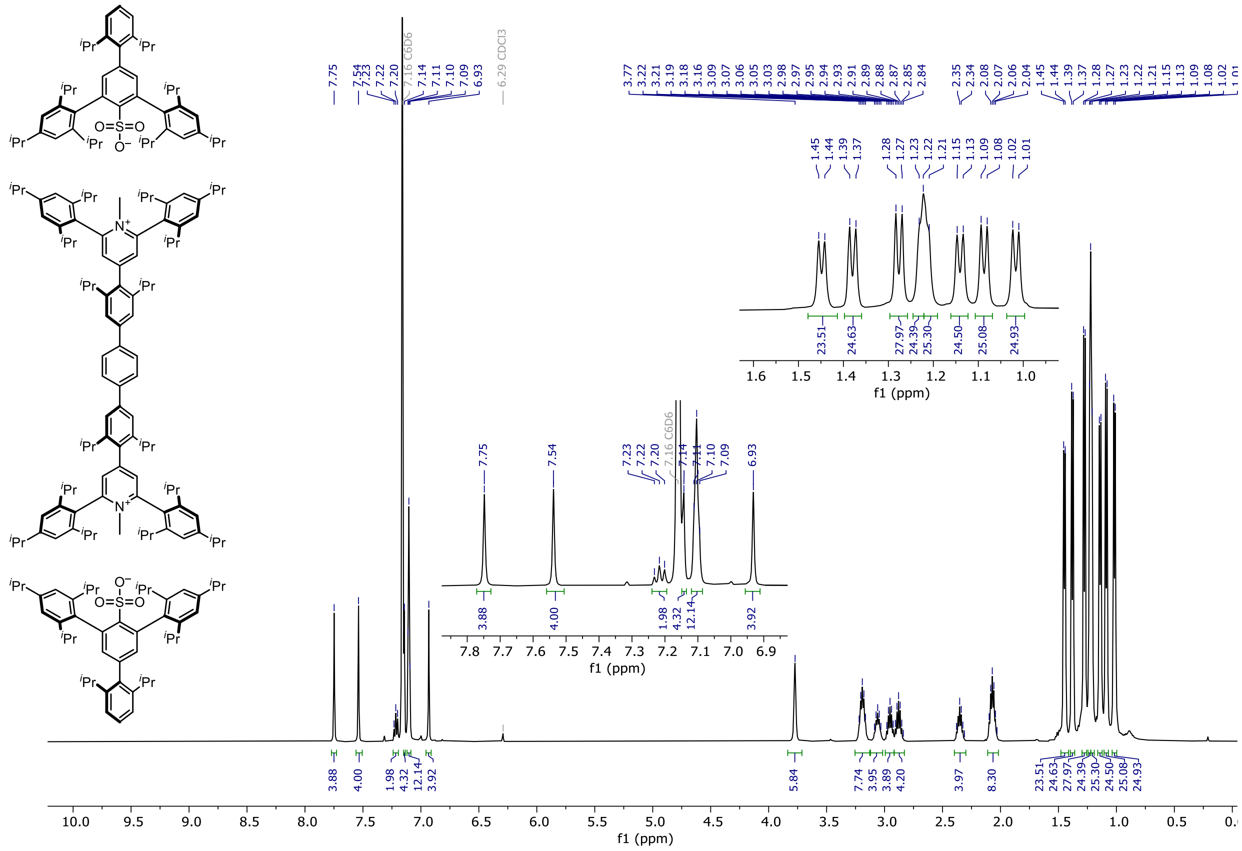

^{13}C NMR (126 MHz, $C_6D_6/CDCl_3$ 9:1) of **7**

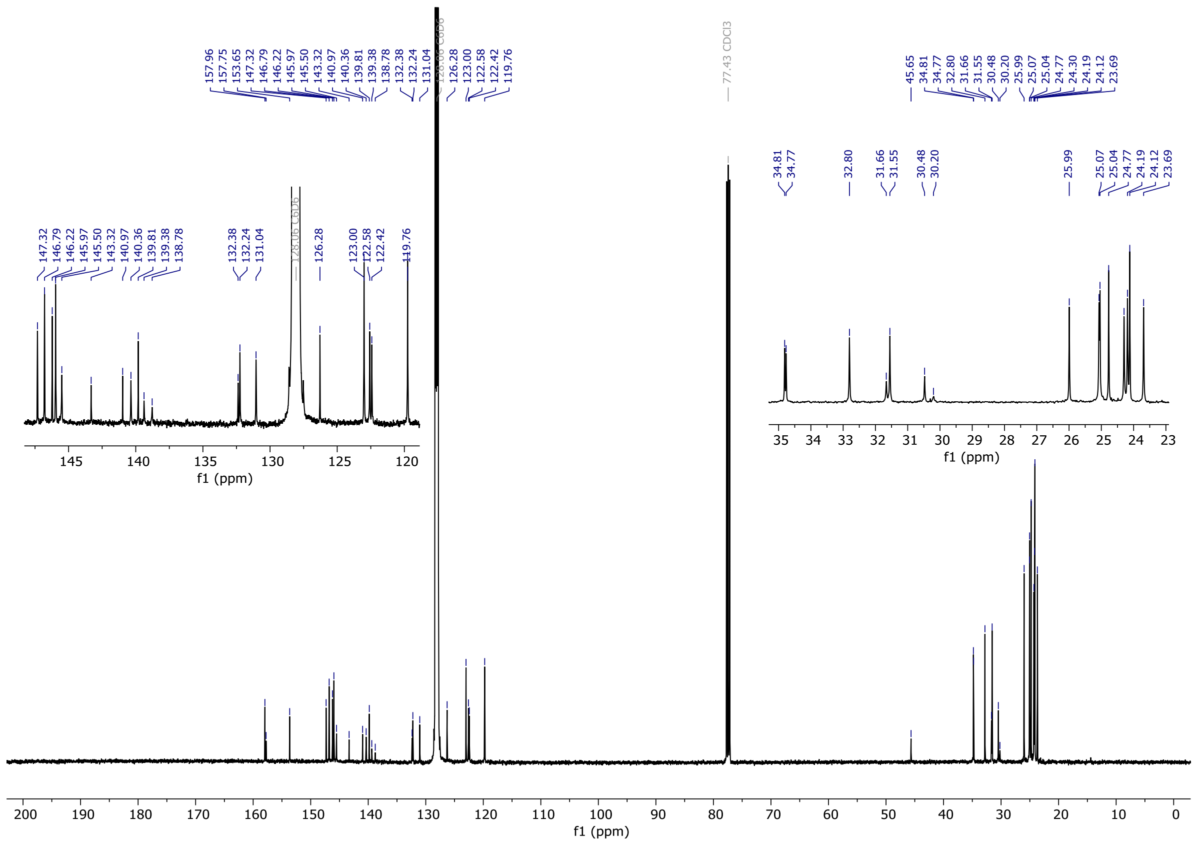

COSY ($C_6D_6/CDCl_3$ 9:1) of **7**

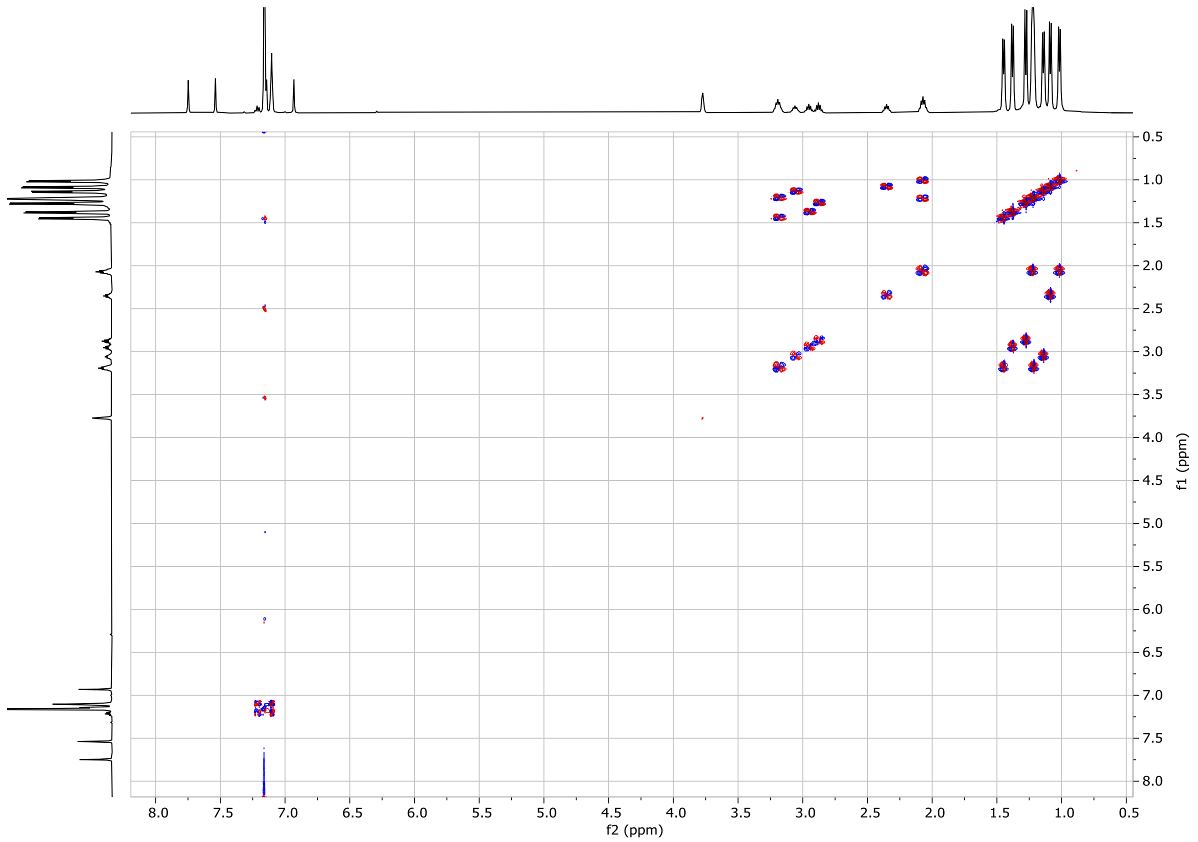

Excerpt from COSY ($C_6D_6/CDCl_3$ 9:1) of **7**

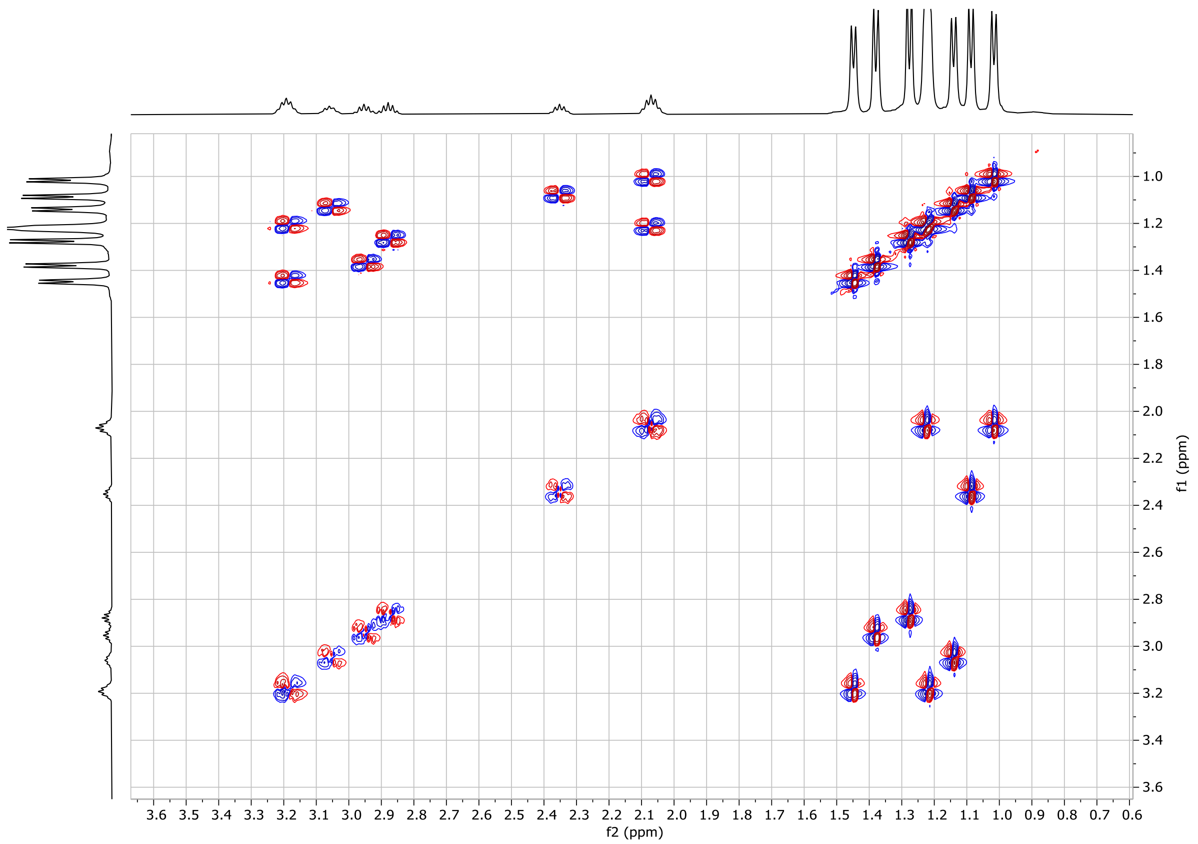

ROESY ($C_6D_6/CDCl_3$ 9:1) of **7**

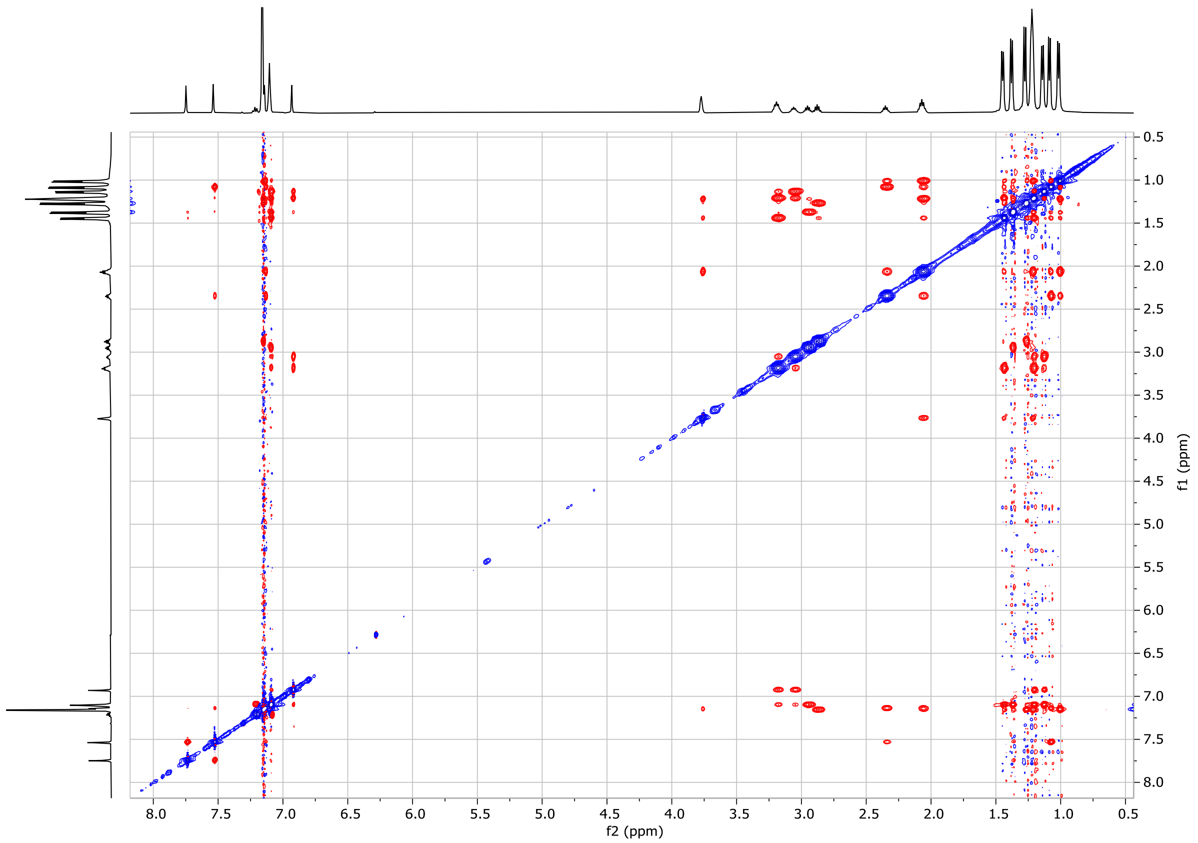

Excerpt from ROESY ($C_6D_6/CDCl_3$ 9:1) of **7**

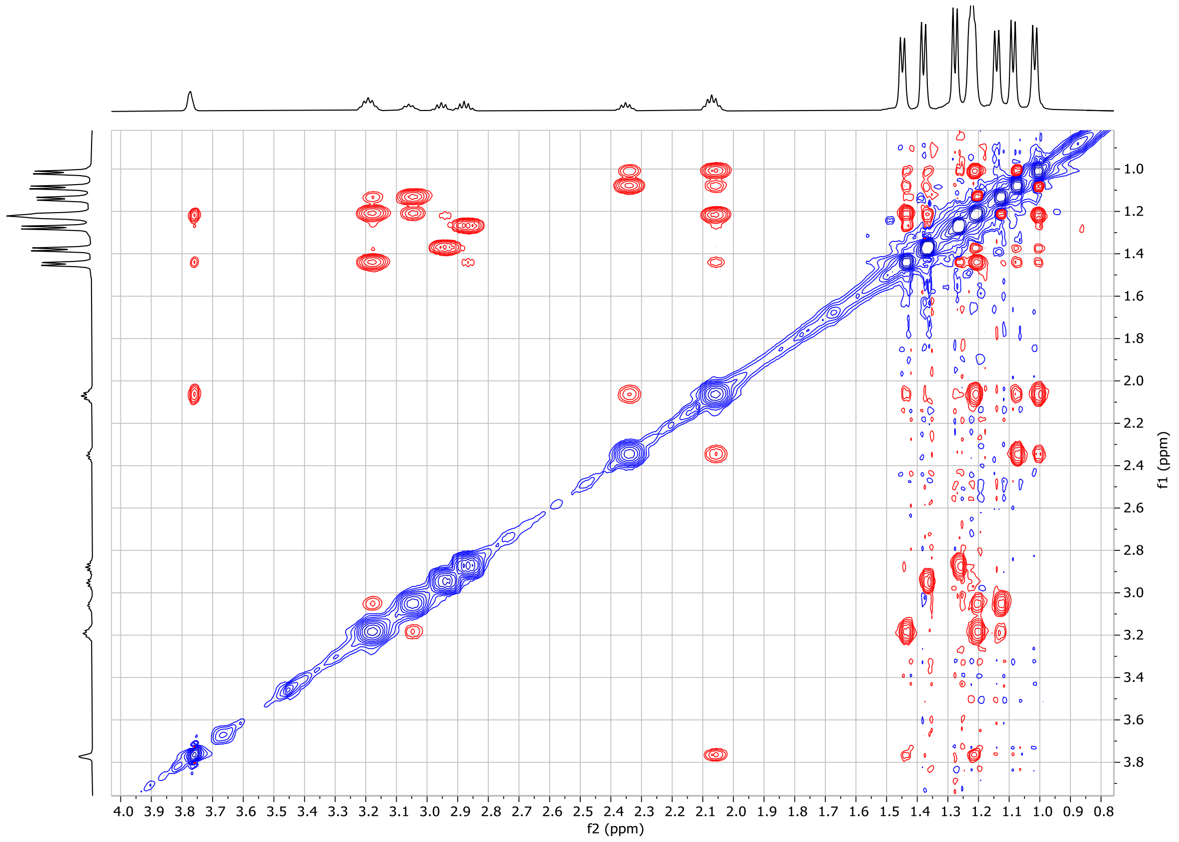

Excerpt from ROESY (C_6D_6/$CDCl_3$ 9:1) of **7**

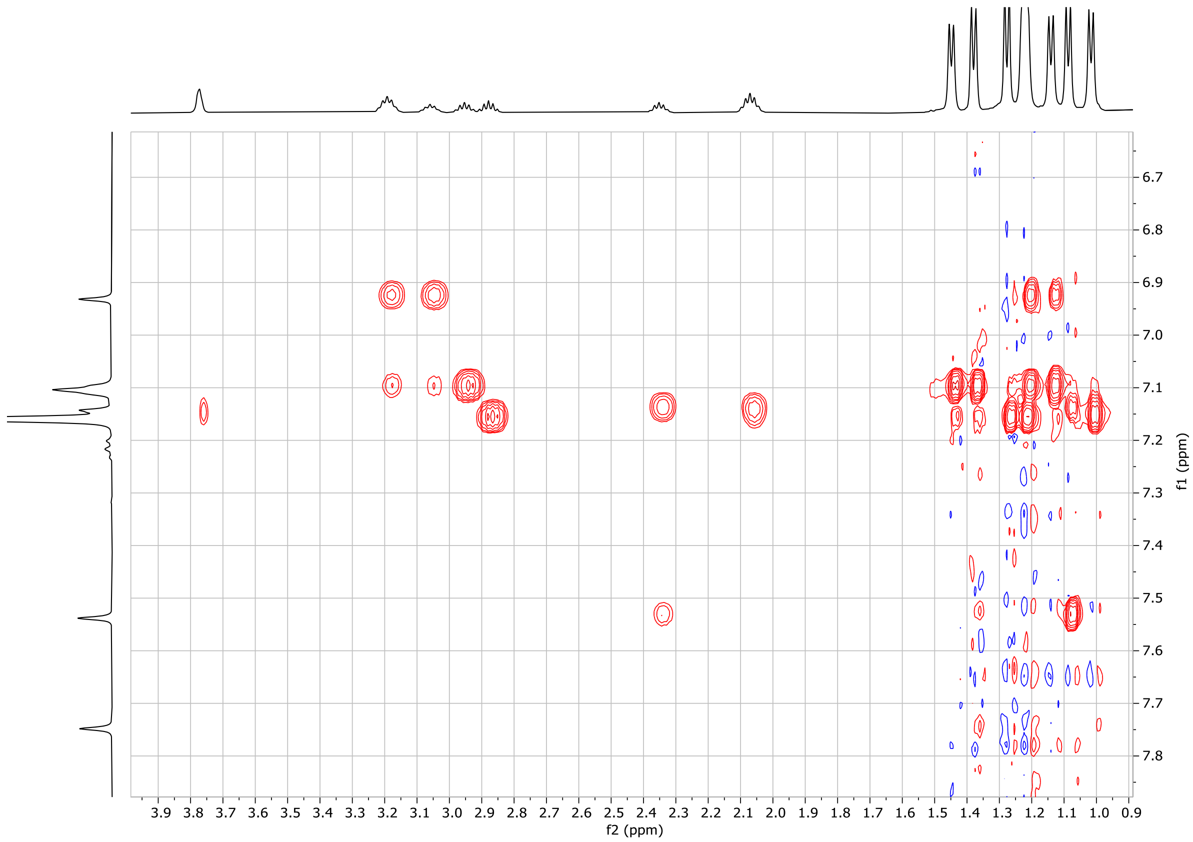

^{1}H NMR (500 MHz, C_6D_6) of **8**

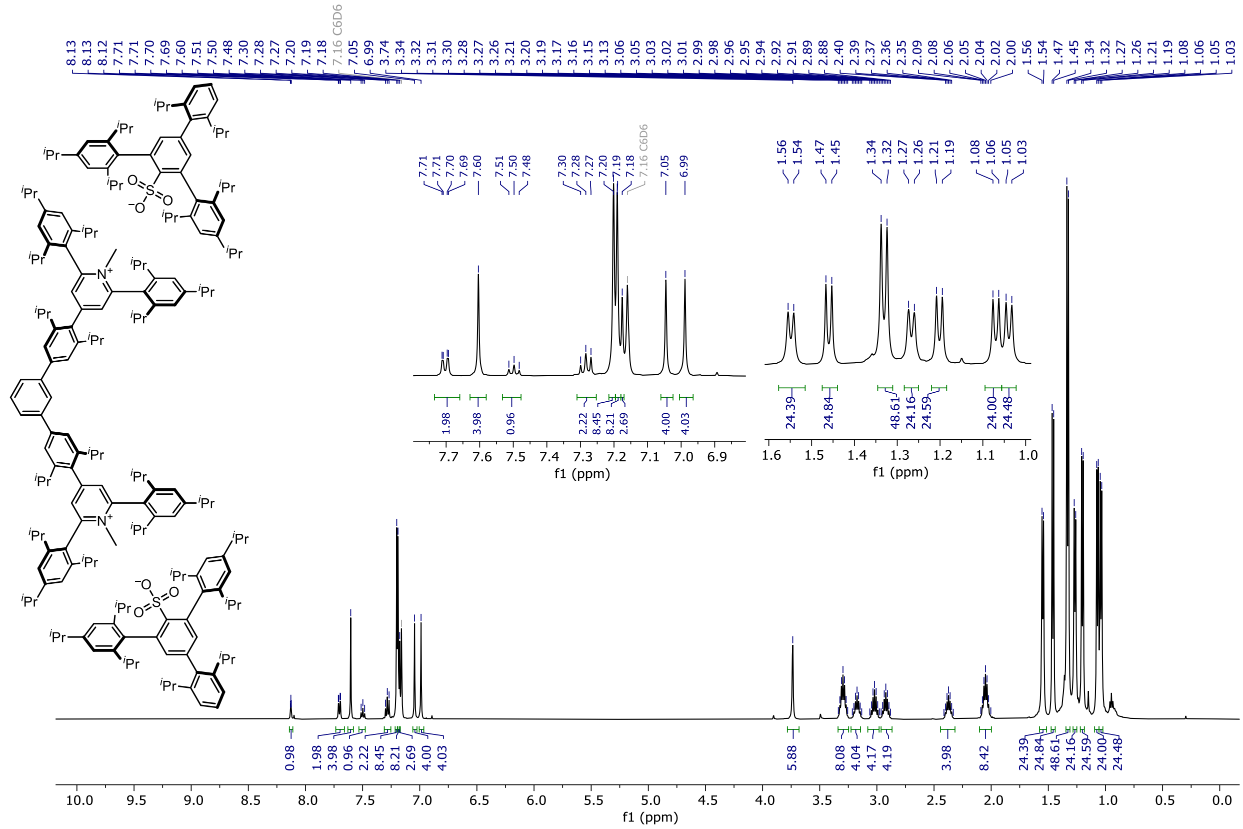

^{13}C NMR (126 MHz, C_6D_6) of **8**

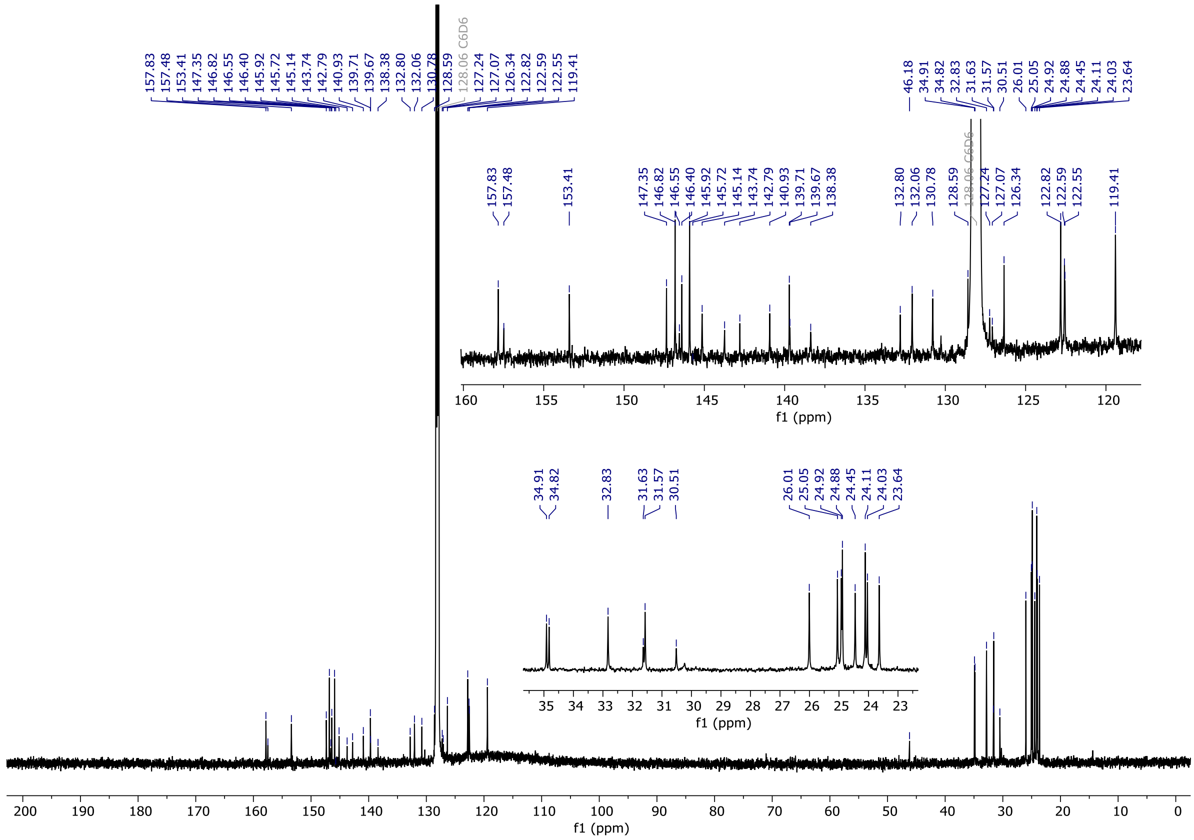

COSY (C_6D_6) of **8**

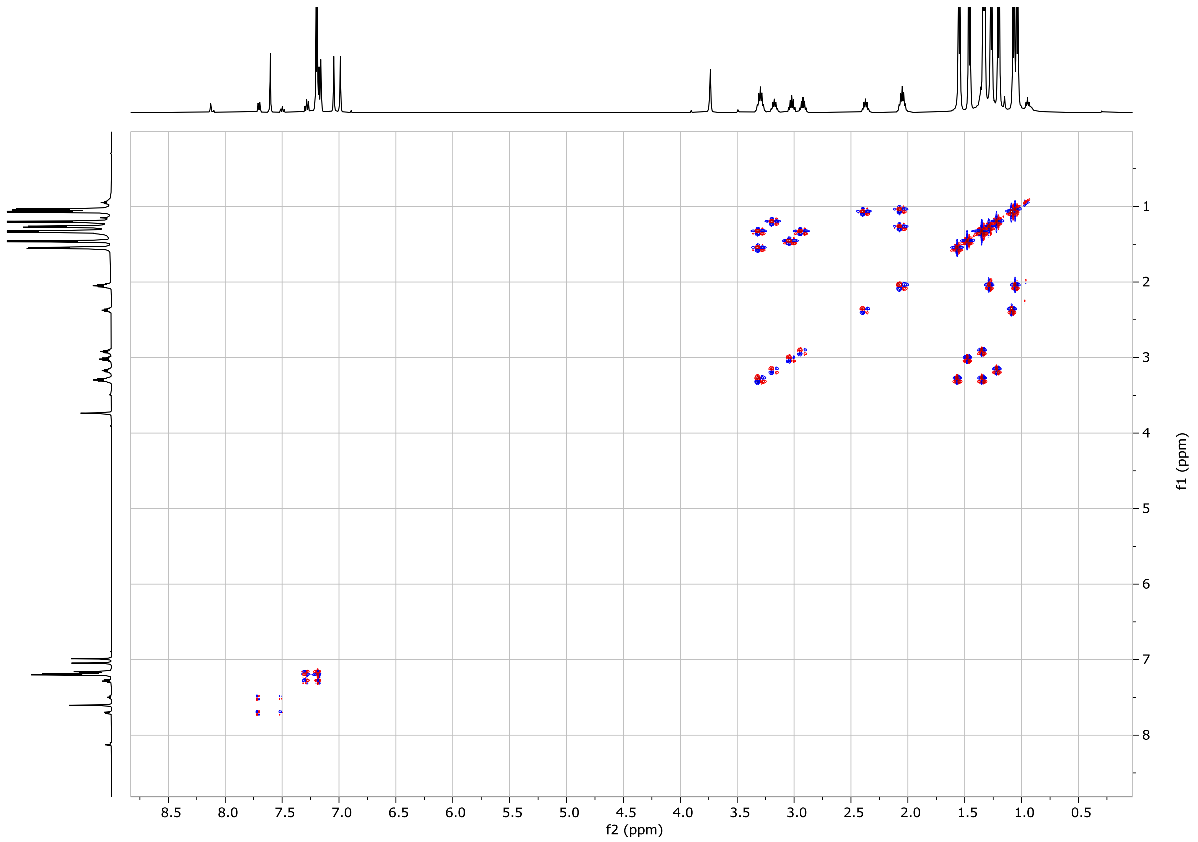

Excerpt from COSY (C_6D_6) of **8**

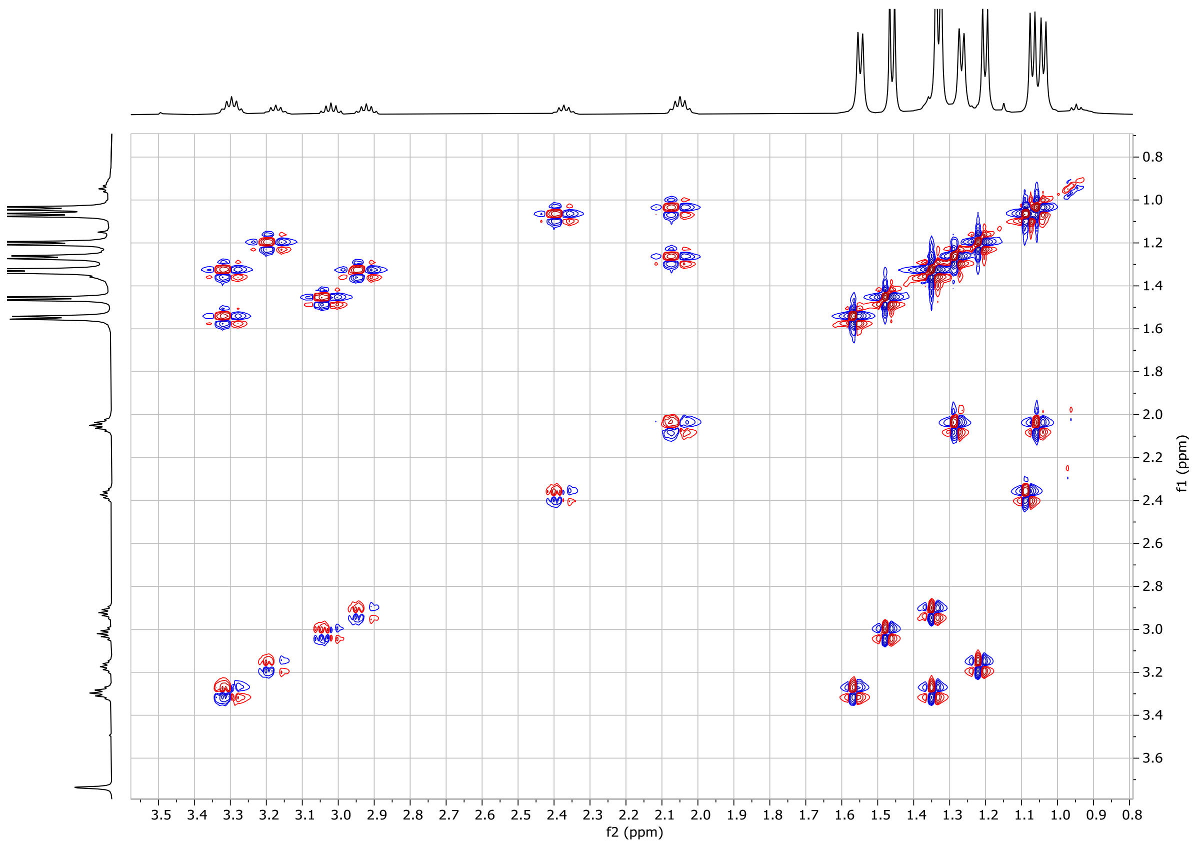

ROESY (C_6D_6) of **8**

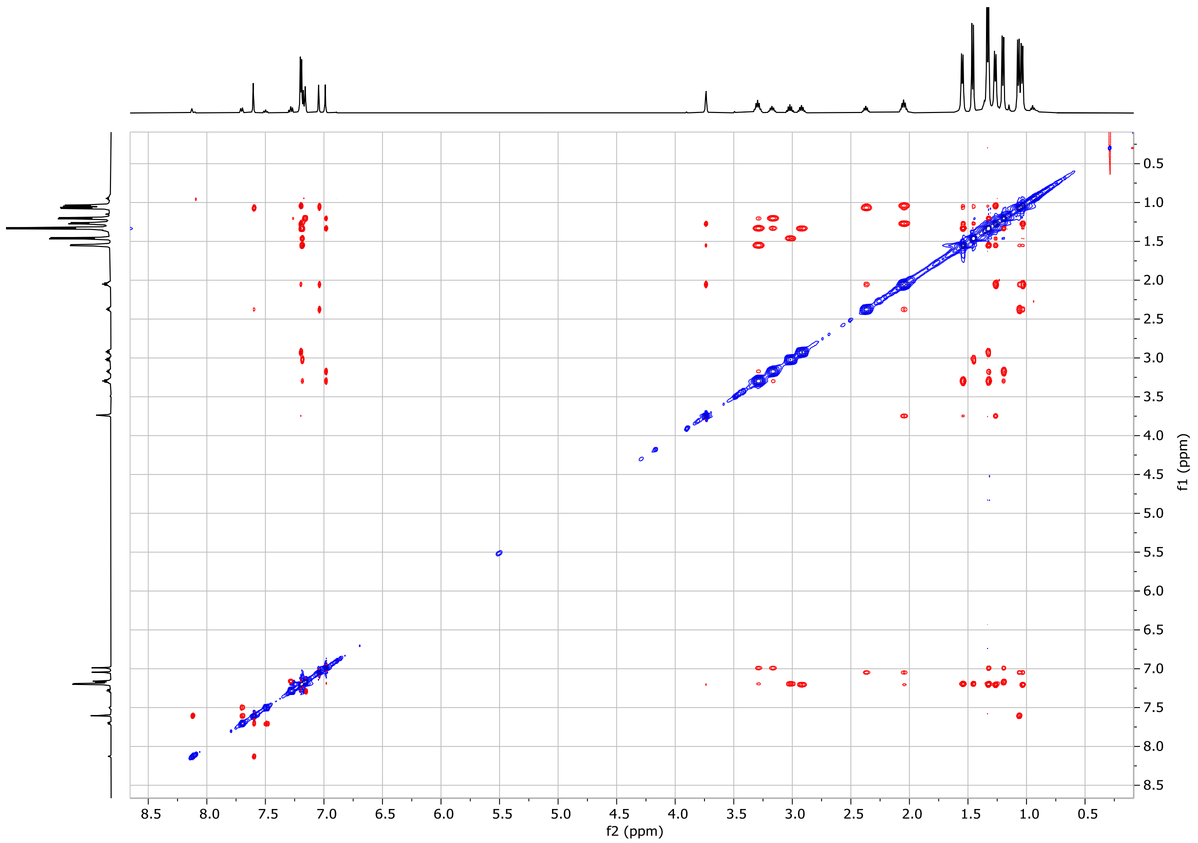

Excerpt from ROESY (C_6D_6) of **8**

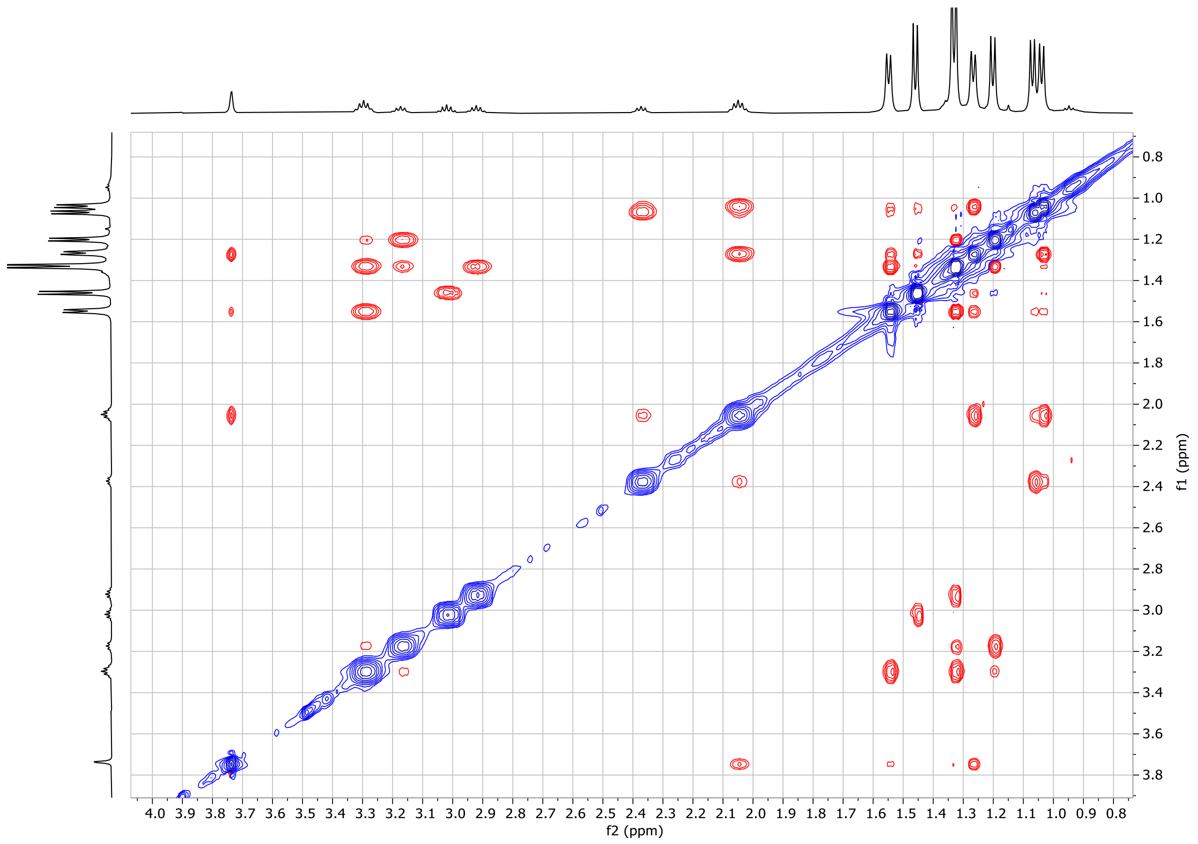

Excerpt from ROESY (C_6D_6) of **8**

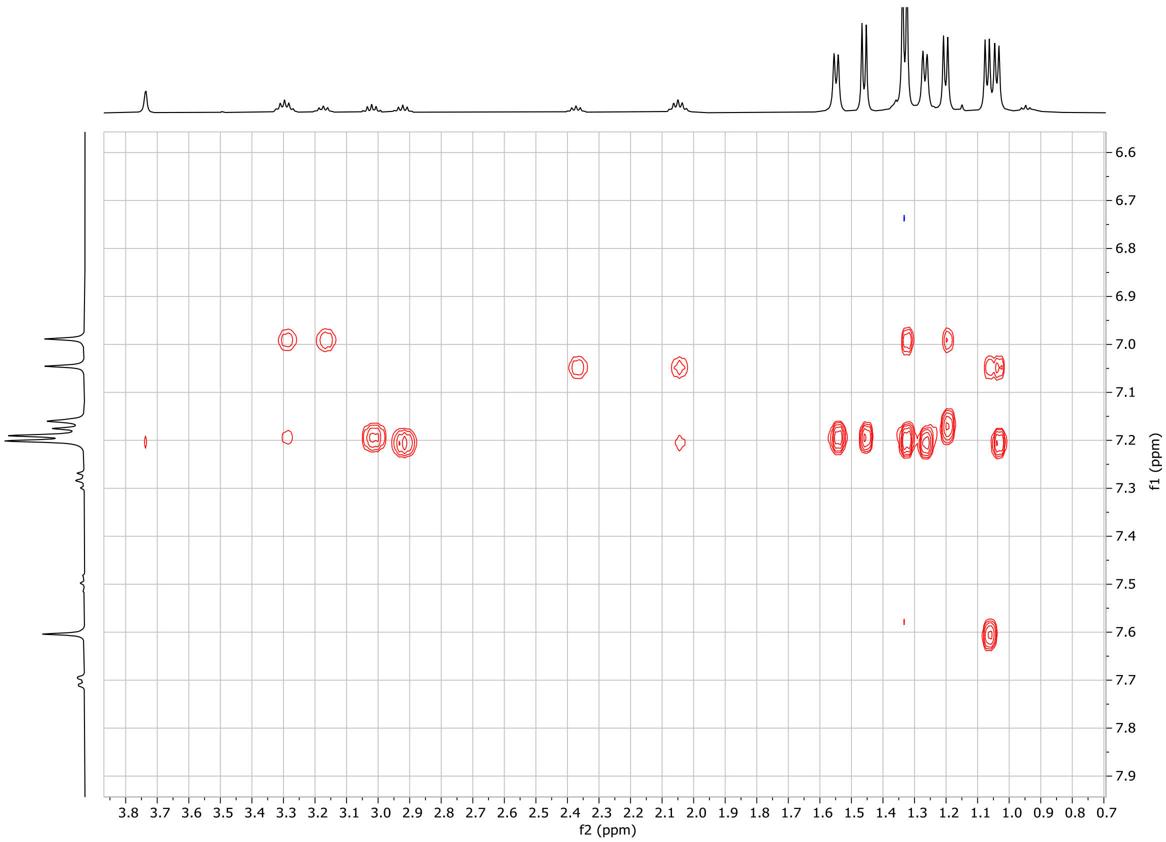

^{1}H NMR (500 MHz, C_6D_6) of **9**

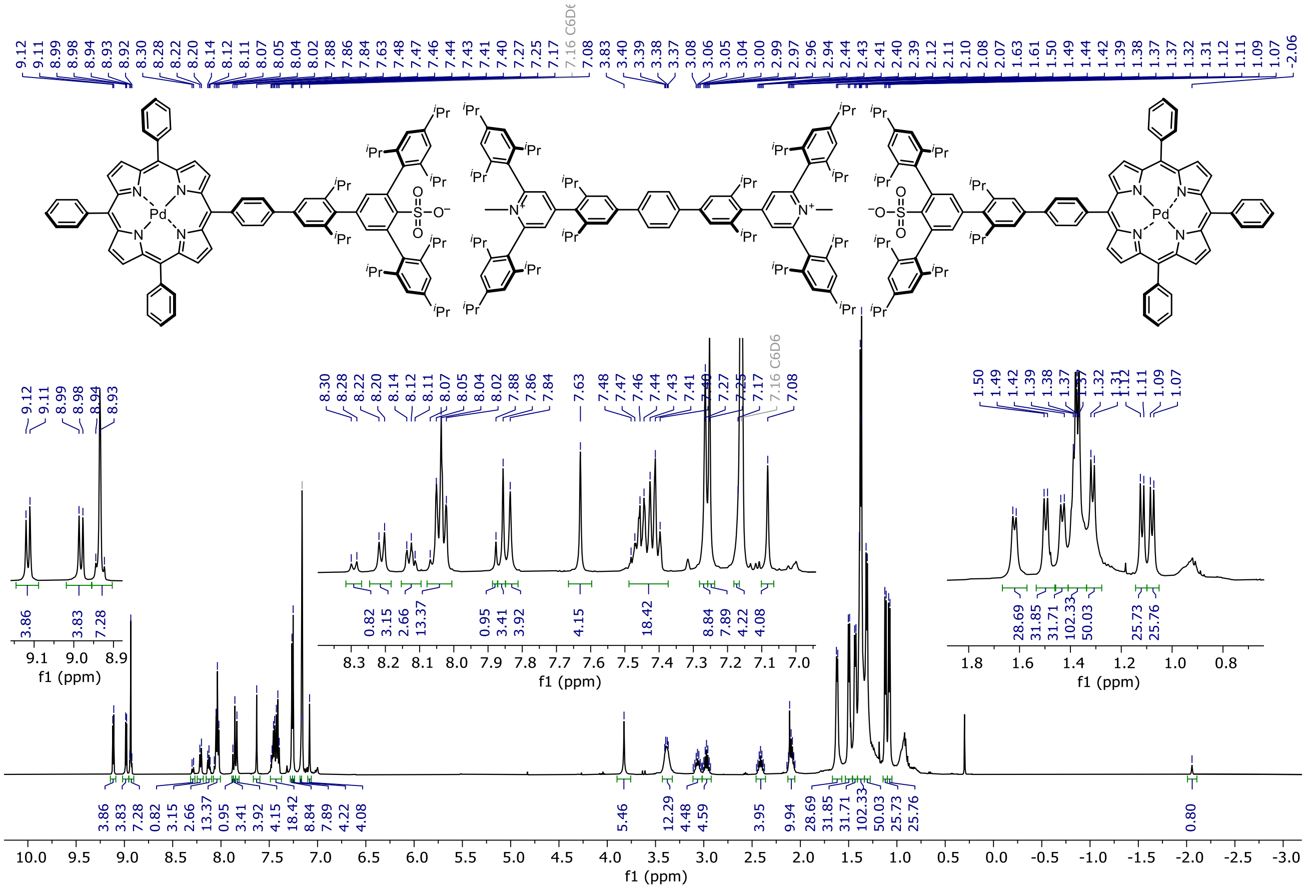

^{13}C NMR (126 MHz, C_6D_6) of **9**

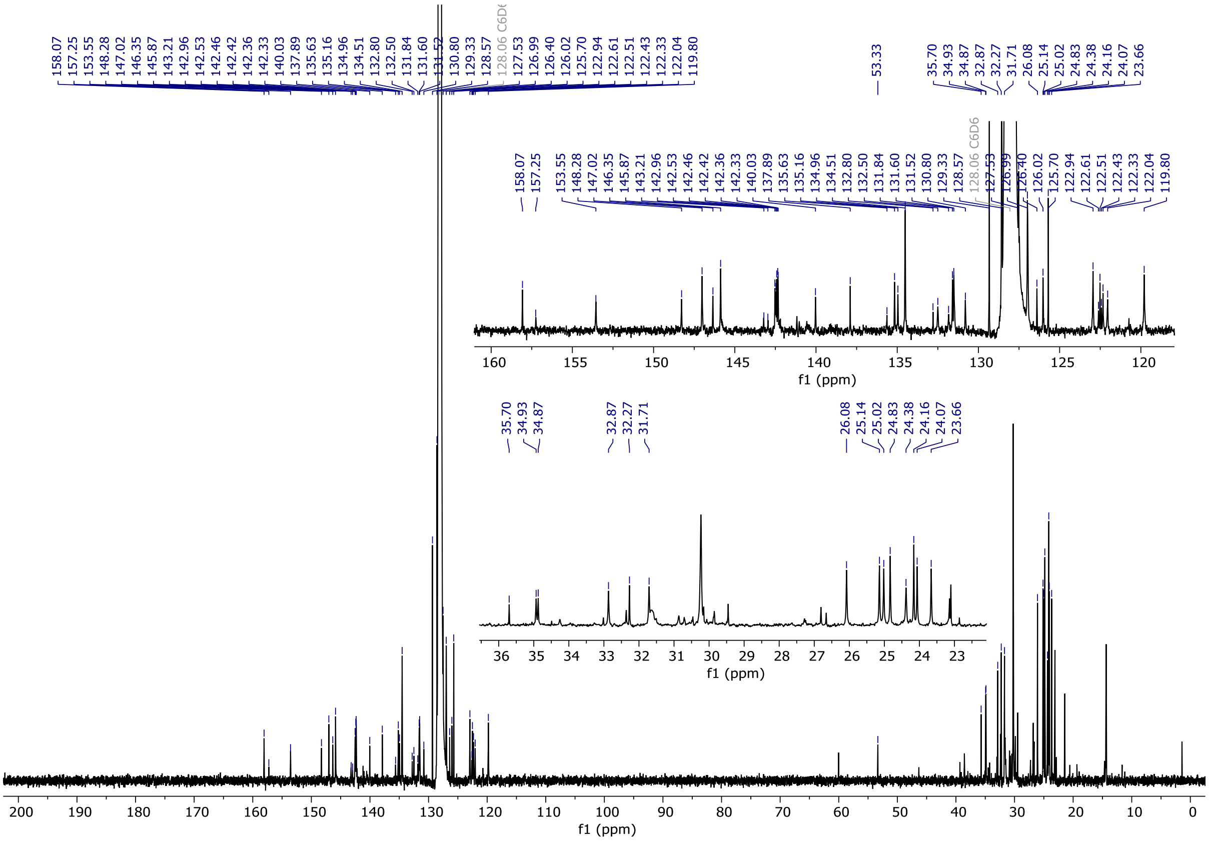

^{1}H NMR (500 MHz, CD_3OD-$CDCl_3$) of **10**

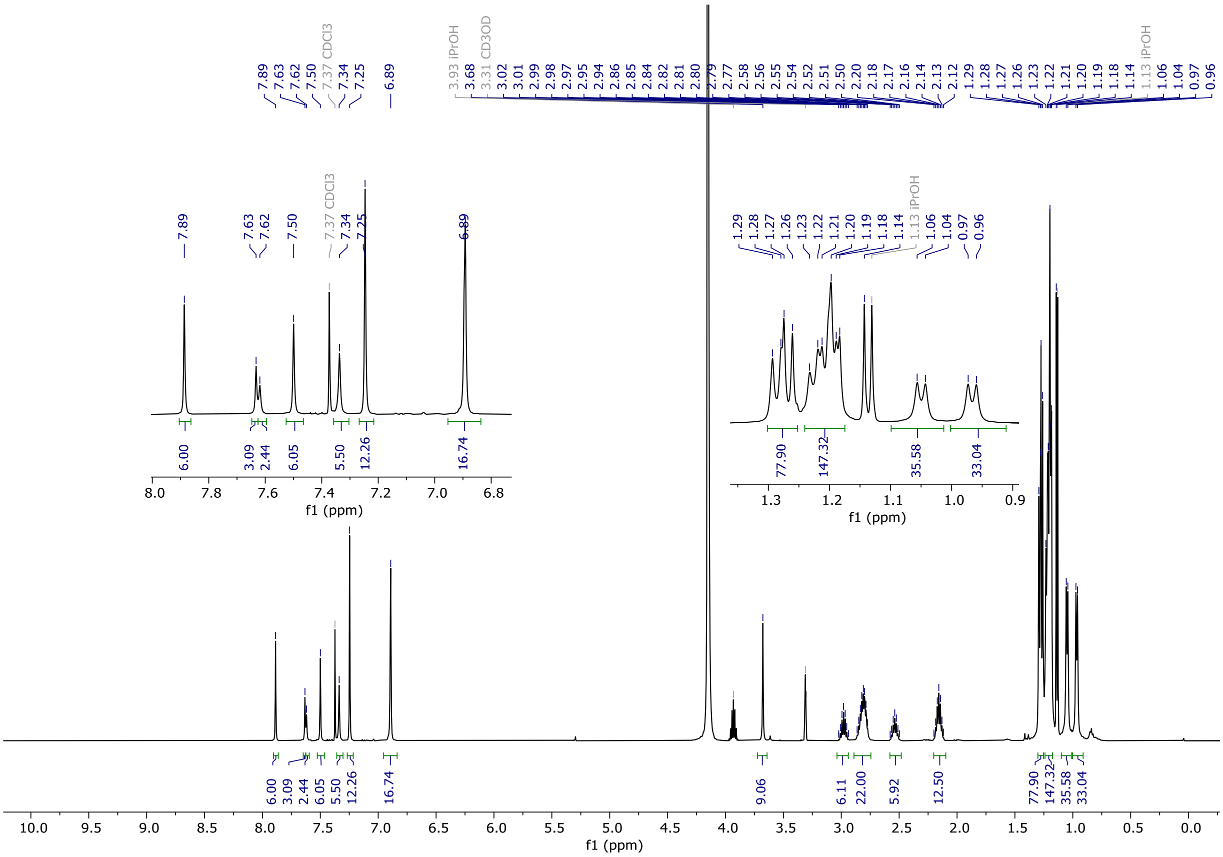

^{13}C NMR (126 MHz, CD_3OD-$CDCl_3$) of **10**

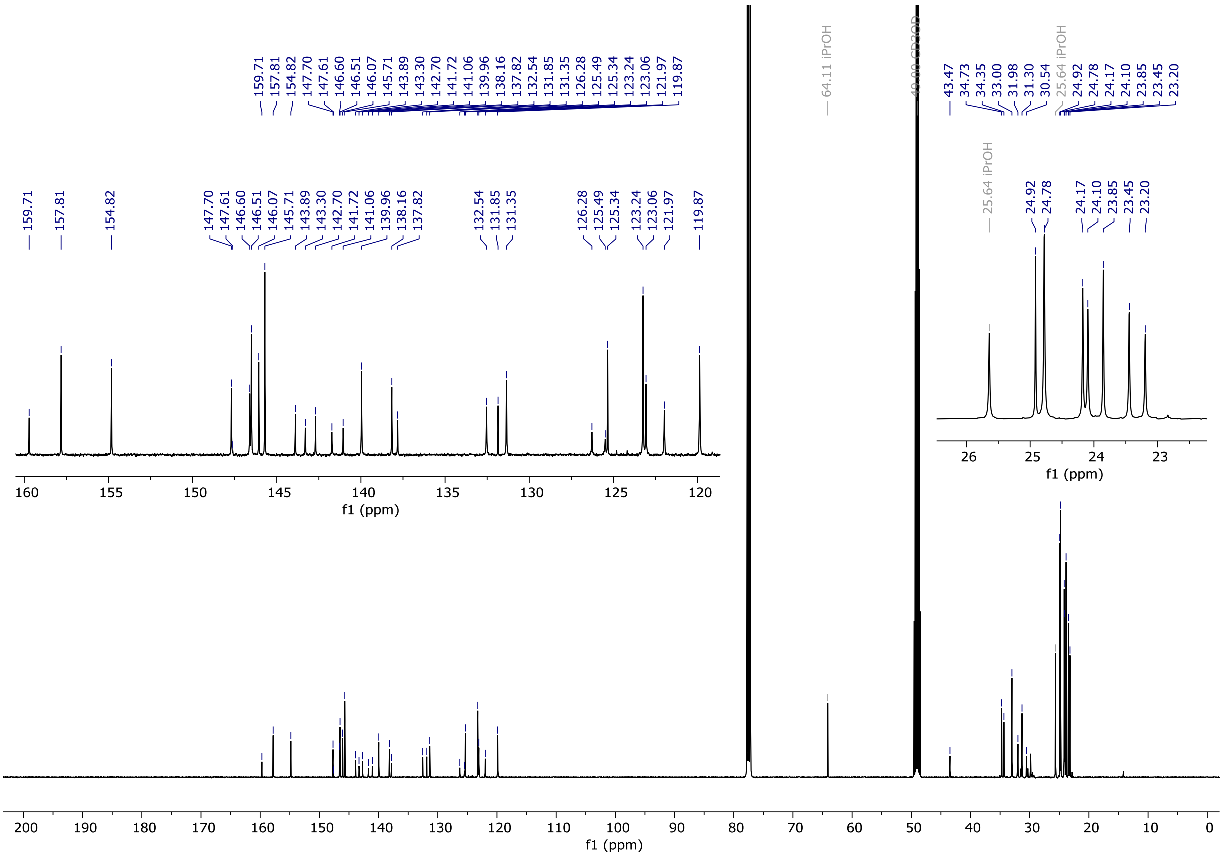

3.2 Cartesian coordinates of DFT and PM6 optimized structures

3.2.1 Structures optimized at the M06-2X/6-31+G(d,p) level of theory

C1⁺

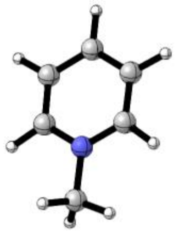

C	-6.3439050	1.3676860	0.0000000
C	-7.4919100	0.5795590	0.0000000
C	-7.3642430	-0.7983170	0.0000000
N	-6.1457410	-1.3752080	0.0000000
C	-5.0211830	-0.6270510	0.0000000
C	-5.0918970	0.7522390	0.0000000
C	-6.0150530	-2.8498230	0.0000000
H	-7.0085210	-3.2928030	0.0000000
H	-5.4743720	-3.1566500	0.8956160
H	-5.4743720	-3.1566500	-0.8956160
H	-8.2159600	-1.4680330	0.0000000
H	-6.4221380	2.4497300	0.0000000
H	-8.4830240	1.0169810	0.0000000
H	-4.0839510	-1.1726680	0.0000000
H	-4.1745060	1.3284920	0.0000000

A1⁻

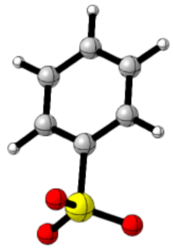

C	-4.7216190	2.4671030	0.1780940
C	-5.9045810	1.7314480	0.0935120
C	-5.8585710	0.3447070	-0.0466120
C	-4.6280510	-0.3054260	-0.1024810
C	-3.4439730	0.4239030	-0.0235380
C	-3.4915500	1.8105090	0.1165690
S	-4.5675460	-2.1141130	-0.2088500
O	-5.7951400	-2.4708000	-0.9504310
O	-3.3062030	-2.3888080	-0.9283170
O	-4.5656190	-2.5528030	1.2026120
H	-2.5013430	-0.1112750	-0.0899660
H	-2.5683520	2.3815300	0.1740250
H	-4.7580560	3.5481020	0.2850890
H	-6.8641520	2.2406910	0.1329680
H	-6.7627080	-0.2509900	-0.1306530

C2$^+$

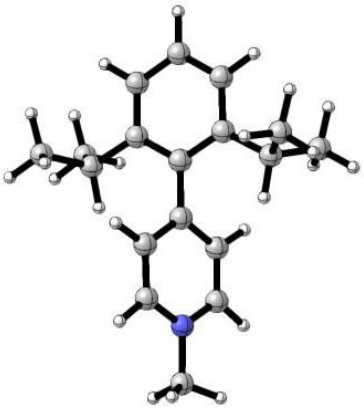

N	4.8644450	20.3881630	18.0126840
C	3.7712730	20.6654590	17.0584340
H	3.6324100	19.7962540	16.4171560
H	4.0435980	21.5245170	16.4480430
H	2.8549590	20.8752070	17.6123680
C	5.6980990	21.3797270	18.3937080
H	5.5290730	22.3502370	17.9417100
C	6.6999840	21.1480540	19.3124150
H	7.3533070	21.9643360	19.6008240
C	6.8677250	19.8726160	19.8735980
C	5.9791580	18.8681970	19.4563890
H	6.0666650	17.8610620	19.8492890
C	4.9959600	19.1476880	18.5320980
H	4.2933990	18.4024000	18.1766370
C	7.9399410	19.5961000	20.8631460
C	9.2909760	19.6930220	20.4609660
C	10.2780710	19.4295630	21.4122390
H	11.3238330	19.4904970	21.1263110
C	9.9464760	19.0790380	22.7154120
H	10.7299440	18.8772540	23.4386180
C	8.6123400	18.9865800	23.0930860
H	8.3651570	18.7197010	24.1162020
C	7.5842850	19.2422190	22.1840270
C	9.7217560	20.0223860	19.0376750
H	8.8317370	20.0991150	18.4039820
C	10.5881120	18.9031330	18.4429990
H	10.8197750	19.1224570	17.3968810
H	10.0779360	17.9369450	18.4899270
H	11.5369860	18.8084840	18.9787150
C	10.4570980	21.3681170	18.9792860
H	11.3805590	21.3308740	19.5652180
H	9.8482410	22.1840960	19.3818170
H	10.7256300	21.6127150	17.9475530
C	6.1443450	19.1718400	22.6752810
H	5.4762180	19.4954380	21.8699800
C	5.9080770	20.1289320	23.8521710
H	4.8523330	20.1206720	24.1376150
H	6.1896980	21.1538500	23.5947950
H	6.4898250	19.8307640	24.7290850
C	5.7590210	17.7360680	23.0563650
H	6.3716530	17.3785590	23.8896390
H	5.9027880	17.0399440	22.2237170
H	4.7116340	17.6912210	23.3684390

A2⁻

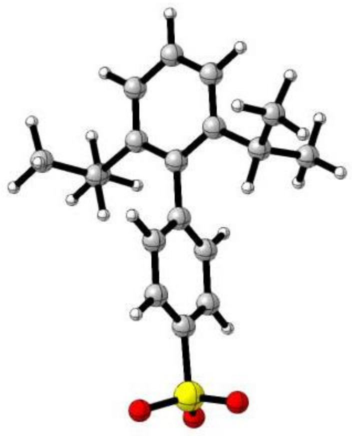

S	19.0322430	12.5889170	16.6507380
O	18.8100310	11.8558580	17.9138820
O	17.9259280	12.4996040	15.6758660
O	20.3741470	12.3925650	16.0643690
C	19.0305770	14.3306400	17.1526000
C	18.6191450	15.3125110	16.2557860
H	18.2455310	15.0035070	15.2839790
C	18.6726900	16.6567430	16.6236320
H	18.3413730	17.4279280	15.9307760
C	19.1368400	17.0314630	17.8887980
C	19.5379850	16.0334260	18.7842340
H	19.8881710	16.3203400	19.7738430
C	19.4858670	14.6893950	18.4191080
H	19.7784300	13.9017080	19.1071830
C	19.1830220	18.4736540	18.2853650
C	18.0526720	19.0755970	18.8716660
C	18.1083990	20.4284300	19.2236990
H	17.2394300	20.9005910	19.6764410
C	19.2580370	21.1772170	19.0031570
H	19.2870380	22.2276060	19.2795660
C	20.3741700	20.5754420	18.4291380
H	21.2702670	21.1663410	18.2598970
C	20.3572640	19.2257860	18.0678560
C	16.7749320	18.2923710	19.1325990
H	16.9372460	17.2620930	18.8051820
C	15.6025980	18.8572000	18.3213370
H	15.3771290	19.8884560	18.6171090
H	14.7023820	18.2546490	18.4807680
H	15.8324690	18.8541660	17.2516680
C	16.4402220	18.2550210	20.6286560
H	17.2694560	17.8302890	21.2022640
H	15.5508260	17.6411780	20.8047420
H	16.2391740	19.2604360	21.0163870
C	21.5726820	18.6004250	17.3996770
H	21.4902300	17.5149830	17.5083250
C	21.5705630	18.9162630	15.8975270
H	21.6278680	19.9994240	15.7352360
H	20.6576810	18.5460920	15.4226700
H	22.4273040	18.4459670	15.4035390
C	22.8973210	19.0312230	18.0377240
H	23.7207420	18.4495670	17.6115730
H	22.8876460	18.8712970	19.1201370
H	23.1142700	20.0890310	17.8514000

C3⁺

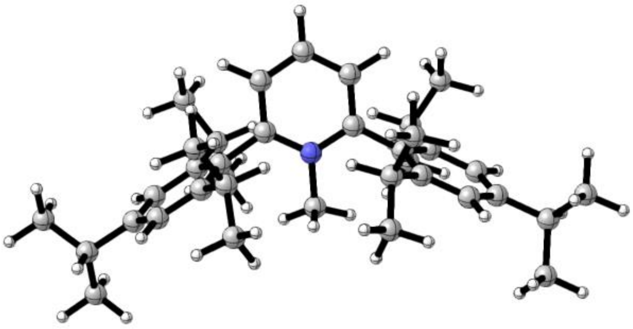

N	0.0007150	0.2062710	0.5259040
C	-1.1937310	0.2509860	1.1845120
C	-1.2052710	0.5135480	2.5480030
H	-2.1654280	0.5470120	3.0499100
C	-0.0119950	0.7112560	3.2327470
H	-0.0170710	0.9138240	4.2988780
C	1.1882430	0.6309020	2.5372690
H	2.1450120	0.7605330	3.0301190
C	1.1909300	0.3695840	1.1728330
C	0.0042010	-0.0820570	-0.9269590
H	0.0211840	-1.1671980	-1.0686300
H	0.8927140	0.3592980	-1.3706480
H	-0.8998370	0.3291100	-1.3689420
C	-2.4549240	-0.0019930	0.4355940
C	-2.8643230	-1.3299920	0.2234770
C	-4.0775370	-1.5428100	-0.4336050
H	-4.4222270	-2.5608250	-0.5978230
C	-4.8717160	-0.4867850	-0.8817680
C	-4.4227160	0.8192630	-0.6672730
H	-5.0287150	1.6510470	-1.0187800
C	-3.2202050	1.0898610	-0.0163340
C	-2.0546440	-2.5134410	0.7332230
H	-1.0549550	-2.1561720	1.0164510
C	-2.7027660	-3.0962800	1.9969440
H	-2.7988140	-2.3387310	2.7809970
H	-2.1066530	-3.9257980	2.3883920
H	-3.7053880	-3.4733870	1.7718930
C	-1.8603640	-3.5982930	-0.3323380
H	-2.8076590	-4.0803060	-0.5900340
H	-1.1904990	-4.3768870	0.0436610
H	-1.4323610	-3.1907060	-1.2539640
C	-6.1877380	-0.7484370	-1.5873610
H	-6.3195490	-1.8362490	-1.6339170
C	-6.1652450	-0.2112630	-3.0238190
H	-6.0629910	0.8792220	-3.0325430
H	-7.0983670	-0.4612280	-3.5363090
H	-5.3347030	-0.6349380	-3.5953730
C	-7.3656910	-0.1607490	-0.8001180
H	-7.3916750	-0.5431040	0.2240020
H	-8.3111680	-0.4155120	-1.2869380
H	-7.2994990	0.9315240	-0.7536430
C	-2.7720370	2.5303490	0.1854150
H	-1.7415430	2.5242110	0.5646400
C	-2.7615620	3.3185510	-1.1305300
H	-2.1572830	2.8198600	-1.8946580
H	-2.3504820	4.3185820	-0.9661640
H	-3.7725160	3.4402660	-1.5301690
C	-3.6476410	3.2294170	1.2339750
H	-4.6888110	3.2697890	0.8982970
H	-3.3052340	4.2549740	1.3990240
H	-3.6280440	2.7001530	2.1916020
C	2.4651890	0.2516300	0.4134660
C	2.9887340	-1.0311730	0.1526260
C	4.2075640	-1.1162740	-0.5149270
H	4.6326040	-2.0971160	-0.7130140
C	4.9056590	0.0227820	-0.9311300
C	4.3485600	1.2728980	-0.6689380
H	4.8814640	2.1622760	-0.9975080
C	3.1294800	1.4177920	-0.0006500
C	2.2888630	-2.2958750	0.6297980
H	1.2573970	-2.0414790	0.9099760
C	2.9784550	-2.8404170	1.8888590
H	4.0124880	-3.1209180	1.6646120
H	2.4573090	-3.7272590	2.2612420
H	3.0009620	-2.0913590	2.6865870
C	2.2063100	-3.3723630	-0.4581140
H	1.7547100	-2.9865790	-1.3777640
H	1.6055070	-4.2157220	-0.1060350
H	3.1963700	-3.7622520	-0.7111310
C	6.2344080	-0.1028840	-1.6496820
H	6.5763660	0.9138380	-1.8780250
C	6.0827310	-0.8612690	-2.9741380
H	5.7697670	-1.8963410	-2.7998560
H	7.0378090	-0.8889390	-3.5059880
H	5.3413950	-0.3867460	-3.6231270
C	7.2872720	-0.7675430	-0.7538040
H	7.4070010	-0.2272530	0.1893040
H	8.2554400	-0.7925820	-1.2615770
H	7.0072870	-1.8004670	-0.5206400
C	2.5613530	2.8093120	0.2355270
H	1.5670180	2.7086470	0.6887680
C	2.3803610	3.5755930	-1.0815410
H	3.3428180	3.7665280	-1.5652080
H	1.9064690	4.5430520	-0.8927330
H	1.7556740	3.0194570	-1.7876570
C	3.4374750	3.6006180	1.2150520
H	3.5432150	3.0774130	2.1703180
H	3.0034350	4.5860080	1.4070130
H	4.4409600	3.7488700	0.8040190

A3⁻

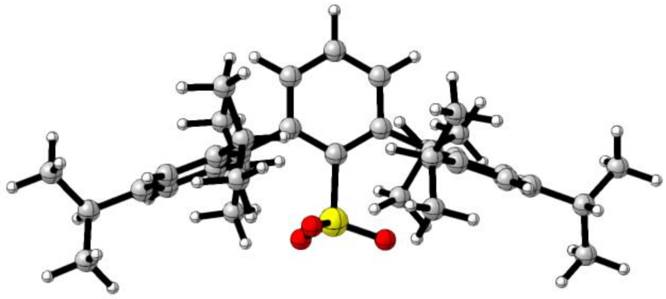

S	-0.0730870	-0.1807470	-1.3615250
O	1.3160240	-0.2196890	-1.8488940
O	-0.7886190	-1.4678690	-1.4913600
O	-0.8318850	0.9998580	-1.8199260
C	0.0266700	0.0427120	0.4519840
C	-1.1879510	0.1739870	1.1563980
C	-1.1653640	0.2916370	2.5479510
C	0.0365320	0.2663640	3.2504730
C	1.2266830	0.1346310	2.5501960
C	1.2437200	0.0313180	1.1499570
C	-2.5405890	0.1945650	0.5046950
C	-3.2680850	-1.0012870	0.3719710
C	-4.5730190	-0.9432100	-0.1197020
C	-5.1651670	0.2636960	-0.4919490
C	-4.4133540	1.4305740	-0.3747660
C	-3.1034730	1.4188740	0.1104660
C	-2.6698430	-2.3409580	0.7721490
C	-2.9045460	-3.4246840	-0.2840980
C	-3.2051000	-2.7824030	2.1412510
C	-6.5799430	0.3035710	-1.0396220
C	-6.6856630	-0.4580170	-2.3667120
C	-7.5981010	-0.2324460	-0.0259520
C	-2.3226920	2.7187290	0.2190660
C	-2.4399240	3.5797960	-1.0416540
C	-2.7545650	3.5040450	1.4651180
C	2.6056070	-0.0889940	0.5295980
C	3.1141570	-1.3509980	0.1920100
C	4.4306190	-1.4456080	-0.2659450
C	5.2396920	-0.3235150	-0.4182550
C	4.7019810	0.9254140	-0.1010610
C	3.3976300	1.0650820	0.3742260
C	2.2621260	-2.6067230	0.2800080
C	2.1936760	-3.3353260	-1.0669300
C	2.7607570	-3.5330580	1.3966490
C	6.6602700	-0.4531620	-0.9360530
C	6.8371000	0.2721720	-2.2757140
C	7.6861900	0.0461360	0.0886670
C	2.8116250	2.4486230	0.6204240
C	2.1147890	2.9577800	-0.6510570
C	3.8393830	3.4713740	1.1143630
H	-2.1107010	0.3964680	3.0748870
H	0.0420750	0.3485260	4.3336590
H	2.1776290	0.1161370	3.0773520
H	-5.1368790	-1.8683970	-0.2245740
H	-4.8589120	2.3741420	-0.6844550
H	-1.5867300	-2.2075590	0.8466500
H	-3.9648160	-3.6948030	-0.3609630
H	-2.3541060	-4.3320690	-0.0105800
H	-2.5431940	-3.0784840	-1.2547490
H	-4.2938000	-2.9119830	2.1047120
H	-2.9793150	-2.0398790	2.9134900
H	-2.7574480	-3.7370570	2.4408160
H	-6.8218140	1.3568360	-1.2352420
H	-6.4668100	-1.5211180	-2.2182360
H	-5.9688380	-0.0714310	-3.0961660
H	-7.6953960	-0.3745280	-2.7851870
H	-7.4077610	-1.2895320	0.1900270
H	-8.6187550	-0.1474030	-0.4163060
H	-7.5381890	0.3173130	0.9180720
H	-1.2650430	2.4582990	0.3224490
H	-3.4552590	3.9732230	-1.1723630
H	-2.1608210	2.9907950	-1.9180080
H	-1.7623860	4.4379530	-0.9667270
H	-3.8168610	3.7702290	1.4021170
H	-2.1753510	4.4301090	1.5576610
H	-2.6073580	2.9139420	2.3756120
H	4.8321830	-2.4228340	-0.5305650
H	5.3143640	1.8142830	-0.2360300
H	1.2383230	-2.3081400	0.5250290
H	3.1732430	-3.7393440	-1.3511730
H	1.8464960	-2.6469280	-1.8413310
H	1.4883110	-4.1710360	-1.0018990
H	3.7856900	-3.8675400	1.1954610
H	2.1232740	-4.4211040	1.4719160
H	2.7569520	-3.0210640	2.3649100
H	6.8460780	-1.5222050	-1.1052590
H	6.6833560	1.3499790	-2.1532380
H	6.1112860	-0.0850500	-3.0112120
H	7.8472350	0.1179370	-2.6724090
H	7.5487560	1.1159010	0.2817610
H	8.7083550	-0.1012780	-0.2785620
H	7.5776560	-0.4815500	1.0408850
H	2.0472570	2.3552470	1.3994660
H	2.8453390	3.0548570	-1.4629280
H	1.6633380	3.9406990	-0.4694350
H	1.3358000	2.2727120	-0.9966040
H	4.5529280	3.7395420	0.3271620
H	4.4051620	3.0936100	1.9727060
H	3.3297100	4.3929930	1.4143470

$C4^{+}$

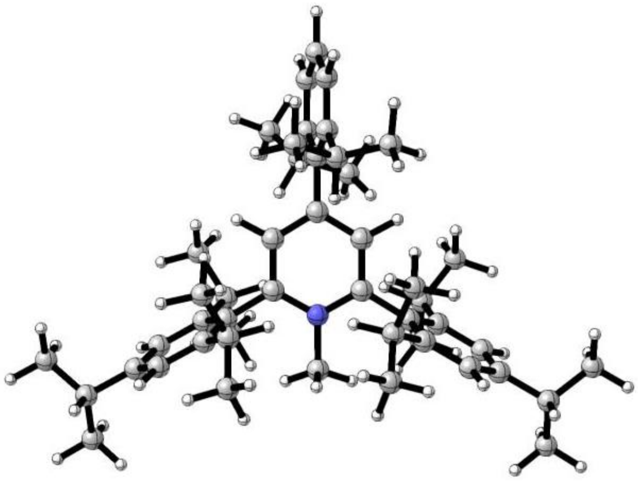

N	-0.0128560	-0.8262170	-0.0373380
C	-0.0081790	-2.3021640	-0.1406380
H	0.8439600	-2.6098600	-0.7416260
H	-0.9415090	-2.6252400	-0.5947500
H	0.0797790	-2.7294000	0.8634170
C	-1.2006770	-0.1690320	0.0997880
C	-1.2049060	1.2129700	0.1910420
H	-2.1608790	1.7161310	0.2979150
C	-0.0179410	1.9458670	0.1390320
C	1.1759990	1.2271890	0.0412400
C	1.1763010	-0.1554430	-0.0363050
C	-2.4725070	-0.9395490	0.1390660
C	-3.2548200	-1.0223930	-1.0279220
C	-4.4680080	-1.7056570	-0.9531610
H	-5.0870690	-1.7780650	-1.8443310
C	-4.9104620	-2.3000500	0.2315760
C	-4.1017890	-2.2052280	1.3654110
H	-4.4436950	-2.6648570	2.2892730
C	-2.8784360	-1.5337630	1.3464080
C	-2.8143200	-0.3914550	-2.3419820
H	-1.7713940	-0.0620010	-2.2385420
C	-3.6617960	0.8483730	-2.6589350
H	-4.7131440	0.5719150	-2.7886540
H	-3.3187670	1.3209000	-3.5846910
H	-3.6104890	1.5898200	-1.8544890
C	-2.8540630	-1.3916190	-3.5040710
H	-2.2691160	-2.2890510	-3.2807300
H	-2.4468260	-0.9321630	-4.4092550
H	-3.8781630	-1.7051560	-3.7267200
C	-6.2357190	-3.0348630	0.2822280
H	-6.3649020	-3.3994820	1.3085000
C	-2.0513110	-1.3978500	2.6164660
H	-1.0297210	-1.1089370	2.3342840
C	-2.6121460	-0.2695860	3.4940750
H	-2.6362100	0.6813600	2.9523680
H	-2.0007070	-0.1397340	4.3922880
H	-3.6347280	-0.5039370	3.8066830
C	-1.9467750	-2.7070900	3.4054280
H	-2.9112390	-2.9970400	3.8320060
H	-1.2473230	-2.5871400	4.2376710
H	-1.5965730	-3.5315000	2.7761780
C	-0.0276710	3.4338150	0.1883810
C	0.3740080	4.0867480	1.3667730
C	0.3582680	5.4856120	1.3808230
H	0.6562630	6.0150410	2.2812730
C	-0.0408620	6.2064200	0.2645040
C	-0.4394370	5.5423970	-0.8938140
H	-0.7499610	6.1227590	-1.7556370
C	-0.4451300	4.1496350	-0.9572150
C	0.7849000	3.3379420	2.6269810
H	0.7204400	2.2599950	2.4351140
C	-0.1715250	3.6439960	3.7874670
H	-0.1241170	4.7003280	4.0692640
H	0.0975020	3.0514130	4.6672180
H	-1.2070510	3.4149960	3.5186420
C	2.2352680	3.6544020	3.0130680
H	2.9282950	3.4175560	2.1990380
H	2.5304660	3.0797560	3.8963970
H	2.3546140	4.7159430	3.2508200
C	-0.8067680	3.4269700	-2.2499500
H	-1.3207120	2.4934940	-1.9879880
C	-1.7583980	4.2165530	-3.1511560
H	-1.2706760	5.0966620	-3.5810620
H	-2.6487330	4.5468250	-2.6081600
H	-2.0803730	3.5889860	-3.9869880
C	0.4685440	3.0511600	-3.0209270
H	0.2189690	2.4874220	-3.9264360
H	1.1456730	2.4406900	-2.4144300
H	1.0114530	3.9546950	-3.3176080
C	2.4477310	-0.9248960	-0.1076670
C	3.1078170	-1.0612760	-1.3419430
C	4.3132970	-1.7629710	-1.3630590
H	4.8390010	-1.8763600	-2.3084420
C	4.8636570	-2.3236300	-0.2082210
C	4.1759350	-2.1720690	0.9978390
H	4.6037160	-2.6036940	1.8989020
C	2.9673630	-1.4797200	1.0759180
C	2.5500020	-0.4703710	-2.6290570
H	1.5446630	-0.0774760	-2.4259510
C	3.4174300	0.7027020	-3.1048470
H	3.5027100	1.4769490	-2.3355410
H	2.9900070	1.1570390	-4.0038990
H	4.4289940	0.3600940	-3.3453970
C	2.4066380	-1.5269020	-3.7317520
H	3.3816810	-1.9152610	-4.0404640
H	1.9321600	-1.0874990	-4.6139250
H	1.7981880	-2.3742730	-3.4007000
C	6.1771300	-3.0790390	-0.2628060
H	6.3982710	-3.4184220	0.7564250
C	6.0702730	-4.3170950	-1.1619730
H	5.8774810	-4.0305720	-2.2013920
H	5.2618720	-4.9776850	-0.8363260
H	7.0060810	-4.8825660	-1.1414280
C	7.3236870	-2.1677010	-0.7184670
H	8.2727250	-2.7103830	-0.6915370
H	7.4120960	-1.2869030	-0.0765600
H	7.1659600	-1.8242550	-1.7464510
C	2.2761510	-1.2660670	2.4151540
H	1.2196730	-1.0311980	2.2251150
C	2.8858300	-0.0502830	3.1284170
H	3.9386240	-0.2367110	3.3639650
H	2.3550830	0.1558130	4.0637200
H	2.8375000	0.8453690	2.5001530
C	2.3033270	-2.5029600	3.3176490
H	1.9153290	-3.3875970	2.8028070
H	1.6931760	-2.3272440	4.2082060
H	3.3169600	-2.7295110	3.6602910
C	-7.4056930	-2.0964150	-0.0385620
H	-8.3563620	-2.6265620	0.0666910
H	-7.3423800	-1.7292740	-1.0685170
H	-7.4178600	-1.2311440	0.6299040
C	-6.2333770	-4.2486130	-0.6556720
H	-5.4089700	-4.9291580	-0.4251100
H	-6.1333630	-3.9351930	-1.7004330
H	-7.1723170	-4.8015640	-0.5629780
H	2.1319150	1.7423850	0.0228560
H	-0.0469900	7.2912690	0.2933120

A4⁻

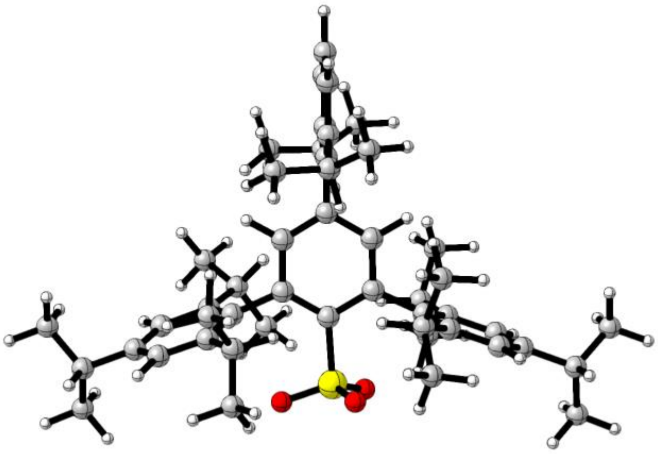

S	0.1361770	-2.6182090	0.2206980
O	-1.2457560	-3.1245270	0.1783430
O	0.8092260	-2.8177610	1.5218690
O	0.9436180	-2.9880420	-0.9587110
C	-3.6199060	0.1077900	-3.4356720
C	-1.8869570	-1.6574840	-2.9594010
C	-2.6401150	-0.4422920	-2.3953240
C	-7.6274530	-1.2116840	-0.3173910
C	-6.7377910	-3.5443370	-0.6782480
C	-6.6183010	-2.2640330	0.1577480
C	-2.8934900	-0.1603270	3.6121450
C	-2.3180990	-2.6053520	3.2656350
C	-2.3482230	-1.2091650	2.6344600
C	-3.2965170	-0.8064640	-1.0694430
C	-4.6016160	-1.2991950	-1.0389290
C	-5.1988490	-1.7273930	0.1483680
C	-4.4499480	-1.6706310	1.3200480
C	-3.1352330	-1.1985860	1.3342800
C	-2.5678400	-0.7482500	0.1346830
C	-1.1847420	3.5892980	3.2518340
C	1.2609660	4.1848270	3.4577030
C	0.1830670	3.5746380	2.5563500
C	-1.7607490	3.2848980	-2.9470020
C	0.5795400	3.9068630	-3.6580620
C	-0.2883510	3.3590250	-2.5204280
C	0.0846240	4.2623740	1.2027920
C	0.0575150	5.6580120	1.1259840
C	-0.0752890	6.3030830	-0.0991850
C	-0.1745560	5.5570590	-1.2693510
C	-0.1503790	4.1599760	-1.2333400
C	-0.0345330	3.5157210	0.0147010
C	3.1304850	0.7933590	3.0819860
C	2.9045990	-1.6730740	3.5984830
C	2.6551320	-0.5716820	2.5650000
C	7.5702930	-1.2030580	-0.9025220
C	6.7104730	-3.5642410	-0.6826050
C	6.6503800	-2.1416800	-0.1124420
C	2.7737450	0.5292480	-3.1815630
C	2.5795540	-1.9759440	-3.4808620
C	2.3996930	-0.8021600	-2.5151340
C	3.2887190	-0.8833830	1.2182130
C	4.6068240	-1.3461400	1.1492200
C	5.2239280	-1.6256290	-0.0668710
C	4.4890000	-1.4454490	-1.2412280
C	3.1723460	-0.9895310	-1.2186980
C	2.5823600	-0.6921620	0.0228690
C	-1.2091560	-0.1119210	0.1320060
C	-1.2057000	1.2929350	0.1282970
C	-0.0251400	2.0230360	0.0682790
C	1.1822940	1.3246580	0.0286510
C	1.2194780	-0.0684360	0.0592940
C	0.0135120	-0.7965590	0.1113980
H	-4.2117910	0.9387260	-3.0364590
H	-3.0674820	0.4675320	-4.3102340
H	-4.3105090	-0.6660920	-3.7885440
H	-2.5936360	-2.4695000	-3.1675420
H	-1.3796090	-1.3896160	-3.8942560
H	-1.1446120	-2.0474000	-2.2576430
H	-1.8993610	0.3431840	-2.2013470
H	-7.4357620	-0.9364620	-1.3604600
H	-8.6515510	-1.5968950	-0.2538480
H	-7.5585920	-0.3017220	0.2859700
H	-6.0250900	-4.3004080	-0.3379320
H	-7.7503540	-3.9589890	-0.6137900
H	-6.5243240	-3.3367900	-1.7326130
H	-6.8608120	-2.5177540	1.1982900
H	-3.9297770	-0.3890950	3.8890000
H	-2.2937550	-0.1398120	4.5292040
H	-2.8778730	0.8398570	3.1663070
H	-1.9358360	-3.3285780	2.5411700
H	-1.6576790	-2.6037580	4.1396300
H	-3.3171580	-2.9144030	3.5968690
H	-1.3100960	-0.9481060	2.4061360
H	-5.1673810	-1.3595210	-1.9658060
H	-4.8983050	-2.0224020	2.2480650
H	-1.9458250	3.1197410	2.6215620
H	-1.1429030	3.0456610	4.2016670
H	-1.5001890	4.6197100	3.4551890
H	1.0049540	5.2056350	3.7626180
H	1.3685010	3.5860200	4.3677490
H	2.2307970	4.2124200	2.9516850
H	0.4544820	2.5278260	2.3836080
H	-2.1526750	4.2894190	-3.1468370
H	-1.8693740	2.6843330	-3.8566110
H	-2.3756710	2.8292670	-2.1642890
H	1.6240640	4.0039730	-3.3469170
H	0.5404640	3.2273480	-4.5153560
H	0.2311300	4.8879010	-3.9996210
H	0.0492210	2.3367450	-2.3175880
H	0.1389320	6.2483360	2.0348590
H	-0.0964520	7.3884290	-0.1428670
H	-0.2710210	6.0693930	-2.2228660
H	2.9001920	1.5909130	2.3667690
H	2.6449780	1.0366430	4.0347680
H	4.2155440	0.7891760	3.2426470
H	3.9631810	-1.7381090	3.8771360
H	2.3370260	-1.4594110	4.5112350
H	2.5714230	-2.6337700	3.1994040
H	1.5723270	-0.5282310	2.4137860
H	7.5442300	-0.1897860	-0.4907980
H	8.6052580	-1.5630160	-0.8807360
H	7.2562300	-1.1463880	-1.9505740
H	6.3692940	-3.5761440	-1.7235440
H	7.7355530	-3.9516090	-0.6570500
H	6.0645130	-4.2395390	-0.1150600
H	7.0148920	-2.1776430	0.9228770
H	3.8374970	0.5416110	-3.4489900
H	2.1890520	0.6806330	-4.0969410
H	2.5854970	1.3753520	-2.5112090
H	2.3299510	-2.9108650	-2.9737460
H	1.9094060	-1.8545320	-4.3393350
H	3.6036150	-2.0319720	-3.8693420
H	1.3360880	-0.7725250	-2.2593690
H	5.1668860	-1.5018860	2.0693960
H	4.9495210	-1.6805000	-2.1989260
H	-2.1620870	1.8127770	0.1537270
H	2.1197240	1.8753180	-0.0258050

C5^{+}

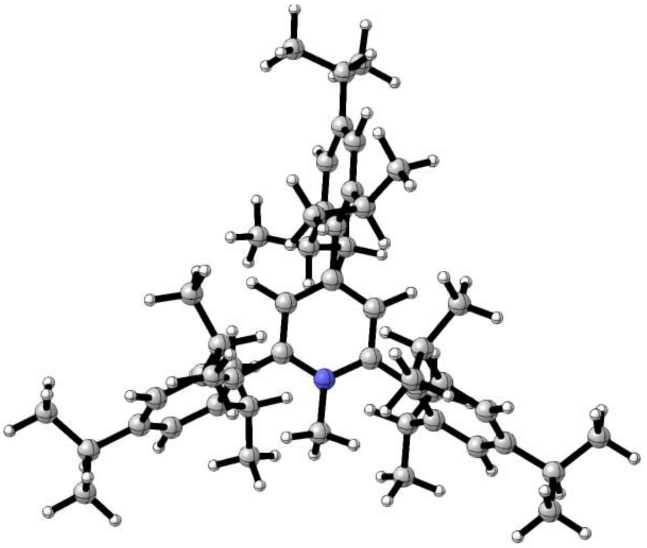

N	1.0622760	0.8152080	0.0118680
C	2.1954410	1.7649970	-0.0321920
H	1.9589730	2.5623350	-0.7337650
H	3.0907000	1.2315130	-0.3394940
H	2.3410680	2.1921230	0.9648560
C	1.2989160	-0.5215650	0.1378140
C	0.2273460	-1.4001070	0.1752500
H	0.4411290	-2.4594830	0.2776260
C	-1.0887450	-0.9463870	0.0728800
C	-1.2814420	0.4367350	-0.0126760
C	-0.2100050	1.3108610	-0.0336410
C	2.6960870	-1.0271250	0.2118280
C	3.2787700	-1.5820220	-0.9428360
C	4.5718750	-2.0946070	-0.8401810
H	5.0373950	-2.5272930	-1.7229270
C	5.2855350	-2.0671890	0.3603340
C	4.6774550	-1.5001080	1.4818860
H	5.2291370	-1.4785960	2.4183330
C	3.3871680	-0.9708240	1.4350460
C	2.5551090	-1.6197370	-2.2820290
H	1.5875410	-1.1122210	-2.1736210
C	2.2696450	-3.0632760	-2.7154720
H	3.2021920	-3.6128190	-2.8783350
H	1.7048910	-3.0745640	-3.6530140
H	1.6925380	-3.6045980	-1.9583250
C	3.3379530	-0.8657530	-3.3650450
H	3.5373140	0.1685140	-3.0672060
H	2.7710620	-0.8506980	-4.3005320
H	4.2998130	-1.3470490	-3.5662970
C	6.6867710	-2.6404250	0.4437210
H	7.0307830	-2.5056560	1.4764770
C	2.7351550	-0.4122950	2.6914250
H	1.8757460	0.2023520	2.3913280
C	2.1905520	-1.5573520	3.5574020
H	1.4756110	-2.1719140	3.0010980
H	1.6885660	-1.1629620	4.4461920
H	3.0081170	-2.2070090	3.8857690
C	3.6721600	0.4908650	3.5002330
H	4.4919020	-0.0784620	3.9476610
H	3.1212200	0.9634920	4.3183790
H	4.1097210	1.2782230	2.8783470
C	-2.2382080	-1.8888630	0.0477730
C	-3.1230820	-1.9366310	1.1389620
C	-4.2039080	-2.8217020	1.0797050
H	-4.8830720	-2.8731900	1.9264540
C	-4.4241560	-3.6436170	-0.0214480
C	-3.5237600	-3.5737460	-1.0899170
H	-3.6952540	-4.2178910	-1.9475530
C	-2.4259720	-2.7173920	-1.0843440
C	-2.9260190	-1.1077700	2.4015390
H	-2.0308060	-0.4861520	2.2821470
C	-2.6817410	-2.0096160	3.6193740
H	-3.5580200	-2.6293240	3.8332090
H	-2.4771500	-1.4020450	4.5063350
H	-1.8315560	-2.6777580	3.4527900
C	-4.1143200	-0.1692400	2.6452400
H	-4.2802790	0.4991270	1.7937070
H	-3.9426230	0.4427550	3.5361610
H	-5.0363140	-0.7368970	2.8041980
C	-5.6265760	-4.5640120	-0.1116480
H	-5.3613170	-5.3673200	-0.8106060
C	-6.0032240	-5.2078890	1.2248450
H	-5.1437410	-5.6957860	1.6934910
H	-6.7813710	-5.9600100	1.0695110
H	-6.4048810	-4.4693030	1.9264400
C	-6.8223760	-3.8004470	-0.7010700
H	-7.1101600	-2.9772070	-0.0379690
H	-7.6842070	-4.4648530	-0.8134950
H	-6.5822880	-3.3778970	-1.6809650
C	-1.5058200	-2.6226000	-2.2982450
H	-0.4756620	-2.5095660	-1.9390310
C	-1.5240240	-3.8680690	-3.1866130
H	-2.4788320	-3.9765210	-3.7102580
H	-1.3412600	-4.7795920	-2.6102390
H	-0.7460790	-3.7858280	-3.9508340
C	-1.8417140	-1.3755010	-3.1316660
H	-1.1555970	-1.2861460	-3.9812140
H	-1.7726190	-0.4563460	-2.5406650
H	-2.8628250	-1.4444620	-3.5213420
C	-0.4103010	2.7840480	-0.0990590
C	-0.6206420	3.4001980	-1.3458970
C	-0.8395680	4.7771720	-1.3643300
H	-1.0081110	5.2708130	-2.3184470
C	-0.8465810	5.5406930	-0.1938230
C	-0.6226570	4.8952330	1.0234690
H	-0.6287960	5.4862810	1.9357120
C	-0.4002400	3.5194160	1.0986820
C	-0.6269590	2.6032910	-2.6429610
H	-0.2228360	1.6021760	-2.4368280
C	-2.0627600	2.4268990	-3.1564190
H	-2.6995960	1.9383280	-2.4119190
H	-2.0742170	1.8217020	-4.0681330
H	-2.5069150	3.4007460	-3.3868690
C	0.2623810	3.2337590	-3.7213290
H	-0.1345160	4.1971790	-4.0544820
H	0.3101810	2.5795000	-4.5964410
H	1.2816960	3.3979620	-3.3585380
C	-1.0835700	7.0373120	-0.2463280
H	-1.0643310	7.4047830	0.7868830
C	0.0332020	7.7465650	-1.0229250
H	0.0409880	7.4303480	-2.0716060
H	1.0159720	7.5287010	-0.5954980
H	-0.1186590	8.8294090	-1.0027880
C	-2.4584670	7.3660670	-0.8408460
H	-2.6370900	8.4444710	-0.8080300
H	-3.2607230	6.8671370	-0.2902810
H	-2.5185510	7.0516620	-1.8882220
C	-0.2259420	2.8314130	2.4450800
H	0.2493590	1.8550540	2.2753230
C	-1.6008480	2.5602240	3.0725750
H	-2.1126440	3.5036640	3.2887330
H	-1.4966740	2.0025570	4.0091910
H	-2.2385040	1.9821490	2.3957390
C	0.6756740	3.6070970	3.4101030
H	1.6422270	3.8477590	2.9560170
H	0.8565940	3.0126360	4.3102770
H	0.2113460	4.5445440	3.7291340
C	6.6901850	-4.1428650	0.1348280
H	7.6958900	-4.5534850	0.2607660
H	6.3813600	-4.3298690	-0.8992800
H	6.0102640	-4.6874930	0.7957640
C	7.6540730	-1.8889390	-0.4793790
H	7.6667370	-0.8182440	-0.2571830
H	7.3712070	-2.0151760	-1.5299550
H	8.6695300	-2.2769950	-0.3603310
H	-2.2835740	0.8511860	-0.0706740

A5⁻

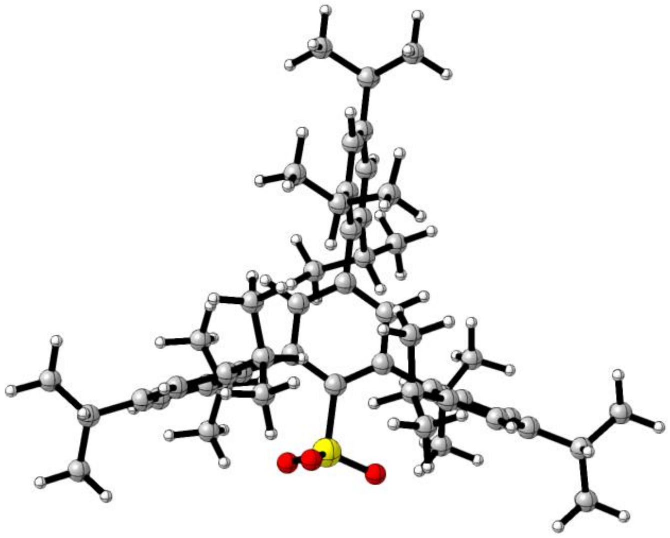

S	-0.9907940	-2.9134710	-0.5787410
C	-0.3910180	-1.2112830	-0.2905080
C	-1.3336240	-0.1654300	-0.2301320
C	-0.8880970	1.1551330	-0.1594410
H	-1.6228980	1.9585820	-0.1226190
C	0.4745630	1.4622220	-0.1070730
C	1.3808960	0.4106760	-0.0503400
H	2.4429890	0.6234630	0.0637990
C	0.9694920	-0.9282420	-0.1240790
C	-2.8097550	-0.4039840	-0.1085220
C	-3.3333860	-0.6654270	1.1692580
C	-4.7166290	-0.8083210	1.3109580
H	-5.1332190	-1.0143210	2.2951430
C	-5.5813440	-0.7024980	0.2249180
C	-5.0338600	-0.4506510	-1.0342650
H	-5.6930310	-0.3801730	-1.8978870
C	-3.6599340	-0.3068450	-1.2225950
C	-2.4364690	-0.7339680	2.3969640
H	-1.4086700	-0.8795320	2.0526170
C	-2.4891500	0.5969690	3.1600550
H	-3.4897520	0.7642520	3.5774360
H	-2.2582130	1.4385190	2.4978200
H	-1.7662950	0.5993930	3.9860760
C	-2.7590610	-1.9151110	3.3147890
H	-2.0183500	-1.9649760	4.1223810
H	-2.7195850	-2.8464340	2.7449060
H	-3.7452420	-1.8111510	3.7826560
C	-3.1112300	-0.0344680	-2.6127530
H	-2.0267770	-0.1808940	-2.5717050
C	-3.6537690	-1.0164090	-3.6558410
H	-3.4570970	-2.0414950	-3.3355160
H	-3.1595320	-0.8458000	-4.6190520
H	-4.7311680	-0.8824580	-3.8109750
C	-3.3995900	1.4160400	-3.0251100
H	-4.4809020	1.5836200	-3.1013160
H	-2.9498800	1.6405640	-3.9994400
H	-3.0049920	2.1276000	-2.2917960
C	0.9535540	2.8764180	-0.0606290
C	1.4731720	3.4785440	-1.2255290
C	1.9102100	4.8038060	-1.1585570
H	2.3089610	5.2827720	-2.0494970
C	1.8589740	5.5398630	0.0256580
C	1.3616150	4.9166930	1.1687570
H	1.3346630	5.4679710	2.1074240
C	0.9071330	3.5954130	1.1485350
C	1.5114580	2.7120010	-2.5419170
H	1.7047150	1.6595220	-2.3056860
C	0.1442060	2.7807650	-3.2373490
H	-0.6428410	2.3696750	-2.5998930
H	0.1574500	2.2068290	-4.1703820
H	-0.1105610	3.8210740	-3.4727340
C	2.6179540	3.1738820	-3.4929470
H	2.4155930	4.1714730	-3.8989820
H	2.6828360	2.4848870	-4.3406520
H	3.5930010	3.1958910	-2.9958560
C	2.3470780	6.9763130	0.0676530
H	2.6759070	7.2363250	-0.9470780
C	1.2230110	7.9424460	0.4607070
H	0.8718630	7.7343030	1.4773100
H	0.3679430	7.8457140	-0.2142930
H	1.5750700	8.9793970	0.4320600
C	3.5493430	7.1336950	1.0067560
H	3.9268080	8.1618860	0.9856400
H	4.3610040	6.4583140	0.7217490
H	3.2663640	6.9001050	2.0390170
C	0.4402430	2.9473810	2.4441190
H	0.0018270	1.9747750	2.2002630
C	-0.6380290	3.7721340	3.1545350
H	-0.2488520	4.7401320	3.4899290
H	-1.0036260	3.2350450	4.0358640
H	-1.4894920	3.9598110	2.4927250
C	1.6343720	2.6868530	3.3718770
H	2.3910820	2.0751300	2.8706340
H	1.3093260	2.1560140	4.2735100
H	2.1050250	3.6295380	3.6758480
C	2.0622920	-1.9388180	0.0511000
C	2.9744690	-2.1561250	-0.9914090
C	4.0894960	-2.9630570	-0.7588400
H	4.7912050	-3.1397050	-1.5734180
C	4.3081430	-3.5662120	0.4781110
C	3.3617950	-3.3700300	1.4846120
H	3.5088830	-3.8725290	2.4374310
C	2.2302270	-2.5737240	1.2961300
C	2.7212940	-1.6090250	-2.3872280
H	1.7833400	-1.0452240	-2.3618410
C	5.5188470	-4.4500590	0.7152440
H	5.4745500	-4.7860150	1.7598060
C	6.8312560	-3.6806180	0.5237660
H	6.9299010	-3.3378860	-0.5122220
H	7.6935470	-4.3179320	0.7505670
H	6.8677480	-2.7998730	1.1716300
C	5.4858650	-5.6946380	-0.1806010
H	4.5559730	-6.2522600	-0.0397420
H	6.3312260	-6.3566920	0.0395920
H	5.5454900	-5.4098400	-1.2368940
C	1.1878650	-2.4185890	2.3989380
H	0.2056130	-2.4065780	1.9159020
C	1.1509600	-3.6021380	3.3677020
H	2.0396700	-3.6393810	4.0099040
H	1.0670210	-4.5466430	2.8227590
H	0.2756360	-3.5088960	4.0183820
C	1.3611690	-1.1029640	3.1721140
H	0.5810490	-1.0084830	3.9383730
H	1.2883410	-0.2348430	2.5096060
H	2.3385090	-1.0720200	3.6702070
O	0.2107540	-3.6857710	-0.9393740
O	-1.6013500	-3.3033080	0.7119720
O	-1.9533940	-2.7491490	-1.6845470
C	-7.0776120	-0.8814400	0.4035350
H	-7.2580990	-1.0487680	1.4737920
C	-7.5859800	-2.1119210	-0.3579330
H	-7.4424520	-1.9831600	-1.4363440
H	-8.6550820	-2.2695100	-0.1745100
H	-7.0404740	-3.0101840	-0.0564590
C	-7.8546670	0.3727840	-0.0142150
H	-7.7195170	0.5726860	-1.0828800
H	-7.5067960	1.2516830	0.5366380
H	-8.9274220	0.2458460	0.1708630
C	2.5178080	-2.7621160	-3.3785770
H	3.4347390	-3.3540430	-3.4865300
H	2.2492480	-2.3719080	-4.3669200
H	1.7165260	-3.4155840	-3.0219350
C	3.8290270	-0.6533990	-2.8449160
H	3.9344330	0.1913520	-2.1544740
H	3.6063690	-0.2561390	-3.8420460
H	4.7964990	-1.1665870	-2.8961330

3

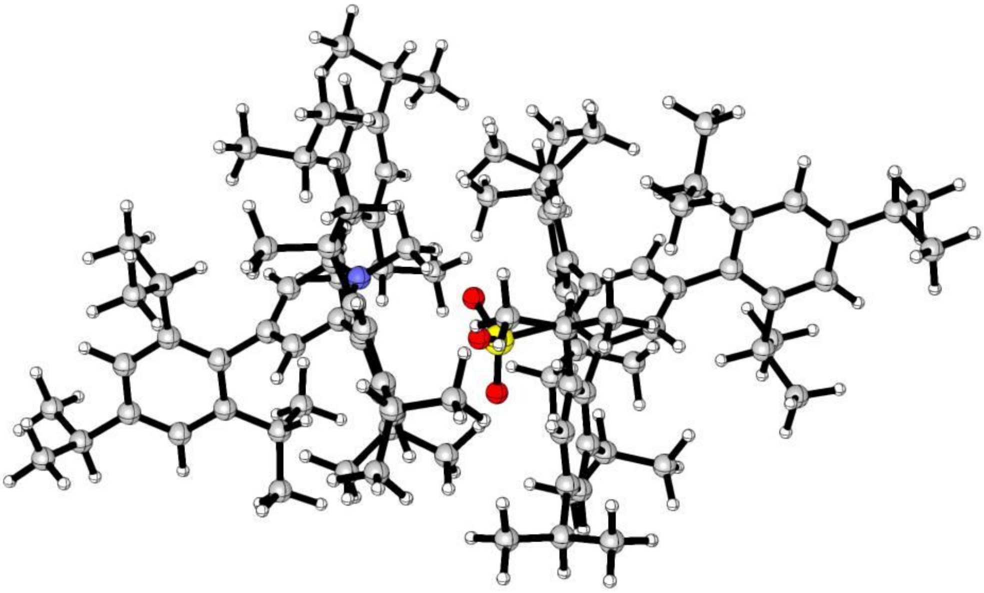

S	0.63606400	-0.24050200	-1.00424900
C	2.42517100	-0.12394300	-0.71284400
C	3.14870600	-1.31790300	-0.51823700
C	4.49376500	-1.25001100	-0.15077400
H	5.04099900	-2.17885900	0.00331700
C	5.15078500	-0.02849200	0.00116700
C	4.44107200	1.12915500	-0.28822500
H	4.95240400	2.08898400	-0.25116200
C	3.08761800	1.11007900	-0.65811400
C	2.60988400	-2.69716600	-0.75762100
C	2.59918400	-3.19520500	-2.07265800
C	2.27682000	-4.54093600	-2.27504300
H	2.28296200	-4.94187600	-3.28666900
C	1.96892200	-5.39510000	-1.21874100
C	1.95736200	-4.86596800	0.07369700
H	1.71136000	-5.51275900	0.91493600
C	2.27393200	-3.52972100	0.32505500
C	3.03224500	-2.33651400	-3.25212400
H	2.97538500	-1.28828700	-2.94537500
C	4.49326900	-2.64710900	-3.60818600
H	4.59166400	-3.67537500	-3.97592000
H	5.14232400	-2.54196400	-2.73221100
H	4.85627000	-1.96713800	-4.38821400
C	2.11943300	-2.48438400	-4.47104800
H	2.42293900	-1.77159700	-5.24618300
H	1.08509400	-2.26918400	-4.19294200
H	2.17836200	-3.48759600	-4.90783900
C	2.27389900	-3.01571600	1.75630900
H	2.50503300	-1.94534600	1.72811000
C	0.89852200	-3.17208100	2.41278700
H	0.12820200	-2.69989600	1.79845000
H	0.89808100	-2.71509000	3.41041700
H	0.64032400	-4.22840400	2.53227600
C	3.35068900	-3.72400100	2.58986600
H	3.12308900	-4.79219800	2.68468200
H	3.40173200	-3.29932600	3.59854600
H	4.33979200	-3.63516700	2.12909500
C	6.58624200	0.04541000	0.40813900
C	6.92016800	0.36927500	1.73871600
C	8.26965300	0.43629600	2.09279300
H	8.54410900	0.68051700	3.11589100
C	9.28871200	0.20323400	1.16875200
C	8.93190300	-0.09922600	-0.14454500
H	9.71382200	-0.26489000	-0.88360700
C	7.59627500	-0.18305800	-0.54480100
C	5.83129200	0.58281300	2.78257300
H	4.96520100	1.02243800	2.27446500
C	5.38334900	-0.76723500	3.35971400
H	5.02028400	-1.42970400	2.56941600
H	4.57480100	-0.62876000	4.08630900
H	6.22061000	-1.26353800	3.86388200
C	6.23299900	1.54034600	3.90655900
H	6.99999600	1.10570200	4.55640800
H	5.36281700	1.75747500	4.53392900
H	6.61424000	2.48729300	3.51158800
C	10.74606600	0.28899600	1.58242000
H	10.76514600	0.53185400	2.65269100
C	11.46446900	-1.05199300	1.38989400
H	11.49086600	-1.32920800	0.33045300
H	10.95602800	-1.85327900	1.93351100
H	12.49789600	-0.99087700	1.74630200
C	11.47671400	1.40918400	0.83192800
H	12.51071600	1.49742900	1.18097400
H	10.97755200	2.37144100	0.97684000
H	11.50223000	1.20254800	-0.24355900
C	7.26998700	-0.45115400	-2.00679400
H	6.19123800	-0.61406300	-2.09097700
C	7.96356400	-1.70903500	-2.54004000
H	9.05289800	-1.59526500	-2.54890100
H	7.64160800	-1.91025800	-3.56700800
H	7.71999000	-2.58313300	-1.92783100
C	7.61078400	0.77398500	-2.86540200
H	7.08809800	1.66415700	-2.50079300
H	7.31448500	0.60583600	-3.90661400
H	8.68719900	0.97910700	-2.84214100
C	2.56641900	2.46726100	-1.03128600
C	2.38831000	3.42908200	-0.02719100
C	2.23905100	4.77348100	-0.38920100
H	2.13746200	5.51677000	0.39856600
C	2.26401700	5.17756600	-1.72135800
C	2.36395500	4.18723900	-2.70397600
H	2.34246400	4.49366300	-3.74773100
C	2.50483200	2.83452900	-2.39261300
C	2.35431500	3.04087900	1.44412600
H	2.43044900	1.94953200	1.50318800
C	2.16087100	6.63599000	-2.13867700
H	2.75785300	6.74291400	-3.05435300
C	2.72458500	7.60955300	-1.10114900
H	2.09212700	7.64835700	-0.20708700
H	2.76169200	8.62169800	-1.51566000
H	3.73445200	7.32503700	-0.79144600
C	0.71377300	7.01074200	-2.48438400
H	0.31619600	6.37115000	-3.27787100
H	0.64887600	8.05376300	-2.81305000
H	0.07541600	6.89057300	-1.60044700
C	2.61147300	1.78322900	-3.49351100
H	2.07732000	0.89187700	-3.14658600
C	1.93147300	2.20699100	-4.79824500
H	2.49121000	2.99556100	-5.31447900
H	0.91318300	2.56485500	-4.62031500
H	1.87598300	1.34772400	-5.47324400
C	4.06941100	1.38762300	-3.77741400
H	4.10613900	0.64461800	-4.58313500
H	4.55476400	0.95316900	-2.89848600
H	4.65145700	2.26248700	-4.09158500
O	0.10195600	1.11934400	-0.75263600
O	0.46253000	-0.70182600	-2.39395200
O	0.16373800	-1.21093300	0.01189500
C	1.71312200	-6.87035200	-1.47057700
H	1.68028400	-7.01241300	-2.55844700
C	0.37702100	-7.34614300	-0.89251300
H	0.36323400	-7.22611000	0.19626600
H	0.21528800	-8.40715900	-1.11091800
H	-0.46170800	-6.77834700	-1.30748800
C	2.86520800	-7.71952800	-0.91650200
H	2.92626200	-7.61625300	0.17282500
H	3.82338200	-7.40135400	-1.33680400
H	2.71640300	-8.77932800	-1.14929800
C	1.01853300	3.43812300	2.08423900
H	0.90522100	4.52859900	2.12232600
H	0.95592600	3.06214500	3.11240900
H	0.17841700	3.03773500	1.50785800
C	3.53151800	3.64186000	2.22262600
H	4.49205400	3.34289100	1.79038500
H	3.51087400	3.31378200	3.26799600
H	3.48662600	4.73686900	2.21210500
N	-2.68032700	0.20172300	1.13109300
C	-1.37693000	0.34586500	1.82505400
H	-0.71667700	0.91244900	1.16621800
H	-0.95794400	-0.64420800	1.97748100
H	-1.53752600	0.87160400	2.76768200
C	-3.23172400	-1.02375200	0.91504400
C	-4.45083600	-1.10655900	0.24738200
H	-4.89311000	-2.08837300	0.12902800
C	-5.11216100	0.01959500	-0.21813800
C	-4.52714900	1.25548500	0.05381100
H	-5.02511800	2.17668900	-0.22832000
C	-3.32548200	1.34239800	0.73305100
C	-2.66984200	-2.31309800	1.41761400
C	-2.30757700	-3.29911700	0.47650800
C	-2.05496800	-4.59361100	0.94403400
H	-1.76422000	-5.35361900	0.22317400
C	-2.18852900	-4.93613700	2.28749500
C	-2.51093200	-3.92625100	3.19674100
H	-2.59766300	-4.18727600	4.24825900
C	-2.75775700	-2.61526500	2.79505100
C	-2.21848300	-3.03328400	-1.02270500
H	-2.22025700	-1.94866000	-1.18427900
C	-3.40073300	-3.67054700	-1.77396400
H	-3.40139900	-4.75566200	-1.62031100
H	-3.29601200	-3.48588500	-2.84827100
H	-4.38006500	-3.29341300	-1.46324000
C	-0.91318900	-3.56669200	-1.62469100
H	-0.05472800	-3.26057700	-1.02858700
H	-0.77892300	-3.15711600	-2.63006800
H	-0.93126800	-4.65961700	-1.69984500
C	-1.99598900	-6.35662300	2.79147000
H	-2.59499200	-6.44515300	3.70763300
C	-3.21493800	-1.58114100	3.81876500
H	-2.83458200	-0.60512100	3.50859500
C	-4.74768300	-1.48066900	3.86504800
H	-5.16992700	-1.21388300	2.89018100
H	-5.05747900	-0.71613200	4.58768100
H	-5.18361800	-2.43807600	4.17018500
C	-2.65510900	-1.82716700	5.22290900
H	-3.11500600	-2.70048300	5.69596600
H	-2.86710500	-0.96391500	5.86093000
H	-1.57195800	-1.97969000	5.19801000

C	-6.41863400	-0.09424600	-0.92326900
C	-7.60835600	0.14061200	-0.21211500
C	-8.82151400	0.01771100	-0.89223500
H	-9.74912200	0.18560400	-0.34844800
C	-8.87571300	-0.32563000	-2.24279600
C	-7.67646500	-0.55887100	-2.91820300
H	-7.71729700	-0.83233800	-3.96889300
C	-6.43670700	-0.45544900	-2.28511600
C	-7.61234500	0.46123300	1.27661800
H	-6.57676400	0.60508700	1.60587900
C	-8.18091500	-0.71535800	2.08116600
H	-9.23009900	-0.89369000	1.82226100
H	-8.12496500	-0.50764900	3.15497600
H	-7.62443200	-1.63618600	1.87888100
C	-8.36908900	1.75815300	1.58418300
H	-7.95788800	2.60015400	1.01850300
H	-8.30051900	1.99359300	2.65106500
H	-9.43035400	1.67001300	1.33039700
C	-10.20623600	-0.45345400	-2.96034200
H	-9.98760700	-0.71735700	-4.00262000
C	-11.05951200	-1.57630700	-2.35767900
H	-10.52327200	-2.52933300	-2.36732900
H	-11.98992000	-1.69614300	-2.92137200
H	-11.32433500	-1.34857800	-1.31935500
C	-10.97324700	0.87439400	-2.95983900
H	-11.23910400	1.17008300	-1.93910700
H	-11.90099000	0.78148600	-3.53287900
H	-10.37427900	1.67769400	-3.39783200

C	-5.13839200	-0.65553700	-3.05662800
H	-4.39925300	-1.08797900	-2.37139000
C	-5.26715900	-1.61798100	-4.23860500
H	-5.87449500	-1.19091900	-5.04341400
H	-5.71322600	-2.57067400	-3.93640300
H	-4.27558400	-1.82019700	-4.65356300
C	-4.58737000	0.69935000	-3.52524500
H	-3.62623400	0.56802200	-4.03221300
H	-4.43188500	1.38049400	-2.68390300
H	-5.28910600	1.17551500	-4.21932700
C	-2.79975500	2.68216000	1.12004700
C	-2.27045800	3.54997200	0.15122900
C	-1.84486600	4.81770800	0.56899600
H	-1.40017600	5.47621000	-0.17110300
C	-1.94004300	5.23257200	1.89305900
C	-2.51278600	4.35742700	2.82329700
H	-2.61672500	4.68618700	3.85567400
C	-2.96290200	3.09297300	2.46165700
C	-2.18957200	3.19122600	-1.32444100
H	-2.25103600	2.10125100	-1.42145600
C	-3.35312200	3.84924400	-2.08581600
H	-4.33770100	3.55145100	-1.71208600
H	-3.30081900	3.59073800	-3.14850100
H	-3.28452700	4.93949800	-1.99913400
C	-0.86629900	3.62444900	-1.96075600
H	-0.84173100	4.70668800	-2.12402200
H	-0.75280100	3.13599000	-2.93349700
H	-0.01522600	3.33214900	-1.34642500

C	-1.47528500	6.60052900	2.36325700
H	-0.91594900	6.43828600	3.29542900
C	-0.54816300	7.30725200	1.37556300
H	-1.09094600	7.61724600	0.47527900
H	0.27710800	6.65863900	1.06429700
H	-0.12682500	8.20795800	1.83136300
C	-2.68467200	7.49119400	2.68677000
H	-2.35657600	8.46325100	3.06826600
H	-3.33612900	7.03138800	3.43543600
H	-3.27809600	7.65998300	1.78163500
C	-3.73331400	2.24055600	3.46292800
H	-3.78155500	1.21120200	3.09094500
C	-5.18475200	2.73746500	3.54612000
H	-5.21960300	3.75534500	3.94897000
H	-5.77961700	2.08600700	4.19648300
H	-5.65115200	2.75343500	2.55497200
C	-3.09230100	2.18659700	4.85231800
H	-2.04642000	1.86581200	4.80378600
H	-3.63877000	1.48099400	5.48705700
H	-3.12209000	3.16160400	5.34817300
C	-2.49845300	-7.41398800	1.80459800
H	-2.46115300	-8.40522400	2.26634300
H	-1.87675900	-7.44960500	0.90445300
H	-3.52950000	-7.21434400	1.49799800
C	-0.53261300	-6.61938700	3.17055100
H	-0.18570200	-5.91746300	3.93482400
H	0.11359800	-6.50627900	2.29311000
H	-0.41244400	-7.63792200	3.55365000

1$_{\mathbf{trunc}}$ (first geometry)

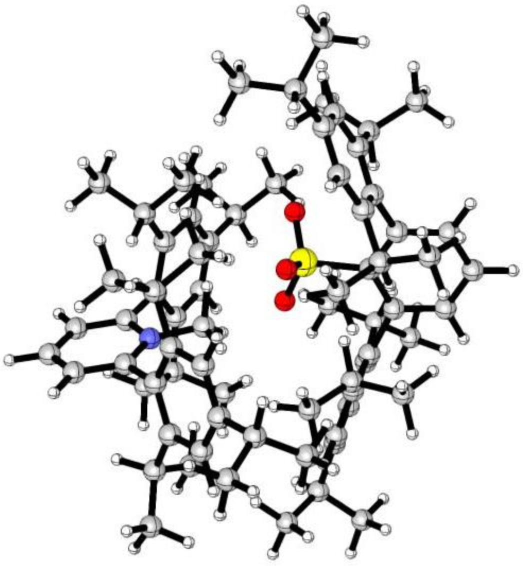

N	-2.2377340	-0.8291440	2.3156690
C	-1.9827860	-0.0909570	3.4303850
C	-2.7635070	-0.2911230	4.5626390
H	-2.5501800	0.3092140	5.4394240
C	-3.7712550	-1.2483500	4.5514550
C	-3.9899920	-1.9960190	3.3999450
H	-4.7559100	-2.7618130	3.3523130
C	-3.2105350	-1.7782760	2.2699450
C	-1.3318620	-0.6289190	1.1627200
H	-1.7289910	-1.1197900	0.2826640
H	-0.3650350	-1.0665570	1.4097030
H	-1.1783970	0.4346460	0.9806830
C	-0.8682020	0.8972890	3.3906880
C	-1.1481190	2.2285610	3.0480040
C	-0.0939210	3.1417790	3.0559370
H	-0.2872160	4.1769060	2.7807000
C	1.2137670	2.7582680	3.3394230
C	1.4657180	1.4134250	3.6192390
H	2.4898440	1.0894410	3.7834510
C	0.4470750	0.4650320	3.6496100
C	-2.5207900	2.6749510	2.5707990
H	-3.1894600	1.8027530	2.5613130
C	-2.4364360	3.2049660	1.1310970
H	-1.8743120	4.1450840	1.0924900
H	-3.4405180	3.3998020	0.7358580
H	-1.9193510	2.5091140	0.4605670
C	-3.1419870	3.7145320	3.5105990
H	-3.2429650	3.3234250	4.5282940
H	-4.1327300	4.0125260	3.1526520
H	-2.5172960	4.6124410	3.5575840
C	2.3311940	3.7824170	3.3316180
H	1.9223230	4.6946450	2.8779860
C	3.5312010	3.3299240	2.4934210
H	4.0563850	2.4993270	2.9778500
H	4.2440450	4.1547780	2.3866920
H	3.2240590	2.9779730	1.5048900
C	2.7600780	4.1183000	4.7667550
H	1.9165820	4.4840960	5.3604350
H	3.5419870	4.8847380	4.7645180
H	3.1618060	3.2270850	5.2621620
C	0.7689150	-0.9895810	3.9638880
H	-0.1103600	-1.6015870	3.7136960
C	1.9436090	-1.5314170	3.1375560
H	1.7973580	-1.3971380	2.0606000
H	2.0767260	-2.5978080	3.3502400
H	2.8789150	-1.0248330	3.3958680
C	1.0257750	-1.1645350	5.4672010
H	1.8975900	-0.5758690	5.7715430
H	1.2227410	-2.2145310	5.7065720
H	0.1701550	-0.8263400	6.0617220
C	-3.3880790	-2.5477180	1.0052490
C	-2.4499930	-3.5402640	0.6721940
C	-2.5864670	-4.1860840	-0.5590790
H	-1.8671660	-4.9529800	-0.8375450
C	-3.6090330	-3.8672200	-1.4497590
C	-4.5272550	-2.8771720	-1.0848080
H	-5.3247080	-2.6195300	-1.7759800
C	-4.4414100	-2.2023790	0.1316010
C	-1.3254190	-3.9446970	1.6161580
H	-1.2812140	-3.2200390	2.4390750
C	0.0548770	-3.9498870	0.9452720
H	0.1093550	-4.7023170	0.1513130
H	0.8192170	-4.2009930	1.6876100
H	0.3173610	-2.9796730	0.5105050
C	-1.6398050	-5.3128440	2.2394260
H	-2.6050850	-5.3037620	2.7560940
H	-0.8631840	-5.5921000	2.9578900
H	-1.6799400	-6.0861180	1.4649640
C	-3.7283760	-4.5826400	-2.7821650
H	-2.9136130	-5.3158810	-2.8284880
C	-3.5592250	-3.6151510	-3.9599450
H	-4.3347860	-2.8413330	-3.9461540
H	-3.6386390	-4.1546630	-4.9087680
H	-2.5874070	-3.1151040	-3.9281090
C	-5.0571540	-5.3419800	-2.8855820
H	-5.1835700	-6.0364900	-2.0501070
H	-5.0994600	-5.9116110	-3.8188570
H	-5.9045460	-4.6477710	-2.8796930
C	-5.4113100	-1.0768390	0.4735300
H	-5.5636220	-1.0845980	1.5589960
C	-4.8202410	0.2921650	0.0984180
H	-3.8320790	0.4627830	0.5417120
H	-5.4832530	1.0969350	0.4338890
H	-4.7074660	0.3709660	-0.9884720
C	-6.7903240	-1.2442970	-0.1700960
H	-6.7492920	-1.0911230	-1.2530670
H	-7.4788290	-0.4973050	0.2350230
H	-7.2074540	-2.2374110	0.0210660
S	1.4790070	0.6407840	-0.1498150
O	0.4235580	1.6251550	0.1771270
O	0.9519830	-0.7440660	-0.2844360
O	2.6576670	0.7385470	0.7283570
C	2.0911220	1.0551460	-1.8050310
C	1.4703380	2.0232900	-2.6041640
C	2.0897840	2.3808280	-3.8129560
H	1.6161360	3.1418450	-4.4281920
C	3.2594280	1.7640390	-4.2349720
H	3.7254770	2.0601820	-5.1698360
C	3.7953220	0.7199630	-3.4827990
H	4.6578280	0.1658620	-3.8433410
C	3.2114530	0.3391160	-2.2748470
C	0.1267920	2.6418260	-2.3429610
C	-1.0365830	1.8913340	-2.6025000
C	-2.2791470	2.5276690	-2.5281090
H	-3.1742080	1.9478350	-2.7343860
C	-2.4006490	3.8722660	-2.1757120
C	-1.2362200	4.5713520	-1.8615540
H	-1.3215910	5.6053810	-1.5272050
C	0.0264940	3.9809620	-1.9345360
C	-0.9603460	0.4042260	-2.9321590
H	-0.1066530	-0.0116720	-2.3891900
C	-2.1814270	-0.3799430	-2.4418320
H	-2.4591650	-0.0704400	-1.4306060
H	-1.9522450	-1.4512970	-2.4168500
H	-3.0571150	-0.2433980	-3.0902880
C	-0.7339880	0.1621080	-4.4304690
H	-1.5636970	0.5780000	-5.0141840
H	-0.6728000	-0.9132140	-4.6386660
H	0.1947770	0.6271060	-4.7744760
C	-3.7357900	4.5963470	-2.1271900
H	-3.7121730	5.2481120	-1.2417850
C	-4.9419700	3.6668310	-1.9894980
H	-5.0855670	3.0675670	-2.8957580
H	-5.8538770	4.2520610	-1.8358200
H	-4.8253310	2.9797900	-1.1453110
C	-3.8985050	5.4927850	-3.3638810
H	-3.0710890	6.2026270	-3.4495390
H	-4.8362890	6.0570580	-3.3179350
H	-3.9092250	4.8792820	-4.2716500
C	1.2442390	4.7554240	-1.4581510
H	2.1338280	4.1597890	-1.6857960
C	1.3988100	6.1094130	-2.1574300
H	1.4505140	5.9898970	-3.2442690
H	2.3139510	6.6080270	-1.8219340
H	0.5580910	6.7744370	-1.9315390
C	1.1847830	4.9126390	0.0686550
H	0.3375430	5.5490350	0.3575590
H	2.1033500	5.3800020	0.4428410
H	1.0599090	3.9321000	0.5415010
C	3.6843570	-0.9186190	-1.6065220
C	4.7213060	-0.9042510	-0.6586570
C	5.1024670	-2.1075630	-0.0647660
H	5.8975270	-2.0992760	0.6774760
C	4.4791100	-3.3155900	-0.3826110
C	3.4597330	-3.3035790	-1.3319370
H	2.9726280	-4.2428740	-1.5887120
C	3.0449550	-2.1231000	-1.9532040
C	5.4350050	0.3881950	-0.2995210
H	4.7672060	1.2133590	-0.5656620
C	6.7304870	0.5240880	-1.1117860
H	7.4144020	-0.3017800	-0.8836570
H	7.2386850	1.4647560	-0.8731170
H	6.5297700	0.5064980	-2.1875330
C	5.7208250	0.5123330	1.1995850
H	4.8042280	0.3412810	1.7702500
H	6.0891290	1.5192100	1.4244680
H	6.4894730	-0.1970800	1.5272690
C	4.8780240	-4.6056040	0.3092440
H	4.3103120	-5.4169430	-0.1654020
C	6.3698380	-4.9151550	0.1420390
H	6.6498100	-4.9417430	-0.9148920
H	6.6160140	-5.8828600	0.5917220
H	6.9844320	-4.1533930	0.6333500
C	4.4930840	-4.5677280	1.7937360
H	5.0316260	-3.7654890	2.3104800
H	4.7375390	-5.5153380	2.2860970
H	3.4221600	-4.3768320	1.9107530
C	1.9529940	-2.1770720	-3.0149760
H	1.6147440	-1.1545920	-3.2085080
C	0.7167970	-2.9722720	-2.5814170
H	0.9524450	-4.0260130	-2.3912580
H	-0.0369530	-2.9460290	-3.3784860
H	0.2868060	-2.5349530	-1.6765840
C	2.5240520	-2.7279150	-4.3292190
H	3.3741380	-2.1280790	-4.6690220
H	1.7605700	-2.7200630	-5.1152790
H	2.8709580	-3.7593350	-4.1972670
H	-4.3762780	-1.4179340	5.4363640

Model ion pair system in Figure 2d (first geometry)

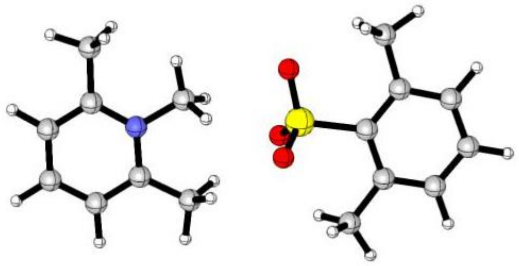

C	2.9083640	-0.1210850	-0.3012030	N	-3.1547440	0.4693120	0.7164670
C	3.2419610	-1.4796870	-0.4802970	C	-1.8133630	1.1061890	0.6740600
C	4.5687060	-1.8060950	-0.7649010	H	-1.4065480	1.0257840	-0.3366530
C	5.5423120	-0.8202290	-0.8720790	H	-1.1423050	0.5763420	1.3518400
C	5.1915320	0.5110680	-0.7023190	H	-1.8735990	2.1460190	0.9681230
C	3.8738370	0.8952020	-0.4183640	C	-3.2078620	-0.8783160	0.5097790
H	4.8335570	-2.8505300	-0.9053490	C	-4.4494910	-1.5108950	0.5366600
H	6.5710040	-1.0893990	-1.0932650	H	-4.4814480	-2.5810180	0.3728490
H	5.9490460	1.2845090	-0.7939410	C	-5.6034510	-0.7822220	0.7649240
S	1.1734970	0.2409240	0.0510040	C	-5.5109300	0.5911170	0.9617110
O	1.0312830	1.6828480	0.3367920	H	-6.3937100	1.1939460	1.1360460
O	0.4251320	-0.1647920	-1.1720910	C	-4.2733120	1.2157330	0.9338900
O	0.8181220	-0.6001380	1.2258160	H	-6.5701820	-1.2745630	0.7861800
C	3.6014430	2.3752440	-0.2700590	C	-4.1581920	2.6988120	1.1314900
C	2.2355770	-2.6016840	-0.3761540	H	-3.5757540	2.9396670	2.0249360
H	2.8358820	2.7141870	-0.9700530	H	-3.6795250	3.1794170	0.2743380
H	3.2286290	2.6160370	0.7267540	H	-5.1564430	3.1194150	1.2499720
H	4.5263590	2.9280350	-0.4535160	C	-1.9493890	-1.6328290	0.2546910
H	1.8953650	-2.7152420	0.6558040	H	-1.3903330	-1.2277130	-0.6002400
H	1.3536010	-2.3912320	-0.9860140	H	-1.2395420	-1.5543340	1.0877580
H	2.6913700	-3.5359700	-0.7130500	H	-2.1895470	-2.6808450	0.0768940

Model hydrogen-bonded system in Figure 2d (first geometry)

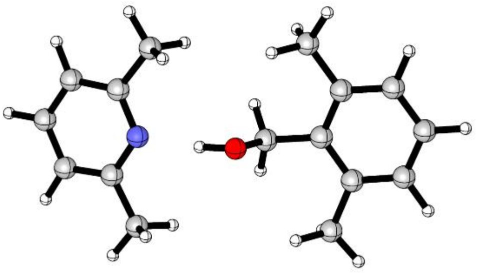

C	1.2639040	2.7486370	1.1666950
C	2.5061670	2.3224920	0.6699450
C	3.6696700	2.9652530	1.1019790
C	3.6080860	4.0055740	2.0234530
C	2.3760870	4.4031590	2.5346960
C	1.1979930	3.7792080	2.1193370
H	4.6320650	2.6373410	0.7176870
H	4.5190400	4.4961520	2.3530690
H	2.3252450	5.2009140	3.2712140
C	-0.1280970	4.2020730	2.7048970
C	2.6044430	1.1721840	-0.3049210
H	-0.7431980	4.7297220	1.9663860
H	-0.6920210	3.3299490	3.0492090
H	0.0226540	4.8750980	3.5521570
H	2.0399430	0.3060340	0.0532250
H	2.2111750	1.4427410	-1.2911250
H	3.6463460	0.8705890	-0.4352220
C	-0.0087340	2.0648830	0.7288890
H	0.1192440	1.6362080	-0.2743210
H	-0.8181190	2.8066210	0.6530680

O	-0.3424270	1.0584420	1.6674280
H	-1.2130780	0.7000200	1.3973710
N	-2.9151300	0.3386220	0.6820060
C	-3.9598900	1.0225050	1.1656930
C	-5.2650150	0.7558860	0.7451190
H	-6.0941320	1.3223730	1.1553650
C	-5.4725380	-0.2430950	-0.1997360
C	-4.3801560	-0.9452030	-0.6983250
H	-4.5061860	-1.7310090	-1.4353620
C	-3.1045580	-0.6237450	-0.2297050
H	-6.4763870	-0.4733440	-0.5437510
C	-1.8677380	-1.3291270	-0.7149020
H	-1.2009930	-0.6167610	-1.2105490
H	-1.3195950	-1.7521240	0.1314650
H	-2.1115120	-2.1282670	-1.4173020
C	-3.6435900	2.0940130	2.1731050
H	-3.0024370	1.6929010	2.9626830
H	-3.0931300	2.9092250	1.6914900
H	-4.5512020	2.5049720	2.6191530

Model halogen-bonded system in Figure 2d (first geometry)

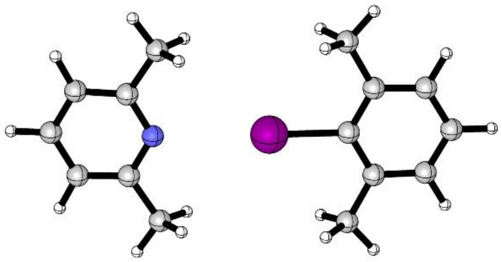

C	1.2187930	2.9045940	0.7800730	N	-3.0389150	-0.1623200	0.6366290
C	1.7339150	3.2944440	2.0248200	C	-3.5539120	-0.6364740	1.7776860
C	2.8919060	4.0788670	2.0394570	C	-4.5407500	-1.6260870	1.7868170
C	3.5155580	4.4621100	0.8582000	H	-4.9390990	-1.9883640	2.7286520
C	2.9843470	4.0630210	-0.3623540	C	-4.9942650	-2.1286100	0.5726520
C	1.8282590	3.2782900	-0.4264360	C	-4.4559340	-1.6324660	-0.6090740
H	3.3032610	4.3886020	2.9960870	H	-4.7867440	-1.9998380	-1.5747750
H	4.4137240	5.0708610	0.8887440	C	-3.4720500	-0.6426200	-0.5354520
H	3.4681990	4.3603160	-1.2885260	H	-5.7606530	-2.8976130	0.5475660
C	1.2862710	2.8672520	-1.7707350	C	-2.8390880	-0.0637180	-1.7716560
C	1.0900140	2.9008940	3.3288000	H	-2.9332730	1.0258290	-1.7666340
H	1.2554070	1.7774040	-1.8686140	H	-1.7696010	-0.2954150	-1.7893500
H	0.2636550	3.2306310	-1.9132830	H	-3.3007690	-0.4584790	-2.6790720
H	1.9102500	3.2682950	-2.5722430	C	-3.0097820	-0.0509740	3.0524740
H	0.0597350	3.2658100	3.3876680	H	-1.9443240	-0.2826050	3.1470250
H	1.0509430	1.8124130	3.4382910	H	-3.1029240	1.0385350	3.0350140
H	1.6509110	3.3125770	4.1705320	H	-3.5346610	-0.4408840	3.9270370
I	-0.5388340	1.7052510	0.7203630				

3.2.2 Structures optimized at the M06-2X/6-31G(d) level of theory

4_{trunc}

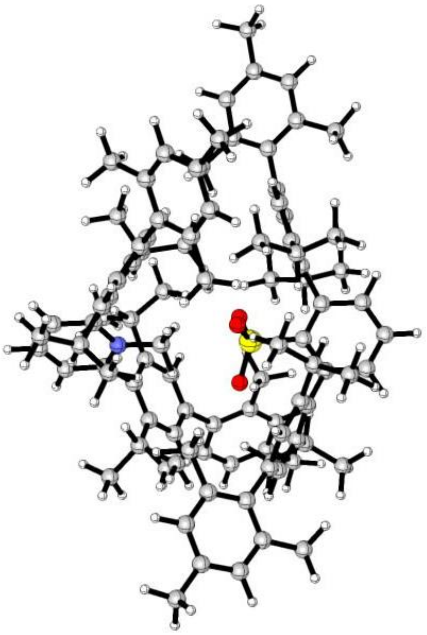

N	0.4940640	1.1208190	3.2893370
C	1.3974230	0.4448950	4.0520790
C	1.5119900	0.7546960	5.3996990
H	2.2473360	0.2155900	5.9855730
C	0.6617480	1.6951760	5.9699780
C	-0.3048400	2.3006140	5.1804500
H	-1.0150460	3.0096800	5.5902000
C	-0.3855010	2.0106630	3.8211840
C	0.4487920	0.8068990	1.8418480
H	-0.1330900	1.5557730	1.3180480
H	-0.0096850	-0.1769170	1.7086610
H	1.4612400	0.7664380	1.4416830
C	2.1352830	-0.6979320	3.4440190
C	3.3765420	-0.5162630	2.8158860
C	4.0238340	-1.6424240	2.3127640
H	4.9852100	-1.5335920	1.8180330
C	3.4440490	-2.9093070	2.3706960
C	2.1967700	-3.0503290	2.9758630
H	1.7381880	-4.0357970	3.0037740
C	1.5197950	-1.9607770	3.5170440
C	4.0028520	0.8577920	2.6430200
H	3.2304710	1.6149580	2.8444170
C	4.4944080	1.0720560	1.2046500
H	5.3616260	0.4382080	0.9881410
H	4.8097130	2.1107020	1.0575480
H	3.7164190	0.8249550	0.4718930
C	5.1343920	1.0690530	3.6559320
H	4.7734940	0.9776920	4.6862740
H	5.5839720	2.0592930	3.5321370
H	5.9190400	0.3188880	3.5081290
C	4.1127350	-4.0830270	1.7395460
C	3.5699550	-4.6213040	0.5580280
C	4.2126450	-5.7077360	-0.0345740
H	3.8044980	-6.1186060	-0.9562700
C	5.3644430	-6.2733370	0.5131770
C	5.8803020	-5.7208590	1.6832090
H	6.7786220	-6.1484420	2.1244200
C	5.2727790	-4.6301030	2.3061770
C	2.3480930	-4.0120890	-0.0829130
H	1.4460060	-4.1567350	0.5203230
H	2.1673210	-4.4591280	-1.0660730
H	2.4475390	-2.9271520	-0.2037560
C	6.0173590	-7.4672520	-0.1362490
H	5.4896970	-8.3919610	0.1231280
H	7.0561470	-7.5766000	0.1867080
H	6.0058980	-7.3797760	-1.2265850
C	5.8594850	-4.0619220	3.5763750
H	6.3074550	-3.0760580	3.4094790
H	6.6380040	-4.7199900	3.9705780
H	5.0916460	-3.9355550	4.3463200
C	0.1458520	-2.1699440	4.1358300
H	-0.2994380	-1.1898910	4.3509260
C	-0.7969130	-2.8938300	3.1657490
H	-0.7673580	-2.4458860	2.1654470
H	-1.8252000	-2.8639130	3.5456310
H	-0.5164940	-3.9484290	3.0651270
C	-1.4135060	2.6563110	2.9583390
C	-2.4780770	1.8889840	2.4389080
C	-3.4227510	2.5357040	1.6459040
H	-4.2339870	1.9598980	1.2079910
C	-3.3363860	3.8965200	1.3522730
C	-2.2780750	4.6280390	1.8863310
H	-2.2029660	5.6863200	1.6477180
C	-1.2975240	4.0309320	2.6779880
C	-2.6452180	0.3978130	2.6961680
H	-1.7396760	0.0155210	3.1818910
C	-2.8360610	-0.3793950	1.3862640
H	-3.8080390	-0.1525050	0.9325460
H	-2.8074370	-1.4579820	1.5680960
H	-2.0578520	-0.1526740	0.6505810
C	-3.8070730	0.1525570	3.6676260
H	-3.6601500	0.6831420	4.6146670
H	-3.9023180	-0.9176100	3.8802060
H	-4.7525640	0.4948980	3.2315350
C	-4.3230350	4.5320840	0.4334630
C	-5.6647770	4.6726620	0.8239660
C	-6.5687810	5.2552210	-0.0630590
H	-7.6058790	5.3751800	0.2442740
C	-6.1786990	5.6862760	-1.3298560
C	-4.8420880	5.5366880	-1.6948790
H	-4.5196410	5.8675590	-2.6801960
C	-3.9018800	4.9708960	-0.8340100
C	-6.1335710	4.2227820	2.1873970
H	-6.1952590	3.1309500	2.2544280
H	-7.1265730	4.6244870	2.4038270
H	-5.4484670	4.5526380	2.9744060
C	-7.1814990	6.2725530	-2.2906760
H	-8.0015340	6.7624800	-1.7585850
H	-7.6182190	5.4912940	-2.9227410
H	-6.7146790	7.0074790	-2.9521750
C	-2.4649980	4.8351390	-1.2804210
H	-1.8264010	5.6017760	-0.8260160
H	-2.3890590	4.9457810	-2.3653880
H	-2.0452990	3.8615670	-1.0092980
C	-0.1056270	4.8710240	3.1134200
H	0.5891670	4.2383300	3.6761040
C	0.6544230	5.3899090	1.8852080
H	0.9307990	4.5680010	1.2174210
H	1.5676280	5.9105820	2.1913840
H	0.0426730	6.0951080	1.3127070
C	-0.5293090	6.0242560	4.0291830
H	-1.2117650	6.7054130	3.5105030
H	0.3445420	6.6022710	4.3456340
H	-1.0427650	5.6576940	4.9239960
S	0.4511540	-0.8093210	-0.8154920
O	1.8813410	-0.6386980	-0.4792740
O	-0.3124030	0.4642090	-0.7954340
O	-0.1846030	-1.8856510	-0.0222410
C	0.3597250	-1.3457240	-2.5373270
C	1.3501360	-0.9639770	-3.4597870
C	1.2764090	-1.4504520	-4.7677280
H	2.0458780	-1.1554070	-5.4762280
C	0.2104170	-2.2439400	-5.1737930
C	-0.8248930	-2.5054540	-4.2845190
H	-1.7053200	-3.0522340	-4.6119280
C	-0.7791510	-2.0476920	-2.9619520
C	2.3662230	0.0963690	-3.1574070
C	3.6479250	-0.2220880	-2.6798000
C	4.5235350	0.8138170	-2.3707040
H	5.5025850	0.5872810	-1.9555160
C	4.1587240	2.1537820	-2.5268700
C	2.9024020	2.4432370	-3.0499310
H	2.6242350	3.4843500	-3.2007770
C	1.9896610	1.4338490	-3.3653830
C	4.0707940	-1.6677860	-2.4946030
H	3.1578120	-2.2698210	-2.4256110
C	4.8708330	-2.1456310	-3.7129710
H	4.2853490	-2.0569130	-4.6337960
H	5.1688190	-3.1930640	-3.5943980
H	5.7793850	-1.5432590	-3.8329390
C	4.8558890	-1.8923580	-1.2025630
H	5.8306000	-1.3900550	-1.2243760
H	5.0413300	-2.9612280	-1.0500220
H	4.2784480	-1.5118000	-0.3549310
C	5.0816710	3.2431110	-2.0983540
C	4.6983990	4.1274280	-1.0747190
C	5.5932080	5.1170470	-0.6581910
H	5.2954890	5.7901970	0.1442540
C	6.8474430	5.2678820	-1.2396770
C	7.2031680	4.3922110	-2.2659210
H	8.1756840	4.5009060	-2.7435740
C	6.3474290	3.3839300	-2.7019180
C	3.3446230	4.0475780	-0.4045980
H	2.6012250	4.6521590	-0.9387580
H	3.4079090	4.4365790	0.6172000
H	2.9587990	3.0250550	-0.3715990
C	7.7941160	6.3537920	-0.7943080
H	7.9131440	7.1161750	-1.5719770
H	8.7887830	5.9497620	-0.5806790
H	7.4295120	6.8518670	0.1080450
C	6.7848540	2.4798360	-3.8306160
H	7.1236620	1.5049300	-3.4639080
H	7.6134290	2.9325100	-4.3818920
H	5.9639120	2.2886810	-4.5270890
C	0.6398130	1.8235920	-3.9511050
H	0.0186230	0.9254930	-4.0182480
C	-0.1149960	2.8235850	-3.0703450
H	-0.2561840	2.4083780	-2.0681130
H	-1.0996350	3.0299810	-3.5076450

H	0.4173660	3.7798690	-2.9931360
C	0.8178120	2.3629580	-5.3758890
H	1.4331140	3.2700670	-5.3757650
H	-0.1534450	2.6113250	-5.8175890
H	1.3090120	1.6240950	-6.0173970
C	-2.0266950	-2.2623630	-2.1492930
C	-3.0148200	-1.2605660	-2.1346670
C	-4.2673180	-1.5665460	-1.5919620
H	-5.0561500	-0.8170860	-1.6075340
C	-4.5564150	-2.8249440	-1.0685910
C	-3.5363820	-3.7771350	-1.0344170
H	-3.7528730	-4.7579760	-0.6155420
C	-2.2702680	-3.5104620	-1.5517080
C	-2.7943180	0.1027640	-2.7787670
H	-1.7175010	0.2491090	-2.8989340
C	-3.2859580	1.2662890	-1.9123610
H	-4.3535590	1.1924600	-1.6739560
H	-3.1417860	2.2117800	-2.4477910
H	-2.7130750	1.3109920	-0.9811500
C	-3.4440840	0.1374650	-4.1678740
H	-3.0572060	-0.6658650	-4.8033510
H	-3.2428070	1.0941420	-4.6636780
H	-4.5315100	0.0156030	-4.0914870
C	-5.9409860	-3.1820310	-0.6327860
C	-6.9357110	-3.3647520	-1.6116130
C	-8.2251270	-3.7186790	-1.2177510
H	-8.9889420	-3.8613130	-1.9803290
C	-8.5581810	-3.9043840	0.1230610
C	-7.5605100	-3.7226820	1.0770340
H	-7.8005730	-3.8593940	2.1299810
C	-6.2582500	-3.3664010	0.7224170
C	-6.6192650	-3.2017030	-3.0791500
H	-6.4371790	-2.1537650	-3.3396920
H	-7.4469370	-3.5645770	-3.6941530
H	-5.7138540	-3.7536800	-3.3500350
C	-9.9513600	-4.3215500	0.5216320
H	-10.7059190	-3.8251910	-0.0957090
H	-10.1539220	-4.0789620	1.5684180
H	-10.0883150	-5.4020010	0.3998120
C	-5.2309770	-3.1366660	1.8038060
H	-4.2841320	-3.6410600	1.5885570
H	-5.6011490	-3.4885810	2.7708980
H	-5.0062290	-2.0684670	1.8946140
C	-1.1850610	-4.5699700	-1.4720170
H	-0.2320470	-4.0801940	-1.6980350
C	-1.4180920	-5.6852550	-2.4973930
H	-2.3777360	-6.1828960	-2.3151270
H	-0.6271300	-6.4406340	-2.4339040
H	-1.4314110	-5.2898740	-3.5178560
C	-1.0709430	-5.1422990	-0.0567810
H	-0.9206510	-4.3284660	0.6584400
H	-0.2203320	-5.8300740	0.0065050
H	-1.9688710	-5.7021710	0.2284650
H	0.7329760	1.9311420	7.0268610
H	0.1606500	-2.6141580	-6.1933010
C	0.2560210	-2.9173600	5.4699570
H	-0.7346140	-3.0625690	5.9126610
H	0.7080540	-3.9033550	5.3188960
H	0.8787260	-2.3698460	6.1857160

6_{trunc}

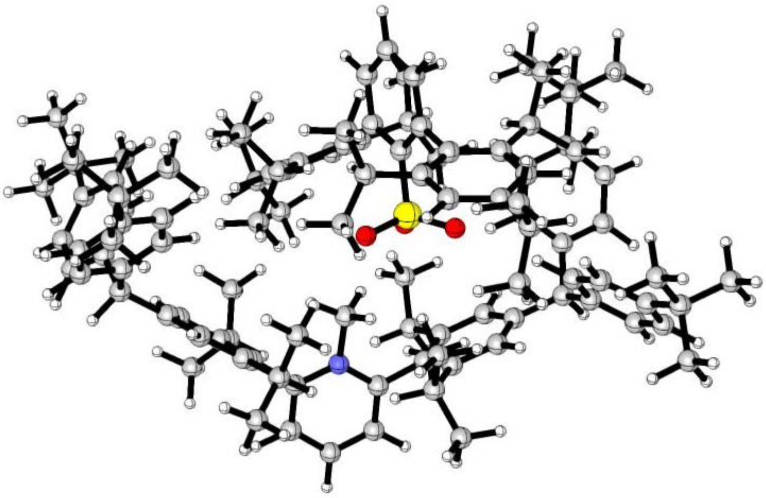

N	-0.8950550	-0.3575840	3.7349330
C	-2.1144180	-0.0912350	4.2721050
C	-2.3078840	-0.2988320	5.6335980
H	-3.2857620	-0.0790330	6.0461060
C	-1.2703300	-0.7743950	6.4239840
C	-0.0361220	-1.0304480	5.8407440
H	0.8083600	-1.3909960	6.4163070
C	0.1475710	-0.8116510	4.4817530
C	-0.6328500	-0.1148370	2.2963720
H	-0.2855520	-1.0313730	1.8115010
H	0.1482940	0.6368940	2.2025940
H	-1.5354970	0.2498780	1.8270630
C	-3.2341530	0.3984450	3.4162520
C	-3.2878050	1.7484920	3.0236440
C	-4.3908580	2.1854010	2.2912320
H	-4.4406510	3.2285300	1.9921800
C	-5.4104530	1.3166780	1.8988210
C	-5.3139250	-0.0184710	2.2876860
H	-6.0827140	-0.7180410	1.9762610
C	-4.2467230	-0.5002700	3.0450180
C	-2.1708920	2.7336310	3.3310560
H	-1.3762350	2.2067230	3.8758710
C	-1.5582410	3.2786440	2.0334010
H	-1.1792350	2.4781040	1.3887450
H	-0.7252380	3.9526680	2.2597870
H	-2.3000650	3.8470780	1.4601620
C	-2.6638130	3.8696470	4.2346420
H	-3.4430470	4.4541500	3.7347400
H	-1.8396190	4.5477040	4.4765400
H	-3.0802370	3.4822570	5.1699440
C	-6.6247760	1.8134700	1.0967360
C	-6.3368330	3.0103890	0.2037580
C	-5.1776440	3.1066450	-0.5660640
H	-4.4194490	2.3330050	-0.4937310
C	-4.9637970	4.1874250	-1.4187460
H	-4.0448210	4.2119140	-1.9947710
C	-5.8991130	5.2180920	-1.5340370
C	-7.0606790	5.1159980	-0.7573780
H	-7.8160950	5.8957330	-0.8118280
C	-7.2760910	4.0356330	0.0919540
H	-8.1922210	3.9899600	0.6764230
C	-5.6970630	6.4246400	-2.4535630
C	-4.3707940	6.3510450	-3.2176640
H	-3.5119830	6.3322770	-2.5381160
H	-4.2694220	7.2319830	-3.8595290
H	-4.3228870	5.4640360	-3.8581920
C	-5.7004710	7.7121070	-1.6123240
H	-6.6453340	7.8406470	-1.0752770
H	-5.5591150	8.5856580	-2.2585010
H	-4.8914510	7.6953620	-0.8748500
C	-6.8434010	6.4815850	-3.4772950
H	-6.8636320	5.5729800	-4.0875450
H	-6.7118540	7.3415790	-4.1434420
H	-7.8170740	6.5814460	-2.9877820
C	-7.2553040	0.6569320	0.3289170
C	-6.7128020	0.1975730	-0.8750190
H	-5.9185350	0.7563550	-1.3611810
C	-7.1674460	-0.9778040	-1.4549590
H	-6.7051180	-1.3117960	-2.3806320
C	-8.1908710	-1.7403950	-0.8733440
C	-8.7683330	-1.2442100	0.2961550
H	-9.5804280	-1.7800640	0.7758970
C	-8.3075360	-0.0648950	0.8850170
H	-8.7557430	0.2760030	1.8163090
C	-8.6039180	-3.0645880	-1.5183980
C	-7.3900440	-4.0115110	-1.5215820
H	-6.5423660	-3.5796310	-2.0639750
H	-7.6481150	-4.9627660	-2.0010210
H	-7.0602620	-4.2215100	-0.4976170
C	-9.7472950	-3.7446580	-0.7594670
H	-9.4644460	-3.9765360	0.2728720
H	-10.0064070	-4.6863960	-1.2537150
H	-10.6456050	-3.1183130	-0.7400920
C	-9.0630290	-2.8166760	-2.9649140
H	-9.9202970	-2.1361360	-2.9882260
H	-9.3601800	-3.7618070	-3.4326810
H	-8.2667660	-2.3782470	-3.5735540
C	-4.2166760	-1.9804610	3.3952190
H	-3.2910780	-2.1989210	3.9409180
C	-5.3921910	-2.3502790	4.3079680
H	-5.3941220	-1.7480440	5.2225010
H	-5.3401560	-3.4064740	4.5899050
H	-6.3467510	-2.1847580	3.7971000
C	-4.2116950	-2.8464360	2.1294800
H	-5.1025430	-2.6587620	1.5179150
H	-4.2085830	-3.9080240	2.3987940
H	-3.3330920	-2.6467020	1.5060190
C	1.4631410	-1.0324900	3.8223850
C	2.3319630	0.0564550	3.6709600
C	3.5953860	-0.1802600	3.1375030
H	4.2801560	0.6535030	3.0412780
C	3.9866480	-1.4392910	2.6957650
C	3.0773350	-2.4936470	2.8134920
H	3.3578740	-3.4722000	2.4300470
C	1.8133010	-2.3172900	3.3707580
C	1.9424770	1.4851960	4.0164740
H	0.8696670	1.5115130	4.2554200
C	2.1753120	2.4196830	2.8176460
H	3.2461490	2.5429860	2.6238620
H	1.7601610	3.4112450	3.0286470
H	1.7299920	2.0325280	1.8932250
C	2.6956890	1.9795130	5.2567580
H	2.4907150	1.3457540	6.1261620
H	2.4060480	3.0067590	5.5005990
H	3.7758710	1.9655550	5.0780420
C	5.3703860	-1.6892140	2.1021900
C	6.2045020	-0.4263830	1.9405080
C	7.4386730	-0.3024390	2.5758360
H	7.7870350	-1.0987420	3.2303310
C	8.2367330	0.8189380	2.3747550
H	9.1964280	0.8756120	2.8825710
C	7.8330070	1.8591860	1.5314760
C	6.5825580	1.7370900	0.9139180
H	6.2154920	2.5116090	0.2463950
C	5.7795410	0.6159470	1.1113290
H	4.8098690	0.5551560	0.6130890
C	8.7575410	3.0606850	1.3186620
C	8.9749730	3.7819430	2.6592110
H	8.0228200	4.1414710	3.0631530
H	9.6397650	4.6430100	2.5246210
H	9.4281530	3.1183490	3.4023580
C	10.1120980	2.5756610	0.7751010
H	10.6065570	1.8917720	1.4714490
H	10.7803780	3.4285880	0.6101000
H	9.9804690	2.0508700	-0.1766380
C	8.1742790	4.0628690	0.3188820
H	8.0050040	3.5974960	-0.6589200
H	8.8723420	4.8951900	0.1795530
H	7.2239400	4.4770350	0.6720480
C	5.3368950	-2.4162830	0.7589180
C	6.4658450	-3.1251250	0.3367120
H	7.3192860	-3.2220890	1.0054260
C	6.5211880	-3.6846940	-0.9321070
H	7.4131190	-4.2343840	-1.2223220
C	5.4613910	-3.5422790	-1.8410600
C	4.3352260	-2.8468970	-1.3992970
H	3.4784250	-2.7024970	-2.0514650
C	4.2635450	-2.3000980	-0.1174440
H	3.3683860	-1.7611630	0.1746260
C	5.5632500	-4.1552620	-3.2409710
C	6.8710790	-3.7221580	-3.9240450
H	6.9067400	-2.6346140	-4.0472520
H	6.9419610	-4.1795070	-4.9175590
H	7.7535930	-4.0266910	-3.3540490
C	5.5479660	-5.6884140	-3.1180050
H	6.3826270	-6.0451750	-2.5055310
H	5.6282190	-6.1525920	-4.1080420
H	4.6176760	-6.0314660	-2.6522450
C	4.3979860	-3.7231590	-4.1365310
H	3.4382410	-4.0752510	-3.7457470
H	4.5249510	-4.1464830	-5.1388060
H	4.3462930	-2.6317280	-4.2318960
C	0.8425510	-3.4857860	3.4515360
H	-0.1380860	-3.0992110	3.7626920
C	0.6523400	-4.1594770	2.0873480
H	0.3806670	-3.4360920	1.3114430
H	-0.1263790	-4.9274920	2.1521070
H	1.5752510	-4.6544730	1.7672790
C	1.2961290	-4.4974090	4.5106950
H	2.2755200	-4.9106170	4.2469330
H	0.5863120	-5.3279130	4.5812150
H	1.3852040	-4.0329560	5.4988270
S	1.1851330	-0.2174900	-0.5475840
O	0.9605690	-1.5835590	-0.0181930
O	2.5380300	0.2922950	-0.2716860
O	0.1085650	0.7368750	-0.1684400
C	1.0343440	-0.3644550	-2.3545290
C	1.7083210	0.5562810	-3.1821810
C	1.5411700	0.4716050	-4.5678680
H	2.0446470	1.2029620	-5.1933620
C	0.7324080	-0.5015310	-5.1387350
C	0.0438510	-1.3781430	-4.3135200
H	-0.6246320	-2.1223940	-4.7384610
C	0.1618330	-1.3143780	-2.9187390
C	2.5482280	1.6929590	-2.6768110
C	3.9505020	1.5958080	-2.6705390
C	4.6917690	2.6950830	-2.2340030
H	5.7784330	2.6355650	-2.2215430
C	4.0844190	3.8730460	-1.8044450
C	2.6941760	3.9480960	-1.8391710
H	2.2040720	4.8656260	-1.5196310
C	1.9105980	2.8783580	-2.2698960
C	4.6632950	0.3393360	-3.1523580
H	3.9611680	-0.4970950	-3.0477670
C	5.9070770	-0.0113340	-2.3346380
H	6.6941170	0.7445260	-2.4402850

H	6.3199470	-0.9652910	-2.6783660
H	5.6705930	-0.1216790	-1.2756570
C	5.0499160	0.4701670	-4.6328670
H	4.1797130	0.6346070	-5.2732300
H	5.5596690	-0.4373220	-4.9779450
H	5.7349050	1.3152110	-4.7693380
C	4.9120130	5.0418900	-1.3057700
H	5.9685470	4.7530200	-1.3854360
C	4.6132140	5.3408140	0.1681110
H	3.5694440	5.6494730	0.2951460
H	5.2522600	6.1499710	0.5399030
H	4.7741860	4.4545500	0.7910250
C	4.7025680	6.2938120	-2.1646910
H	4.9295530	6.0913270	-3.2153600
H	5.3446290	7.1123310	-1.8217600
H	3.6633310	6.6356520	-2.1057610
C	0.3989740	3.0389170	-2.3441120
H	-0.0483640	2.0419860	-2.3470640
C	-0.1755510	3.7712860	-1.1301360
H	0.2124930	3.3294690	-0.2095240
H	-1.2686450	3.6793880	-1.1216430
H	0.0634250	4.8413580	-1.1444990
C	0.0033140	3.7450780	-3.6470790
H	0.4552470	4.7427330	-3.6957280
H	-1.0854860	3.8599050	-3.7108260
H	0.3391390	3.1788510	-4.5217590
C	-0.7509210	-2.2534580	-2.1786850
C	-0.4346250	-3.6185580	-2.0652200
C	-1.3832660	-4.4950060	-1.5398540
H	-1.1273530	-5.5495140	-1.4510390
C	-2.6480360	-4.0639920	-1.1404480
C	-2.9393410	-2.7087580	-1.2612820
H	-3.9223300	-2.3493920	-0.9519380
C	-2.0083620	-1.7930670	-1.7597920
C	0.9168770	-4.1695300	-2.4846080
H	1.5123630	-3.3344130	-2.8698840
C	0.7736280	-5.2017710	-3.6093270
H	0.1743320	-6.0586630	-3.2820500
H	1.7547320	-5.5819010	-3.9153420
H	0.2851950	-4.7661020	-4.4870610
C	1.6610410	-4.7551800	-1.2811990
H	1.8079470	-3.9816390	-0.5221440
H	2.6450920	-5.1350920	-1.5803860
H	1.0974710	-5.5846890	-0.8367870
C	-3.6891680	-5.0393810	-0.6256870
H	-4.5106580	-4.4435430	-0.2042910
C	-3.1434370	-5.9448560	0.4829290
H	-2.3808420	-6.6279330	0.0934320
H	-3.9451930	-6.5560540	0.9117360
H	-2.6852620	-5.3569660	1.2838780
C	-4.2611830	-5.8818560	-1.7725940
H	-4.6711350	-5.2446720	-2.5618940
H	-5.0565960	-6.5447860	-1.4133890
H	-3.4749900	-6.5021200	-2.2179080
C	-2.3858630	-0.3236360	-1.8071820
H	-1.4781200	0.2568850	-1.9792240
C	-3.3708770	-0.0259070	-2.9420570
H	-2.9317410	-0.2550630	-3.9183780
H	-3.6594100	1.0330400	-2.9358070
H	-4.2817910	-0.6274930	-2.8291730
C	-2.9523450	0.1209510	-0.4526790
H	-4.0333870	-0.0509280	-0.3832970
H	-2.7474750	1.1831180	-0.2775620
H	-2.4803250	-0.4449720	0.3515030
H	0.6185420	-0.5585020	-6.2170290
H	-1.4206030	-0.9363910	7.4865260
H	-7.3663320	2.1418700	1.8391270
H	5.9048820	-2.3387680	2.8105390

3.2.3 Structure optimized with the PM6 semi-empirical method

8

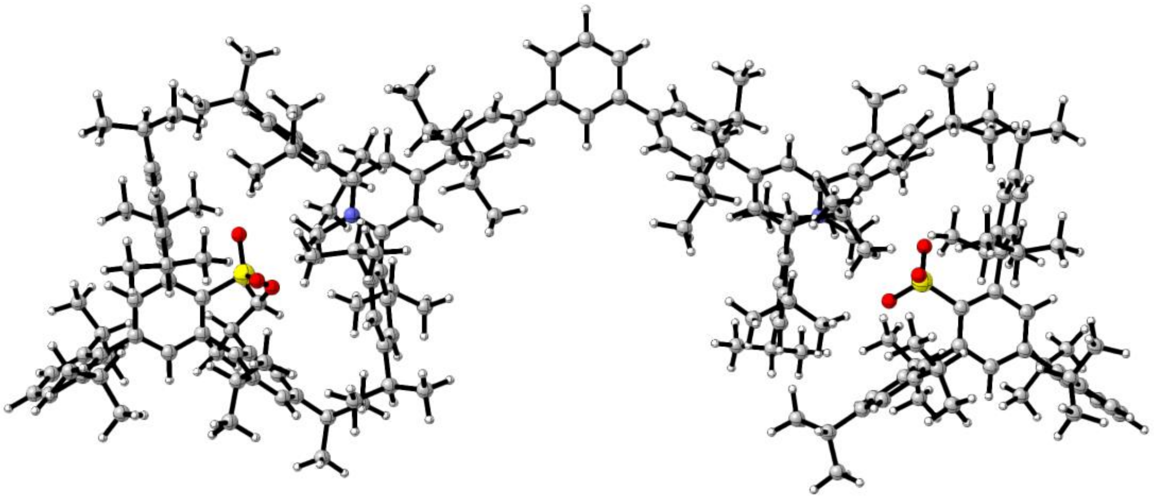

S	12.4632140	-0.7867360	0.0528080
S	-12.4752630	-0.7982350	0.0278280
O	11.3399240	-1.5965000	-0.5078710
O	12.1314740	-0.3846900	1.4570980
O	12.6360990	0.4177090	-0.8102350
O	-12.7052910	0.6279280	-0.3484170
O	-11.5487130	-1.3963620	-0.9781270
O	-11.8684760	-0.8591100	1.3967560
N	8.6455490	1.6039730	-0.1315780
N	-8.5547830	1.4090740	-0.1511470
C	9.4271930	-7.8098740	0.7698910
C	7.9821210	-6.0044190	-0.2558240
C	9.0423480	-6.3237760	0.8061640
C	6.0111670	-3.9103600	-4.5146130
C	10.2910300	-5.4747590	0.6397650
C	10.8664490	-5.2639230	-0.6173030
C	12.8863440	-5.6190110	-2.8354800
C	10.8946360	-4.9194050	1.7743890
C	7.3109910	-3.9049410	-3.6990330
C	16.7753380	-5.4652040	-2.8005580
C	8.5338890	-4.1403310	-4.5892760
C	12.0250400	-4.4870420	-0.7540920
C	19.9015410	-5.6005970	0.3621170
C	17.4713930	-4.9925180	3.8714080
C	12.6232640	-4.2764610	-2.1374040
C	19.3157690	-5.3063440	-0.8697060
C	11.7011390	-3.3738270	-2.9605750
C	19.3190110	-5.1408070	1.5439120
C	13.1041400	-4.7436810	3.8630990
C	12.0578000	-4.1441540	1.6637570
C	12.6175490	-3.9131650	0.3911220
C	18.1442360	-4.5380290	-0.9345640
C	18.1355360	-4.3892720	1.5080970
C	7.4395920	-2.5980610	-2.9362680
C	15.0587670	-3.8990830	0.3115320
C	17.5174220	-4.2256810	-2.2835330
C	7.4035520	-2.6070480	-1.5387390
C	17.5519750	-4.0838970	0.2614300
C	12.7076280	-3.5936160	2.9244550
C	17.5123380	-3.8929070	2.8024410
C	13.8786070	-3.1354190	0.2597490
C	8.7041710	-2.2801780	1.1830170
C	16.3066920	-3.2775250	0.2074230
C	11.7785010	-2.5903690	3.6134720
C	6.1710870	-2.0840180	1.2316810
C	18.5640920	-3.7357760	-3.2925630
C	7.5912760	-1.3769700	-3.6111630
C	7.4795240	-1.4168680	-0.8039170
C	7.4862640	-1.4829030	0.7155070
C	18.2795750	-2.6590240	3.2956870
C	13.9364120	-1.7421400	0.0931470
C	16.3728690	-1.8898640	0.0518940
C	7.6724110	-0.1738600	-2.9072460
C	7.5702690	-0.1897800	-1.4956630
C	15.1936080	-1.1252510	-0.0044220
C	7.0619890	1.2340950	-4.9210180
C	7.9238680	1.1258150	-3.6568760
C	16.8533970	0.9310450	3.1231270
C	7.5192240	1.0921420	-0.7638350
C	9.4187430	1.2149010	-3.9870970
C	9.9097070	0.8568730	-0.1223050
C	15.2841680	0.0240580	-2.7092340
C	14.0792340	0.5172180	-3.5177100
C	6.3171390	1.8114540	-0.7197040
C	3.5428940	1.2651660	1.8537920
C	15.3970300	0.3374370	-0.1744300
C	9.9178250	0.7447270	3.4641060
C	15.5619470	0.5891470	2.3635580
C	16.5721670	0.0063320	-3.5449830
C	15.4665660	0.8899490	-1.4712830
C	15.6009600	1.1578870	0.9529290
C	9.0808100	2.0180930	3.3106600
C	6.2362100	3.0459150	-0.0650120
C	14.3201610	1.0960450	3.1003280
C	4.3341490	2.5546560	2.1063530
C	8.5855400	2.8241770	0.5366360
C	5.2227720	4.2437370	-3.4313260
C	4.9618200	3.7966220	-0.0332710
C	8.5514450	2.5171590	4.6607650
C	4.0345590	3.5560290	1.0018380
C	7.3881610	3.5458850	0.5578570
C	5.6675480	4.9967550	-2.1713110
C	4.6750550	4.7311650	-1.0501980
C	15.7159410	2.2604810	-1.6258190
C	9.9391510	3.0645440	2.6174260
C	15.8544050	2.5249160	0.7720840
C	2.8114260	4.2378800	0.9956050
C	9.7723150	3.3477770	1.2430330
C	4.0266170	3.1398080	3.4895030
C	3.4466040	5.4027130	-1.0341690
C	2.5148550	5.1583790	-0.0172720
C	15.8956290	3.0839690	-0.5097800
C	10.9558100	3.7107650	3.3271970
C	10.7091160	4.1591590	0.5661100
C	5.8238570	6.4976290	-2.4420120
C	10.6724550	4.3063740	-0.9468600
C	11.8176510	3.4848020	-1.5436390
C	11.8323590	4.5943910	2.6833090
C	11.7181560	4.7905090	1.3010150
C	16.1403480	4.5748480	-0.6660120
C	17.5419310	4.8238150	-1.2389190
C	10.7497790	5.7789260	-1.3706350
C	12.9058690	5.3014990	3.4916310
C	15.0559440	5.2175620	-1.5410370
C	14.2910610	4.8187440	3.0538960
C	12.7684800	6.8233670	3.3589260
C	-1.2104120	5.8248940	-0.0252680
C	-1.2367720	7.2280170	0.0015020
C	0.0173240	5.1476530	-0.0317000
C	-0.0386350	7.9465060	0.0176170
C	1.2170810	5.8731770	-0.0132970
C	1.1872540	7.2763230	0.0093180
C	-15.9990110	-0.0794670	4.2444040
C	-13.5071810	0.1848080	3.8209230
C	-14.8741670	-0.1134280	3.1983510
C	-17.1877720	4.8104860	2.4287480
C	-14.6829310	5.1845980	2.5051460
C	-15.8548520	4.6204920	1.6919690
C	-17.1294460	1.3197220	-2.2813790
C	-14.6088080	1.4956050	-2.5645540
C	-15.7538690	0.9192610	-1.7280920
C	-15.1681160	0.8671810	2.0729600
C	-15.3441890	2.2251100	2.3764100
C	-15.6448520	3.1503210	1.3719340
C	-15.7756740	2.7121900	0.0489110
C	-15.6008570	1.3611820	-0.2798630
C	-15.2904950	0.4335830	0.7376650
C	-18.9823730	-2.2659930	-2.6730260
C	-17.9556120	-4.4893010	-3.3376290
C	-18.0405360	-3.4035600	-2.2568550
C	-18.2193560	-3.8447470	3.9349690
C	-16.5452010	-5.4957920	2.9863080
C	-17.3439250	-4.2110740	2.7295730
C	-18.4941580	-3.9936130	-0.9316710
C	-19.7011660	-4.7058290	-0.8667880
C	-20.1328550	-5.2572720	0.3397680
C	-19.3698590	-5.0966850	1.4974690
C	-18.1680490	-4.3753320	1.4630920
C	-17.7273190	-3.8303010	0.2392060
C	-13.9464020	-4.3247910	-3.9636440
C	-12.3377210	-2.3608780	-3.7868210
C	-13.2752040	-3.3077420	-3.0295040
C	-9.6930280	-7.8281070	-1.1765380
C	-8.2170480	-5.7651060	-1.1985880
C	-9.5260560	-6.3940150	-1.6941650
C	-12.7399340	-5.8494340	2.5005060
C	-11.4791260	-3.6538700	2.6674690
C	-12.5290810	-4.4527720	1.8942420
C	-12.5060990	-4.0219960	-1.9277700
C	-11.4320050	-4.8539190	-2.2719080
C	-10.6999970	-5.5219450	-1.2840900
C	-11.0555020	-5.3688640	0.0600850
C	-12.1383390	-4.5575760	0.4275650
C	-12.8581780	-3.8693610	-0.5700780
C	-15.1900250	-1.0138000	0.4106580
C	-16.4071600	-1.7173980	0.4626630
C	-16.4436910	-3.0865940	0.1835770
C	-15.2599230	-3.7516560	-0.1485930
C	-14.0426440	-3.0489450	-0.2042260
C	-13.9980060	-1.6724670	0.0685460
C	-11.8630210	3.1148550	1.5181600
C	-10.5054390	5.2601160	1.6569310
C	-10.6226200	3.8640090	1.0296960
C	-14.3023990	5.1660690	-2.5725670
C	-12.6194410	6.9979380	-3.0496140
C	-12.9317320	5.4996110	-3.1667970
C	-9.6574380	1.1591170	-3.9718590
C	-8.4537260	3.2322580	-4.8003480
C	-8.9305840	2.4484280	-3.5714180
C	-10.6665520	3.9556540	-0.4875290
C	-11.7041740	4.6667810	-1.1022610
C	-11.8292600	4.6993880	-2.4952520
C	-10.9147460	3.9855410	-3.2846850
C	-9.8609620	3.2783430	-2.6999580
C	-9.7071380	3.3060560	-1.2948680
C	-6.3167190	6.0451200	1.9424760
C	-4.9344250	4.5742660	3.4755810

C	-5.6133950	4.6919770	2.1060670
C	-3.9824980	3.1795410	-3.6367390
C	-3.3357960	1.2452490	-2.1311890
C	-4.2124770	2.4943960	-2.2846300
C	-4.6131940	4.5001460	0.9767750
C	-3.4149000	5.2247440	0.9817070
C	-2.4785650	5.0590450	-0.0465260
C	-2.7430040	4.1693550	-1.0953390
C	-3.9434830	3.4493510	-1.1321100
C	-4.8775320	3.6140810	-0.0882950
C	-8.4594490	-2.1158470	-1.9253060
C	-5.9235150	-2.2049060	-1.9508990
C	-7.1650900	-1.5240770	-1.3605400
C	-8.1930880	-4.8519850	3.6026350
C	-5.6924620	-4.4440820	3.5715710
C	-6.9896610	-4.4381300	2.7517280
C	-9.6963950	0.2843780	3.4342050
C	-7.4261250	0.5646980	4.5322060
C	-8.1927250	0.4655390	3.2075250
C	-7.2036180	-1.6465820	0.1546780
C	-7.0456010	-2.9010950	0.7535680
C	-7.1991240	-3.0657270	2.1362080
C	-7.5495680	-1.9616290	2.9241440
C	-7.7008790	-0.6934360	2.3550140
C	-7.4570750	-0.5246770	0.9730860
C	-9.8085790	0.6455040	-0.1337730
C	-8.5103730	2.6908770	-0.6858150
C	-7.3072270	3.4086890	-0.6591650
C	-6.1454670	2.8522630	-0.1114050
C	-6.2129140	1.5544030	0.4136400
C	-7.4124190	0.8355480	0.3989540
H	0.0390630	4.0583350	-0.0506830
H	-2.1904970	7.7540470	0.0032540
H	8.5544210	-8.4526630	0.9161820
H	9.8861110	-8.0810650	-0.1877370
H	10.1559590	-8.0454490	1.5541060
H	7.0301320	-6.4890450	-0.0214470
H	8.2920340	-6.3454040	-1.2500180
H	5.8725520	-4.8696770	-5.0251690
H	8.5923160	-6.1051370	1.8094330
H	7.8063160	-4.9168420	-0.3257270
H	11.9575260	-6.1572570	-3.0492680
H	10.4074870	-5.6962650	-1.5028020
H	5.1386990	-3.7446810	-3.8731790
H	8.4999070	-5.1270780	-5.0610820
H	15.9864110	-5.7669610	-2.0999110
H	6.0088440	-3.1286770	-5.2820780
H	7.2588540	-4.7501640	-2.9624360
H	13.5083500	-6.2709810	-2.2128550
H	13.4047460	-5.4661440	-3.7872260
H	17.0038830	-5.9036780	3.4808640
H	10.4612910	-5.0866270	2.7574600
H	17.4480280	-6.3194640	-2.9282590
H	10.7730620	-3.8819350	-3.2591520
H	12.2284680	-5.2807030	4.2409580
H	20.8154880	-6.1903010	0.4013810
H	18.4716090	-5.2600500	4.2274570
H	16.2996900	-5.2668070	-3.7664730
H	13.7413510	-5.4700140	3.3471260
H	8.6170630	-3.3951360	-5.3866070
H	19.7719100	-5.6739260	-1.7866280
H	14.9969410	-4.9789000	0.4359630
H	19.7857520	-5.3681430	2.5002310
H	16.8902340	-4.6683240	4.7414680
H	7.3589580	-3.5621850	-0.9984950
H	9.4698150	-4.0856900	-3.9968380
H	12.1960030	-2.9981050	-3.8568360
H	8.5786640	-3.3619890	1.0710000
H	13.6581070	-4.3662410	4.7282300
H	11.3929980	-2.4980350	-2.3449130
H	6.0285050	-3.1125160	0.8804790
H	13.6106940	-3.7526080	-2.0431670
H	10.8003230	-3.0145530	3.8453520
H	16.7652200	-3.4002640	-2.1633780
H	16.4500190	-3.5831570	2.6091630
H	19.2650110	-4.5270760	-3.5777250
H	8.9501400	-2.0809000	2.2288940
H	9.6224500	-2.0365940	0.5950240
H	13.6472030	-3.0423490	2.6549040
H	6.1674250	-2.1185660	2.3270280
H	7.6671340	-1.3738210	-4.6976990
H	18.0839380	-3.3774280	-4.2094700
H	19.3344340	-2.8833050	3.4839210
H	12.2175870	-2.2106620	4.5389810
H	5.3055670	-1.4995470	0.9064980
H	11.6210810	-1.7128150	2.9485160
H	17.8488870	-2.2711330	4.2245630
H	19.1508460	-2.9072720	-2.8793430
H	18.2394930	-1.8526230	2.5522000
H	7.5714240	-0.4543960	1.1465820
H	7.3535840	0.4966390	-5.6787350
H	17.3397920	-1.3957500	-0.0267180
H	17.7383390	0.6418340	2.5462990
H	9.7360760	0.4099090	-4.6615310
H	15.0667440	-1.0322450	-2.4018200
H	16.8898980	0.4054740	4.0825300
H	6.0020000	1.0726550	-4.6984870
H	10.2569400	0.6334580	0.9278100
H	5.4350660	1.3882980	-1.2109610
H	13.2061450	0.6152480	-2.8374530
H	3.7915420	0.8281150	0.8799680
H	13.8116410	-0.1863080	-4.3081330
H	9.3144470	-0.1073810	3.7847860
H	15.4904760	-0.5297300	2.3163480
H	9.8627630	-0.1312400	-0.6500900
H	10.0412710	1.1197210	-3.0846040
H	16.4593440	-0.6401630	-4.4207610
H	10.4263480	0.4522900	2.5130910
H	3.7619630	0.5118660	2.6195440
H	7.1649380	2.2226700	-5.3815090
H	2.4612070	1.4403660	1.8689870
H	16.9315610	2.0026020	3.3320570
H	7.6607880	2.0000740	-3.0084920
H	9.6703400	2.1655290	-4.4661810
H	17.4163200	-0.3694320	-2.9562960
H	8.1884420	1.7736620	2.6810000
H	10.7583360	1.4231940	-0.6023350
H	5.1698730	3.1631980	-3.2537830
H	10.7346930	0.8662950	4.1847130
H	14.2770520	0.7194250	4.1238210
H	14.2522080	1.4956140	-3.9696210
H	7.8749500	1.7812650	5.1093050
H	16.8385340	1.0054100	-3.9042030
H	6.6786570	4.6110840	-1.8767050
H	5.4255860	2.2966670	2.0954180
H	5.9232300	4.4083460	-4.2580960
H	13.3981080	0.7420850	2.5854060
H	14.2674390	2.1938820	3.1305310
H	4.2331300	4.5693020	-3.7721870
H	4.3679300	2.4665090	4.2845240
H	8.0058800	3.4600860	4.5546960
H	9.3619860	2.6837710	5.3804890
H	2.9536540	3.3011100	3.6431620
H	15.7570560	2.6865550	-2.6250290
H	2.0851830	4.0556090	1.7880150
H	3.2079190	6.1156840	-1.8235140
H	16.0189680	3.1530690	1.6436620
H	7.3617000	4.5084410	1.0792090
H	9.7094070	3.9004250	-1.3475570
H	11.8463530	2.4372930	-1.1552320
H	11.0823680	3.4998000	4.3886740
H	4.5273320	4.1040110	3.6355140
H	6.6391350	6.6850410	-3.1505830
H	6.0529980	7.0476910	-1.5220120
H	11.7550000	3.4194500	-2.6317140
H	17.6585240	4.3691790	-2.2292790
H	4.9173200	6.9382330	-2.8721420
H	18.3126250	4.3884860	-0.5925020
H	12.7764920	5.0400020	4.5747940
H	12.8104100	3.8975070	-1.2907370
H	15.1973050	4.9794300	-2.6009870
H	14.3553090	3.7116210	3.0995640
H	16.0912670	5.0558560	0.3454760
H	10.6434860	5.8775180	-2.4565050
H	14.0538980	4.8422810	-1.2624500
H	12.4428440	5.4215910	0.7869490
H	9.9633140	6.3747920	-0.8968940
H	11.7122230	6.2306750	-1.1022200
H	17.7479110	5.8936180	-1.3375650
H	11.7677510	7.1595240	3.6511840
H	15.0792690	5.2258320	3.6931750
H	15.0560750	6.3057420	-1.4428670
H	14.5176470	5.1024510	2.0192320
H	12.9422670	7.1608880	2.3311760
H	13.4942450	7.3390670	3.9973520
H	-0.0604080	9.0358950	0.0369550
H	2.1192050	7.8397950	0.0271980
H	-16.9745110	-0.2547260	3.7779750
H	-15.8467760	-0.8521270	5.0043920
H	-16.0465420	0.8850440	4.7596140
H	-13.4203350	1.2154460	4.1685720
H	-13.2968860	-0.4817530	4.6603960
H	-12.7094070	0.0126150	3.0652660
H	-14.8303310	-1.1570320	2.7881590
H	-17.2027210	4.2656340	3.3795710
H	-17.3757150	5.8658010	2.6468000
H	-18.0244840	4.4340390	1.8291650
H	-13.7185960	4.8642360	2.0700330
H	-14.7032150	6.2768540	2.5308280
H	-14.6979070	4.8278540	3.5406380
H	-15.9076520	5.1934510	0.7299440
H	-17.2511110	2.4064330	-2.3274960
H	-17.2686470	0.9295360	-3.2944800
H	-17.9353520	0.9236750	-1.6541630
H	-13.6399880	1.2891700	-2.0556680
H	-14.5693030	1.0517070	-3.5597710
H	-14.6758300	2.5877930	-2.6713980
H	-15.6834850	-0.1985620	-1.7891010
H	-15.2452810	2.5583960	3.4065580
H	-16.0093720	3.4242940	-0.7382920
H	-19.0177560	-1.4870490	-1.9016420
H	-18.6446070	-1.7956570	-3.6023560
H	-20.0064970	-2.6185520	-2.8319860
H	-18.9395320	-4.8961580	-3.5920660
H	-17.5153590	-4.0913990	-4.2581590
H	-17.3293360	-5.3253420	-3.0047770
H	-17.0122090	-2.9670940	-2.1426640
H	-18.8728610	-4.6697460	4.2368300
H	-17.6006960	-3.5840260	4.8004840
H	-18.8567910	-2.9818240	3.7106470
H	-15.8657690	-5.7056590	2.1501230
H	-15.9348870	-5.4077000	3.8909970
H	-17.1995590	-6.3650760	3.1071360
H	-16.6107360	-3.3715990	2.5893860
H	-20.3026480	-4.8305410	-1.7647430
H	-21.0668900	-5.8146020	0.3787310
H	-19.7105720	-5.5347510	2.4334840
H	-14.6250820	-4.9801130	-3.4066480
H	-14.5301080	-3.8165480	-4.7374290
H	-13.2125910	-4.9610980	-4.4684690
H	-11.5857810	-2.8964200	-4.3692800
H	-12.8905810	-1.7017590	-4.4586030
H	-11.7979710	-1.7254800	-3.0522790
H	-14.0863730	-2.6795300	-2.5768740
H	-10.6437900	-8.2570190	-1.5139800
H	-8.8860390	-8.4752110	-1.5329590
H	-9.6916080	-7.8683310	-0.0818360
H	-8.1492820	-5.7948100	-0.1053790
H	-7.3449480	-6.2807140	-1.6073310
H	-8.1668050	-4.7012990	-1.4980650
H	-9.4835190	-6.4397750	-2.8135860
H	-11.8014280	-6.4079480	2.5722620
H	-13.1570740	-5.7753670	3.5096280
H	-13.4309610	-6.4428830	1.8923920
H	-11.4032490	-2.6202530	2.2586970
H	-11.7435140	-3.5571780	3.7221660
H	-10.4763920	-4.0984870	2.5977350
H	-13.5028510	-3.9021000	1.9846480
H	-11.1621150	-4.9660830	-3.3190100
H	-10.4858060	-5.8813090	0.8310020
H	-17.3235120	-1.1903270	0.7234350
H	-15.2769320	-4.8181410	-0.3667390
H	-12.1382880	2.2615480	0.8523080
H	-11.7239940	2.6901800	2.5150360
H	-12.7623900	3.7504290	1.5469430
H	-11.3612360	5.8954740	1.4012590
H	-10.4739650	5.1905400	2.7500970
H	-9.6001980	5.7759190	1.3227940
H	-9.7255280	3.2804800	1.3552110
H	-14.3927580	5.4881420	-1.5292950
H	-15.1092770	5.6413460	-3.1378360
H	-14.4826440	4.0714170	-2.5831250
H	-11.6484250	7.2389170	-3.4953900
H	-13.3801860	7.5986810	-3.5597450
H	-12.5919510	7.3243370	-2.0038200
H	-12.9597750	5.2348050	-4.2562930
H	-10.5353700	1.3614600	-4.5972710
H	-9.0028810	0.4834670	-4.5300170
H	-10.0300080	0.6107180	-3.0922290
H	-8.0040550	4.1890760	-4.5149450
H	-7.7065610	2.6619260	-5.3628320
H	-9.2769440	3.4498430	-5.4914520
H	-8.0164240	2.1619010	-2.9920840
H	-12.4453430	5.1719120	-0.4819310
H	-11.0371920	3.9657860	-4.3667100
H	-6.8256590	6.1166460	0.9744430
H	-7.0706520	6.1945620	2.7241890
H	-5.6123570	6.8823680	2.0051580
H	-4.2373670	5.3982180	3.6655690
H	-5.6758980	4.5876100	4.2830190
H	-4.3686250	3.6394660	3.5616730
H	-6.3961120	3.8903130	2.0578950
H	-2.9282160	3.4291750	-3.8014170
H	-4.2927470	2.5302400	-4.4635680
H	-4.5552260	4.1110200	-3.7144630
H	-3.5226690	0.7411700	-1.1760900
H	-3.5355960	0.5226130	-2.9308340
H	-2.2678300	1.4878990	-2.1705770
H	-5.2843030	2.1656940	-2.2611750
H	-3.2042980	5.9179280	1.7962670
H	-2.0113780	4.0445490	-1.8937530
H	-9.3737260	-1.6845990	-1.4504170
H	-8.5532090	-1.9511660	-3.0008090

H	-8.5394900	-3.2029810	-1.7462470
H	-5.9341960	-3.2888680	-1.7851800
H	-5.8733030	-2.0496710	-3.0343610
H	-5.0024060	-1.8125900	-1.5087190
H	-7.1189460	-0.4460580	-1.6579030
H	-9.1441070	-4.6850730	3.0570300
H	-8.1471400	-5.9119330	3.8701060
H	-8.2592400	-4.2801800	4.5340760
H	-5.7336040	-3.7287650	4.4006510

H	-5.5049620	-5.4337420	4.0023370
H	-4.8286280	-4.1799610	2.9519830
H	-6.8814960	-5.1913000	1.9271260
H	-9.9221520	-0.5781630	4.0714170
H	-10.1519320	1.1652710	3.8921370
H	-10.2494740	0.0831100	2.4842440
H	-6.3461150	0.6301940	4.3675640
H	-7.7383080	1.4504740	5.0965410
H	-7.6105290	-0.3035590	5.1761440

H	-8.0368250	1.4330300	2.6667300
H	-6.8245360	-3.7653820	0.1276660
H	-7.7307150	-2.0973150	3.9900220
H	-10.0927990	0.3231660	0.9093380
H	-10.6899680	1.2099830	-0.5353830
H	-9.7507900	-0.3077140	-0.7343840
H	-7.2907850	4.4200110	-1.0786070
H	-5.3233460	1.0870570	0.8479590

3.3 Crystallographic data

General procedure. The measurements for compound **10** were made on a *Rigaku Oxford Diffraction XtaLAB Synergy-S* diffractometer fitted with a *HyPix 6000HE* hybrid pixel array detector using Cu *K*α radiation (λ = 1.54184 Å) from a *PhotonJet* micro-focus X-ray source and an *Oxford Cryosystems Cryostream 800+* cooler. For all other compounds, the measurements were made on an a *Rigaku Oxford Diffraction SuperNova* diffractometer fitted with an Atlas CCD area-detector using Cu *K*α radiation from a micro-focus X-ray source and an *Oxford Instruments Cryojet XL* cooler. Data reduction was performed with *CrysAlisPro*.[34a-d] The intensities were corrected for Lorentz and polarization effects, and usually an empirical absorption correction using spherical harmonics[34] was applied. Where explicitly mentioned below, a numerical absorption correction[35] was applied instead. The space groups were determined from the systematic absences, packing considerations, a statistical analysis of intensity distribution, and the successful solution and refinement of the structures. Equivalent reflections, other than Friedel pairs for the structures with non-centrosymmetric space groups, were merged. The structures were solved by dual space methods using the *SHELXT-2014 or SHELXT-2018* programs,[36] which revealed the positions of all non-hydrogen atoms. The non-hydrogen atoms were refined anisotropically. Unless otherwise stated below, all of the H-atoms were placed in geometrically calculated positions and refined by using a riding model where each H-atom was assigned a fixed isotropic displacement parameter with a value equal to $1.2U_{eq}$ of its parent atom ($1.5U_{eq}$ for the methyl groups and any water H-atoms). The refinement of each structure was carried out on F^2 by using full-matrix least-squares procedures, which minimised the function $\Sigma w(F_o^2 - F_c^2)^2$. Neutral atom scattering factors for non-hydrogen atoms were taken from Maslen, Fox and O'Keefe,[37] and the scattering factors for H-atoms were taken from Stewart, Davidson and Simpson.[38] Anomalous dispersion effects were included in F_c[39]; the values for f' and f'' were those of Creagh and McAuley.[40] The values of the mass attenuation coefficients are those of Creagh and Hubbel.[41] The *SHELXL-2014 or SHELXL-2018* programs[42] were used for all calculations. Displacement ellipsoid plots of the molecules are drawn with 50% probability ellipsoids[43,44] hydrogen atoms are omitted for clarity; the colour codes are: gray, carbon; red, oxygen; blue, nitrogen; sea green, palladium; yellow green, fluorine; pink, boron.

Crystal structure of C3$^+$BF$_4^-$

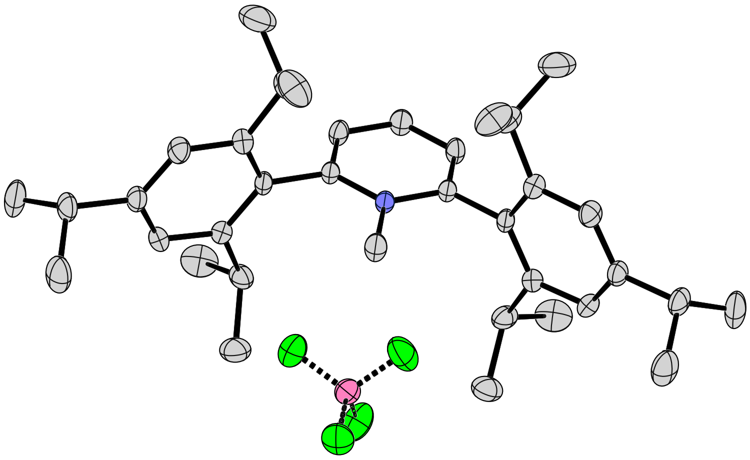

In the structure of **C3$^+$BF$_4^-$** ($C_{36}H_{52}N^+$ BF_4^-), the unique cation and anion sit across a mirror plane. The methyl H-atoms of the methylamine group are disordered across the mirror and necessarily have equal site occupation. The anion is disordered over four orientations, defined as two mirror-related pairs. Two sets of positions were defined for all atoms of the anion and the site occupation factor of the major conformation refined to 0.342(2). The minor conformation occupancy is 0.158(2) and then the mirror-related orientations duplicate these. Similarity restraints were applied to all B–F bond lengths and F···F distances within the anion, while neighbouring atoms within and between each orientation of the disordered anion were restrained to have similar atomic displacement parameters. These orientations of the anion approximate a "spinning top" type disorder where the four positions of one F-atom and the B-atom are almost on identical sites across all orientations, while the remaining three F-atoms are approximating a toroidal ring of electron density.

Crystallographic data

Crystallised from	EtOAc
Empirical formula	$C_{36}H_{52}BF_4N$
Formula weight [g mol^{-1}]	585.59
Crystal colour, habit	colourless, prism
Crystal dimensions [mm]	0.04 × 0.06 × 0.10
Temperature [K]	160(1)
Crystal system	orthorhombic
Space group	*Pnma* (#62)
Z	4
Reflections for cell determination	14705
2θ range for cell determination [°]	7–149
Unit cell parameters *a* [Å]	12.1344(2)

b [Å]	25.1177(4)
c [Å]	11.0932(2)
α [°]	90
β [°]	90
γ [°]	90
V [Å^3]	3381.08(10)
$F(000)$	1264
D_x [g cm^{-3}]	1.150
μ(Cu $K\alpha$) [mm^{-1}]	0.648
Scan type	ω
$2\theta_{(max)}$ [°]	148.5
Transmission factors (min; max)	0.892; 1.000
Total reflections measured	32180
Symmetry independent reflections	3515
R_{int}	0.025
Reflections with $I > 2\sigma(I)$	3160
Reflections used in refinement	3515
Parameters refined; restraints	270; 255
Final $R(F)$ [$I > 2\sigma(I)$ reflections]	0.0551
$wR(F^2)$ (all data)	0.1582
Weights:	$w = [\sigma^2(F_o^2) + (0.0703P)^2 + 2.3425P]^{-1}$ where $P = (F_o^2 + 2F_c^2)/3$
Goodness of fit	1.127
Final Δ_{max}/σ	0.000
$\Delta\rho$ (max; min) [e Å^{-3}]	0.24; -0.25
$\sigma(d_{(C-C)})$ [Å]	0.0019 – 0.003

Crystal structure of $C4^{+}BF_4^{-}$

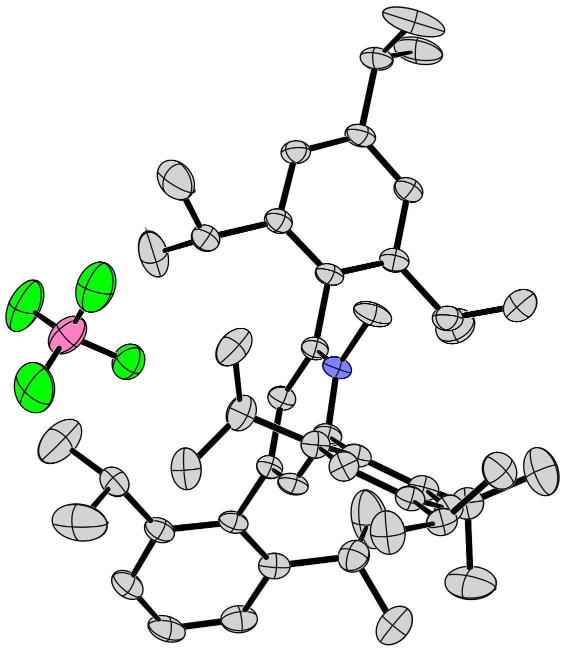

The anion in the crystal structure of **$C4^{+}BF_4^{-}$** ($C_{48}H_{68}N^{+}$ BF_4^{-}) is disordered over two orientations which are approximately mirror-related. Two sets of positions were defined for all atoms of the anion and the site occupation factor of the major orientation refined to 0.660(3). Similarity restraints were applied to all B–F bond lengths, while neighboring atoms within and between each orientation of the disordered anion were restrained to have similar atomic displacement parameters. A correction for secondary extinction was applied.

Crystallographic data:

Crystallised from	CH_2Cl_2 / iPrOH
Empirical formula	$C_{48}H_{68}BF_4N$
Formula weight [g mol^{-1}]	745.84
Crystal colour, habit	colourless, needle
Crystal dimensions [mm]	0.03 × 0.06 × 0.18
Temperature [K]	160(1)
Crystal system	triclinic
Space group	$P\bar{1}$ (#2)
Z	2
Reflections for cell determination	21858
2θ range for cell determination [°]	6–149

Unit cell parameters	a [Å]	11.4195(3)
	b [Å]	13.2952(3)
	c [Å]	15.0834(3)
	α [°]	95.3682(19)
	β [°]	101.1467(19)
	γ [°]	93.8514(19)
	V [Å^3]	2228.44(9)
F(000)		808
D_x [g cm^{-3}]		1.112
μ(Cu $K\alpha$) [mm^{-1}]		0.589
Scan type		ω
$2\theta_{(max)}$ [°]		148.7
Transmission factors (min; max)		0.904; 1.000
Total reflections measured		41871
Symmetry independent reflections		8819
R_{int}		0.019
Reflections with $I > 2\sigma(I)$		7704
Reflections used in refinement		8819
Parameters refined; restraints		551; 52
Final	$R(F)$ [$I > 2\sigma(I)$ reflections]	0.0467
	$wR(F^2)$ (all data)	0.1287
Weights:		$w = [\sigma^2(F_o^2) + (0.0621P)^2 + 0.8820P]^{-1}$ where $P = (F_o^2 + 2F_c^2)/3$
Goodness of fit		1.034
Secondary extinction coefficient		0.0006(2)
Final Δ_{max}/σ		0.001
$\Delta\rho$ (max; min) [e Å^{-3}]		0.39; -0.33
$\sigma(d_{(C-C)})$ [Å]		0.0015 – 0.002

Crystal structure of $C5^{+}BF_4^{-}$

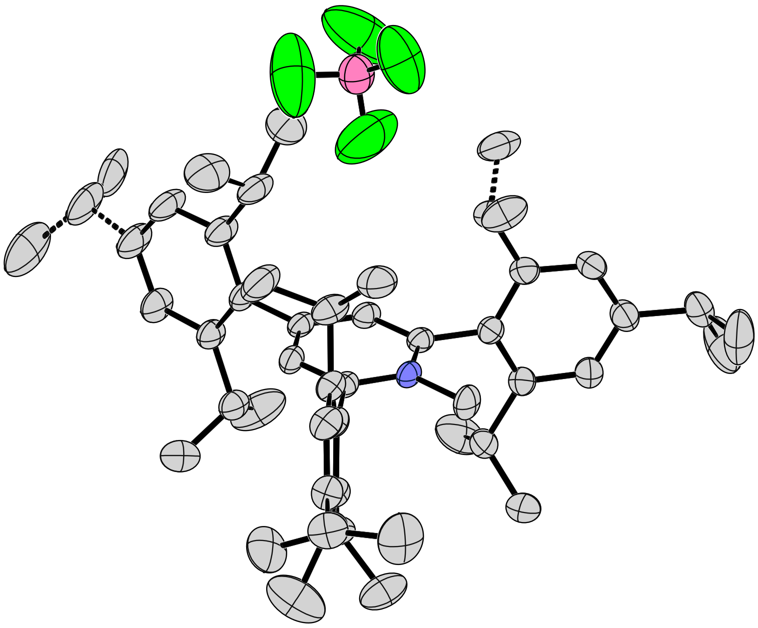

The asymmetric unit of **$C5^{+}BF_4^{-}$** ($C_{51}H_{74}N^{+}$ $BF_4^{-}\cdot C_7H_{16}$) contains one half of a cation, one half of an anion and one half of a disordered solvent molecule, estimated to be *n*-heptane. Each of these entities lie across crystallographic mirror planes. The disordered solvent molecule could not be modelled adequately, so its contribution to the diffraction data was removed using the *SQUEEZE* routine of the program *PLATON*.[45] When the solvent molecule is excluded from the model, there are four cavities of 233 Å^3 per unit cell. The total number of electrons contributing to each void was calculated by the *SQUEEZE* routine to be approximately 54 e. An *n*-heptane molecule has 58 e, which suggests that each cavity has one molecule of *n*-heptane, rather than the other solvent used, which was benzene (42 e). This approximation was used in the subsequent calculation of the empirical formula, formula weight, density, linear absorption coefficient and F(000). Based on the assumption, the overall ratio of cations to anions and n-heptane molecules is 1:1:1. Two of the symmetry-unique ^{i}Pr groups are disordered over two conformations, one of which is disordered across the mirror plane. The site occupation factors of the unique atoms of the ^{i}Pr group disordered across the mirror plane were fixed at 0.5. Two sets of positions were defined for the atoms of the other disordered ^{i}Pr group and the site occupation factor of the major conformation refined to 0.650(11). Similarity restraints were applied to the chemically equivalent bond lengths involving all disordered C-atoms, while neighbouring atoms within and between each of the disordered conformations were restrained to have similar atomic displacement parameters. A numerical absorption correction was applied.

Crystallographic data:

Crystallised from	benzene / *n*-heptane
Empirical formula	$C_{58}H_{90}BF_4N$
Formula weight [g mol^{-1}]	888.11

Crystal colour, habit		colourless, prism
Crystal dimensions [mm]		$0.14 \times 0.17 \times 0.43$
Temperature [K]		160(1)
Crystal system		orthorhombic
Space group		*Pnma* (#62)
Z		4
Reflections for cell determination		16310
2θ range for cell determination [°]		7–146
Unit cell parameters	a [Å]	17.99832(15)
	b [Å]	20.8154(2)
	c [Å]	14.54327(12)
	α [°]	90
	β [°]	90
	γ [°]	90
	V [Å^3]	5448.52(8)
$F(000)$		1944
D_x [g cm^{-3}]		1.083
μ(Cu $K\alpha$) [mm^{-1}]		0.549
Scan type		ω
$2\theta_{(max)}$ [°]		146.3
Transmission factors (min; max)		0.549; 1.000
Total reflections measured		27340
Symmetry independent reflections		5528
R_{int}		0.019
Reflections with $I > 2\sigma(I)$		4852
Reflections used in refinement		5528
Parameters refined; restraints		324; 63
Final	$R(F)$ [$I > 2\sigma(I)$ reflections]	0.0722

$wR(F^2)$ (all data)	0.2169
Weights:	$w = [\sigma^2(F_o^2) + (0.1102P)^2 + 3.3647P]^{-1}$ where $P = (F_o^2 + 2F_c^2)/3$
Goodness of fit	1.064
Final Δ_{max}/σ	0.000
$\Delta\rho$ (max; min) [e Å^{-3}]	0.75; -0.56
$\sigma(d_{(C–C)})$ [Å]	0.002 – 0.004

Crystal structure of C7$^+$BF$_4^-$

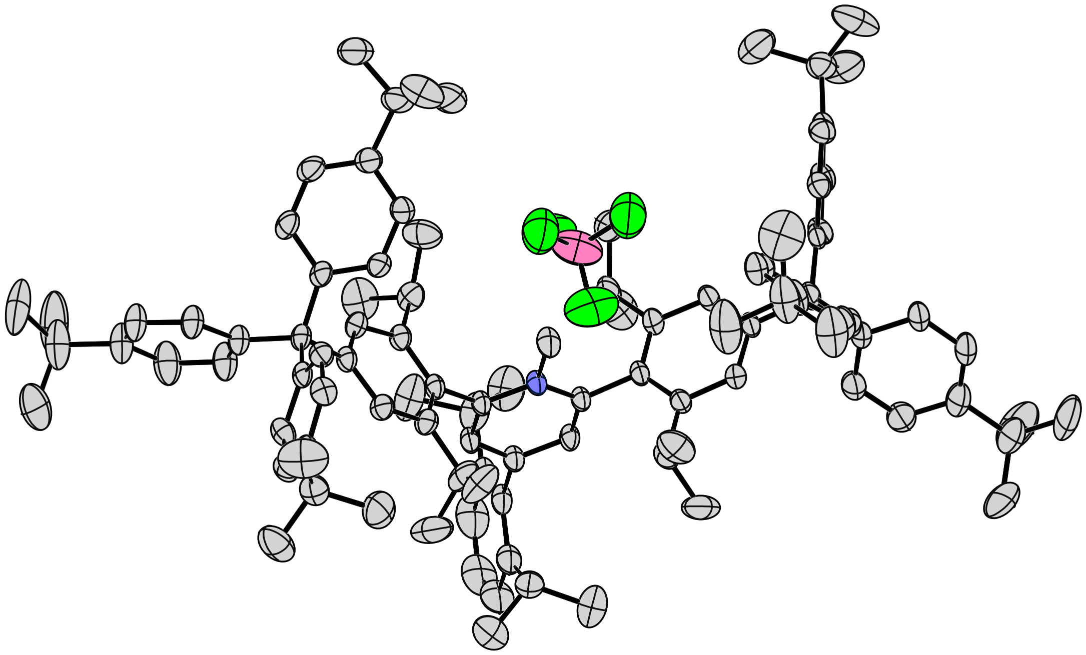

The asymmetric unit of **C7$^+$BF$_4^-$** ($C_{104}H_{132}N^+$ $BF_4^-\cdot 3CH_2Cl_2$) contains half of a cation and half of an anion, each of which sit across a C_2-axis, plus one and a half molecules of CH_2Cl_2, one of which is disordered across a centre of inversion. The F-atoms of the anion and one of the t-butyl groups of the cation are also disordered. Two sets of positions were defined for the symmetry-unique F-atoms and the atoms of the disordered t-butyl group and the site occupation factors of the major conformations of these groups refined to 0.683(4) and 0.673(8), respectively. Similarity restraints were applied to the chemically equivalent bond lengths involving all disordered F- and C-atoms, as well as the $C_{methyl}\cdots C_{methyl}$ distances within the disordered t-butyl group. Neighbouring atoms within and between each conformation of the disordered groups were restrained to have similar atomic displacement parameters and pseudo-isotropic restraints were applied additionally to some t-butyl C_{methyl} atoms. Attempts to model the disordered CH_2Cl_2 molecule led to unsatisfactory results, so the *SQUEEZE* routine of the program *PLATON*[45] was employed to account for this solvent molecule. Omission of the disordered CH_2Cl_2 molecule leaves four cavities of 96 Å^3 and four of 114 Å^3 per unit cell, centred about centres of inversion. The total number of electrons contributing to the larger voids in the unit cell was calculated by the *SQUEEZE* routine to be approximately 31 e, although this can be an underestimate. A negligible electron count was assigned to the smaller voids. Allowing for one molecule of CH_2Cl_2 per void yields 42 e and this approximation was used in the subsequent calculation of the empirical formula, formula weight, density, linear absorption coefficient and F(000). Based on the assumption, the overall ratio of cation:anion:CH_2Cl_2 in the structure is 1:1:3.

Crystallographic data:

Crystallised from	CH_2Cl_2/hexane
Empirical formula	$C_{107}H_{138}BCl_6F_4N$
Formula weight [g mol^{-1}]	1737.69

Crystal colour, habit	colourless, block
Crystal dimensions [mm]	$0.28 \times 0.31 \times 0.35$
Temperature [K]	160(1)
Crystal system	monoclinic
Space group	*C*2/*c* (#15)
Z	4
Reflections for cell determination	30803
2θ range for cell determination [°]	7–146
Unit cell parameters *a* [Å]	20.56989(16)
b [Å]	15.92753(12)
c [Å]	31.9108(2)
α [°]	90
β [°]	93.9468(6)
γ [°]	90
V [Å^3]	10430.06(13)
F(000)	3720
D_x [g cm^{-3}]	1.107
μ(Cu $K\alpha$) [mm^{-1}]	1.893
Scan type	ω
$2\theta_{(max)}$ [°]	146.0
Transmission factors (min; max)	0.886; 1.000
Total reflections measured	49228
Symmetry independent reflections	10211
R_{int}	0.015
Reflections with $I > 2\sigma(I)$	9268
Reflections used in refinement	10211
Parameters refined; restraints	592; 120
Final $R(F)$ [$I > 2\sigma(I)$ reflections]	0.0712

$wR(F^2)$ (all data)	0.2129
Weights:	$w = [\sigma^2(F_O^2) + (0.1193P)^2 + 13.6373P]^{-1}$ where $P = (F_O^2 + 2F_C^2)/3$
Goodness of fit	1.037
Final Δ_{max}/σ	0.001
$\Delta\rho$ (max; min) [e Å^{-3}]	0.72; -0.62
$\sigma(d_{(C-C)})$ [Å]	0.002 – 0.006

Crystal structure of $C8^{2+}(BF_4^-)_2$

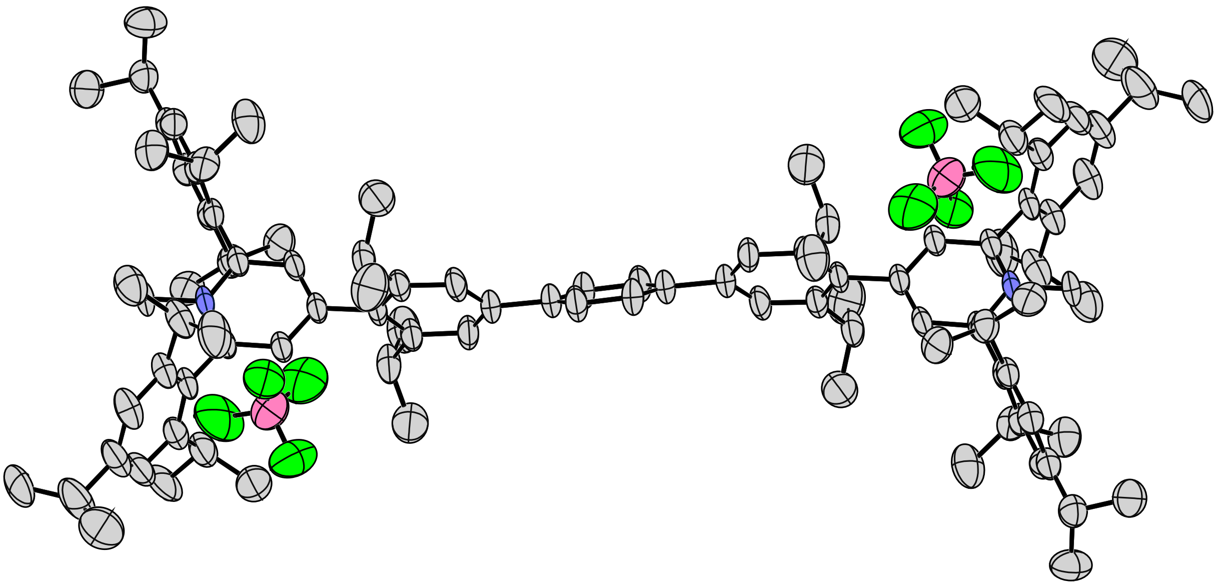

The asymmetric unit in the structure of $\mathbf{C8^{2+}(BF_4^-)_2}$ ($C_{102}H_{138}N_2^{2+}$ $2BF_4^-{\cdot}2C_7H_{14}{\cdot}2C_6D_6$) contains one half of a cation, which sits about a crystallographic centre of inversion, one tetrafluoroborate anion and, at a rough estimate, one disordered molecule of deuterobenzene and one disordered molecule of *n*-heptane. An indication of which and how many solvent molecules were present could not be derived from the residual electron density peaks. The contribution of the solvent molecules to the diffraction data was removed using the *SQUEEZE* routine of the program *PLATON*.[45] When the solvent molecules are excluded from the model, there are two cavities of 1584 Å^3 per unit cell. The total number of electrons contributing to each void was calculated by the *SQUEEZE* routine to be approximately 386 e. A deuterobenzene molecule has 42 e and a *n*-heptane molecule has 58 e. If it is assumed that each cavity contains four of each of these molecules, there are 400 e per cavity (one of each solvent molecule or 100 e per asymmetric unit), which is fairly close to the electron count calculated by SQUEEZE. This approximation was used in the subsequent calculation of the empirical formula, formula weight, density, linear absorption coefficient and F(000). Based on the assumption, the overall ratio of cation:anion:deuterobenzene:*n*-heptane is 1:2:2:2. Four ^{i}Pr groups in the symmetry-unique half of the cation, the central phenyl ring of the cation and the anion were modelled as disordered over two conformations. Two sets of positions were defined for the atoms of each disordered group and the site occupation factors of the major conformations of these groups refined to between 0.509(15) and 0.578(19) for the ^{i}Pr groups, to 0.756(4) for the central phenyl ring of the cation and to 0.705(3) for the anion. Similarity restraints were applied to the chemically equivalent bond lengths involving all disordered atoms, as well as to the F···F distances within the anion, while neighbouring atoms within and between each of the disordered conformations were restrained to have similar atomic displacement parameters. A numerical absorption correction was applied.

Crystallographic data:

Crystallised from	deuterobenzene / *n*-heptane
Empirical formula	$C_{128}H_{182}B_2F_8N_2$

Formula weight [g mol^{-1}]		1922.37
Crystal colour, habit		colourless, needle
Crystal dimensions [mm]		0.06 × 0.09 × 0.33
Temperature [K]		160(1)
Crystal system		monoclinic
Space group		*C*2/*c* (#15)
Z		4
Reflections for cell determination		15613
2θ range for cell determination [°]		6–144
Unit cell parameters	*a* [Å]	40.0822(9)
	b [Å]	11.1343(2)
	c [Å]	26.9243(7)
	α [°]	90
	β [°]	97.360(2)
	γ [°]	90
	V [Å^3]	11917.0(5)
F(000)		4184
D_x [g cm^{-3}]		1.071
μ(Cu *K*α) [mm^{-1}]		0.538
Scan type		ω
2$\theta_{(max)}$ [°]		144.8
Transmission factors (min; max)		0.765; 1.000
Total reflections measured		56841
Symmetry independent reflections		11514
R_{int}		0.029
Reflections with $I > 2\sigma(I)$		8523
Reflections used in refinement		11514
Parameters refined; restraints		680; 376

Final	$R(F)$ [$I > 2\sigma(I)$ reflections]	0.0921
	$wR(F^2)$ (all data)	0.3268
Weights:		$w = [\sigma^2(F_o^2) + (0.2000P)^2]^{-1}$ where $P = (F_o^2 + 2F_c^2)/3$
Goodness of fit		1.345
Final Δ_{max}/σ		0.001
$\Delta\rho$ (max; min) [e Å^{-3}]		1.05; -1.00
$\sigma(d_{(C-C)})$ [Å]		0.002 – 0.010

Crystal structure of C2⁺A4⁻

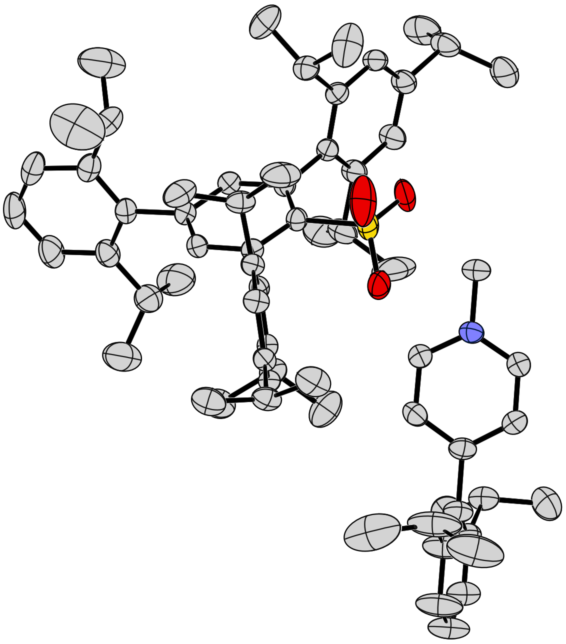

The asymmetric unit in the structure of **C2⁺A4⁻** ($C_{18}H_{24}N^{+}$ $C_{48}H_{65}O_3S^{-}\cdot1.5(C_6H_5Cl)$) contains one cation, one anion and one and a half disordered molecules of chlorobenzene, with the half-molecule arising from a chlorobenzene molecule that sits across a crystallographic centre of inversion. The disordered solvent molecules could not be modelled adequately, so their contribution to the diffraction data was removed using the *SQUEEZE* routine of the program *PLATON*.[45] When the solvent molecules are excluded from the model, there are four cavities of 217 Å^3 and two cavities of 172 Å^3 per unit cell, with the latter sitting across centres of inversion. The total number of electrons contributing to each void was calculated by the *SQUEEZE* routine to be approximately 58 e. A chlorobenzene molecule has 58 e, which suggests that each cavity contains one molecule of chlorobenzene and this approximation was used in the subsequent calculation of the empirical formula, formula weight, density, linear absorption coefficient and F(000). Based on the assumption, the overall ratio of cations : anions : solvent molecules is 1:1:1.5. The $–SO_3^-$ group is disordered over two orientations. Two sets of positions were defined for the atoms of this group and the site occupation factor of the major conformation of the group refined to 0.737(3). Similarity restraints were applied to the chemically equivalent bond lengths involving all disordered O-atoms, while neighbouring atoms within and between each of the disordered orientations were restrained to have similar atomic displacement parameters. Pseudo-isotropic restraints were applied to the atomic displacement parameters of the atoms of one isopropyl group. A numerical absorption correction was applied.

Crystallographic data

Crystallised from	chlorobenzene / hexane
Empirical formula	$C_{75}H_{96.5}Cl_{1.5}NO_3S$
Formula weight [g mol^{-1}]	1145.26
Crystal colour, habit	colourless, plate

Crystal dimensions [mm]		0.04 × 0.14 × 0.16
Temperature [K]		160(1)
Crystal system		monoclinic
Space group		$P2_1/c$ (#14)
Z		4
Reflections for cell determination		22564
2θ range for cell determination [°]		5−144
Unit cell parameters	a [Å]	10.59983(10)
	b [Å]	21.2959(2)
	c [Å]	30.1508(2)
	α [°]	90
	β [°]	91.5265(8)
	γ [°]	90
	V [Å^3]	6803.61(10)
$F(000)$		2476
D_x [g cm^{-3}]		1.118
μ(Cu $K\alpha$) [mm^{-1}]		1.306
Scan type		ω
$2\theta_{(max)}$ [°]		143.5
Transmission factors (min; max)		0.780; 1.000
Total reflections measured		64901
Symmetry independent reflections		13200
R_{int}		0.028
Reflections with $I > 2\sigma(I)$		10750
Reflections used in refinement		13200
Parameters refined; restraints		689; 69
Final	$R(F)$ [$I > 2\sigma(I)$ reflections]	0.0523
	$wR(F^2)$ (all data)	0.1517
Weights:		$w = [\sigma^2(F_o^2) + (0.0756P)^2 + 2.5261P]^{-1}$ where $P = (F_o^2 + 2F_c^2)/3$
Goodness of fit		1.050
Final Δ_{max}/σ		0.001

$\Delta\rho$ (max; min) [e Å^{-3}]	0.45; -0.30
$\sigma(d_{(C-C)})$ [Å]	0.0019 – 0.006

Crystal structure of C4$^+$A2$^-$

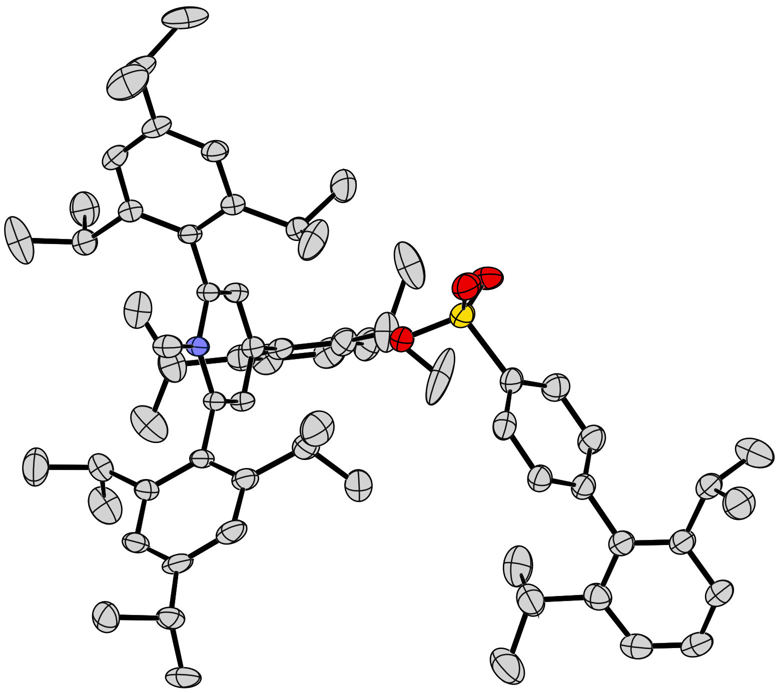

The asymmetric unit in the structure of **C4$^+$A2$^-$** ($C_{48}H_{68}N^+$ $C_{18}H_{21}O_3S^-$ $\cdot 0.5(C_8H_{10})$), contains one cation, one anion and one half of a disordered molecule of *p*-xylene with the half-molecule arising from a *p*-xylene molecule that sits across a crystallographic centre of inversion. In the anion, the $-SO_3^-$ group is disordered, while in the cation, three ^{i}Pr groups are disordered. Two sets of positions were defined for the O-atoms of the $-SO_3^-$ group (site occupation factor of the major conformation refined to 0.58(3)), for the two methyl groups of two ^{i}Pr groups (site occupation factors for the major conformations of the groups bonded to C30 and C45 refined to 0.591(19) and 0.644(15), respectively), for the entire ^{i}Pr group at C55 (site occupation factor for the major conformation refined to 0.672(5)), and for the symmetry-unique atoms of the solvent molecule (site occupation factor for the major conformation refined to 0.700(5)). Similarity restraints were applied to the chemically equivalent bond lengths involving all disordered atoms, as well as to the O···O distances in the $-SO_3^-$ group, while neighbouring atoms within and between each of the disordered conformations were restrained to have similar atomic displacement parameters. Pseudo-isotropic restraints were also applied to the atomic displacement parameters of the atoms of one disordered ^{i}Pr group. A numerical absorption correction was applied.

Crystallographic data

Crystallised from	Et_2O / hexanes / *p*-xylene
Empirical formula	$C_{70}H_{94}NO_3S$
Formula weight [g mol^{-1}]	1029.52
Crystal colour, habit	colourless, prism
Crystal dimensions [mm]	0.09 × 0.25 × 0.36
Temperature [K]	160(1)

Crystal system		triclinic
Space group		$P\bar{1}$ (#2)
Z		2
Reflections for cell determination		38626
2θ range for cell determination [°]		4–153
Unit cell parameters	a [Å]	12.7871(4)
	b [Å]	13.2999(5)
	c [Å]	21.1254(5)
	α [°]	73.610(2)
	β [°]	84.331(2)
	γ [°]	64.782(3)
	V [Å^3]	3117.29(19)
$F(000)$		1122
D_x [g cm^{-3}]		1.097
μ(Cu $K\alpha$) [mm^{-1}]		0.796
Scan type		ω
$2\theta_{(max)}$ [°]		153.7
Transmission factors (min; max)		0.385; 1.000
Total reflections measured		62886
Symmetry independent reflections		12803
R_{int}		0.020
Reflections with $I > 2\sigma(I)$		11622
Reflections used in refinement		12803
Parameters refined; restraints		837; 326
Final	$R(F)$ [$I > 2\sigma(I)$ reflections]	0.0474
	$wR(F^2)$ (all data)	0.1337
Weights:		$w = [\sigma^2(F_o^2) + (0.0692P)^2 + 1.0638P]^{-1}$ where $P = (F_o^2 + 2F_c^2)/3$
Goodness of fit		1.044
Final Δ_{max}/σ		0.002
$\Delta\rho$ (max; min) [e Å^{-3}]		0.56; -0.58
$\sigma(d_{(C-C)})$ [Å]		0.0014 – 0.013

Crystal structure of C3⁺A3⁻

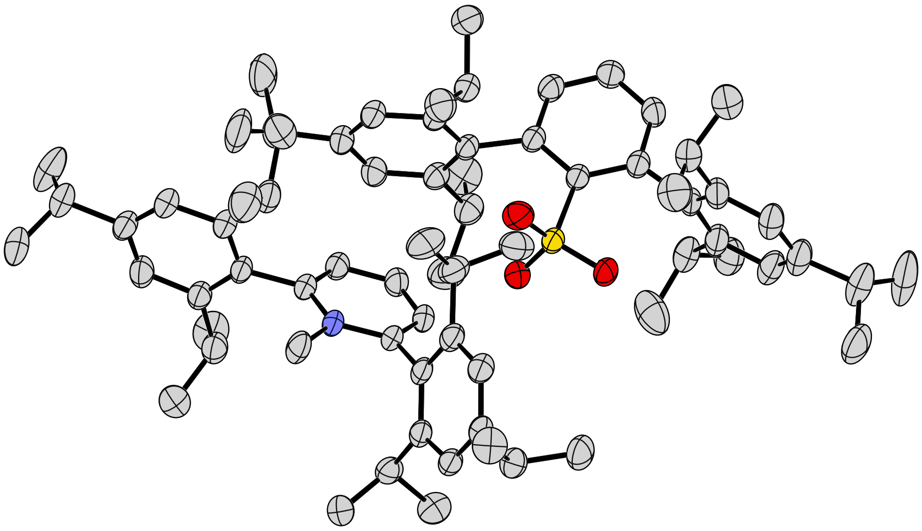

The asymmetric unit in the structure of **C3⁺A3⁻** ($C_{36}H_{52}N^+$ $C_{36}H_{49}O_3S^-$) contains one cation and one anion. Some of the isopropyl groups are possibly slightly disordered. Disorder has been modelled for one of these in the anion. In other groups, modelling the disorder does not bring any improvement overall. Two sets of positions were defined for the methyl atoms of the isopropyl group for which disorder was modelled and the site occupation factor of the major conformation of this group refined to 0.778(13). Similarity restraints were applied to the chemically equivalent C–C bond lengths involving all disordered atoms and these C-atoms were restrained to have similar and pseudo-isotropic atomic displacement parameters. In addition, rigid bond restraints were applied to the atomic displacement parameters of all non-hydrogen atoms.

Crystallographic data:

Crystallised from	hexanes
Empirical formula	$C_{72}H_{101}NO_3S$
Formula weight [g mol^{-1}]	1060.59
Crystal colour, habit	colourless, prism
Crystal dimensions [mm]	0.06 × 0.10 × 0.20
Temperature [K]	160(1)
Crystal system	triclinic
Space group	$P\bar{1}$ (#2)
Z	2
Reflections for cell determination	23840
2θ range for cell determination [°]	6–149

Unit cell parameters	a [Å]	13.0647(2)
	b [Å]	13.6914(3)
	c [Å]	20.0229(4)
	α [°]	81.6247(15)
	β [°]	78.9167(15)
	γ [°]	73.8402(16)
	V [Å^3]	3359.73(12)
F(000)		1160
D_x [g cm^{-3}]		1.048
μ(Cu $K\alpha$) [mm^{-1}]		0.750
Scan type		ω
$2\theta_{(max)}$ [°]		148.9
Transmission factors (min; max)		0.168; 1.000
Total reflections measured		63461
Symmetry independent reflections		13524
R_{int}		0.048
Reflections with $I > 2\sigma(I)$		10537
Reflections used in refinement		13524
Parameters refined; restraints		740; 666
Final	$R(F)$ [$I > 2\sigma(I)$ reflections]	0.0527
	$wR(F^2)$ (all data)	0.1529
Weights:		$w = [\sigma^2(F_o^2) + (0.0769P)^2 + 1.0432P]^{-1}$ where $P = (F_o^2 + 2F_c^2)/3$
Goodness of fit		1.026
Final Δ_{max}/σ		0.054
$\Delta\rho$ (max; min) [e Å^{-3}]		0.56; -0.29
$\sigma(d_{(C-C)})$ [Å]		0.002 – 0.005

Crystal structure of 1

view 1:

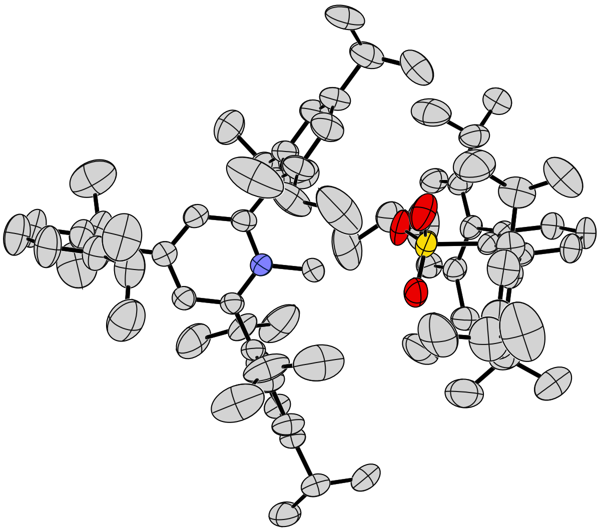

view 2:

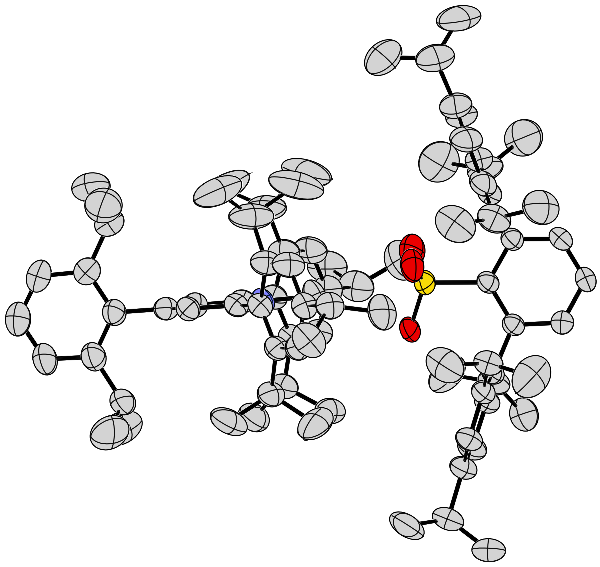

view 3:

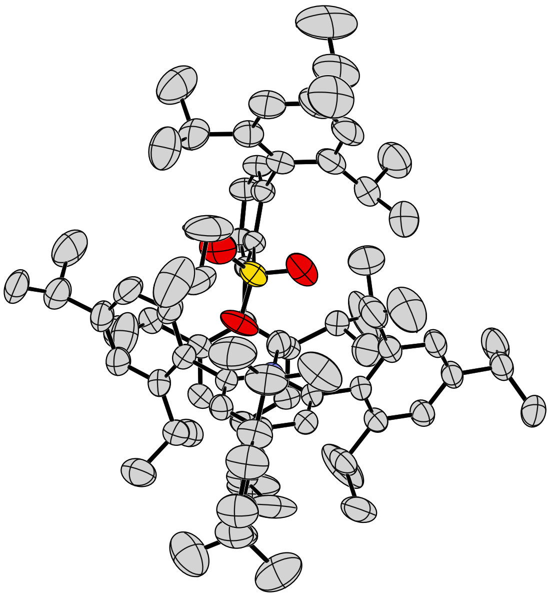

The crystals of **1** ($C_{48}H_{68}N^+$ $C_{36}H_{49}O_3S^-$ $\cdot C_6H_{12}$) were very weakly diffracting and while the molecular connectivity is unambiguous, the overall quality of the structure is poorer than normal. There is probably disorder in some of the isopropyl groups, because the atomic displacement parameters for the atoms of these groups are quite elongated. Attempts to model the disorder did not yield any improvement, and an ordered model was employed for the isopropyl groups in the final refinement. On the other hand, the O-atoms of the sulfonate group could be modelled as disordered over two positions and the site occupation factor of the major orientation of this group refined to 0.792(4). Similarity restraints were applied to the S–O bond lengths and O···O distances, while the O-atoms were restrained to have similar atomic displacement parameters. The asymmetric unit appears to contain one disordered molecule of cyclohexane, although this could not be modelled successfully, so the *SQUEEZE* routine of the program *PLATON*[45] was employed. Omission of the solvent molecules leaves two centrosymmetric cavities of 555 $Å^3$ per unit cell. The number of electrons contributing to each void in the structure was calculated by the *SQUEEZE* routine to be approximately 104 e. Allowing for two cyclohexane molecules per cavity (one per asymmetric unit) yields 96 e and this approximation was been used in the subsequent calculation of the empirical formula, formula weight, density, linear absorption coefficient and *F*(000). Based on the assumption, the ratio of cations and anions to cyclohexane in the structure is 1:1.

Crystallographic data

Crystallised from		hexane
Empirical formula		$C_{90}H_{129}NO_3S$
Formula weight [g mol^{-1}]		1304.99
Crystal colour, habit		colourless, tablet
Crystal dimensions [mm]		0.03 × 0.07 × 0.14
Temperature [K]		160(1)
Crystal system		monoclinic
Space group		$P2_1/n$ (#14)
Z		4
Reflections for cell determination		10964
2θ range for cell determination [°]		7–120
Unit cell parameters	*a* [Å]	12.6383(3)
	b [Å]	41.8526(11)
	c [Å]	16.5501(4)
	α [°]	90
	β [°]	105.600(3)

γ [°]	90
V [Å^3]	8431.6(4)
F(000)	2864
D_x [g cm^{-3}]	1.028
μ(Cu $K\alpha$) [mm^{-1}]	0.675
Scan type	ω
$2\theta_{(max)}$ [°]	148.4
Transmission factors (min; max)	0.609; 1.000
Total reflections measured	79461
Symmetry independent reflections	16862
R_{int}	0.062
Reflections with $I > 2\sigma(I)$	10209
Reflections used in refinement	16860
Parameters refined; restraints	859; 66
Final $R(F)$ [$I > 2\sigma(I)$ reflections]	0.0801
$wR(F^2)$ (all data)	0.2468
Weights:	$w = [\sigma^2(F_o^2) + (0.1236P)^2 + 2.3129P]^{-1}$ where $P = (F_o^2 + 2F_c^2)/3$
Goodness of fit	1.033
Final Δ_{max}/σ	0.001
$\Delta\rho$ (max; min) [e Å^{-3}]	1.04; -0.43
$\sigma(d_{(C-C)})$ [Å]	0.004 – 0.008

Crystal structure of 2

view 1:

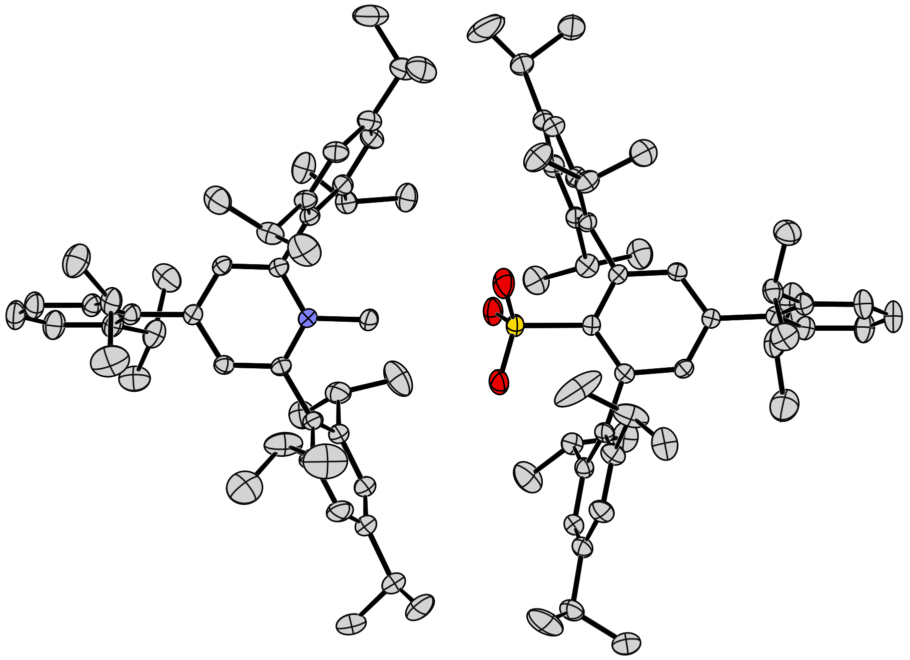

view 2:

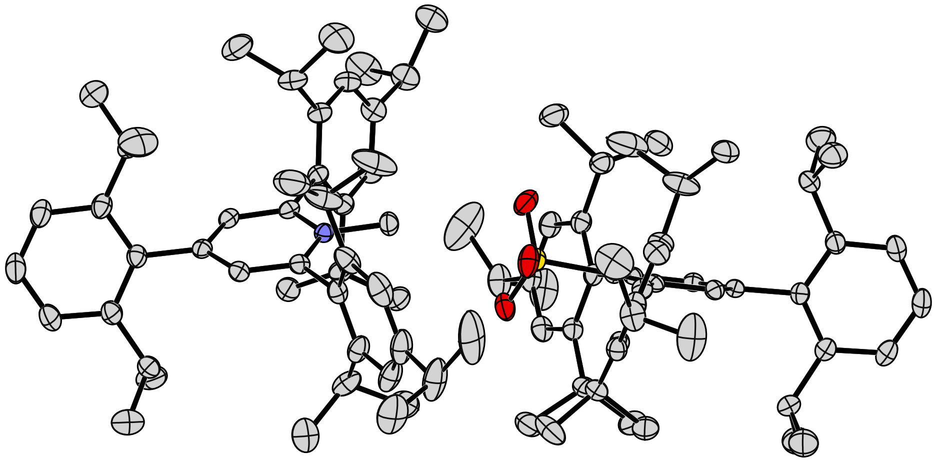

view 3:

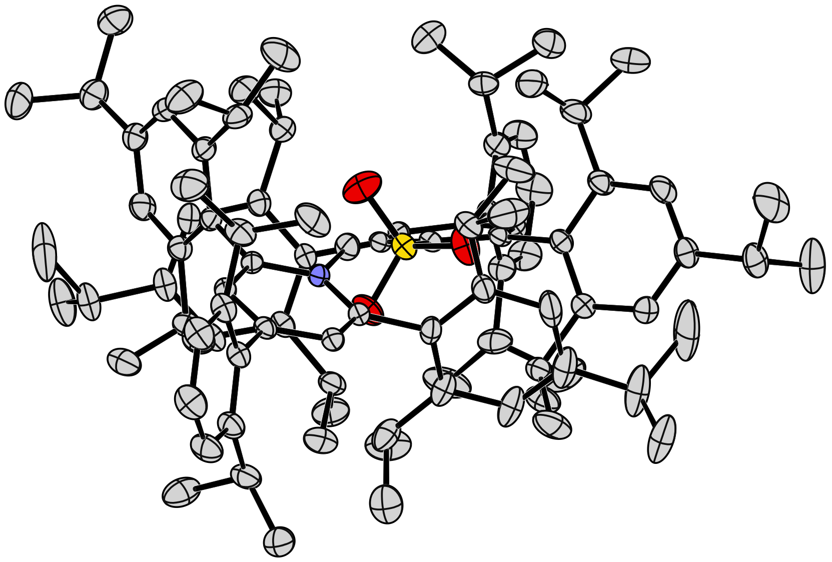

The asymmetric unit in the structure of **2** ($C_{48}H_{68}N^+$ $C_{48}H_{65}O_3S^-$ $\cdot 0.5C_6H_{14}\cdot 2H_2O$) contains one cation, one anion and some highly diffuse and disordered solvent molecules whose exact nature cannot be elucidated, but is it surmised that hexane and water might be present. The contribution of the solvent molecules to the diffraction data was removed by using the *SQUEEZE* routine of the program *PLATON.*[45] Omission of the solvent molecules leaves one cavity of 511 Å^3, situated about a centre of inversion, and two cavities of 90 Å^3 per unit cell. The number of electrons contributing the first void was calculated by the *SQUEEZE* routine to be 77 e, while 15 e are contained in each of the smaller voids in the unit cell (total 107 e per unit cell). Allowing for one molecule of hexane (50 e) and two water molecules (10 e each) in the large void and one water molecule in each of the smaller voids yields 90 e and this approximation was used in the subsequent calculation of the empirical formula, formula weight, density, linear absorption coefficient and F(000). Based on the assumption, the ratio of cations:anions:hexane:H_2O is 1:1:0.5:2. Some of the ^{i}Pr groups show evidence of disorder. Disorder was modelled for one of these groups, but attempts to model the disorder for other groups did not yield any improvement in the model, so these groups were refined as ordered, albeit with enlarged atomic displacement parameters for some C-atoms. Two sets of positions were defined for the methyl atoms of one ^{i}Pr group and the site occupation factor of the major conformation refined to 0.512(8). Similarity restraints were applied to the C–C bond lengths involving all disordered C-atoms, while neighbouring disordered atoms were restrained to have similar atomic displacement parameters. A correction for secondary extinction was applied.

Crystallographic data

Crystallised from		hexane
Empirical formula		$C_{99}H_{144}NO_6S$
Formula weight [g mol^{-1}]		1460.20
Crystal colour, habit		colourless, prism
Crystal dimensions [mm]		0.16 × 0.20 × 0.22
Temperature [K]		160(1)
Crystal system		triclinic
Space group		$P\bar{1}$ (#2)
Z		2
Reflections for cell determination		40984
2θ range for cell determination [°]		6–149
Unit cell parameters	*a* [Å]	14.5585(3)
	b [Å]	17.4027(3)
	c [Å]	21.0730(4)
	α [°]	102.6796(16)

β [°]	103.5351(15)
γ [°]	106.4067(16)
V [Å^3]	4740.12(17)
$F(000)$	1602
D_x [g cm^{-3}]	1.023
μ(Cu $K\alpha$) [mm^{-1}]	0.661
Scan type	ω
$2\theta_{(max)}$ [°]	148.9
Transmission factors (min; max)	0.835; 1.000
Total reflections measured	87128
Symmetry independent reflections	18497
R_{int}	0.020
Reflections with $I > 2\sigma(I)$	15960
Reflections used in refinement	18497
Parameters refined; restraints	965; 18
Final $R(F)$ [$I > 2\sigma(I)$ reflections]	0.0534
$wR(F^2)$ (all data)	0.1581
Weights:	$w = [\sigma^2(F_o^2) + (0.0816P)^2 + 1.8296P]^{-1}$ where $P = (F_o^2 + 2F_c^2)/3$
Goodness of fit	1.039
Secondary extinction coefficient	0.00049(9)
Final Δ_{max}/σ	0.001
$\Delta\rho$ (max; min) [e Å^{-3}]	0.49; -0.34
$\sigma(d_{(C-C)})$ [Å]	0.002 – 0.005

Crystal structure of 3

view 1:

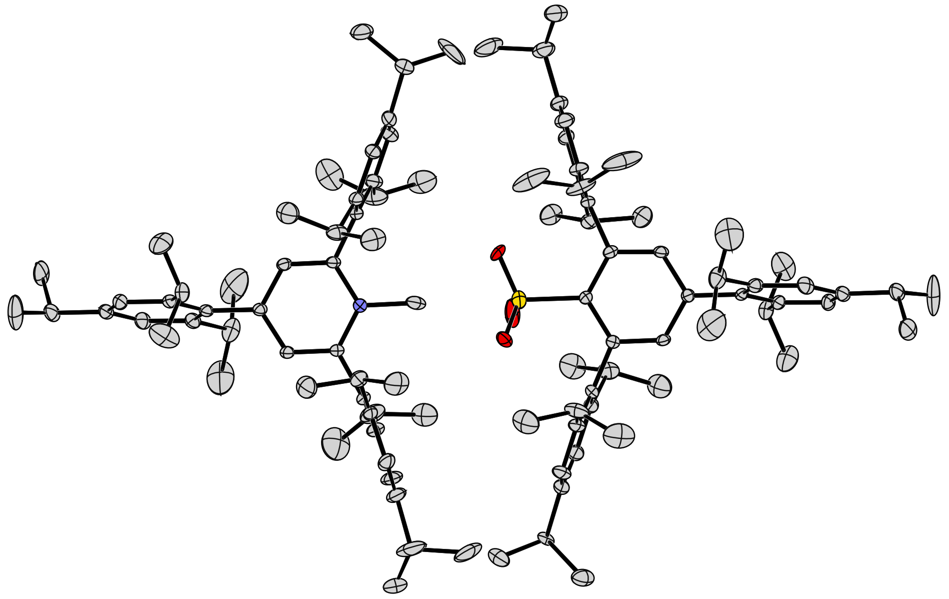

view 2:

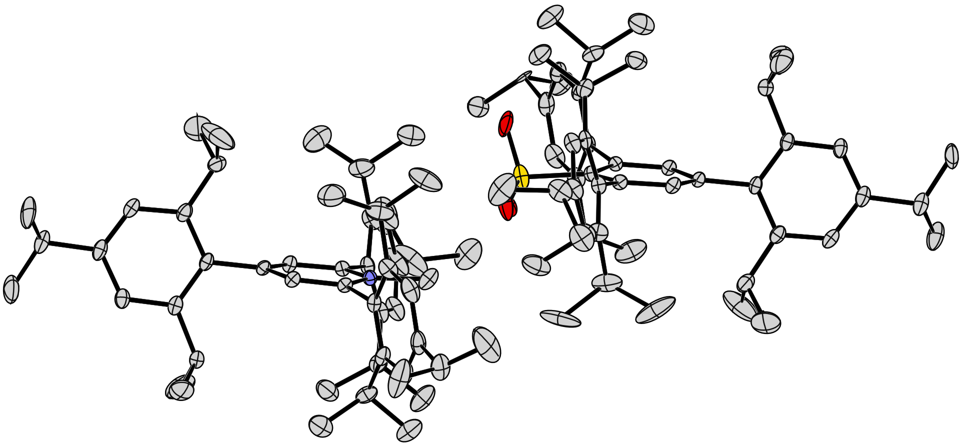

view 3:

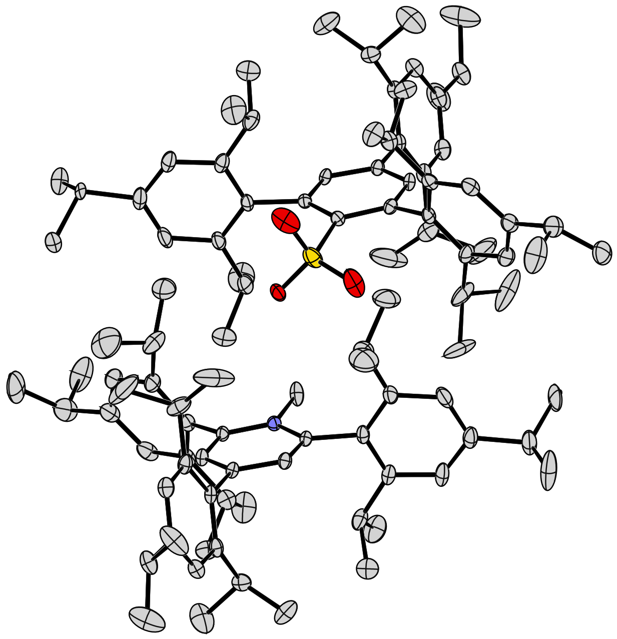

The space group of the structure of **3** ($C_{51}H_{74}N^+$ $C_{51}H_{71}O_3S^-$) is non-centrosymmetric, but achiral. Refinement of the absolute structure parameter[46] indicates that the crystals are inversion twins. The asymmetric unit contains one cation and one anion and the structure is actually very close to being centrosymmetric, with the carbon framework of the cation closely related to that of the anion by a centre of inversion. However, the potential symmetry is broken by the MeN^+– and –SO_3^- groups, which are not disordered across both entities in the asymmetric unit. Several ^{i}Pr groups and the SO_3^- group are disordered and three of the ^{i}Pr groups and the –SO_3^- group were modelled with two sets of positions for the atoms; the site occupation factors of the major conformations of these groups refined to 0.592(9), 0.67(3) and 0.853(10) for the three ^{i}Pr groups and 0.61(3) for the –SO_3^- group. Similarity restraints were applied to the chemically equivalent bond lengths involving all disordered atoms, as well as to the methyl C···C and sulfonate O···O distances within these groups. Neighbouring atoms within and between each of the disordered conformations were restrained to have similar, and for one methine C-atom pseudo-isotropic, atomic displacement parameters.

Crystallographic data

Crystallised from		*n*-hexane / benzene
Empirical formula		$C_{102}H_{145}NO_3S$
Formula weight [g mol^{-1}]		1465.24
Crystal colour, habit		colourless, prism
Crystal dimensions [mm]		0.09 × 0.12 × 0.21
Temperature [K]		160(1)
Crystal system		orthorhombic
Space group		*Pca*2$_1$ (#29)
Z		4
Reflections for cell determination		25563
2θ range for cell determination [°]		6–144
Unit cell parameters	*a* [Å]	20.7763(9)
	b [Å]	15.59946(12)
	c [Å]	28.00819(12)
	α [°]	90
	β [°]	90
	γ [°]	90
	V [Å^3]	9077.4(4)
F(000)		3216

D_x [g cm^{-3}]	1.072
μ(Cu $K\alpha$) [mm^{-1}]	0.675
Scan type	ω
$2\theta_{(max)}$ [°]	143.6
Transmission factors (min; max)	0.675; 1.000
Total reflections measured	45042
Symmetry independent reflections	16806
R_{int}	0.023
Reflections with $I > 2\sigma(I)$	15546
Reflections used in refinement	16806
Parameters refined; restraints	1102; 269
Final $R(F)$ [$I > 2\sigma(I)$ reflections]	0.0643
$wR(F^2)$ (all data)	0.1810
Weights:	$w = [\sigma^2(F_o^2) + (0.1133P)^2 + 4.3950P]^{-1}$ where $P = (F_o^2 + 2F_c^2)/3$
Goodness of fit	1.022
Final Δ_{max}/σ	0.000
$\Delta\rho$ (max; min) [e Å^{-3}]	0.60; -0.34
$\sigma(d_{(C-C)})$ [Å]	0.004 – 0.011

Crystal structure of 4

view 1:

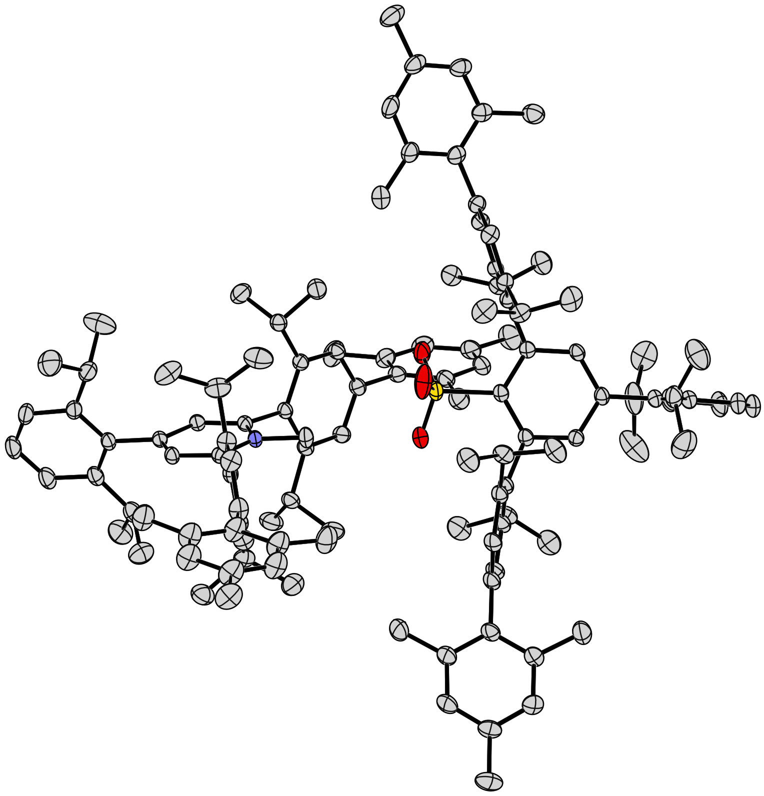

view 2:

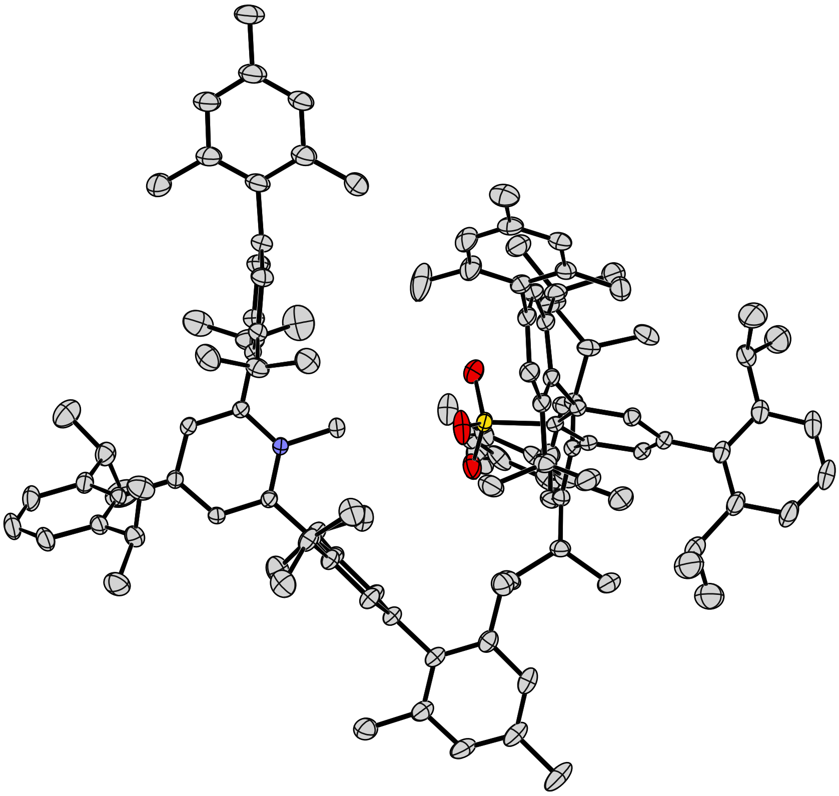

view 3:

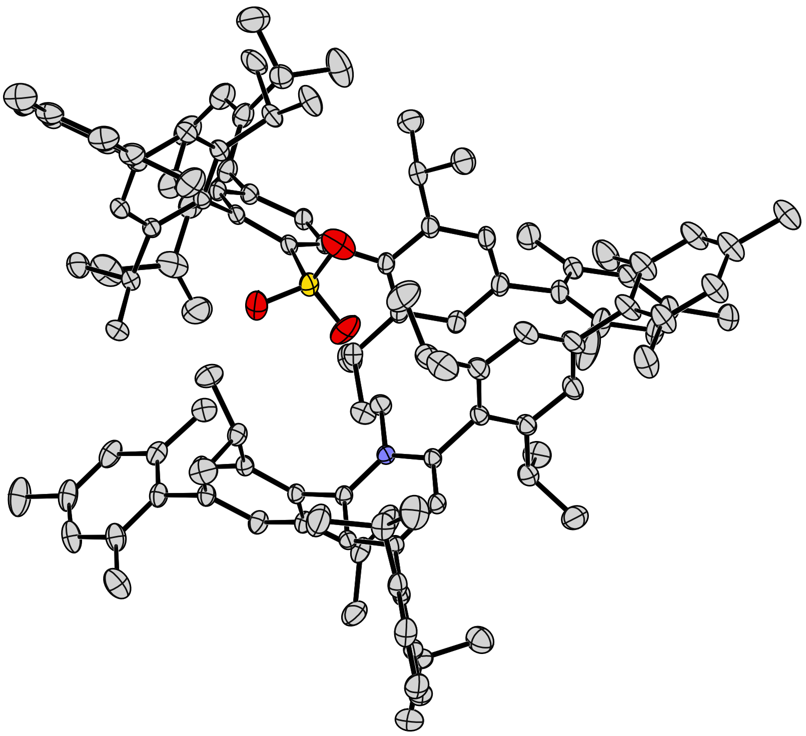

The asymmetric unit in the structure of **4** ($C_{60}H_{76}N^{+}$ $C_{60}H_{73}O_3S^{-}$ $\cdot 1.65C_6H_6 \cdot 1.63H_2O$) contains one cation, one anion, one fully occupied ordered benzene molecule on a general position, one half of a fully occupied disordered benzene molecule, which sits across a centre of inversion, another half of a partially occupied ordered benzene molecule, which also sits across a centre of inversion, and two sites for water molecules, one of which is partially occupied. For the partially occupied benzene and water molecules, the site occupation factors refined to 0.303(5) and 0.627(7), respectively. For the disordered benzene molecule, two sets of positions were defined for the symmetry-unique atoms and the site occupation factor of the major conformation refined to 0.556(8). Bond length restraints of 1.395(5) Å were applied to the C–C bond lengths within both of the benzene molecules that lie across centres of inversion, while neighbouring atoms within these benzene molecules were restrained to have similar atomic displacement parameters. One of the mesitylene groups is also disordered. Two sets of positions were defined for the atoms of this mesitylene group and the site occupation factor of the major conformation refined to 0.685(9). Similarity restraints were applied to the chemically equivalent bond lengths and angles involving all disordered C-atoms of this mesitylene group, while neighbouring atoms within and between each conformation of the disordered mesitylene group were restrained to have similar atomic displacement parameters. The water H-atoms could not be located in any difference electron density map. Three of the four water H-atoms were placed in calculated positions which yielded the best hydrogen bonding interactions with anion O-atoms or a neighbouring water molecule and their positions were refined using a riding model. The fourth water H-atom was omitted from the model. All remaining H-atoms were placed in geometrically calculated positions and refined using a riding model.

Crystallographic data

Crystallised from	benzene / heptane

Empirical formula		$C_{129.91}H_{162.11}NO_{4.60}S$
Formula weight [g mol^{-1}]		1843.27
Crystal colour, habit		colourless, needle
Crystal dimensions [mm]		0.04 × 0.07 × 0.27
Temperature [K]		160(1)
Crystal system		triclinic
Space group		$P\bar{1}$ (#2)
Z		2
Reflections for cell determination		42204
2θ range for cell determination [°]		7–149
Unit cell parameters	a [Å]	12.8742(2)
	b [Å]	17.7178(2)
	c [Å]	26.5501(5)
	α [°]	101.7022(13)
	β [°]	94.6920(12)
	γ [°]	104.1168(15)
	V [Å^3]	5695.96(16)
$F(000)$		2003
D_x [g cm^{-3}]		1.075
μ(Cu $K\alpha$) [mm^{-1}]		0.643
Scan type		ω
$2\theta_{(max)}$ [°]		149.0
Transmission factors (min; max)		0.760; 1.000
Total reflections measured		106920
Symmetry independent reflections		22424
R_{int}		0.034
Reflections with $I > 2\sigma(I)$		18536
Reflections used in refinement		22424
Parameters refined; restraints		1403; 424
Final	$R(F)$ [$I > 2\sigma(I)$ reflections]	0.0539
	$wR(F^2)$ (all data)	0.1631

Weights:	$w = [\sigma^2(F_O^2) + (0.0905P)^2 + 1.8135P]^{-1}$ where $P = (F_O^2 + 2F_C^2)/3$
Goodness of fit	1.031
Final Δ_{max}/σ	0.001
$\Delta\rho$ (max; min) [e Å^{-3}]	0.78; -0.48
$\sigma(d_{(C–C)})$ [Å]	0.0019 – 0.014

Crystal structure of 5

view 1:

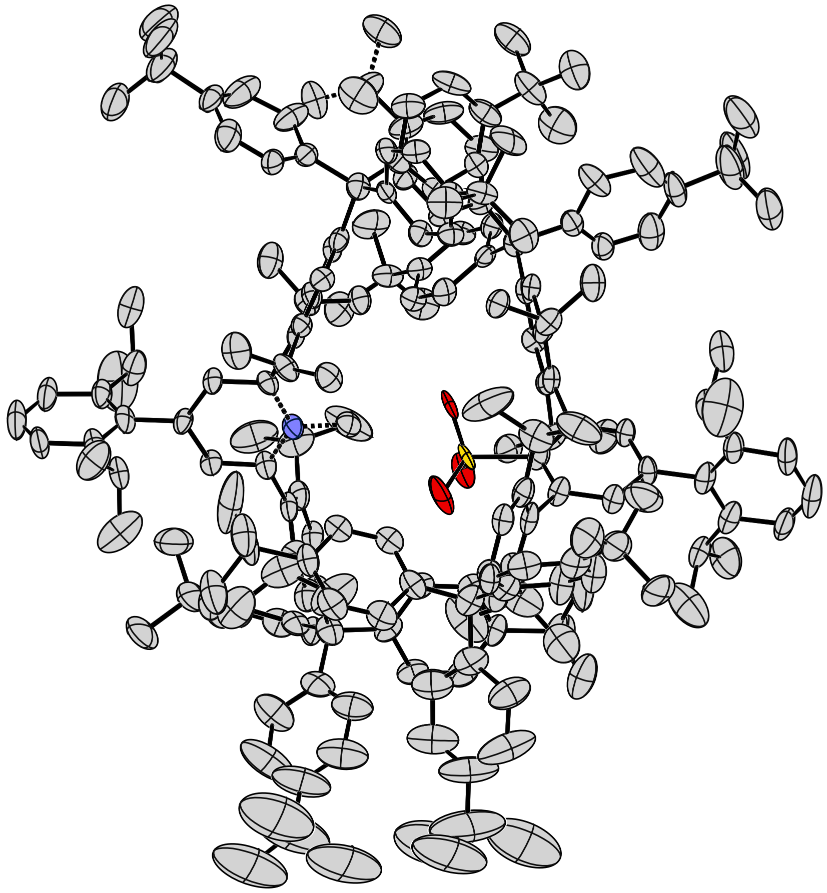

view 2:

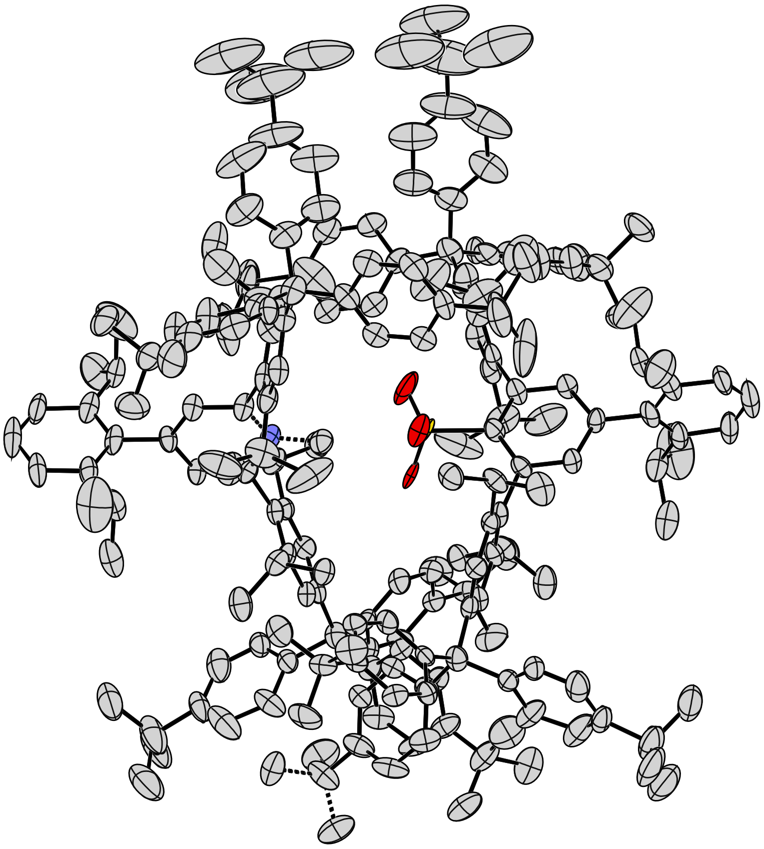

view 3:

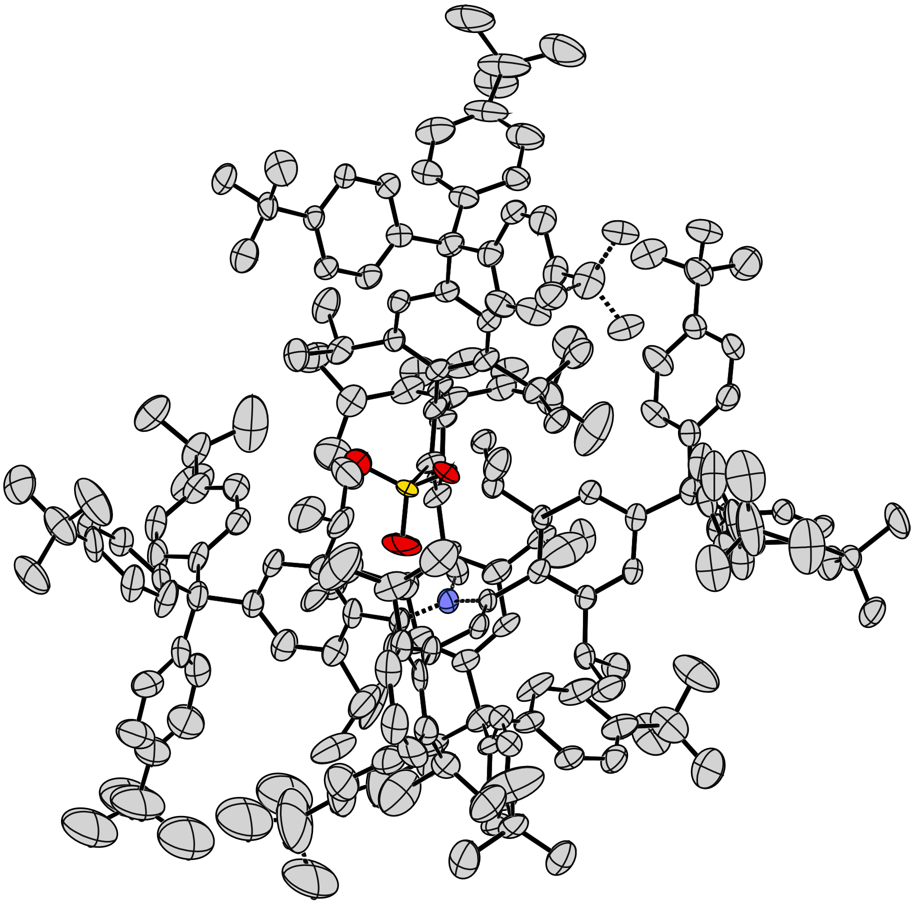

The structure of **5** ($C_{104}H_{132}N^+$ $C_{104}H_{129}O_3S^-$ $\cdot 30H_2O$) is an inversion twin; refinement of the absolute structure parameter[14] yielded a value of 0.53(2). In the structure, the cation and anion, being topologically almost identical, occupy the same sites in the crystal structure, so the C–SO_3 and N-Me groups are disordered. There are two such sites in the asymmetric unit and short contacts dictate that the cation:anion distribution at each site is 1:1. Each of these independent sites displays further disorder of some *t*-butyl groups, two in the ions at one site, three in the other. Two sets of positions were defined for the atoms of each disordered *t*-butyl group and for the disordered C–SO_3 and N-Me groups. The site occupation factors for the *t*-butyl groups were refined, while their sums were constrained to 1.0 within each disordered entity. Similarity restraints were applied to the C–C bond lengths and $C_{methyl}\cdots C_{methyl}$ distances involving all disordered *t*-butyl groups, while neighbouring atoms within and between each conformation of the disordered *t*-butyl groups were restrained to have similar atomic displacement parameters. Analogous similarity restraints were also applied to the N–C, C–S, S–O and O···O distances. For two *t*-butyl groups and the C–S and O-atoms, pseudo-isotropic restraints were additionally applied to the atomic displacement parameters, while rigid bond restraints were applied to all atoms in the structure model. Furthermore, the positions and atomic displacement parameters of the perfectly overlapping C- and N-atoms were constrained to be identical.

There are large solvent regions in the structure. Although the crystals were grown from hexane, no residual electron density peaks could be discerned that were indicative of hexane molecules. Instead, the

peaks were scattered throughout the solvent region with no recognisable pattern and were sometimes close to one another suggesting disorder. Consequently, the solvent mask routine of the program *OLEX2*[47] was employed to account for all solvent molecules. For convenience, it was assumed that all solvent molecules were water, although without further elemental or thermogravimetric analyses this is pure assumption. Omission of the solvent molecules leaves one cavity of 13925 Å^3 per unit cell. The total number of electrons contributing to this void in the unit cell was calculated to be approximately 1876 e. Allowing for 180 molecules of water per void yields 1800 e and this approximation was used in the subsequent calculation of the empirical formula, formula weight, density, linear absorption coefficient and F(000). Based on the assumption, the overall ratio of cations:anions:toluene:water is 1:1:30. However, the estimated solvent content is possibly too low, because the calculated density is lower than might be expected.

Crystallographic data

Crystallised from		hexane
Empirical formula		$C_{208}H_{321}NO_{33}S$
Formula weight [g mol^{-1}]		3395.70
Crystal colour, habit		colourless, prism
Crystal dimensions [mm]		0.11 × 0.15 × 0.23
Temperature [K]		160(1)
Crystal system		trigonal
Space group		*P*321 (#150)
Z		6
Reflections for cell determination		63910
2θ range for cell determination [°]		6–146
Unit cell parameters	*a* [Å]	35.75342(15)
	b [Å]	35.75342(15)
	c [Å]	33.28987(10)
	α [°]	90
	β [°]	90
	γ [°]	120
	V [Å^3]	36853.4(3)
F(000)		11136
D_x [g cm^{-3}]		0.918
μ(Cu *K*α) [mm^{-1}]		0.555

Scan type	ω
$2\theta_{(max)}$ [°]	146.1
Transmission factors (min; max)	0.709; 1.000
Total reflections measured	128470
Symmetry independent reflections	40388
R_{int}	0.015
Reflections with $I > 2\sigma(I)$	35260
Reflections used in refinement	40388
Parameters refined; restraints	2180; 3191
Final $R(F)$ [$I > 2\sigma(I)$ reflections]	0.0454
$wR(F^2)$ (all data)	0.1459
Weights:	$w = [\sigma^2(F_o^2) + (0.0940P)^2 + 2.1344P]^{-1}$ where $P = (F_o^2 + 2F_c^2)/3$
Goodness of fit	1.084
Final Δ_{max}/σ	0.001
$\Delta\rho$ (max; min) [e Å^{-3}]	0.35; -0.21
$\sigma(d_{(C–C)})$ [Å]	0.003 – 0.008

Crystal structure of 6

view 1:

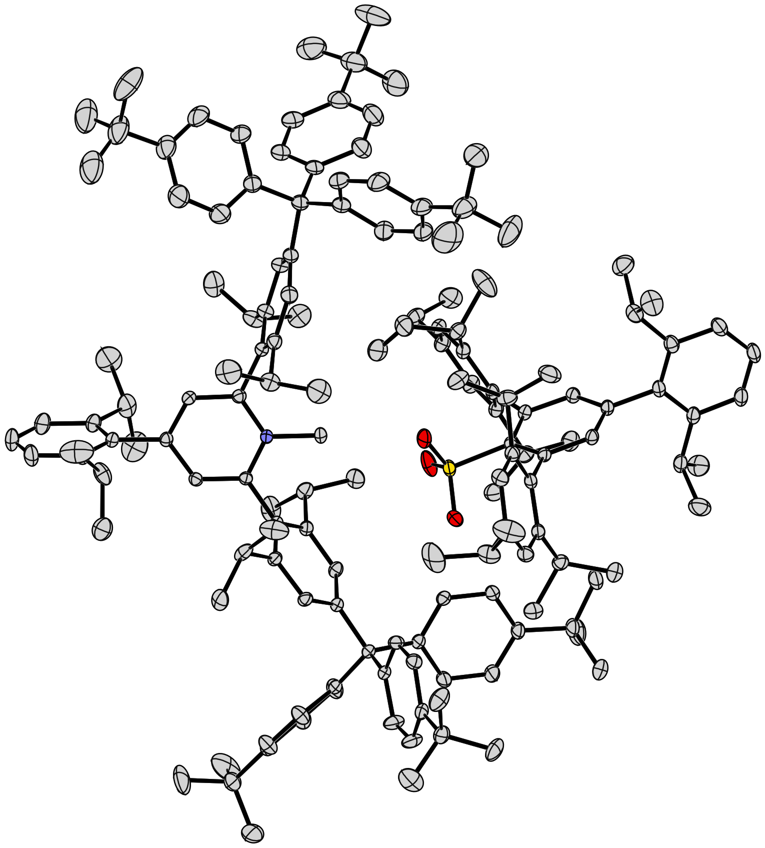

view 2:

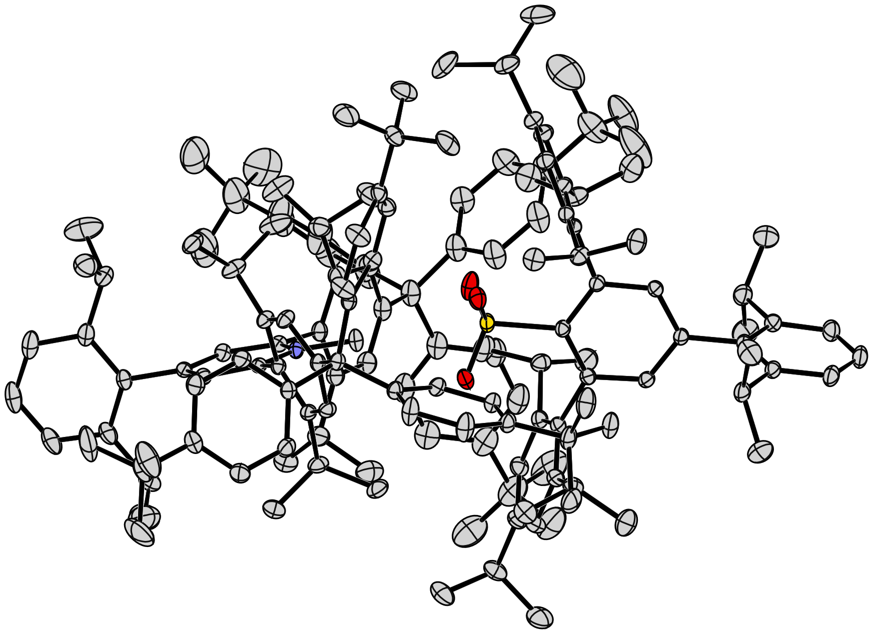

view 3:

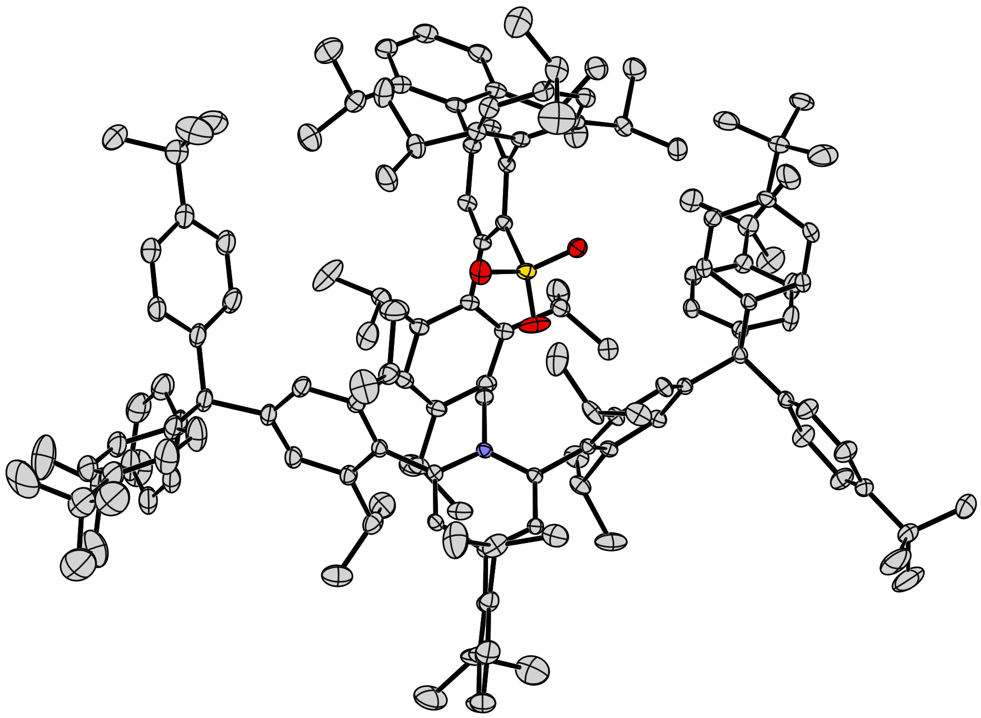

The asymmetric unit in the structure of **6** ($C_{104}H_{132}N^{+}$ $C_{48}H_{65}O_3S^{-}$ $\cdot 3.5(C_7H_8)\cdot H_2O$) contains one cation, one anion, one water molecule and 3.5 molecules of toluene, one of which is disordered across a centre of inversion, and a further two of which are also disordered. A disorder model for one of the latter toluene molecules could not be established, so the *SQUEEZE* routine of the program *PLATON*[45] was employed to account for this toluene molecule. Omission of this one toluene molecule leaves one cavity of 694 Å^3 per unit cell, centred about a centre of inversion. The total number of electrons contributing to this void in the unit cell was calculated by the *SQUEEZE* routine to be approximately 112 e. Several additional tiny voids are also present, but contain a zero electron count. Allowing for two molecules of toluene per void yields 100 e and this approximation was used in the subsequent calculation of the empirical formula, formula weight, density, linear absorption coefficient and F(000). Based on the assumption, the overall ratio of cations:anions:toluene:water is 1:1:3.5:1.

Two *t*-butyl groups in the cation are disordered over two orientations. Two sets of positions were defined for the atoms of each disordered *t*-butyl group and the site occupation factors of the major conformations of these groups refined to 0.706(6) and 0.897(4) for the disordered group at C(23) and C(33), respectively. Similarity restraints were applied to the chemically equivalent bond lengths and C···C distances involving all disordered C-atoms, while neighbouring atoms within and between each orientation of the disordered *t*-butyl groups were restrained to have similar and pseudo-isotropic atomic displacement parameters. For the modelled disordered toluene molecule in a general position, two sets of positions were defined for the atoms and the site occupation factor of the major orientation refined to 0.523(6). For the toluene molecule disordered about a centre of inversion, a single full molecule was defined with all atoms having a site occupation factor of 0.5; the inversion symmetry generating the other orientation of the molecule. For both disordered toluene molecules, the ring atoms were constrained to a perfect hexagon and, for

the toluene molecule in a general position, the ring-methyl C–C bonds were restrained to be similar. For all modelled toluene molecules, neighbouring atoms were restrained to have similar atomic displacement parameters. The non-hydrogen atoms were refined anisotropically. Only one potential position for a water H-atom could be located in a difference electron density map and its position was allowed to refine while applying an O–H distance restraint of 1.84(1) Å. The other water H-atom was not included in the model. A correction for secondary extinction was applied.

Crystallographic data

Crystallised from		toluene
Empirical formula		$C_{176.5}H_{227}NO_4S$
Formula weight [g mol^{-1}]		2458.64
Crystal colour, habit		colourless, prism
Crystal dimensions [mm]		0.05 × 0.06 × 0.13
Temperature [K]		160(1)
Crystal system		triclinic
Space group		$P\bar{1}$ (#2)
Z		2
Reflections for cell determination		54954
2θ range for cell determination [°]		6–147
Unit cell parameters	*a* [Å]	15.1995(2)
	b [Å]	15.9154(2)
	c [Å]	34.2180(5)
	α [°]	87.6311(11)
	β [°]	79.9452(12)
	γ [°]	73.6514(12)
	V [Å^3]	7820.60(19)
F(000)		2682
D_x [g cm^{-3}]		1.044
μ(Cu *K*α) [mm^{-1}]		0.572
Scan type		ω
$2\theta_{(max)}$ [°]		146.1
Transmission factors (min; max)		0.882; 1.000
Total reflections measured		133077

Symmetry independent reflections	30364
R_{int}	0.022
Reflections with $I > 2\sigma(I)$	25964
Reflections used in refinement	30364
Parameters refined; restraints	1744; 512
Final $R(F)$ [$I > 2\sigma(I)$ reflections]	0.0546
$wR(F^2)$ (all data)	0.1659
Weights:	$w = [\sigma^2(F_o^2) + (0.0936P)^2 + 2.8180P]^{-1}$ where $P = (F_o^2 + 2F_c^2)/3$
Goodness of fit	1.046
Secondary extinction coefficient	0.00017(4)
Final Δ_{max}/σ	0.000
$\Delta\rho$ (max; min) [e Å^{-3}]	0.72; -0.50
$\sigma(d_{(C-C)})$ [Å]	0.0018 – 0.005

Crystal structure of 7

view 1:

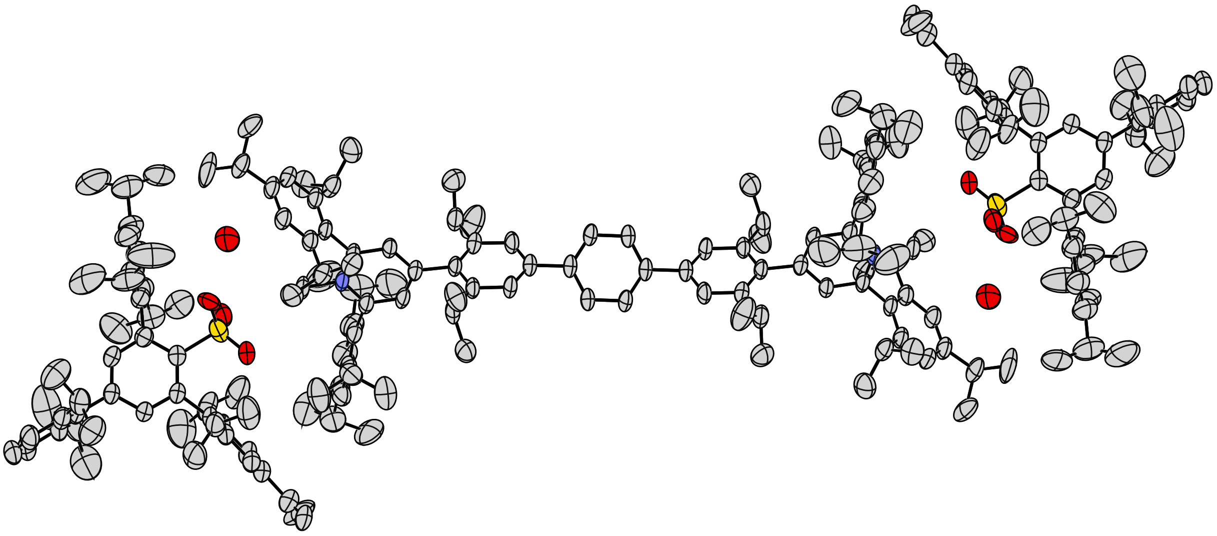

view 2:

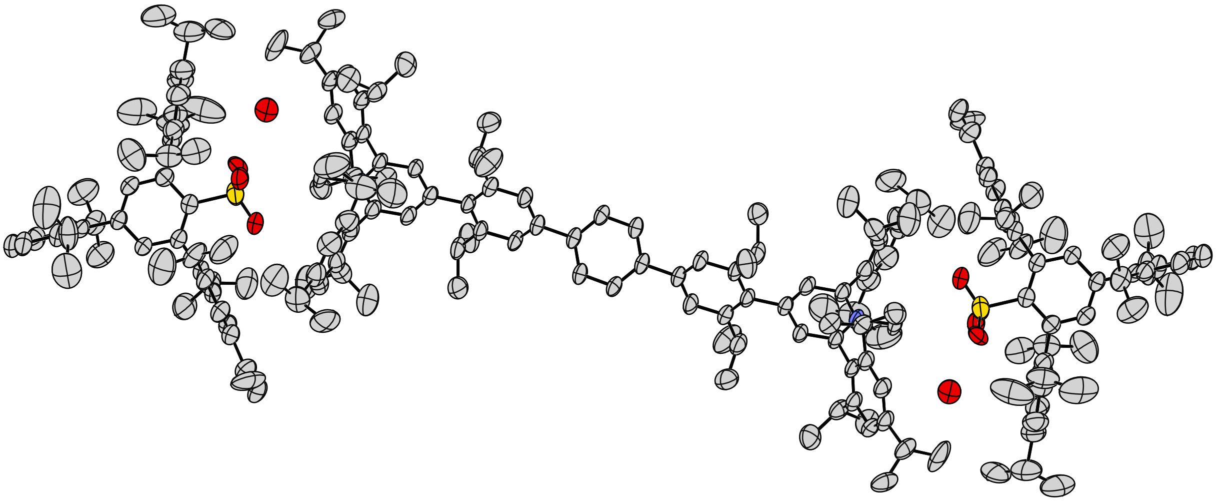

For the structure of **7** ($C_{102}H_{138}N_2^{2+}$ $(C_{48}H_{65}O_3S^-)_2\cdot 7C_6H_6\cdot 2H_2O$), the overall quality of the results is poorer than usual. This may in part be due to extensive disorder in the ^{i}Pr groups and several disordered solvent molecules of benzene. The asymmetric unit contains one half of a centrosymmetric cation, one anion, one water molecule and 3.5 disordered molecules of benzene, where one of these molecules sits across a crystallographic centre of inversion. Although the benzene molecules could be discerned clearly from the residual electron density peaks, they are quite disordered and the best refinement results were obtained when their contribution to the diffraction data was removed using the *SQUEEZE* routine of the program *PLATON*.[45] When the solvent molecules are excluded from the model, there is one centrosymmetric cavity of 843 Å^3 and one centrosymmetric cavity of 332 Å^3 per unit cell. The total number of electrons contributing to these voids was calculated by the *SQUEEZE* routine to be approximately 193 and 85 e, respectively. A benzene molecule has 42 e which suggests that the cavities have a total of seven molecules of benzene per unit cell (3.5 per asymmetric unit) and this approximation was used in the subsequent calculation of the empirical formula, formula weight, density, linear absorption coefficient and

F(000). Based on the assumption, the overall ratio of cations:anions:benzene:water is 1:2:7:2. Although there is probably some degree of disorder among most of the ^{i}Pr groups, disorder was modelled for just two ^{i}Pr groups as well as for the sulfonate O-atoms. Two sets of positions were defined for the atoms of each disordered group and the site occupation factors of the major conformations of these groups refined to 0.596(11), 0.658(12) (^{i}Pr groups) and 0.537(10) (sulfonate O-atoms). Similarity restraints were applied to the chemically equivalent bond lengths involving all disordered atoms, as well as to the sulfonate O···O distances, while neighbouring atoms within and between each of the disordered conformations were restrained to have similar atomic displacement parameters. The H-atoms of the water molecule were placed in the positions indicated by a difference electron density map and the water molecule was refined as a rigid group. All other H-atoms were placed in geometrically calculated positions and refined using a riding model. A numerical absorption correction was applied.

Crystallographic data

Crystallised from		mesitylene / *n*-hexane / *n*-pentane / benzene
Empirical formula		$C_{240}H_{314}N_2O_8S_2$
Formula weight [g mol^{-1}]		3419.03
Crystal colour, habit		pale-yellow, prism
Crystal dimensions [mm]		0.14 × 0.18 × 0.22
Temperature [K]		160(1)
Crystal system		triclinic
Space group		$P\bar{1}$ (#2)
Z		1
Reflections for cell determination		24800
2θ range for cell determination [°]		5–146
Unit cell parameters	*a* [Å]	15.4354(4)
	b [Å]	16.6547(5)
	c [Å]	22.8322(6)
	α [°]	83.077(2)
	β [°]	78.545(2)
	γ [°]	66.641(3)
	V [Å^3]	5275.4(3)
F(000)		1864
D_x [g cm^{-3}]		1.076
μ(Cu $K\alpha$) [mm^{-1}]		0.654

Scan type	ω
$2\theta_{(max)}$ [°]	146.5
Transmission factors (min; max)	0.614; 1.000
Total reflections measured	76688
Symmetry independent reflections	20551
R_{int}	0.045
Reflections with $I > 2\sigma(I)$	14687
Reflections used in refinement	20551
Parameters refined; restraints	1041; 159
Final $R(F)$ [$I > 2\sigma(I)$ reflections]	0.0951
$wR(F^2)$ (all data)	0.3180
Weights:	$w = [\sigma^2(F_o^2) + (0.1680P)^2 + 3.0737P]^{-1}$ where $P = (F_o^2 + 2F_c^2)/3$
Goodness of fit	1.085
Final Δ_{max}/σ	0.000
$\Delta\rho$ (max; min) [e Å^{-3}]	1.14; -0.42
$\sigma(d_{(C-C)})$ [Å]	0.003 – 0.011

Crystal structure of 9

view 1:

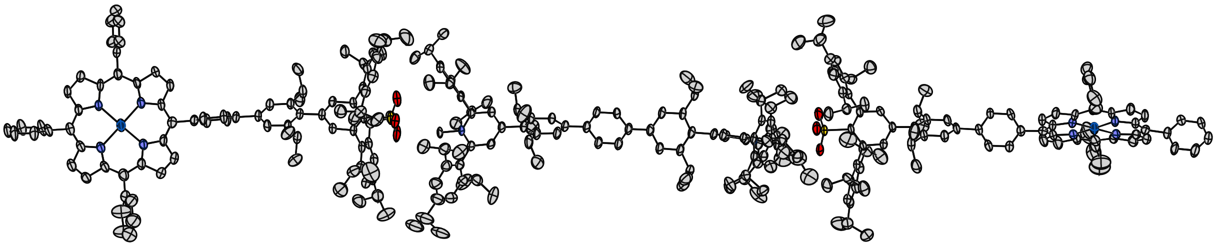

view 2:

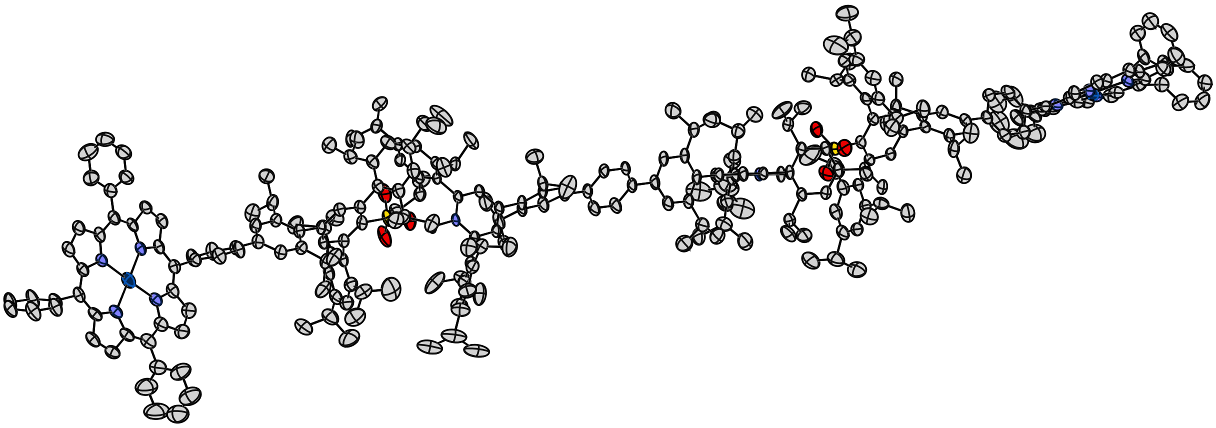

view 3:

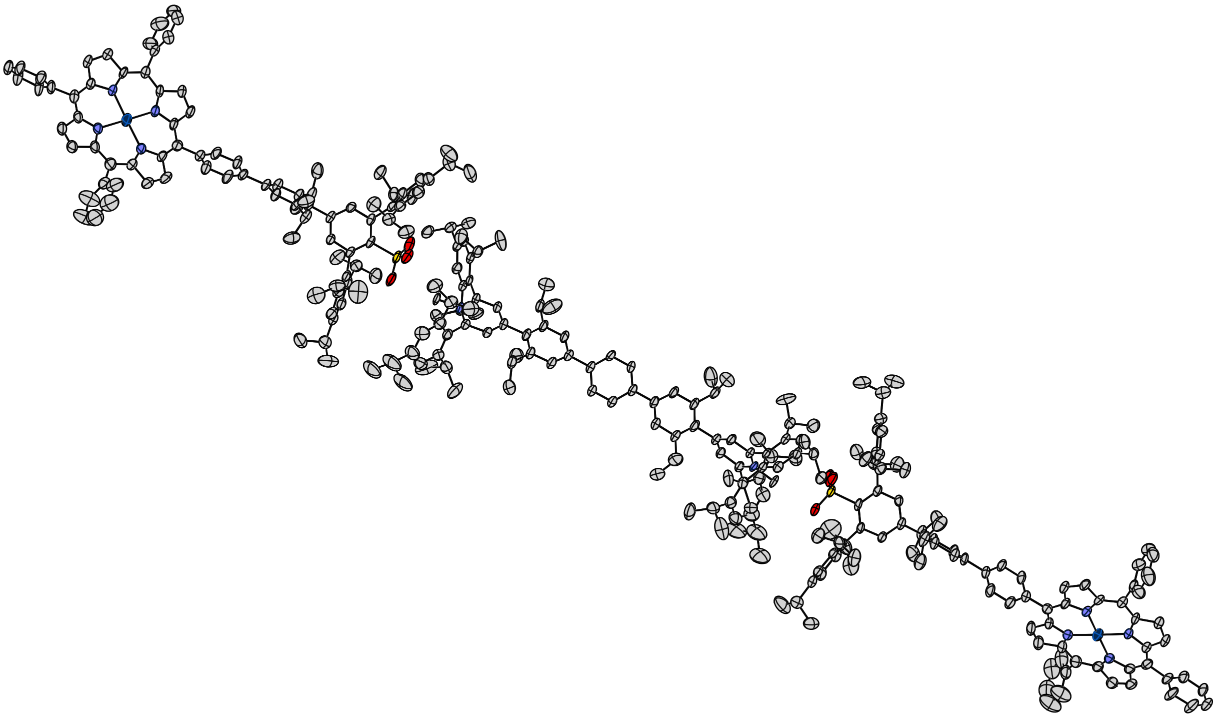

The crystal of **9** $[C_{102}H_{138}N_2^{2+}][C_{92}H_{91}N_4O_3PdS^-]_2 \cdot 2(H_2O) \cdot 10(C_7H_8)$ was weakly diffracting and many of the high angle reflections have barely measurable intensities. Only data to a resolution of 0.90 Å was used during refinement. In addition, the crystal appeared to suffer decay during the latter part of the data collection, as was apparent from an increase in R_{int} and unstable frame scaling. A reasonable value for R_{int} and good frame scaling was obtained when only the first 15 of the 19 runs were used (3558 out of 4410

frames). Nonetheless, almost full completeness of the data to the desired resolution was attained. Overall, the final results are of modest quality. The geometries of the main species present are clearly defined, but the precision of the geometric parameters is modest and could be of limited accuracy where disorder has not been modelled. The asymmetric unit contains one half of a C_2-symmetric cation, one anion, one water molecule and an estimated five molecules of toluene. Only some of the toluene molecules were fully visible in difference electron density maps and, even then, they showed evidence of being disordered. Therefore, the contribution of all toluene solvent molecules to the diffraction data was removed using the *SQUEEZE* routine of the program *PLATON*.[45] When the solvent molecules are excluded from the model, there are two main cavities of 4806 Å^3 per unit cell. The total number of electrons contributing to each of these voids was calculated by the *SQUEEZE* routine to be approximately 1018 e. A toluene molecule has 50 e which suggests that each cavity contains twenty molecules of toluene (5 per asymmetric unit) and this approximation was used in the subsequent calculation of the empirical formula, formula weight, density, linear absorption coefficient and F(000). Based on the assumption, the overall ratio of cation:anion:water:toluene is 1:2:2:10. Some of the ^{i}Pr groups and porphyrin phenyl rings are probably disordered, but this disorder was only modelled for two of the ^{i}Pr groups. Two sets of positions were defined for the atoms of these two groups and the site occupation factors of the major conformations of the groups at C134 and C137 refined to 0.543(17) and 0.67(6), respectively. Similarity restraints were applied to the chemically equivalent bond lengths involving all disordered C-atoms, as well as to the C···C distances between the disordered terminal methyl C-atoms. Neighbouring atoms within and between each of the disordered conformations were restrained to have similar atomic displacement parameters. A numerical absorption correction was applied.

Crystallographic data

Crystallised from		toluene / *n*-heptane
Empirical formula		$C_{356}H_{404}N_{10}O_8Pd_2S_2$
Formula weight [g mol^{-1}]		5227.79
Crystal colour, habit		red, plate
Crystal dimensions [mm]		0.07 × 0.11 × 0.43
Temperature [K]		160(1)
Crystal system		monoclinic
Space group		*I*2/*a* (#15)
Z		4
Reflections for cell determination		38697
2θ range for cell determination [°]		4–147
Unit cell parameters	*a* [Å]	31.1028(2)
	b [Å]	23.6402(2)

c [Å]	44.8209(5)
α [°]	90
β [°]	104.6052(9)
γ [°]	90
V [Å^3]	31890.8(5)
$F(000)$	11192
D_x [g cm^{-3}]	1.089
μ(Cu $K\alpha$) [mm^{-1}]	1.465
Scan type	ω
$2\theta_{(max)}$ [°]	133.2
Transmission factors (min; max)	0.273; 1.000
Total reflections measured	120757
Symmetry independent reflections	28085
R_{int}	0.039
Reflections with $I > 2\sigma(I)$	18077
Reflections used in refinement	28085
Parameters refined; restraints	1462; 82
Final $R(F)$ [$I > 2\sigma(I)$ reflections]	0.1025
$wR(F^2)$ (all data)	0.3605
Weights:	$w = [\sigma^2(F_o^2) + (0.2000P)^2]^{-1}$ where $P = (F_o^2 + 2F_c^2)/3$
Goodness of fit	1.372
Final Δ_{max}/σ	0.001
$\Delta\rho$ (max; min) [e Å^{-3}]	1.99; -1.58
$\sigma(d_{(C-C)})$ [Å]	0.006 – 0.016

Crystal structure of 10

view 1:

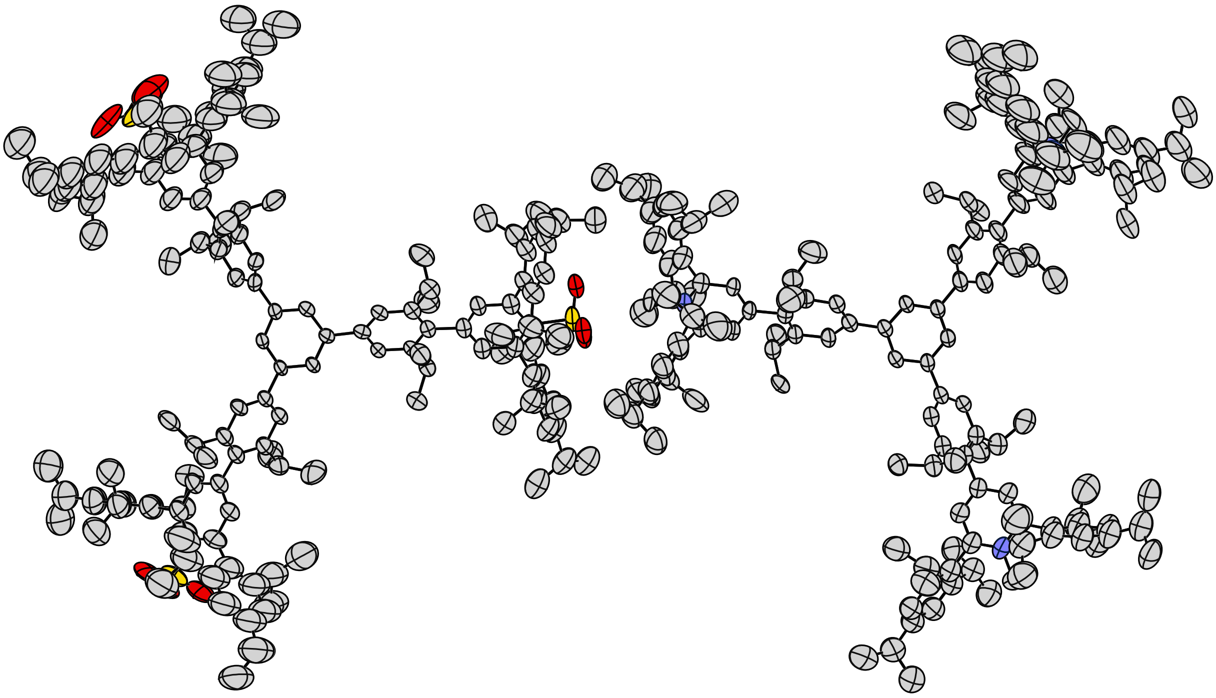

view 2:

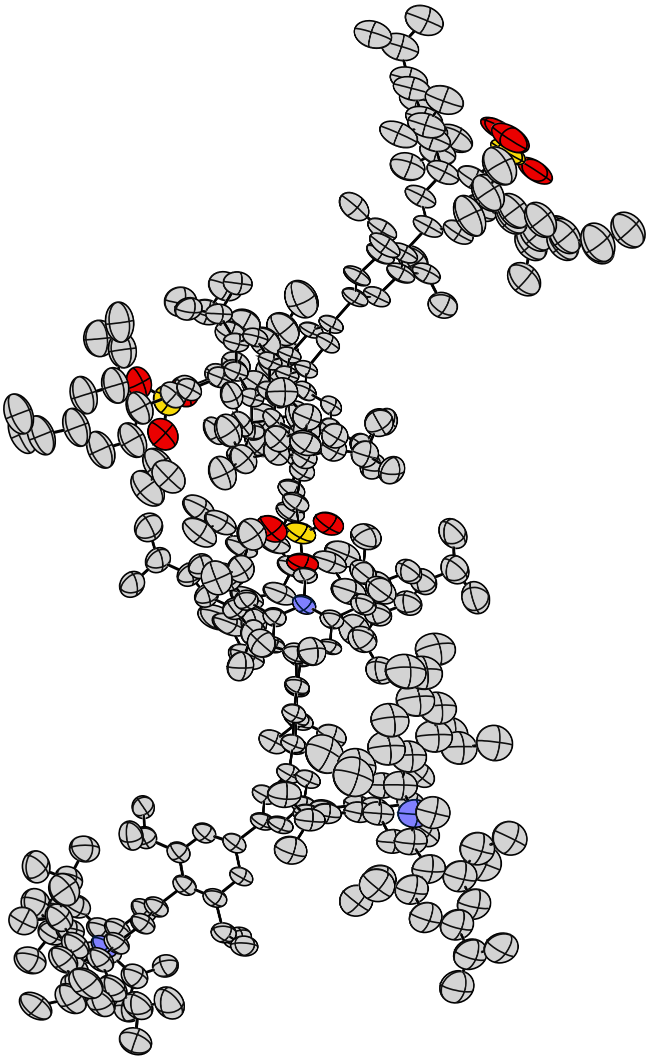

view 3:

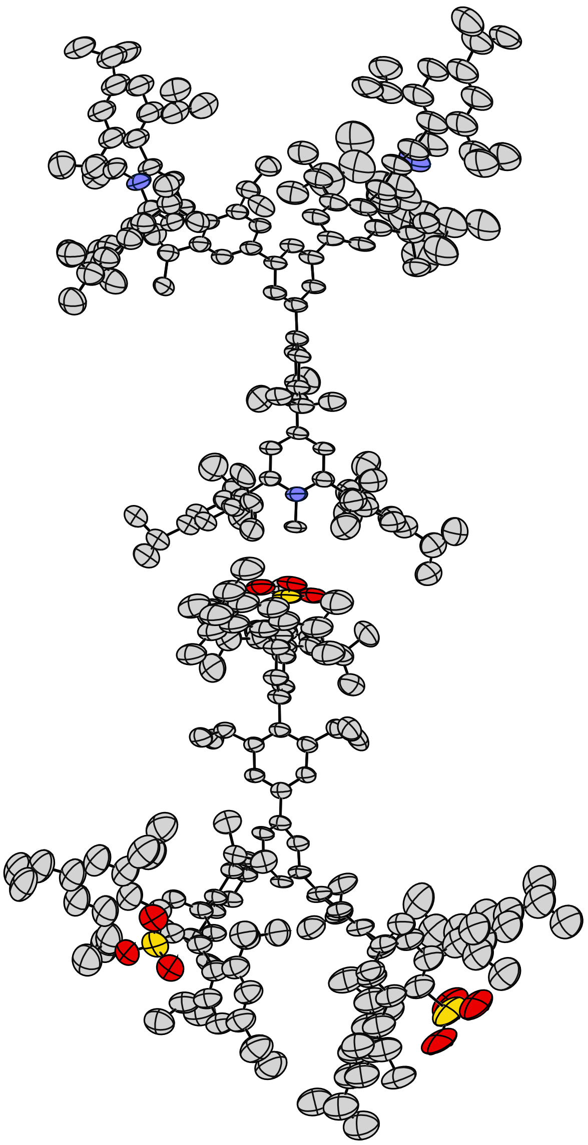

view 4 (hexagon):

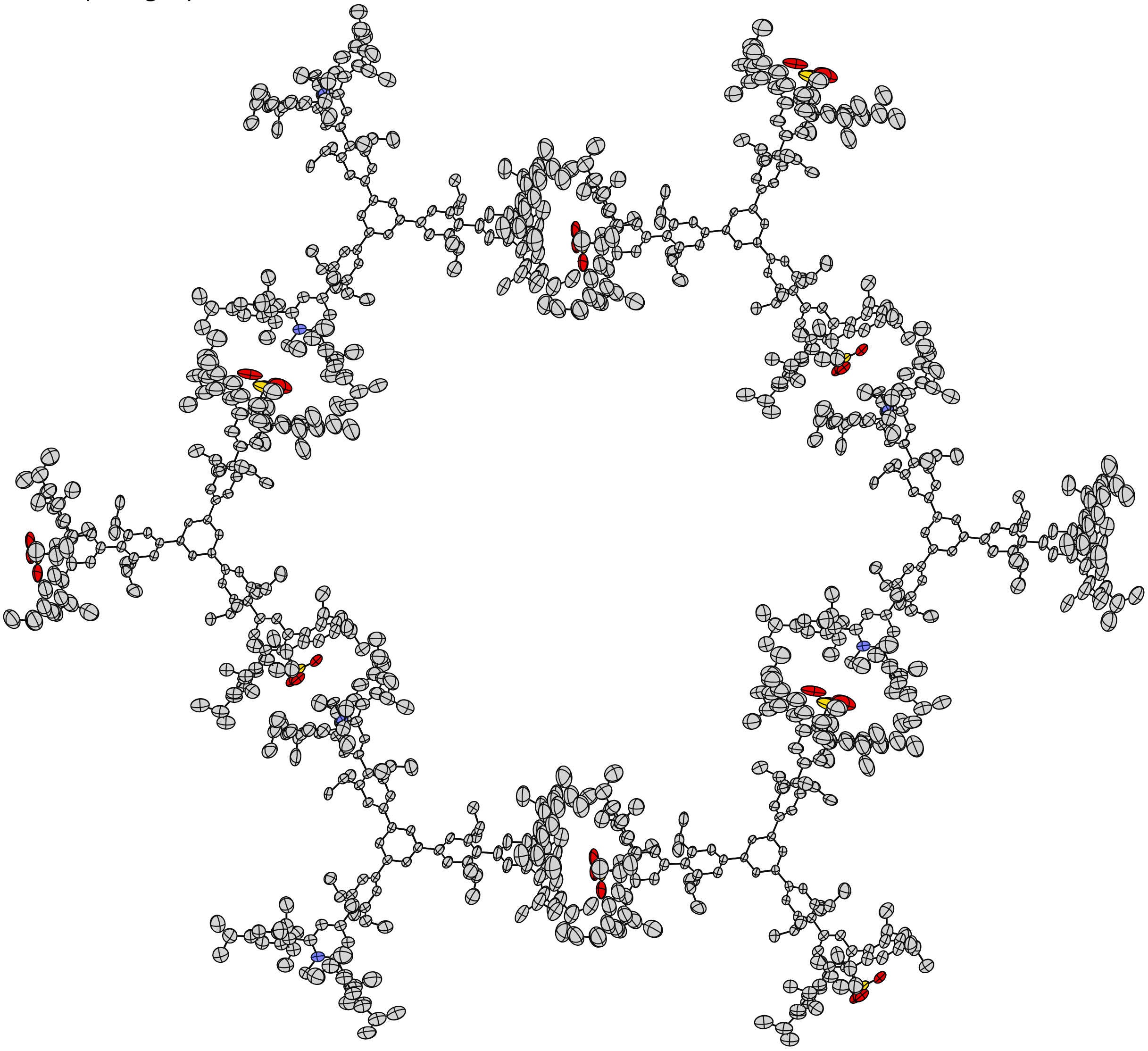

view 5 (hexagon):

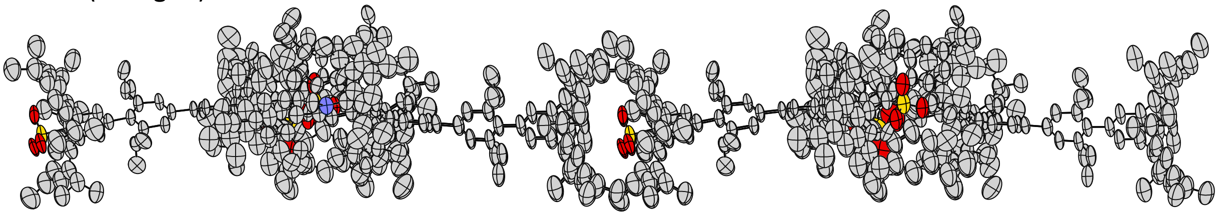

Crystal packing in **10**, (100) view:

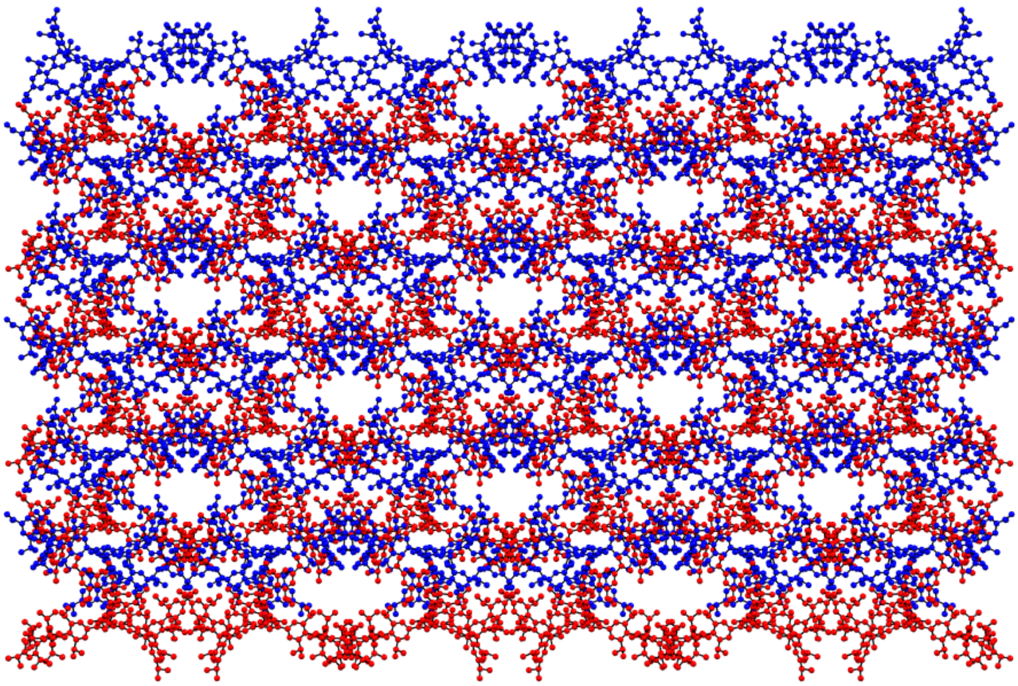

Crystal packing in **10**, (010) view:

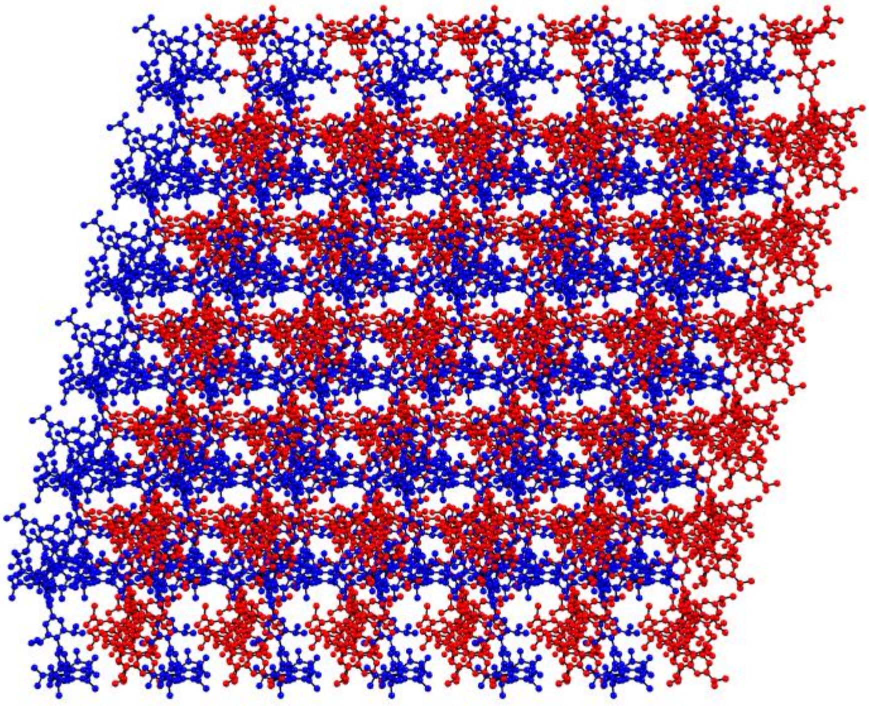

Crystal packing in **10**, (001) view:

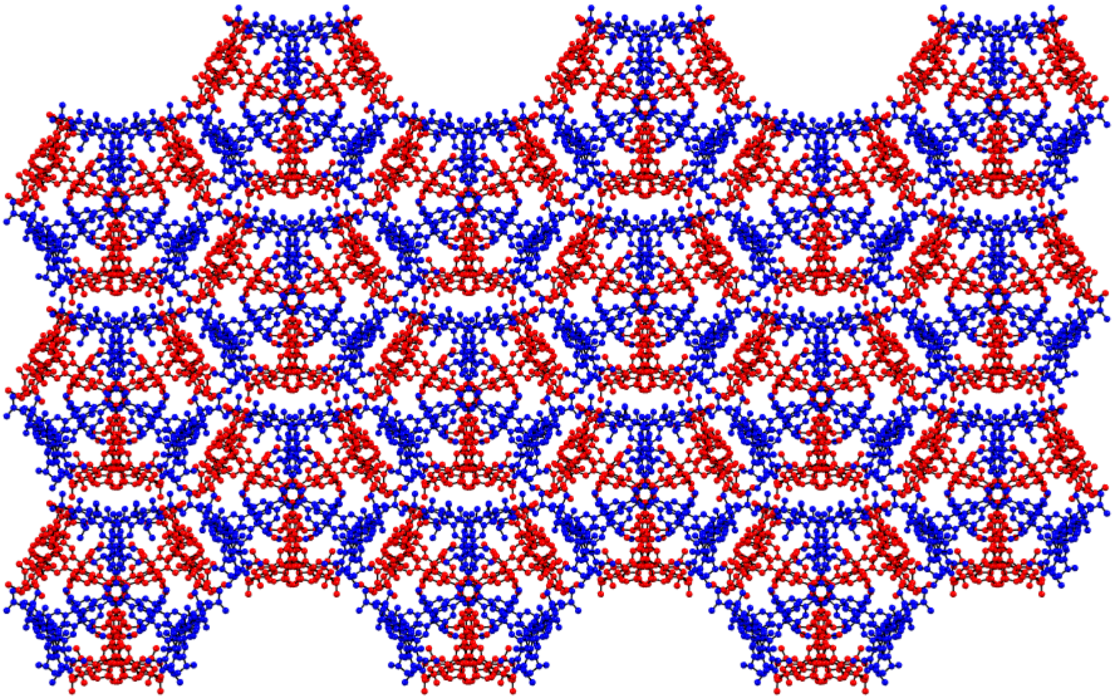

The structure **10** ($C_{150}H_{204}N_3^+$ $C_{150}H_{195}O_9S_3^-$ [+ solvent]) has been solved and refined successfully, albeit the overall quality of the results is modest. All crystals tried were very weakly diffracting, despite a good size, because of the very large cavities with disordered solvent and disorder of some arms of the ions. For the best crystal tried, for which the results are reported here, no diffraction was observed at all beyond a resolution of 1.10 Å, so the data were truncated at this resolution. The space group is non-centrosymmetric, but achiral, because of the presence of glide planes. The absolute structure has been determined by the diffraction experiment and indicates that the chosen crystal was a partial inversion twin. Two of the three sulfonate groups have been modelled as disordered with two positions for each of the O-atoms. All rings outside the central four of each ion were constrained to be perfect hexagons. All chemically equivalent C–C bonds between the rings, C(methyl)–N bonds, S–O bonds, O···O distances within the sulfonate groups, and C–C bonds involving isopropyl groups were restrained to be of similar length (SADI restraints). All non-H atoms were restrained to have similar atomic displacement parameters to those of their immediate neighbours (SIMU restraints). Anti-bumping restraints were used to prevent unreasonably short intra- and intermolecular H···H contacts and to generally stabilise the refinement convergence and the geometry of the isopropyl groups. A solvent mask was used to account for the highly diffuse electron density in the solvent accessible voids in the structure, with 2563 electrons accounted for, 2545 of which occurred in one large void per unit cell of 10828 Å^{-3}. This corresponds with approximately 8.5 molecules of trifluorotoluene per asymmetric unit. The absence of large residual electron density peaks before applying the solvent mask suggests that there is no CH_2Cl_2 in the solvent region.

Crystallographic data:

Crystallised from		trifluorotoluene / CH_2Cl_2
Empirical formula		$C_{300}H_{399}N_3O_9S_3$ [+ $8.5C_7H_5F_3$]
Formula weight [g mol^{-1}]		5529.31
Crystal colour, habit		colourless, block
Crystal dimensions [mm]		$0.12 \times 0.16 \times 0.22$
Temperature [K]		160(1)
Crystal system		monoclinic
Space group		*Ia* (#9)
Z		4
Reflections for cell determination		22453
2θ range for cell determination [°]		5–90
Unit cell parameters	*a* [Å]	26.4108(3)
	b [Å]	46.3352(5)
	c [Å]	28.9924(6)
	α [°]	90
	β [°]	104.7669(16)
	γ [°]	90
	V [Å^3]	34307.6(9)
F(000)		11876
D_x [g cm^{-3}]		1.071
μ(Cu $K\alpha$) [mm^{-1}]		0.740
Scan type		ω
$2\theta_{(max)}$ [°]		89.2
Transmission factors (min; max)		0.826; 1.000
Total reflections measured		41157
Symmetry independent reflections		22731
R_{int}		0.023

Reflections with $I > 2\sigma(I)$	19530
Reflections used in refinement	22731
Parameters refined; restraints	2677; 16148
Final $R(F)$ [$I > 2\sigma(I)$ reflections]	0.1049
$wR(F^2)$ (all data)	0.2935
Weights:	$w = [\sigma^2(F_o^2) + (0.2P)^2]^{-1}$ where $P = (F_o^2 + 2F_c^2)/3$
Goodness of fit	1.335
Final Δ_{max}/σ	0.003
$\Delta\rho$ (max; min) [e Å^{-3}]	0.52; -0.28
$\sigma(d_{(C–C)})$ [Å]	0.005 – 0.017

4 Supplementary References

(1) Pettersen, E. F.; Goddard, T. D.; Huang, C. C.; Couch, G. S.; Greenblatt, D. M.; Meng, E. C.; Ferrin, T. E. UCSF Chimera – A Visualization System for Exploratory Research and Analysis. *J. Comput. Chem.* **2004**, *25*, 1605–1612. https://doi.org/10.1002/jcc.20084.

(2) Voss, N. R.; Gerstein, M. 3V: Cavity, Channel and Cleft Volume Calculator and Extractor. *Nucleic Acid. Res.* **2010**, *38*, W555–W562. https://doi.org/10.1093/nar/gkq395.

(3) Ämmälahti, E.; Bardet, M.; Molko, D.; Cadet, J. Evaluation of Distances from ROESY Experiments with the Intensity-Ratio Method. *J. Magn. Reson. A* **1996**, *122*, 230–232. https://doi.org/10.1006/jmra.1996.0199.

(4) Bruno, I. J.; Cole, J. C.; Edgington, P. R.; Kessler, M.; Macrae, C. F.; McCabe, P.; Pearson, J.; Taylor, R. New Software for Searching the Cambridge Structural Database and Visualizing Crystal Structures. *Acta Crystallogr. Sect. B Struct. Sci.* **2002**, *58*, 389–397. https://doi.org/10.1107/S0108768102003324.

(5) Chemcraft - Graphical Software for Visualization of Quantum Chemistry Computations. https://www.chemcraftprog.com (accessed 2023-01-24).

(6) Knorr, R.; Ruhdorfer, J.; Böhrer, P.; Bronberger, H.; Räpple, E. Sterically Congested Molecules, 11. The 2,6-Diisopropylphenyl Substituent at C=C and C=N Groups. *Liebigs Ann. der Chemie* **1994**, *1994*, 433–438. https://doi.org/10.1002/jlac.199419940417.

(7) Li, G.; Han, A.; Pulling, M. E.; Estes, D. P.; Norton, J. R. Evidence for Formation of a Co-H Bond from $(H_2O)_2Co(DmgBF_2)_2$ under H_2: Application to Radical Cyclizations. *J. Am. Chem. Soc.* **2012**, *134*, 14662–14665. https://doi.org/10.1021/ja306037w.

(8) Dible, B. R.; Cowley, R. E.; Holland, P. L. Remote Substitution on N-Heterocyclic Carbenes Heightens the Catalytic Reactivity of Their Palladium Complexes. *Organometallics* **2011**, *30*, 5123–5132. https://doi.org/10.1021/om200349y.

(9) Hondros, C. J.; Aravindu, K.; Diers, J. R.; Holten, D.; Lindsey, J. S.; Bocian, D. F. Effects of Linker Torsional Constraints on the Rate of Ground-State Hole Transfer in Porphyrin Dyads. *Inorg. Chem.* **2012**, *51*, 11076–11086. https://doi.org/10.1021/ic301613k.

(10) Dhankhar, J.; González-Fernández, E.; Dong, C.-C.; Mukhopadhyay, T. K.; Linden, A.; Čorić, I. Spatial Anion Control on Palladium for Mild C–H Arylation of Arenes. *J. Am. Chem. Soc.* **2020**, *142*, 19040–19046. https://doi.org/10.1021/jacs.0c09611.

(11) Chen, H. C.; Chen, S. H. Diffusion of Crown Ethers in Alcohols. *J. Phys. Chem.* **1984**, *88*, 5118–5121. https://doi.org/10.1021/j150665a063.

(12) Zhao, Y. H.; Abraham, M. H.; Zissimos, A. M. Fast Calculation of van der Waals Volume as a Sum of Atomic and Bond Contributions and Its Application to Drug Compounds. *J. Org. Chem.* **2003**, *68*, 7368–7373. https://doi.org/10.1021/jo034808o.

(13) Schulze, B. M.; Watkins, D. L.; Zhang, J.; Ghiviriga, I.; Castellano, R. K. Estimating the Shape and Size of Supramolecular Assemblies by Variable Temperature Diffusion Ordered Spectroscopy. *Org. Biomol. Chem.* **2014**, *12*, 7932–7936. https://doi.org/10.1039/C4OB01373E.

(14) Pfeuffer-Rooschüz, J.; Schmid, L.; Prescimone, A.; Tiefenbacher, K. Xanthene[*n*]Arenes: Exceptionally Large, Bowl-Shaped Macrocyclic Building Blocks Suitable for Self-Assembly. *JACS Au* **2021**, *1*, 1885–1891. https://doi.org/10.1021/jacsau.1c00343.

(15) Beaudoin, D.; Rominger, F.; Mastalerz, M. Chirality-Assisted Synthesis of a Very Large Octameric Hydrogen-Bonded Capsule. *Angew. Chem. Int. Ed.* **2016**, *55*, 15599–15603. https://doi.org/10.1002/anie.201609073.

(16) Qiu, Y.; Song, B.; Pezzato, C.; Shen, D.; Liu, W.; Zhang, L.; Feng, Y.; Guo, Q.-H.; Cai, K.; Li, W.; Chen, H.; Nguyen, M. T.; Shi, Y.; Cheng, C.; Astumian, R. D.; Li, X.; Stoddart, J. F. A Precise Polyrotaxane Synthesizer. *Science* **2020**, *368*, 1247–1253. https://doi.org/10.1126/science.abb3962.

(17) Frisch, M. J.; Trucks, G. W.; Schlegel, H. B.; Scuseria, G. E.; Robb, M. A.; Cheeseman, J. R.; Scalmani, G.; Barone, V.; Petersson, G. A.; Nakatsuji, H.; Li, X.; Caricato, M.; Marenich, A. v; Bloino, J.; Janesko, B. G.; Gomperts, R.; Mennucci, B.; Hratchian, H. P.; Ortiz, J. v; Izmaylov, A. F.; Sonnenberg, J. L.; Williams-Young, D.; Ding, F.; Lipparini, F.; Egidi, F.; Goings, J.; Peng, B.; Petrone, A.; Henderson, T.; Ranasinghe, D.; Zakrzewski, V. G.; Gao, J.; Rega, N.; Zheng, G.; Liang, W.; Hada, M.; Ehara, M.; Toyota, K.; Fukuda, R.; Hasegawa, J.; Ishida, M.; Nakajima, T.; Honda, Y.; Kitao, O.; Nakai, H.; Vreven, T.; Throssell, K.; Montgomery Jr., J. A.; Peralta, J. E.; Ogliaro, F.; Bearpark, M. J.; Heyd, J. J.; Brothers, E. N.; Kudin, K. N.; Staroverov, V. N.; Keith, T. A.; Kobayashi, R.; Normand, J.; Raghavachari, K.; Rendell, A. P.; Burant, J. C.; Iyengar, S. S.; Tomasi, J.; Cossi, M.; Millam, J. M.; Klene, M.; Adamo, C.; Cammi, R.; Ochterski, J. W.; Martin, R. L.; Morokuma, K.; Farkas, O.; Foresman, J. B.; Fox, D. J. Gaussian 16 Revision C.01. (2016).

(18) Luchini, G.; Alegre-Requena, J. V.; Funes-Ardoiz, I.; Paton, R. S. GoodVibes: Automated Thermochemistry for Heterogeneous Computational Chemistry Data. *F1000Res* **2020**, *9*, 291. https://doi.org/10.12688/f1000research.22758.1.

(19) Grimme, S. Supramolecular Binding Thermodynamics by Dispersion-Corrected Density Functional Theory. *Chem. Eur. J.* **2012**, *18*, 9955–9964. https://doi.org/10.1002/chem.201200497.

(20) Barone, V.; Cossi, M. Quantum Calculation of Molecular Energies and Energy Gradients in Solution by a Conductor Solvent Model. *J. Phys. Chem. A* **1998**, *102*, 1995–2001. https://doi.org/10.1021/jp9716997.

(21) Cossi, M.; Rega, N.; Scalmani, G.; Barone, V. Energies, Structures, and Electronic Properties of Molecules in Solution with the C-PCM Solvation Model. *J. Comput. Chem.* **2003**, *24*, 669–681. https://doi.org/10.1002/jcc.10189.

(22) Liu, Y.; Sengupta, A.; Raghavachari, K.; Flood, A. H. Anion Binding in Solution: Beyond the Electrostatic Regime. *Chem* **2017**, *3*, 411–427. https://doi.org/10.1016/j.chempr.2017.08.003.

(23) Humphrey, W.; Dalke, A.; Schulten, K. VMD - Visual Molecular Dynamics. *J. Mol. Graph.* **1996**, *14*, 33–38.

(24) Legault, C. Y. CYLview20. Université de Sherbrooke (2020). Université de Sherbrooke. http://www.cylview.org.

(25) Hohenstein, E. G.; Sherrill, C. D. Wavefunction Methods for Noncovalent Interactions. *WIREs Comput. Mol. Sci.* **2012**, *2*, 304–326. https://doi.org/10.1002/wcms.84.

(26) Szalewicz, K. Symmetry-Adapted Perturbation Theory of Intermolecular Forces. *WIREs Comput. Mol. Sci.* **2012**, *2*, 254–272. https://doi.org/10.1002/wcms.86.

(27) Hohenstein, E. G.; Parrish, R. M.; Sherrill, C. D.; Turney, J. M.; Schaefer, H. F., III Large-Scale Symmetry-Adapted Perturbation Theory Computations via Density Fitting and Laplace Transformation Techniques: Investigating the Fundamental Forces of DNA-Intercalator Interactions. *J. Chem. Phys.* **2011**, *135*, 174107. https://doi.org/10.1063/1.3656681.

(28) Hohenstein, E. G.; Sherrill, C. D. Density Fitting and Cholesky Decomposition Approximations in Symmetry-Adapted Perturbation Theory: Implementation and Application to Probe the Nature of π-π Interactions in Linear Acenes. *J. Chem. Phys.* **2010**, *132*, 184111. https://doi.org/10.1063/1.3426316.

(29) Smith, D. G. A.; Burns, L. A.; Simmonett, A. C.; Parrish, R. M.; Schieber, M. C.; Galvelis, R.; Kraus, P.; Kruse, H.; Di Remigio, R.; Alenaizan, A.; James, A. M.; Lehtola, S.; Misiewicz, J. P.; Scheurer, M.; Shaw, R. A.; Schriber, J. B.; Xie, Y.; Glick, Z. L.; Sirianni, D. A.; O'Brien, J. S.; Waldrop, J. M.; Kumar, A.; Hohenstein, E. G.; Pritchard, B. P.; Brooks, B. R.; Schaefer, H. F., III; Sokolov, A. Yu.; Patkowski, K.; DePrince, A. E.; Bozkaya, U.; King, R. A.; Evangelista, F. A.; Turney, J. M.; Crawford, T. D.; Sherrill, C. D. Psi4 1.4: Open-Source Software for High-Throughput Quantum Chemistry. *J. Chem. Phys,* **2020**, *152*, 184108. https://doi.org/10.1063/5.0006002.

(30) Wadt, W. R.; Hay, P. J. *Ab Initio* Effective Core Potentials for Molecular Calculations. Potentials for Main Group Elements Na to Bi. *J. Chem. Phys.* **1985**, *82*, 284–298. https://doi.org/10.1063/1.448800.

(31) Roy, L. E.; Hay, P. J.; Martin, R. L. Revised Basis Sets for the LANL Effective Core Potentials. *J. Chem. Theory Comput.* **2008**, *4*, 1029–1031. https://doi.org/10.1021/ct8000409.

(32) Hay, P. J.; Wadt, W. R. *Ab Initio* Effective Core Potentials for Molecular Calculations. Potentials for K to Au Including the Outermost Core Orbitals. *J. Chem. Phys.* **1985**, *82*, 299–310. https://doi.org/10.1063/1.448975.

(33) Ehlers, A. W.; Böhme, M.; Dapprich, S.; Gobbi, A.; Höllwarth, A.; Jonas, V.; Köhler, K. F.; Stegmann, R.; Veldkamp, A.; Frenking, G. A Set of F-Polarization Functions for Pseudo-Potential Basis Sets of the Transition Metals Sc-Cu, Y-Ag and La-Au. *Chem. Phys. Lett.* **1993**, *208*, 111–114. https://doi.org/10.1016/0009-2614(93)80086-5.

(34) Rigaku Oxford Diffraction, CrysAlisPro Software System. (a) Rigaku Oxford Diffraction: Abingdon, Oxfordshire, England 2017. (b) Rigaku Corporation: Wroclaw, Poland 2018. (c) Rigaku Corporation: Oxford, UK 2021. (d) Rigaku Corporation: Oxford, UK 2022. Rigaku Oxford Diffraction: Abingdon, Oxfordshire, England.

(35) Coppens, P.; Leiserowitz, L.; Rabinovich, D. Calculation of Absorption Corrections for Camera and Diffractometer Data. *Acta Crystallogr.* **1965**, *18*, 1035–1038. https://doi.org/10.1107/S0365110X65002487.

(36) Sheldrick, G. M. SHELXT – Integrated Space-Group and Crystal-Structure Determination. *Acta Crystallogr. Sect. A Found. Adv.* **2015**, *71*, 3–8. https://doi.org/10.1107/S2053273314026370.

(37) Maslen, E. N.; Fox, A. G.; O'Keefe, M. A. Table 6.1.1.1. In *International Tables for Crystallography*; Wilson, A. J., Ed.; Kluwer Academic Publishers: Dordrecht, 1992; Vol. C, Pp 477–486.

(38) Stewart, R. F.; Davidson, E. R.; Simpson, W. T. Coherent X-Ray Scattering for the Hydrogen Atom in the Hydrogen Molecule. *J. Chem. Phys.* **1965**, *42*, 3175–3187.

(39) Ibers, J. A.; Hamilton, W. C. Dispersion Corrections and Crystal Structure Refinements. *Acta Crystallogr.* **1964**, *17*, 781–782. https://doi.org/10.1107/S0365110X64002067.

(40) Creagh, D. C.; McAuley, W. J. Table 4.2.6.8. In *International Tables for Crystallography*; Wilson, A. J., Ed.; Kluwer Academic Publishers: Dordrecht, 1992; Pp 219–222.

(41) Creagh, D. C.; Hubbel, J. H. Table 4.2.4.3. In *International Tables for Crystallography*; Wilson, A. J., Ed.; Kluwer Academic Publishers: Dordrecht, 1992; Pp 200–206.

(42) Sheldrick, G. M. Crystal Structure Refinement with SHELXL. *Acta Crystallogr. C Struct. Chem.* **2015**, *71*, 3–8. https://doi.org/10.1107/S2053229614024218.

(43) Macrae, C. F.; Sovago, I.; Cottrell, S. J.; Galek, P. T. A.; McCabe, P.; Pidcock, E.; Platings, M.; Shields, G. P.; Stevens, J. S.; Towler, M.; Wood, P. A. Mercury 4.0 : From Visualization to Analysis, Design and Prediction. *J. Appl. Crystallogr.* **2020**, *53*, 226–235. https://doi.org/10.1107/S1600576719014092.

(44) Farrugia, L. J. WinGX and ORTEP for Windows : An Update. *J. Appl. Crystallogr.* **2012**, *45*, 849–854. https://doi.org/10.1107/S0021889812029111.

(45) Spek, A. L. PLATON SQUEEZE: A Tool for the Calculation of the Disordered Solvent Contribution to the Calculated Structure Factors. *Acta. Crystallogr. C Struct. Chem.* **2015**, *71*, 9–18. https://doi.org/10.1107/S2053229614024929.

(46) Parsons, S.; Flack, H. D.; Wagner, T. Use of Intensity Quotients and Differences in Absolute Structure Refinement. *Acta Crystallogr. B Struct. Sci. Cryst. Eng. Mater.* **2013**, *69*, 249–259. https://doi.org/10.1107/S2052519213010014.

(47) Dolomanov, O. V.; Bourhis, L. J.; Gildea, R. J.; Howard, J. A. K.; Puschmann, H. OLEX2: A Complete Structure Solution, Refinement and Analysis Program. *J. Appl. Crystallogr.* **2009**, *42*, 339–341. https://doi.org/10.1107/S0021889808042726.
